# Direct access to tetrasubstituted cyclopentenyl scaffolds through a diastereoselective isocyanide-based multicomponent reaction†

**DOI:** 10.1039/d1sc04158d

**Published:** 2021-09-16

**Authors:** Vitor A. Fernandes, Rafaely N. Lima, Yoisel B. Broterson, Meire Y. Kawamura, Radell Echemendía, Alexander F. de la Torre, Marco A. B. Ferreira, Daniel G. Rivera, Marcio W. Paixão

**Affiliations:** Centre of Excellence for Research in Sustainable Chemistry (CERSusChem), Department of Chemistry, Federal University of São Carlos São Carlos São Paulo 13565-905 Brazil mwpaixao@ufscar.br; Departamento de Química Orgánica, Facultad de Ciencias Químicas, Universidad de Concepción Concepción Chile; Faculty of Chemistry, University of Havana La Habana Cuba

## Abstract

An efficient strategy combining the stereocontrol of organocatalysis with the diversity-generating character of multicomponent reactions is described to produce structurally unique, tetrasubstituted cyclopentenyl frameworks. An asymmetric Michael addition–hemiacetalization between α-cyanoketones and α,β-unsaturated aliphatic aldehydes was performed for constructing cyclic hemiacetals, which were next employed as chiral bifunctional substrates in a new diastereoselective intramolecular isocyanide-based multicomponent reaction. This approach furnished a diversity of structurally complex compounds – including peptidomimetics and natural product hybrids in high stereoselectivity (up to >99% ee and up to >99 : 1 dr) and in moderate to high yields.

## Abstract 2

Simple and available reagents are combined in this new three-component isocyanide-based multicomponent reaction providing an interesting and straightforward way to prepare complex and highly functionalized cyclopentenyl rings.

## Graphical abstract

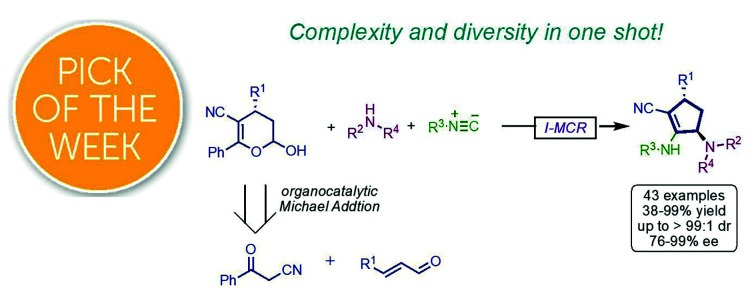

## Introduction

**Fig. 1:**
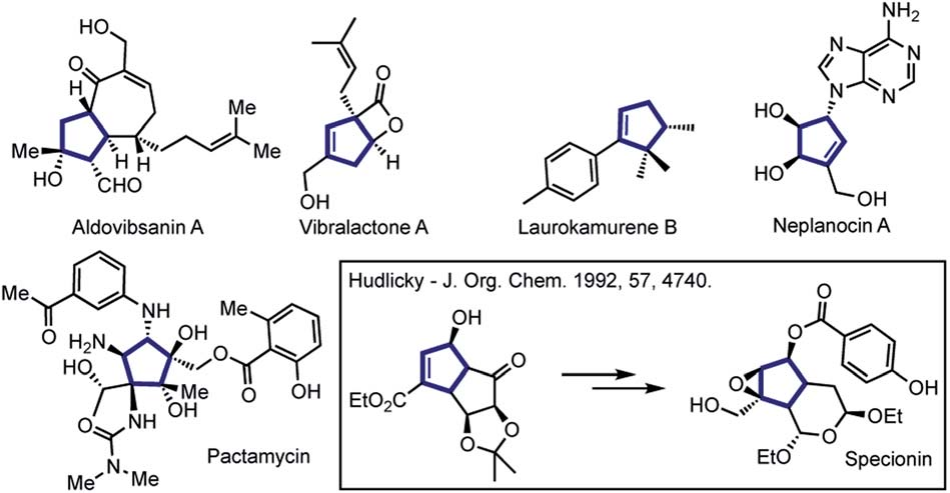
Representative synthetic and natural bioactive molecules containing polysubstituted cyclopentene or cyclopentane frameworks.

Functionalized five-membered carbocyclic frameworks are special core units present in numerous natural products and biologically relevant molecules.^1–4^ Cyclopentenes have also been widely used as versatile building blocks in the total synthesis of complex bioactive molecules (Fig. 1).^5–10^

Owing to the predominance of polysubstituted cyclopentene and cyclopentane skeletons in bioactive compounds, several strategies have been developed for the stereoselective synthesis of such functionalized rings.

**Scheme 1:**
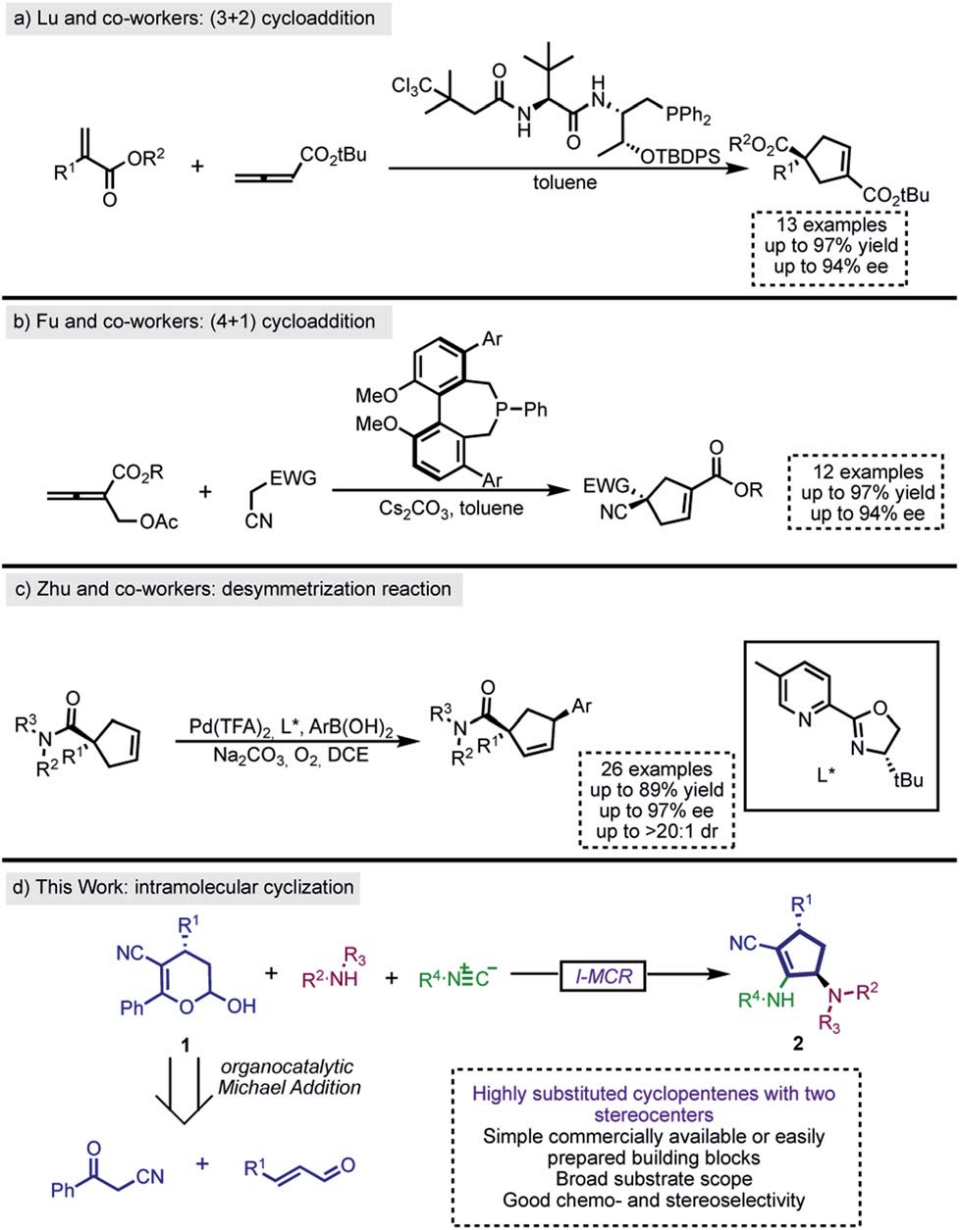
Recent examples of stereoselective synthesis of cyclopentenes.

Among the numerous methodologies for the synthesis of functionalized cyclopentenes, the phosphine-catalyzed (3 + 2) annulation of allenoates with electron-deficient olefins, first reported by Lu and co-workers in 1995,^11^ stands out as the most explored strategy.^12^ Therefore, there are several phosphine (3 + 2) annulation methodologies in the literature, including their asymmetric variants.^13^ Recently, Lu's group described a phosphine-catalyzed (3 + 2) annulation of electron-poor allenes with activated alkenes for the construction of functionalized cyclopentenes bearing quaternary centers^14^ (Scheme 1a). Besides, Fu,^15^ Lu,^16,17^ Miller^18^ and others^19^ subsequently developed and expanded the scope of the enantioselective intramolecular formal (3 + 2) cycloaddition between allenoates and activated alkenes to create fused chiral ring scaffolds. Moreover, distinct formal (3 + 2) cycloadditions which have also been useful for the construction of enantioenriched cyclopentene derivatives employed N-heterocyclic carbene (NHC)-catalyzed reactions,^20–22^ metal carbenoids,^23^ and the ring opening of cyclopropanes.^24–26^

Although less explored, formal (4 + 1) cycloaddition is also a suitable strategy and, according to recent literature,^27–29^ phosphine^30^ and metal catalysis^31^ are versatile and powerful approaches for the construction of these five membered ring systems. In 2014, Fu and co-workers disclosed a biphenyl phosphine-catalyzed enantioselective (4 + 1) annulation of allenoates with Michael donors (Scheme 1b).^32^

The enantioselective synthesis of pentacyclic cores can also be achieved by desymmetrization reactions.^33^ Although there are other approaches for performing such transformation,^34,35^ the most explored is certainly the enantioselective Heck reaction of *C*_2_-symmetrical cyclopentenes. Correia,^36–41^ Toste^42^ and others^43–45^ have explored and expanded the scope of this type of transformation over the last few years. In this context, Zhu and co-workers recently reported a palladium-catalyzed oxidative Heck reaction between 4,4-disubstituted cyclopentenes and aryl boronic acids (Scheme 1c).^45^

While these methods provide valuable enantioenriched cyclopentane, they have limitations that include the multistep synthesis of starting materials and the limited variation of input elements contributing to increased skeletal diversity. These factors may restrict applications in skeletal diversification strategies such as those required in modern drug discovery approaches. Consequently, the design of new transformations enabling the rapid construction of structurally complex carbocycles from simple and available starting materials is of interest in contemporary organic chemistry.^46^

Multicomponent reactions (MCRs) have proven to be among the most useful processes for the rapid generation of structural diversity and complexity.^47^ Among them, isocyanide-based MCRs (I-MCRs) stand out as powerful approaches to produce highly functionalized natural product-like molecules with high chemical efficiency, convergence and atom economy.^48,49^ Unfortunately, a limitation of I-MCRs is their poor stereoselectivity, which often leads to mixtures of stereoisomers that are suitable in the drug discovery process but not in lead optimization and development steps.

As part of the ongoing interest in developing diastereoselective I-MCRs towards chiral and biologically relevant scaffolds,^50–52^ we report the discovery of a novel 4-center-3-component reaction (*i.e.*, a novel Ugi-4C-3CR) incorporating accessible hemiacetals^53^ as a chiral bifunctional substrate, an amine, and an isocyanide component. Novel tetrasubstituted cyclopentene adducts containing two new stereogenic centers are obtained with excellent enantio- and diastereoselectivity through this methodology.

The methodological tactic enabling the discovery of this interesting process followed two main aspects: (a) the early synthesis of an enantiomerically enriched building block bearing two sites of reactivity in a subsequent I-MCR, and (b) the design of a multicomponent event proceeding *via* conformationally restricted intermediates to better control the stereochemical outcome.

Herein we disclose the results of such a new intramolecular multicomponent process delivering complex and diverse chiral scaffolds – including peptidomimetics and sugar hybrids. Computational studies based on density functional theory (DFT) calculations provided valuable insights into the possible reaction mechanisms and the selectivities (chemo- and stereoselectivity) of this new procedure.

## Results and discussion

Our research group recently developed a powerful Ugi-type MCR for the stereoselective synthesis of tetrahydropyridines (THP) by employing aryl-containing hemiacetals as bifunctional substrates.^54^ The previous approach also showed broad substrate scope for both the amino and isocyanide components. However, only aryl-containing hemiacetals could be incorporated in the reaction sequence, limiting application in the parallel construction of a more diverse library. In an attempt to broaden the substrate scope, we sought to employ hemiacetals bearing alkyl substituents, which upon reaction with a primary or secondary amine generated unexpected cyclopentenyl scaffolds in good yields and great stereoselectivity. The discovery of this unexpected reaction product meant a different reaction pathway and led us to address the versatility and efficiency of this new multicomponent procedure. An important focus was placed on studying the reactivity and stereoselectivity of this intriguing transformation.

**Scheme 2:**
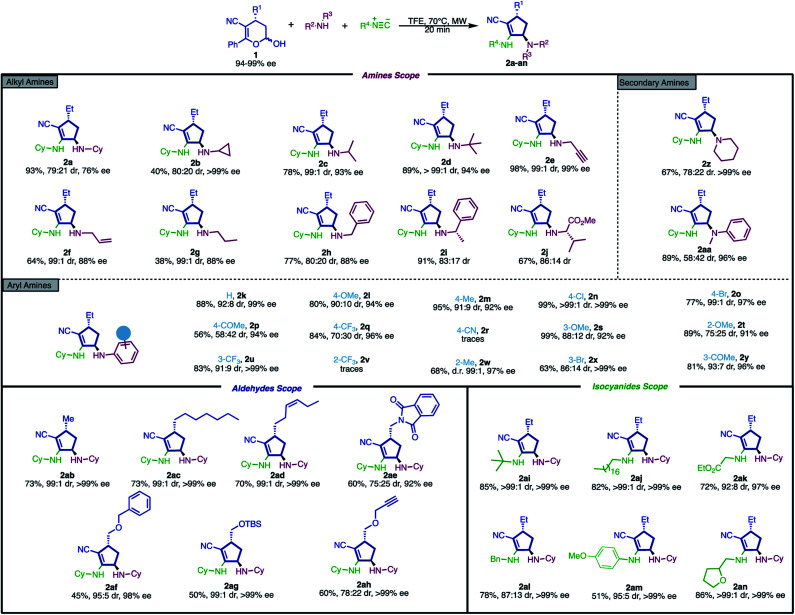
Substrate scope for the synthesis of cyclopentenyl amines. Reaction conditions: hemiacetal 1 (0.15 mmol, 1.0 equiv.), amine (1.0 equiv.) and isocyanide (1.0 equiv.) in TFE (0.3 mL) at 70 °C under microwave irradiation (300 W). TFE = 2,2,2-trifluoroethanol. The diastereoisomeric ratio (dr) was determined by ^1^H NMR analysis.

We began our investigation by subjecting hemiacetal 1a to a short optimization, using *tert*-butyl amine and cyclohexyl isocyanide as model substrates for this study. The best reaction conditions were achieved by employing 1,1,1-trifluoroethanol (TFE) as a solvent under microwave irradiation (300 W) at 70 °C for 20 min, affording the cyclopentene 2d in high yield and excellent stereoselectivity (Scheme 2, 89% yield, 94% ee, >99 : 1 dr). As a consequence of this satisfactory initial result, we decided to apply these conditions to exploit the generality and limitations. In this context, the influence of the amine component was selected to be the first evaluated component in the reaction protocol. To broaden the substrate scope, a wide range of amines with distinct electronic and steric properties were chosen (Scheme 2), generating a large set of products in good to high yields with excellent stereoselectivity (28 examples, 38–99%, up to >99% ee, up to >99 : 1 dr).

The reaction proved to be efficient for a wide variety of alkylamines – linear, branched, sterically hindered, cyclic and acyclic ones – affording products 2a–g in moderate to high yields and good stereoselectivities (Scheme 2, first section). Additionally, propargyl (2e) and allyl (2f) amines, which are useful moieties for further orthogonal derivatization, *e.g.*, cross-coupling^55^ and metal-catalyzed reactions,^56–58^ as well as bioconjugation, were also amenable to the reaction conditions (2e and 2f in 98% yield, 99 : 1 dr, 99% ee and 64% yield, 99 : 1 dr, 88% ee, respectively). Benzyl amine is also a suitable substrate, affording 2h in 73% yield with 80 : 20 diastereoselectivity and 86% ee. Moreover, α-methylbenzylamine afforded the desired products in both excellent yield and good diastereoselectivity (2i in 91% yield, 83 : 17 dr). Interestingly, l-valine methyl ester displayed promising results in both yield and stereoselectivity (2j in 67% yield, 86 : 14 dr), demonstrating that amino acids can be used as suitable substrates in this procedure.

We were pleased to find that less nucleophilic substituted anilines could also be applied in this transformation, generating products in good to excellent yields with high stereoselectivity (56–99% yield, up to 99 : 1 dr and up to 99% ee). Anilines bearing electron-donating (2k–m), halogen (2n and 2o) and some electron-withdrawing (2p and 2q) groups at the *para*-position were also found to be competent substrates for this multicomponent reaction. However, only traces of the product were obtained when employing a strong electron-withdrawing group, *e.g.*, cyano, as an aniline substituent, likely due to its reduced nucleophilicity. Furthermore, *meta*- and *ortho*-substituted anilines efficiently underwent this transformation (2s–y, 63–99% yield and good stereoselectivities). However, when *ortho*-trifluoromethyl aniline was employed as a substrate, no formation of cyclopentene 2v was observed. This result can be explained by the hyperconjugative electron-withdrawing nature of the trifluoromethyl group. The *ortho*-methoxy (2t) and -methyl (2w) derivatives were synthesized in good yields (89% and 68% yield, respectively) and selectivities (75 : 25 dr, 91% ee and 99 : 1 dr, 97% ee, respectively). Thus, by analyzing this set of results, it was possible to conclude that the reaction outcome is not significantly influenced by steric hindrance.

We next turned our attention to evaluate the scope of the amino component for secondary amines. To our delight, piperidine and *N*-methylaniline proved to be compatible with the presented method, affording the tertiary amine products (2z–2aa) in good yields (67–89%), although with moderate stereoselectivity (up to 78 : 22).

Having examined the reaction scope for the amino component, we focused on defining the scope of the bifunctional component. To our satisfaction, a variety of products with different alkyl substituents were produced in moderate to good yields and excellent stereoselectivities (2ab–2ah, 45–73% and up to 99 : 1 dr). The versatility of the method to install functionalized substituents at that position is a clear advantage, as they can be used for further derivatization of the chiral cyclopentenyl scaffold.

The reaction displayed no influence concerning the length of the carbon chain, presenting good yields and stereoselectivity for methyl (2ab, 73%, 99 : 1 dr, >99% ee), heptyl (2ac, 73%, 99 : 1 dr, 99% ee) and (*Z*)-hex-3-enyl (2ad, 70%, 99 : 1 dr, >99% ee) substituted hemiacetals. Subsequently, hemiacetals bearing electronegative heteroatoms (*e.g.*, nitrogen and oxygen) in the side chain show diminished yields under the optimized conditions (2ae–ah, 45–60%, up to 99 : 1 dr, up to >99% ee). These *O*- and *N*-substituted products are far more interesting as synthetic intermediates because they can be deprotected into free amine (2ae) and hydroxy groups (2af and 2ag), enabling additional functionalization *via* conventional synthetic methods.^59^ Likewise, product 2ah, which contains a terminal alkyne, can be easily employed as a substrate in a wide range of chemical transformations, such as Sonogashira coupling^60^ and Click reactions.^61^ Furthermore, it is noteworthy that this method was compatible with all protecting groups employed (*i.e.*, Bn, TBS and Phth), which is, from the synthetic point of view, a desirable aspect due to the ubiquitous presence of protecting groups in the total synthesis of complex molecules.^62^

Aiming to better cover the reaction, we evaluated the third component of this reaction: the isocyanide. Under optimized reaction conditions, five cyclopentenyl derivatives were accessed in moderate to high yields with good diastereo- and enantioselectivities. The method showed good tolerance to both bulky and long alkyl chains, displaying high yields and excellent diastereoselectivity (2ai and 2aj in 85% yield, 99 : 1 dr and 99% ee, and 82% yield, >99 : 1 dr and >99% ee). Glycine-derived isocyanide afforded the desired product 2ak in good yield and excellent stereoselectivity (72% yield, 92 : 8 dr and 97% ee). Although there is a slight decrease in the stereoselectivity (87 : 13 dr and >99% ee) upon using benzyl isocyanide, product 2al was isolated in 78% yield. An aromatic isocyanide was also evaluated, giving rise to product 2am in 51% yield and excellent stereoselectivity (95 : 5 dr and >99% ee). Moreover, the tetrahydrofuran moiety was demonstrated to be compatible with this transformation, generating product 2an in 86% yield and excellent selectivity (99 : 1 dr and >99% ee).

**Scheme 3:**
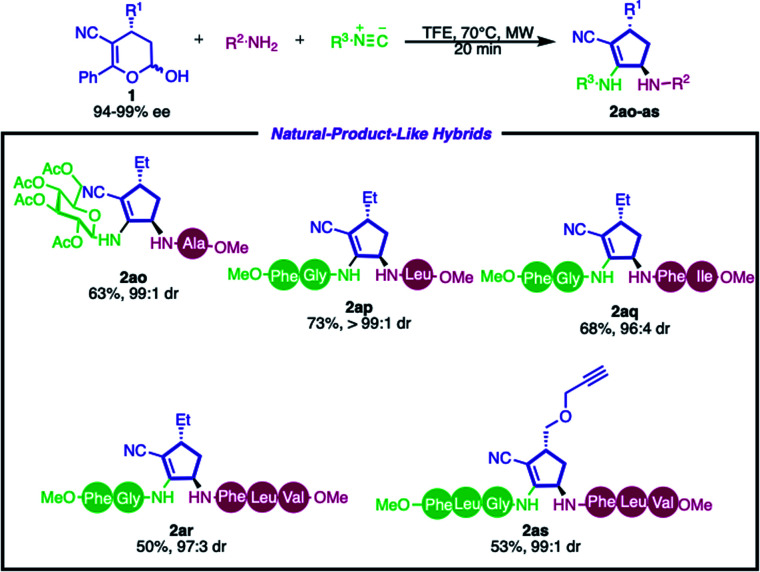
Stereoselective synthesis of cyclopeptene-peptide and carbohydrate hybrids. Reaction conditions: hemiacetal 1 (0.15 mmol, 1.0 equiv.), amine (1.0 equiv.) and isocyanide (1.0 equiv.) in TFE (0.3 mL) at 70 °C under microwave irradiation (300 W). TFE = 2,2,2-trifluoroethanol. The diastereoisomeric ratio (dr) was determined by ^1^H NMR analysis.

To demonstrate the synthetic applicability of this methodology, we focused our efforts on the synthesis of complex molecular hybrids. We further investigated the incorporation of natural product fragments such as peptides and saccharides into the cyclopentenyl core. As depicted in Scheme 3, glucose, di- and tripeptides containing the cyclopentenyl scaffold were synthesized by employing the enantioenriched hemiacetal 1 in good yields and excellent diastereoselectivities (2ao–as, 50–73%, from 96 : 4 to >99 : 1 dr).

These examples demonstrate the feasibility of the developed method to obtain complex architectures, which shows the great potential of this approach for late-stage modification of peptides.^63^ Another important feature is that this multicomponent strategy enables the side-specific insertion of rigid cyclopentenyl structures into peptide side chains, which would modulate the conformation, dynamics, and proteolytic susceptibility of native peptides and, consequently, provide a foundation for sophisticated molecular function.^64–66^ Furthermore, product 2as can be used as a substrate in cycloaddition reactions with azides – a common strategy employed in bioconjugation^61^ – showing that this method can provide a simple, fast and efficient route to link peptides with probes.

Taking advantage of the robustness and practicality of this methodology, we envisioned a one-pot continuous flow procedure (Scheme 5), in which the hemiacetal generated by the organocatalytic Michael addition was directly used in the MCR. After optimization, product 2d was isolated with high yield and excellent selectivity (83% yield, >99 : 1 dr and >99% ee).

**Scheme 4:**
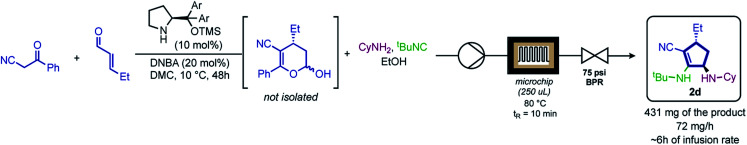
Scale-up continuous flow experiment; DNBA = 3,5-dinitrobenzoic acid; DMC = dimethylcarbonate; Ar = 3,5-(CF_3_)_2_C_6_H_3_.

Next, a 7-center-5-component reaction (7C-5CR) was performed (Scheme 4).

**Scheme 5:**
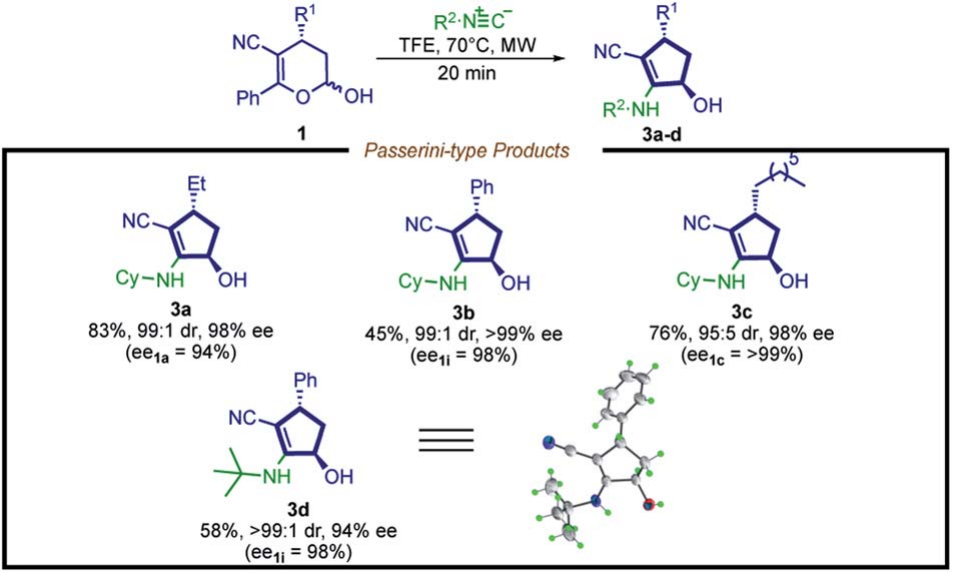
Substrate scope for the synthesis of cyclopentenols. Reaction conditions: hemiacetal 1 (0.15 mmol, 1.0 equiv.) and isocyanide (1.0 equiv.) in TFE (0.3 mL) at 70 °C under microwave irradiation (300 W). TFE = 2,2,2-trifluoroethanol. The diastereoisomeric ratio (dr) was determined by ^1^H NMR analysis.

Aiming to extend the scope of this method, we performed the reaction with hemiacetal 1a under the same previously presented conditions, except in the absence of amine. To our satisfaction, a Passerini-type product 3a was obtained in good yield and high stereoselectivity (Scheme 4, 3a, 83%, 99 : 1 dr and 98% ee). Inspired by this result, we then evaluated a narrow scope of substrates for this transformation (Scheme 5).

In contrast to the limitation described for the synthesis of cyclopentenyl amines, hemiacetals bearing an aryl moiety at the R^1^ position are compatible with this new methodology, affording product 3b in moderate yield and excellent stereoselection (45% yield, 99 : 1 dr, >99% ee).

A possible explanation is that the weaker nucleophilic nature of the oxygen increases the energetic barrier of the Mumm-type rearrangement, disfavoring the formation of the 3,4-dihydro-2*H*-pyran scaffold byproduct. It is possible to note that this reaction shows potential for further improvement, plus the 2-cyclopentenol derivatives are valuable synthetic intermediates widely employed in synthetic programs.^67–70^ Moreover, the structure of the product 3d was unambiguously confirmed by X-ray analysis, showing a *trans* configuration, which is consistent with the NOESY ^1^H NMR analysis of compound 2d (for details, see the ESI†).

**Scheme 6:**
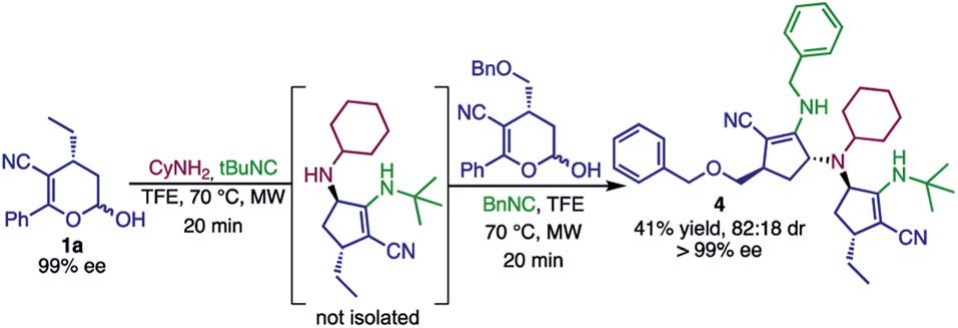
Synthesis of tertiary amines *via* a 7C-5CR reaction.

We envisioned that products 2 could be further employed as the amino component in a new Ugi-4C-3CR. To this end, product 2d was obtained by reacting hemiacetal 1a, cyclohexylamine and *tert*-butyl isocyanide in a one-pot manner. Compound 2d was used as the substrate in a second Ugi-4C-3CR with hemiacetal 1f and benzyl isocyanide, generating the tertiary amine 4 in 41% yield (Scheme 6). Once a large library of compounds is synthesized with simples substrates, this strategy becomes an interesting approach for the synthesis of chiral bulky tertiary amines, widely used in asymmetric organocatalysis^71,72^ and as ligands for asymmetric transition metal catalysts.^73^

**Scheme 7:**
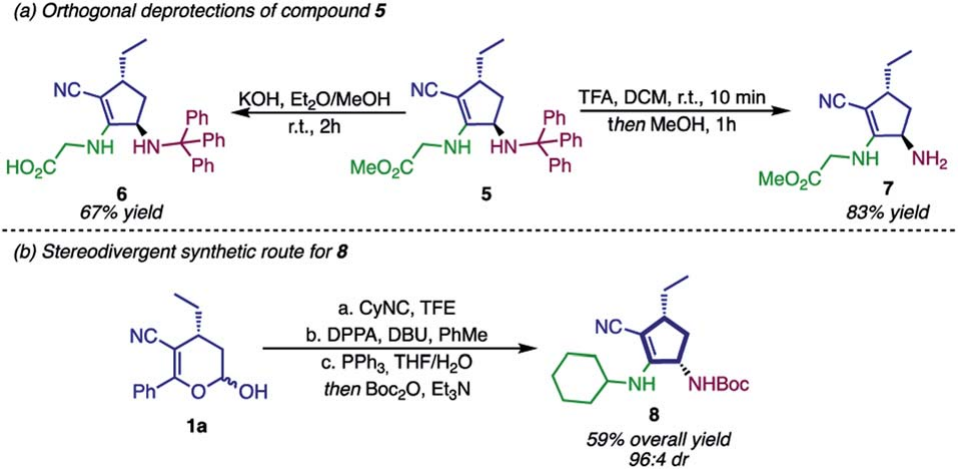
(a) Orthogonal deprotection of 5; (b) stereodivergent synthetic route for 8.

To further show the synthetic potential of this strategy, compound 5 was submitted to orthogonal deprotection, affording the cyclopentene derivatives 6 and 7 (Scheme 7a). First, hydrolysis of the ester in alkaline media gave rise to the carboxylate 6. Thereafter, the deprotection of the *N*-trityl was performed, affording product 7 containing a primary free amine group that can be used in a wide range of chemical reactions.^74–78^

As depicted in Scheme 7a, both of these structures are peptidomimetics, and therefore they can be employed as substrates in peptide chemistry. While compound 6 is used as a C-terminal dipeptide mimetic, 7 would react in the N-terminal position. This example of orthogonal deprotection indicates that compound 5 and derivatives can be employed – regardless of the strategy – in solid-phase peptide synthesis. As an attempt to broaden the applicability of this methodology, we envisioned that the *syn*-diastereoisomer of cyclopentyl amines 2 could be accessed by using Passerini-type cyclopentenols 3 as substrates (Scheme 7b). After a sequence of Mitsunobu reaction, Staudinger reduction and Boc protection, compound 8 could be synthesized in excellent selectivity (94 : 6 dr), although in moderate yield (59%).

**Scheme 8:**
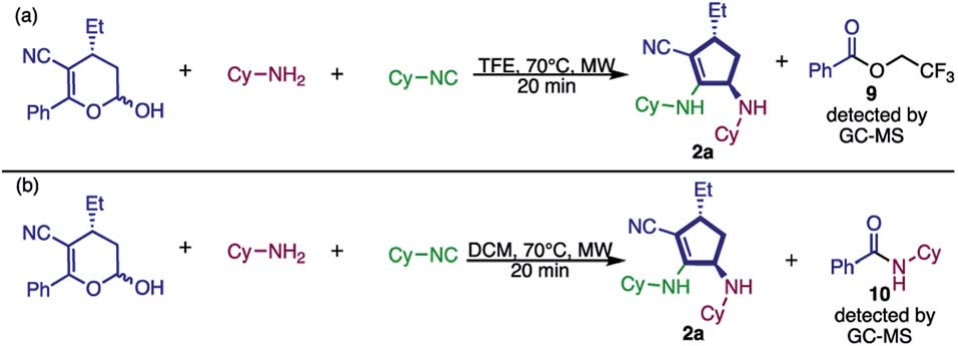
Experiments performed to gain some insights into the mechanism.

To gain some insights into the possible mechanism of this new approach, some experiments were performed. Initially, the reaction was carried out under the same reaction conditions described and the crude mixture was analyzed by GC-MS, through which 2,2,2-trifluoroethyl benzoate (9, Scheme 8a) was detected.

The presence of this species drove us to the conclusion that the solvent – 2,2,2-trifluoroethanol – reacts, as a nucleophile, with the ketone moiety present in the substrate. Therefore, we substituted the solvent for dichloromethane (a non-nucleophilic solvent), aiming at the isolation of any intermediate of this transformation. However, under these conditions (Scheme 8b) we observed product formation and instead of by-product 9, the generation of *N*-cyclohexylbenzamide (10) was detected. These results prove that the nucleophilic attack during the elimination of the ketone portion is not a rate determining step (RDS). Also, we assume that the nucleophilicity of the reagent has no influence because both TFE and cyclohexylamine, which are very distinct in nucleophilicity, can play this role.

**Fig. 2:**
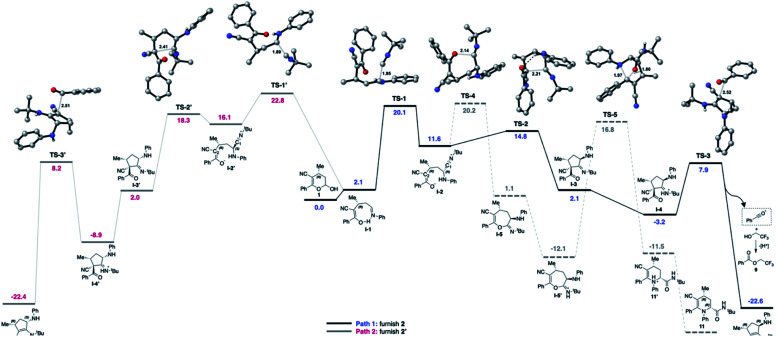
Gibbs corrected reaction energy profile in kcal mol^−1^ and transition state geometries at the B3LYP-D3/def2-TZVP [TFE].

Furthermore, a theoretical investigation using DFT calculations of the most important elementary steps in the MCR was conducted to elucidate the factors that control the observed diastereoselectivity (up to 99 : 1 dr) towards the obtained products (Fig. 2). As we described earlier,^54^ the multicomponent sequence begins with amine addition to the hemiacetal 1 with formation of the imine I-1, featuring an intramolecular hydrogen bond. This cyclic conformation introduces the conformational rigidity for the high dr obtained. The diastereoselectivity is controlled by the attractive non-covalent interactions between the isocyanide and the conjugated enol π-system (Fig. S12 in the ESI†), favoring the *Si*-face attack on the imine I-1 through the rate- and diastereoselectivity-determining transition state TS-1 (*Si*-face) over TS-1′ (*Re*-face). The computational study shows that TS-1 is 2.7 kcal mol^−1^ lower in energy than TS-1′, giving a theoretical diastereoselectivity of 98 : 2 at 70 °C in favor of the major diastereoisomer, in excellent agreement with experimental results. Next, the most intriguing aspect of this transformation is the unexpected cyclization to furnish the cyclopentenyl moiety 2. After the formation of intermediate I-2, it was expected that the oxygen would attack the electrophilic carbon (C1) of the nitrilium ion *via*TS-4, ultimately affording the possible product 11.^54^ However, the attack of the enolate by the α-ketonic carbon (C2) *via*TS-2/TS-2′ (ΔΔ*G*^‡^ = 3.2 and 2.1 kcal mol^−1^) is much lower in energy compared with TS-4 (ΔΔ*G*^‡^ = 8.1 kcal mol^−1^), corroborating the experimental exclusive formation of 2. The probable origin of this energy difference is associated with the stabilizing intramolecular hydrogen bond featured in TS-2 (N–H⋯O=C, 2.06 Å). Analyzing the competitive attacks of the enolate by the α-ketonic carbon (C2) *via*TS-2 and TS-2′, both have similar geometries except for the disposition of the NH-Ph group. The axial arrangement in the TS-2 conformation brings together the atoms involved in the mentioned hydrogen bond, which is strong enough to overcome the repulsion exerted by the NH-Ph group. TS-2 is 3.5 kcal mol^−1^ more stable than TS-2′, which has an NH-Ph substituent in a pseudo-equatorial position. This very compact enolate transition state is probably very susceptible to energetic variations due to backbone substitutions and conformations.

Probably for steric reasons, the less hindered TS-4 is preferred over TS-2 when using the slightly bulkier –Ph substituent in the R^1^ position, justifying the reactivity reported in this work compared to the one reported previously.^54^

Once intermediate I-3 is formed, the protonation of the imine favors the acyliminium cleavage by TS-3 (ΔΔ*G*^‡^ = 10.8 kcal mol^−1^) to furnish product 2 and the acylium ion (see Scheme S1 in the ESI† for further details). This step is somehow in accordance with the experimental observation of 9 and 10 (Scheme 8), resulting from the addition of a nucleophilic species to the acylium ion. The acyl transfer mechanism involving the nucleophilic attack of TFE, as well as non-catalyzed pathways were explored but presented much higher energy barriers (see the ESI† for further details).

## Conclusions

In summary, we have reported a new isocyanide-based multicomponent reaction using easily accessible hemiacetals as bifunctional substrates. This method displayed good functional group tolerance and high stereoselectivity, and a broad scope of substrates could be employed. Furthermore, this approach furnished a diversity of structurally complex compounds – including peptidomimetics and natural product hybrids. A DFT mechanistic investigation elucidated the features behind the unexpected 5-*exo*-dig cyclization, which opens new avenues for developing new cascade processes.

## Data availability

All the experimental and computational data have been included in the ESI.†

## Author contributions

V. A. F., R. N. L., Y. B. B. and R. E. contributed to the investigation; M. Y. K. and M. A. B. F. contributed to the formal analysis; V. A. F., A. F. T., D. G. R. and M. W. P. contributed to the conceptualization, design and writing of the paper.

## Conflicts of interest

There are no conflicts to declare.

## Supplementary Material

SC-012-D1SC04158D-s001

SC-012-D1SC04158D-s002

SC-012-D1SC04158D-s003
