# Supplementary material for: Direct access to tetrasubstituted cyclopentenyl scaffolds through a diastereoselective isocyanide-based multicomponent reaction
Source: Chem Sci. 2021 Sep 16;12(48):15862–9. doi: 10.1039/d1sc04158d (PMC8672720; doi:10.1039/d1sc04158d)
Supplement: SC-012-D1SC04158D-s001 [file SC-012-D1SC04158D-s001.pdf]

Electronic Supplementary Material (ESI) for Chemical Science.
This journal is © The Royal Society of Chemistry 2021

Supplementary information II– Computational Studies

Direct Access to Tetrasubstituted Cyclopentenyl Scaffolds through Diastereoselective Isocyanide-based Multicomponent Reactions

Vitor A. Fernandes,[a] Rafaely N. Lima,[a] Yoisel B. Broterson,[a] Meire Y. Kawamura,[a] Radell Echemendía, [a,c] Alexander F. de la Torre,[b] Marco A. B. Ferreira,*[a] Daniel G. Rivera*[c] and Marcio W. Paixão*[a]

a. Centre of Excellence for Research in Sustainable Chemistry (CERSusChem), Department of Chemistry, Federal University of São Carlos – UFSCar, Rodovia Washington Luís, km 235 - SP-310 - São Carlos - São Paulo - Brazil -13565-905. E-mail: mpwpaixao@ufscar.br, marco.ferreira@ufscar.br

b. Facultad de Ciencias Químicas, Universidad de Concepción, Chile.

c. Center for Natural Products Research, Faculty of Chemistry, University of Havana, Zapata y G, 10400, Havana, Cuba. E-mail: dgr@fq.uh.cu

Table of Contents

A. Computational Details S2
B. Discussion of Computational Results S3
Scheme S1. Summary of all investigated intermediates and transition states S5
Figure S1. Calculated structures of found transition states for **B3LYP-D3/def2-tzvp/IEF-PCM**. Distances in angstrom S6
Table S1: Energies (in Hartree) for studied compounds at **B3LYP-D3/def2-tzvp/IEF-PCM**.......... S7
Table S2: Single point Electronic Energies (in Hartree) for studied compounds at different levels of theory for **B3LYP-D3/def2-tzvp/IEF-PCM** geometries S8
Table S3: Gibbs free Energies (in Hartree) for studied compounds at different levels of theory for **B3LYP-D3/def2-tzvp/IEF-PCM** geometries S9
Table S4. Reaction free energy profiles for all investigated levels of theory**.** Relative Gibbs free Energies (in kcal mol^{-1}) for studied compounds at different levels of theory for **B3LYP-D3/def2-tzvp/IEF-PCM** geometries. Reaction barrier in bracket S10
Figure S2. IRC for **TS-1** at **B3LYP-D3/def2-tzvp/IEF-PCM**.......... S11
Figure S3. IRC for **TS-1'** at **B3LYP-D3/def2-tzvp/IEF-PCM**.......... S11
Figure S4. IRC for **TS-2** at **B3LYP-D3/def2-tzvp/IEF-PCM**.......... S12
Figure S5. IRC for **TS-2'** at **B3LYP-D3/def2-tzvp/IEF-PCM**.......... S12
Figure S6. IRC for **TS-3** at **B3LYP-D3/def2-tzvp/IEF-PCM**.......... S13
Figure S7. IRC for **TS-3'** at **B3LYP-D3/def2-tzvp/IEF-PCM**.......... S13
Figure S8. IRC for **TS-4** at **B3LYP-D3/def2-tzvp/IEF-PCM**.......... S14
Figure S9. IRC for **TS-5** at **B3LYP-D3/def2-tzvp/IEF-PCM**.......... S14
Figure S10. IRC for **TS-6** at **B3LYP-D3/def2-tzvp/IEF-PCM**.......... S15
Figure S11. IRC for **TS-7** at **B3LYP-D3/def2-tzvp/IEF-PCM**.......... S15
Table S5: Energies (in Hartree) for conformers of structure **I-1** and **I-1'** at **B3LYP-D3/def2-tzvp/IEF-PCM**.... S16
Table S6: Energies (in Hartree) for conformers of structure **I-2** and **I-2'** at **B3LYP-D3/def2-tzvp/IEF-PCM**.... S17
Figure S12. NCI analysis for **TS-1** and **TS-1'** at **B3LYP-D3/def2-TZVP**.......... S18
C. Cartesian Coordinates S19
D. References S35

A. Computational Details

The DFT calculations were performed with Gaussian 16[1] suit of programs. Full optimizations were conducted using B3LYP-D3/def2-tzvp level of theory. The inclusion of the solvent effect for all optimizations was done using IEF-PCM [TFE]. All cartesian coordinates are supplied in this SI. Visualizations were done with the beta version of CYLview.[2] Frequency calculations at 295.15 K (1 atm) ensured that the stationary points represent either minima (no imaginary frequency) or transition states (single imaginary frequency) on the potential-energy surface, furnishing also the zero-point vibrational energies, the thermal and entropic correction from which the Gibbs free energies were determined. The IRC calculations were done ensuring that each transition state connects reagents and products. The Non Covalent Interaction (NCI) analysis was carried out with the NCIplot 3.0 software,[3] with VMD[4] as visual interface.

The conformational searches were done in gas phase using the Monte Carlo (MCMM) method as implemented as implemented in MacroModel (Version 9.9).[5] The energy minimization was carried out using the Polak-Ribiere Conjugate Gradient (PRCG), and the MMFF force field, using dielectric constant-dependent electrostatics (e=1) and normal cut-off points to model the non-bonded interactions. All heavy atoms and hydrogens at heteroatoms were included in the test for redundant conformers, using the default cutoff (maximum atom deviation) of 0.5 Å. All rotatable single bonds were included in the conformational search. The energy window for saving new structures was 5 kcal/mol relative to the current global minimum, using a maximum number of steps of 30000 and 1000 steps per rotable bond. Each search was continued until the global energy minima were found at least 10-20 times, thus giving confidence that all the relevant conformers had been found.

To refine the electronic energy, single-point calculations were performed using the B3LYP-D3/def2-tzvp [IEF-PCM] geometries. The functionals B3PW91, B97D3, M062X and WB97XD was explored. The Grime D3[6] empirical dispersion was used for B3LYP and B97 functional.

B. Discussion of Computational Results

Computational studies were initiated in order to investigate the reaction energy profile for the MCR. The relative energies and geometries for intermediates and transition states were calculated at **B3LYP-D3/def2-tzvp/IEF-PCM**. In the Scheme S1, it is shown the summary of all investigated intermediates and transition states and relative Gibbs Free energies. In Figure S1, we represent the calculated structures of found transition states. In Table S1 are the absolute energy, Gibbs free energy corrections and the imaginary frequency for transition states **B3LYP-D3/def2-tzvp/IEF-PCM**. In Table S2 are the single point electronic energies at different levels of theory for **B3LYP-D3/def2-tzvp/IEF-PCM** geometries. In Table S3 are the relative Gibbs corrected energies at different levels of theory for **B3LYP-D3/def2-tzvp/IEF-PCM** geometries. In Figures S2-S11 are the IRC analyses for the found transition states.

Following a similar mechanistc pathway of our previous work[7], we provide a full description of the reaction path for the steroselective MCR (Scheme S1), starting from the hemiacetal derivative **1**. The addition of the aniline leads to the formation of the species **I-1**. Based on extensive conformational search, there is an energetic preference for the highly substituted cyclic intermediate **I-1 (A)** over the acyclic intermediate **B** (Table S5). This conformation is the key for understanding the found experimental diastereoselectivity. The isocyanide can approach via the *Re* or *Si* face of the imine, but it has a clear preference for the *Si* face via **TS-1** that leads to the formation of the favored diastereoisomer, which is lower in energy than **TS-1'.** The H-bonding activates the imine for the isocyanide attack, and the reduced energy of **TS-1** comes from a stabilizing non-covalent interaction, as revealed by NCI analysis (Figure S12). The **TS-2/TS-2'** leads to the experimental product **2/2'** while **TS-4** is the possible competitive transition state that would furnish product **11**.

After the formation of the intermediantes **I-3/I-3'** and **I-5**, we also investigated a protonated pathway by the intermediates **I-4/I-4'**and **I-5'**, respectively. In the case of the path for product **11**, the **TS-5** ($\Delta\Delta G^{\ddagger}$ = 28.9 kcal mol^{-1}) (protonated *via*) is much less energetic than the **TS-6** ($\Delta\Delta G^{\ddagger}$ = 35.3 kcal mol^{-1}) (no protonated *via*). Whereas, for product **2**, we considered 2 differents ways to obtain the final product after the formation of intermediante **I-3**: 1) the formation an acylium ion through the cleavage of ketone moiety by **TS-3** (protonated *via*) or **TS-8** (no protonated *via*), unfortunately the **TS-8** was not found; 2) the nucleophilic attack of the solvent (TFE) to the carbonyl portion by **TS-7**, wherein 2 molecules of TFE were used in the transition state, or by **TS-9** in which it was considered a protonation of the carbonyl moiety and 1 molecule of TFE, but we couldn't find it. The **TS-3** ($\Delta\Delta G^{\ddagger}$ = 11.1 kcal mol^{-1}) is extremely

favorable compared with **TS-7** ($\Delta\Delta G^{‡}$ = 44.8 kcal mol^{-1}). Despite we couldn't find the **TS-9**, a strong evidence that the mechanism pass through the **TS-3** is the high preference of an imine to be protonated compared with a carbonyl portion ($pKa_{iminium\ ion}$ ~ 10; $pKa_{carbonyl\ oxonium}$ ~ -3.1).[8,9] Therefore, this demonstrates the propensity that if there is a catalytic amont of acid, the imine will be protonated instead of the carbonyl moiety.

Ultimately, assuming that the rate determining step of the reaction consists in the **TS-1/TS-1'**, the diastereoselectivity can be determinated by the energy difference between **TS-1** and **TS-1'** ($\Delta\Delta G$ = 2.7 kcal mol^{-1}), delivering a theoretical diastereoselectivity of 98:2 after Boltzmann analysis at 70 ^{o}C, in excellent agreement with experimental results.

In order to check the reliability of these computational results, we describe in Tables S2-S3 the single point energies and Gibbs free energy corrections for the other investigated levels of theory. In Table S4 the relative reaction free energy profile and barriers for all investigated levels of theory are represented. The WB97XD and M06-2X are the functionals that are more closed in energy with B3LYP-D3, and they are very well documented in literature as appropriate to describe the thermodynamics of reaction mechanisms. The reaction energy barriers of reaction energy profile for all employed levels of theory are similar and consistent with the preference for **TS-2** (leads to the product **2**) compared with **TS-4** (leads to **11**, not experimentaly observed), and in favor of major diastereoisomer (**TS-1** lower in energy than **TS-1'**), except for the B3PW91 (without empirical dispersion), which predict the opposite diastereoselectivity.

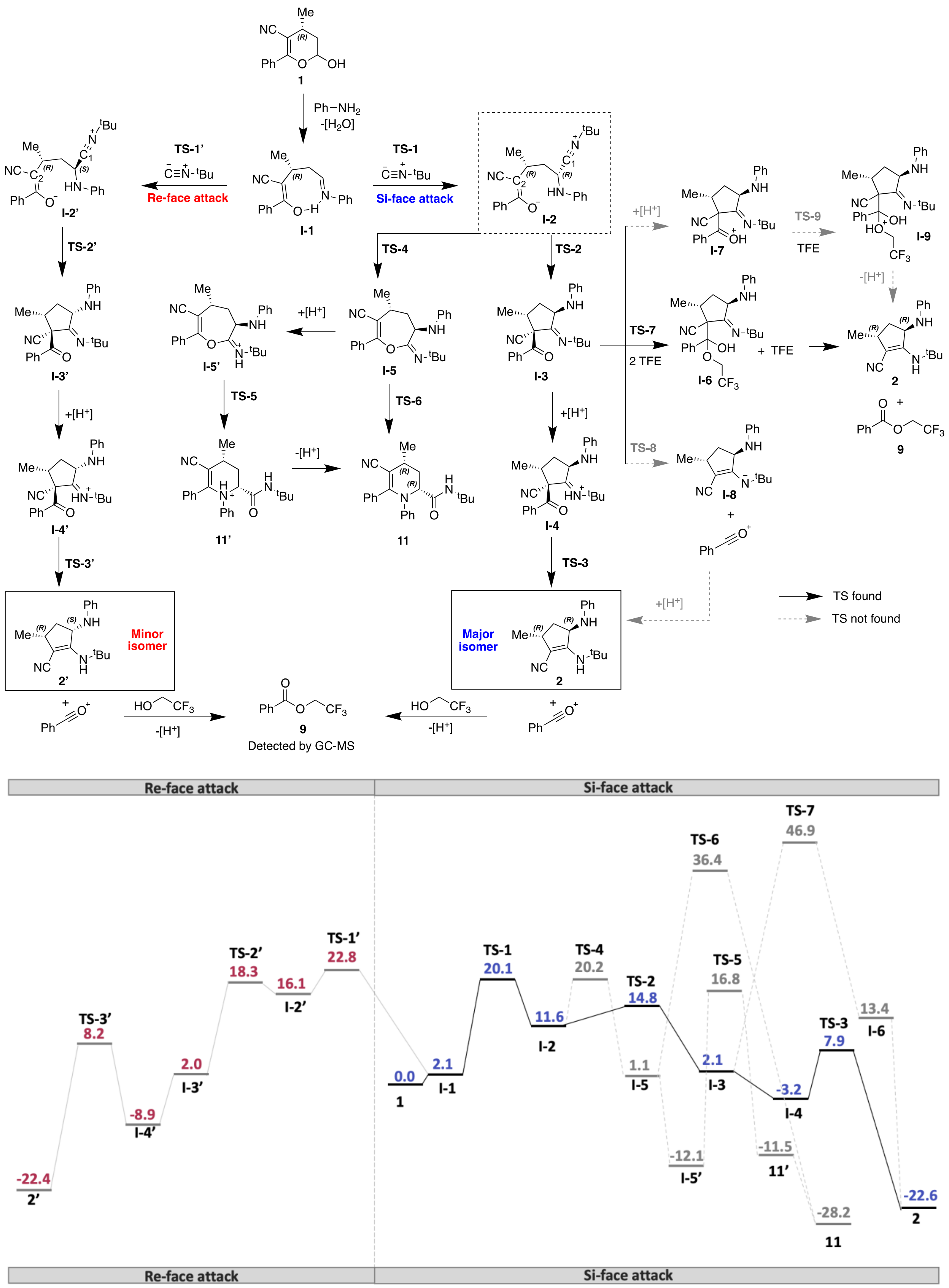

Scheme S1. Summary of all investigated intermediates and transition states.

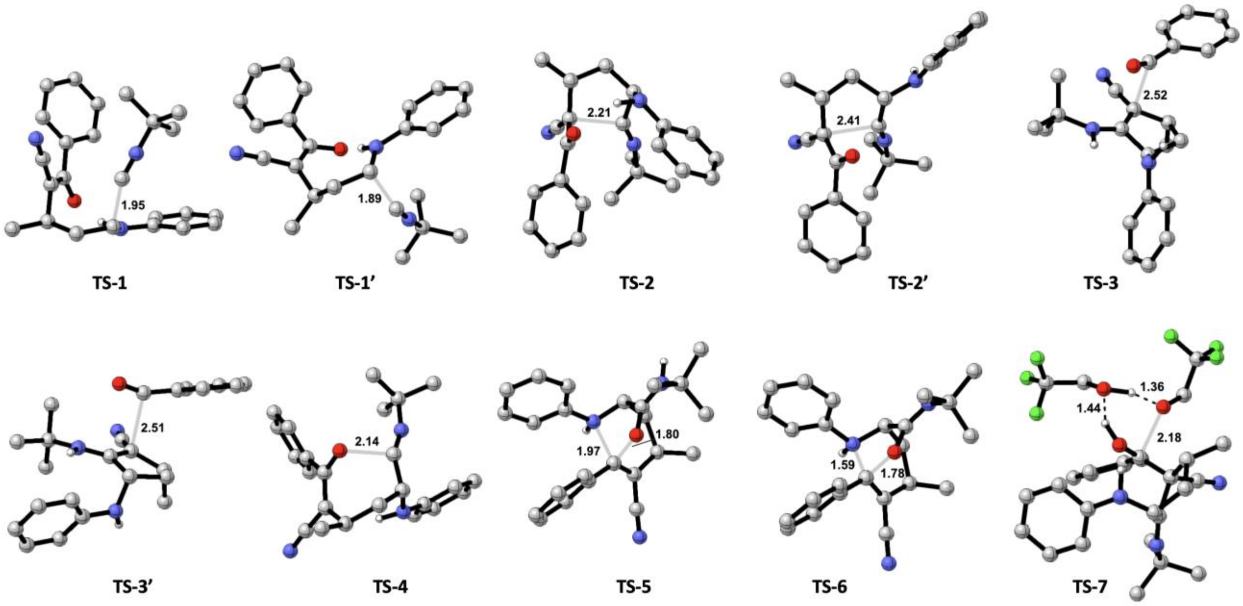

Figure S1. Calculated structures of found transition states for B3LYP-D3/def2-tzvp/IEF-PCM. Distances in angstrom.

Table S1: Energies (in Hartree) at **B3LYP-D3/def2-TZVP/IEFPCM**.

Name	ΔE_{ele}	Gibbs corretion	Imaginary Frequency (cm^{-1})	$\Delta E_{ele+ Gibbs correction}$
**water**	-76.47015956	0.003448	-	-76.46671156
**benzoyl acetonitrile**	-477.3383762	0.101227	-	-477.2371492
**pent-2-enal**	-231.3435219	0.06066	-	-231.2828619
**aniline**	-287.7320153	0.087715	-	-287.6443003
**^{t}Bu-isocyanide**	-250.7825555	0.099579	-	-250.6829765
**1**	-708.7016433	0.193162	-	-708.5084813
**I-1**	-919.9579949	0.275193	-	-919.6828019
**TS-1**	-1170.736751	0.399757	-387.33	-1170.336994
**TS-1'**	-1170.729227	0.396493	-391.54	-1170.332734
**I-2**	-1170.751617	0.401133	-	-1170.350484
**I-2'**	-1170.739708	0.396391	-	-1170.343317
**TS-2**	-1170.747183	0.401779	-157.19	-1170.345404
**TS-2'**	-1170.738601	0.398655	-115.39	-1170.339946
**I-3**	-1170.769574	0.403861	-	-1170.365713
**I-3'**	-1170.769738	0.403877	-	-1170.365861
**I-4**	-1171.2097	0.419834	-	-1170.789866
**I-4'**	-1171.216327	0.41742	-	-1170.798907
**TS-3**	-1171.186263	0.414112	-173.32	-1170.772151
**TS-3'**	-1171.184701	0.41293	-161.51	-1170.771771
**2**	-826.2714769	0.320771	-	-825.9507059
**2'**	-826.2724217	0.322	-	-825.9504217
**TS-4**	-1170.735056	0.398218	-163.04	-1170.336838
**I-5**	-1170.771653	0.404285	-	-1170.367368
**I-5'**	-1171.223958	0.419929	-	-1,170.80403
**TS-5**	-1,171	0.420358	-402.42	-1,170.75795
**TS-6**	-1170.71862	0.407649	-301.36	-1170.310971
**11'**	-1171.223442	0.420339	-	-1,170.80310
**11**	-1170.81929	0.405302	-	-1170.413988
**TS-7**	-2076.709286	0.499643	-360.24	-2,076.20964
**I-6**	-1623.761757	0.456411	-	-1018640.338
**acilium ion**	-344.9062218	0.069004	-	-344.8372178
**9**	-797.5264874	0.106446	-	-797.4200414
**TFE**	-452.9836824	0.02598	-	-452.9577024
**NH_3^+Ph/9/TFE**	-1085.703755	0.231734	-	-1085.472021
**NH_3^+Ph /TFE**	-741.1648802	0.147163	-	-741.0177172

Table S2: Single point Electronic Energies (in Hartree) at different levels of theory for **B3LYP-D3/def2-tzvp/IEF-PCM** geometries.

Name	B97D3/ def2-TZVP	B3PW91/ def2-TZVP	WB97XD/ def2-TZVP	M06-2X/ def2-TZVP
**water**	-76.43008351	-76.44058184	-76.44493442	-76.433403
**benzoyl acetonitrile**	-477.0101685	-477.126046	-477.150415	-477.12605
**pent-2-enal**	-231.1910933	-231.2422696	-231.2520372	-231.22251
**aniline**	-287.5310827	-287.6095876	-287.6179578	-287.59155
**^{t}Bu-isocyanide**	-250.6054294	-250.6673649	-250.6841015	-250.64936
**1**	-708.2184191	-708.38963	-708.432702	-708.38577
**I-1**	-919.3194672	-919.5430722	-919.5948959	-919.5305
**TS-1**	-1169.926157	-1170.189064	-1170.276617	-1170.1733
**TS-1'**	-1169.917705	-1170.19045	-1170.268005	-1170.1649
**I-2**	-1169.940215	-1170.206466	-1170.29547	-1170.1905
**I-2'**	-1169.926514	-1170.207496	-1170.28267	-1170.1783
**TS-2**	-1169.939813	-1170.204053	-1170.289678	-1170.1838
**TS-2'**	-1169.929374	-1170.201977	-1170.280915	-1170.1763
**I-3**	-1169.956075	-1170.228023	-1170.323392	-1170.2148
**I-3'**	-1169.955458	-1170.236828	-1170.323002	-1170.2153
**I-4**	-1170.402226	-1170.66762	-1170.76452	-1170.6462
**I-4'**	-1170.406007	-1170.683909	-1170.77174	-1170.6538
**TS-3**	-1170.383985	-1170.641278	-1170.728887	-1170.6104
**TS-3'**	-1170.383759	-1170.648784	-1170.730337	-1170.6125
**2**	-825.6923671	-825.9002085	-825.9560511	-825.86178
**2'**	-825.6927729	-825.9032269	-825.9570873	-825.86319
**TS-4**	-1169.924665	-1170.193349	-1170.277055	-1170.1718
**I-5**	-1169.95606	-1170.240107	-1170.320028	-1170.2174
**I-5’**	-1170.411575	-1170.692957	-1170.775104	-1170.662624
**TS-5**	-1170.376645	-1170.639424	-1170.725553	-1170.610812
**TS-6**	-1169.909791	-1170.183035	-1170.269744	-1170.1646
**11’**	-1170.414962	-1170.685227	-1170.776311	-1170.661665
**11**	-1170.005215	-1170.284936	-1170.371758	-1170.2642
**TS-7**	-2075.410907	-2075.78309	-2075.984375	-2075.813748
**I-6**	-1622.696721	-1623.035306	-1623.185896	-1623.04929
**acilium ion**	-344.6777256	-344.7558866	-344.7697119	-344.74676
**9**	-797.0448302	-797.1968321	-797.2574337	-797.21193
**TFE**	-452.7400114	-452.8068071	-452.8489512	-452.81762
**NH_3^+Ph/9/TFE**	-1085.025608	-1085.232372	-1085.322559	-1085.2404
**NH_3^+Ph /TFE**	-740.7233966	-740.8591727	-740.9180153	-740.85089

Table S3: Gibbs corrected Energies (in Hartree) at different levels of theory for **B3LYP-D3/def2-tzvp/IEF-PCM** geometries.

Name	B97D3/ def2-TZVP	B3PW91/ def2-TZVP	WB97XD/ def2-TZVP	M06-2X/ def2-TZVP
**water**	-76.42663551	-76.43713384	-76.44148642	-76.42995487
**benzoyl acetonitrile**	-476.9089415	-477.024819	-477.049188	-477.0248267
**pent-2-enal**	-231.1304333	-231.1816096	-231.1913772	-231.1618479
**aniline**	-287.4433677	-287.5218726	-287.5302428	-287.5038303
**^{t}Bu-isocyanide**	-250.5059044	-250.5678399	-250.5845765	-250.5498374
**1**	-708.0252571	-708.196468	-708.23954	-708.192604
**I-1**	-919.0442742	-919.2678792	-919.3197029	-919.2553029
**TS-1**	-1169.526407	-1169.789314	-1169.876867	-1169.773532
**TS-1'**	-1169.521212	-1169.793957	-1169.871512	-1169.768372
**I-2**	-1169.539082	-1169.805333	-1169.894337	-1169.789352
**I-2'**	-1169.530118	-1169.8111	-1169.886274	-1169.781863
**TS-2**	-1169.538034	-1169.802274	-1169.887899	-1169.782063
**TS-2'**	-1169.530719	-1169.803322	-1169.88226	-1169.777676
**I-3**	-1169.552214	-1169.824162	-1169.919531	-1169.810936
**I-3'**	-1169.551581	-1169.832951	-1169.919125	-1169.811428
**I-4**	-1169.982364	-1170.247758	-1170.344658	-1170.2263
**I-4'**	-1169.988584	-1170.266486	-1170.354317	-1170.236346
**TS-3**	-1169.969843	-1170.227136	-1170.314745	-1170.196257
**TS-3'**	-1169.970735	-1170.23576	-1170.317313	-1170.199521
**2**	-825.3715961	-825.5794375	-825.6352801	-825.5410117
**2'**	-825.3707729	-825.5812269	-825.6350873	-825.541188
**TS-4**	-1169.526447	-1169.795131	-1169.878837	-1169.773554
**I-5**	-1169.551775	-1169.835822	-1169.915743	-1169.813151
**I-5’**	-1169.991646	-1170.273028	-1170.355175	-1170.242695
**TS-5**	-1169.956287	-1170.219066	-1170.305195	-1170.190454
**TS-6**	-1169.502146	-1169.77539	-1169.862099	-1169.756998
**11’**	-1169.994623	-1170.264888	-1170.355972	-1170.241326
**11**	-1169.599915	-1169.879636	-1169.966458	-1169.858854
**TS-7**	-2074.911264	-2075.283447	-2075.484732	-2075.314105
**I-6**	-1622.24031	-1622.578895	-1622.729485	-1622.592879
**acilium ion**	-344.6087166	-344.6868776	-344.7007029	-344.6777515
**9**	-796.9383802	-797.0903821	-797.1509837	-797.1054761
**TFE**	-452.7140314	-452.7808271	-452.8229712	-452.7916383
**NH_3^+Ph/9/TFE**	-1084.793879	-1085.000643	-1085.09083	-1085.008626
**NH_3^+Ph /TFE**	-740.5762586	-740.7120347	-740.7708773	-740.7037522

Table S4. Gibbs corrected energy profiles for all investigated levels of theory**.** Energies (in kcal mol^{-1}) for studied compounds at different levels of theory for **B3LYP-D3/def2-tzvp/IEF-PCM** geometries. Reaction barrier in bracket.

	**B3LYP-D3/ def2-TZVP**	**B97D3/ def2-TZVP**	**B3PW91/ def2-TZVP**	**WB97XD/ def2-TZVP**	**M062X/ def2-TZVP**
**1**	0.0	0.0	0.0	0.0	0.0
**I-1**	2.1	-1.4	8.4	5.4	7.0
**TS-1**	20.1 [18.0]	13.5 [14.9]	37.5 [29.1]	22.6 [17.2]	26.8 [19.8]
**TS-1'**	22.8 [20.7]	16.7 [18.1]	34.6 [26.2]	26.0 [20.6]	30.1 [23.1]
**I-2**	11.7	5.5	27.4	11.6	16.9
**I-2'**	16.2	11.2	23.8	16.7	21.6
**TS-2**	14.9 [3.2]	6.2 [0.7]	29.4 [2.0]	15.7 [4.1]	21.5 [4.6]
**TS-2'**	18.3 [2.1]	10.8 [-0.4]	28.7 [4.9]	19.2 [2.5]	24.2 [2.6]
**I-3**	2.1	-2.7	15.6	-4.2	3.4
**I-3'**	2.0	-2.3	10.1	-3.9	3.1
**I-4**	-3.1	-9.8	6.7	-8.9	-1.1
**I-4'**	-8.8	-13.7	-5.1	-14.9	-7.4
**TS-3**	8.0 [11.1]	-1.9 [7.9]	19.6 [12.9]	9.9 [18.8]	17.8 [18.9]
**TS-3'**	8.3 [17.1]	-2.5 [11.2]	14.2 [19.3]	8.3 [23.2]	15.7 [23.1]
**2**	-22.5	-25.9	-11.9	-26.6	-18.6
**2'**	-22.3	-25.4	-13.0	-26.5	-18.7
**TS-4**	20.2 [8.5]	13.5 [8.0]	33.8 [6.4]	21.4 [9.8]	26.8 [9.9]
**I-5**	1.1	-2.4	8.3	-1.8	2.0
**I-5'**	-12.1	-15.6	-9.2	-15.5	-11.4
**TS-5**	16.8 [28.9]	6.6 [22.2]	24.7 [33.9]	15.9 [31.4]	21.4 [32.8]
**TS-6**	36.5 [35.4]	28.7 [31.1]	46.2 [37.9]	31.9 [33.7]	37.2 [35.2]
**11'**	-11.5	-17.5	-4.1	-16	-10.5
**11**	-28.2	-32.6	-19.2	-33.6	-26.7
**TS-7**	46.9 [44.8]	40.6 [43.3]	76.9 [61.3.0]	46.5 [50.7]	53.6 [50.2]
**I-6**	13.4	13.6	32.0	4.0	9.5

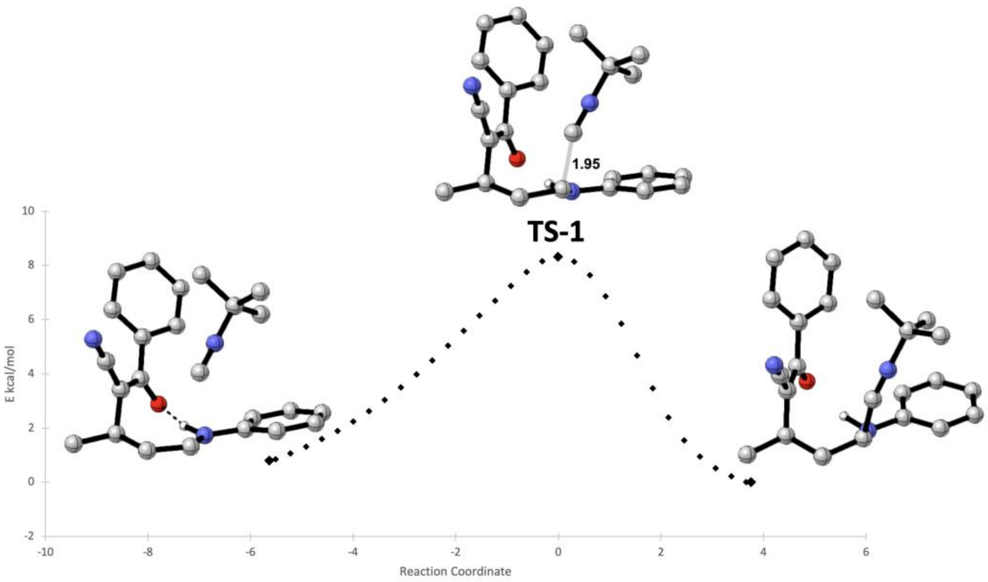

Figure S2. IRC for **TS-1** at **B3LYP-D3/def2-tzvp/IEF-PCM**.

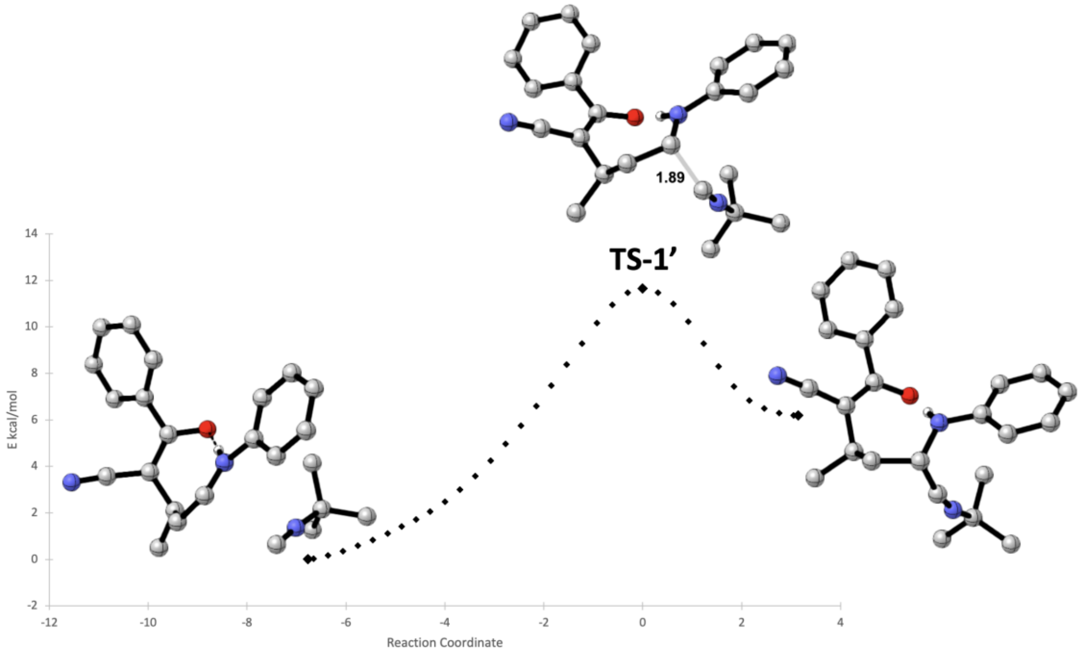

Figure S3. IRC for **TS-1’** at **B3LYP-D3/def2-tzvp/IEF-PCM**.

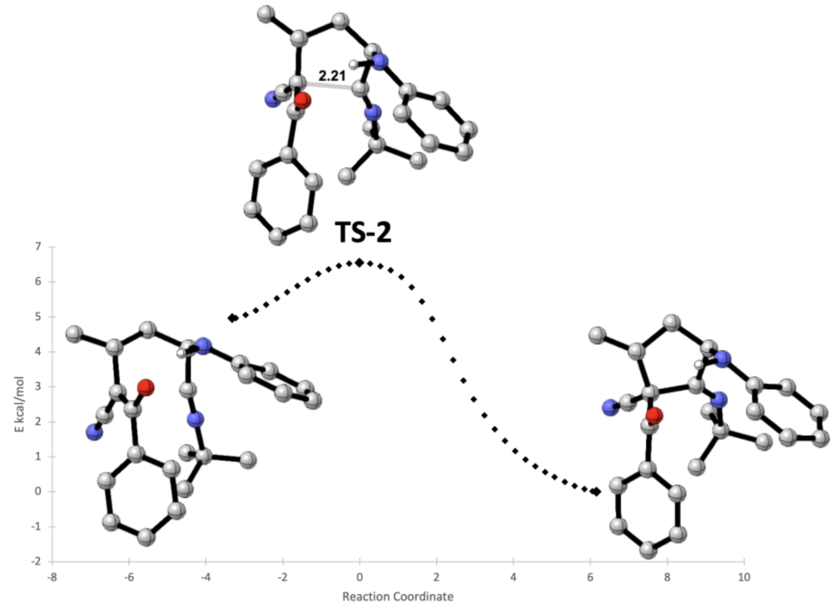

Figure S4. IRC for **TS-2** at **B3LYP-D3def2-tzvp/IEF-PCM**.

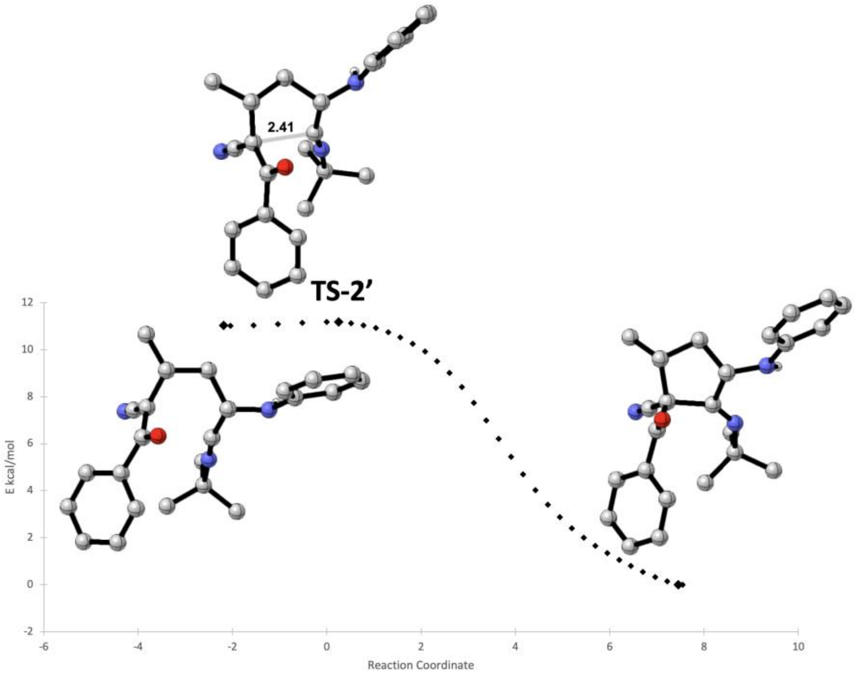

Figure S5. IRC for **TS-2'** at **B3LYP-D3/def2-tzvp/IEF-PCM**.

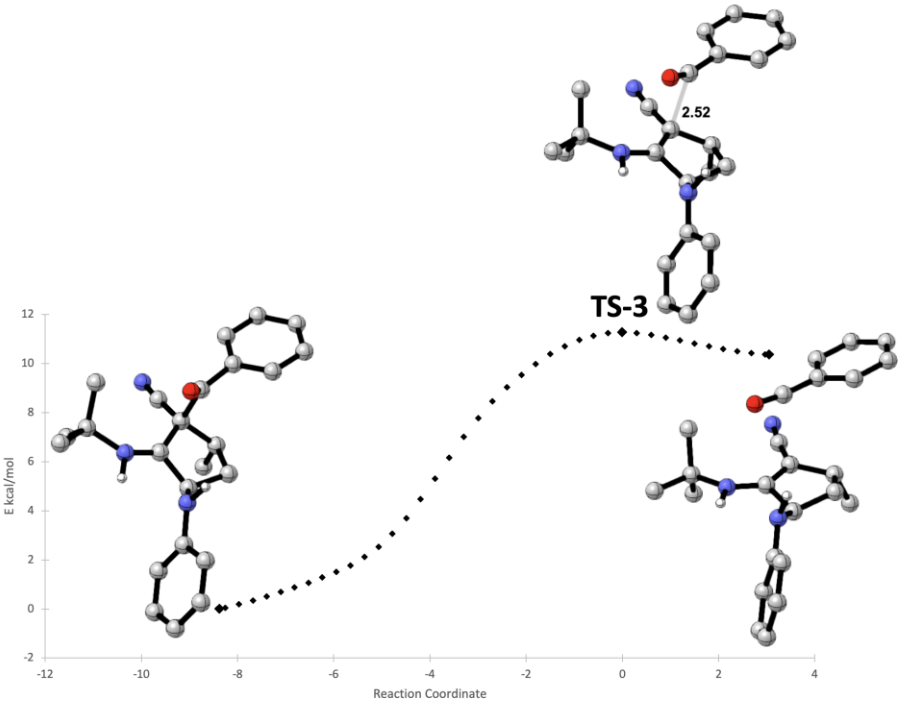

Figure S6. IRC for **TS-3** at **B3LYP-D3/def2-tzvp/IEF-PCM**.

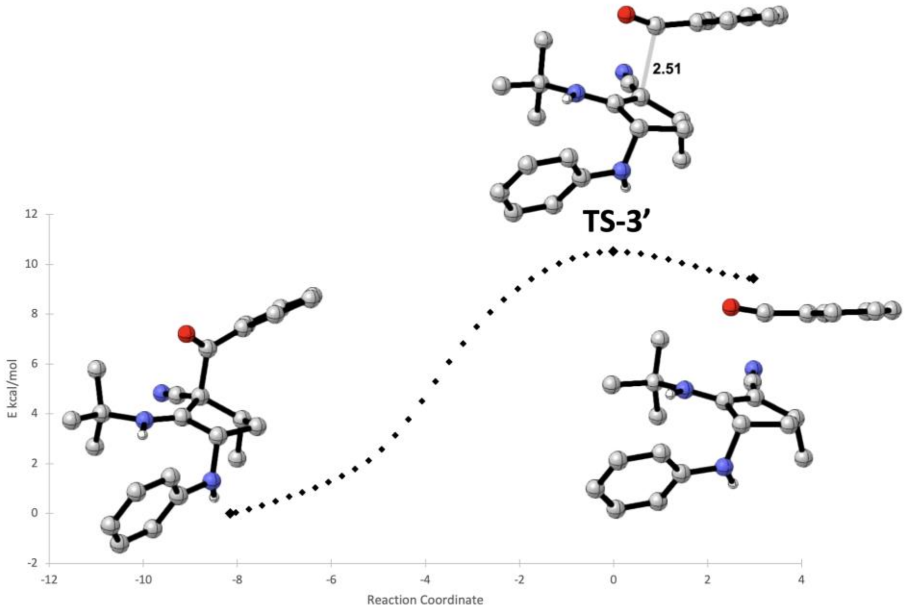

Figure S7. IRC for **TS-3’** at **B3LYP-D3/def2-tzvp/IEF-PCM**.

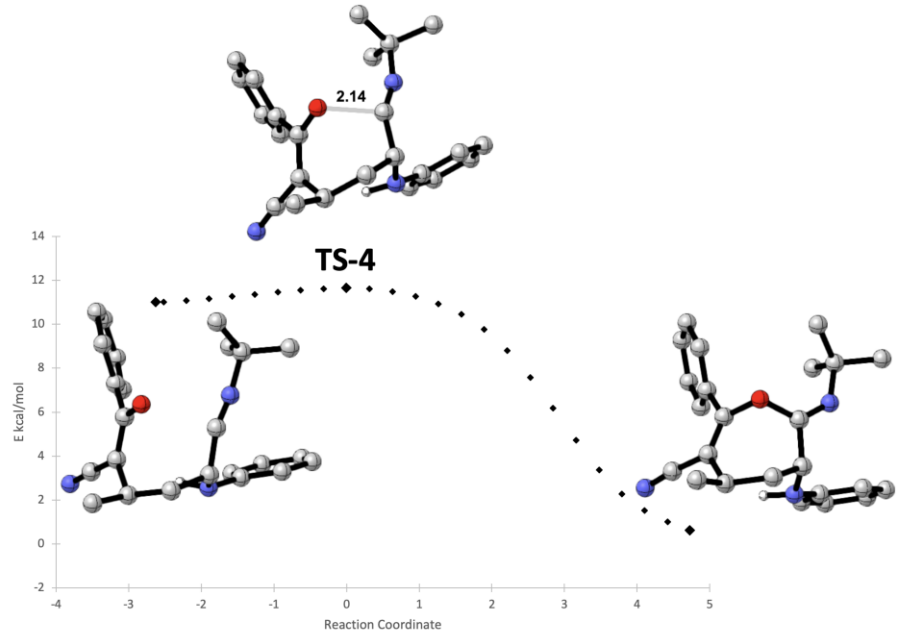

Figure S8. IRC for **TS-4** at **B3LYP-D3/def2-tzvp/IEF-PCM**.

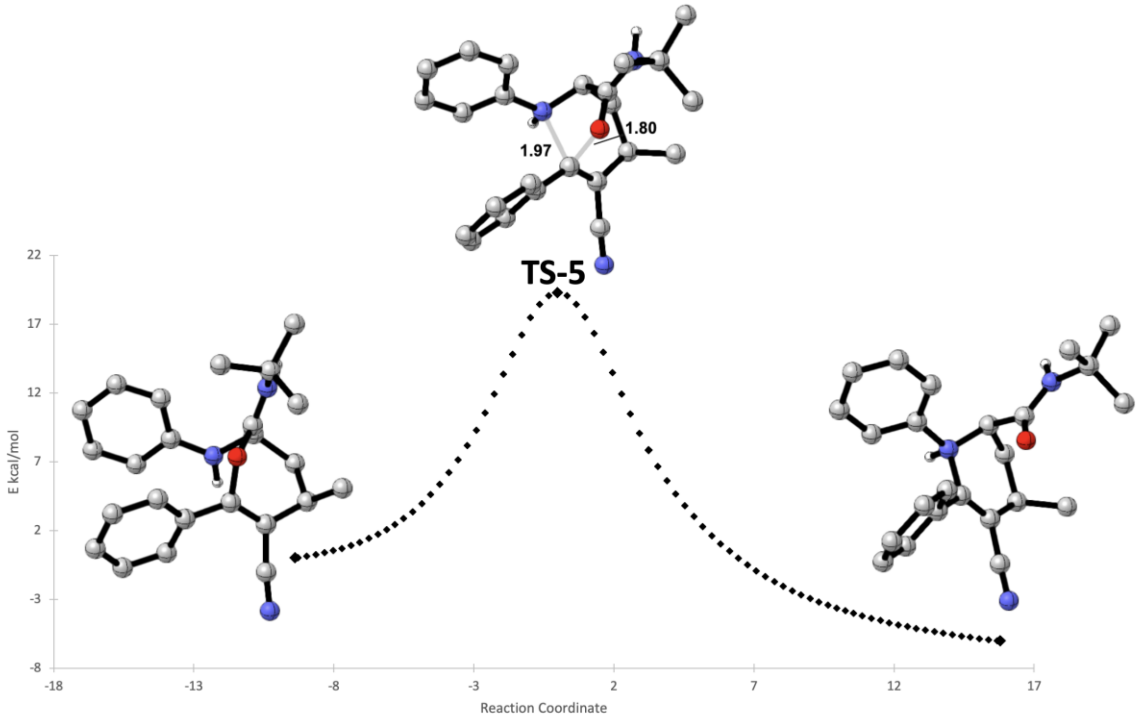

Figure S9. IRC for **TS-5** at **B3LYP-D3/def2-tzvp/IEF-PCM**.

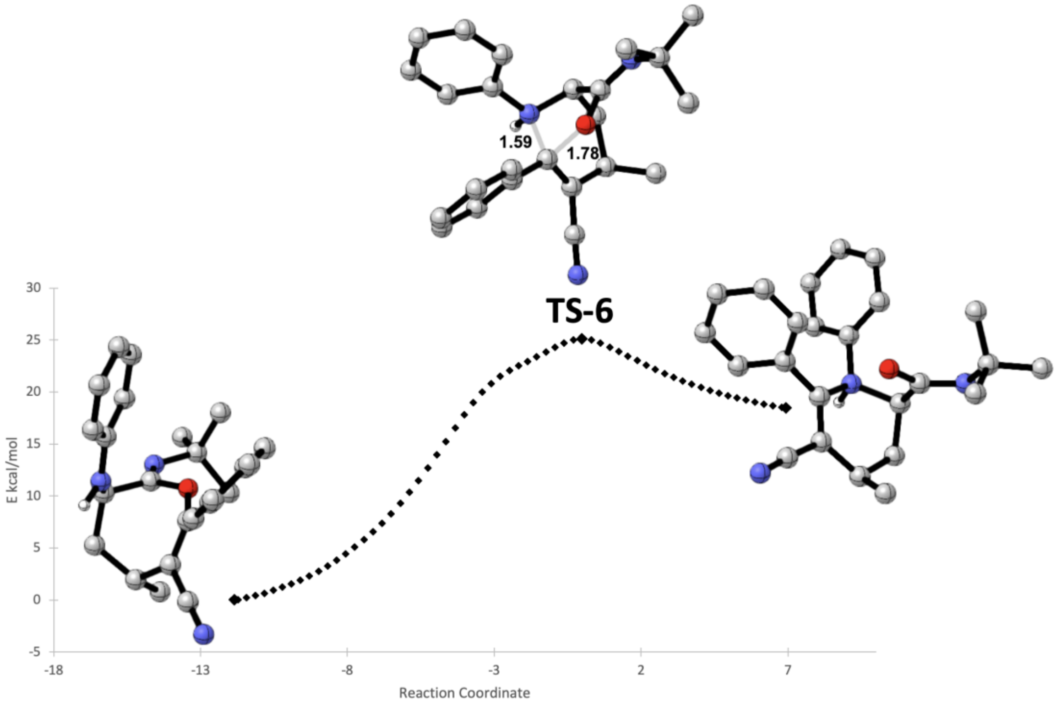

Figure S10. IRC for **TS-6** at **B3LYP-D3/def2-tzvp/IEF-PCM**.

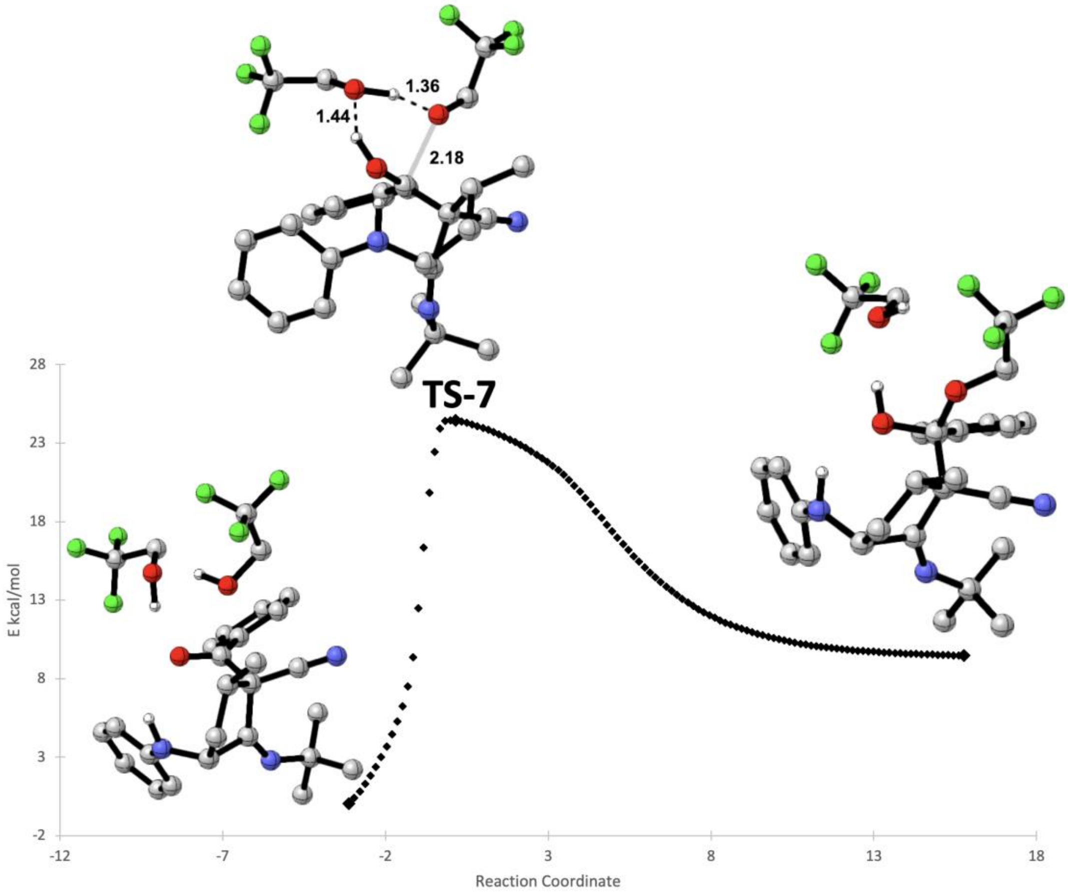

Figure S11. IRC for **TS-7** at **B3LYP-D3/def2-tzvp/IEF-PCM**.

Table S5: Energies for lowest conformers of structures **I-1** at **B3LYP-D3/def2-tzvp/IEFPCM.**

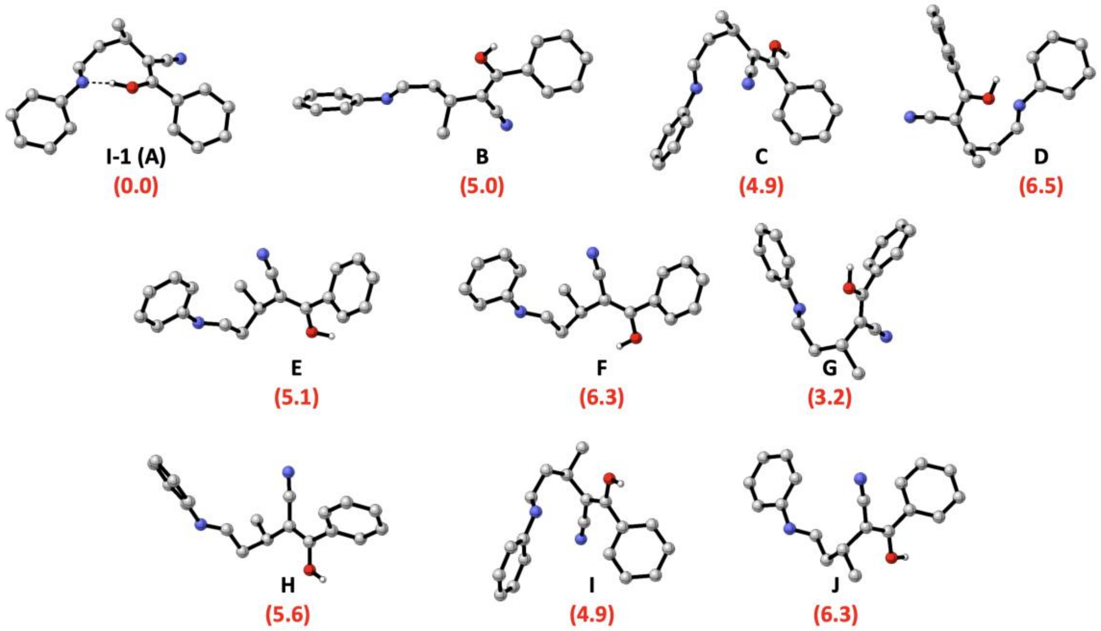

**Conformer**	**ΔE_{ele}** (hartree)	**ZPE correction** (hartree)	**$\Delta E_{ele+ZPE}$** (hartree)	**$\Delta\Delta E_{ele+ZPE}$** (kcal mol-1)
I-1 (A)	**-919.9579949**	**0.324564**	**-919.63343**	**0.0**
B	-919.9495355	0.324093	-919.62544	5.0
C	-919.9501639	0.324475	-919.62569	4.9
D	-919.9479514	0.324808	-919.62314	6.5
E	-919.9499496	0.324646	-919.6253	5.1
F	-919.9479626	0.32465	-919.62331	6.3
G	-919.9528311	0.324429	-919.6284	3.2
H	-919.9488455	0.324407	-919.62444	5.6
I	-919.9502012	0.32455	-919.62565	4.9
J	-919.9482357	0.324767	-919.62347	6.3

Table S6: Energies for lowest conformers of structures **I-2/I-2'** at **B3LYP-D3/def2-tzvp/IEFPCM.**

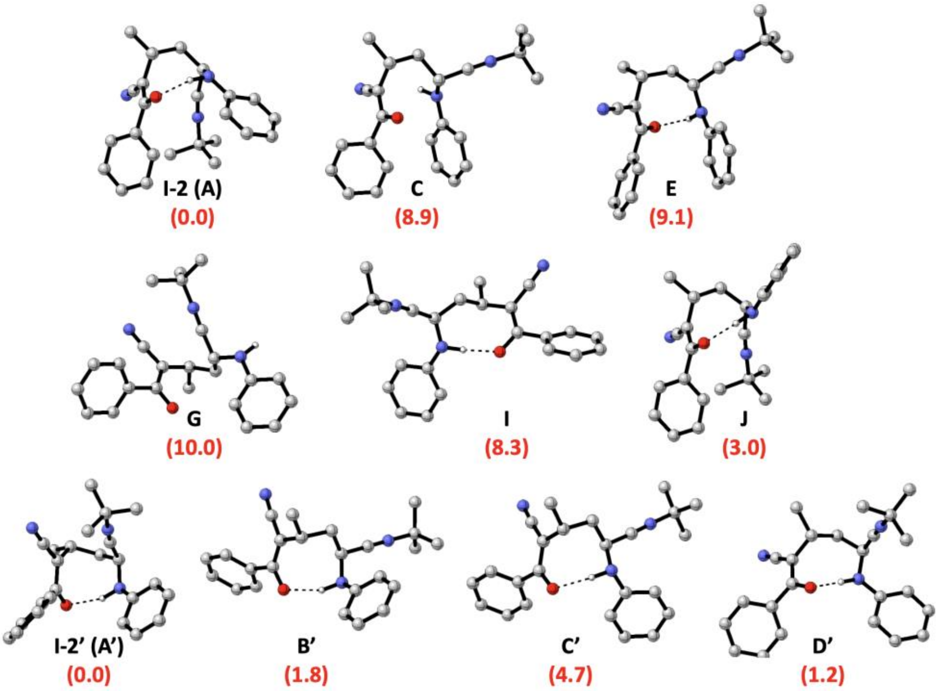

**Conformer**	**ΔE_{ele}** (hartree)	**ZPE correction** (hartree)	**$\Delta E_{ele+ZPE}$** (hartree)	**$\Delta\Delta E_{ele+ZPE}$** (kcal mol-1)
I-2 (A)	**-1170.7516**	**0.459186**	**-1170.2924**	**0.0**
B	-1170.7296	0.458414	-1170.2712	13.3
C	-1170.7362	0.457944	-1170.2783	8.9
D	-1170.735	0.458595	-1170.2764	10.1
E	-1170.7363	0.458289	-1170.278	9.1
F	-1170.7267	0.458622	-1170.2681	15.3
G	-1170.7347	0.458206	-1170.2765	10.0
H	-1170.7328	0.45855	-1170.2742	11.4
I	-1170.7374	0.458197	-1170.2792	8.3
J	-1170.7463	0.458647	-1170.2876	3.0
I-2' (A')	**-1170.7423**	**0.458905**	**-1170.2834**	**0.0**
B'	-1170.7391	0.458531	-1170.2806	1.8
C'	-1170.7347	0.458776	-1170.2759	4.7
D'	-1170.7397	0.458301	-1170.2814	1.2

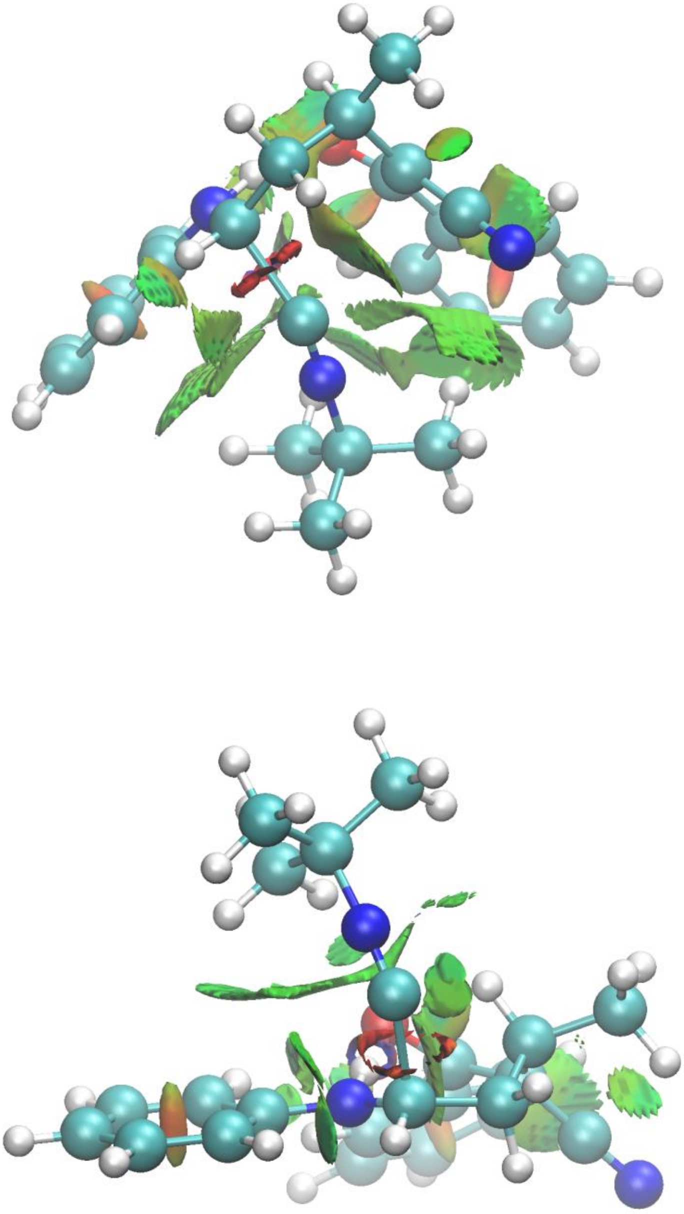

Figure S12. NCI analysis for **TS-1** (top) and **TS-1' (**bottom) at **B3LYP-D3/def2-TZVP**.

C. Cartesian Coordinates

B3LYP-D3/def2-tzvp/IEFPCM

1 Energy: -444716.9918446

C	0.08997	-0.28734	-0.00936
C	0.94471	0.76645	0.05051
O	0.51172	-1.56699	-0.10827
O	2.24161	-1.94311	1.39912
H	1.78810	-2.70417	1.78304
C	2.76444	-0.77888	-0.59445
H	2.54941	-0.76143	-1.66524
H	3.81669	-1.03166	-0.46601
C	2.45763	0.58537	0.02294
H	2.81450	0.58384	1.05783
C	1.92173	-1.85769	0.04144
H	2.02930	-2.82101	-0.45864
C	0.44322	2.08891	0.10722
N	0.10395	3.19375	0.14986
C	-1.38644	-0.20938	-0.02401
C	-2.08618	0.59492	0.87839
C	-2.10124	-0.98255	-0.94575
C	-3.47477	0.64059	0.84587
H	-1.54783	1.17141	1.61754
C	-3.48762	-0.92725	-0.98134
H	-1.56454	-1.61816	-1.63701
C	-4.17845	-0.11449	-0.08637
H	-4.00614	1.26192	1.55514
H	-4.02987	-1.51965	-1.70708
H	-5.25993	-0.07567	-0.11171
C	3.18291	1.69942	-0.73311
H	3.01817	2.67228	-0.26960
H	2.84178	1.75310	-1.76941
H	4.25652	1.50514	-0.73648

I-1 Energy: -577282.3528858

C	1.34194	1.52121	-0.03161
C	1.33483	0.22838	0.41267
C	2.35601	1.94150	-0.92730
C	0.34508	2.62737	0.33396
C	-0.83017	2.78222	-0.65127
C	-1.90788	1.75199	-0.68224
N	-1.88979	0.66284	-0.02838
N	3.12848	2.35829	-1.68263
H	0.91570	3.55006	0.20191
H	-0.44612	2.87015	-1.67475
H	-1.32603	3.74186	-0.46146
C	4.51119	-2.58511	-0.11206
C	4.80544	-1.27688	0.25964
C	3.78247	-0.35224	0.42811
C	2.45097	-0.72591	0.22689
C	2.16232	-2.04942	-0.12420
C	3.18634	-2.96931	-0.30260
H	5.30942	-3.30424	-0.24488
H	5.83207	-0.97733	0.42686
H	4.01594	0.65795	0.73537
H	1.13195	-2.34661	-0.26427
H	2.95177	-3.98691	-0.58784
C	-5.04075	-2.11955	-0.14691
C	-3.71360	-2.54023	-0.17203
C	-2.68325	-1.61044	-0.12770
C	-2.97479	-0.24536	-0.08241
C	-4.30587	0.17685	-0.03987
C	-5.33144	-0.76069	-0.07401
H	-5.84231	-2.84634	-0.17066
H	-3.47933	-3.59599	-0.21988
H	-1.64949	-1.93033	-0.13784
H	-4.53519	1.23107	0.04657
H	-6.36052	-0.42699	-0.03362
O	0.29599	-0.30167	1.05741
H	-0.57121	0.13447	0.74360
H	-2.75299	1.98798	-1.33534
C	-0.09406	2.63128	1.80552
H	-0.74763	1.80372	2.06656
H	0.77891	2.59126	2.45784
H	-0.63244	3.55763	2.01412

I-1’ Energy: -577277.0433518

C	-1.54123	0.47162	0.22975
C	-2.43473	-0.52906	0.42637
C	-1.94948	1.66535	-0.41664
C	-0.06827	0.34825	0.58683
C	0.76097	0.01143	-0.67778
C	2.17907	-0.31843	-0.34721
N	3.16194	0.34497	-0.79872
N	-2.20495	2.65896	-0.95008
H	0.01530	-0.49341	1.27548
H	0.73161	0.84497	-1.38213
H	0.30827	-0.86028	-1.16092
C	-6.62533	-0.39245	-0.37653
C	-6.02304	0.64191	0.33354
C	-4.65965	0.60903	0.59409
C	-3.88316	-0.46275	0.14487
C	-4.49723	-1.50599	-0.55884
C	-5.86003	-1.46606	-0.82173
H	-7.68813	-0.36372	-0.57928
H	-6.61691	1.47259	0.69199
H	-4.19714	1.40580	1.16025
H	-3.90371	-2.33594	-0.92166
H	-6.32282	-2.27058	-1.37825
C	7.11915	-0.55270	0.31680
C	6.12901	-0.48966	1.29217
C	4.81313	-0.20756	0.94227
C	4.47375	0.02000	-0.39645

C	5.47782	-0.01581	-1.36911
C	6.78669	-0.31484	-1.01517
H	8.14269	-0.77165	0.59245
H	6.38070	-0.65540	2.33235
H	4.04962	-0.13862	1.70667
H	5.21503	0.18353	-2.40029
H	7.55213	-0.35376	-1.78011
O	-1.96178	-1.71096	0.88852
H	-2.69340	-2.27965	1.16336
H	2.32758	-1.17708	0.32072
C	0.45588	1.60333	1.29245
H	1.49013	1.46132	1.60791
H	-0.14209	1.82747	2.17681
H	0.42652	2.47091	0.63017

I-2 Energy: -734657.7254957

C	1.70339	-1.61233	0.31133
C	1.37744	-0.69654	1.33347
C	2.49944	-1.28140	-0.79307
C	1.14693	-3.02568	0.36329
C	-0.00663	-3.24230	-0.63762
N	-1.60988	-1.79971	0.62064
N	3.12664	-1.07320	-1.75081
H	0.74307	-3.14541	1.36911
H	-0.53383	-4.16924	-0.40384
H	0.39436	-3.34785	-1.64934
C	2.74916	3.37933	1.28414
C	3.64772	2.35757	0.99392
C	3.22748	1.03141	1.00120
C	1.90418	0.70902	1.30710
C	1.01637	1.74155	1.62694
C	1.42999	3.06674	1.60298
H	3.07537	4.41155	1.26732
H	4.67881	2.59230	0.76115
H	3.93484	0.24570	0.77524
H	-0.00439	1.48844	1.88182
H	0.72457	3.85614	1.83068
C	-4.60147	1.13403	1.06887
C	-4.55415	0.36756	-0.08958
C	-3.57426	-0.60445	-0.26251
C	-2.61547	-0.82390	0.73311
C	-2.66380	-0.04633	1.89867
C	-3.64915	0.91584	2.06223
H	-5.36443	1.89068	1.19573
H	-5.28432	0.52380	-0.87383
H	-3.56841	-1.18477	-1.17534
H	-1.90791	-0.19529	2.65933
H	-3.66483	1.50851	2.96852
O	0.57718	-0.98444	2.27152
H	-0.87902	-1.67151	1.32799
C	-1.06016	-2.13030	-0.67335
H	-1.85033	-2.45200	-1.35724
C	-0.54465	-0.87530	-1.29540
N	-0.40298	0.16648	-1.74639
C	-0.27592	1.51118	-2.29915
C	-0.73010	1.43355	-3.76058
H	-0.09231	0.75745	-4.32929
H	-1.76381	1.09611	-3.83067
H	-0.65733	2.42982	-4.19559
C	1.19029	1.93620	-2.18272
H	1.28985	2.92835	-2.62218
H	1.50269	1.98140	-1.14170
H	1.83985	1.24219	-2.71309
C	-1.19775	2.40794	-1.46287
H	-2.23036	2.06405	-1.51425
H	-0.87914	2.42321	-0.42188
H	-1.14081	3.42064	-1.86083
C	2.21130	-4.10787	0.15447
H	1.77048	-5.10476	0.22668
H	2.67785	-4.01742	-0.82925
H	2.99444	-4.02467	0.90918

I-2’ Energy: -734650.2477164

C	-1.84817	-1.46890	0.50662
C	-2.02047	-0.42928	-0.41171
C	-2.86642	-1.85929	1.38751
C	-0.50348	-2.16604	0.66603
C	0.32095	-1.64802	1.87511
N	0.67388	0.67360	1.00919
N	-3.65648	-2.21981	2.16260
H	0.05040	-1.95015	-0.25147
H	0.92243	-2.45691	2.29596
H	-0.34415	-1.30258	2.66755
C	-5.84159	1.41172	-1.13552
C	-4.69861	2.18596	-0.94835
C	-3.47389	1.57358	-0.71533
C	-3.37329	0.18121	-0.64016
C	-4.52378	-0.58588	-0.83378
C	-5.74871	0.02447	-1.08459
H	-6.79589	1.88682	-1.32567
H	-4.76298	3.26638	-0.98838
H	-2.57963	2.16917	-0.58578
H	-4.45791	-1.66527	-0.80056
H	-6.63001	-0.58449	-1.24385
C	2.63404	4.35996	0.47889
C	3.15128	3.43072	1.37228
C	2.53478	2.19639	1.55948
C	1.37220	1.87405	0.84813
C	0.85846	2.81310	-0.06144
C	1.48148	4.03771	-0.23731
H	3.11981	5.31640	0.33766
H	4.04838	3.65951	1.93419
H	2.96960	1.50094	2.26436
H	-0.02954	2.55921	-0.62701

H	1.06668	4.74584	-0.94407
O	-1.05103	0.07557	-1.06727
H	0.05000	0.45487	0.21357
C	1.29044	-0.48650	1.58634
H	1.79981	-0.23938	2.51908
C	2.34881	-0.95070	0.64481
N	3.07469	-1.20267	-0.19990
C	3.96587	-1.48129	-1.32251
C	3.41175	-2.72110	-2.03192
H	3.40014	-3.58008	-1.36194
H	2.40267	-2.54036	-2.40060
H	4.05882	-2.94674	-2.87872
C	3.92930	-0.24195	-2.22467
H	4.28588	0.63845	-1.69124
H	4.58082	-0.42284	-3.07898
H	2.91920	-0.05491	-2.58738
C	5.36250	-1.72892	-0.74547
H	5.72123	-0.85450	-0.20351
H	5.36050	-2.58940	-0.07728
H	6.04253	-1.93067	-1.57226
C	-0.65557	-3.69021	0.76170
H	-1.17007	-3.97888	1.68112
H	-1.23444	-4.07071	-0.08098
H	0.32203	-4.17692	0.75619

I-3 Energy: -734668.9934260

C	-1.61378	0.82190	-1.96217
N	1.25205	-0.01249	-1.74584
C	-1.93223	1.16636	0.50388
C	-1.83863	0.07157	-0.58734
C	-0.69030	-1.01980	-0.57762
C	0.09825	-0.87879	-1.89897
C	-0.91170	-0.22505	-2.83270
O	-3.02907	1.48309	0.91575
N	-0.42855	-1.95958	0.20611
C	-0.97308	-2.37776	1.50134
C	0.25396	-2.79556	2.33067
C	-1.77511	-1.34096	2.29803
C	-1.84597	-3.61108	1.21460
C	-2.85757	1.44342	-2.57645
C	-3.10102	-0.66125	-0.64034
N	-4.07344	-1.26971	-0.72248
C	-0.71791	1.93383	0.90078
C	-0.89352	3.29909	1.16402
C	0.18407	4.08674	1.53695
C	1.44866	3.51749	1.67379
C	1.62791	2.16019	1.43372
C	0.55339	1.37219	1.04072
C	2.46149	-0.35295	-1.17726
C	2.68049	-1.58147	-0.53517
C	3.91730	-1.85927	0.03834
C	4.95767	-0.93839	-0.00548
C	4.74193	0.28444	-0.64104
C	3.51691	0.57747	-1.21625
H	-0.90481	1.62215	-1.74108
H	1.23220	0.85535	-2.25403
H	0.40579	-1.87238	-2.23083
H	-1.63301	-0.97114	-3.17297
H	-0.44849	0.21790	-3.71474
H	-0.06198	-3.20630	3.29092
H	0.83510	-3.54889	1.79867
H	0.90045	-1.93657	2.51989
H	-2.02468	-1.76363	3.27236
H	-2.71247	-1.07238	1.81761
H	-1.18954	-0.43936	2.47772
H	-2.71704	-3.33830	0.61855
H	-1.27491	-4.36709	0.67466
H	-2.19112	-4.04211	2.15571
H	-2.57824	2.00398	-3.46954
H	-3.58226	0.68178	-2.86726
H	-3.34301	2.13225	-1.88356
H	-1.87878	3.73172	1.05706
H	0.04052	5.14352	1.72098
H	2.29007	4.13154	1.96847
H	2.60478	1.70857	1.54163
H	0.71679	0.32187	0.87248
H	1.87828	-2.29899	-0.45480
H	4.05996	-2.81281	0.53279
H	5.91449	-1.16288	0.44723
H	5.53568	1.02030	-0.68593
H	3.36118	1.53545	-1.69890

I-3’ Energy: -734669.0968647

C	-0.23680	-2.01297	0.47659
N	2.81001	0.14927	0.45884
C	-1.82096	-0.49950	-0.82200
C	-0.86648	-0.55650	0.39998
C	0.39366	0.36616	0.14991
C	1.61542	-0.55171	0.06410
C	1.20844	-1.76116	0.90177
O	-1.28548	-0.41944	-1.90869
N	0.54766	1.60457	0.10254
C	-0.35277	2.75790	0.20725
C	-0.23624	3.26787	1.65342
C	0.22605	3.80881	-0.75447
C	-1.82327	2.52014	-0.15721
C	-0.97655	-3.03077	1.32961
C	-1.41945	-0.18118	1.69183
N	-1.79235	0.11165	2.74056
C	-3.29905	-0.57832	-0.69993
C	-4.05310	-0.08549	-1.77427
C	-5.43630	-0.14079	-1.74613
C	-6.08821	-0.70944	-0.65328
C	-5.34943	-1.21487	0.41029

C	-3.96227	-1.14151	0.39429
C	4.06607	-0.19425	-0.01645
C	5.11676	0.73492	0.09337
C	6.39404	0.41786	-0.33904
C	6.66989	-0.83122	-0.89574
C	5.63684	-1.75392	-1.00919
C	4.34947	-1.44899	-0.57714
H	-0.22348	-2.36542	-0.55665
H	2.63778	1.14551	0.52579
H	1.67880	-0.87174	-0.98788
H	1.26695	-1.51161	1.96486
H	1.82848	-2.63876	0.72608
H	-0.79204	4.20099	1.75814
H	-0.64266	2.54178	2.35671
H	0.80730	3.45571	1.90897
H	-0.34612	4.73583	-0.69133
H	0.18460	3.44834	-1.78409
H	1.26632	4.02055	-0.50565
H	-1.91798	2.13646	-1.17221
H	-2.32580	1.84520	0.53211
H	-2.35681	3.47061	-0.11188
H	-0.42097	-3.96964	1.32033
H	-1.06190	-2.70326	2.36669
H	-1.97541	-3.23788	0.94579
H	-3.53800	0.34817	-2.62008
H	-6.00897	0.25673	-2.57379
H	-7.16939	-0.75700	-0.63227
H	-5.85117	-1.66488	1.25666
H	-3.41396	-1.54054	1.23226
H	4.91605	1.70957	0.52337
H	7.18234	1.15488	-0.24410
H	7.66864	-1.07629	-1.23223
H	5.82771	-2.72981	-1.43924
H	3.57171	-2.19272	-0.67595

I-4 Energy: -734945.1608265

C	-1.83420	-0.05574	-2.03476
N	1.24411	-0.01515	-1.92530
C	-2.24873	0.60888	0.36124
C	-1.73785	-0.57814	-0.53712
C	-0.32611	-1.16859	-0.47252
C	0.32891	-1.13794	-1.85587
C	-0.85841	-0.95741	-2.79429
O	-3.39920	0.55064	0.72255
N	0.28066	-1.71605	0.51841
C	0.01495	-1.98509	1.98517
C	1.33626	-1.65101	2.68780
C	-1.12104	-1.16389	2.57292
C	-0.29272	-3.48647	2.06438
C	-3.24127	-0.00677	-2.60436
C	-2.63169	-1.72255	-0.34699
N	-3.29786	-2.64957	-0.21852
C	-1.41771	1.81865	0.57412
C	-2.10921	3.02611	0.74669
C	-1.41678	4.20222	0.98080
C	-0.02694	4.18618	1.07063
C	0.66626	2.99140	0.91385
C	-0.02249	1.81479	0.65300
C	2.47514	0.02634	-1.27375
C	3.17515	-1.13457	-0.91733
C	4.40740	-1.04283	-0.27951
C	4.97129	0.19415	0.00575
C	4.28878	1.34998	-0.36772
C	3.05551	1.27229	-0.99446
H	-1.43782	0.96192	-2.00771
H	0.81986	0.88135	-2.10608
H	0.86283	-2.06635	-2.05039
H	-1.31948	-1.92753	-2.98738
H	-0.55469	-0.52897	-3.74772
H	1.24038	-1.89041	3.74576
H	2.16445	-2.23581	2.28429
H	1.57611	-0.59184	2.59321
H	-1.18791	-1.41739	3.63026
H	-2.08586	-1.39888	2.13177
H	-0.92729	-0.09527	2.51182
H	-1.21799	-3.72206	1.53967
H	0.51698	-4.08282	1.64262
H	-0.40655	-3.76112	3.11272
H	-3.20542	0.42940	-3.60257
H	-3.67193	-1.00545	-2.68470
H	-3.90016	0.60696	-1.99013
H	-3.18821	3.02661	0.68475
H	-1.95884	5.13109	1.09795
H	0.51427	5.10273	1.26569
H	1.74363	2.96976	0.99883
H	0.54670	0.90513	0.55520
H	2.79225	-2.11570	-1.16647
H	4.92752	-1.95314	-0.01069
H	5.92797	0.25914	0.50602
H	4.71554	2.32335	-0.16188
H	2.53405	2.17794	-1.27746
H	1.17791	-2.12234	0.26419

I-4’ Energy: -734949.3200847

C	-0.54021	-2.00540	0.98362
N	2.78534	-0.25904	0.82316
C	-1.68667	-0.45502	-0.72584
C	-0.96022	-0.48694	0.66563
C	0.39332	0.20206	0.49248
C	1.50349	-0.82879	0.51310
C	0.92260	-1.90710	1.43084
O	-0.93701	-0.41373	-1.67922
N	0.70969	1.43301	0.42604
C	0.00371	2.76290	0.34996

C	-0.02151	3.33592	1.77151
C	0.89381	3.61357	-0.56481
C	-1.39387	2.64980	-0.24816
C	-1.43598	-2.75759	1.95253
C	-1.64980	0.10827	1.80158
N	-2.15381	0.56244	2.72941
C	-3.15237	-0.51617	-0.86496
C	-3.67684	-0.22974	-2.13583
C	-5.04156	-0.27807	-2.35593
C	-5.90275	-0.62718	-1.31667
C	-5.39311	-0.92183	-0.05701
C	-4.02606	-0.85978	0.17307
C	3.90801	-0.45246	-0.00897
C	5.16880	-0.61579	0.57273
C	6.29707	-0.75568	-0.22426
C	6.18874	-0.74795	-1.61191
C	4.93499	-0.58535	-2.19133
C	3.80088	-0.42847	-1.40255
H	-0.56271	-2.51137	0.01752
H	3.00714	-0.30093	1.80803
H	1.51777	-1.23299	-0.50499
H	0.99089	-1.59906	2.47765
H	1.44089	-2.85656	1.31565
H	-0.43919	4.34191	1.73242
H	-0.63921	2.73007	2.43126
H	0.98458	3.39960	2.18713
H	0.46756	4.61266	-0.64163
H	0.95075	3.18325	-1.56499
H	1.90380	3.70880	-0.16220
H	-1.36544	2.20305	-1.24016
H	-2.08473	2.09979	0.38577
H	-1.79440	3.65775	-0.34674
H	-1.02893	-3.75924	2.09400
H	-1.47787	-2.27086	2.92708
H	-2.44941	-2.86845	1.56983
H	-3.00000	0.03622	-2.93533
H	-5.43818	-0.04552	-3.33519
H	-6.97027	-0.66778	-1.49005
H	-6.05898	-1.19796	0.74931
H	-3.66071	-1.09176	1.15988
H	5.26147	-0.63788	1.65232
H	7.26457	-0.88204	0.24502
H	7.06813	-0.86363	-2.23141
H	4.83330	-0.56682	-3.26905
H	2.84324	-0.26817	-1.88118
H	1.73662	1.52366	0.44042

I-5 Energy: -734670.2985892

C	1.27777	-2.62713	-0.44132
C	-0.05790	-2.49430	-1.19162
C	-0.72466	-1.13034	-0.95341
C	-0.56533	-0.72330	0.50967
O	0.67336	-0.16234	0.84822
C	1.79086	-0.21980	0.06175
C	2.11550	-1.35996	-0.59740
H	1.05072	-2.70078	0.62627
H	0.08734	-2.62744	-2.26569
H	-0.72512	-3.28913	-0.84799
H	-0.18863	-0.39476	-1.55485
N	-2.09850	-1.16027	-1.39039
N	-1.48401	-0.84145	1.34619
C	-1.40887	-0.47054	2.77202
C	-0.30240	-1.25842	3.49169
H	0.68666	-0.97774	3.13505
H	-0.34979	-1.06284	4.56446
H	-0.43770	-2.33051	3.33529
C	-2.76881	-0.84408	3.37602
H	-2.95336	-1.91444	3.26705
H	-2.80125	-0.59351	4.43792
H	-3.57091	-0.30644	2.86737
C	-1.18925	1.04398	2.91936
H	-1.26081	1.32522	3.97163
H	-0.21114	1.34327	2.54809
H	-1.95401	1.59226	2.36611
C	3.88944	3.49314	0.13501
C	4.60379	2.29888	0.12376
C	3.93334	1.08358	0.10145
C	2.53498	1.04880	0.08732
C	1.82338	2.25517	0.12326
C	2.49753	3.46753	0.13719
H	4.41486	4.43931	0.15231
H	5.68574	2.31254	0.14305
H	4.49733	0.16190	0.11891
H	0.74243	2.23843	0.13463
H	1.93662	4.39296	0.15318
C	3.18694	-1.37707	-1.52363
N	4.03501	-1.45526	-2.30496
H	-2.60568	-1.94717	-1.01180
C	-4.52041	2.27740	-1.64186
C	-3.13603	2.40077	-1.64077
C	-2.31542	1.28186	-1.54075
C	-2.87219	-0.00068	-1.44420
C	-4.27133	-0.11764	-1.44318
C	-5.07929	1.00422	-1.54176
H	-5.15230	3.15262	-1.71565
H	-2.67923	3.38034	-1.71286
H	-1.24379	1.42036	-1.53966
H	-4.71962	-1.10149	-1.36291
H	-6.15567	0.88309	-1.53543
C	2.01789	-3.90107	-0.84319
H	2.26148	-3.90118	-1.90691
H	2.94509	-4.01404	-0.28003
H	1.38916	-4.76918	-0.64148

I-5’ Energy: -734954.1241153

C	1.20656	-2.71269	0.05786
C	-0.22681	-2.66339	-0.49921
C	-0.83763	-1.24717	-0.48250
C	-0.41641	-0.52448	0.78623
O	0.81096	-0.08048	0.94890
C	1.83599	-0.28314	0.00273
C	2.03173	-1.53537	-0.45652
H	1.14884	-2.58358	1.14263
H	-0.25422	-3.01773	-1.53043
H	-0.84849	-3.32978	0.10209
H	-0.39721	-0.67722	-1.30777
N	-2.27124	-1.29204	-0.58678
N	-1.27160	-0.31373	1.72353
C	-1.10306	0.36478	3.05427
C	-0.08745	-0.42212	3.88749
H	0.90236	-0.40419	3.43500
H	-0.01766	0.03270	4.87532
H	-0.40406	-1.45880	4.00628
C	-2.48638	0.32798	3.70523
H	-2.83367	-0.69811	3.83707
H	-2.43222	0.79666	4.68649
H	-3.21613	0.87570	3.10644
C	-0.65554	1.81282	2.83116
H	-0.64712	2.32500	3.79300
H	0.34612	1.86661	2.41088
H	-1.34753	2.33433	2.16915
C	3.89768	3.34524	-0.81487
C	4.59936	2.14380	-0.77769
C	3.93736	0.95609	-0.50558
C	2.55657	0.95774	-0.27164
C	1.86087	2.17481	-0.28971
C	2.52817	3.35780	-0.56703
H	4.41829	4.27004	-1.02645
H	5.66747	2.13237	-0.94917
H	4.49709	0.03377	-0.44996
H	0.79795	2.19659	-0.09557
H	1.97926	4.28980	-0.58812
C	2.97267	-1.77529	-1.49258
N	3.70516	-2.03396	-2.34537
H	-2.64821	-2.21799	-0.71613
C	-4.57729	1.74286	-2.37346
C	-3.28032	2.01272	-1.95776
C	-2.49580	1.02066	-1.37608
C	-3.00707	-0.26937	-1.20685
C	-4.31616	-0.53915	-1.62625
C	-5.08816	0.45737	-2.20223
H	-5.18230	2.51834	-2.82386
H	-2.86464	3.00467	-2.08138
H	-1.49247	1.27315	-1.06295
H	-4.72301	-1.53536	-1.49858
H	-6.09622	0.22653	-2.52276
C	1.85101	-4.07099	-0.20963
H	1.92705	-4.27096	-1.27916
H	2.84996	-4.12301	0.22320
H	1.24360	-4.85654	0.23999
H	-2.19603	-0.67009	1.48153

I-6 Energy: -1018925.8781623

C	0.73298	-0.31603	2.45330
N	2.23628	0.26691	-0.21639
C	-0.50152	0.19465	1.63495
C	0.11360	1.17649	0.56718
C	1.63821	1.17294	0.75203
C	1.80276	0.75053	2.21444
N	-0.64975	1.82826	-0.17979
C	-0.35581	2.81073	-1.23007
C	-0.86796	2.18352	-2.53844
C	-1.21645	4.03629	-0.87991
C	1.10104	3.25483	-1.42735
C	0.50912	-0.62344	3.92793
C	-1.39331	0.97893	2.48730
N	-2.05413	1.62039	3.17679
C	-0.57123	-1.74556	-0.07708
C	-0.62918	-1.31249	-1.40328
C	0.08846	-1.97189	-2.39314
C	0.87299	-3.07488	-2.07120
C	0.92790	-3.51962	-0.75506
C	0.20830	-2.86112	0.23636
C	3.60115	0.07130	-0.32637
C	4.07960	-1.03675	-1.04900
C	5.43925	-1.23320	-1.22696
C	6.36648	-0.34071	-0.68914
C	5.90000	0.75223	0.03237
C	4.53765	0.96247	0.22016
H	1.06416	-1.22876	1.96016
H	1.66555	-0.50906	-0.50968
H	2.03459	2.16970	0.59368
H	1.62345	1.61491	2.86080
H	2.80349	0.37750	2.42827
H	-0.83222	2.92347	-3.33954
H	-0.24407	1.33715	-2.82915
H	-1.89633	1.83943	-2.42671
H	-1.12258	4.79489	-1.65883
H	-0.89223	4.47306	0.06657
H	-2.26557	3.75422	-0.78782
H	1.48053	3.79010	-0.55673
H	1.75364	2.40970	-1.64426
H	1.14575	3.94058	-2.27507
H	1.44052	-0.99097	4.36263
H	0.21532	0.27213	4.47825
H	-0.25866	-1.38176	4.06584
H	-1.23839	-0.45820	-1.65112
H	0.03592	-1.62069	-3.41536

H	1.43726	-3.58474	-2.84113
H	1.53420	-4.37722	-0.49500
H	0.26355	-3.21854	1.25557
H	3.36909	-1.73834	-1.46968
H	5.77900	-2.09512	-1.78851
H	7.42793	-0.49849	-0.82741
H	6.60236	1.45558	0.46322
H	4.20961	1.81962	0.79107
C	-1.33923	-0.98551	1.00382
O	-1.68624	-1.81635	2.08320
H	-1.91926	-2.69800	1.76599
C	-3.53630	-1.21881	0.05663
C	-4.47903	-0.39580	-0.79531
O	-2.49207	-0.36378	0.45992
H	-4.10013	-1.60629	0.90816
H	-3.18971	-2.05431	-0.55809
F	-4.97307	0.66787	-0.13535
F	-5.52455	-1.15369	-1.18552
F	-3.88057	0.07092	-1.91027

TS-1 Energy: -734648.3936581

C	1.80185	1.63765	-0.16910
C	1.48923	0.73271	-1.19366
C	2.54390	1.29103	0.96773
C	1.25127	3.05553	-0.22485
C	0.04166	3.29374	0.70433
N	-1.43334	1.77333	-0.54834
N	3.13342	1.07459	1.94730
H	0.91255	3.19808	-1.25192
H	-0.39853	4.26917	0.46885
H	0.36486	3.34360	1.74542
C	2.93180	-3.32105	-1.28782
C	1.64499	-3.00254	-1.71335
C	1.21086	-1.68417	-1.68727
C	2.04090	-0.66247	-1.21346
C	3.33693	-0.99097	-0.80804
C	3.77857	-2.30931	-0.84585
H	3.27449	-4.34791	-1.30801
H	0.97970	-3.78228	-2.06280
H	0.21673	-1.42703	-2.02642
H	4.00971	-0.21528	-0.47147
H	4.78777	-2.54543	-0.53230
C	-4.62632	-0.86535	-1.25469
C	-4.62404	-0.09065	-0.09987
C	-3.58777	0.79937	0.15631
C	-2.53687	0.92001	-0.75513
C	-2.53790	0.14322	-1.91777
C	-3.57803	-0.74146	-2.16212
H	-5.43456	-1.55924	-1.44425
H	-5.43214	-0.17896	0.61475
H	-3.61187	1.38463	1.06460
H	-1.70844	0.22765	-2.60781
H	-3.56413	-1.34201	-3.06256
O	0.67272	1.03045	-2.12756
H	-0.67002	1.64484	-1.24413
C	-1.09331	2.31091	0.62980
H	-1.90954	2.53724	1.30339
C	-0.52885	0.77803	1.69232
N	-0.56490	-0.35814	1.88266
C	-0.60736	-1.79411	2.09766
C	0.83562	-2.31020	2.07985
H	1.31249	-2.09992	1.12415
H	1.41993	-1.85029	2.87626
H	0.82088	-3.38897	2.23396
C	-1.43532	-2.39536	0.95455
H	-1.49495	-3.47411	1.09808
H	-2.44390	-1.98286	0.94580
H	-0.96571	-2.19664	-0.00663
C	-1.27550	-2.03572	3.45555
H	-0.70136	-1.57297	4.25811
H	-2.28878	-1.63399	3.46553
H	-1.32433	-3.10940	3.63562
C	2.31690	4.11882	0.06498
H	2.71119	4.01689	1.07866
H	3.15005	4.02314	-0.63242
H	1.90065	5.12390	-0.03395

TS-1’ Energy: -734643.6757470

C	1.87795	-1.34909	-0.61845
C	1.89788	-0.29180	0.29100
C	2.98071	-1.64628	-1.43387
C	0.62138	-2.18462	-0.83122
C	-0.21467	-1.75266	-2.06328
N	-0.90137	0.42876	-1.13852
N	3.84635	-1.93309	-2.15614
H	0.01093	-2.03817	0.06228
H	-0.75581	-2.60581	-2.47441
H	0.45120	-1.39692	-2.85576
C	5.47310	1.89412	1.25407
C	4.27908	2.56012	0.98672
C	3.13395	1.83784	0.67683
C	3.16708	0.44204	0.60462
C	4.36742	-0.21632	0.87928
C	5.51164	0.50417	1.20627
H	6.36493	2.45505	1.50403
H	4.24133	3.64184	1.02399
H	2.20005	2.34932	0.48361
H	4.40255	-1.29721	0.85018
H	6.43244	-0.02104	1.42773
C	-3.17030	3.92937	-0.65903
C	-3.58210	2.97162	-1.57900
C	-2.85226	1.80262	-1.76363
C	-1.68862	1.58750	-1.02087
C	-1.27705	2.54766	-0.08898

C	-2.01390	3.70890	0.08545
H	-3.74518	4.83553	-0.52067
H	-4.48226	3.12836	-2.15939
H	-3.20149	1.07372	-2.48116
H	-0.38633	2.36144	0.49707
H	-1.68525	4.44325	0.80968
O	0.83916	0.12722	0.87486
H	-0.21967	0.29355	-0.34953
C	-1.23246	-0.65664	-1.85466
H	-1.85435	-0.47868	-2.72195
C	-2.53540	-1.48627	-0.77420
N	-2.93738	-1.53816	0.30537
C	-3.29304	-1.46285	1.71285
C	-2.49166	-0.28863	2.29562
H	-1.42132	-0.43185	2.14468
H	-2.78653	0.64948	1.82580
H	-2.69635	-0.22564	3.36448
C	-4.80191	-1.21901	1.80575
H	-5.35748	-2.04348	1.35927
H	-5.07864	-1.14075	2.85678
H	-5.07584	-0.29157	1.30318
C	-2.89507	-2.79268	2.36143
H	-3.42822	-3.62421	1.90090
H	-1.82261	-2.96326	2.27018
H	-3.15320	-2.75608	3.41940
C	0.94101	-3.68183	-0.93082
H	1.51163	-3.90559	-1.83490
H	1.53086	-4.00518	-0.07208
H	0.02160	-4.27002	-0.95852

TS-2 Energy: -734654.9432192

C	1.15293	2.78547	-0.99735
N	-1.73801	1.79069	-0.83742
C	1.09532	0.29725	-1.54239
C	1.46220	1.34067	-0.59344
C	-0.15865	1.14241	0.89582
C	-0.96135	2.27471	0.28356
C	0.02671	3.35939	-0.10731
O	0.34245	0.53788	-2.50021
N	-0.17568	0.37847	1.78578
C	0.16705	-0.65056	2.74632
C	0.91447	0.05202	3.88870
C	-1.15184	-1.25954	3.24147
C	1.04831	-1.71948	2.09291
C	2.36761	3.71367	-1.00556
C	2.50607	1.14959	0.33908
N	3.32187	1.04822	1.15582
C	1.51913	-1.12552	-1.34537
C	0.53681	-2.11040	-1.49396
C	0.85639	-3.45165	-1.34070
C	2.16907	-3.82771	-1.06492
C	3.15606	-2.85562	-0.94453
C	2.83289	-1.50897	-1.07627
C	-2.64984	0.73839	-0.68188
C	-2.92932	-0.07803	-1.78801
C	-3.84610	-1.11367	-1.68835
C	-4.50305	-1.36781	-0.48604
C	-4.22549	-0.56427	0.61397
C	-3.30990	0.47851	0.52541
H	0.77603	2.72256	-2.01797
H	-1.16908	1.66833	-1.66848
H	-1.62147	2.63466	1.07443
H	0.45389	3.76862	0.81195
H	-0.49658	4.16952	-0.61639
H	1.16308	-0.68700	4.65042
H	1.83559	0.50225	3.51935
H	0.29329	0.82458	4.34209
H	-0.93434	-2.03258	3.97854
H	-1.70181	-1.70867	2.41433
H	-1.77975	-0.49861	3.70506
H	0.53366	-2.19298	1.25924
H	1.97842	-1.28840	1.73116
H	1.27932	-2.48201	2.83746
H	2.07314	4.71109	-1.33827
H	2.80424	3.80760	-0.00919
H	3.13923	3.34309	-1.68191
H	-0.47929	-1.80867	-1.71256
H	0.08388	-4.20428	-1.43510
H	2.42130	-4.87420	-0.94937
H	4.18050	-3.14378	-0.74609
H	3.60937	-0.76359	-0.98212
H	-2.40734	0.10017	-2.71984
H	-4.04142	-1.73281	-2.55527
H	-5.21423	-2.17965	-0.40872
H	-4.71967	-0.74988	1.55963
H	-3.10958	1.07673	1.40308

TS-2’ Energy: -734649.5575376

C	-0.33251	-2.11792	-1.46350
N	-2.54951	0.76213	-0.19374
C	1.29338	-1.64778	0.44220
C	0.93164	-1.44373	-0.93166
C	-0.19101	0.62126	-0.37988
C	-1.44873	-0.16469	-0.32656
C	-1.48595	-1.10499	-1.54198
O	0.50151	-2.17000	1.25392
N	0.40906	1.60989	-0.40977
C	1.30311	2.74829	-0.52536
C	1.31738	3.14433	-2.00739
C	0.72692	3.86809	0.34955
C	2.69786	2.32912	-0.04856
C	-0.13889	-2.84167	-2.79524
C	1.78897	-0.84154	-1.86460
N	2.43067	-0.30353	-2.67009

C	2.61040	-1.15653	0.97307
C	2.61919	-0.53145	2.22351
C	3.80462	-0.06499	2.77425
C	5.00590	-0.23962	2.09124
C	5.01113	-0.88557	0.85951
C	3.81982	-1.33681	0.30065
C	-3.78780	0.35544	0.30942
C	-4.89000	1.20856	0.14139
C	-6.13046	0.87262	0.65963
C	-6.31019	-0.31875	1.36072
C	-5.22316	-1.16785	1.52607
C	-3.97327	-0.84553	1.00551
H	-0.60331	-2.85305	-0.70398
H	-2.61054	1.43500	-0.94449
H	-1.36517	-0.77032	0.57864
H	-1.42358	-0.50556	-2.45545
H	-2.45171	-1.61201	-1.54702
H	1.97387	4.00573	-2.12776
H	1.69419	2.32316	-2.61600
H	0.31787	3.41724	-2.34604
H	1.37298	4.74222	0.27021
H	0.68531	3.55999	1.39428
H	-0.27510	4.14252	0.02007
H	2.67558	2.00744	0.99111
H	3.08660	1.51649	-0.65868
H	3.36243	3.18879	-0.13526
H	-1.06662	-3.33190	-3.09813
H	0.14696	-2.14650	-3.58767
H	0.63921	-3.60250	-2.71529
H	1.68275	-0.40818	2.75129
H	3.79386	0.43518	3.73455
H	5.93269	0.11988	2.52031
H	5.94375	-1.04011	0.33167
H	3.83760	-1.84163	-0.65492
H	-4.76207	2.14101	-0.39595
H	-6.96406	1.54869	0.51466
H	-7.27935	-0.57884	1.76517
H	-5.34122	-2.10195	2.06126
H	-3.15666	-1.53988	1.14556

TS-3 Energy: -734930.4540063

C	-1.04999	-0.90300	1.60710
N	2.04305	-0.83362	-0.50411
C	-1.92660	-0.11993	-1.37661
C	-1.01436	0.45809	0.89674
C	0.25444	0.67829	0.34527
C	1.11200	-0.57192	0.56523
C	0.08907	-1.66122	0.90419
O	-1.20231	0.31725	-2.14111
C	0.49353	3.11326	-0.47427
C	1.66335	3.70128	-1.27073
C	-0.80525	3.31177	-1.26059
C	0.42805	3.77087	0.90964
C	-0.85449	-0.78550	3.12275
C	-1.97484	1.42221	1.26070
N	-2.82293	2.13798	1.58736
C	-3.05941	-0.85555	-0.96649
C	-3.00613	-2.25710	-1.08186
C	-4.11090	-2.99023	-0.69035
C	-5.24189	-2.34462	-0.19477
C	-5.28221	-0.95555	-0.08661
C	-4.19072	-0.19348	-0.45904
C	3.40171	-1.10072	-0.28024
C	4.07185	-1.99875	-1.12091
C	5.42868	-2.24186	-0.95793
C	6.14724	-1.60718	0.05158
C	5.48561	-0.71410	0.88787
C	4.13042	-0.45183	0.72399
H	-2.01041	-1.38964	1.42729
H	1.64570	-1.37927	-1.25564
H	1.68622	-0.34699	1.46651
H	0.51801	-2.44761	1.52209
H	-0.26700	-2.12326	-0.01906
H	1.50163	4.76762	-1.42149
H	2.60618	3.57349	-0.73526
H	1.74685	3.22775	-2.25079
H	-0.98228	4.38073	-1.38112
H	-1.66911	2.89515	-0.74924
H	-0.72896	2.86299	-2.25005
H	-0.38318	3.37098	1.51338
H	1.36677	3.62443	1.44497
H	0.26072	4.84180	0.79107
H	-0.89542	-1.77397	3.58222
H	0.10770	-0.33258	3.36940
H	-1.63910	-0.17190	3.56762
H	-2.12594	-2.74532	-1.47396
H	-4.08977	-4.06776	-0.77410
H	-6.09994	-2.92912	0.10876
H	-6.16762	-0.46470	0.29232
H	-4.20642	0.88171	-0.37407
H	3.52115	-2.51008	-1.90201
H	5.92386	-2.94125	-1.62004
H	7.20334	-1.80280	0.18164
H	6.02859	-0.20231	1.67272
H	3.65265	0.27442	1.36819
H	1.71509	1.38568	-0.70002
N	0.81761	1.66486	-0.31188

TS-3’ Energy: -734929.4749158

C	-1.40833	-1.07113	1.35936
N	1.67644	-1.94611	0.56259
C	-2.12414	0.68365	-1.29442
C	-0.84728	0.24505	0.82180
C	0.29465	0.02107	0.04454

C	0.48757	-1.48748	-0.16154
C	-0.80013	-2.10729	0.39346
O	-1.38485	1.17887	-2.00701
C	1.63337	2.22452	-0.19468
C	2.93933	2.39778	-0.98123
C	0.61116	3.26085	-0.66801
C	1.92881	2.34072	1.30492
C	-1.06420	-1.30116	2.83505
C	-1.25566	1.44027	1.45163
N	-1.68612	2.36898	1.99029
C	-3.37209	0.12511	-0.93901
C	-3.76665	-1.03776	-1.62725
C	-4.98602	-1.60733	-1.30976
C	-5.79693	-1.03105	-0.33465
C	-5.39555	0.12193	0.33859
C	-4.17525	0.70621	0.05668
C	2.96318	-1.58207	0.13584
C	3.91572	-1.15539	1.06932
C	5.19901	-0.81386	0.66452
C	5.55757	-0.87216	-0.67907
C	4.61639	-1.29896	-1.61071
C	3.33487	-1.66021	-1.21365
H	-2.49656	-1.07217	1.27391
H	1.56661	-1.85018	1.56275
H	0.61437	-1.71825	-1.21747
H	-0.60068	-3.06232	0.87579
H	-1.48960	-2.29610	-0.42809
H	3.34429	3.39220	-0.79895
H	3.68528	1.66323	-0.67072
H	2.76772	2.29346	-2.05446
H	1.00332	4.25999	-0.47533
H	-0.33796	3.17324	-0.14655
H	0.43353	3.16070	-1.73837
H	1.03792	2.21600	1.91463
H	2.66482	1.59327	1.60298
H	2.33594	3.33076	1.51189
H	-1.45658	-2.26444	3.16334
H	0.01352	-1.29329	3.00783
H	-1.50384	-0.52394	3.46186
H	-3.13123	-1.46999	-2.38648
H	-5.30539	-2.50059	-1.82807
H	-6.74974	-1.48439	-0.09677
H	-6.03481	0.56328	1.09018
H	-3.84808	1.59198	0.57910
H	3.63996	-1.08032	2.11455
H	5.91678	-0.48208	1.40413
H	6.55317	-0.59022	-0.99446
H	4.87976	-1.35891	-2.65914
H	2.62860	-2.01030	-1.95480
H	1.82516	0.35762	-1.12548
N	1.17910	0.83183	-0.51031

TS-4 Energy: -734647.3331859

C	1.52684	-2.85812	-0.78895
C	0.32899	-2.65534	-1.74278
C	-0.84662	-1.79928	-1.25959
C	-0.71164	-0.32800	-1.54591
O	1.41587	-0.07240	-1.61800
C	1.86662	-0.33925	-0.44452
C	1.95571	-1.64026	0.03167
H	1.22126	-3.62134	-0.06721
H	0.66987	-2.23005	-2.68436
H	-0.07498	-3.64525	-1.96282
H	-1.72078	-2.08140	-1.85170
N	-1.14837	-1.97441	0.14642
N	-1.20629	0.71732	-1.58670
C	-1.35876	2.16259	-1.56721
C	-0.27525	2.76300	-2.46949
H	-0.41024	2.43879	-3.50160
H	-0.35345	3.84967	-2.43169
H	0.71152	2.45637	-2.12813
C	-1.17381	2.59536	-0.10571
H	-0.17255	2.35031	0.24573
H	-1.31587	3.67403	-0.04331
H	-1.90624	2.10663	0.53707
C	-2.76700	2.49079	-2.06967
H	-2.90458	3.57179	-2.05698
H	-2.90731	2.13476	-3.09050
H	-3.52122	2.03407	-1.42862
C	2.78711	3.19210	1.82434
C	2.11926	2.13765	2.43798
C	1.85077	0.97208	1.72819
C	2.23717	0.84824	0.39182
C	2.89178	1.92121	-0.22052
C	3.17603	3.07870	0.49152
H	2.99648	4.09918	2.37714
H	1.79701	2.22449	3.46803
H	1.31317	0.16682	2.20907
H	3.16704	1.83967	-1.26358
H	3.69285	3.89700	0.00586
C	2.58193	-1.94254	1.25334
N	3.09272	-2.26631	2.24584
H	-0.31044	-1.91274	0.70967
C	-4.42381	-0.17315	2.08428
C	-4.49753	-0.46491	0.72715
C	-3.42700	-1.05351	0.06332
C	-2.25130	-1.36149	0.75706
C	-2.17993	-1.06362	2.12490
C	-3.25557	-0.48096	2.77782
H	-5.25934	0.28885	2.59321
H	-5.39562	-0.22986	0.16971
H	-3.51534	-1.25978	-0.99420
H	-1.27225	-1.29033	2.67202
H	-3.17575	-0.25774	3.83438

C	2.70443	-3.43782	-1.58737
H	3.06705	-2.70393	-2.30985
H	3.52837	-3.69918	-0.92167
H	2.40750	-4.33863	-2.13063

TS-5 Energy: -734925.4773981

C	0.42952	1.19589	-0.01202
C	-0.03335	2.38710	-0.45410
C	-1.02229	-0.04102	-1.78877
C	-1.23869	2.53953	-1.38360
C	-1.45109	1.33285	-2.33290
H	-1.30060	-0.83539	-2.48061
H	-1.02748	3.39317	-2.02973
H	-0.89576	1.48287	-3.26029
H	-2.50235	1.27516	-2.61154
C	0.53148	3.57509	0.05874
N	0.95360	4.58192	0.44339
C	1.58739	0.86592	0.79890
C	2.81574	1.45537	0.46152
C	1.53748	-0.05221	1.85460
C	3.95837	1.15490	1.18559
H	2.87224	2.13959	-0.37378
C	2.68842	-0.35448	2.56762
H	0.60409	-0.52305	2.11737
C	3.89861	0.24765	2.23903
H	4.89809	1.61929	0.91781
H	2.63846	-1.06390	3.38298
H	4.79356	0.00615	2.79753
O	-0.93234	0.16438	0.55616
C	-1.61288	-0.28268	-0.40721
N	-2.75516	-0.90133	-0.26424
C	-3.44436	-1.26016	1.02009
N	0.42281	-0.01921	-1.55662
H	0.89145	0.59005	-2.21813
C	1.15900	-1.23362	-1.38380
C	0.56964	-2.39686	-0.89659
C	2.52708	-1.19884	-1.65301
C	1.35553	-3.52174	-0.67808
H	-0.48732	-2.45329	-0.68645
C	3.30260	-2.32605	-1.42907
H	2.98257	-0.29337	-2.03236
C	2.72066	-3.49038	-0.93675
H	0.89239	-4.42372	-0.30112
H	4.36320	-2.29229	-1.63905
H	3.32723	-4.36848	-0.76009
C	-2.53787	-2.20460	1.81614
H	-1.60406	-1.71877	2.09404
H	-3.04956	-2.50449	2.73051
H	-2.31083	-3.10228	1.23982
C	-4.73598	-1.97085	0.61366
H	-5.37477	-1.31468	0.01962
H	-4.52573	-2.87549	0.03987
H	-5.28491	-2.25896	1.50897
C	-3.76198	0.00929	1.81411
H	-2.85583	0.54112	2.09795
H	-4.40179	0.67738	1.23702
H	-4.29285	-0.26903	2.72458
C	-2.50518	2.88730	-0.58344
H	-2.33764	3.77851	0.02208
H	-3.33625	3.08736	-1.26139
H	-2.79612	2.08205	0.08824
H	-3.19657	-1.22463	-1.11485

TS-6 Energy: -734637.0170055

C	0.42173	1.01350	-0.20505
C	-0.00755	2.26763	-0.63281
C	-1.03893	-0.14078	-1.74917
C	-1.19991	2.43323	-1.58036
C	-1.44866	1.16409	-2.43033
H	-1.18767	-0.99638	-2.40101
H	-0.96293	3.23696	-2.28207
H	-0.90467	1.22847	-3.37601
H	-2.50529	1.08383	-2.68405
C	0.40496	3.40822	0.06612
N	0.71542	4.39526	0.59742
C	1.60136	0.85972	0.69255
C	2.80551	1.46223	0.31493
C	1.55880	0.10529	1.86543
C	3.94240	1.32012	1.09867
H	2.84893	2.04217	-0.59706
C	2.69795	-0.03117	2.65036
H	0.63666	-0.36942	2.16014
C	3.89163	0.57255	2.27072
H	4.86680	1.79219	0.79195
H	2.65025	-0.61415	3.56119
H	4.77719	0.46054	2.88317
O	-0.90063	0.11129	0.57290
C	-1.67693	-0.35771	-0.38134
N	-2.80003	-0.94668	-0.30601
C	-3.45691	-1.20262	0.99203
N	0.42409	-0.02111	-1.41203
H	0.88746	0.48744	-2.16368
C	1.17278	-1.27150	-1.22270
C	0.64123	-2.34796	-0.52629
C	2.45370	-1.32369	-1.75707
C	1.41509	-3.49107	-0.36550
H	-0.35111	-2.31322	-0.10726
C	3.21911	-2.47002	-1.58754
H	2.85865	-0.47764	-2.29762
C	2.70175	-3.55485	-0.88874
H	1.00509	-4.33265	0.17662
H	4.21715	-2.51055	-2.00244
H	3.29789	-4.44778	-0.75494
C	-2.58266	-2.11601	1.86806

H	-1.65037	-1.62416	2.14225
H	-3.11432	-2.38169	2.78415
H	-2.34547	-3.03937	1.33471
C	-4.77111	-1.92527	0.67086
H	-5.40232	-1.30159	0.03469
H	-4.57308	-2.85932	0.14120
H	-5.32204	-2.15601	1.58512
C	-3.77128	0.10545	1.73490
H	-2.85881	0.64208	1.98981
H	-4.39466	0.75335	1.11555
H	-4.31697	-0.10834	2.65651
C	-2.46852	2.85703	-0.82041
H	-2.28843	3.77239	-0.25473
H	-3.28702	3.04586	-1.51892
H	-2.78541	2.08582	-0.12013

TS-7 Energy: -1303154.7412186

C	0.26320	-2.12728	-1.99247
N	2.16323	0.23004	-2.60423
C	0.64077	-1.66182	-0.51929
C	2.24953	-1.35564	-0.64955
C	2.49505	-1.11404	-2.14948
C	1.58267	-2.14830	-2.78710
N	3.21305	-1.42420	0.12815
C	3.54971	-1.71900	1.51831
C	3.82033	-3.23204	1.58671
C	4.85757	-0.95165	1.78470
C	2.52015	-1.30785	2.57295
C	-0.50148	-3.43960	-2.09575
C	0.40910	-2.73451	0.42629
N	0.21908	-3.63420	1.11688
C	-0.16694	0.15101	1.24211
C	0.33688	1.44853	1.41700
C	0.32972	2.03689	2.67314
C	-0.19579	1.34896	3.76066
C	-0.70162	0.06254	3.59061
C	-0.67907	-0.53968	2.34327
C	2.62145	1.37827	-1.96375
C	1.86349	2.55784	-2.04916
C	2.29065	3.72302	-1.43049
C	3.48205	3.74790	-0.70848
C	4.24075	2.58461	-0.62726
C	3.82448	1.41075	-1.24422
H	-0.38154	-1.34497	-2.38080
H	1.20990	0.32007	-2.91731
H	3.54829	-1.31484	-2.33997
H	2.05418	-3.12921	-2.69821
H	1.40944	-1.95777	-3.84678
H	4.19514	-3.48543	2.57946
H	2.90877	-3.79972	1.40490
H	4.56948	-3.52218	0.84925
H	5.22560	-1.17002	2.78834
H	4.68954	0.12365	1.70436
H	5.62110	-1.23490	1.05982
H	2.27528	-0.25068	2.48563
H	1.60901	-1.89084	2.50727
H	2.94562	-1.47362	3.56399
H	-0.71677	-3.63375	-3.14822
H	0.07838	-4.28087	-1.71341
H	-1.44876	-3.38860	-1.56273
H	0.75456	1.98170	0.57402
H	0.73263	3.03295	2.79817
H	-0.21113	1.81109	4.73904
H	-1.11424	-0.47471	4.43398
H	-1.06737	-1.53738	2.23053
H	0.92655	2.54837	-2.59288
H	1.67995	4.61456	-1.50330
H	3.81007	4.65534	-0.21874
H	5.16936	2.58221	-0.06936
H	4.42057	0.51623	-1.14405
C	-0.09385	-0.38934	-0.13123
O	-0.23169	0.45558	-1.08693
H	-1.06495	1.08856	-0.89973
H	-2.52991	0.22511	-0.50204
C	-2.96365	2.09052	0.34547
H	-2.49521	1.84609	1.30335
H	-4.04744	1.99499	0.44755
C	-2.96709	-1.85301	0.44731
H	-2.50868	-2.81778	0.69646
H	-3.25705	-1.36607	1.38973
O	-2.15470	-1.07627	-0.35975
O	-2.47915	1.30361	-0.71324
C	-4.27059	-2.17672	-0.26551
C	-2.66289	3.54661	0.05864
F	-4.07909	-2.86942	-1.40843
F	-5.08232	-2.92293	0.51920
F	-4.95772	-1.05963	-0.59742
F	-1.34032	3.76499	-0.11435
F	-3.06915	4.32412	1.08365
F	-3.28039	3.99019	-1.05434

2 Energy: -518493.1757332

C	-2.36719	-2.23107	-0.18223
N	1.18834	-1.36495	-0.79316
C	-2.28708	-0.70209	-0.15553
C	-0.98867	-0.26445	-0.11052
C	-0.05415	-1.48954	-0.07377
C	-0.94099	-2.61840	-0.60433
C	-0.93025	2.31242	-0.02689
C	-1.73380	2.60783	-1.30064
C	-1.76611	2.53182	1.24103
C	0.29734	3.23149	0.01708
C	-2.77856	-2.82416	1.16995
C	-3.48445	0.02293	-0.12313

N	-4.52235	0.54209	-0.10640
C	2.36419	-0.81779	-0.30315
C	3.43304	-0.59824	-1.19189
C	4.63515	-0.07674	-0.74339
C	4.81797	0.24493	0.60118
C	3.76593	0.03515	1.48421
C	2.55125	-0.48482	1.04851
H	-3.08886	-2.55877	-0.93441
H	1.10492	-1.38686	-1.79779
H	0.17981	-1.67746	0.97639
H	-0.63115	-3.59255	-0.22737
H	-0.87185	-2.63760	-1.69483
H	-2.07462	3.64437	-1.28772
H	-1.10758	2.46122	-2.18222
H	-2.60962	1.97016	-1.38890
H	-2.11557	3.56487	1.27662
H	-1.15941	2.34145	2.12781
H	-2.63810	1.88385	1.27337
H	0.90092	3.03462	0.90559
H	0.92252	3.09192	-0.86762
H	-0.02089	4.27314	0.04586
H	-2.83976	-3.91316	1.11233
H	-2.05776	-2.56190	1.94814
H	-3.75546	-2.44600	1.47750
H	3.30801	-0.84467	-2.24006
H	5.43820	0.08160	-1.45287
H	5.75747	0.65401	0.94805
H	3.88094	0.28301	2.53238
H	1.75239	-0.61767	1.76424
N	-0.39299	0.93485	-0.05713
H	0.61276	0.89288	-0.09652

2’ Energy: -518493.7686204

C	-1.06466	2.55381	0.19731
N	1.34652	-0.23230	-0.42414
C	-1.85666	1.24279	0.12793
C	-1.02598	0.15176	0.15039
C	0.42118	0.63291	0.27783
C	0.35220	2.09573	-0.17336
C	-2.39229	-2.03121	0.05807
C	-1.87452	-3.47453	0.02275
C	-3.26814	-1.84212	1.30410
C	-3.17051	-1.74586	-1.23404
C	-1.59815	3.66870	-0.69632
C	-3.25546	1.29515	0.13952
N	-4.40831	1.42837	0.14556
C	2.72053	-0.22044	-0.17827
C	3.28725	0.34327	0.97269
C	4.66015	0.27101	1.18837
C	5.50032	-0.34633	0.26955
C	4.94245	-0.89718	-0.88317
C	3.57596	-0.83780	-1.10599
H	-1.06586	2.91519	1.23351
H	1.12073	-0.33795	-1.40419
H	0.65895	0.57960	1.34418
H	0.47259	2.12904	-1.26009
H	1.13159	2.71246	0.27299
H	-2.71354	-4.16751	-0.03197
H	-1.23927	-3.64070	-0.85006
H	-1.30052	-3.70562	0.92261
H	-4.12738	-2.51310	1.25546
H	-3.64325	-0.82541	1.38847
H	-2.69698	-2.07710	2.20368
H	-3.54967	-0.72766	-1.26709
H	-2.52901	-1.90546	-2.10228
H	-4.02334	-2.42271	-1.30574
H	-0.95880	4.55124	-0.62635
H	-1.62432	3.34599	-1.74000
H	-2.60846	3.96300	-0.40623
H	2.66867	0.84588	1.70242
H	5.07256	0.71389	2.08678
H	6.56735	-0.39364	0.44238
H	5.57728	-1.37879	-1.61686
H	3.15508	-1.27650	-2.00358
N	-1.18940	-1.17745	0.13941
H	-0.30822	-1.67239	0.11199

11’ Energy: -734953.8002451

C	1.19539	0.97186	-0.52209
C	0.77422	2.23892	-0.62255
C	-1.04439	0.23293	-1.48080
C	-0.47304	2.73736	-1.32772
C	-1.15456	1.61271	-2.12200
H	-1.39218	-0.52394	-2.18094
H	-0.13258	3.47684	-2.05732
H	-0.71960	1.55056	-3.12138
H	-2.21153	1.83780	-2.25976
C	1.57146	3.25476	-0.00927
N	2.17472	4.12568	0.44371
C	2.38832	0.52022	0.21261
C	3.44711	-0.10927	-0.44824
C	2.45517	0.72049	1.59306
C	4.55948	-0.52851	0.26669
H	3.40886	-0.26496	-1.51821
C	3.56990	0.29696	2.30417
H	1.62463	1.18902	2.10356
C	4.62154	-0.32870	1.64325
H	5.37885	-1.00933	-0.25095
H	3.61276	0.44998	3.37436
H	5.48867	-0.66156	2.19862
O	-1.25620	0.45788	0.90726
C	-1.78732	0.10679	-0.13954
N	-3.03367	-0.36039	-0.25940
C	-4.01713	-0.50592	0.84701

N	0.44239	-0.08667	-1.29069
H	0.82281	-0.02536	-2.23933
C	0.66770	-1.50803	-0.89380
C	0.52102	-1.93003	0.41856
C	1.00533	-2.38524	-1.91266
C	0.71695	-3.27496	0.70513
H	0.26634	-1.23215	1.19818
C	1.19659	-3.72825	-1.61142
H	1.12506	-2.03619	-2.93113
C	1.05202	-4.17315	-0.30286
H	0.60983	-3.61626	1.72577
H	1.46214	-4.41830	-2.40042
H	1.20452	-5.21794	-0.06736
C	-3.47851	-1.50119	1.88233
H	-2.55938	-1.13314	2.33383
H	-4.22006	-1.64556	2.66917
H	-3.27756	-2.46669	1.41537
C	-5.29824	-1.04968	0.21067
H	-5.68488	-0.36080	-0.54333
H	-5.12283	-2.02100	-0.25675
H	-6.06179	-1.17651	0.97744
C	-4.28601	0.86228	1.48430
H	-3.38249	1.26762	1.93540
H	-4.65262	1.56729	0.73655
H	-5.04442	0.75843	2.26130
C	-1.41620	3.46569	-0.35468
H	-0.90602	4.29915	0.12854
H	-2.26308	3.86817	-0.91077
H	-1.78682	2.79686	0.41848
H	-3.34178	-0.65368	-1.17343

11 Energy: -734700.1881453

C	0.97613	0.27791	-1.16836
C	1.18673	-0.88574	-2.15019
C	0.77585	-2.24012	-1.56383
C	-0.50818	-2.07832	-0.76715
C	-1.04722	-0.86446	-0.43579
H	1.17453	1.20762	-1.70196
H	2.22465	-0.91898	-2.48139
H	0.57728	-0.66766	-3.02881
H	0.56180	-2.90195	-2.40915
N	-0.41848	0.31226	-0.75084
C	-4.58799	-0.59708	1.94683
C	-4.63729	-1.23054	0.70939
C	-3.49232	-1.32028	-0.07387
C	-2.29173	-0.77114	0.37237
C	-2.24647	-0.13359	1.61469
C	-3.38864	-0.05211	2.39934
H	-5.47977	-0.52740	2.55660
H	-5.56756	-1.65245	0.35116
H	-3.53022	-1.80672	-1.03980
H	-1.31069	0.29027	1.95493
H	-3.34416	0.43680	3.36418
C	-2.34705	4.05973	-0.72002
C	-1.12269	3.89409	-0.08010
C	-0.49229	2.65552	-0.07719
C	-1.07965	1.57133	-0.72995
C	-2.29744	1.74245	-1.38947
C	-2.93147	2.97797	-1.37236
H	-2.83982	5.02326	-0.71339
H	-0.66060	4.72759	0.43345
H	0.44112	2.52157	0.45196
H	-2.74183	0.90764	-1.91321
H	-3.87840	3.09774	-1.88319
C	1.91457	0.25518	0.06577
O	1.47781	0.11044	1.20107
H	3.44771	0.56637	-1.20612
N	3.21397	0.42904	-0.23595
C	4.34354	0.47855	0.72286
C	4.43947	-0.84641	1.48939
H	3.53401	-1.02656	2.06566
H	4.58616	-1.67792	0.79847
H	5.28819	-0.81370	2.17441
C	5.60958	0.68763	-0.11230
H	5.75463	-0.13533	-0.81570
H	5.55977	1.62351	-0.67353
H	6.48006	0.73151	0.54202
C	4.15334	1.65504	1.68885
H	5.00351	1.71535	2.37035
H	4.08661	2.59426	1.13674
H	3.24491	1.52893	2.27487
C	-1.10691	-3.26968	-0.30238
N	-1.53318	-4.29055	0.04235
C	1.88460	-2.90630	-0.73583
H	1.57347	-3.89909	-0.40711
H	2.79031	-3.01682	-1.33535
H	2.12687	-2.32435	0.15219

9 Energy: -500455.4224518

O	0.65062	0.20233	0.00005
C	-0.41649	1.03584	0.00001
C	1.92815	0.82838	0.00006
H	2.07104	1.44151	-0.88933
H	2.07103	1.44142	0.88951
C	2.95695	-0.28143	-0.00000
F	2.85326	-1.07324	1.08399
F	4.19450	0.25106	0.00001
F	2.85324	-1.07314	-1.08407
C	-1.70715	0.30600	-0.00002
C	-1.77256	-1.09044	-0.00001
C	-2.88573	1.05689	-0.00002
C	-3.00756	-1.72475	0.00001
H	-0.86303	-1.67342	-0.00002
C	-4.11710	0.41841	-0.00002

H	-2.82262	2.13637	-0.00002
C	-4.17921	-0.97296	0.00001
H	-3.05638	-2.80578	0.00003
H	-5.02778	1.00291	-0.00003
H	-5.14045	-1.47089	0.00003
O	-0.29269	2.23892	0.00001

pent-2-enal Energy: -145170.2505778

C	-1.03954	0.39060	-0.00000
H	-0.96106	1.47583	0.00007
C	0.09291	-0.32419	-0.00012
H	0.08449	-1.40942	-0.00018
C	1.39258	0.33215	-0.00006
H	1.36026	1.43985	-0.00011
O	2.46283	-0.25093	0.00010
C	-2.41734	-0.16813	0.00005
H	-2.96916	0.18819	-0.87490
H	-2.41975	-1.25771	0.00000
H	-2.96908	0.18811	0.87509

aniline Energy: -180554.5638647

C	-1.87733	0.00000	0.00592
C	-1.16852	1.19886	0.00317
C	0.22021	1.20474	-0.00364
C	0.93801	0.00000	-0.00781
C	0.22021	-1.20474	-0.00364
C	-1.16852	-1.19886	0.00316
H	-2.95936	-0.00000	0.01222
H	-1.70061	2.14252	0.00854
H	0.75885	2.14541	-0.00622
H	0.75885	-2.14541	-0.00623
H	-1.70062	-2.14252	0.00854
N	2.32824	-0.00000	-0.07536
H	2.78040	0.83555	0.26387
H	2.78040	-0.83554	0.26392

benzoylacetonitrile
Energy: -299534.3510058

C	1.86976	-0.80638	-0.00004
H	1.67510	-1.42817	-0.87714
H	1.67517	-1.42829	0.87700
C	0.92084	0.40390	0.00006
C	3.26959	-0.41352	-0.00007
O	1.36805	1.53147	0.00017
N	4.37897	-0.11353	-0.00009
C	-0.53845	0.12487	0.00003
C	-1.06004	-1.17382	0.00008
C	-1.41841	1.21496	-0.00006
C	-2.43419	-1.37575	0.00005
H	-0.40926	-2.03686	0.00015
C	-2.78836	1.01059	-0.00009
H	-1.01009	2.21612	-0.00010
C	-3.29888	-0.28632	-0.00004
H	-2.82904	-2.38308	0.00009
H	-3.46079	1.85845	-0.00017
H	-4.36946	-0.44632	-0.00007

isocyanide Energy: -157368.4254093

C	0.26003	-0.00003	0.00006
C	0.73204	1.18097	0.85718
H	0.37223	1.08268	1.88152
H	1.82200	1.20061	0.87095
H	0.37169	2.12419	0.44620
C	0.73164	-1.33309	0.59394
H	0.37104	-2.17065	-0.00345
H	1.82159	-1.35545	0.60329
H	0.37174	-1.44894	1.61644
C	0.73145	0.15196	-1.45138
H	1.82138	0.15416	-1.47552
H	0.37088	-0.67544	-2.06276
H	0.37135	1.08833	-1.87800
C	-2.35081	0.00019	0.00022
N	-1.18857	0.00006	0.00017

water Energy: -47985.7491813

O	0.00000	0.00000	0.11773
H	0.00000	0.76334	-0.47093
H	0.00000	-0.76334	-0.47093

TFE Energy: -284251.5500010

C	-0.91054	0.74538	-0.00019
H	-0.92885	1.38013	0.89056
H	-0.92871	1.37997	-0.89107
C	0.41502	0.01533	0.00001
O	-1.93624	-0.22481	-0.00020
H	-2.78704	0.22816	0.00137
F	1.43097	0.90428	-0.00023
F	0.56828	-0.77152	1.08412
F	0.56827	-0.77210	-1.08369

acilium ion Energy: -216431.9139996

C	-1.92877	-0.00033	-0.00003
O	-3.05362	0.00012	-0.00018
C	-0.55060	-0.00026	0.00014
C	0.12921	1.24074	0.00022
C	1.50668	1.21892	-0.00002
C	2.18831	0.00022	-0.00020
C	1.50716	-1.21863	-0.00001
C	0.12960	-1.24090	0.00020
H	-0.42200	2.16971	0.00013
H	2.05591	2.14945	-0.00011
H	3.27013	0.00050	-0.00040
H	2.05654	-2.14907	-0.00004
H	-0.42116	-2.17013	0.00011

NH_3^+Ph/9/TFE Energy: -681289.3733125

O	1.31895	-1.22718	0.42919
H	0.55337	0.20500	1.61013
N	0.15056	1.10722	1.90738
H	0.93160	1.73695	2.10313
H	-0.34483	0.95629	2.78767
C	-0.74670	1.65278	0.86700
C	-0.18250	2.19264	-0.27707
C	-2.11461	1.56896	1.05273
C	-1.02898	2.66163	-1.27394
H	0.89159	2.24247	-0.39714
C	-2.94919	2.04197	0.04694
H	-2.52934	1.13432	1.95233
C	-2.40870	2.58522	-1.11284
H	-0.60620	3.08460	-2.17489
H	-4.02110	1.97661	0.17208
H	-3.06324	2.94837	-1.89376
C	0.52850	-1.54319	-0.65711
C	2.73610	-1.30397	0.26354
H	3.14947	-1.67741	1.19702
H	3.00249	-1.95824	-0.56300
C	3.31714	0.07381	-0.00061
F	2.88475	0.60687	-1.15451
F	4.65412	0.01067	-0.04074
F	2.97612	0.94920	0.98130
C	-0.90165	-1.60095	-0.28862
C	-1.31699	-1.91548	1.00864
C	-1.84757	-1.35881	-1.28751
C	-2.67239	-1.98361	1.30020
H	-0.58581	-2.12635	1.77706
C	-3.19930	-1.41048	-0.98465
H	-1.51266	-1.11627	-2.28656
C	-3.61239	-1.72546	0.30716
H	-2.99469	-2.23685	2.30136
H	-3.93141	-1.20189	-1.75318
H	-4.66837	-1.76753	0.54028
O	0.98552	-1.73051	-1.75193

NH_3^+Ph/TFE Energy: -465087.9696226

O	1.35565	-1.58661	-0.26891
H	1.98337	-2.06708	-0.82329
H	-0.33410	-2.01597	-0.23566
N	-1.37271	-2.11772	-0.23970
H	-1.63078	-2.72876	0.53613
H	-1.63409	-2.59979	-1.10299
C	-2.02830	-0.79993	-0.13588
C	-3.19643	-0.67808	0.59724
C	-1.44979	0.27456	-0.79167
C	-3.80547	0.56982	0.67439
H	-3.62664	-1.53313	1.10239
C	-2.06862	1.51608	-0.70594
H	-0.52964	0.15059	-1.34570
C	-3.24392	1.66355	0.02388
H	-4.71709	0.68302	1.24524
H	-1.62691	2.36639	-1.20761
H	-3.72072	2.63256	0.08879
C	2.03616	-0.85647	0.73651
H	2.82884	-1.43962	1.20663
H	1.30840	-0.56586	1.49113
C	2.65193	0.40900	0.16364
F	1.72581	1.22893	-0.36960
F	3.29855	1.09201	1.12608
F	3.54117	0.12831	-0.81191

D. References

[1] Gaussian 16, Revision C.01, Frisch, M. J.; Trucks, G. W.; Schlegel, H. B.; Scuseria, G. E.; Robb, M. A.; Cheeseman, J. R.; Scalmani, G.; Barone, V.; Petersson, G. A.; Nakatsuji, H.; Li, X.; Caricato, M.; Marenich, A. V.; Bloino, J.; Janesko, B. G.; Gomperts, R.; Mennucci, B.; Hratchian, H. P.; Ortiz, J. V.; Izmaylov, A. F.; Sonnenberg, J. L.; Williams-Young, D.; Ding, F.; Lipparini, F.; Egidi, F.; Goings, J.; Peng, B.; Petrone, A.; Henderson, T.; Ranasinghe, D.; Zakrzewski, V. G.; Gao, J.; Rega, N.; Zheng, G.; Liang, W.; Hada, M.; Ehara, M.; Toyota, K.; Fukuda, R.; Hasegawa, J.; Ishida, M.; Nakajima, T.; Honda, Y.; Kitao, O.; Nakai, H.; Vreven, T.; Throssell, K.; Montgomery, J. A., Jr.; Peralta, J. E.; Ogliaro, F.; Bearpark, M. J.; Heyd, J. J.; Brothers, E. N.; Kudin, K. N.; Staroverov, V. N.; Keith, T. A.; Kobayashi, R.; Normand, J.; Raghavachari, K.; Rendell, A. P.; Burant, J. C.; Iyengar, S. S.; Tomasi, J.; Cossi, M.; Millam, J. M.; Klene, M.; Adamo, C.; Cammi, R.; Ochterski, J. W.; Martin, R. L.; Morokuma, K.; Farkas, O.; Foresman, J. B.; Fox, D. J. Gaussian, Inc., Wallingford CT, 2016.

[2] C. Y. Legault, *CYLview. version 1.0b*, Université de Sherbrooke, Canada, 2008.

[3] J. Contreras-Garcia, E. R. Johnson, S. Keinan, R. Chaudret, J.-P. Piquemal, D. N. Beratan, W. T. Yang, *J. Chem. Theory Comput.*, 2011, **7**, 625-632.

[4] W. Humphrey, A. Dalke, K. Schulten, *J. Mol. Graphics*, 1996, **14**, 33-38.

[5] a) MacroModel, version 9.9, Schrödinger, LLC, New York, NY, 2012; b) N. G. J. Richards, W. C. Guida, R. Liskamp, M. Lipton, C. Caufield, G. Chang, T. Hendrickson, W. C. Still, *J. Comput. Chem.*, 1990, **11**, 440-467.

[6] S. Grimme, J. Antony, S. Ehrlich, H. Krieg, *J. Chem. Phys.*, **2010**, *132*, 154104.

[7] R. Echemendía, G. P. da Silva, M. Y. Kawamura, A. F. de la Torre, A. G. Corrêa, M. A. B. Ferreira, D. G. Rivera, M. W. Paixão, *Chem. Commun.* **2019**, *55*, 286–289.

[8] J. Crugeiras, A. Rios, E. Riveiros, J. P. Richard, *J. Am. Chem. Soc.* **2009**, *131*, 15815–15824.

[9] A. Bagno, G. Scorrano, *J. Am. Chem. Soc.* **1988**, *110*, 4577–4582.
