# Supplementary material for: Direct access to tetrasubstituted cyclopentenyl scaffolds through a diastereoselective isocyanide-based multicomponent reaction
Source: Chem Sci. 2021 Sep 16;12(48):15862–9. doi: 10.1039/d1sc04158d (PMC8672720; doi:10.1039/d1sc04158d)
Supplement: SC-012-D1SC04158D-s002 [file SC-012-D1SC04158D-s002.pdf]

Electronic Supplementary Material (ESI) for Chemical Science.
This journal is © The Royal Society of Chemistry 2021

Supporting Information

Direct Access to Tetrasubstituted Cyclopentenyl Scaffolds through Diastereoselective Isocyanide-based Multicomponent Reactions

Vitor A. Fernandes,[a] Rafaely N. Lima,[a] Yoisel B. Broterson,[a] Meire Y. Kawamura,[a] Radell Echemendía,[a,c] Alexander F. de la Torre,[b] Marco A. B. Ferreira,*[a] Daniel G. Rivera*[c] and Marcio W. Paixão*[a]

a.Centre of Excellence for Research in Sustainable Chemistry (CERSusChem), Department of Chemistry, Federal University of São Carlos – UFSCar, Rodovia Washington Luís, km 235 - SP-310 - São Carlos - São Paulo - Brazil -13565-905. E-mail: mpwpaixao@ufscar.br, marco.ferreira@ufscar.br

b.Facultad de Ciencias Químicas, Universidad de Concepción, Chile.

c.Center for Natural Products Research, Faculty of Chemistry, University of Havana, Zapata y G, 10400, Havana, Cuba. E-mail: dgr@fq.uh.cu

Content

General information S2

General procedure for the synthesis of hemiacetals 1a-h S3

General multicomponent reaction procedure for the synthesis of cyclopentenes 2a-2z/2aa-2as S10

General reaction procedure for the synthesis of cyclopentenols 3a-d S39

Continuous-flow one-pot synthesis of (3R,5R)-3-(tert-butylamino)-2-(cyclohexylamino)-5-ethylcyclopent-1-ene-1-carbonitrile (2d) S41

Synthesis of the bulky tertiary amine 4 .... S43

Synthesis of methyl ((3R,5R)-2-cyano-3-ethyl-5-(tritylamino)cyclopent-1-en-1-yl)glycinate (5) S44

Procedure for ester deprotection of compound 5 .... S44

Procedure for the trityl deprotection of compound 5 .... S45

One-pot procedure for the synthesis of cis-cyclopentenyl amine 8 .... S46

GC-MS chromatograms and mass fragmentation analyses S47

NMR Spectra S49

UPC^2 and HPLC chromatograms S114

General information

Reagents and materials were purchased from the highest commercially available grade and used without further purification. Flash column chromatography was carried out using silica gel 60 (230-400 mesh) and reactions were monitored using analytical thin layer chromatography (TLC). NMR spectra were recorded at 400 MHz for ^{1}H and 100 MHz for ^{13}C, unless otherwise noted. Chemical shifts (δ =) are reported in parts per million relatives to the tetramethylsilane (TMS) and coupling constants (*J*) are reported in hertz (Hz). All signals are reported in ppm with the internal reference of residual solvent signals (7.26 or 77.1 ppm for chloroform; 3.31 or 49.0 ppm for methanol). Data are presented as follows: multiplicity (s = singlet, d = doublet, t = triplet, q = quartet, quint = quintet, sext = sextet, m = multiplet, br = broad, dd = doublet of doublet, dt = doublet of triplet), coupling constant (J/Hz) and integration. The diastereoisomeric ratio (dr) was determined by integration of the ^{1}H NMR spectra of the crude reaction mixture. Only the characterization data for the major isomer is given.

The enantiomeric excess (ee) of products was determined by High Performance Liquid Chromatography (HPLC) or Ultraperformance Convergence Chromatography (UPC^2) analysis. HPLC chromatograms were obtained on an apparatus with a LC-10AT Pump, SPD-10AUV-Vis Detector, SCL-10A System Controller, using a Chiralpak AS-H column (4.6 mmØ × 250 mmL, particle size 5 μm). UPC^2 chromatograms were recorded by Waters ACQUITY UPC^2 using Trefoil CEL2 (2.5 μm, 3 x 15 mm), AMY1 (2.5 μm, 3 x 15 mm) and CEL1 (2.5 μm, 3 x 15 mm) columns as chiral stationary phases with PDA detection. Optical rotations were measured on a PerkinElmer 241 polarimeter at 589 nm and 20 °C, $[\alpha]_D$ values are given in deg·cm^3·g^{-1}·dm^{-1}; concentration c in g. (100 mL)$^{-1}$. High resolution mass spectra (HRMS) were performed on a Waters Acquity UPLC H-Class System Xevo G2-XS Q-TOF Spectrometer (ESI-Q-TOF). Melting points were determined using a Büchi M-560 Basic Melting Point Apparatus. GC-MS analysis were performed using a Shimadzu GCMS-QP2010S Gas Chromatograph coupled to a MS detector equipped with an Zebron ZB5-MS capillary column (30 m x 0.32 mm, 0.25 μm) under the operation parameters: temperature of inlet of 250 °C, temperature of the interface of 300 °C, temperature ramp of the oven from 50 to 250 °C at a rate of 10 °C min^{-1}.

General procedure for the synthesis of hemiacetals 1a-h

To a solution of Jørgensen catalyst (0.1 equiv.) and 3,5-dinitrobenzoic acid (0.2 equiv.) in toluene (0.1 M) at –20 °C was added the α,β-unsaturated aldehyde under magnetic stirring. After 20 min, benzoyl acetonitrile was introduced into the reaction mixture. The resulting solution was stirred for 48h at the same temperature. The volatiles were concentrated under reduced pressure and the resulting crude product was purified by flash column chromatography.

(4*R*)-Ethyl-2-hydroxy-6-phenyl-3,4-dihydro-2*H*-pyran-5-carbonitrile (1a)

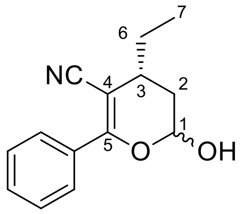

Prepared according general procedure using benzoylacetonitrile (888 mg, 6.1 mmol) and pent-2-enal (390 µL, 4.0 mmol). Obtained after flash column chromatography purification (*n*-hexane/EtOAc 9:1) as a yellow solid (595 mg, 2.6 mmol, 65% yield, 94% ee). m.p. 66-68 °C. $[\alpha]_D^{20}$ + 4.0 (*c* 0.5, acetone, 20°C). R_f = 0.23 (*n*-hexane/ EtOAc 4:1).

^{1}H NMR (400 MHz, $CDCl_3$) δ = 7.75 – 7.67 (m, 2H, Ph), 7.46 – 7.37 (m, 3H, Ph), 5.66 (t, *J* = 3.5 Hz, H-1_{major}), 5.47 (d, *J*=8.7, 1H, H-1_{minor}), 3.65 (brs, 1H, OH), 2.68 – 2.53 (m, 1H), 2.11 – 1.92 (m, 2H), 1.76 – 1.46 (m, 2H), 1.02 (t, *J* = 7.4 Hz, 3H, H-7).

^{13}C NMR (100 MHz, $CDCl_3$) δ = 163.6 (C-5_{minor}), 162.5 (C-5_{major}), 133.5, 130.9 (minor), 130.7 (major), 128.5(minor), 128.4 (major), 128.3 (minor), 128.2 (major), 119.6 (CN), 96.2 (C-1_{minor}), 92.8 (C-1_{major}), 89.3 (C-4_{major}), 88.8 (C-4_{minor}), 34.4 (C-2_{minor}), 33.1 (C-6_{minor}), 30.8 (C-2_{major}), 30.3 (C-6_{major}), 26.6 (C-3), 10.8 (C-7_{major}), 10.6 (C-7_{minor}).

HRMS (ESI-Q-TOF) *m/z*: 230.1182 $[M+H]^+$; calcd. for $C_{14}H_{16}NO_2$: 230.1176.

The enantiomeric ratio was determined by UPC^2 analysis using Trefoil CEL2, gradient CO_2/iPrOH = 100:0 to 90:10 until 5 min then isocratic 90:10, flow rate = 2 mL/min, 25 °C, λ = 264 nm, t_R = 4.79 min (minor) and t_R = 5.19 min (major), 94% ee.

(4*R*)-2-hydroxy-4-methyl-6-phenyl-3,4-dihydro-2*H*-pyran-5-carbonitrile (1b)

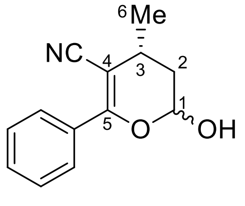

Prepared according general procedure using benzoylacetonitrile (145 mg, 1 mmol) and (*E*)-but-2-enal (165 µL, 2 mmol). Obtained after flash column chromatography purification (*n*-hexane/EtOAc 4:1) as a brown oil (193 mg, 0.9 mmol, 90% yield, 2:1 dr, 98%

ee). $[\alpha]_D^{20}$ + 5.8 (*c* 0.6, acetone, 20°C). R_f= 0.36 (*n*-hexane/ EtOAc 7:3).

^{1}H NMR (400 MHz, $CDCl_3$) δ = 7.73 – 7.64 (m, 2H, Ph), 7.45 – 7.30 (m, 3H, Ph), 5.56 (t, *J* = 2.5 Hz, 1H, H-1_{major}), 5.39 (d, *J* = 8.6 Hz, 1H, H-1_{minor}), 4.58 (brs, 1H, OH), 2.82 – 2.68 (m, $1H_{major}$), 2.71 – 2.57 (m, $1H_{minor}$), 2.18 (ddd, *J* = 13.5, 6.4, 1.7 Hz, $1H_{minor}$), 2.02 (dt, *J* = 13.6, 5.0 Hz, $1H_{major}$), 1.67 – 1.56 (m, 1H), 1.33 – 1.22 (m, 3H, H-6).

^{13}C NMR (100 MHz, $CDCl_3$) δ = 163.5 (C-5_{minor}), 162.6 (C-5_{major}), 133.4 (Ph_{major}), 133.0 (Ph_{minor}), 130.8 (Ph_{minor}), 130.6 (Ph_{minor}), 128.3 (Ph_{minor}), 128.3 (Ph_{major}), 128.1 (Ph_{major}), 128.1 (Ph_{major}), 119.7 (CN), 95.8 (C-1_{minor}), 92.5 (C-1_{major}), 89.4 (C-5_{major}), 88.8 (C-4_{minor}), 36.2 (C-2_{minor}), 34.3 (C-2_{major}), 28.3 (C-6_{minor}), 24.2 (C-6_{major}), 19.8 (C-3).

HRMS (ESI-Q-TOF) *m/z*: 216.1025 $[M+H]^+$; calcd. for $C_{13}H_{14}NO_2$: 216.1019.

The enantiomeric ratio was determined by UPC^2 analysis using Trefoil CEL2, gradient CO_2/iPrOH = 100:0 to 60:40 in 5 min, flow rate = 1 mL/min, 35 °C, λ = 248 nm, t_R = 4.23 min (minor) and t_R = 4.52 min (major), 98% ee.

(4*R*)-4-heptyl-2-hydroxy-6-phenyl-3,4-dihydro-2*H*-pyran-5-carbonitrile (1c)

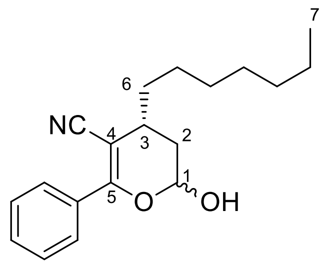

Prepared according general procedure using benzoylacetronitrile (87 mg, 0.6 mmol) and (*E*)-dec-2-enal (183 µL, 1 mmol). Obtained after flash column chromatography purification (*n*-hexane/EtOAc 4:1) as a brown oil (126 mg, 0.42 mmol, 70% yield, 2:1 dr, > 99% ee). $[\alpha]_D^{20}$ + 1.6 (*c* 0.5, acetone, 20°C). R_f= 0.53 (*n*-hexane/ EtOAc 7:2).

^{1}H NMR (400 MHz, $CDCl_3$) δ = 7.74 – 7.66 (m, 2H), 7.45 – 7.33 (m, 3H), 5.61 (s, 1H, H-1_{major}), 5.42 (t, *J* = 7.2 Hz, 1H, H-1_{minor}), 3.88 (brs, 1H, OH), 2.69 – 2.49 (m, 1H), 2.23 (dd, *J* = 13.4, 6.3 Hz, 1H, H-1_{minor}), 2.04 (dt, *J* = 13.7, 5.2 Hz, 1H, H-1_{major}), 1.97 – 1.83 (m, 1H), 1.78 – 1.58 (m, 1H), 1.51 – 1.17 (m, 11H), 0.89 (t, *J* = 6.6 Hz, 3H, H-7).

^{13}C NMR (100 MHz, $CDCl_3$) δ = 163.6 (C-5_{minor}), 162.5 (C-5_{major}) , 133.6 (Ph_{major}), 133.1 (Ph_{minor}), 130.8 (Ph_{minor}), 130.7 (Ph_{minor}), 128.4 (Ph_{major}), 128.3 (Ph_{major}), 128.2 (Ph_{major}), 119.7 (CN), 96.2 (C-1_{minor}), 92.8 (C-1_{major}), 89.4 (C-4_{major}), 88.9 (C-4_{minor}), 33.9 (major), 33.9 (minor), 33.7 (minor), 33.2 (minor), 31.9 (major), 31.5 (major), 29.7

(major), 29.6 (minor), 29.3 (major), 29.2 (major), 26.5 (major), 26.4 (minor), 22.8 (major), 14.2 (C-7).

HRMS (ESI-Q-TOF) *m/z*: 300.1971 $[M+H]^+$; calcd. for $C_{19}H_{26}NO_2$: 300.1958.

The enantiomeric ratio was determined by UPC^2 analysis using Trefoil CEL2, gradient CO_2/iPrOH = 100:0 to 80:20 until 2 min then isocratic 80:20, flow rate = 0.8 mL/min, 25 °C, λ = 264 nm, t_R = 4.01 min (minor) and t_R = 4.25 min (major), > 99% ee.

(4*R*)-4-((*Z*)-hex-3-en-1-yl)-2-hydroxy-6-phenyl-3,4-dihydro-2*H*-pyran-5-carbonitrile (1d)

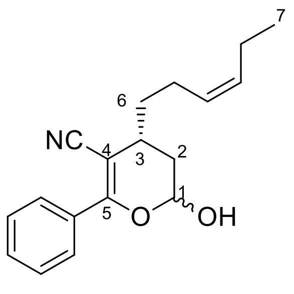

Prepared according general procedure using benzoylacetonitrile (44 mg, 0.3 mmol) and (2*E*,6*Z*)-nona-2,6-dienal (96 µL, 0.6 mmol). Obtained after column chromatography purification (*n*-hexane/EtOAc 4:1) as a brown oil (59 mg, 0.21 mmol, 70% yield, 3:1 dr, 98% ee). $[\alpha]_D^{20}$ + 4.2 (*c* 0.5, acetone, 20°C). R_f = 0.61 (*n*-hexane/ EtOAc 7:3).

^{1}H NMR (400 MHz, $CDCl_3$) δ = 7.81 – 7.63 (m, 2H), 7.52 – 7.38 (m, 3H), 5.65 (t, *J* = 3.4 Hz, 1H, H-1), 5.56 – 5.27 (m, 2H), 3.54 (brs, 1H, OH), 2.79 – 2.56 (m, 1H), 2.26 – 1.94 (m, 6H), 1.84 – 1.41 (m, 3H), 0.98 (td, *J* = 7.5, 1.1 Hz, 3H, H-7).

^{13}C NMR (100 MHz, $CDCl_3$) δ = 162.4 (C-5), 133.5 (minor), 132.9 (major), 130.9 (Ph_{major}), 130.7 (major), 128.4 (Ph_{major}), 128.3 (Ph_{major}), 128.2 (Ph_{major}), 127.7 (Ph_{minor}), 127.7 (Ph_{minor}), 119.6 (CN_{minor}), 119.5 (CN_{major}), 96.0 (C-1_{minor}), 92.7 (C-1_{major}), 89.4 (C-4_{major}), 89.0 (C-4_{minor}), 33.8 (major), 33.8 (minor), 33.5 (minor), 32.7 (minor), 31.4 (major), 28.8 (major), 24.1 (major), 24.0 (minor), 20.8 (major), 14.4 (C-7).

HRMS (ESI-Q-TOF) *m/z*: 284.3326 $[M+H]^+$; calcd. for $C_{18}H_{22}NO_2$: 284.3345.

The enantiomeric ratio was determined by UPC^2 analysis using Trefoil CEL2, gradient CO_2/iPrOH = 100:0 to 80:20 until 2 min then isocratic 80:20, flow rate = 1 mL/min, 25 °C, λ = 220 nm, t_R = 4.59 min (minor) and t_R = 4.31 min (major), 98% ee.

(4*R*)-4-((1,3-dioxoisoindolin-2-yl)methyl)-2-hydroxy-6-phenyl-3,4-dihydro-2*H*-pyran-5-carbonitrile (1e)

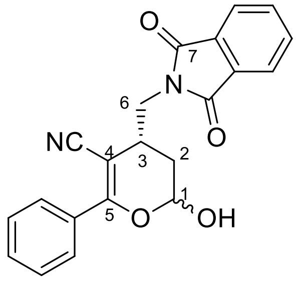

Prepared according general procedure using benzoylacetonitrile (43.5 mg, 0.3 mmol) and (*E*)-4-(1,3-dioxoisoindolin-2-yl)but-2-enal (77.4 mg, 0.36 mmol).

Obtained after flash column chromatography purification (*n*-hexane/EtOAc 4:1) as a brown oil (101 mg, 0.28 mmol, 93% yield, 2:1 dr, > 99% ee). $[\alpha]_D^{20}$ + 7.4 (*c* 0.5, acetone, 20°C). R_f = 0.36 (*n*-hexane/ EtOAc 7:3).

^{1}H NMR (400 MHz, $CDCl_3$) δ = 7.99 – 7.83 (m, 2H, Ph), 7.81 – 7.66 (m, 4H, Ph), 7.54 – 7.33 (m, 3H, Ph), 5.70 (brs, 1H, H-1_{major}), 5.65 (brs, 1H, H-1_{minor}), 4.33 (dd, *J*=13.6, 7.2 Hz, 1H, H-$6a_{minor}$), 4.14 (dd, *J* = 13.7, 5.8 Hz, 1H, H-$6a_{major}$), 3.92 (dd, *J* = 13.8, 7.9 Hz, 1H, H-$6b_{minor}$), 3.81 (dd, *J* = 13.7, 9.5, 1H, H-6_{major}), 3.31 – 3.10 (m, 1H), 2.22 – 1.79 (m, 2H), 1.71 (brs, 1H, OH).

^{13}C NMR (100 MHz, $CDCl_3$) δ = 168.7 (C-7_{minor}), 168.4 (C-7_{major}), 164.1 (C-5 $_{major}$), 164.0 (C-5_{minor}), 134.3 (Ph_{major}), 134.2 (Ph_{minor}), 132.9 (Ph_{minor}), 132.8 (Ph_{minor}), 131.9 (Ph_{minor}), 131.8 (Ph_{minor}), 130.9 (Ph_{major}), 128.4 (Ph_{major}), 128.2 (Ph_{major}), 128.2 (Ph_{major}), 123.6 (Ph_{major}), 119.4 (CN_{minor}), 119.0 (CN_{major}), 94.1 (C-1_{minor}), 92.6 (C-1_{major}), 84.8 (C-4_{minor}), 84.8 (C-4_{major}), 41.6 (C-6_{minor}), 41.2 (C-6_{major}), 31.2 (C-2_{minor}), 30.1 (C-2_{major}), 29.8 (C-3).

HRMS (ESI-Q-TOF) *m/z*: 361.1181 $[M+H]^+$; calcd. for $C_{21}H_{17}N_2O_4$: 361.1183.

The enantiomeric ratio was determined by UPC^2 analysis using Trefoil AMY1, isocratic CO_2/MeOH = 65:35, flow rate = 1 mL/min, 35 °C, λ = 267 nm, t_R = 1.71 min (major) and t_R = 2.15 min (minor), > 99% ee.

(4*R*)-4-((benzyloxy)methyl)-2-hydroxy-6-phenyl-3,4-dihydro-2*H*-pyran-5-carbonitrile (1f)

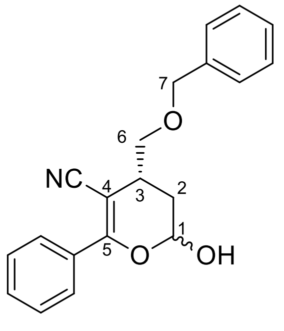

Prepared according the general procedure using benzoylacetonitrile (290 mg, 2.0 mmol) and (*E*)-4-(benzyloxy)but-2-enal (532 mg, 2.0 mmol). Obtained after flash column chromatography purification (*n*-hexane/EtOAc 9:1) as an orange oil (386 mg, 1.20 mmol, 60% yield, 3:1 dr, > 99% ee). $[\alpha]_D^{20}$ + 1.1 (*c* 0.5, acetone, 20°C). R_f = 0.10 (*n*-hexane/ EtOAc 4:1).

^{1}H NMR (400 MHz, $CDCl_3$) δ = 7.80 – 7.69 (m, 2H), 7.48 – 7.27 (m, 8H), 6.89 (d, *J* = 11.2, 1H, OH), 5.66 (s, 1H, H-1a), 5.58 (d, *J* = 11.2 Hz, 1H, H-1b), 4.74 – 4.59 (m, 2H, H-7b), 4.59 (s, 2H, H-7a), 3.98 (dd, *J* = 9.7, 3.2 Hz, 1H, H-6b), 3.77 (dd, *J* = 9.7, 3.4 Hz, 1H, H-6a), 3.69 (dd, *J* = 9.5, 6.5 Hz, 1H, H-6a), 3.53 (dd, *J* = 9.8, 2.3 Hz, 1H, H-

6b), 2.92 (qd, J = 6.9, 3.4 Hz, 1H, H-3b), 2.82 (d, J = 8.5 Hz, 1H, H-3a), 2.44 (ddd, J = 13.2, 9.1, 3.5 Hz, 1H, H-2), 2.11 – 2.02 (m, 1H, H-2).

^{13}C NMR (100 MHz, CDCl3) δ = 164.7 (C-5b), 163.8 (C-5a), 138.1 (Ph-a), 136.2 (Ph-b), 133.6 (Ph-a), 133.6 (Ph-b), 133.4 (Ph-a), 130.9 (Ph-b), 130.8 (Ph-a), 128.8 (Ph-b), 128.6 (Ph-a), 128.4 (Ph-b), 128.2 (Ph-b), 127.9 (Ph-a), 127.9 (Ph-a), 127.8 (Ph-b), 119.6 (CN-b), 119.3 (CN-a), 93.2 (C-1a), 91.6 (C-1b), 85.4 (C-4a), 84.1 (C-4b), 74.2 (C-6b), 73.6 (C-6a), 71.3 (C-7a), 71.3 (C-7b), 31.9 (C-3b), 31.5 (C-3b), 31.1 (C-2a), 30.1 (C-3a).

HRMS (ESI-Q-TOF) *m/z*: 344.1443 [M+H]$^+$; calcd. for $C_{20}H_{19}NO_3Na$: 344.1263.

The enantiomeric ratio was determined by UPC^2 analysis using Trefoil CEL2, gradient CO_2/MeOH = 100:0 to 60:40 in 6 min, flow rate = 1 mL/min, 35 °C, λ = 292 nm, t_R = 5.27 min (major) and t_R = 5.40 min (minor), > 99% ee.

(4*R*)-4-(((*tert*-butyldimethylsilyl)oxy)methyl)-2-hydroxy-6-phenyl-3,4-dihydro-2*H*-pyran-5-carbonitrile (1g)

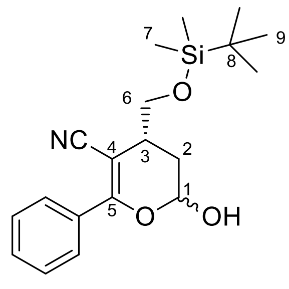

Prepared according the general procedure using benzoylacetonitrile (290 mg, 2.0 mmol) and *(E)*-4-((tert-butyldimethylsilyl)oxy)but-2-enal (400 mg, 2.0 mmol). Obtained after flash column chromatography purification (*n*-hexane/EtOAc 4:1) as a yellow oil (132 mg, 0.38 mmol, 19% yield, 9:1 dr, > 99% ee). $[\alpha]_D^{20}$ + 2.6 (*c* 0.5, acetone, 20°C). R_f = 0.33 (*n*-hexane/ EtOAc 4:1).

^{1}H NMR (400 MHz, $CDCl_3$) δ = 7.77 – 7.67 (m, 2H, Ph), 7.49 – 7.36 (m, 3H, Ph), 7.30 – 7.23 (m, 1H), 5.72 (brs, 1H, H-1a), 5.59 (d, J = 11.7 Hz, 1H, H-1b), 4.18 (dd, J=10.7, 3.0, 1H, H-6b), 3.92 – 3.80 (m, 1H, H-6a), 3.67 (dd, J = 10.6, 1.9 Hz, 1H, H-6b), 3.56 (brs, 1H, H-6a), 2.82 – 2.72 (m, 1H, H-2), 2.47 (dddd, J = 14.0, 9.1, 3.4, 1.2 Hz, 1H, H-3), 2.18 – 1.92 (m, 1H, H-2), 0.90 (d, J = 3.5 Hz, 9H, H-9), 0.16 (d, J = 5.6 Hz, 3H, H-7), 0.10 (d, J = 3.8 Hz, 3H, H-7).

^{13}C NMR (100 MHz, CDCl3) δ = 165.0 (C-5b), 164.0 (C-5a), 133.7 (Ph-b), 130.8 (Ph-b), 130.6 (Ph-a), 128.4 (Ph-b), 128.3 (Ph-a), 128.2 (Ph-b), 128.1 (Ph-a), 119.7 (CN), 93.4 (C-1a), 91.5 (C-1b), 85.2 (C-4a), 83.8 (C-4b), 65.4 (C-6b), 64.3 (C-6a), 33.6 (C-2b), 33.2 (C-2a), 31.7 (C-8b), 30.0 (C-8a), 25.9 (C-9a), 25.7 (C-9b), 18.3 (C-3), -5.4 (C-7a), -5.6 (C-7b), -5.6 (C-7b).

HRMS (ESI-Q-TOF) *m/z*: 346.1842 $[M+H]^+$; calcd. for $C_{19}H_{28}NO_3Si$: 346.1833.

The enantiomeric ratio was determined by UPC^2 analysis using Trefoil CEL2, gradient CO_2/MeOH = 100:0 to 60:40 in 6 min, flow rate = 1 mL/min, 35 °C, λ = 280 nm, t_R = 3.29 min (major) and t_R = 3.94 min (minor), > 99% ee.

(4*R*)-2-hydroxy-6-phenyl-4-((prop-2-yn-1-yloxy)methyl)-3,4-dihydro-2*H*-pyran-5-carbonitrile (1h)

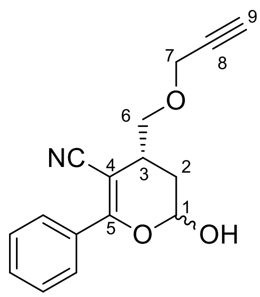

Prepared according the general procedure using benzoylacetonitrile (145 mg, 1.0 mmol) and *(E)*-4-(prop-2-yn-1-yloxy)but-2-enal (149 mg, 1.2 mmol). Obtained after flash column chromatography purification (*n*-hexane/EtOAc 9:1) as a yellow oil (128 mg, 0.48 mmol, 48% yield, 1:1 dr, > 99% ee). $[\alpha]_D^{20}$ + 2.2 (*c* 0.5, acetone, 20°C). R_f = 0.08 (*n*-hexane/ EtOAc 4:1).

^{1}H NMR (400 MHz, $CDCl_3$) δ = 7.79 – 7.67 (m, 2H, Ph), 7.48 – 7.35 (m, 3H, Ph), 6.20 (s, 1H, OH), 5.69 (t, *J* = 3.8 Hz, 1H, H-1a), 5.59 (brs, 1H, H-1b), 4.37 – 4.18 (m, 2H), 4.00 (dd, *J* = 9.6, 3.7 Hz, 1H, H-6a), 3.83 (dd, *J* = 9.4, 3.4 Hz, 1H, H-6b), 3.76 – 3.66 (m, 1H), 2.96 – 2.83 (m, 1H), 2.50 (dd, *J* = 23.0, 2.1 Hz, 1H), 2.42 (dd, *J* = 9.5, 3.8 Hz, 1H, H-2a), 2.12 (dt, *J* = 14.8, 2.6 Hz, 1H, H-2b), 2.05 (dd, *J* = 7.4, 3.7 Hz, 1H).

^{13}C NMR (100 MHz, CDCl3) δ = 164.7 (C-5a), 163.9 (C-5b), 133.5 (Ph-a), 133.3 (Ph-b), 131.0 (Ph-a), 130.8 (Ph-b), 128.4, 128.2, 119.4 (CN-a), 119.2 (CN-b), 93.2 (C-1a), 91.9 (C-1b), 85.2 (C-4a), 84.0 (C-4b), 79.4 (C-8a), 77.9 (C-8b), 76.3 (C-9a), 75.0 (C-9b), 71.1 (C-6a), 70.9 (C-6b), 59.0 (C-7a), 58.7 (C-7b), 31.8 (C-2a), 31.3 (C-3a), 30.8 (C-2b), 30.0 (C-3b).

HRMS (ESI-Q-TOF) *m/z*: 268.0975 $[M-H]^-$; calcd. for $C_{16}H_{14}NO_3$: 268.0979.

The enantiomeric ratio was determined by UPC^2 analysis using Trefoil CEL2, gradient CO_2/MeCN = 100:0 to 60:40 in 6 min, flow rate = 1 mL/min, 35 °C, λ = 300 nm, t_R = 3.64 min (minor) and t_R = 3.74 min (major), > 99% ee.

(4*S*)-2-hydroxy-4,6-diphenyl-3,4-dihydro-2*H*-pyran-5-carbonitrile (1i)

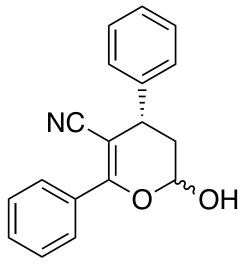

Prepared according procedure of the respective literature.[1] All spectroscopic data are in accordance with the same reference.

The enantiomeric ratio was determined by UPC^2 analysis using Trefoil CEL2, Gradient CO_2/iPrOH = 100:0 to 80:20 until 2 min then isocratic 80:20, flow rate = 1 mL/min, 25 °C, λ = 220 nm, t_R = 4.13 min (minor) and t_R = 4.50 min (major), 98% ee.

[1] Z. Niu, X. He, Y. Shang, *Tetrahedron: Asymmetry* **2014**, *25*, 796–801.

General multicomponent reaction procedure for the synthesis of cyclopentenes 2a-2z/2aa-2as

The hemiacetal **1a-1h** (0.15 mmol, 1 equiv.) was dissolved in trifluoroethanol (0.3 mL), and the amine (0.15 mmol, 1.0 equiv.) was added to this mixture. Triethylamine (0.16 mmol, 1.1 equiv.) was also added to the reaction when α-amino acid and peptide methyl ester hydrochlorides were employed as amino components. After 10 minutes, the isocyanide (0.15 mmol, 1.0 equiv.) was introduced into this solution and the glass tube was sealed. The flask was irradiated for 20 min (300 W) under high-speed magnetic stirring in the microwave reactor, while the temperature was raised up to 70 °C. The volatiles was concentrated under reduced pressure and the resulting crude product was purified by flash column chromatography.

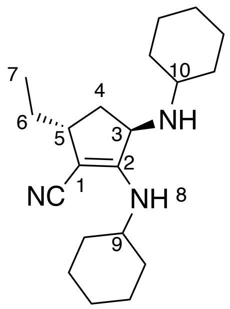

(3*R*,5*R*)-2,3-bis(cyclohexylamino)-5-ethylcyclopent-1-ene-1-carbonitrile (2a)

Prepared according the general procedure using hemiacetal **1a** (34 mg, 0.15 mmol), cyclohexylamine (17 µL, 0.15 mmol) and cyclohexyl isocyanide (19 µL, 0.15 mmol). Obtained after flash column chromatography purification (*n*-hexane/EtOAc 5:1) as a pale yellow oil (44 mg, 0.14 mmol, 93% yield, 79:21 dr, 76% ee). $[\alpha]_D^{20}$ – 12.4 (*c* 0.5, acetone, 20°C). R_f= 0.36 (*n*-hexane/ EtOAc 6:1).

^{1}H NMR (400 MHz, $CDCl_3$) δ = 5.11 (d, *J* = 8.0 Hz, 1H, H-8), 3.84-3.71 (m, 1H, H-9), 3.66 (dd, *J* = 9.0/ 7.6 Hz, 1H, H-3), 2.64 (td, *J* = 8.2/5.6 Hz, 1H, H-5), 2.54-2.43 (m, 1H, H-10), 2.09-1.99 (m, 4H), 1.76-1.66 (m, 4H), 1.65-1.32 (m, 8H), 1.28-1.06 (m, 8H), 0.92 (t, J = 7.4 Hz, 3H, H-7).

^{13}C NMR (100 MHz, $CDCl_3$) δ = 159.3 (C), 121.4, 71.5, 60.3 54.9, 51.3, 43.5, 37.6 (CH_2), 35.2, 33.6, 33.4, 28.0, 25.9, 25.6, 25.0, 24.8, 24.3, 24.2, 11.8 (CH_3).

HRMS (ESI-Q-TOF) *m/z*: 316.2755 $[M+H]^+$; calcd. for $C_{20}H_{34}N_3$: 316.2757.

The enantiomeric ratio was determined by UPC^2 analysis using Trefoil CEL2, isocratic gradient CO_2/iPrOH = 90:10, flow rate = 1 mL/min, 35 °C, λ = 269 nm, t_R = 3.64 min (minor) and t_R = 3.64 min (major), 76% ee.

(3*R*,5*R*)-2-(cyclohexylamino)-3-(cyclopropylamino)-5-ethylcyclopent-1-ene-1-carbonitrile (2b)

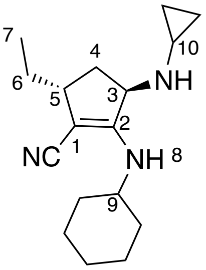

Prepared according the general procedure using hemiacetal **1a** (34 mg, 0.15 mmol), cyclopropylamine (10 μL, 0.15 mmol) and cyclohexyl isocyanide (19 μL, 0.15 mmol). Obtained after flash column chromatography purification (*n*-hexane/EtOAc 9:1) as a brown oil (16 mg, 0.06 mmol, 40% yield, 80:20 dr, > 99% ee). $[\alpha]_D^{20}$ – 5.6 (*c* 0.5, acetone, 20°C). R_f = 0.37 (*n*-hexane/ EtOAc 4:1).

^{1}H NMR (400 MHz, $CDCl_3$) δ = 4.92 (d, *J* = 8.3 Hz, 1H, H-8), 3.80 – 3.70 (m, 2H), 2.75 – 2.67 (m, 1H), 2.25 – 2.18 (m, 1H), 2.12 – 1.97 (m, 3H), 1.76 – 1.58 (m, 5H), 1.48 – 1.08 (m, 7H), 0.93 (t, *J* = 7.4 Hz, 3H, H-7), 0.57 – 0.43 (m, 3H), 0.35 – 0.27 (m, 1H).

^{13}C NMR (100 MHz, CDCl3) δ = 157.5 (C-2), 121.2 (CN), 73.2 (C-1), 63.0, 51.7, 44.0, 35.9, 33.7, 33.7, 28.2, 28.1, 25.7, 24.5, 24.4, 11.8 (C-7), 6.8, 6.5.

HRMS (ESI-Q-TOF) *m/z*: 274.2278 $[M+H]^+$; calcd. for $C_{17}H_{27}N_3$: 274.2288.

The enantiomeric ratio was determined by UPC^2 analysis using Trefoil CEL2, gradient CO_2/MeOH = 100:0 to 60:40 in 6 min, flow rate = 1 mL/min, 35 °C, λ = 245 nm, t_R = 3.55 min (minor) and t_R = 3.79 min (major), > 99% ee.

(3*R*,5*R*)-2-(cyclohexylamino)-5-ethyl-3-(isopropylamino)cyclopent-1-ene-1-carbonitrile (2c)

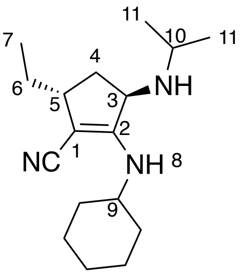

Prepared according the general procedure using hemiacetal **1a** (34 mg, 0.15 mmol), isopropylamine (12 μL, 0.15 mmol) and cyclohexyl isocyanide (19 μL, 0.15 mmol). Obtained after flash column chromatography purification (*n*-hexane/EtOAc 4:1) as a pale yellow oil (32 mg, 0.12 mmol, 78% yield, 99:1 dr, 93% ee). $[\alpha]_D^{20}$ – 8.1 (*c* 0.5, acetone, 20°C). R_f = 0.36 (*n*-hexane/ EtOAc 7:3).

^{1}H NMR (400 MHz, $CDCl_3$) δ = 5.10 (d, *J* = 8.7 Hz, 1H), 381 – 3.71 (m, 1H), 3.63 (dd, *J* = 9.3, 7.2 Hz, 1H), 2.88 (hept, *J* = 6.2 Hz, 1H), 2.67- 2.61 (m, 1H), 2.08 – 2.02 (m, 2H), 1.75 – 1.66 (m, 2H), 1.66 – 1.53 (m, 2H), 1.51 – 1.35 (m, 3H), 1.35 – 1.23 (m, 1H), 1.21 – 1.10 (m, 4H), 1.04 (d, *J* = 6.3 Hz, 3H, H-11), 1.00 (d, *J* = 6.1 Hz, 3H, H-11), 0.91 (t, *J* = 7.4 Hz, 3H, H-7).

^{13}C NMR (100 MHz, $CDCl_3$) δ = 159.2 (C-2), 121.6 (CN), 71.7 (C-1), 60.5, 51.5, 46.9, 43.7, 37.3, 33.7, 33.6, 28.1, 25.7, 24.7, 24.5, 24.3, 22.8, 11.9.

HRMS (ESI-Q-TOF) *m/z*: 276.2431 $[M+H]^+$; calcd. for $C_{17}H_{30}N_3$: 276.2434.

The enantiomeric ratio was determined by UPC^2 analysis using Trefoil CEL1, gradient CO_2/EtOH = 100:0 to 85:15 until 2 min then isocratic 85:15, flow rate = 1 mL/min, 25 °C, λ = 267 nm, t_R = 3.80 min (major) and t_R = 4.45 min (minor), 93% ee.

(3*R*,5*R*)-3-(*tert*-butylamino)-2-(cyclohexylamino)-5-ethylcyclopent-1-ene-1-carbonitrile (2d)

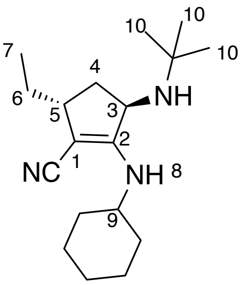

Prepared according the general procedure using hemiacetal **1a** (34 mg, 0.15 mmol), *tert*-butylamine (37 μL, 0.15 mmol) and cyclohexyl isocyanide (19 μL, 0.15 mmol). Obtained after flash column chromatography purification (*n*-hexane/EtOAc 5:1) as a pale yellow oil (39 mg, 0.13 mmol, 89% yield, > 99:1 dr, 94% ee). $[\alpha]_D^{20}$ – 10.3 (*c* 0.4, acetone, 20°C). R_f = 0.40 (*n*-hexane/ EtOAc 3:1).

^{1}H NMR (400 MHz, $CDCl_3$) δ = 5.18 (d, *J* = 8.6 Hz, 1H, H-8), 3.77 (dtd, *J* = 13.4, 9.3, 3.8 Hz, 1H, H-9), 3.60 (dd, *J* = 10.0, 7.2 Hz, 1H, H-3), 2.60 (td, *J* = 8.3, 5.1 Hz, 1H, H-5), 2.08 (dd, *J* = 12.5, 7.2 Hz, 1H, H-4a), 2.05 – 1.98 (m, 2H), 1.73 – 1.51 (m, 5H), 1.50 – 1.35 (m, 2H), 1.39 – 1.27 (m, 1H), 1.25 – 1.11 (m, 4H), 1.08 (s, 9H, H-10), 0.93 (t, *J* = 7.3 Hz, 3H, H-7).

^{13}C NMR (100 MHz, $CDCl_3$) δ = 160.1 (C-2), 121.7 (CN), 70.7 (C-1), 57.5, 51.2, 50.7, 43.5, 40.6, 33.7, 33.4, 30.6 (C-10), 28.1, 25.8, 24.3, 24.2, 12.1 (C-7).

HRMS (ESI-Q-TOF) *m/z*: 290.2598 $[M+H]^+$; calcd. for $C_{18}H_{32}N_3$: 290.2596.

The enantiomeric ratio was determined by UPC^2 analysis using Trefoil CEL1, isocratic gradient CO_2/MeCN = 95:5, flow rate = 1 mL/min, 25 °C, λ = 269 nm, t_R = 2.32 min (minor) and t_R = 2.49 min (major), 94% ee.

(3*R*,5*R*)-2-(cyclohexylamino)-5-ethyl-3-(prop-2-yn-1-ylamino)cyclopent-1-ene-1-carbonitrile (2e)

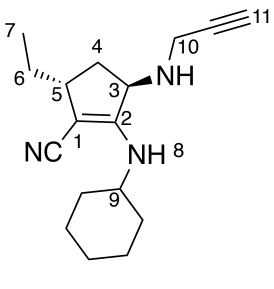

Prepared according the general procedure using hemiacetal **1a** (34 mg, 0.15 mmol), propargyl amine (10 μL, 0.15 mmol) and cyclohexyl isocyanide (19 μL, 0.15 mmol). Obtained after flash column chromatography purification (*n*-hexane/EtOAc 4:1) as a pale brown oil (40 mg, 0.15 mmol, 98% yield, 99:1

dr, 99% ee). $[\alpha]_D^{20}$ – 4.8 (*c* 0.5, acetone, 20°C). R_f= 0.55 (*n*-hexane/ EtOAc 7:3).

^{1}H NMR (400 MHz, $CDCl_3$) δ = 4.93 (d, *J* = 8.7 Hz, 1H, H-8), 3.78 – 3.65 (m, 2H, H-3, H-9), 3.45 – 3.27 (m, 2H, H-10), 2.70 – 2.59 (m, 1H), 2.20 (t, *J*=2.1, 1H, H-11), 2.06 – 1.93 (m, 3H), 1.72 – 1.49 (m, 6H), 1.40 – 1.30 (m, 2H), 1.30 – 1.16 (m, 1H), 1.16 – 1.01 (m, 3H), 0.86 (t, *J* = 7.4 Hz, 3H, H-7).

^{13}C NMR (100 MHz, $CDCl_3$) δ = 157.3 (C-2), 121.1 (CN), 81.9, 73.3 (C-1), 72.2 (C-11), 62.8, 51.8, 44.0, 36.2, 35.8, 33.7, 28.1, 25.7, 24.5, 24.4, 11.7 (C-7).

HRMS (ESI-Q-TOF) *m/z*: 272.2121 $[M+H]^+$; calcd. for $C_{17}H_{26}N_3$: 272.2121.

The enantiomeric ratio was determined by UPC^2 analysis using Trefoil CEL2, gradient CO_2/iPrOH = 100:0 to 90:10 in 24 min, flow rate = 0.8 mL/min, 25 °C, λ = 254 nm, t_R = 13.40 min (minor) and t_R = 14.35 min (major), 99% ee.

(3*R*,5*R*)-3-(allylamino)-2-(cyclohexylamino)-5-ethylcyclopent-1-ene-1-carbonitrile (2f)

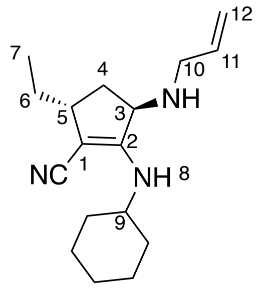

Prepared according the general procedure using hemiacetal **1a** (34 mg, 0.15 mmol), allylamine (11 µL, 0.15 mmol) and cyclohexyl isocyanide (19 µL, 0.15 mmol). Obtained after flash column chromatography purification (*n*-hexane/EtOAc 4:1) as a pale brown oil (26 mg, 0.10 mmol, 64% yield, 99:1 dr, 88% ee). $[\alpha]_D^{20}$ – 6.3 (*c* 0.5, acetone, 20°C). R_f= 0.39 (*n*-hexane/ EtOAc 7:3).

^{1}H NMR (400 MHz, $CDCl_3$) δ = 5.86 (ddt, *J* = 16.4, 10.7, 5.9 Hz, 1H, H-11), 5.19 (d, *J* = 17.2 Hz, 1H, H-12), 5.10 (d, *J* = 10.2 Hz, 1H, H-12), 5.05 (d, *J* = 8.7 Hz, 1H, H-8), 3.78 (tq, *J* = 10.1, 4.1 Hz, 1H, H-10), 3.68 (t, *J* = 8.1 Hz, 1H, H-3), 3.30 – 3.12 (m, 2H), 2.68 (q, *J* = 7.9 Hz, 1H), 2.10 – 1.93 (m, 3H), 1.75 – 1.66 (m, 2H), 1.65 – 1.54 (m, 3H), 1.48 – 1.34 (m, 2H), 1.34 – 1.23 (m, 1H), 1.22 – 1.07 (m, 2H), 0.91 (t, *J* = 7.4 Hz, 3H, H-7).

^{13}C NMR (100 MHz, $CDCl_3$) δ = 158.0 (C-2), 136.7 (C-11), 121.3 (CN), 116.3 (C-12), 72.6 (C-1), 62.3, 51.6, 49.4, 43.8, 35.7, 33.7, 33.6, 28.2, 25.7, 24.4, 24.3, 11.8 (C-7).

HRMS (ESI-Q-TOF) *m/z*: 276.2286 $[M+H]^+$; calcd. for $C_{17}H_{28}N_3$: 276.2278.

The enantiomeric ratio was determined by UPC^2 analysis using Trefoil CEL1, gradient CO_2/iPrOH = 100:0 to 85:15 in 9 min, flow rate = 2 mL/min, 25 °C, λ = 254 nm, t_R = 7.28 min (major) and t_R = 8.48 min (minor), 88% ee.

(3*R*,5*R*)-2-(cyclohexylamino)-5-ethyl-3-(propylamino)cyclopent-1-ene-1-carbonitrile (2g)

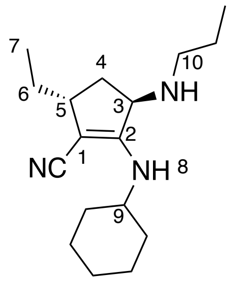

Prepared according the general procedure using hemiacetal **1a** (34 mg, 0.15 mmol), *n*-propylamine (12 µL, 0.15 mmol) and cyclohexyl isocyanide (18.7 µL, 0.15 mmol). Obtained after flash column chromatography purification (*n*-hexane/EtOAc 4:1) as a pale brown oil (16 mg, 0.06 mmol, 38% yield, 99:1 dr, 88% ee). $[\alpha]_D^{20}$ + 1.3 (*c* 0.2, acetone, 20°C). R_f = 0.35 (*n*-hexane/ EtOAc 7:3).

^{1}H NMR (400 MHz, $CDCl_3$) δ = 5.15 (d, *J* = 8.6 Hz, 1H, H-8), 3.85 – 3.71 (m, 1H, H-9), 3.69 (t, *J* = 8.1 Hz, 1H, H-3), 2.74 – 2.62 (m, 1H), 2.52 (dddd, *J* = 17.8, 14.2, 11.4, 6.7 Hz, 2H), 2.04 (d, *J* = 11.4 Hz, 2H), 1.96 (ddd, *J* = 12.8, 7.4, 1.9 Hz, 1H), 1.76 – 1.62 (m, 2H), 1.67 – 1.54 (m, 3H), 1.53 – 1.38 (m, 4H), 1.37 – 1.21 (m, 2H), 1.22 – 1.08 (m, 3H), 0.92 (t, *J* = 7.4 Hz, 1H), 0.91 (t, *J* = 7.4 Hz, 1H, H-7).

^{13}C NMR (100 MHz, $CDCl_3$) δ = 157-8 (C-2), 121.3 (CN), 72.7 (C-2), 62.7 , 51.6, 48.2, 43.9, 35.2, 33.7, 33.6, 28.2, 25.7, 24.4, 24.3, 23.7, 11.9, 11.8 (C-7).

HRMS (ESI-Q-TOF) *m/z*: 274.2445 $[M+H]^+$; calcd. for $C_{17}H_{28}N_3$: 274.2434.

The enantiomeric ratio was determined by UPC^2 analysis using Trefoil CEL2, gradient CO_2/iPrOH = 100:0 to 90:10 in 24 min, flow rate = 0.8 mL/min, 25 °C, λ = 254 nm, t_R = 13.40 min (minor) and t_R = 14.35 min (major), 88% ee.

(3*R*,5*R*)-3-(benzylamino)-2-(cyclohexylamino)-5-ethylcyclopent-1-ene-1-carbonitrile (2h)

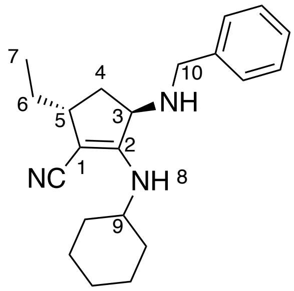

Prepared according the general procedure using hemiacetal **1a** (34 mg, 0.15 mmol), benzylamine (16 µL, 0.15 mmol), and cyclohexyl isocyanide (19 µL, 0.15 mmol). Obtained after flash column chromatography purification as an orange oil (25 mg, 0.12 mmol, 77% yield, 80:20 dr, 88% ee). $[\alpha]_D^{20}$ – 9.2 (*c* 0.5, acetone, 20°C). R_f = 0.32 (*n*-hexane/ EtOAc 4:1).

^{1}H NMR (400 MHz, $CDCl_3$) δ = 7.36 – 7.25 (m, 5H, Ph), 5.05 (d, *J* = 8.7 Hz, 1H, H-8), 3.82 – 3.69 (m, 4H), 2.74 – 2.67 (m, 1H, H-9), 2.05 – 1.95 (m, 3H), 1.77 – 1.53 (m, 6H), 1.48 – 1.36 (m, 2H), 1.34 – 1.07 (m, 5H), 0.91 (t, *J* = 7.4 Hz, 3H, H-7).

^{13}C NMR (100 MHz, $CDCl_3$) δ = 157.9 (C-2), 39.9, 128.6, 128.2, 127.4, 121.2 (CN), 72.9 (C-1), 62.4, 51.6, 50.7, 43.9, 35.5, 33.7, 28.2, 25.7, 24.4, 24.3, 11.7 (C-7).

HRMS (ESI-Q-TOF) *m/z*: 324.2443 $[M+H]^+$; calcd. for $C_{21}H_{30}N_3$: 324.2440.

The enantiomeric ratio was determined by UPC^2 analysis using Trefoil AMY1, gradient CO_2/iPrOH = 100:0 to 60:40 until 5 min then isocratic 60:40, flow rate = 1 mL/min, 35 °C, λ = 269 nm, t_R = 5.32 min (minor) and t_R = 5.47 min (major), 88% ee.

(3*R*,5*R*)-2-(cyclohexylamino)-5-ethyl-3-(((*S*)-1-phenylethyl)amino)cyclopent-1-ene-1-carbonitrile (2i)

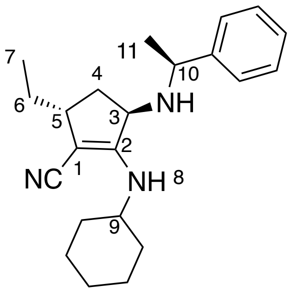

Prepared according the general procedure using hemiacetal **1a** (34 mg, 0.15 mmol), (*S*)-α-methylbenzylamine (19 μL, 0.15 mmol) and cyclohexyl isocyanide (19 μL, 0.15 mmol). Obtained after flash column chromatography purification (*n*-hexane/EtOAc 5:1) as a brown oil (46 mg, 0.14 mmol, 91% yield, 83:17 dr). $[\alpha]_D^{20}$ – 5.9 (*c* 0.6, acetone, 20°C). R_f = 0.37 (*n*-hexane/ EtOAc 4:1).

^{1}H NMR (400 MHz, $CDCl_3$) δ = 7.29 – 7.24 (m, 3H, Ph), 7.23 – 7.16 (m, 2H, Ph), 4.86 (d, *J* = 8.7, 1H, H-8), 3.80 (q, *J* = 6.5, 1H, H-10), 3.74 – 3.58 (m, 1H, H-9), 3.28 (t, *J* = 7.6, 1H, H-3), 2.64 – 2.47 (m, 1H), 2.03 – 1.85 (m, 3H), 1.70 – 1.57 (m, 3H), 1.53 – 1.33 (m, 4H), 1.26 (d, *J* = 6.6, 3H, H-11), 1.21 – 1.00 (m, 6H), 0.77 (t, *J* = 7.4, 3H, H-7).

^{13}C NMR (100 MHz, $CDCl_3$) δ = 158.9 (C-2), 144.9, 128.7, 127.5, 126.9, 121.3 (CN), 72.2 (C-1) , 60.4, 56.1, 51.4, 43.7, 36.6, 33.7, 33.6, 28.2, 25.7, 25.6 (C-11), 24.4, 24.3, 11.8 (C-7).

HRMS (ESI-Q-TOF) *m/z*: 338.2596 $[M+H]^+$; calcd. for $C_{22}H_{32}N_3$: 338.2599.

Methyl ((1*R*,4*R*)-3-cyano-2-(cyclohexylamino)-4-ethylcyclopent-2-en-1-yl)-*L*-valinate (2j)

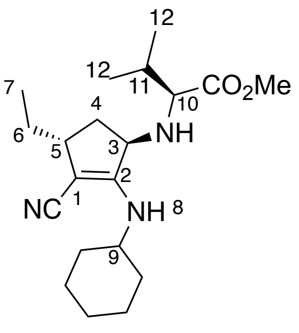

Prepared according the general procedure using hemiacetal **1a** (34 mg, 0.15 mmol), *L*-valine methyl ester hydrochloride (25 mg, 0.15 mmol) and cyclohexyl isocyanide (19 μL, 0.15 mmol) and triethylamine (23 μL, 0.16 mmol). Obtained after flash column chromatography purification (*n*-hexane/EtOAc 9:1) as a brown oil (35 mg, 0.10 mmol, 67% yield, 86:14 dr).

$[\alpha]_D^{20}$ – 28.2 (*c* 0.6, acetone, 20°C). R_f= 0.33 (*n*-hexane/ EtOAc 4:1).

^{1}H NMR (400 MHz, $CDCl_3$) δ = 5.27 (d, *J* = 8.4 Hz, 1H, H-8), 3.72 (s, 3H, OMe), 3.63 (t, *J* = 7.7 Hz, 1H, H-3), 3.01 (d, *J* = 5.1 Hz, 1H, H-10), 2.72 – 2.61 (m, 1H, H-9), 2.09 – 2.02 (m, 2H), 1.94 (td, *J* = 13.3 and 6.8 Hz, 1H), 1.76 – 1.69 (m, 4H), 1.63 – 1.56 (m, 3H), 1.38 – 1.17 (m, 7H), 0.94 (d, *J* = 6.8 Hz, 3H, H-12), 0.90 (d, *J* = 6.9 Hz, 3H, H-12), 0.89 (t, *J* = 7.5 Hz, 3H, H-7).

^{13}C NMR (100 MHz, $CDCl_3$) δ = 176.2 (C=O), 157.6 (C-2), 121.2 (CN), 73.1 (C-1), 63.7, 62.1, 52.1, 51.6, 43.8, 34.8, 33.7, 33.6, 31.9, 27.9, 25.7, 24.4, 24.3, 19.6, 18.4, 11.8 (C-7).

HRMS (ESI-Q-TOF) *m/z*: 348.2654 $[M+H]^+$; calcd. for $C_{22}H_{32}N_3$: 348.2646.

(3*R*,5*R*)-2-(cyclohexylamino)-5-ethyl-3-(phenylamino)cyclopent-1-ene-1-carbonitrile (2k)

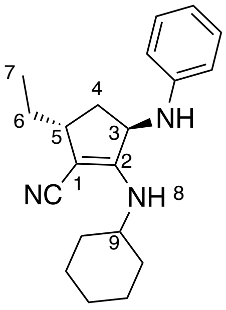

Prepared according the general procedure using hemiacetal **1a** (34 mg, 0.15 mmol), aniline (14 µL, 0.15 mmol) and cyclohexyl isocyanide (19 µL, 0.15 mmol). Obtained after flash column chromatography purification (*n*-hexane/EtOAc 4:1) as a pale yellow oil (41 mg, 0.13 mmol, 88% yield, 92:8 dr, 99% ee). $[\alpha]_D^{20}$–10.1 (*c* 0.6, acetone, 20°C). R_f= 0.35 (*n*-hexane/ EtOAc 3:1).

^{1}H NMR (400 MHz, $CDCl_3$) δ = 7.21 (t, *J* = 7.7 Hz, 2H, Ph), 6.81 (t, *J* = 7.4 Hz, 1H, Ph), 6.68 (d, *J* = 8.0 Hz, 2H, Ph), 4.71 (d, *J* = 8.7 Hz, 1H, H-8), 4.49 (t, *J* = 7.8 Hz, 1H, H-3), 3.89 – 3.70 (m, 1H, H-9), 2.80 – 2.71 (m, 1H), 2.11 – 1.94 (m, 3H), 1.93 – 1.80 (m, 1H), 1.77 – 1.57 (m, 4H), 1.50 – 1.05 (m, 7H), 0.94 (t, *J* = 7.3 Hz, 2H, H-7).

^{13}C NMR (100 MHz, $CDCl_3$) δ = 157.2 (C-2), 146.2 (Ph), 129.6 (Ph), 120.7 (CN), 119.3 (Ph), 114.5 (Ph), 74.2 (C-1), 59.7, 52.0, 43.6, 34.7, 33.7, 28.1, 25.6, 24.4, 11.6 (C-7).

HRMS (ESI-Q-TOF) *m/z*: 310.2283 $[M+H]^+$; calcd. for $C_{20}H_{28}N_3$: 310.2288.

The enantiomeric ratio was determined by UPC^2 analysis using Trefoil AMY1, gradient CO_2/iPrOH = 100:0 to 60:40 until 5 min then isocratic 60:40, flow rate = 1 mL/min, 35 °C, λ = 265 nm, t_R = 5.84 min (major) and t_R = 5.99 min (minor), 99% ee.

(3*R*,5*R*)-2-(cyclohexylamino)-5-ethyl-3-((4-methoxyphenyl)amino)cyclopent-1-ene-1-carbonitrile (2l)

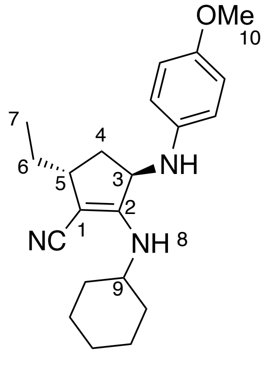

Prepared according the general procedure using hemiacetal **1a** (34 mg, 0.15 mmol), *p*-anisidine (18 mg, 0.15 mmol) and cyclohexyl isocyanide (19 µL, 0.15 mmol). Obtained after flash column chromatography purification (*n*-hexane/EtOAc 5:1) as a brown oil (41 mg, 0.12 mmol, 80% yield, 90:10 dr, 94% ee). $[\alpha]_D^{20}$ – 11.7 (*c* 0.6, acetone, 20°C). R_f = 0.36 (*n*-hexane/ EtOAc 4:1).

^{1}H NMR (400 MHz, $CDCl_3$) δ =7.24-7.20 (m, 2H, Ph), 7.16-6.82 (m, 2H, Ph), 5.98 (d, *J* = 8.2 Hz, 1H, H-8), 4.52-4.38 (m, 1H, H-9), 4.13 (t, *J* = 7.2 Hz, 1H, H-3), 3.68 (s, 3H, H-10), 2.29-2.09 (m, 2H, H-4), 2.00-1.92 (m, 2H), 1.85-1.57 (m, 6H), 1.55-1.39 (m, 4H), 1.28-1.10 (m, 1H), 0.89 (t, *J* = 7.4 Hz, 3H, H-7).

^{13}C NMR (100 MHz, $CDCl_3$) δ = 157.2 (C-2), 153.2, 140.2, 120.7, 115.9, 115.0, 114.1 (CN), 73.7 (C-1), 60.5, 55.7, 51.8, 43.5, 34.6, 33.6, 33.5, 25.5, 24.3, 24.2, 11.5 (C-7).

HRMS (ESI-Q-TOF) *m/z*: 340.2391 $[M+H]^+$; calcd. for $C_{21}H_{30}N_3O$: 340.2389.

The enantiomeric ratio was determined by UPC^2 analysis using Trefoil AMY1, gradient CO_2/iPrOH = 100:0 to 80:20 until 5 min then isocratic 80:20, flow rate = 1 mL/min, 25 °C, λ = 265 nm, t_R = 5.90 min (major) and t_R = 6.01 min (minor), 94% ee.

(3*R*,5*R*)-2-(cyclohexylamino)-5-ethyl-3-(*p*-tolylamino)cyclopent-1-ene-1-carbonitrile (2m)

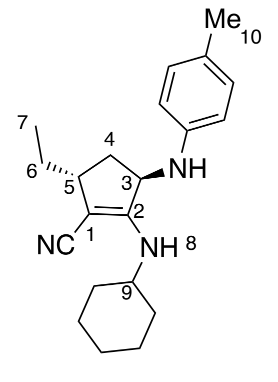

Prepared according the general procedure using hemiacetal **1a** (34 mg, 0.15 mmol), *p*-toluidine (16 µL, 0.15 mmol) and cyclohexyl isocyanide (19 µL, 0.15 mmol). Obtained after flash column chromatography purification (*n*-hexane/EtOAc 4:1) as a pale brown oil (46 mg, 0.14 mmol, 95% yield, 91:9 dr, 92% ee). $[\alpha]_D^{20}$ – 7.9 (*c* 0.5, acetone, 20°C). R_f = 0.76 (*n*-hexane/ EtOAc 7:3).

^{1}H NMR (400 MHz, $CDCl_3$) δ = 7.01 (d, *J* = 8.6, 2H), 6.58 (d, *J* = 8.4 Hz, 2H), 4.69 (d, *J* = 8.6 Hz, 1H, H-8), 4.44 (t, *J* = 7.9 Hz, 1H), 3.86 – 3.74 (m, 1H), 2.25 (s, 3H, H-10), 2.10 – 2.00 (m, 2H), 1.97 (ddd, *J* = 13.0, 7.6, 2.5 Hz, 1H), 1.81 (dt, *J* = 13.0, 8.3 Hz, 1H), 1.73 – 1.55 (m, 5H), 1.47 – 1.30 (m, 3H), 1.21 – 1.03 (m, 3H), 0.93 (t, *J* = 7.4 Hz, 3H, H-7).

^{13}**C NMR** (100 MHz, $CDCl_3$) δ = 157.5 (C-2), 144.0, 130.0, 128.4, 120.7, 114.5, 73.8 (C-1), 59.8, 51.8, 43.5, 34.7, 33.6, 28.1, 25.5, 24.3, 24.2, 20.4, 11.5 (C-7).

HRMS (ESI-Q-TOF) *m/z*: 324.2445 $[M+H]^+$; calcd. for $C_{21}H_{30}N_3$: 324.2434.

The enantiomeric ratio was determined by HLPC analysis using Chiralpak AS-H, isocratic Hex/EtOH = 90:10, flow rate = 0.5 mL/min, 25 °C, λ = 254 nm, t_R = 12.0 min (minor) and t_R = 16.0 min (major), 92% ee.

(3*R*,5*R*)-3-((4-chlorophenyl)amino)-2-(cyclohexylamino)-5-ethylcyclopent-1-ene-1-carbonitrile (2n)

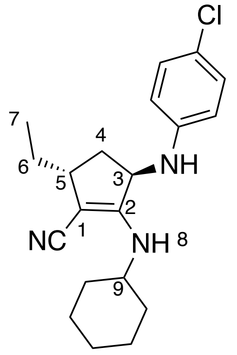

Prepared according the general procedure using hemiacetal **1a** (34 mg, 0.15 mmol), 4-chloroaniline (19 mg, 0.15 mmol) and cyclohexyl isocyanide (19 µL, 0.15 mmol). Obtained after flash column chromatography purification (*n*-hexane/EtOAc 9:1) as a brown solid (51 mg, 0.15 mmol, 99% yield, > 99:1 dr, > 99% ee). m.p. 78-82°C. $[\alpha]_D^{20}$ – 8.9 (*c* 0.5, acetone, 20°C). R_f = 0.53 (*n*-hexane/ EtOAc 4:1).

^{1}**H NMR** (400 MHz, $CDCl_3$) δ = 7.14 (d, *J* = 8.4 Hz, 2H), 6.58 (d, *J* = 8.4 Hz, 2H), 4.64 (d, *J* = 8.6 Hz, 1H, H-8), 4.38 (t, *J* = 8.2 Hz, 1H, H-3), 3.89 – 3.74 (m, 1H), 3.50 (brs, 1H, NH), 2.83 – 2.54 (m, 1H), 2.17 – 1.77 (m, 4H), 1.76 – 1.55 (m, 4H), 1.49 – 1.04 (m, 7H), 0.92 (t, *J* = 7.4 Hz, 3H, H-7).

^{13}**C NMR** (100 MHz, $CDCl_3$) δ = 156.9 (C-2), 145.0 (C), 129.5 (CH), 123.8 (C), 120.5 (CN), 115.6 (C), 115.5 (CH), 74.6 (CN), 59.8, 52.1, 43.7, 34.5, 33.8, 28.2, 25.6, 24.5, 24.3, 11.6 (C-7).

HRMS (ESI-Q-TOF) *m/z*: 344.1906 $[M+H]^+$; calcd. for $C_{20}H_{26}ClN_3$: 344.1888.

The enantiomeric ratio was determined by UPC^2 analysis using Trefoil AMY1, gradient CO_2/MeOH = 100:0 to 60:40 in 6 min, flow rate = 1 mL/min, 35 °C, λ = 315 nm, t_R = 4.79 min (minor) and t_R = 4.84 min (major), > 99% ee.

(3*R*,5*R*)-3-((4-bromophenyl)amino)-2-(cyclohexylamino)-5-ethylcyclopent-1-ene-1-carbonitrile (2o)

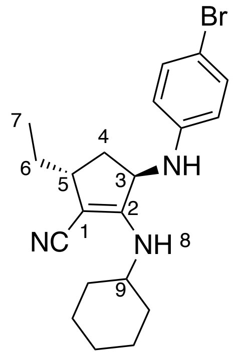

Prepared according the general procedure using hemiacetal **1a** (34 mg, 0.15 mmol), 4-bromoaniline (26 mg, 0.15 mmol) and cyclohexyl isocyanide (19 μL, 0.15 mmol). Obtained after flash column chromatography purification (*n*-hexane/EtOAc 5:1) as a dark yellow oil (45 mg, 0.12 mmol, 77% yield, 99:1 dr, 97% ee). $[\alpha]_D^{20}$ – 15.4 (*c* 0.6, acetone, 20°C). R_f = 0.39 (*n*-hexane/ EtOAc 4:1).

^{1}H NMR (400 MHz, $CDCl_3$) δ = 7.20 (d, *J* = 8.7, 2H, Ph), 6.46 (d, *J* = 8.7, 2H, Ph), 4.52 (d, *J* = 8.6 Hz, 1H, H-8), 4.36 (t, *J* = 7.8 Hz, 1H, H-3), 3.75-3.61 (m, 1H, H-9), 2.76-2.65 (m, 1H), 2.03-1.93 (m, 2H), 1.91-1.71 (m, 2H, H-4), 1.67-1.51 (m, 4H), 1.41-1.23 (m, 3H), 1.14-0.97 (m, 3H), 0.86 (t, *J* = 7.3 Hz, 3H, H-7).

^{13}C NMR (100 MHz, $CDCl_3$) δ = 156.9 (C-2), 145.5, 132.3, 120.5, 115.9, 110.7 (CN), 74.5 (C-1), 59.6, 52.0, 43.6, 34.5, 33.7, 28.1, 25.6, 24.4, 24.3, 24.2, 11.6 (C-7).

HRMS (ESI-Q-TOF) *m/z*: 388.1392 $[M+H]^+$; calcd. for $C_{20}H_{27}BrN_3$: 388.1388.

The enantiomeric ratio was determined by UPC^2 analysis using Trefoil AMY1, gradient CO_2/MeCN = 100:0 to 80:20 until 5 min then isocratic 80:20, flow rate = 1 mL/min, 25 °C, λ = 261 nm, t_R = 5.65 min (major) and t_R = 5.76 min (minor), 97% ee.

(3*R*,5*R*)-3-((4-acetylphenyl)amino)-2-(cyclohexylamino)-5-ethylcyclopent-1-ene-1-carbonitrile (2p)

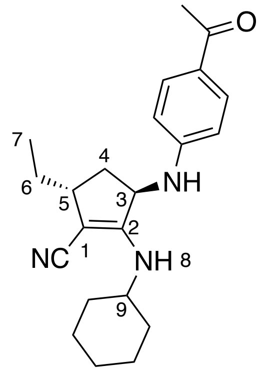

Prepared according the general procedure using hemiacetal **1a** (34 mg, 0.15 mmol), 4'-aminoacetophenone (20 mg, 0.15 mmol) and cyclohexyl isocyanide (19 μL, 0.15 mmol). Obtained after flash column chromatography purification (*n*-hexane/EtOAc 9:1) as an orange oil (30 mg, 0.08 mmol, 56% yield, 58:42 dr, 94% ee). $[\alpha]_D^{20}$ – 2.1 (*c* 0.5, acetone, 20°C). R_f = 0.20 (*n*-hexane/ EtOAc 4:1).

^{1}H NMR (400 MHz, $CDCl_3$) δ = 7.83 (d, *J* = 8.6 Hz, 2H, Ph), 6.72 – 6.54 (m, 2H, Ph), 4.67 – 4.42 (m, 2H, H-3, H-8), 3.78 (brs, 1H, NH), 2.82 – 2.76 (m, 1H, H-9), 2.51 (s, 3H, $CH_3C=O$), 2.10 – 1.77 (m, 4H), 1.80 – 1.57 (m, 4H), 1.48 – 1.21 (m, 3H), 1.21 – 1.06 (m, 3H), 0.94 (t, *J* = 7.2 Hz, 3H, H-7).

^{13}C NMR (100 MHz, $CDCl_3$) δ = 196.5 (C=O), 156.4 (C-2), 150.8 (C), 130.9 (CH), 128.2 (C), 120.2 (CN), 112.8 (CH), 75.8 (C-1), 59.0, 52.2, 43.7, 34.9, 33.8, 33.7, 28.1, 26.2, 25.5, 24.4, 24.3, 11.5 (C-7).

HRMS (ESI-Q-TOF) *m/z*: 352.2391 $[M+H]^+$; calcd. for $C_{22}H_{29}N_3O$: 352.2383.

The enantiomeric ratio was determined by UPC^2 analysis using Trefoil CEL2, gradient CO_2/iPrOH = 100:0 to 55:45 in 7 min, flow rate = 1 mL/min, 35 °C, λ = 315 nm, t_R = 5.48 min (major) and t_R = 5.66 min (minor), 94% ee.

(3*R*,5*R*)-2-(cyclohexylamino)-5-ethyl-3-((4-(trifluoromethyl)phenyl)amino) cyclopent-1-ene-1-carbonitrile (2q)

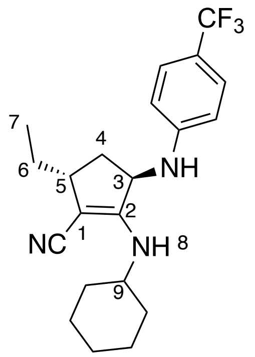

Prepared according the general procedure using hemiacetal **1a** (34 mg, 0.15 mmol), 4-trifluoromethylaniline (19 µL, 0.15 mmol) and cyclohexyl isocyanide (19 µL, 0.15 mmol). Obtained after flash column chromatography purification (*n*-hexane/EtOAc 5:1) as a pale orange oil (25 mg, 0.07 mmol, 44% yield, 70:30 dr, 96% ee). $[\alpha]_D^{20}$ – 6.8 (*c* 0.5, acetone, 20°C). R_f = 0.31 (*n*-hexane/ EtOAc 3:1).

^{1}H NMR (400 MHz, $CDCl_3$) δ =7.81 (d, *J* = 8.6 Hz, 2H, Ph), 7.48 (d, *J* = 8.7 Hz, 2H, Ph), 6.13 (d, *J* = 8.0 Hz, 1H, H-8), 3.92 (t, *J* = 7.9 Hz, 1H, H-3), 3.80-3.68 (m, 1H, H-9), 2.85-2.73 (m, 1H), 2.45-2.40 (m, 1H), 2.27-2.23 (m, 2H), 1.68-1.55 (m, 2H), 1.52-1.42 (m, 2H), 1.40-1.28 (m, 3H), 1.26-1.22 (m, 1H), 1.20-1.12 (m, 3H), 0.90 (t, J = 7.4 Hz, 3H, H-7).

^{13}C NMR (100 MHz, $CDCl_3$) δ = 156.5 (C-2), 149.2, 133.8, 126.9 (q, *J* = 3.9 Hz), 124.9 (q, *J* = 270.5 Hz), 120.5 (q, *J* = 33.6 Hz), 120.2 (CN), 114.3, 113.3, 75.0 (C-1), 59.2, 52.2, 43.7, 34.6, 33.8, 33.7, 28.1, 25.5, 24.4, 24.3, 11.5 (C-7).

HRMS (ESI-Q-TOF) *m/z*: 378.2160 $[M+H]^+$; calcd. for $C_{21}H_{27}F_3N_3$: 378.2157.

The enantiomeric ratio was determined by UPC^2 analysis using Trefoil CEL2, gradient CO_2/iPrOH = 100:0 to 60:40 until 5 min then isocratic 60:40, flow rate = 1 mL/min, 35 °C, λ = 265 nm, t_R = 2.90 min (minor) and t_R = 3.07 min (major), 96% ee.

(3*R*,5*R*)-2-(cyclohexylamino)-5-ethyl-3-((3-methoxyphenyl)amino)cyclopent-1-ene-1-carbonitrile (2s)

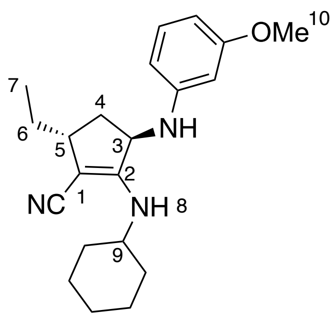

Prepared according the general procedure using hemiacetal **1a** (34 mg, 0.15 mmol), *m*-anisidine (16 µL, 0.15 mmol) and cyclohexyl isocyanide (19 µL, 0.15 mmol). Obtained after flash column chromatography purification (*n*-hexane/EtOAc 4:1) as a pale brown oil (50 mg, 0.15 mmol, 99% yield, 88:12 dr, 92% ee). $[\alpha]_D^{20}$ – 5.1 (*c* 0.5, acetone, 20°C). R_f = 0.79 (*n*-hexane/ EtOAc 7:3).

^{1}H NMR (400 MHz, $CDCl_3$) δ = 6.90 – 6.70 (m, 3H), 6.66 (dd, *J* = 7.8, 1.5 Hz, 1H), 4.68 (d, *J* = 8.6 Hz, 1H, H-8), 4.50 (t, *J* = 7.9 Hz, 1H), 3.85 (s, 3H, H-10), 3.85 – 3.73 (m, 1H), 2.83 – 2.72 (m, 1H), 2.11 – 2.00 (m, 2H), 2.00 – 1.76 (m, 2H), 1.79 – 1.54 (m, 4H), 1.48 – 1.31 (m, 3H), 1.23 – 1.03 (m, 3H), 0.94 (t, *J* = 7.4 Hz, 3H, H-7).

^{13}C NMR (100 MHz, $CDCl_3$) δ = 157.8 (C-2), 147.5, 136.3, 121.3, 120.9, 118.2, 111.7, 110.1, 74.0 (C-1), 59.3, 55.6 (C-10), 52.0, 43.7, 42.1, 34.7, 33.7, 28.2, 25.6, 24.4, 24.3, 11.7 (C-7).

HRMS (ESI-Q-TOF) *m/z*: 340.2380 $[M+H]^+$; calcd. for $C_{21}H_{31}N_3O$: 340.2383.

The enantiomeric ratio was determined by UPC^2 analysis using Trefoil CEL1, gradient CO_2/iPrOH = 100:0 to 85:15 in 2 min then isocratic 85:15, flow rate = 1 mL/min, 25 °C, λ = 266 nm, t_R = 4.62 min (major) and t_R = 5.05 min (minor), 92% ee.

(3*R*,5*R*)-2-(cyclohexylamino)-5-ethyl-3-((2-methoxyphenyl)amino)cyclopent-1-ene-1-carbonitrile (2t)

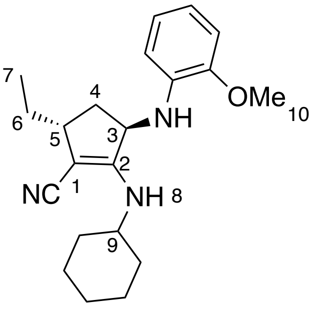

Prepared according general procedure using hemiacetal **1a** (35 mg, 0.15 mmol), 2-methoxyaniline (15.7 µL, 0.15 mmol) and cyclohexylisocyanide (18.7 µL, 0.15 mmol). Obtained after flash column chromatography purification (*n*-hexane/EtOAc 4:1) as a pale brown oil (43 mg, 0.13 mmol, 89% yield, 75:25 dr, 91% ee). $[\alpha]_D^{20}$ – 7.5 (*c* 0.5, acetone, 20°C). R_f= 0.66 (*n*-hexane/ EtOAc 7:3).

^{1}H NMR (400 MHz, $CDCl_3$) δ = 7.11 (t, *J* = 8.1 Hz, 1H), 6.35 (ddd, *J* = 8.2, 2.4, 0.8 Hz, 1H), 6.27 (ddd, *J* = 8.1, 2.3, 0.8 Hz, 1H), 6.21 (t, *J* = 2.3 Hz, 1H), 4.62 (d, *J* = 8.6 Hz, 1H, H-8), 4.47 (t, *J* = 7.7 Hz, 1H), 3.77 (s, 3H, H-10), 3.65 – 3.52 (m, 1H), 2.83 –

2.69 (m, 1H), 2.08 – 2.01 (m, 2H), 1.98 (dd, *J* = 7.6, 2.7 Hz, 1H), 1.83 (dt, *J* = 13.0, 8.1 Hz, 1H), 1.74 – 1.66 (m, 2H), 1.64 – 1.57 (m, 2H), 1.48 – 1.31 (m, 3H), 1.21 – 1.05 (m, 3H), 0.94 (t, *J* = 7.4 Hz, 3H, H-7).

^{13}C NMR (100 MHz, $CDCl_3$) δ = 161.0, 157.4 (C-2), 148.0, 130.4, 120.7, 107.1, 104.0, 100.5, 74.3 (C-1), 59.6, 55.3 (C-10), 52.0, 43.7, 35.1, 33.8, 33.7, 28.2, 25.6, 24.4, 24.3, 11.6 (C-7).

HRMS (ESI-Q-TOF) *m/z*: 340.2384 $[M+H]^+$; calcd. for $C_{21}H_{30}N_3O$: 340.2383.

The enantiomeric ratio was determined by HLPC analysis using Chiralpak AS-H, isocratic Hex/EtOH = 90:10, flow rate = 0.5 mL/min, 25 °C, λ = 254 nm, t_R = 25.5 min (major) and t_R = 33.5 min (minor), 91% ee.

(3*R*,5*R*)-2-(cyclohexylamino)-5-ethyl-3-((3-(trifluoromethyl)phenyl)amino) cyclopent-1-ene-1-carbonitrile (2u)

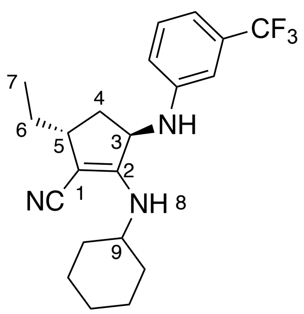

Prepared according the general procedure using hemiacetal **1a** (34 mg, 0.15 mmol), 3-(trifluoromethyl)aniline (15 μL, 0.15 mmol) and cyclohexyl isocyanide (19 μL, 0.15 mmol). Obtained after flash column chromatography purification (*n*-hexane/EtOAc 9:1) as a yellow oil (47 mg, 0.12 mmol, 83% yield, 91:9 dr, > 99% ee). $[\alpha]_D^{20}$ – 4.6 (*c* 0.5, acetone, 20°C). R_f = 0.55 (*n*-hexane/ EtOAc 4:1).

^{1}H NMR (400 MHz, $CDCl_3$) δ = 7.28 (t, *J* = 8.0 Hz, 1H), 7.01 (dt, *J* = 8.0, 2.0 Hz 1H), 6.86 (t, *J* = 2.0 Hz, 1H), 6.80 (dd, *J* = 8.0, 2.0 Hz, 1H), 4.57 (d, *J* = 8.6 Hz, 1H, H-8), 4.50 (t, *J* = 7.7 Hz, 1H, H-3), 3.94 (brs, 1H, NH), 3.79 (tdd, *J* = 10.3, 8.5, 5.2 Hz, 1H, H-9), 2.79 (ddtd, *J* = 9.5, 4.3, 3.0, 1.5 Hz, 1H, H-5), 2.12 – 2.01 (m, 2H), 1.97 (ddd, *J* = 13.0, 7.7, 3.0 Hz, 1H), 1.86 (dt, *J* = 13.0, 7.7 Hz, 1H), 1.76 – 1.54 (m, 4H), 1.47 – 1.31 (m, 3H), 1.20 – 1.05 (m, 3H), 0.94 (t, *J* = 7.4 Hz, 3H, H-7).

^{13}C NMR (100 MHz, $CDCl_3$) δ = 156.7 (C-2), 146.9 (C), 131.9 (q, *J* = 32.0 Hz, CH), 130.0, 124.2 (q, *J* = 272.3 Hz, CF_3), 120.4 (CN), 117.0 (CH), 115.3 (q, *J* = 3.9 Hz, CH), 110.5 (q, *J* = 3.6 Hz, CH), 74.8 (C-1), 59.4, 52.1, 43.7, 34.6, 33.7, 33.6, 28.1, 25.5, 24.4, 24.2, 11.5 (C-7).

HRMS (ESI-Q-TOF) *m/z*: 378.2159 $[M+H]^+$; calcd. for $C_{21}H_{26}F_3N_3$: 378.2152.

The enantiomeric ratio was determined by UPC^2 analysis using Trefoil CEL1, gradient CO_2/EtOH = 100:0 to 60:40 in 5 min, flow rate = 1 mL/min, 35 °C, λ = 318 nm, t_R = 3.81 min (minor) and t_R = 3.88 min (major), > 99% ee.

(3*R*,5*R*)-2-(cyclohexylamino)-5-ethyl-3-(*o*-tolylamino)cyclopent-1-ene-1-carbonitrile (2w)

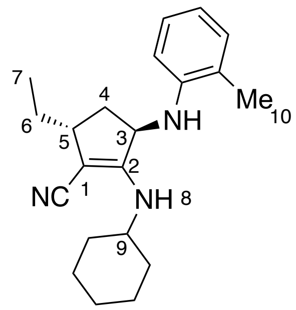

Prepared according the general procedure using hemiacetal **1a** (34 mg, 0.15 mmol), *o*-toluidine (16 µL, 0.15 mmol) and cyclohexyl isocyanide (19 µL, 0.15 mmol). Obtained after flash column chromatography purification (*n*-hexane/EtOAc 6:1) as a pale orange oil (25 mg, 0.10 mmol, 68% yield, 99:1 dr, 97% ee). $[\alpha]_D^{20}$– 7.2 (*c* 0.5, acetone, 20°C). R_f = 0.42 (*n*-hexane/ EtOAc 3:1).

^{1}H NMR (400 MHz, $CDCl_3$) δ =7.20-7.04 (m, 2H, Ph), 6.78-6.70 (m, 1H, Ph), 6.67 (d, *J* = 8.0 Hz, 1H, Ph), 4.65 (d, *J* = 7.9 Hz, 1H, H-8), 4.54 (t, *J* = 7.9 Hz, 1H, H-3), 3.87-3.63 (m, 1H, H-9), 2.89-2.72 (m, 1H), 2.15 (s, 3H, H-10), 2.07-2.00 (m, 2H), 1.89-1.79 (m, 1H), 1.73-1.58 (m, 4H), 1.21-1.06 (m, 3H), 0.94 (t, J = 7.4 Hz, 3H, H-7).

^{13}C NMR (100 MHz, $CDCl_3$) δ = 157.6 (C-2), 144.6, 130.8, 127.4, 123.3 (CN), 120.6, 118.6, 111.4, 74.5 (C-1), 59.4, 51.9, 43.7, 35.5, 33.7, 29.8, 28.2, 25.6, 24.4, 24.3, 17.8 (C-10), 11.6 (C-7).

HRMS (ESI-Q-TOF) *m/z*: 324.2436 $[M+H]^+$; calcd. for $C_{21}H_{30}N_3$: 324.2440.

The enantiomeric ratio was determined by UPC^2 analysis using Trefoil AMY1, gradient CO_2/iPrOH = 100:0 to 80:20 in 5 min then isocratic 80:20, flow rate = 1 mL/min, 25 °C, λ = 269 nm, t_R = 5.07 min (major) and t_R = 5.20 min (minor), 97% ee.

(3*R*,5*R*)-3-((3-bromophenyl)amino)-2-(cyclohexylamino)-5-ethylcyclopent-1-ene-1-carbonitrile (2x)

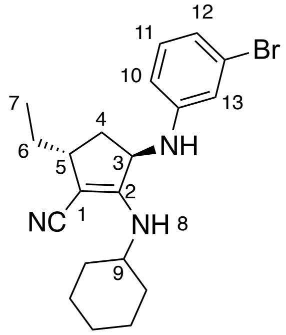

Prepared according the general procedure using hemiacetal **1a** (34 mg, 0.15 mmol), *m*-bromoaniline (26 mg, 0.15 mmol) and cyclohexyl isocyanide (19 µL, 0.15 mmol). Obtained after flash column chromatography purification (*n*-hexane/EtOAc 9:1) as a yellow oil (37 mg, 0.10 mmol, 63% yield, 86:14 dr, >99% ee).

$[\alpha]_D^{20}$ – 3.8 (*c* 0.5, acetone, 20°C). R_f = 0.55 (*n*-hexane/ EtOAc 4:1).

^{1}H NMR (400 MHz, $CDCl_3$) δ = 7.04 (t, *J* = 8.0 Hz, 1H, H-11), 6.89 (ddd, *J* = 8.0, 2.0, 0.9 Hz, 1H, H-10), 6.79 (t, *J* = 2.0 Hz, 1H, H-13), 6.56 (ddd, *J* = 8.0, 2.0, 0.9 Hz, 1H, H-12), 4.55 (d, *J* = 8.6 Hz, 1H, H-8), 4.44 (t, *J* = 6.7 Hz, 1H, H-3), 3.86 – 3.68 (m, 2H), 2.77 (ddtd, *J* = 8.4, 4.1, 3.1, 1.4 Hz, 1H, H-5), 2.09 – 2.00 (m, 2H), 1.96 (ddd, *J* = 13.0, 7.6, 2.9 Hz, 1H), 1.83 (dt, *J* = 13.0, 8.0 Hz, 1H), 1.77 – 1.57 (m, 5H), 1.48 – 1.31 (m, 3H), 1.26 – 1.04 (m, 4H), 0.94 (t, *J* = 7.4 Hz, 3H, H-7).

^{13}C NMR (100 MHz, $CDCl_3$) δ = 156.8 (C-2), 147.9 (C), 130.8 (CH), 123.5 (C-Br), 121.7 (CH), 120.4 (CN), 116.9 (CH), 112.8 (CH), 74.8 (C-1), 59.4, 52.0, 43.7, 34.8, 33.7, 33.7, 28.1, 25.6, 24.4, 24.3, 11.6 (C-7).

HRMS (ESI-Q-TOF) *m/z*: 388.1395 $[M+H]^+$; calcd. for $C_{20}H_{26}ClN_3$: 388.1383.

The enantiomeric ratio was determined by UPC^2 analysis using Trefoil CEL1, isocratic CO_2/EtOH = 80:20, flow rate = 1 mL/min, 35 °C, λ = 250 nm, t_R = 2.33 min (major) and t_R = 3.13 min (minor), > 99% ee.

(3*R*,5*R*)-3-((3-acetylphenyl)amino)-2-(cyclohexylamino)-5-ethylcyclopent-1-ene-1-carbonitrile (2y)

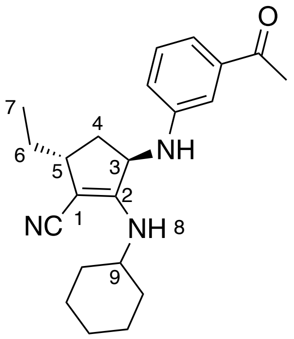

Prepared according the general procedure using hemiacetal **1a** (34 mg, 0.15 mmol), 3'-aminoacetophenone (20 mg, 0.15 mmol) and cyclohexyl isocyanide (19 µL, 0.15 mmol). Obtained after flash column chromatography purification (*n*-hexane/EtOAc 9:1) as a yellow oil (43 mg, 0.12 mmol, 81% yield, 97:3 dr, 96% ee). $[\alpha]_D^{20}$ – 7.9 (*c* 0.5, acetone, 20°C). R_f = 0.37 (*n*-hexane/ EtOAc 4:1).

^{1}H NMR (400 MHz, $CDCl_3$) δ = 7.40 – 7.20 (m, 3H), 6.91 – 6.81 (m, 1H), 4.58 (d, *J* = 8.7 Hz, 1H, H-8), 4.54 (t, *J* = 7.6 Hz, 1H, H-3), 3.88 – 3.70 (m, 1H, H-9), 2.78 (dq, *J* = 8.2, 4.2, 3.8 Hz, 1H), 2.57 (s, 3H, CH_3C=O), 2.10 – 1.95 (m, 3H), 1.84 (dt, *J* = 13.0, 7.9 Hz, 1H), 1.75 – 1.56 (m, 4H), 1.48 – 1.31 (m, 3H), 1.23 – 1.06 (m, 3H), 0.94 (t, *J* = 7.4 Hz, 3H, H-7).

^{13}C NMR (100 MHz, $CDCl_3$) δ = 198.4 (C=O), 157.0 (C-2), 147.0 (C), 138.5 (C), 129.8 (CH), 120.4 (CN), 119.4 (CH), 118.8 (CH), 113.02 (CH), 74.9 (C-1), 59.6, 52.1, 43.8, 34.9, 33.8, 33.7, 28.2, 26.8, 25.6, 24.4, 24.3, 11.6 (C-7).

HRMS (ESI-Q-TOF) *m/z*: 352.2394 $[M+H]^+$; calcd. for $C_{22}H_{29}N_3O$: 352.2383.

The enantiomeric ratio was determined by UPC^2 analysis using Trefoil AMY1, gradient CO_2/iPrOH = 100:0 to 80:20 in 6 min, flow rate = 1 mL/min, 35 °C, λ = 262 nm, t_R = 5.40 min (major) and t_R = 5.68 min (minor), 96% ee.

(3*R*,5*R*)-2-(cyclohexylamino)-5-ethyl-3-(piperidin-1-yl)cyclopent-1-ene-1-carbonitrile (2z)

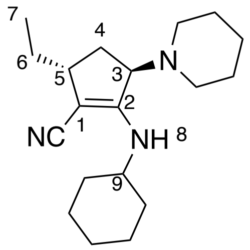

Prepared according the general procedure using hemiacetal **1a** (34 mg, 0.15 mmol), piperidine (15 µL, 0.15 mmol) and cyclohexyl isocyanide (19 µL, 0.15 mmol). Obtained after flash column chromatography purification (*n*-hexane/EtOAc 9:1) as a brown oil (30 mg, 67% yield, 78:22 dr, > 99% ee). $[\alpha]_D^{20}$ + 3.5 (*c* 0.5, acetone, 20°C). R_f= 0.57 (*n*-hexane/ EtOAc 4:1).

^{1}H NMR (400 MHz, $CDCl_3$) δ = 5.17 (d, *J* = 8.7 Hz, 1H, H-8), 3.81 (qt, *J* = 9.2, 3.8 Hz, 1H, H-10), 3.54 (t, *J* = 9.0 Hz, 1H, H-3), 2.54 (dq, *J* = 12.6, 4.5, 3.6 Hz, 1H, H-5), 2.35 (t, *J* = 5.2 Hz, 4H), 2.07 – 1.97 (m, 2H), 1.93 – 1.78 (m, 2H), 1.76 – 1.55 (m, 3H), 1.54 – 1.36 (m, 5H), 0.90 (t, *J* = 7.4 Hz, 3H, H-7).

^{13}C NMR (100 MHz, $CDCl_3$) δ = 157.6 (C-2), 121.0 (CN), 73.2 (C-1), 70.2, 51.2, 49.9 (C-10), 42.5, 33.9, 33.3, 28.0, 26.7, 25.8, 25.6, 24.6, 24.4, 24.2, 11.0 (C-7).

HRMS (ESI-Q-TOF) *m/z*: 302.2599 $[M+H]^+$; calcd. for $C_{19}H_{31}N_3$: 302.2591.

The enantiomeric ratio was determined by UPC^2 analysis using Trefoil CEL1, gradient CO_2/MeOH = 100:0 to 60:40 in 6 min, flow rate = 1 mL/min, 35 °C, λ = 210 nm, t_R = 2.59 min (major) and t_R = 2.68 min (minor), > 99% ee.

(3*R*,5*R*)-2-(cyclohexylamino)-5-ethyl-3-(methyl(phenyl)amino)cyclopent-1-ene-1-carbonitrile (2aa)

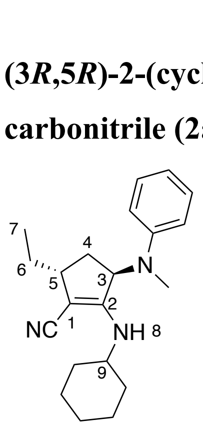

Prepared according the general procedure using hemiacetal **1a** (34 mg, 0.15 mmol), *N*-methylaniline (16 µL, 0.15 mmol) and cyclohexyl isocyanide (19 µL, 0.15 mmol). Obtained after flash column chromatography purification (*n*-hexane/EtOAc 9:1) as a dark yellow oil (43 mg, 0.13 mmol, 89% yield, 58:42 dr, 96% ee). $[\alpha]_D^{20}$ + 1.1 (*c* 0.5, acetone, 20°C). R_f= 0.70 (*n*-hexane/ EtOAc 4:1).

^{1}**H NMR** (400 MHz, $CDCl_3$) δ = 7.33 – 7.19 (m, 2H), 6.92 – 6.77 (m, 3H), 4.76 (t, *J* = 9.1 Hz, 1H, H-3), 4.57 (d, *J* = 10.1 Hz, 1H, H-8), 3.85 (ddt, *J* = 13.9, 9.1, 5.1 Hz, 1H), 2.69 (s, 3H, NCH_3), 2.29 – 1.03 (m, 14H), 0.92 (t, *J* = 7.9 Hz, 3H, H-7).
^{13}**C NMR** (100 MHz, $CDCl_3$) δ = 156.6 (C-2), 149.9 (C), 129.5, 120.3 (CN),118.8, 114.5, 74.8 (C-1), 65.7, 51.9, 44.1, 42.2, 34.0, 33.8, 28.6, 28.0, 25.6, 24.6, 24.3, 10.9 (C-7). **HRMS** (ESI-Q-TOF) *m/z*: 324.2443 $[M+H]^+$; calcd. for $C_{21}H_{29}N_3$: 324.2434.
The enantiomeric ratio was determined by UPC^2 analysis using Trefoil CEL2, gradient CO_2/iPrOH = 100:0 to 70:30 in 10 min, flow rate = 1 mL/min, 35 °C, λ = 280 nm, t_R = 5.91 min (minor) and t_R = 6.02 min (major), 96% ee.

(3*R*,5*R*)-2,3-bis (cyclohexylamino)-5-methylcyclopent-1-ene-1-carbonitrile (2ab)

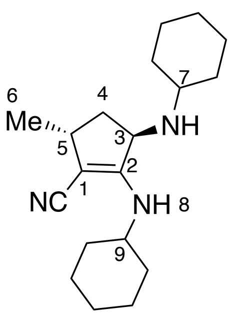

Prepared according the general procedure using hemiacetal **1b** (32 mg, 0.15 mmol), cyclohexylamine (17 µL, 0.15 mmol) and cyclohexyl isocyanide (19 µL, 0.15 mmol). Obtained after flash column chromatography purification (*n*-hexane/EtOAc 4:1) as a pale brown oil (32 mg, 0.11 mmol, 73% yield, 99:1 dr, > 99 % ee). $[\alpha]_D^{20}$ – 5.3 (*c* 0.5, acetone, 20°C). R_f= 0.55 (*n*-hexane/ EtOAc 7:3).
^{1}**H NMR** (400 MH, $CDCl_3$) δ = 5.10 (d, *J* = 8.7 Hz, 1H, H-8), 3.80 – 3.65 (m, 2H), 2.86 – 2.78 (m, 1H), 2.47 (tt, *J* = 10.2, 3.5 Hz, 1H), 2.08 – 1.99 (m, 2H), 1.94 (ddd, *J* = 12.3, 7.1, 1.4 Hz, 1H), 1.85 (d, *J* = 13.0 Hz, 1H), 1.75 – 1.66 (m, 5H), 1.65 – 1.52 (m, 2H), 1.48 – 1.36 (m, 2H), 1.29 – 1.10 (m, 9H), 1.08 (d, *J* = 6.9 Hz, 3H, H-6).
^{13}**C NMR** (100 MHz, $CDCl_3$) δ = 158.9 (C-2), 121.3 (CN), 73.1 (C-1), 60.0, 55.0, 51.5, 40.3, 36.8, 35.3, 33.8, 33.5, 26.1, 25.7, 25.1, 24.9, 24.5, 24.3, 20.8 (C-6).
HRMS (ESI-Q-TOF) *m/z*: 302.2589 $[M+H]^+$; calcd. for $C_{19}H_{32}N_3$: 302.2591.
The enantiomeric ratio was determined by UPC^2 analysis using Trefoil CEL1, gradient CO_2/iPrOH = 100:0 to 80:20 until 2 min then isocratic 80:20, flow rate = 1 mL/min, 25 °C, λ = 245 nm, t_R = 4.39 min (major) and t_R = 5.17 min (minor), > 99% ee.

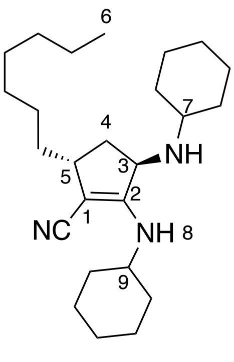

(3*R*,5*R*)-2,3-bis (cyclohexylamino)-5-heptylcyclopent-1-ene-1-carbonitrile (2ac)

Prepared according the general procedure using hemiacetal **1c** (45 mg, 0.15 mmol), cyclohexylamine (17 µL, 0.15 mmol) and cyclohexyl isocyanide (19 µL, 0.15 mmol). Obtained after flash

column chromatography purification (*n*-hexane/EtOAc 4:1) as a pale brown oil (42 mg, 0.15 mmol, 73% yield, 99:1 dr, >99% ee). $[\alpha]_D^{20}$ – 6.1 (*c* 0.5, acetone, 20°C). R_f = 0.55 (*n*-hexane/ EtOAc 7:3).

^{1}H NMR (400 MHz, $CDCl_3$) δ = 5.11 (d, *J* = 8.7 Hz, 1H, H-8), 3.82 – 3.70 (m, 1H, H-9), 3.66 (dd, *J* = 9.4, 7.1 Hz, 1H, H-3), 2.70 – 2.64 (m, 1H), 2.48 (tt, *J* = 10.5, 3.6 Hz, 1H), 2.09 – 1.99 (m, 2H), 1.85 (d, *J* = 12.4 Hz, 1H), 1.75 – 1.67 (m, 4H), 1.66 – 1.58 (m, 2H), 1.55 – 1.35 (m, 4H), 1.35 – 0.98 (m, 22H), 0.87 (t, *J* = 7.4 Hz, 3H, H-6).

^{13}C NMR (100 MHz, $CDCl_3$) δ = 159.3 (C-2), 121.7 (CN), 71.9 (C-1), 60.3, 55.0, 51.5, 42.2, 38.1, 35.5, 35.4, 33.8, 33.6, 32.0, 29.9, 29.5, 27.9, 26.1, 25.8, 25.2, 24.9, 24.5, 24.3, 22.8, 14.3 (C-6).

HRMS (ESI-Q-TOF) *m/z*: 386.3534 $[M+H]^+$; calcd. for $C_{25}H_{44}N_3$: 386.3530.

The enantiomeric ratio was determined by UPC^2 analysis using Trefoil CEL2, gradient CO_2/EtOH = 100:0 to 85:15 in 24 min, flow rate = 0.8 mL/min, 25 °C, λ = 254 nm, t_R = 15.52 min (major) and t_R = 15.28 min (minor), > 99% ee.

(3*R*,5*R*)-2,3-bis (cyclohexylamino)-5-((*Z*)-hex-3-en-1-yl)cyclopent-1-ene-1-carbonitrile (2ad)

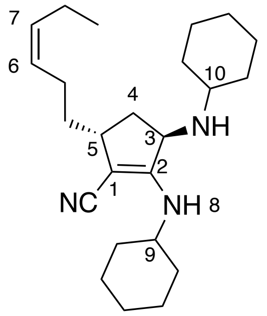

Prepared according the general procedure using hemiacetal **1d** (55 mg, 0.15 mmol), cyclohexylamine (17.2 µL, 0.15 mmol) and cyclohexyl isocyanide (18.7 µL, 0.15 mmol). Obtained after flash column chromatography purification (*n*-hexane/EtOAc 4:1) as a pale brown oil (39 mg, 0.10 mmol, 70% yield, 99:1 dr, > 99% ee). $[\alpha]_D^{20}$ – 5.7 (*c* 0.5, acetone, 20°C). R_f = 0.74 (*n*-hexane/ EtOAc 7:3).

^{1}H NMR (400 MHz, $CDCl_3$) δ = 5.42 – 5.25 (m, 2H, H-6, H-7), 5.12 (d, *J* = 8.7 Hz, 1H, H-8), 3.82 – 3.76 (m, 1H, H-9), 3.67 (dd, *J* = 9.4, 7.5 Hz, 1H, H-3), 2.74 – 2.68 (m, 1H), 2.52 – 2.44 (m, 1H), 2.13 – 1.98 (m, 7H), 1.85 (d, *J* = 12.8 Hz, 1H), 1.75 – 1.66 (m, 4H), 1.67 – 1.56 (m, 3H), 1.54 – 1.36 (m, 3H), 1.35 – 1.05 (m, 10H), 0.96 (t, *J* = 7.5 Hz, 3H).

^{13}C NMR (100 MHz, $CDCl_3$) δ = 159.4 (C-2), 132.1 (C-7, 128.8 (C-6), 121.5 (CN), 71.7 (C-1), 60.3, 55.0, 51.5, 41.8, 37.9, 35.4, 35.3, 33.8, 33.6, 26.1, 25.8, 25.5, 25.1, 24.9, 24.5, 24.3, 20.7, 14.5.

HRMS (ESI-Q-TOF) *m/z*: 370.3215 $[M+H]^+$; calcd. for $C_{24}H_{40}N_3$: 370.3217.

The enantiomeric ratio was determined by UPC[2] analysis using Trefoil CEL1, gradient CO_2/iPrOH = 100:0 to 77:23 until 2 min then isocratic 77:23, flow rate = 1 mL/min, 25 °C, λ = 256 nm, t_R = 4.62 min (major) and t_R = 5.63 min (minor), > 99% ee.

(3*R*,5*R*)-2,3-bis(cyclohexylamino)-5-((1,3-dioxoisoindolin-2-yl)methyl)cyclopent-1-ene-1-carbonitrile (2ae)

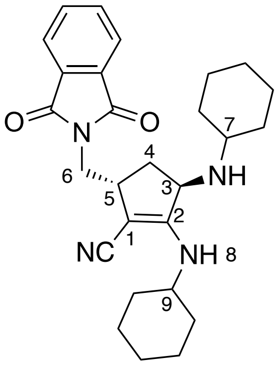

Prepared according the general procedure using hemiacetal **1e** (54 mg, 0.15 mmol), cyclohexylamine (17.2 µL, 0.15 mmol) and cyclohexyl isocyanide (18.7 µL, 0.15 mmol). Obtained after flash column chromatography purification (*n*-hexane/EtOAc 4:1) as a pale brown oil (40 mg, 0.09 mmol, 60% yield, 75:25 dr, 92% ee). $[\alpha]_D^{20}$ + 0.1 (*c* 0.5, acetone, 20°C). R_f= 0.32 (*n*-hexane/ EtOAc 7:3).

^{1}H NMR (400 MHz, $CDCl_3$) δ = 7.86 (dd, *J* = 5.5, 3.1 Hz, 2H), 7.78 – 7.61 (m, 2H), 5.35 (d, *J* = 8.6 Hz, 1H, H-8), 4.12 (q, *J* = 7.0 Hz, 1H), 3.88 – 3.70 (m, 2H, H-6), 3.62 (td, *J* = 13.1, 8.9 Hz, 1H, H-3), 3.23 (q, *J* = 7.8 Hz, 1H), 2.56 – 2.38 (m, 1H), 2.15 (dd, *J* = 12.6, 6.9 Hz, 1H), 2.09 – 1.94 (m, 2H), 1.87 (d, *J* = 13.6 Hz, 1H), 1.75 – 1.65 (m, 5H), 1.65 – 1.57 (m, 2H), 1.50 – 1.34 (m, 3H), 1.33 – 1.00 (m, 10H).

^{13}C NMR (100 MHz, $CDCl_3$) δ = 168.6 (C=O), 160.6 (C-2), 133.9, 132.0, 123.3, 120.5, 67.7 (C-1), 59.6, 54.8, 51.3, 43.1, 41.8, 40.8, 39.3, 36.0, 35.1, 33.5, 25.9, 25.6, 24.9, 24.7, 24.2, 24.1, 14.2

HRMS (ESI-Q-TOF) *m/z*: 447.2760 $[M+H]^+$; calcd. for $C_{27}H_{35}N_4O_2$: 447.2749.

The enantiomeric ratio was determined by UPC[2] analysis using Trefoil AMY1, isocratic CO_2/MeCN = 68:32, flow rate = 1 mL/min, 35 °C, λ = 217 nm, t_R = 3.46 min (major) and t_R = 4.86 min (major), 92% ee.

(3*R*,5*R*)-5-((benzyloxy)methyl)-2,3-bis(cyclohexylamino)cyclopent-1-ene-1-carbonitrile (2af)

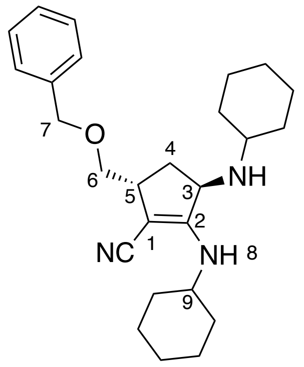

Prepared according the general procedure using hemiacetal **1f** (48 mg, 0.15 mmol), cyclohexylamine (17 μL, 0.15 mmol) and cyclohexyl isocyanide (19 μL, 0.15 mmol). Obtained after flash column chromatography purification (*n*-hexane/EtOAc 9:1) as a dark brown oil (28 mg, 0.07 mmol, 45% yield, 95:5 dr, 98% ee). $[\alpha]_D^{20}$ – 5.8 (*c* 0.5, acetone, 20°C). R_f = 0.41 (*n*-hexane/ EtOAc 4:1).

^{1}H NMR (400 MHz, $CDCl_3$) δ = 7.29 – 7.17 (m, 5H, Ph), 4.52 – 4.39 (m, 2H), 3.78 – 3.65 (m, 1H), 3.48 (dd, *J* = 9.4, 4.0 Hz, 1H), 3.24 (dd, *J* = 9.4, 7.6 Hz, 1H), 2.97 – 2.87 (m, 1H), 2.52 – 2.39 (m, 1H), 2.24 (dd, *J* = 12.8, 7.4 Hz, 1H), 1.97 (d, *J* = 12.4 Hz, 2H), 1.65 – 1.02 (m, 21H).

^{13}C NMR (100 MHz, $CDCl_3$) δ = 160.3 (C-2), 138.6, 128.5, 127.7, 127.7, 121.0 (CN), 73.7, 73.3, 72.7 (C-1), 67.7, 60.2, 55.0, 51.6, 42.6, 35.7, 35.1, 33.7, 33.5, 26.0, 25.7, 25.1, 24.9, 24.4, 24.3.

HRMS (ESI-Q-TOF) *m/z*: 408.3017 $[M+H]^+$; calcd. for $C_{26}H_{37}N_3O$: 408.3009.

The enantiomeric ratio was determined by UPC^2 analysis using Trefoil CEL2, gradient CO_2/MeOH = 90:10 to 60:40 in 6 min, flow rate = 1 mL/min, 35 °C, λ = 213 nm, t_R = 3.39 min (minor) and t_R = 3.57 min (major), 98% ee.

(3*R*,5*R*)-5-(((*tert*-butyldimethylsilyl)oxy)methyl)-2,3-bis(cyclohexylamino) cyclopent-1-ene-1-carbonitrile (2ag)

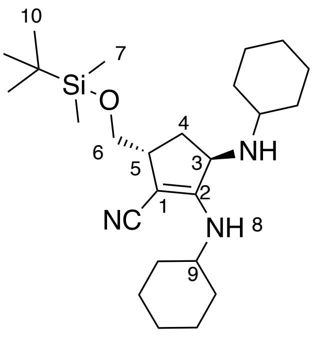

Prepared according the general procedure using hemiacetal **1g** (52 mg, 0.15 mmol), cyclohexylamine (17 μL, 0.15 mmol) and cyclohexyl isocyanide (19 μL, 0.15 mmol). Obtained after flash column chromatography purification (*n*-hexane/EtOAc 9:1) as a yellow oil (32 mg, 0.08 mmol, 50% yield, 99:1 dr, > 99% ee). $[\alpha]_D^{20}$ – 4.0 (*c* 0.5, acetone, 20°C). R_f = 0.62 (*n*-hexane/ EtOAc 4:1).

^{1}H NMR (400 MHz, $CDCl_3$) δ = 5.29 (brs, 1H, H-8), 3.85 – 3.70 (m, 2H), 3.63 (dd, *J* = 10.0, 3.6 Hz, 1H, H-6), 3.46 (dd, *J* = 10.1, 5.9 Hz, 1H, H-6), 2.84 – 2.75 (m, 1H), 2.54 – 2.43 (m, 1H), 2.28 (dd, *J* = 12.4, 7.2 Hz, 1H), 2.02 (dd, *J* = 11.6, 4.4 Hz, 2H), 1.83 (d, *J*

= 11.6 Hz, 1H), 1.76 – 1.65 (m, 6H), 1.65 – 1.55 (m, 2H), 1.50 – 1.34 (m, 3H), 1.31 – 1.04 (m, 8H), 0.87 (s, 9H, H-10), 0.04 (s, 6H, H-7).

^{13}C NMR (100 MHz, $CDCl_3$) δ = 160.8 (C-2), 121.2 (CN), 67.6 (C-1), 66.0, 65.5, 60.7, 55.1, 51.5, 45.0, 36.0, 35.2, 33.8, 33.7, 33.5, 26.1, 26.1 (C-10), 25.8, 25.2, 24.9, 24.4, 24.4, 18.4, -5.2 (C-7), -5.2 (C-7).

HRMS (ESI-Q-TOF) *m/z*: 432.3412 $[M+H]^+$; calcd. for $C_{25}H_{45}N_3OSi$: 432.3405.

The enantiomeric ratio was determined by UPC^2 analysis using Trefoil AMY1, gradient CO_2/MeOH = 100:0 to 60:40 in 6 min, flow rate = 1 mL/min, 35 °C, λ = 290 nm, t_R = 3.11 min (major) and t_R = 3.40 min (minor), > 99% ee.

(3*R*,5*R*)-2,3-bis(cyclohexylamino)-5-((prop-2-yn-1-yloxy)methyl)cyclopent-1-ene-1-carbonitrile (2ah)

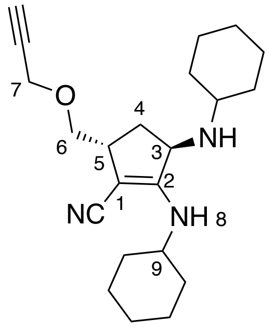

Prepared according the general procedure using hemiacetal **1h** (40 mg, 0.15 mmol), cyclohexylamine (17 µL, 0.15 mmol) and cyclohexyl isocyanide (19 µL, 0.15 mmol). Obtained after flash column chromatography purification (*n*-hexane/EtOAc 9:1) as a yellow oil (32 mg, 0.09 mmol, 60% yield, 78:22 dr, > 99% ee). $[\alpha]_D^{20}$ – 3.8 (*c* 0.5, acetone, 20°C). R_f = 0.34 (*n*-hexane/ EtOAc 4:1).

^{1}H NMR (400 MHz, $CDCl_3$) δ = 5.29 (d, *J* = 8.7 Hz, 1H, H-8), 4.15 (d, *J* = 2.5 Hz, 2H, H-7), 3.84 – 3.70 (m, 2H), 3.57 (dd, *J* = 9.3, 4.1 Hz, 1H), 3.32 (t, *J* = 8.7 Hz, 1H), 2.94 (td, *J* = 8.2, 4.0 Hz, 1H), 2.53 – 2.43 (m, 1H), 2.41 (t, *J* = 2.5 Hz, 1H), 2.33 (dd, *J* = 12.6, 7.2 Hz, 1H), 2.09 – 1.96 (m, 2H), 1.84 (d, *J* = 12.4 Hz, 1H), 1.76 – 1.61 (m, 6H), 1.51 – 1.33 (m, 4H), 1.25 – 1.01 (m, 9H).

^{13}C NMR (100 MHz, $CDCl_3$) δ = 160.8 (C-2), 120.9 (CN), 80.0, 74.4, 72.4, 67.1, 60.2, 58.5, 55.1, 51.5, 42.4, 35.9, 35.3, 33.8, 33.7, 33.5, 26.1, 25.7, 25.1, 24.9, 24.4, 24.3.

HRMS (ESI-Q-TOF) *m/z*: 356.2696 $[M+H]^+$; calcd. for $C_{22}H_{33}N_3O$: 356.2705.

The enantiomeric ratio was determined by UPC^2 analysis using Trefoil CEL1, gradient CO_2/iPrOH = 90:10 to 60:40 in 6 min, flow rate = 1 mL/min, 35 °C, λ = 220 nm, t_R = 3.76 min (minor) and t_R = 4.00 min (major), > 99% ee.

(3*R*,5*R*)-2-(*tert*-butylamino)-3-(cyclohexylamino)-5-ethylcyclopent-1-ene-1-carbonitrile (2ai)

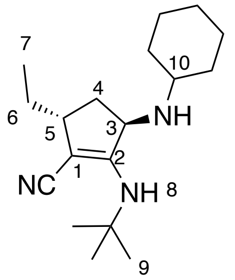

Prepared according the general procedure using hemiacetal **1a** (34 mg, 0.15 mmol), cyclohexylamine (17 μL, 0.15 mmol) and *tert*-butyl isocyanide (17 μL, 0.15 mmol). Obtained after flash column chromatography purification (*n*-hex/EtOAc 6:1) as a yellow oil (37 mg, 0.13 mmol, 85% yield, >99:1 dr, >99% ee). $[\alpha]_D^{20}$ – 12.8 (*c* 0.7, acetone, 20°C). R_f= 0.34 (*n*-hex/EtOAc 5:1).

^{1}H NMR (400 MHz, $CDCl_3$) δ = 5.67 (s, 1H, H-8), 3.60 (dd, *J* = 10.0, 7.1 Hz, 1H, H-3), 2.66 (td, *J* = 8.4, 4.6 Hz, 1H), 2.48 (tt, *J* = 10.2, 3.7 Hz, 1H), 2.05 (dd, *J* = 12.4, 7.1 Hz, 1H), 1.82 (d, *J* = 12.3 Hz, 1H), 1.77 – 1.66 (m, 3H), 1.64 – 1.54 (m, 2H), 1.40 (s, 9H, H-9), 1.36 – 0.95 (m, 8H), 0.91 (t, *J* = 7.4 Hz, 3H, H-7).

^{13}C NMR (100 MHz, $CDCl_3$) δ = 156.8 (C-2), 123.5 (CN), 71.4 (C-1), 62.0, 55.1, 51.3, 44.6, 36.7, 35.2, 33.8, 30.2 (C-9), 27.9, 26.0, 25.1, 24.8, 12.0 (C-7).

HRMS (ESI-Q-TOF) *m/z*: 290.2599 $[M+H]^+$; calcd. for $C_{18}H_{32}N_3$: 290.2596.

The enantiomeric ratio was determined by UPC^2 analysis using Trefoil CEL2, isocratic CO_2/MeCN = 95:5, flow rate = 1 mL/min, 25 °C, λ = 265 nm, t_R = 5.84 min (major) and t_R = 5.99 min (minor), >99% ee.

(3*R*,5*R*)-3-(cyclohexylamino)-5-ethyl-2-(octadecylamino)cyclopent-1-ene-1-carbonitrile (2aj)

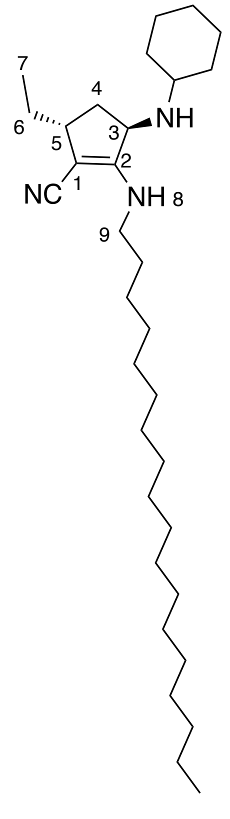

Prepared according the general procedure using hemiacetal **1a** (34 mg, 0.15 mmol), cyclohexylamine (17 μL, 0.15 mmol) and octadecyl isocyanide (42 mg, 0.15 mmol). Obtained after flash chromatography purification (*n*-hexane/EtOAc 20:1) as a brown solid (60 mg, 82% yield, >99:1 dr, > 99% ee). m.p. 40-42 °C. $[\alpha]_D^{20}$ – 41.8 (*c* 0.5, acetone, 20°C). R_f= 0.60 (*n*-hex/EtOAc 4:1).

^{1}H NMR (400 MHz, $CDCl_3$) δ = 5.18 (s, 1H, H-8), 3.69 (t, *J* = 8.3 Hz, 1H, H-3), 3.52 – 3.37 (m, 2H, H-9), 2.65 (td, *J* = 8.4, 5.5 Hz, 1H), 2.50 (td, *J* = 10.3, 8.6, 5.0 Hz, 1H), 2.06 (dd, *J* = 12.6, 7.3 Hz, 1H), 1.85 (d, *J* = 12.0 Hz, 1H), 1.76 – 1.68 (m, 5H), 1.63 – 1.46 (m,

5H), 1.39 – 1.15 (m, 36H), 0.92 (t, J = 7.4 Hz, 3H, H-7), 0.87 (t, J = 6.6 Hz, 3H).

^{13}C NMR (100 MHz, $CDCl_3$) δ = 160.3 (C-2), 121.5 (CN), 72.4 (C-1), 60.3, 55.1, 43.9, 43.7, 37.5, 35.2, 33.6, 32.1, 30.0, 29.8, 29.7, 29.7, 29.5, 28.1 (CH_2), 26.8 (CH_2), 26.1, 25.1, 24.9, 22.8 (CH_2), 14.2 (CH_3), 11.9 (C-7).

HRMS (ESI-Q-TOF) m/z: 486.4796 $[M+H]^+$, calcd. for $C_{32}H_{60}N_3$: 486.4782.

The enantiomeric ratio was determined by UPC^2 analysis using Trefoil AMY1, gradient CO_2/iPrOH = 100:0 to 60:40 until 5 min then isocratic 60:40, flow rate = 1 mL/min, 35 °C, λ = 286 nm, t_R = 5.09 min (major) and t_R = 5.47 min (minor), > 99% ee.

Ethyl ((3*R*,5*R*)-2-cyano-5-(cyclohexylamino)-3-ethylcyclopent-1-en-1-yl)glycinate (2ak)

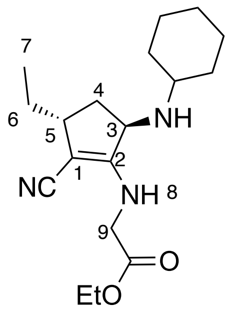

Prepared according the general procedure using hemiacetal **1a** (34 mg, 0.15 mmol), cyclohexylamine (17 µL, 0.15 mmol) and ethyl isocyanoacetate (16 µL, 0.15 mmol). Obtained after flash column chromatography purification (*n*-hexane/EtOAc 4:1) as a yellow oil (22 mg, 0.11 mmol, 72% yield, 92:8 dr, 97% ee). $[\alpha]_D^{20}$ – 9.9 (*c* 0.5, acetone, 20°C). R_f = 0.37 (*n*-hexane/ EtOAc 2:1).

^{1}H NMR (400 MHz, $CDCl_3$) δ = 5.76 (s, 1H), 4.31 – 4.19 (m, 4H), 3.78 (t, J = 8.1 Hz, 1H, H-3), 2.67 (q, J = 7.8 Hz, 1H), 2.52 (tt, J = 10.2, 3.7 Hz, 1H), 2.07 (dd, J = 12.6, 7.4 Hz, 1H), 1.87 (d, J = 12.3 Hz, 1H), 1.79 – 1.67 (m, 3H), 1.63 – 1.47 (m, 4H), 1.29 (t, J = 7.1 Hz, 3H), 1.22 – 1.00 (m, 4H), 0.92 (t, J = 7.3 Hz, 3H, H-7).

^{13}C NMR (100 MHz, $CDCl_3$) δ = 170.1 (C=O), 160.1 (C-1), 120.6 (CN), 75.0 (C-2), 61.8, 60.3, 55.2, 45.2, 43.7, 37.6, 35.1, 33.5, 27.9, 26.0, 25.1, 24.8, 14.3, 11.8 (C-7).

HRMS (ESI-Q-TOF) m/z: 320.2339 $[M+H]^+$; calcd. for $C_{18}H_{30}N_3O_2$: 320.2338.

The enantiomeric ratio was determined by UPC^2 analysis using Trefoil CEL2, gradient CO_2/MeOH = 100:60 in 5 min, flow rate = 1 mL/min, 25 °C, λ = 265 nm, t_R = 4.03 min (minor) and t_R = 4.23 min (major), 97% ee.

(3*R*,5*R*)-2-(benzylamino)-3-(cyclohexylamino)-5-ethylcyclopent-1-ene-1-carbonitrile (2al)

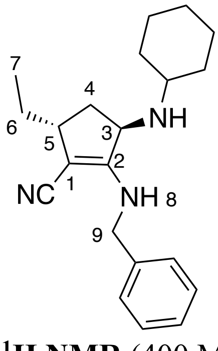

Prepared according the general procedure using hemiacetal **1a** (34 mg, 0.15 mmol), cyclohexylamine (17 μL, 0.15 mmol) and benzyl isocyanide (19 μL, 0.15 mmol). Obtained after flash chromatography purification (*n*-hexane/EtOAc 20:1) as a brown solid (38 mg, 0.12 mmol, 78% yield, 87:13 dr, > 99% ee). $[\alpha]_D^{20}$ – 57.4 (*c* 0.5, acetone, 20°C). R_f = 0.40 (*n*-hex/EtOAc 4:1).

^{1}H NMR (400 MHz, $CDCl_3$) δ = 7.40 – 7.27 (m, 5H), 5.42 (brs, 1H), 4.69 (qd, *J* = 14.5, 5.9 Hz, 2H), 3.75 (t, *J* = 8.2 Hz, 1H), 2.69 (dd, *J* = 13.2, 8.0 Hz, 1H), 2.52 – 2.42 (m, 1H), 2.09 (dd, *J* = 12.6, 7.3 Hz, 1H), 1.83 (d, *J* = 13.0 Hz, 1H), 1.71 (d, *J* = 9.5 Hz, 3H), 1.64 – 1.49 (m, 3H), 1.37 – 1.03 (m, 7H), 0.94 (t, *J* = 7.4 Hz, 3H).

^{13}C NMR (100 MHz, $CDCl_3$) δ = 160.6 (C-2), 138.4, 128.9, 127.8, 127.7, 121.2 (CN), 73.5 (C-1), 60.3, 55.2, 43.7, 37.9, 35.3, 33.6, 26.1, 25.2, 24.9, 11.9 (C-7).

HRMS (ESI-Q-TOF) m/z: 324.2433 $[M+H]^+$, calcd. for $C_{21}H_{30}N_3$: 324.2434.

The enantiomeric ratio was determined by UPC^2 analysis using Trefoil CEL1, isocratic CO_2/MeOH = 85:15, flow rate = 1 mL/min, 35 °C, λ = 286 nm, t_R = 1.67 min (minor) and t_R = 1.80 min (major), > 99% ee.

(3*R*,5*R*)-3-(cyclohexylamino)-5-ethyl-2-((4-methoxyphenyl)amino)cyclopent-1-enecarbonitrile (2am)

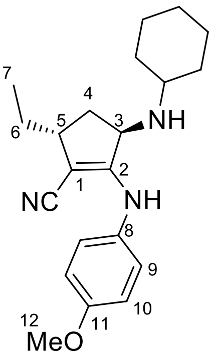

Prepared according the general procedure using hemiacetal **1a** (34 mg, 0.15 mmol), cyclohexylamine (17 μL, 0.15 mmol) and *p*-methoxyphenyl isocyanide (20 mg, 0.15 mmol). Obtained after flash chromatography purification (*n*-hexane/EtOAc 4:1) as an orange oil (25 mg, 0.08 mmol, 51% yield, 95:5 dr, > 99% ee). $[\alpha]_D^{20}$ – 8.5 (*c* 0.5, acetone, 20°C). R_f = 0.36 (*n*-hex/EtOAc 7:3).

^{1}H NMR (400 MHz, $CDCl_3$) δ = 7.13 (d, *J* = 8.8 Hz, 2H, H-9), 6.92 – 6.82 (m, 3H, H-10 and NH), 3.89 (t, *J* = 8.1 Hz, 1H, H-3), 3.79 (s, 3H, H-12), 2.71 (td, *J* = 8.2, 5.2 Hz, 1H), 2.59 – 2.49 (m, 1H), 2.12 (dd, *J* = 12.7, 7.2 Hz, 1H), 1.88 (d, *J* = 12.8 Hz, 1H), 1.83 – 1.68 (m, 1H), 1.66 – 1.53 (m, 3H), 1.41 – 1.01 (m, 7H), 0.95 (t, *J* = 7.4, 3H, H-7).

^{13}C NMR (100 MHz, $CDCl_3$) δ = 157.4 (C-2), 157.3 (C-11), 131.7 (C-8), 125.4 (C-9), 119.1 (CN), 114.0 (C-10), 76.5 (C-1), 60.7, 55.5, 55.3, 44.1, 37.4, 35.2, 33.6, 28.0, 26.0, 25.1, 24.9, 11.9 (C-7).

HRMS (ESI-Q-TOF) m/z: 340.2308 $[M+H]^+$, calcd. for $C_{21}H_{30}N_3O$: 340.2383.

The enantiomeric ratio was determined by UPC^2 analysis using Trefoil CEL1, isocratic CO_2/MeOH = 90:10, flow rate = 1 mL/min, 35 °C, λ = 286 nm, t_R = 3.44 min (minor) and t_R = 3.78 min (major), > 99% ee.

(3*R*,5*R*)-3-(cyclohexylamino)-5-ethyl-2-(((tetrahydrofuran-2-yl)methyl)amino) cyclopent-1-ene-1-carbonitrile (2an)

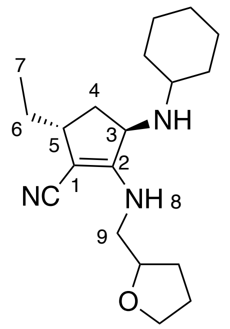

Prepared according the general procedure using hemiacetal **1a** (34 mg, 0.15 mmol), cyclohexylamine (17 μL, 0.15 mmol) and 2-(isocyanomethyl)tetrahydrofuran (17 μL, 0.15 mmol). Obtained after flash chromatography purification (*n*-hexane/EtOAc 4:1) as a brown oil (41 mg, 0.13 mmol, 86% yield, >99:1 dr, > 99% ee). $[\alpha]_D^{20}$ – 48.0 (*c* 0.5, acetone, 20°C). R_f= 0.17 (*n*-hex/EtOAc 4:1).

^{1}H NMR (400 MHz, $CDCl_3$) δ = 5.54 (d, *J* = 18.5 Hz, 1H), 4.11 (qd, *J* = 7.4, 3.0 Hz, 1H), 3.91 – 3.82 (m, 1H), 3.82 – 3.66 (m, 3H), 3.48 – 3.32 (m, 1H), 2.70 – 2.60 (m, 1H), 2.49 (tt, *J* = 10.3, 3.7 Hz, 1H), 2.13 – 2.00 (m, 2H), 1.95 – 1.80 (m, 3H), 1.77 – 1.65 (m, 3H), 1.63 – 1.45 (m, 4H), 1.35 – 0.97 (m, 7H), 0.92 (t, *J* = 7.4 Hz, 3H, H-7).

^{13}C NMR (100 MHz, $CDCl_3$) δ = 160.9 (C-2), 121.6 (CN), 77.7 (C-1), 68.5, 60.2, 55.1, 47.7, 43.6, 37.7, 35.1, 33.5, 28.8, 28.7, 28.1, 26.1, 26.0, 25.1, 24.9, 11.9 (C-7).

HRMS (ESI-Q-TOF) m/z: 318.2538 $[M+H]^+$, calcd. for $C_{19}H_{32}N_3O$: 318.2540.

The enantiomeric ratio was determined by UPC^2 analysis using Trefoil AMY1, isocratic CO_2/iPrOH = 70:30, flow rate = 1 mL/min, 35 °C, λ = 267 nm, t_R = 1.41 min (major) and t_R = 1.62 min (minor), > 99% ee.

(2*S*,3*S*,4*S*,5*S*,6*S*)-2-(acetoxymethyl)-6-(((3*R*,5*R*)-2-cyano-3-ethyl-5-(((*S*)-1-methoxy-1-oxopropan-2-yl)amino)cyclopent-1-en-1-yl)amino)tetrahydro-2*H*-pyran-3,4,5-triyl triacetate (2ao)

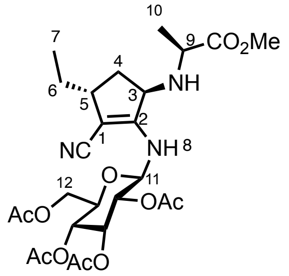

Prepared according the general procedure using hemiacetal **1a** (34 mg, 0.15 mmol), HCl.NH$_2$-Ala-OMe (21 mg, 0.15 mmol), triethylamine (23 µL, 0.16 mmol), and β-D-glucosyl isocyanide (54 mg, 0.15 mmol). Obtained after flash column chromatography purification (*n*-hexane/EtOAc 2:1) as a pale yellow oil (53 mg, 0.09 mmol, 63% yield, 99:1 dr).$[\alpha]_D^{20}$ –14.4 (*c* 0.6, acetone, 20°C). R_f = 0.40 (*n*-hexane/ EtOAc 1:1).

^{1}H NMR (400 MHz, $CDCl_3$) δ = 6.62 (brs, 1H, H-8), 5.30 (t, *J* = 9.3 Hz, 1H), 5.23 (t, *J* = 9.0 Hz, 1H), 5.15 (t, *J* = 9.7 Hz, 1H), 5.05 (t, *J* = 9.3 Hz, 1H), 4.30 (dd, *J* = 12.4, 4.2 Hz, 1H, H-12b), 4.19 (d, *J* = 12.0 Hz, 1H, H-12a), 4.03 – 3.87 (m, 2H), 3.76 (s, 3H, OMe), 3.55 (q, *J* = 7.1 Hz, 1H, H-9), 2.73 (brs, 1H), 2.08 (d, *J* = 5.6 Hz, 6H), 2.02 (d, *J* = 5.5 Hz, 6H), 1.94 – 1.86 (m, 1H), 1.80 (d, *J* = 6.7 Hz, 1H), 1.74 – 1.60 (m, 1H), 1.43 (d, *J* = 7.0 Hz, 3H, H-10), 1.36 – 1.20 (m, 1H), 1.13 – 0.97 (m, 1H), 0.91 (t, *J* = 7.3 Hz, 3H, H-7).

^{13}C NMR (100 MHz, $CDCl_3$) δ = 170.8 (C=O), 170.8 (C=O), 170.3 (C=O), 170.0 (C=O), 169.7 (C=O), 158.6 (C-2), 118.0 (CN), 81.9 (C-11), 73.5, 72.0, 71.0, 68.3, 68.2, 62.0, 61.8, 53.2, 45.1, 33.3, 28.1, 20.9, 20.8, 20.7, 18.1, 11.4 (C-7).

HRMS (ESI-Q-TOF) *m/z*: 568.2509 $[M+H]^+$; calcd. for $C_{26}H_{38}N_3O_{11}$: 568.2506.

Methyl ((1*R*,4*R*)-3-cyano-4-ethyl-2-((2-(((*S*)-1-methoxy-1-oxo-3-phenylpropan-2-yl)amino)-2-oxoethyl)amino)cyclopent-2-en-1-yl)-*L*-leucinate (2ap)

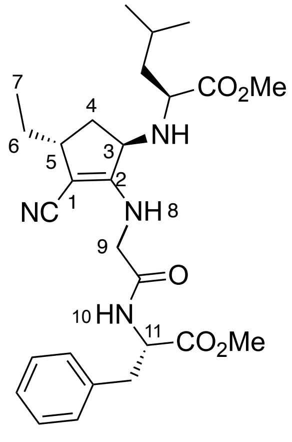

Prepared according the general procedure using hemiacetal **1a** (34 mg, 0.15 mmol), HCl.NH$_2$-Leu-OMe (27 mg, 0.15 mmol), triethylamine (23 µL, 0.16 mmol) and CN-Gly-Phe-OMe (37 mg, 0.15 mmol). Obtained after flash column chromatography purification (*n*-hexane/EtOAc 1:1) as a yellow oil (55 mg, 0.11 mmol, 73% yield, >99:1 dr).$[\alpha]_D^{20}$ – 19.8 (*c* 0.6, acetone, 20°C). R_f= 0.33 (*n*-hexane/ EtOAc 1:1).

^{1}H NMR (400 MHz, $CDCl_3$) δ = 7.32 – 7.26 (m, 3H, Ph), 7.11 (d, *J* = 7.0 Hz, 2H, Ph), 6.57 (d, *J* = 7.9 Hz, 1H, H-10), 6.12 (brs, 1H, H-8), 4.95 (dt, *J* =

8.1, 5.9 Hz, 1H, H-11), 4.20 (t, J = 6.0 Hz, 2H, H-9), 3.73 (s, 3H, OMe), 3.71 (s, 3H, OMe), 3.64 (t, J = 7.2 Hz, 1H), 3.35 (dd, J = 8.8, 5.4 Hz, 1H), 3.14 (t, J = 5.6 Hz, 2H), 2.72 (hept, J = 4.0 Hz, 1H), 1.89 (brs, 1H), 1.83 (ddd, J = 12.9, 7.5, 3.4 Hz, 1H), 1.74 – 1.57 (m, 2H), 1.52 – 1.44 (m, 1H), 1.43 – 1.35 (m, 1H), 1.31 – 1.19 (m, 2H), 0.92 – 0.86 (m, 9H).

^{13}C NMR (100 MHz, $CDCl_3$) δ = 176.4 (C=O), 171.8 (C=O), 168.3 (C=O), 159.1 (C-2), 135.8, 129.4, 128.8, 127.3, 120.3 (CN), 77.4 (C-1), 62.0, 57.6, 53.4, 52.5, 52.4, 46.6, 43.9, 42.3, 38.0, 35.8, 27.8, 24.9, 23.0, 21.8, 11.6 (C-7).

HRMS (ESI-Q-TOF) *m/z*: 499.2925 $[M+H]^+$; calcd. for $C_{27}H_{39}N_4O_5$: 499.2920.

Methyl ((1*R*,4*R*)-3-cyano-4-ethyl-2-((2-(((*S*)-1-methoxy-1-oxo-3-phenylpropan-2-yl)amino)-2-oxoethyl)amino)cyclopent-2-en-1-yl)-*L*-phenylalanyl-*L*-isoleucinate (2aq)

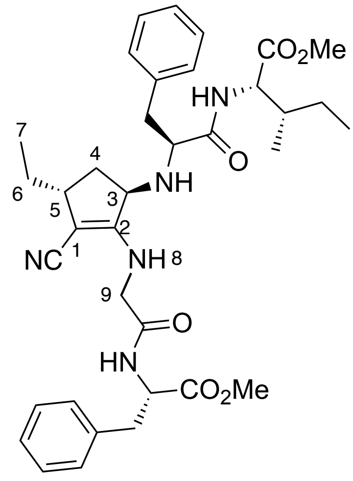

Prepared according the general procedure using hemiacetal **1a** (34 mg, 0.15 mmol), HCl Phe-Ile- OMe (33 mg, 0.15 mmol), triethylamine (23 μL, 0.16 mmol) and CN-Gly-Phe-OMe (37 mg, 0.15 mmol). Obtained after flash column chromatography purification (DCM/MeOH 15:1) as a light yellow oil (64 mg, 0.10 mmol, 68% yield, 96:4 dr). $[\alpha]_D^{20}$ – 7.7 (c 0.7, acetone, 20°C). R_f = 0.40 (DCM/MeOH 10:1).

^{1}H NMR (400 MHz, $CDCl_3$) δ = 7.91 (d, J = 9.1 Hz, 1H, NH), 7.36 – 7.27 (m, 5H, Ph), 7.26 – 7.17 (m, 3H, Ph), 7.13 (d, J = 7.1 Hz, 2H, Ph), 6.64 (d, J = 7.9 Hz, 1H, NH), 6.28 (brs, 1H, H-8), 4.93 (dt, J = 8.2, 5.9 Hz, 1H), 4.58 (dd, J = 9.2, 4.6 Hz, 1H), 4.24 (dd, J = 17.2, 6.2 Hz, 1H), 4.09 (dd, J = 17.3, 5.8, 1H), 3.70 (s, 3H, OMe), 3.69 (s, 3H, OMe), 3.27 (dd, J = 13.7, 4.3 Hz, 1H), 3.20 – 3.06 (m, 3H), 2.73 (s, 1H), 2.33 (s, 1H), 2.00 – 1.88 (m, 1H), 1.65 – 1.48 (m, 2H), 1.47 – 1.36 (m, 1H), 1.38 – 1.26 (m, 1H), 1.27 – 1.19 (m, 1H), 1.17 – 1.04 (m, 1H), 0.96 – 0.88 (m, 6H), 0.87 – 0.80 (m, 1H), 0.76 (t, J = 7.4 Hz, 3H). **^{13}C NMR** (100 MHz, $CDCl_3$) δ = 173.3 (C=O), 173.2 (C=O), 171.9 (C=O), 168.7 (C=O), 162.8 (C-2), 136.3, 135.8, 129.4, 129.3, 129.1, 128.8, 127.6, 127.3, 119.3 (CN), 77.4 (C-1), 62.7, 62.6, 56.5, 53.5, 52.6, 52.5, 46.8, 43.6, 39.4, 38.1, 37.9, 34.2, 27.5, 25.2, 15.8, 11.7, 11.1.

HRMS (ESI-Q-TOF) *m/z*: 646.3609 $[M+H]^+$; calcd. for $C_{36}H_{48}N_5O_6$: 646.3605.

Methyl ((1*R*,4*R*)-3-cyano-4-ethyl-2-((2-(((*S*)-1-methoxy-1-oxo-3-phenylpropan-2-yl)amino)-2-oxoethyl)amino)cyclopent-2-en-1-yl)-*L*-phenylalanyl-*L*-leucyl-*L*-valinate (2ar)

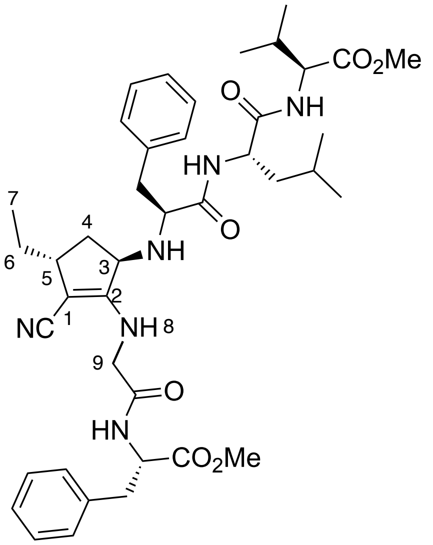

Prepared according the general procedure using hemiacetal **1a** (34 mg, 0.15 mmol), HCl.NH_2-Phe-Leu-Val-OMe (64 mg, 0.15 mmol), triethylamine (23 µL, 0.16 mmol), and CN-Gly-Phe-OMe (37 mg, 0.15 mmol). Obtained after flash column chromatography purification (DCM/MeOH 10:1) as a pale yellow oil (56 mg, 0.08 mmol, 50% yield, 97:3 dr). $[\alpha]_D^{20}$ – 5.2 (*c* 0.5, acetone, 20°C). R_f = 0.17 (DCM/MeOH 99:1).

^{1}H NMR (600 MHz, $CDCl_3$) δ = 7.33 – 7.30 (m, 2H), 7.26 – 7.20 (m, 6H), 7.15 – 7.13 (m, 2H), 6.92 (s, 1H), 6.54 (d, *J* = 8.8 Hz, 1H), 4.92 (dt, *J*=7.9, 6.0, 1H), 4.57 (q, *J* = 8.4 Hz, 1H), 4.50 (dd, *J* = 8.8, 4.9 Hz, 1H), 4.17 (dd, *J* = 19.1, 5.2, 1H), 4.05 (dd, *J* = 17.1, 5.9 Hz, 1H), 3.74 (s, 3H, OMe), 3.70 (s, 3H, OMe), 3.23 (dd, *J* = 13.7, 3.8 Hz, 1H), 3.14 (d, *J* = 6.0 Hz, 2H), 2.95 (s, 1H), 2.88 (s, 1H), 2.65 – 2.47 (m, 1H), 2.25 – 2.08 (m, 3H), 1.75 – 1.54 (m, 6H), 0.97 (d, *J* = 6.5 Hz, 6H), 0.95 – 0.91 (m, 3H), 0.88 (d, *J* = 6.9 Hz, 7H), 0.71 (t, *J* = 7.4 Hz, 3H, H-7).

^{13}C NMR (150 MHz, $CDCl_3$) δ = 173.1 (C=O), 172.2 (C=O), 172.0 (C=O), 168.8 (C=O), 162.7 (C-1), 136.9, 136.1, 129.4, 129.2, 129.1, 128.7, 127.5, 127.2, 119.3 (CN), 63.2 (C-2), 62.9, 57.4, 53.5, 52.4, 52.4, 51.5, 46.9, 43.3, 41.7, 39.6, 38.2, 34.8, 31.4, 27.4, 25.0, 23.1, 22.0, 19.0, 17.8, 11.1 (C-7).

HRMS (ESI-Q-TOF) *m/z*: 745.4286 $[M+H]^+$; calcd. for $C_{41}H_{57}N_6O_7$: 745.4289.

Methyl ((1*R*,4*R*)-3-cyano-2-((2-(((*S*)-1-(((*S*)-1-methoxy-1-oxo-3-phenylpropan-2-yl)amino)-4-methyl-1-oxopentan-2-yl)amino)-2-oxoethyl)amino)-4-((prop-2-yn-1-yloxy)methyl)cyclopent-2-en-1-yl)-*L*-phenylalanyl-*L*-leucyl-*L*-valinate (2as)

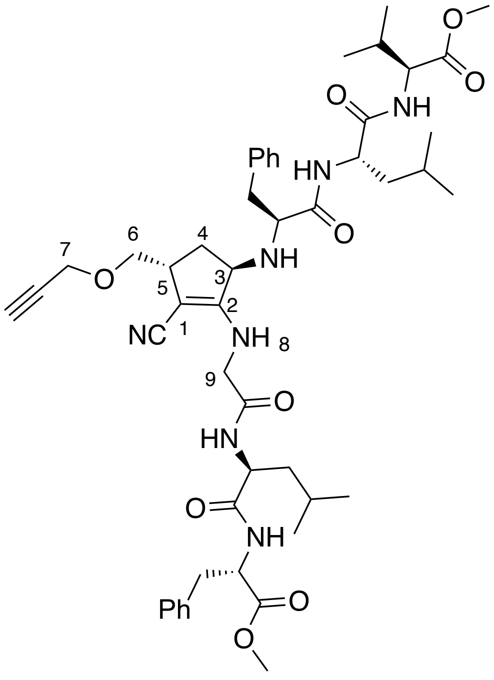

Prepared according the general procedure using hemiacetal **1a** (34 mg, 0.15 mmol), TFA.NH_2-Phe-Leu-Val-OMe (76 mg, 0.15 mmol) and CN-Gly-Leu-Phe-OMe (54 mg, 0.15 mmol). Obtained after flash chromatography purification as a brown oil (70 mg, 0.08 mmol, 52% yield, 99:1 dr). $[\alpha]_D^{20}$ – 5.0 (*c* 0.5, acetone, 20°C). R_f = 0.47 (DCM/MeOH 9:1).

^{1}H NMR (600 MHz, $CDCl_3$) δ = 7.80 (d, *J* = 9.0, 1H, NH), 7.33 (t, *J* = 7.4, 2H, Ph), 7.31 – 7.21 (m, 6H, Ph), 7.13 (d, *J* = 7.2, 2H, Ph), 6.88 (brs, 1H, NH), 6.59 (d, *J* = 7.8, 1H, NH), 6.51 (d, *J* = 8.8, 1H, NH), 6.22 (brs, 1H, NH), 4.82 (dt, *J* = 7.9, 6.1, 1H), 4.58 (q, *J* = 8.4, 1H), 4.49 (dd, *J* = 8.8, 4.9 Hz, 2H, H-9), 4.16 (dd, *J* = 17.0, 6.1, 1H), 4.06 (d, *J* = 2.3, 1.6, 2H, H-7), 4.00 (dd, *J* = 17.2, 5.9, 1H), 3.73 (s, 3H, OMe), 3.70 (s, 3H, OMe), 3.47 – 3.40 (m, 2H), 3.38 (dd, *J* = 10.4, 3.8, 1H), 3.27 (dd, *J* = 9.3, 6.7, 1H), 3.21 (dd, *J* = 13.7, 3.7, 1H), 3.12 (dd, *J* = 6.2, 4.3, 2H), 2.62 (s, 3H), 2.39 (t, *J* = 2.3, 1H, H-10), 2.17 (pd, *J* = 6.9, 4.8, 1H), 1.86 – 1.79 (m, 2H), 1.69 – 1.59 (m, 6H), 1.56 – 1.48 (m, 2H), 0.97 (d, *J* = 3.8, 3H), 0.96 (d, *J* = 3.9, 3H), 0.92 – 0.88 (m, 12H).

^{13}C NMR (150 MHz, $CDCl_3$) δ = 173.9 (C=O), 173.0 (C=O), 172.1 (C=O), 171.8 (C=O), 171.6 (C=O), 169.0 (C=O), 160.2 (C-2), 137.0, 135.9, 129.4, 129.3, 129.1, 128.8, 127.5, 127.3, 119.0 (CN), 79.7 (C-1), 74.7, 71.7, 63.3, 62.8, 58.5, 57.4, 53.5, 52.5, 52.4, 51.8, 51.4, 46.9, 42.1, 41.7, 41.4, 41.1, 39.9, 37.8, 33.3, 31.4, 25.0, 24.8, 23.1, 23.0, 22.3, 22.1, 19.1, 17.8.

HRMS (ESI-Q-TOF) m/z: 898.5079 $[M+H]^+$, calcd. for $C_{49}H_{68}N_7O_9$: 898.5073.

General reaction procedure for the synthesis of cyclopentenols 3a-d

The hemiacetal **1** (0.15 mmol, 1 equiv.) was dissolved in trifluoroethanol (0.3 mL), the isocyanide (0.15 mmol, 1.0 equiv.) was introduced into this solution and the glass tube was sealed. The flask was irradiated for 20 min (300 W) under high-speed magnetic stirring in the microwave reactor, while the temperature was raised up to 70 °C. The volatiles was concentrated under reduced pressure and the resulting crude product was purified by flash column chromatography.

(3*R*,5*R*)-2-(cyclohexylamino)-5-ethyl-3-hydroxycyclopent-1-ene-1-carbonitrile (3a)

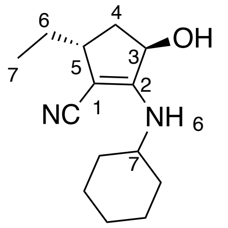

Prepared according the general procedure using hemiacetal **1a** (34 mg, 0.15 mmol) and cyclohexyl isocyanide (19 µL, 0.15 mmol). Obtained after flash column chromatography purification (*n*-hexane/EtOAc 4:1) as a pale brown oil (29 mg, 0.12 mmol, 83% yield, 99:1 dr, 98% ee). $[\alpha]_D^{20}$ – 1.1 (*c* 0.5, acetone, 20°C). R_f = 0.32 (*n*-hexane/ EtOAc 7:3).

^{1}H NMR (400 MHz, $CDCl_3$) δ = 4.62 (t, *J* = 7.2 Hz, 1H, H-3), 4.56 (d, *J* = 8.6 Hz, 1H, H-6), 3.83 – 3.64 (m, 1H), 2.86 – 2.69 (m, 1H), 2.13 – 1.95 (m, 2H), 1.89 – 1.67 (m, 4H), 1.68 – 1.58 (m, 2H), 1.48 – 1.36 (m, 2H), 1.34 – 1.22 (m, 1H), 1.22 – 1.09 (m, 3H), 0.91 (t, *J* = 7.4 Hz, 3H, H-7).

^{13}C NMR (100 MHz, $CDCl_3$) δ = 157.9 (C-2), 120.5 (CN), 75.9 (C-1), 75.4 (C-3), 52.0, 43.4, 38.3, 33.8, 33.8, 28.3, 25.7, 24.5, 24.4, 11.5 (C-7).

HRMS (ESI-Q-TOF) *m/z*: 235.1799 $[M+H]^+$; calcd. for $C_{14}H_{23}N_2O$: 235.1805.

The enantiomeric ratio was determined by UPC^2 analysis using Trefoil CEL1, gradient CO_2/iPrOH = 100:0 to 85:15 until 2 min then isocratic 85:15, flow rate = 1 mL/min, 25 °C, λ = 290 nm, t_R = 5.21 min (minor) and t_R = 5.82 min (major), 98% ee.

(3*R*,5*S*)-2-(cyclohexylamino)-3-hydroxy-5-phenylcyclopent-1-ene-1-carbonitrile (3b)

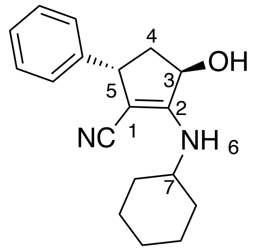

Prepared according the general procedure using hemiacetal **1i** (42 mg, 0.15 mmol) and cyclohexyl isocyanide (19 µL, 0.15 mmol). Obtained after flash column chromatography purification (*n*-hexane/EtOAc 4:1) as a pale brown oil (19 mg, 0.07 mmol, 45% yield, 99:1 dr, > 99% ee). $[\alpha]_D^{20}$ – 3.4 (*c* 0.5, acetone, 20°C). R_f = 0.34 (*n*-hexane/ EtOAc 7:3).

^{1}H NMR (400 MHz, $CDCl_3$) δ = 7.38 – 7.26 (m, 3H), 7.28 – 7.16 (m, 2H), 4.79 (t, *J* = 7.4 Hz, 1H, H-3), 4.09 (d, *J* = 8.7 Hz, 1H, H-6), 3.92 – 3.78 (m, 1H, H-7), 2.32 – 2.07 (m, 3H), 1.86 – 1.64 (m, 5H), 1.53 – 1.39 (m, 2H), 1.30 – 1.11 (m, 3H).

^{13}C NMR (100 MHz, $CDCl_3$) δ = 158.8 (C-2), 143.7, 128.7, 126.8, 126.7, 120.0 (CN), 75.3 (C-3), 74.9 (C-1), 52.0, 47.4, 42.2, 33.8, 33.6, 25.5, 24.3, 24.3.

HRMS (ESI-Q-TOF) *m/z*: 283.1815 $[M+H]^+$; calcd. for $C_{18}H_{22}N_2O$: 283.1805.

The enantiomeric ratio was determined by UPC^2 analysis using Trefoil CEL1, gradient CO_2/iPrOH = 100:0 to 85:15 until 2 min then isocratic 85:15, flow rate = 1 mL/min, 25 °C, λ = 290 nm, t_R = 7.49 min (minor) and t_R = 9.71 min (major), > 99% ee.

(3*R*,5*R*)-2-(cyclohexylamino)-5-heptyl-3-hydroxycyclopent-1-ene-1-carbonitrile (3c)

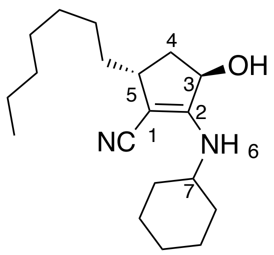

Prepared according the general procedure using hemiacetal **1c** (45 mg, 0.15 mmol) and cyclohexyl isocyanide (19 µL, 0.15 mmol). Obtained after flash column chromatography purification (*n*-hexane/EtOAc 4:1) as a pale brown oil (35 mg, 0.11 mmol, 76% yield, 95:5 dr, 98% ee). $[\alpha]_D^{20}$ – 1.8 (*c* 0.5, acetone, 20°C). R_f = 0.61 (*n*-hexane/ EtOAc 7:3).

^{1}H NMR (400 MHz, $CDCl_3$) δ = 4.67 (d, *J* = 8.5 Hz, 1H, H-6), 4.60 (t, *J* = 7.2 Hz, 1H, H-3), 3.78 – 3.64 (m, 1H, H-7), 2.82 – 2.73 (m, 1H), 2.73 – 2.52 (m, 1H), 2.09 – 2.00 (m, 1H), 1.98 (ddd, *J* = 13.2, 7.4, 2.6 Hz, 1H), 1.84 – 1.75 (m, 1H), 1.77 – 1.66 (m, 2H), 1.66 – 1.51 (m, 2H), 1.46 – 1.32 (m, 2H), 1.33 – 1.08 (m, 14H), 0.86 (t, *J* = 6.6 Hz, 3H).

^{13}C NMR (100 MHz, $CDCl_3$) δ = 158.4 (C-2), 120.8 (CN), 75.5 (C-3), 74.1 (C-1), 52.0, 42.0, 38.4, 35.8, 33.7, 33.7, 32.0, 29.8, 29.4, 27.5, 25.6, 24.4, 24.3, 22.7, 14.2.

HRMS (ESI-Q-TOF) *m/z*: 305.2581 $[M+H]^+$; calcd. for $C_{19}H_{33}N_2O$: 305.2587.

The enantiomeric ratio was determined by UPC^2 analysis using Trefoil CEL1, gradient CO_2/iPrOH = 100:0 to 85:15 until 2 min then isocratic 85:15, flow rate = 1 mL/min, 25 °C, λ = 254 nm, t_R = 5.72 min (minor) and t_R = 6.23 min (major), 98% ee.

(3*R*,5*S*)-2-(*tert*-butylamino)-3-hydroxy-5-phenylcyclopent-1-ene-1-carbonitrile (3d)

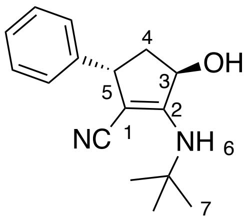

Prepared according the general procedure using hemiacetal **1i** (42 mg, 0.15 mmol) and *tert*-butyl isocyanide (17 µL, 0.15 mmol). Obtained after flash column chromatography purification (*n*-hexane/EtOAc 4:1) as a pale brown solid (22 mg, 0.09 mmol, 58% yield, > 99:1 dr, 94% ee). $[\alpha]_D^{20}$ – 4.4 (*c* 0.5, acetone, 20°C). R_f = 0.26 (*n*-hexane/ EtOAc 7:3).

^{1}H NMR (400 MHz, Acetone-d_6) δ = 7.24 – 7.14 (m, 2H), 7.08 (d, *J* = 7.6 Hz, 3H), 5.40 (s, 1H, H-6), 4.60 (t, *J* = 11.7 Hz, 1H, H-3), 3.94 (t, *J* = 8.4 Hz, 1H, H-5), 2.09 – 1.92 (m, 2H, H-4), 1.36 (s, 9H, H-7).

^{13}C NMR (100 MHz, $CDCl_3$) δ = 157.0 (C-2), 143.7, 128.9, 127.0, 126.9, 121.7 (CN), 76.5 (C-3), 74.1 (C-1), 52.4, 48.3, 41.9, 30.4.

HRMS (ESI-Q-TOF) *m/z*: 257.1641 $[M+H]^+$; calcd. for $C_{16}H_{21}N_2O$: 257.1648.

The enantiomeric ratio was determined by UPC^2 analysis using Trefoil CEL1, gradient CO_2/iPrOH = 100:0 to 85:15 until 2 min then isocratic 85:15, flow rate = 1 mL/min, 25 °C, λ = 254 nm, t_R = 5.32 min (minor) and t_R = 6.04 min (major), 94% ee.

Continuous-flow one-pot synthesis of (3*R*,5*R*)-3-(*tert*-butylamino)-2-(cyclohexylamino)-5-ethylcyclopent-1-ene-1-carbonitrile (2d)

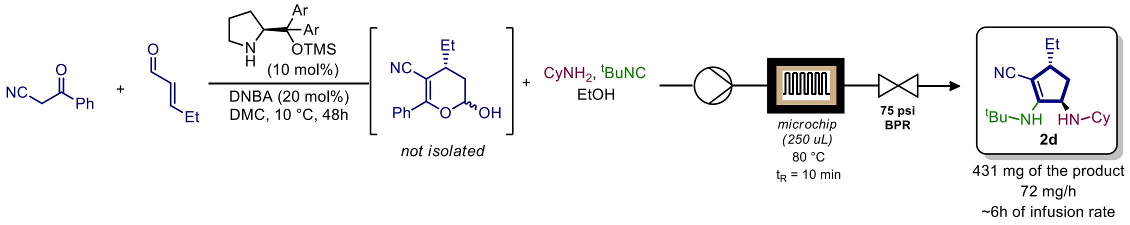

General material information for continuous-flow setup:

1. The Syringe Pump was a Cole-Palmer apparatus 74900 Series model (East Bunker Ct Vernon Hills , IL).
2. 5 mL Syringe Pack, (10 mm diameter).
3. Fluorinated ethylene propylene (FEP) tubings were purchased from IDEX health & science (Oak Harbor, WA).
4. Super Flangeless fittings (include nuts and ferrules) were purchased from IDEX health & science (Oak Harbor, WA).
5. The Asia Heater block was purchased from Syrris (Orchard, Rd).

6. Microchip (250 μL) was purchased from Syrris (Orchard, Rd).
7. Back pack pressure (75 psi), was purchased from IDEX health & science (Oak Harbor, WA).

Experimental procedure:

To a solution of Jørgensen's catalyst (0.015 mmol, 0.1 equiv.), 3,5-dinitrobenzoic acid (0.03 mmol, 0.2 equiv.) and *trans*-pent-2-enal (0.18 mmol, 1.2 equiv.) in dimethyl carbonate (1.0 mL) was added benzoylacetonitrile (0.15 mmol, 1.0 equiv.). The resulting solution was stirred for 48h at 10 °C. After this time, ethanol (1.0 mL), the cyclohexylamine (0.15 mmol, 1.0 equiv.) and *t*-butyl isocyanide (0.15 mmol, 1.0 equiv.) were added to the crude hemiacetal solution. The Syringe Pump, a Cole-Palmer apparatus, was used to deliver the reaction mixture from a plastic syringe (5 mL), which was connected to the fluorinated ethylene propylene (FEP; thru hole 0.3") tubing at a microchip reactor. The microchip reactor was heated using an Asia Heater block. This reaction mixture was injected into the microreactor at 80 °C and residence time of 10 min by a syringe pump until the standard condition is reached. The back-pack pressure of 75 psi was used.

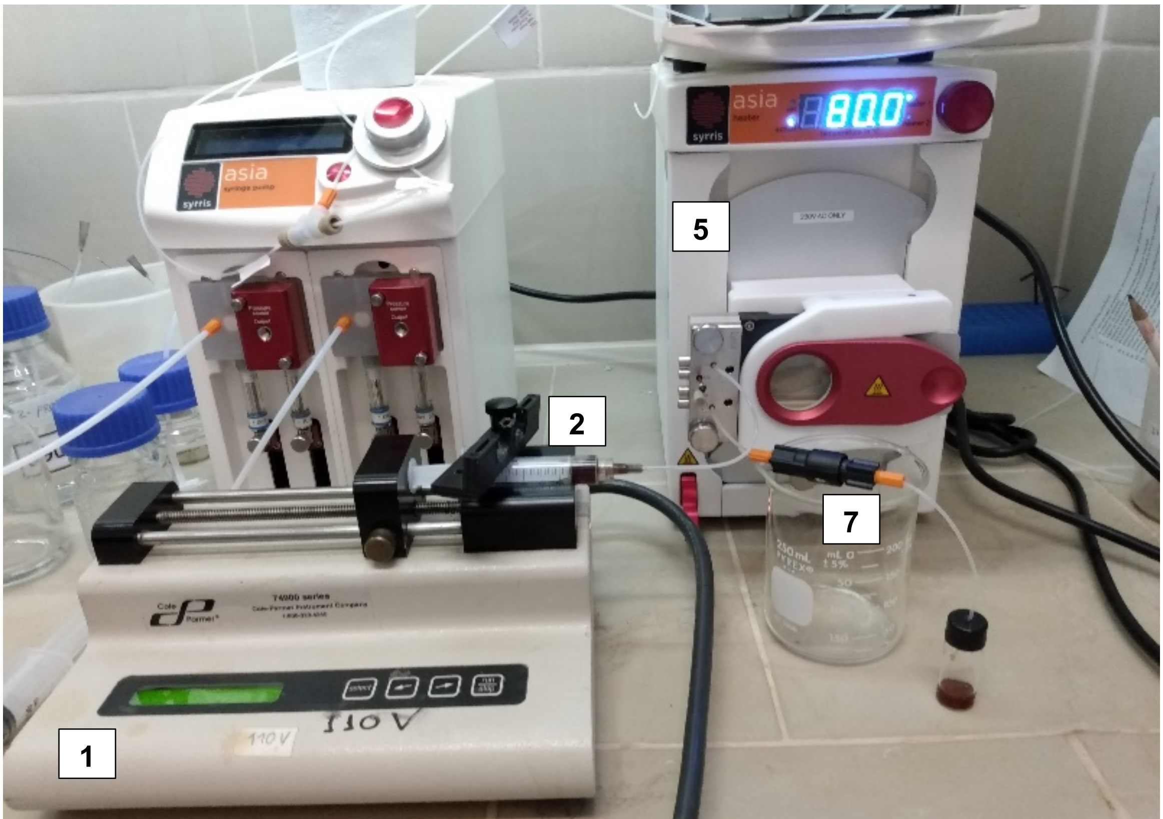

Figure S1. Setup of the continuous-flow system.

Synthesis of the bulky tertiary amine 4

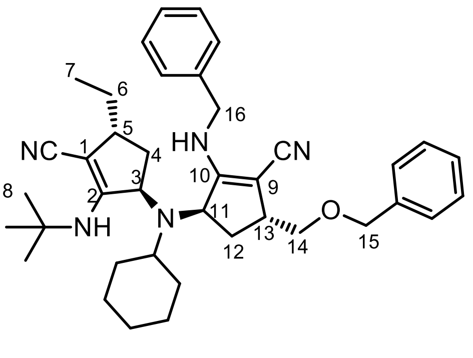

The hemiacetal **1a** (34 mg, 0.15 mmol), was dissolved in trifluoroethanol (0.3 mL) and cyclohexylamine (17 mg, 0.15 mmol) was added to the mixture under magnetic stirring at room temperature. After 10 minutes, tert-butyl isocyanide (17 μL, 0.15 mmol) was dissolved in the reaction mixture. The flask was sealed and irradiated for 20 min under high-speed magnetic stirring in the microwave reactor (300 W), while the temperature was raised up to 70 °C. Then hemiacetal **1f** (48 mg, 0.15 mmol) was added to the mixture and stirred at room temperature for 10 min. Benzyl isocyanide (19 μL, 0.15 mmol) was introduced to this solution and the mixture was irradiated again under the same conditions above. The product **4** was obtained after flash chromatography purification as a brown oil (38 mg, 0.06 mmol, 41% yield, 82:18 dr, > 99% ee). $[\alpha]_D^{20}$ – 1.9 (*c* 0.5, acetone, 20°C). R_f= 0.60 (*n*-hexane/ EtOAc 7:3).

^{1}H NMR (400 MHz, $CDCl_3$) δ = 7.38 – 7.28 (m, 12H),4.78 – 4.63 (m, 3H), 4.58 (s, 2H), 3.99 – 3.83 (m, 2H), 3.70 (dd, *J* = 9.4, 3.7 Hz, 1H), 3.52 (dd, *J* = 9.4, 6.2 Hz, 1H), 2.92 – 2.82 (m, 1H), 2.64 – 2.51 (m, 1H), 2.48 – 2.23 (m, 1H), 2.16 (dt, *J* = 12.1, 7.5 Hz, 1H), 2.10 – 1.95 (m, 1H), 1.79 – 1.67 (m, 3H), 1.64 – 1.50 (m, 4H), 1.42 (s, 9H, H-), 1.33 – 1.25 (m, 4H), 1.21 – 1.08 (m, 4H), 0.84 (t, *J* = 7.2 Hz, 3H, H-7).

^{13}C NMR (100 MHz, $CDCl_3$) δ = 159.9 (C-10), 154.9 (C-2), 138.6, 137.7, 129.1, 128.5, 128.2, 127.9, 127.7, 127.6, 122.7 (CN), 119.6 (CN), 73.7 (C-1), 73.5 (C-9) 73.4 (C-15), 72.2 (C-14), 63.5, 62.1, 54.2, 52.0, 48.6 (C-16), 43.6, 40.2, 35.6, 30.3 (C-8), 30.2, 30.0, 28.0, 26.3, 25.4, 11.9 (C-7).

HRMS (ESI-Q-TOF) m/z: 606.4169 $[M+H]^+$, calcd. for $C_{39}H_{52}N_5O$: 606.4166.

The enantiomeric ratio was determined by UPC^2 analysis using Trefoil CEL1, gradient CO_2/MeCN = 100:0 to 60:40 in 5 min then isocratic 60:40, flow rate = 1 mL/min, 35 °C, λ = 286 nm, t_R = 4.84 min (major) and t_R = 5.48 min (minor), >99% ee.

Synthesis of methyl ((3*R*,5*R*)-2-cyano-3-ethyl-5-(tritylamino)cyclopent-1-en-1-yl)glycinate (5)

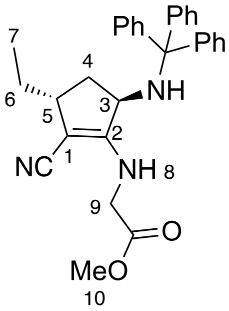

Prepared according the general procedure for the synthesis of cyclopentenyl amines using hemiacetal **1a** (133 mg, 0.58 mmol), tritylamine (150 mg, 0.58 mmol) and methyl isocyanoacetate (56 µL, 0.58 mmol). Obtained after flash chromatography purification as a yellow solid (157 mg, 58% yield, 0.34 mmol). m.p.: 76-81 °C. $[\alpha]_D^{20}$ + 35.6 (*c* 0.5, acetone, 20°C). R_f= 0.27 (*n*-hex/EtOAc 4:1).

^{1}H NMR (400 MHz, $CDCl_3$) δ = 7.41 (d, *J* = 8.0 Hz, 5H), 7.24 – 7.09 (m, 10H), 6.15 (t, *J* = 5.1 Hz, 1H, H-8), 4.39 – 4.24 (m, 2H, H-9), 3.77 (s, 3H, H-10), 2.27 – 2.13 (m, 1H), 1.27 – 1.13 (m, 2H), 0.95 – 0.62 (m, 2H), 0.55 (t, *J* = 7.4 Hz, 3H, H-7), 0.38 (dd, *J* = 12.7, 6.8 Hz, 1H, H-4a).

^{13}C NMR (100 MHz, $CDCl_3$) δ = 170.7 (C=O), 160.6 (C-2), 146.3, 128.8, 128.3, 126.9, 120.7 (CN), 75.2, 70.7, 58.8, 52.8, 45.4, 43.3, 38.0, 27.0, 11.9 (C-7).

HRMS (ESI-Q-TOF) m/z: 488.2756 $[M+Na]^+$, calcd. for $C_{30}H_{31}N_3O_2Na$: 488.2308.

Procedure for ester deprotection of compound 5

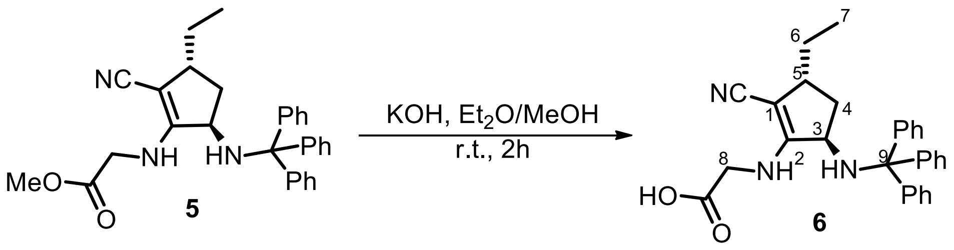

The compound **5** (73 mg, 0.15 mmol) was dissolved in Et_2O/MeOH (10:1, 2 mL). Next, KOH (9 mg, 1.0 equiv.) was added to this mixture under magnetic at room temperature. After 2 hours, the solvent was removed and the residue suspended in EtOAc (20 mL) and washed with 0.5 M citric acid solution (2x10 mL). The carboxylic acid **6** was obtained after flash chromatography purification as a yellow oil (54 mg, 76% yield). R_f = 0.30 (DCM/MeOH 9:1).

^{1}H NMR (400 MHz, CD_3OD) δ = 7.58 (d, *J* = 7.9 Hz, 6H), 7.30 (t, *J* = 7.7 Hz, 6H), 7.20 (t, *J* = 7.2 Hz, 3H), 4.18 – 4.05 (m, 2H, H-8), 3.76 (dd, *J* = 10.4, 6.7 Hz, 1H, H-3), 2.22 (dd, *J* = 13.4, 8.2 Hz, 1H, H-5), 1.97 – 1.55 (m, 1H), 1.30 – 1.14 (m, 1H), 1.08 – 0.85 (m, 3H), 0.67 (dd, *J* = 12.8, 5.4 Hz, 3H, H-7), 0.40 (dd, *J* = 12.7, 6.7 Hz, 1H, H-4a).

^{13}C NMR (100 MHz, CD_3OD) δ = 175.9 (C=O), 163.9 (C-2), 148.1, 130.1, 129.0, 127.5, 123.0 (CN), 71.7 (C-9), 71.4 (C-1), 59.6 (C-3), 48.8 (C-8), 44.4 (C-5), 38.2 (C-4), 28.1 (C-6), 12.2 (C-7).

HRMS (ESI-Q-TOF) m/z: 450.2184 $[M-H]^-$, calcd. for $C_{29}H_{28}N_3O_2$: 450.2187.

Procedure for the trityl deprotection of compound 5

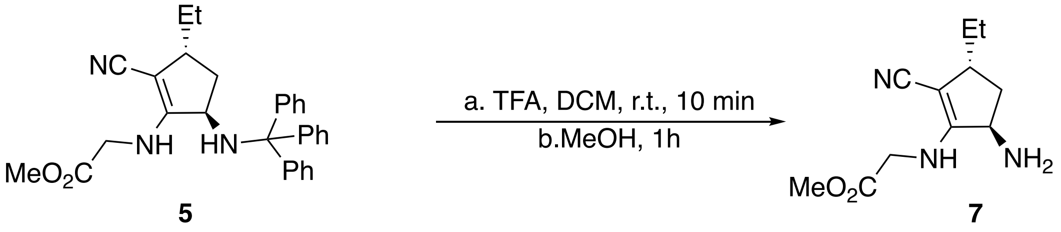

The compound **5** was added to a solution of TFA (60% v/v) in DCM (0.8 mL) under magnetic stirring at room temperature for 10 min, and methanol (0.5 mL) was added to the reaction. After 1h, the solvent was removed under reduced pressure and the residue was dissolved in EtOAc (10 mL) and washed with water (2x20 mL). Aqueous solution was adjusted until pH = 10 with NaOH 1M, and extracted with EtOAc (3x 20 mL), the organic solvent was removed under reduced pressure. The amine **7** was obtained after flash chromatography purification as a yellow solid (29 mg, 83% yield). m.p.: 104-110 °C. R_f= 0.37 (DCM/MeOH 9:1).

^{1}H NMR (400 MHz, CD_3OD) δ = 4.31 – 4.25 (m, 3H), 3.81 (s, 3H), 3.01 (qd, *J* = 7.3, 3.9 Hz, 1H), 2.08 (ddd, *J* = 14.5, 7.2, 2.7 Hz, 1H), 1.96 (dt, *J* = 14.6, 7.5 Hz, 1H), 1.78 (dqd, *J* = 14.8, 7.5, 4.1 Hz, 1H), 1.34 (ddd, *J* = 15.5, 13.7, 7.6 Hz, 1H), 0.96 (t, *J* = 7.4 Hz, 3H).

^{13}C NMR (100 MHz, CD_3OD) δ = 171.9, 155.5, 119.3, 81.7, 57.4, 53.2, 46.2, 45.2, 34.0, 28.4, 10.8.

HRMS (ESI-Q-TOF) m/z: 246.1207 $[M+Na]^+$, calcd. for $C_{11}H_{17}N_3O_2Na$: 246.1213.

One-pot procedure for the synthesis of *cis*-cyclopentenyl amine 8

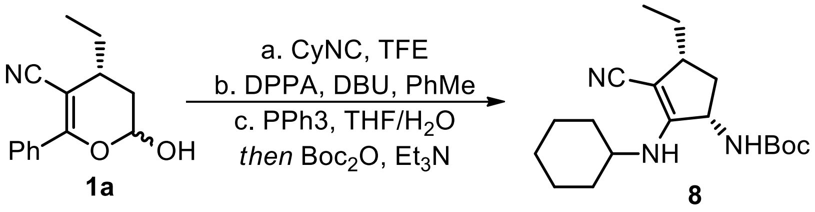

Step a): the cyclohexyl isocyanide (127 µL, 1 mmol) was added to a solution of the hemiacetal **1a** (229 mg, 1 mmol) in trifluoroethanol (2 mL) at room temperature. The flask was sealed and irradiated for 20 min (300 W) under high-speed magnetic stirring in the microwave reactor, while the temperature was raised up to 70 °C. The volatiles were concentrated under reduced pressure and the resulting crude product was used without further purification.

Step b): the crude product was dissolved in toluene (4 mL) and 0.43 mL (2.0 equiv.) of diphenylphosphoryl azide (DPPA) was added to the solution under magnetic stirring at room temperature. After 5 minutes, 1,8-diazabicyclo(5.4.0)undec-7-ene (DBU, 0.3 mL, 2.0 equiv.) was added to this mixture and allowed to react for 24 hours. The solvent was evaporated under reduced pressure and product used in the next step.

Step c): the crude product was dissolved in THF (10 mL) and triphenylphosphine (2.0 equiv, 525 mg) was added. After 1 hour under magnetic stirring at room temperature, water (1 mL) was introduced and the mixture was allowed to react. After 24 hours, triethylamine (3.0 equiv, 0.2 mL) and Boc_2O (3.0 equiv, 0.32 mL) were added to the reaction mixture. After 24 hours, the solvent was removed under reduced pressure and the residue dissolved in EtOAc (30 mL), washed with water (2x20 mL) and concentrated. The compound **8** was obtained after flash chromatography purification (*n*-hexano/EtOAc) as a yellow oil (90 mg, 59% yield, 96:4 dr). $[\alpha]_D^{20}$ -62.8 (*c* 0.5, acetone, 20°C). R_f= 0.50 (*n*-hex/EtOAc 4:1).

^{1}H NMR (400 MHz, $CDCl_3$) δ = 5.21 (d, *J* = 7.0 Hz, 1H), 4.56 (s, 2H), 3.74 (qd, *J* = 9.4, 4.7 Hz, 1H), 2.69 – 2.59 (m, 1H), 2.48 – 2.39 (m, 1H), 2.06 – 1.90 (m, 2H), 1.88 – 1.76 (m, 1H), 1.71 – 1.53 (m, 4H), 1.48 – 1.38 (m, 11H), 1.30 – 1.01 (m, 5H), 0.90 (t, *J* = 7.4 Hz, 3H).

^{13}C NMR (100 MHz, $CDCl_3$) δ = 158.0, 156.7, 120.4, 80.7, 74.0, 56.3, 51.6, 43.1, 35.5, 33.6, 33.3, 28.4, 25.7, 24.1, 24.0, 11.0.

HRMS (ESI-Q-TOF) m/z: 334.2407 $[M+H]^+$, calcd. for $C_{19}H_{32}N_3O_2$: 334.2489.

GC-MS chromatograms and mass fragmentation analyses

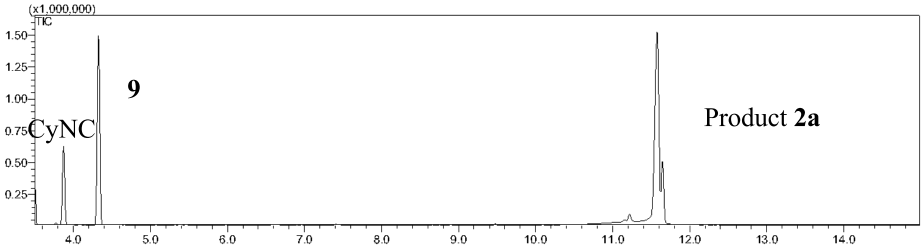

Figure S2. Chromatogram of crude reaction mixture of compound **2a** in TFE.

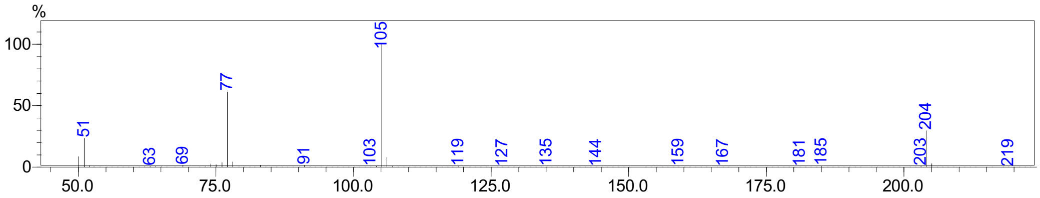

Figure S3. MS Spectrum of compound **9**.

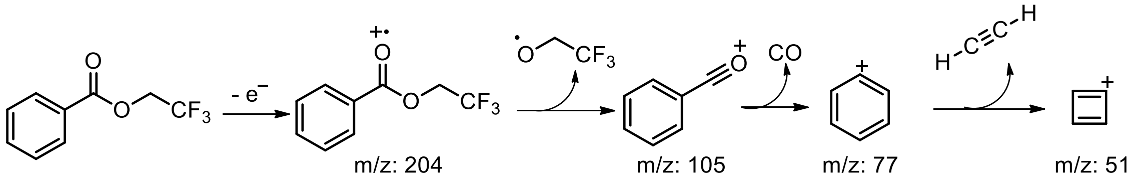

Scheme S1. Fragmentation proposal of compound **9**.

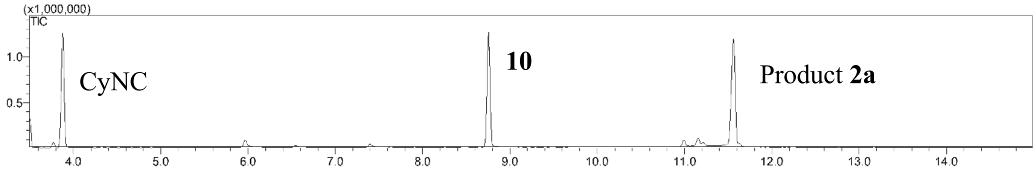

Figure S4. Chromatogram of the crude reaction mixture of compound **2a** in DCM.

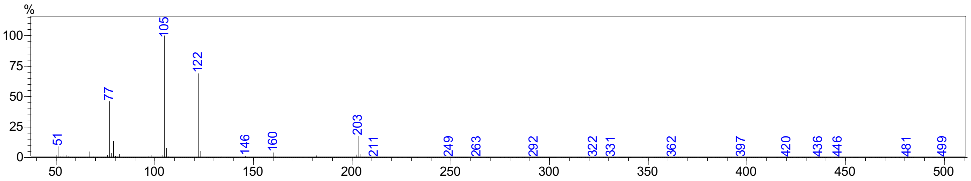

Figure S5. MS Spectrum of compound **10**.

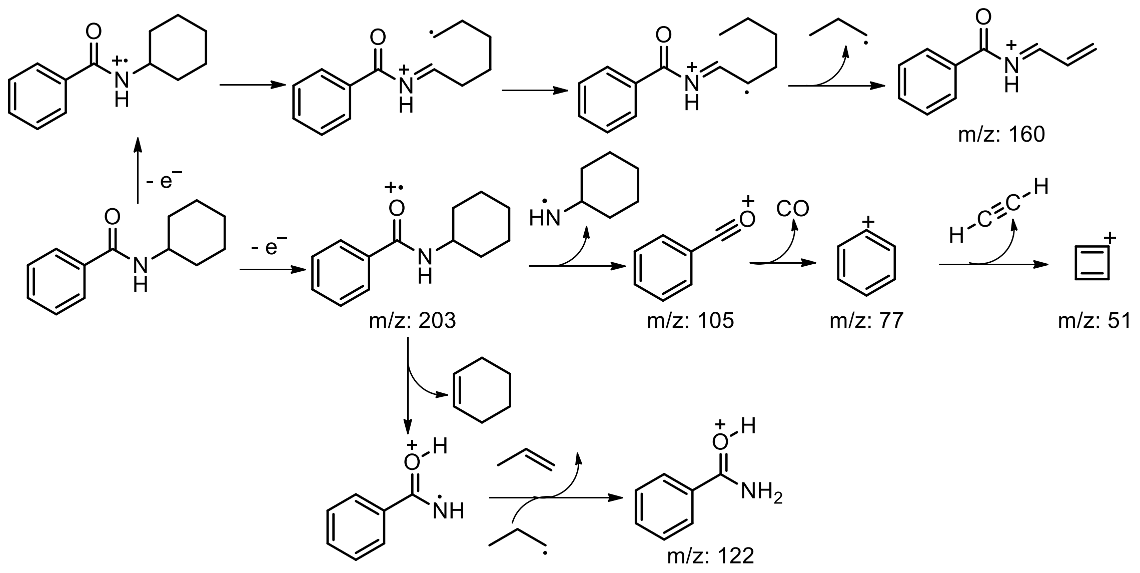

Scheme S2. Fragmentation proposal of compound **10**.

NMR Spectra

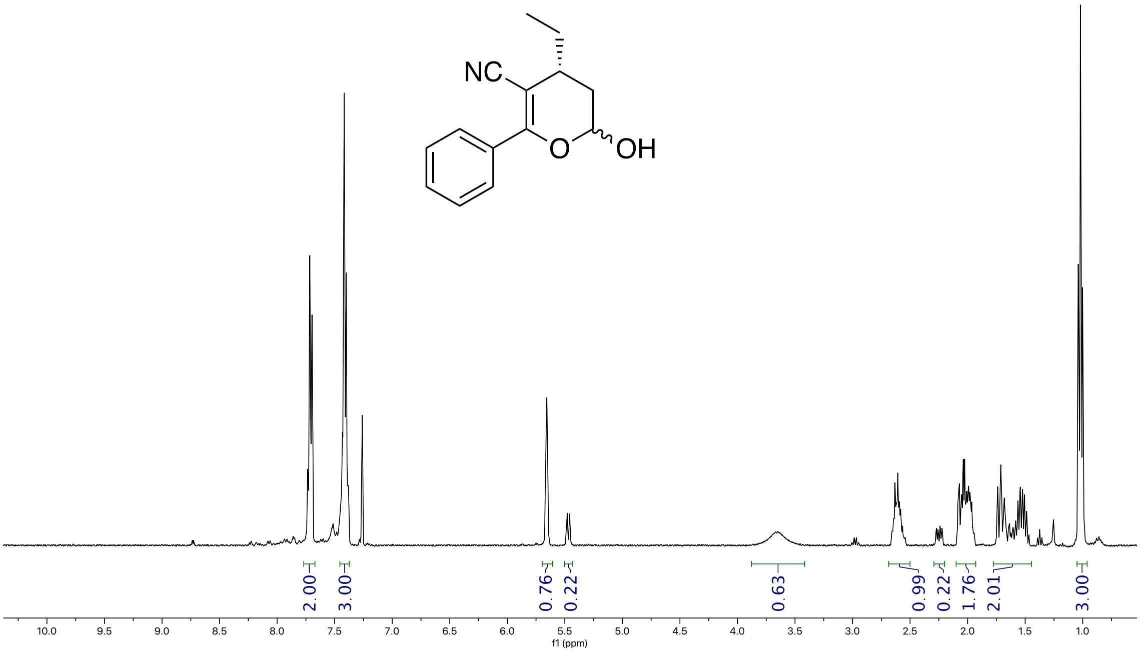

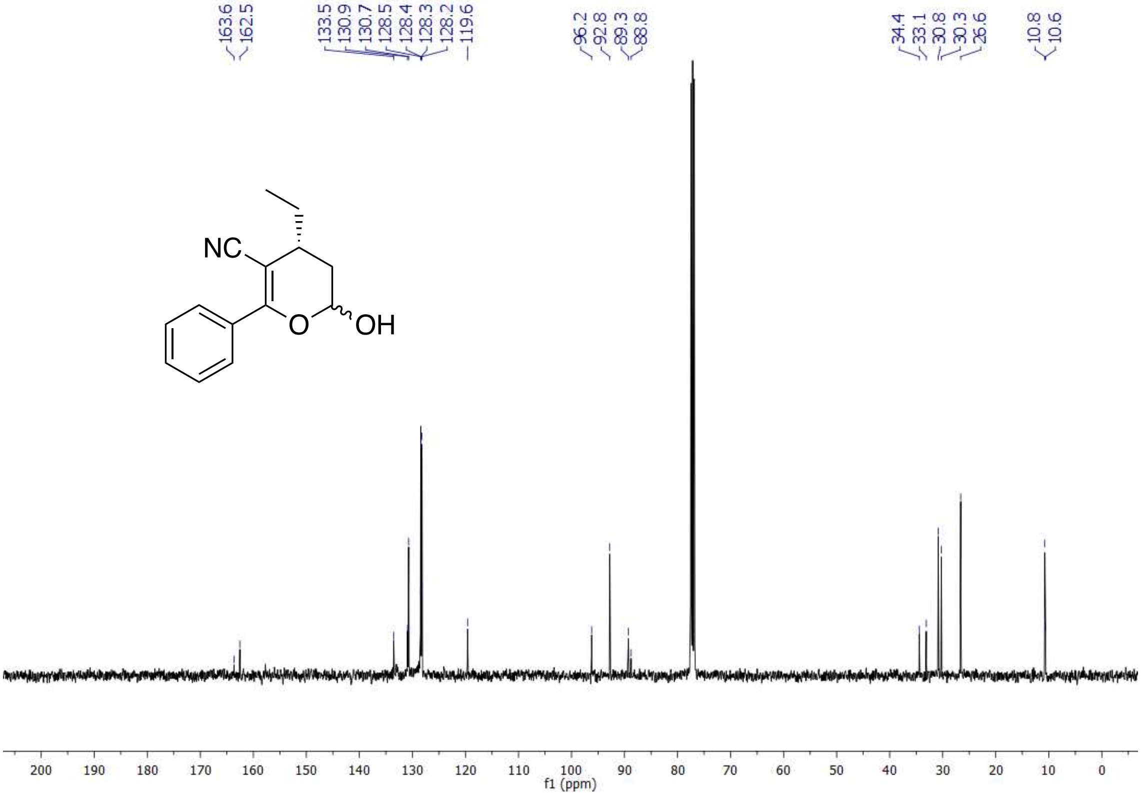

Figure S6. ^{1}H and ^{13}C NMR spectra in $CDCl_3$ of compound **1a**.

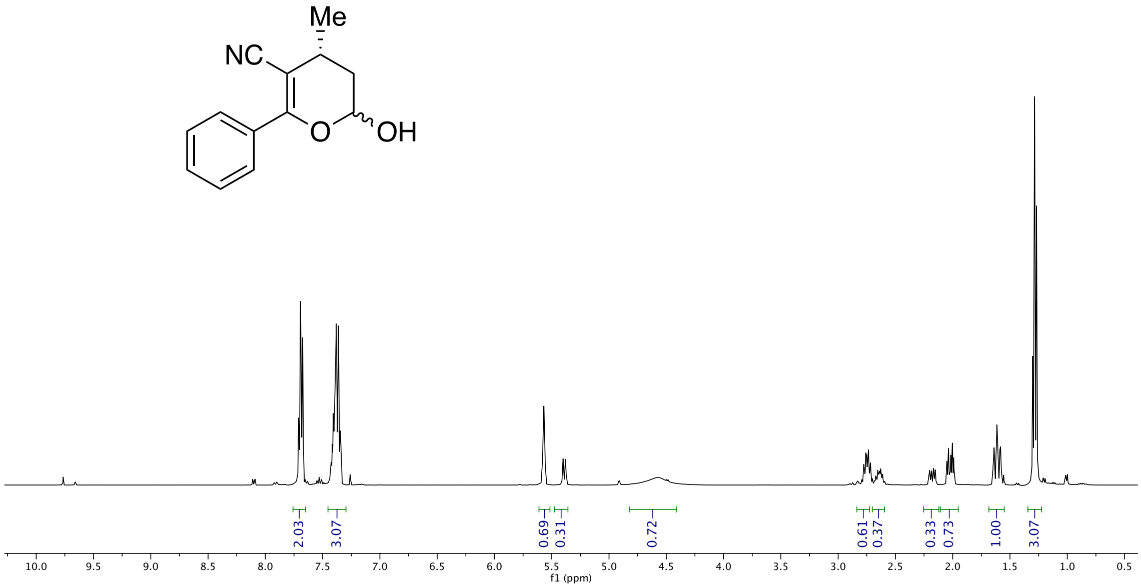

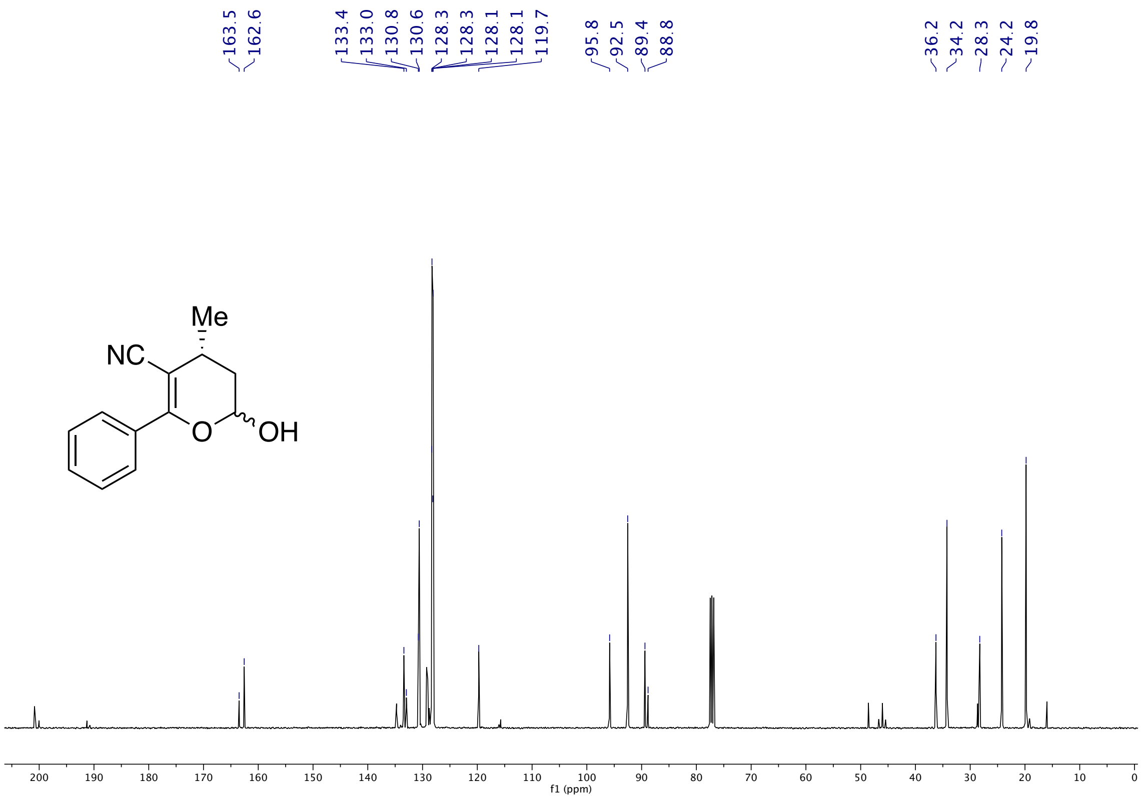

Figure S7. ^{1}H and ^{13}C NMR spectra in $CDCl_3$ of compound **1b**.

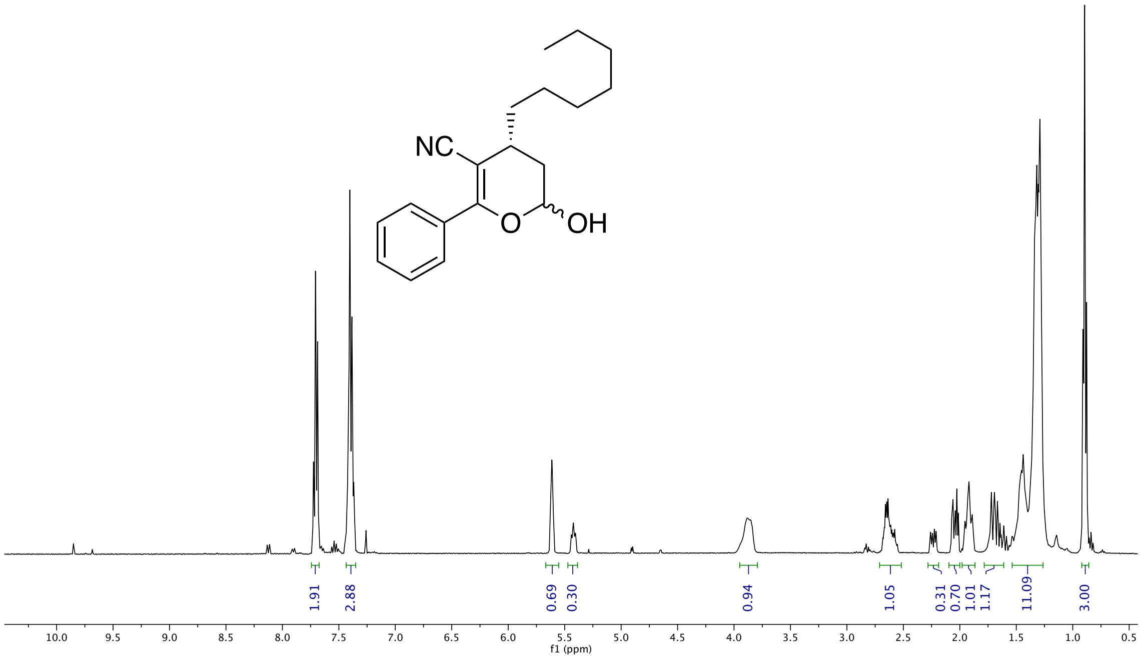

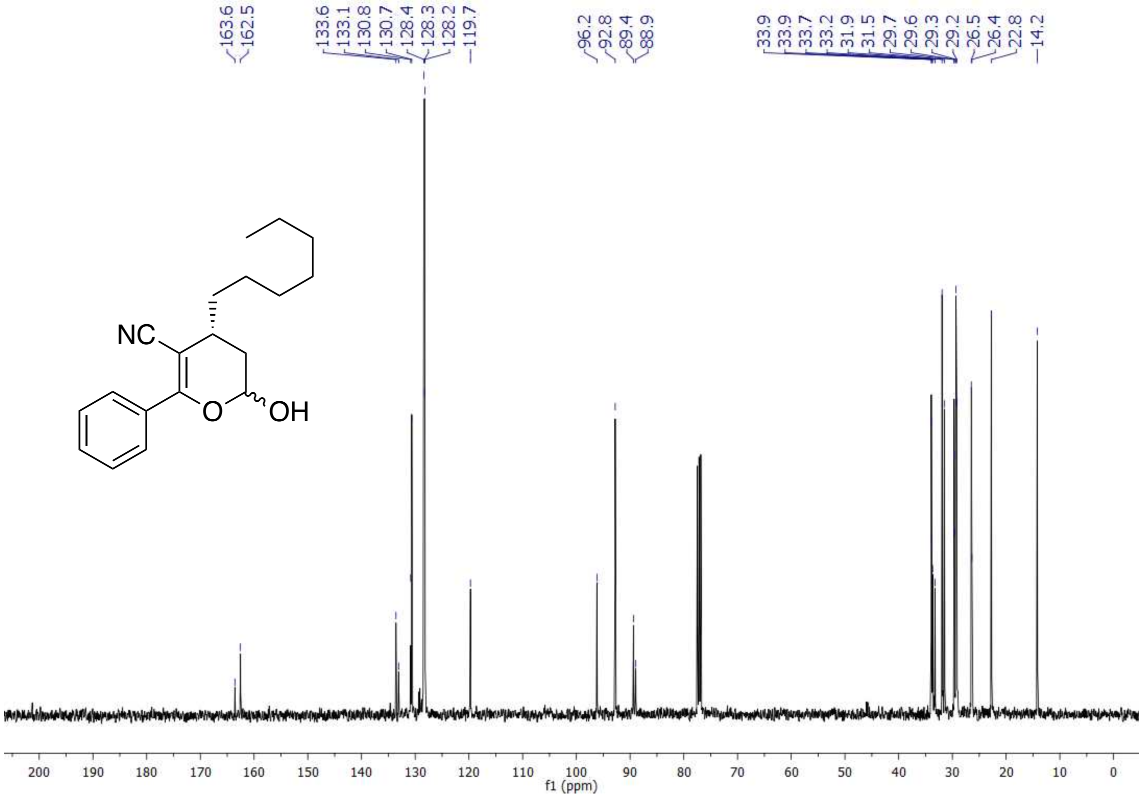

Figure S8. ^{1}H and ^{13}C NMR spectra in $CDCl_3$ of compound **1c**.

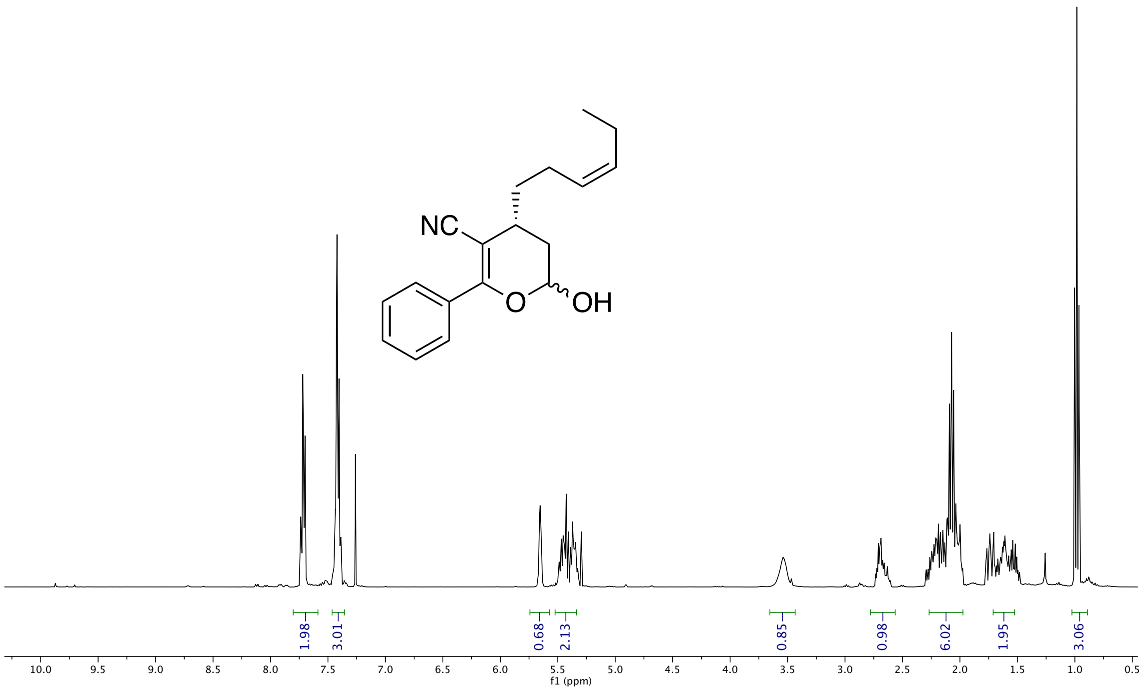

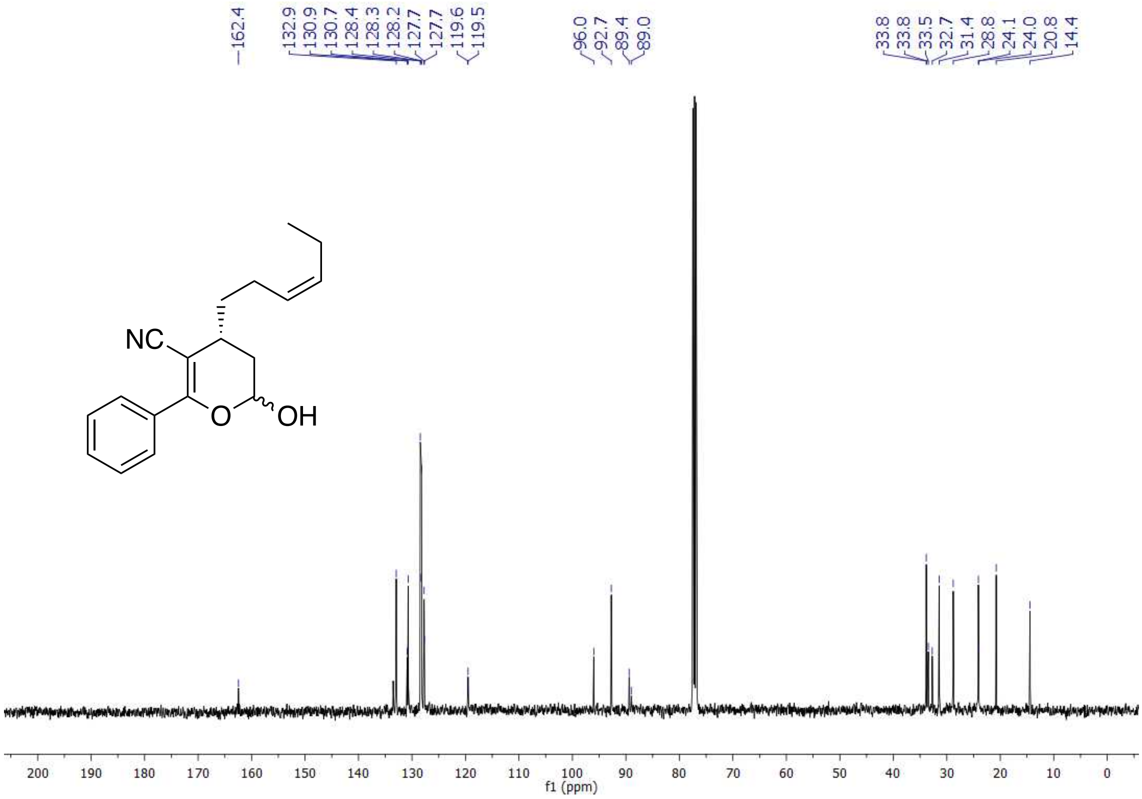

Figure S9. ^{1}H and ^{13}C NMR spectra in $CDCl_3$ of compound **1d**.

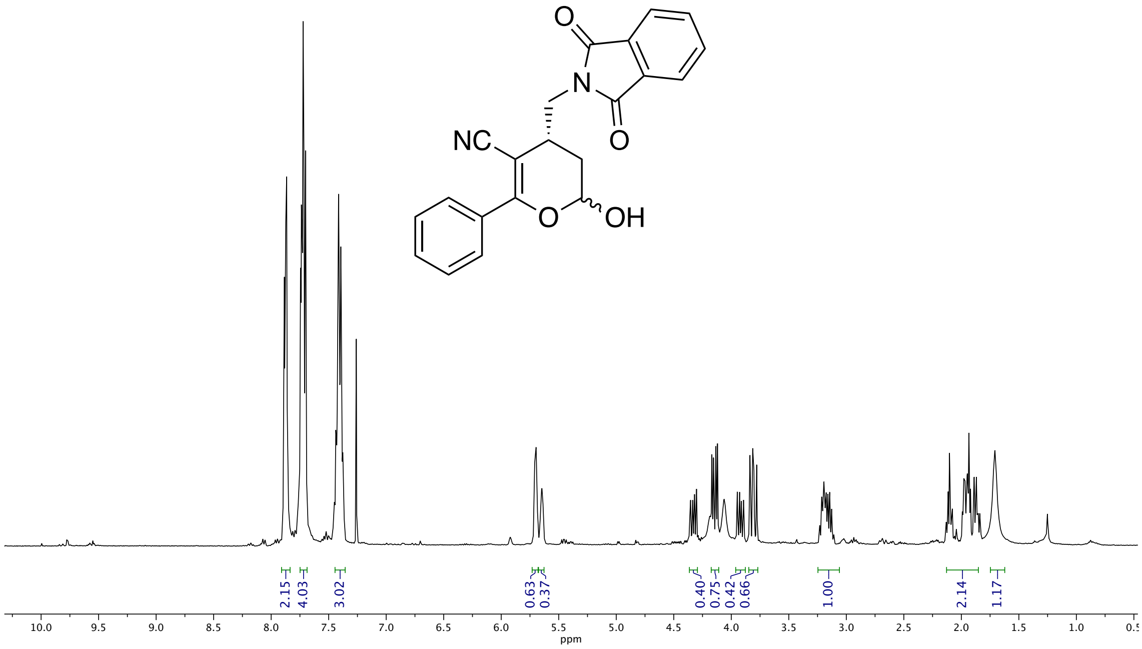

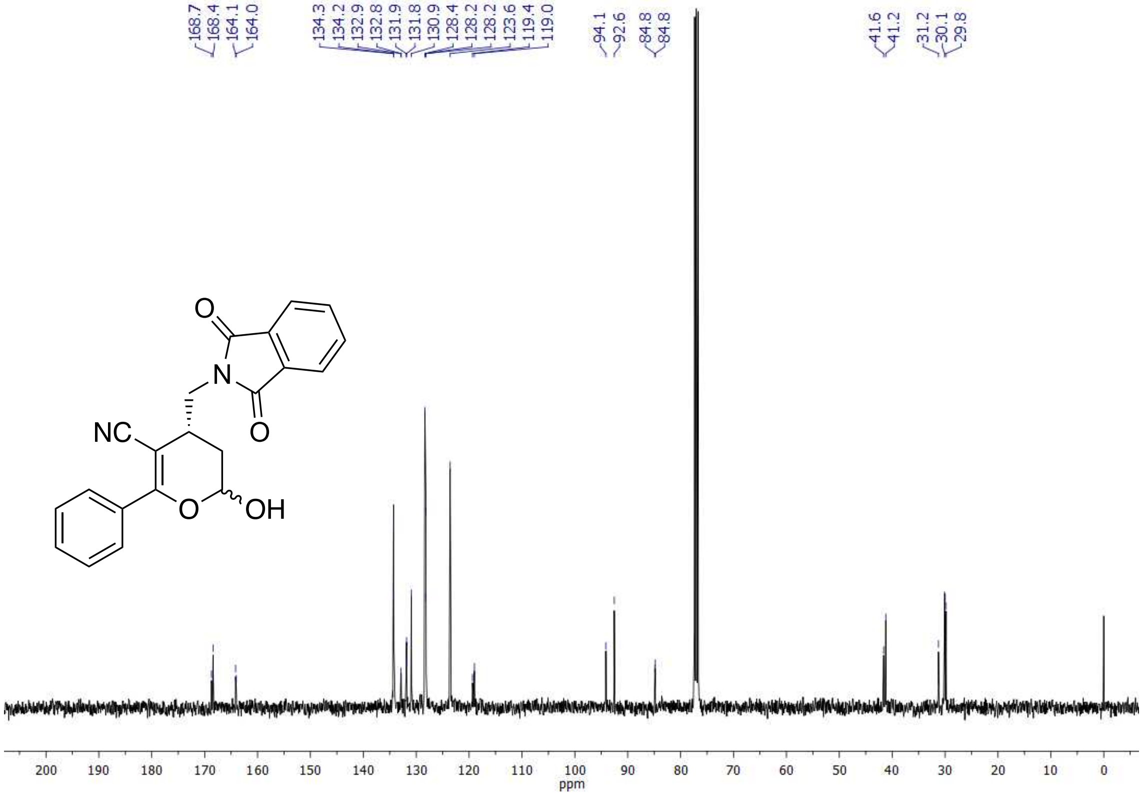

Figure S10. ^{1}H and ^{13}C NMR spectra in $CDCl_3$ of compound **1e**.

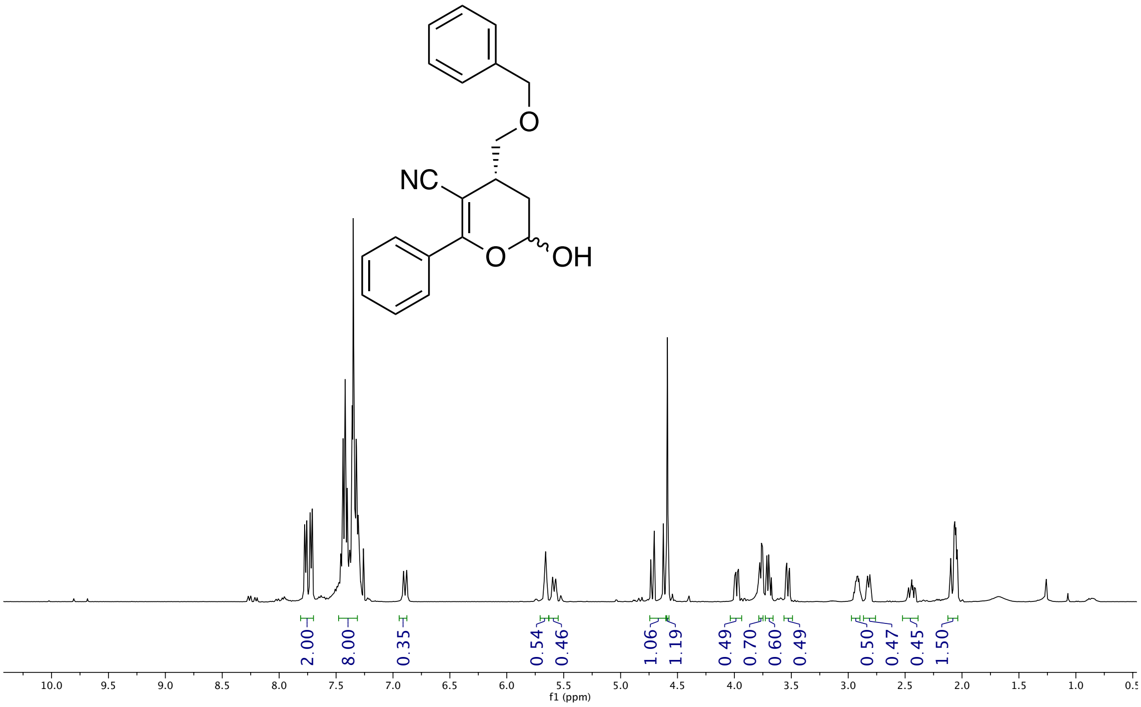

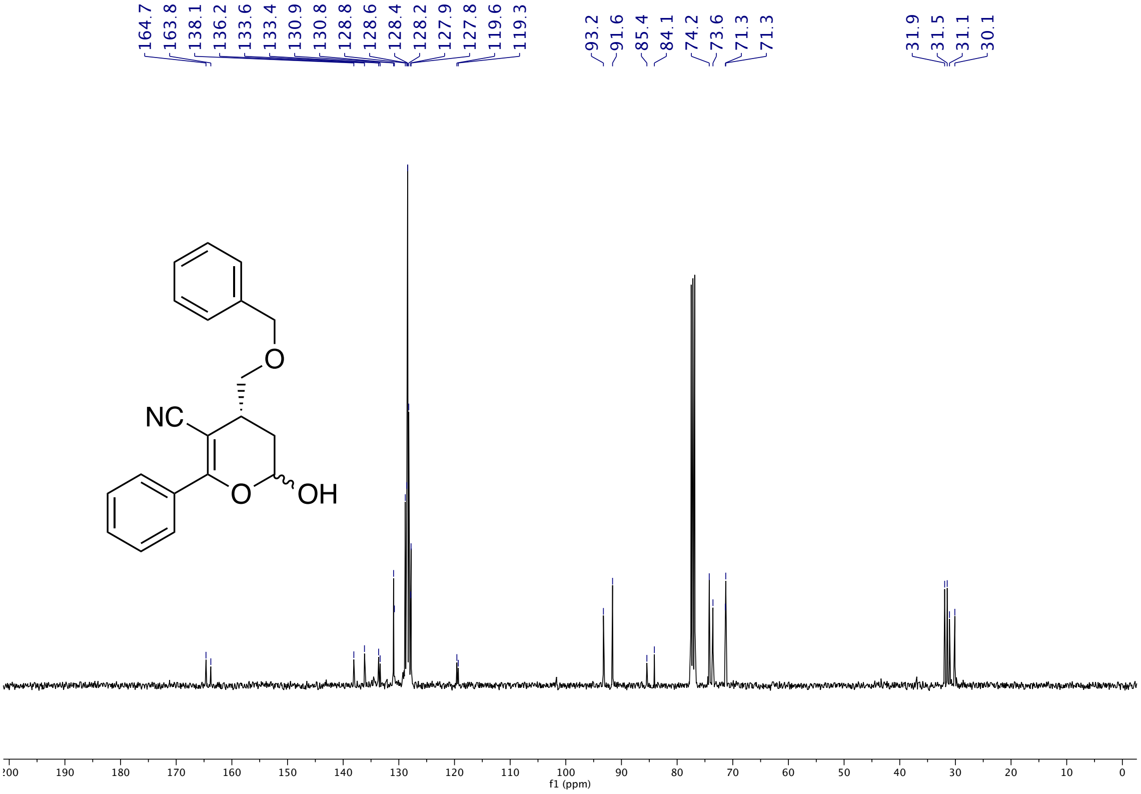

Figure S11. ^{1}H and ^{13}C NMR spectra in $CDCl_3$ of compound **1f.**

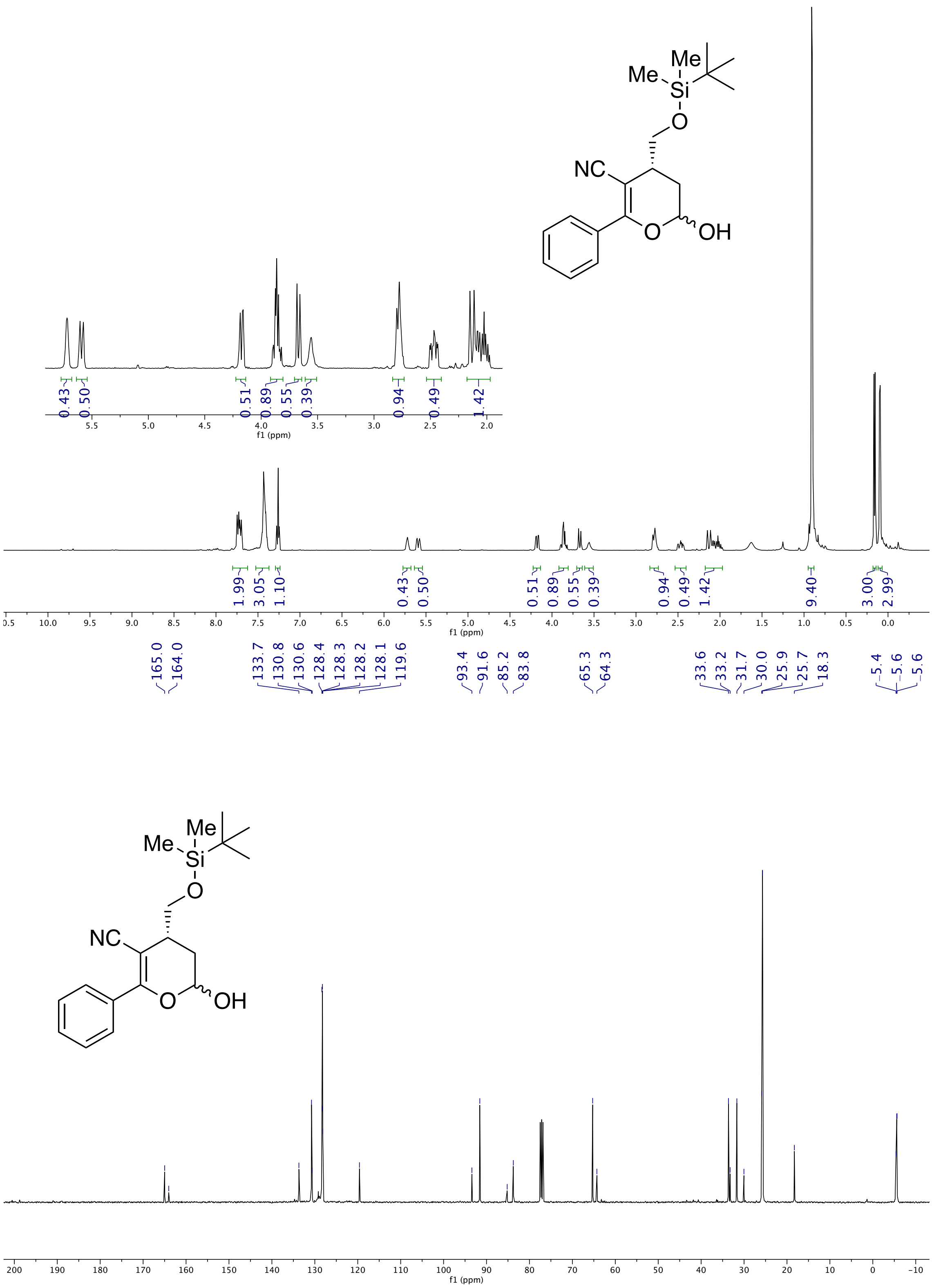

Figure S12. ^{1}H and ^{13}C NMR spectra in $CDCl_3$ of compound **1g.**

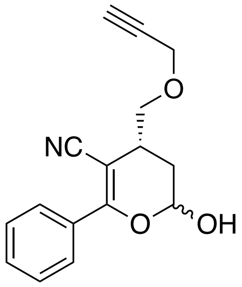

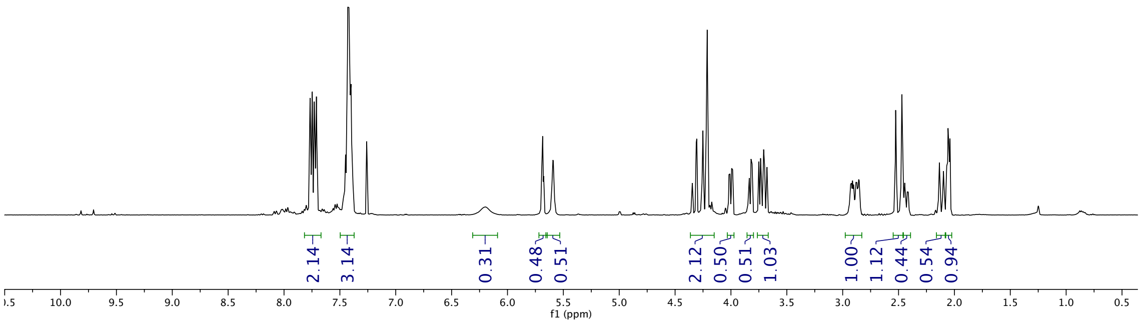

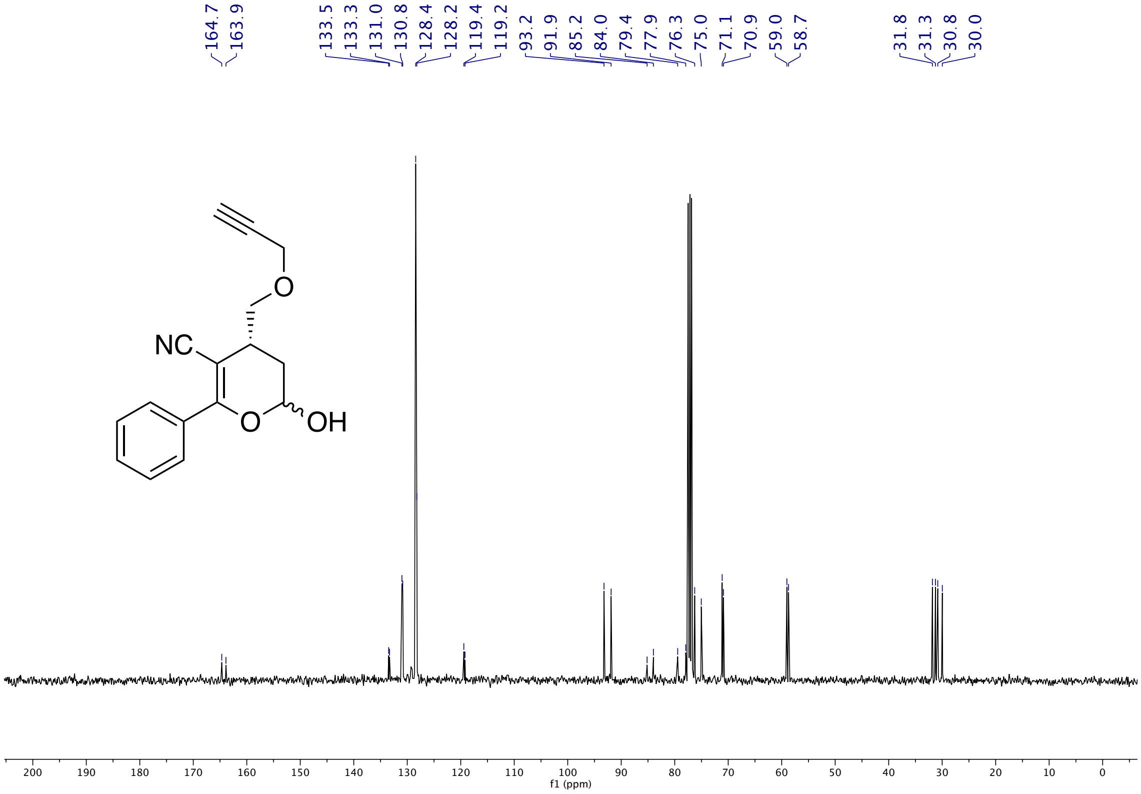

Figure S13. ^{1}H and ^{13}C NMR spectra in $CDCl_3$ of compound **1h.**

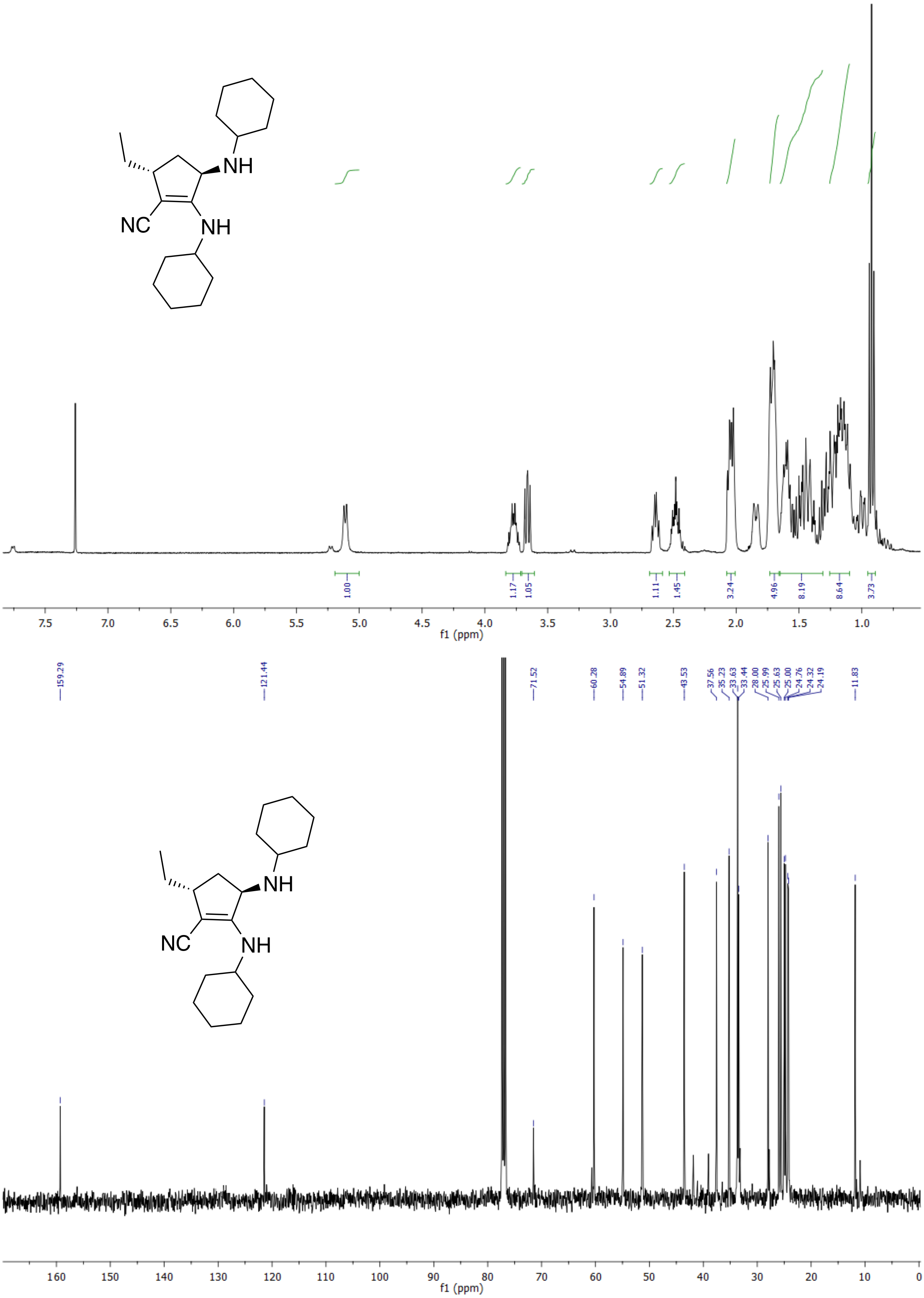

Figure S14.^{1}H and ^{13}C NMR spectra in $CDCl_3$ of compound **2a.**

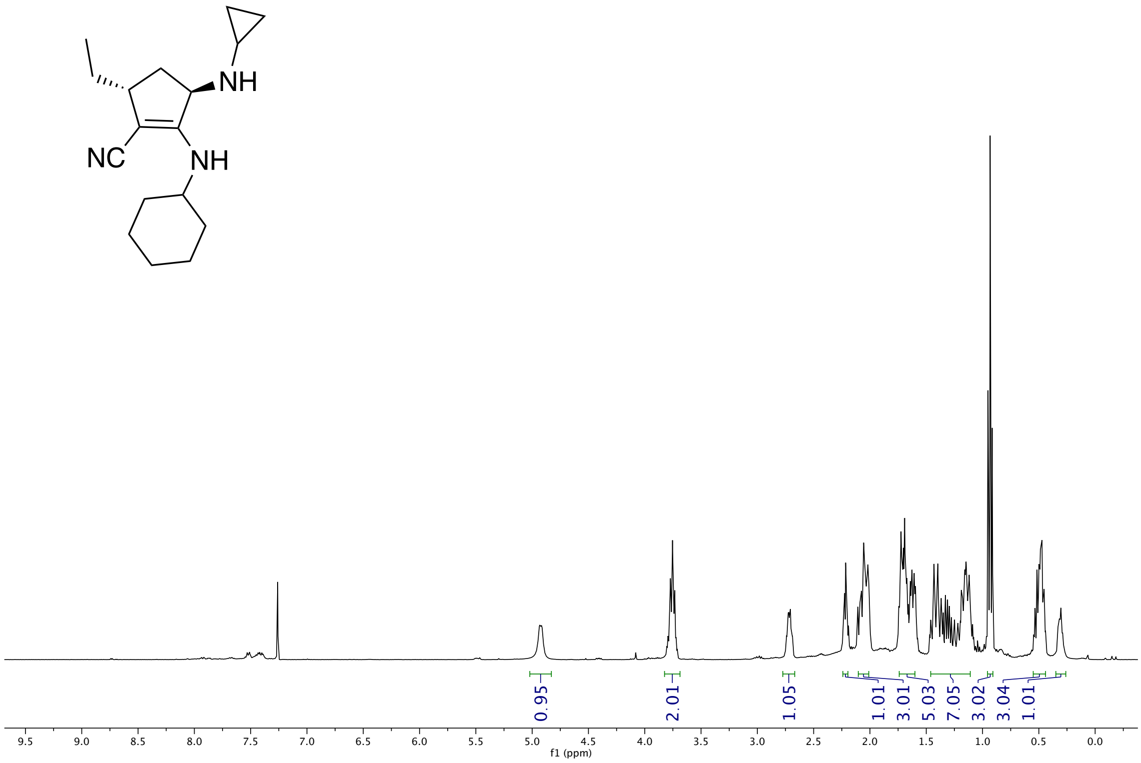

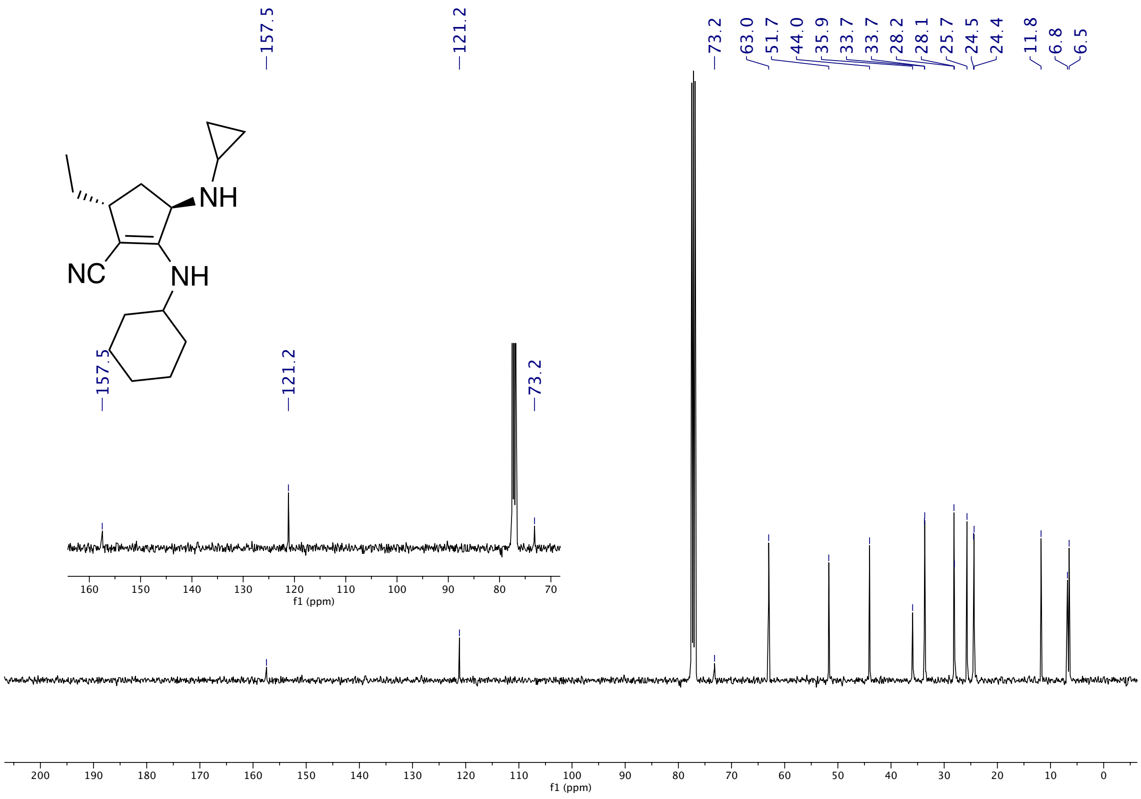

Figure S15. ^{1}H and ^{13}C NMR spectra in $CDCl_3$ of compound **2b.**

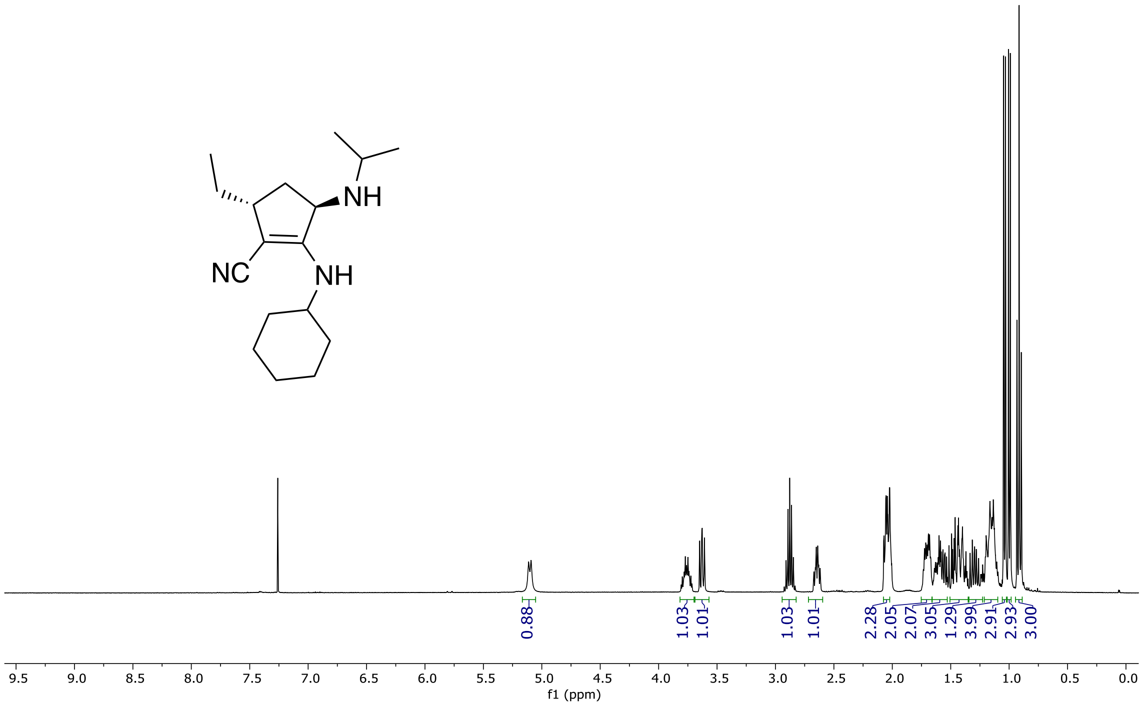

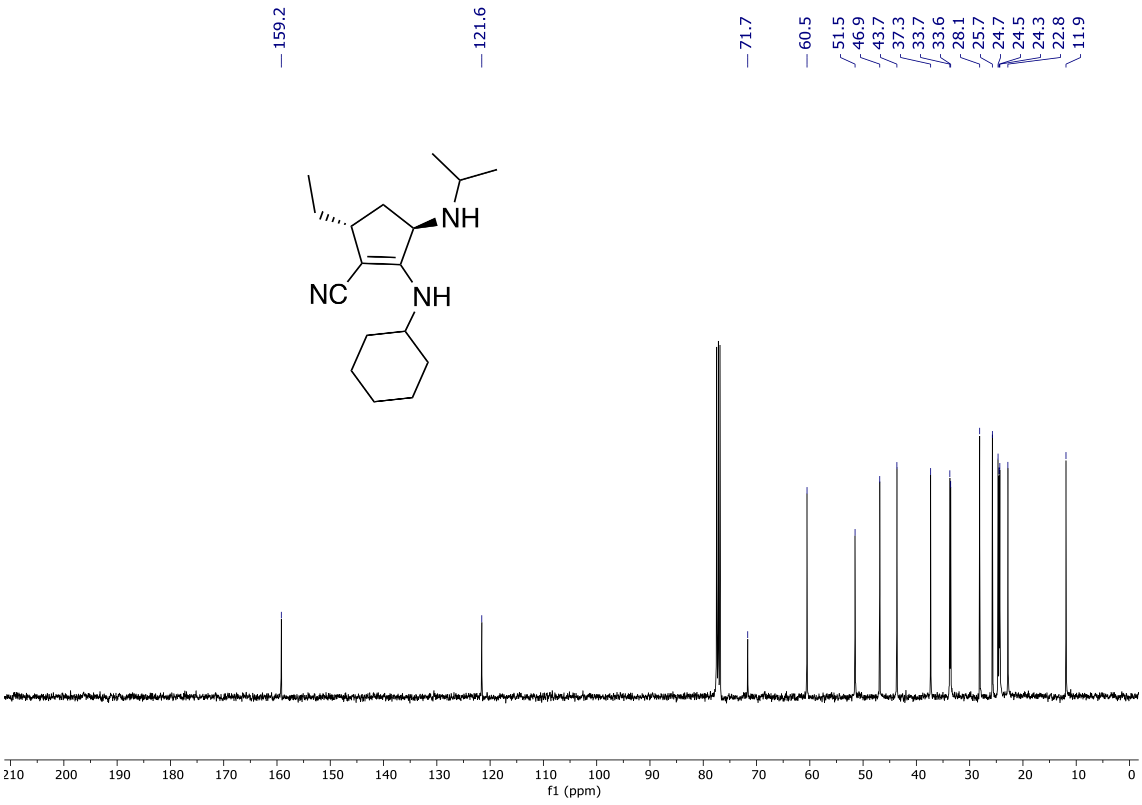

Figure S16. ^{1}H and ^{13}C NMR spectra in $CDCl_3$ of compound **2c**.

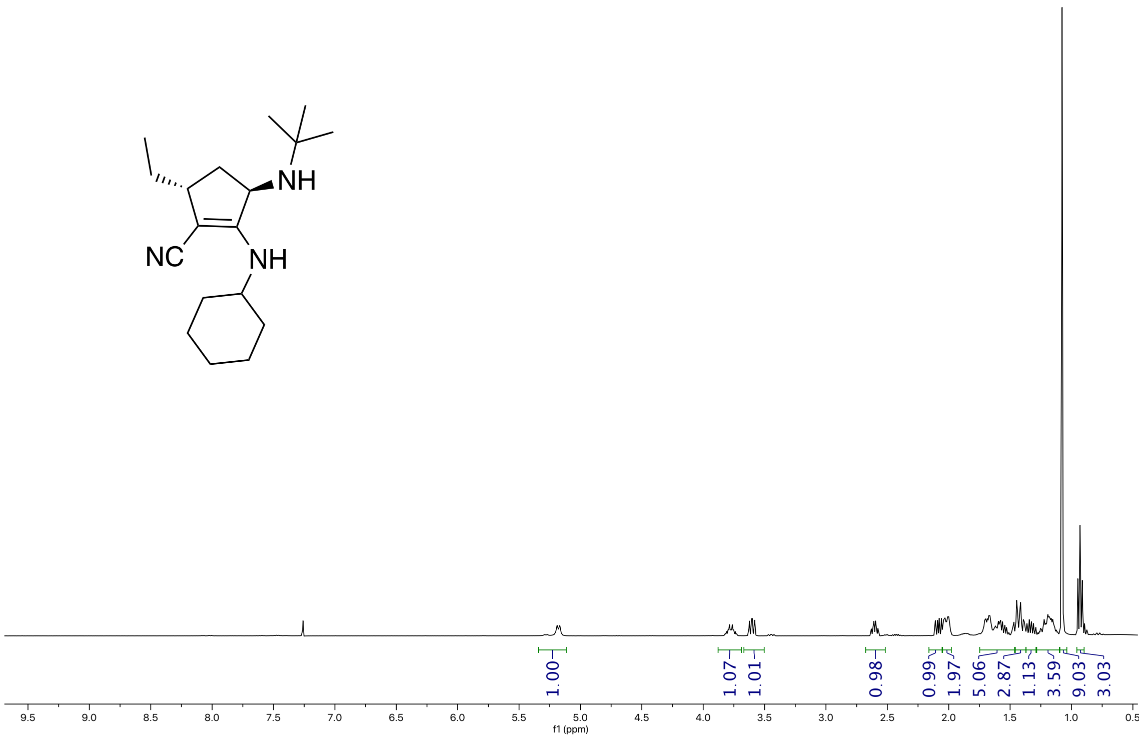

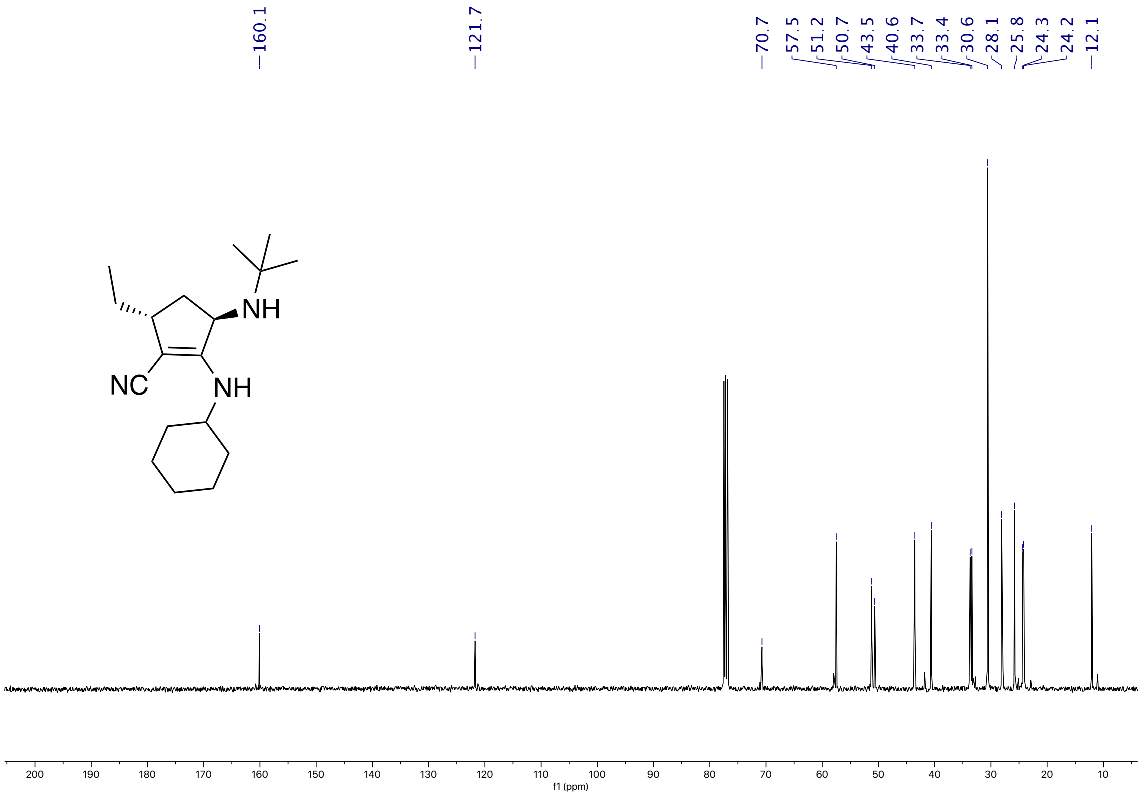

Figure S17. ^{1}H and ^{13}C NMR spectra in $CDCl_3$ of compound **2d**.

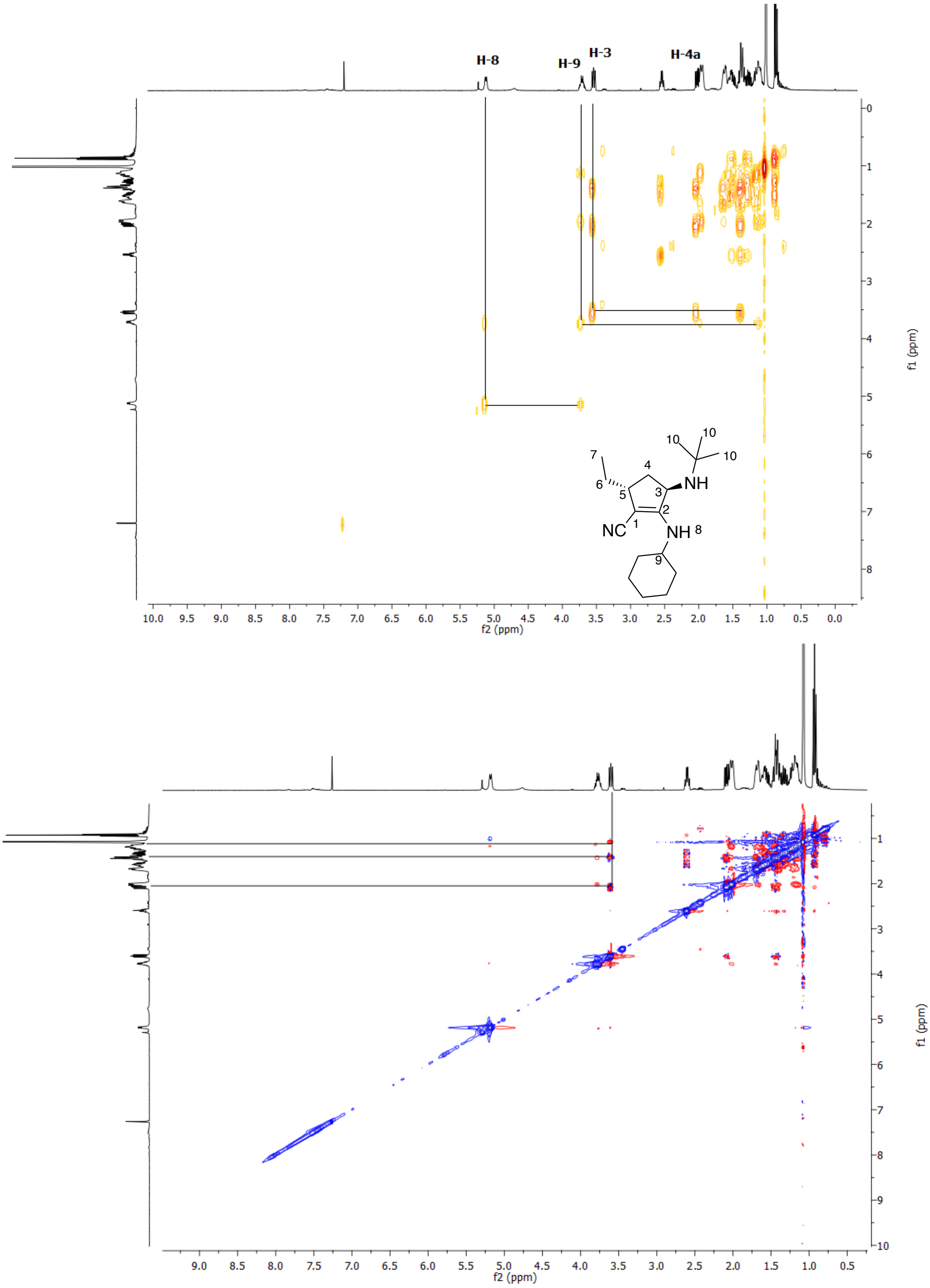

Figure S18. COSY and NOESY NMR spectra in $CDCl_3$ of compound **2d**.

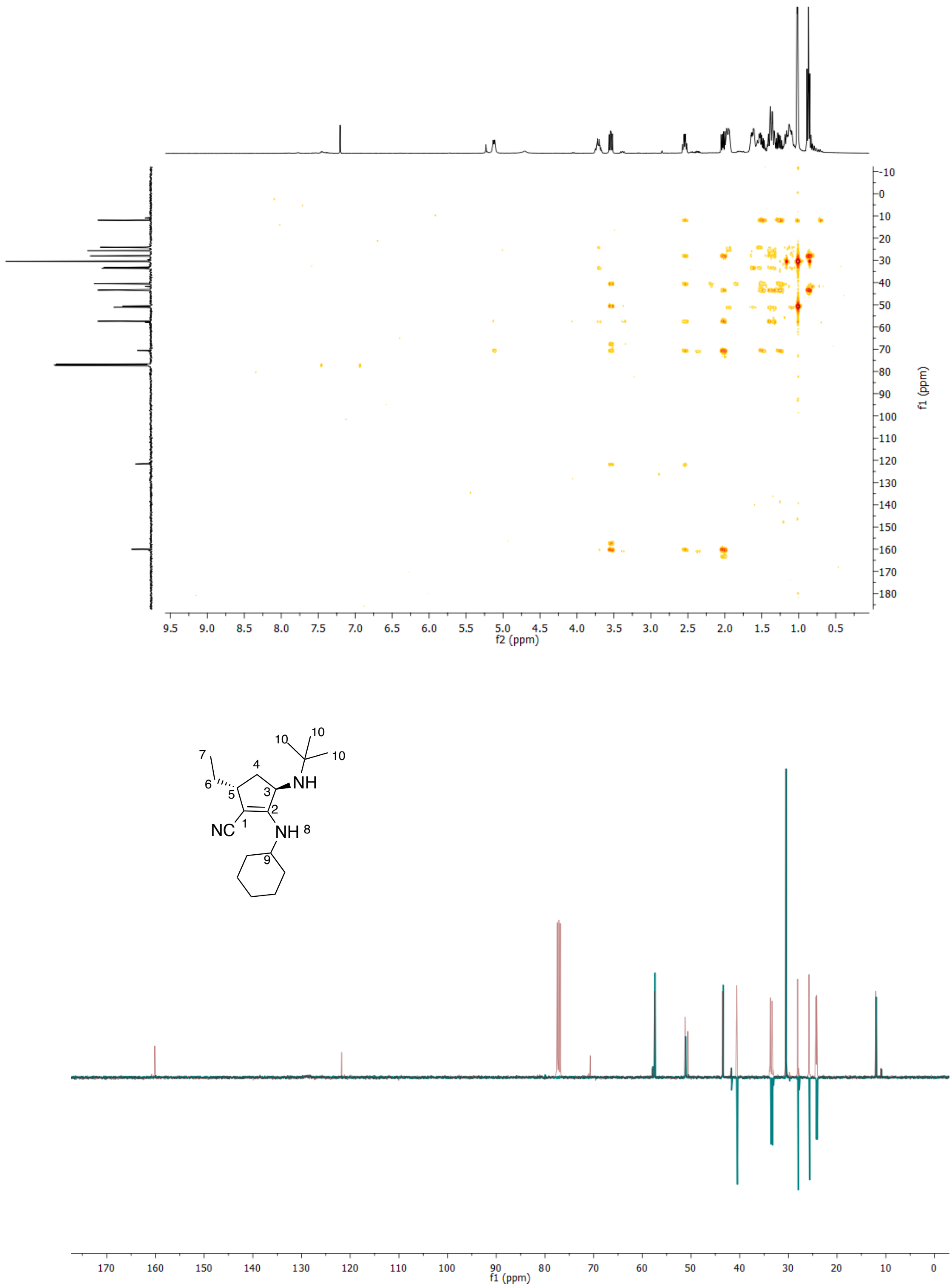

Figure S19. HMBC and superimposed ^{13}C-DEPT 135 NMR spectra in $CDCl_3$ of compound **2d**.

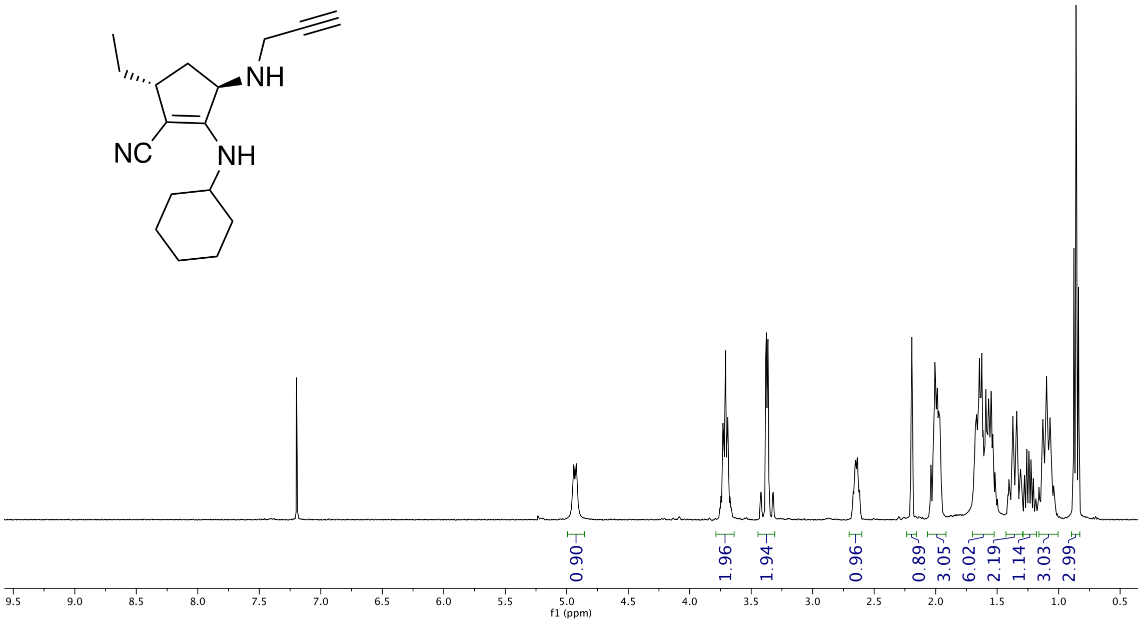

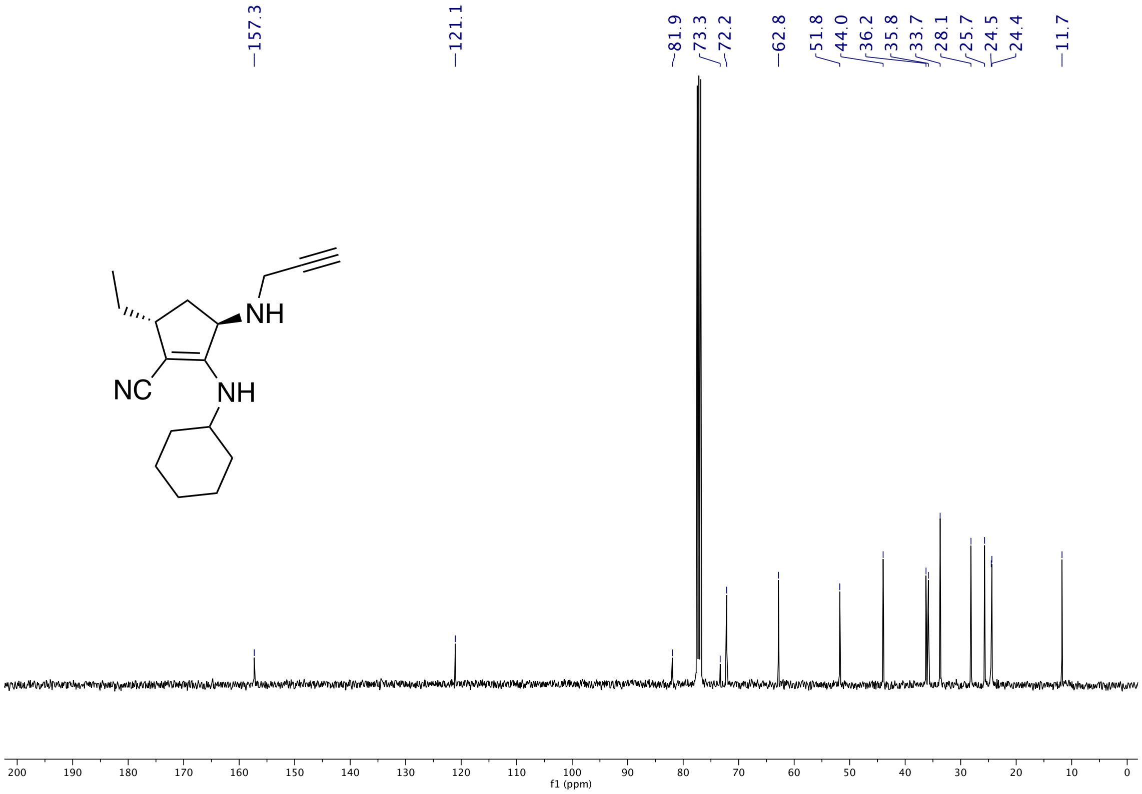

Figure S20. ^{1}H and ^{13}C NMR spectra in $CDCl_3$ of compound **2e**.

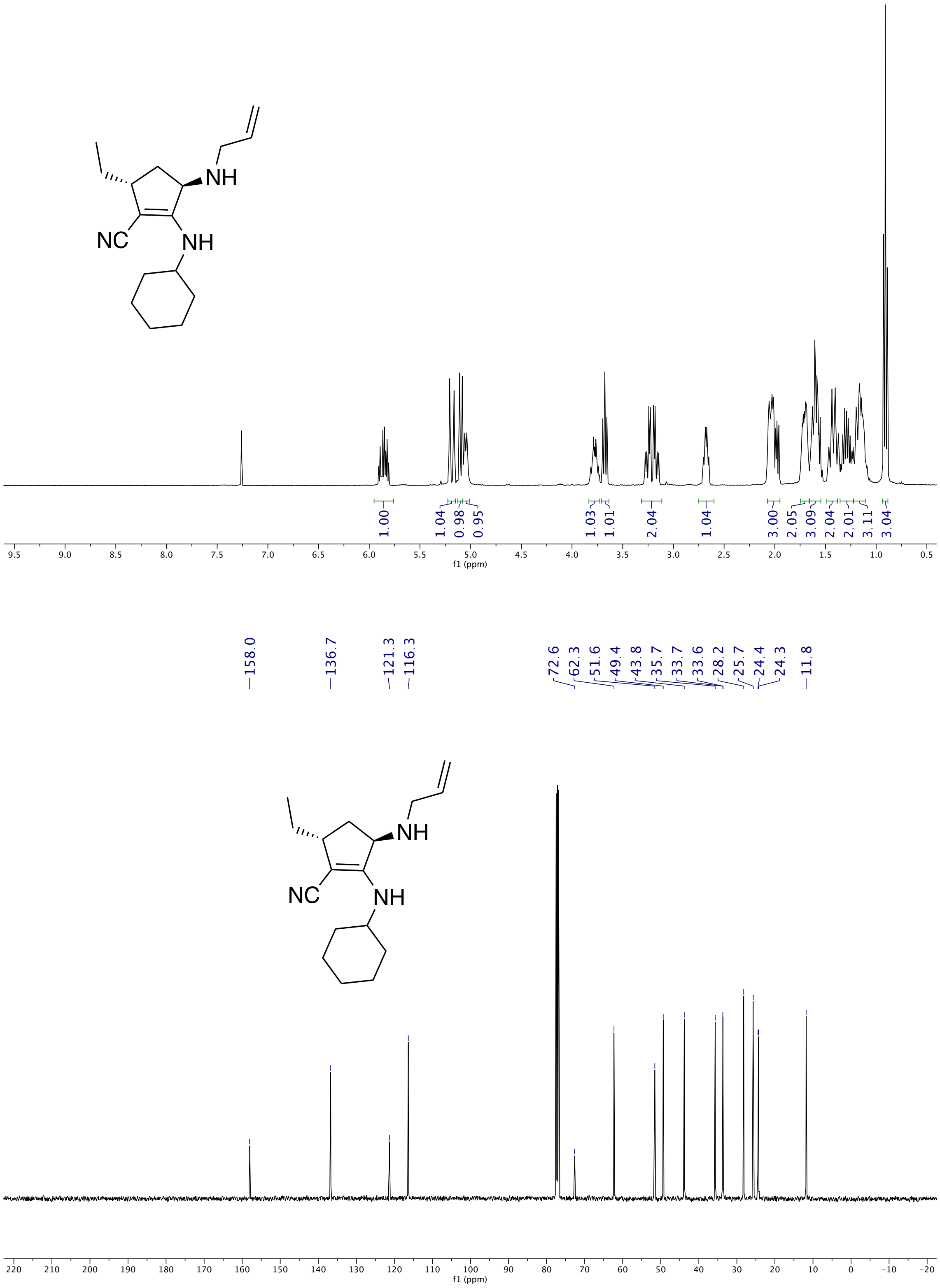

Figure S21. ^{1}H and ^{13}C NMR spectra in $CDCl_3$ of compound **2f**.

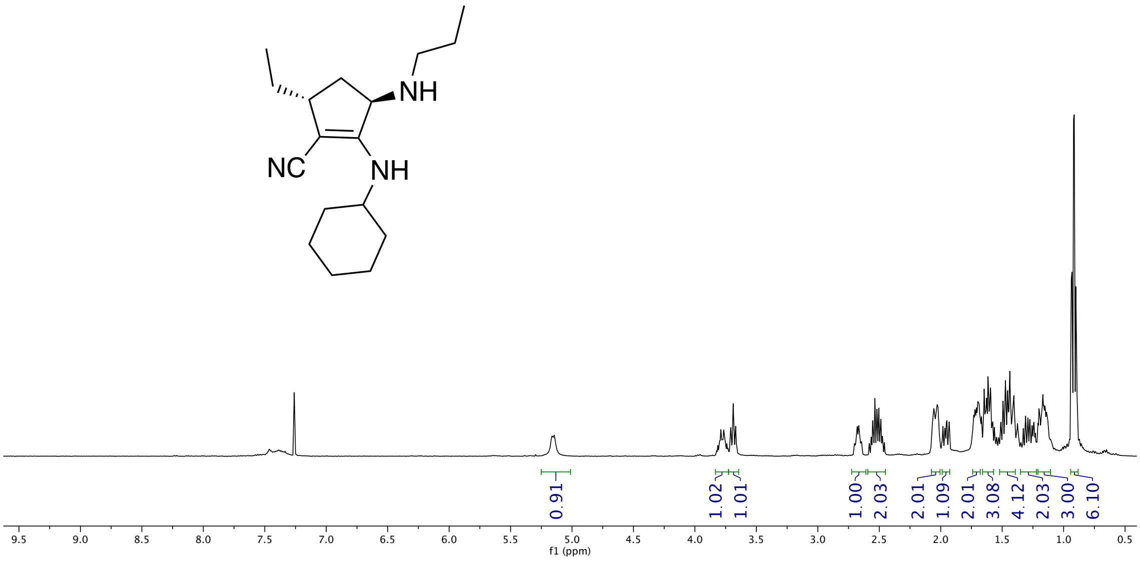

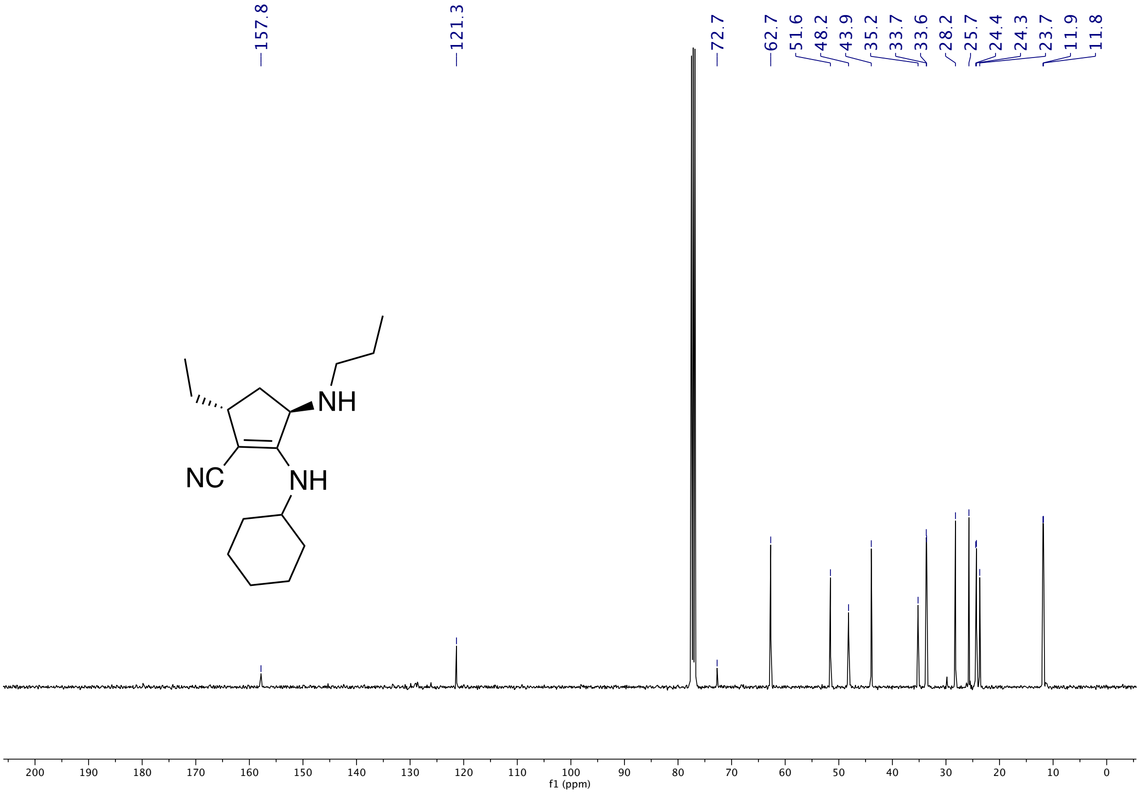

Figure S22. ^{1}H and ^{13}C NMR spectra in $CDCl_3$ of compound **2g**.

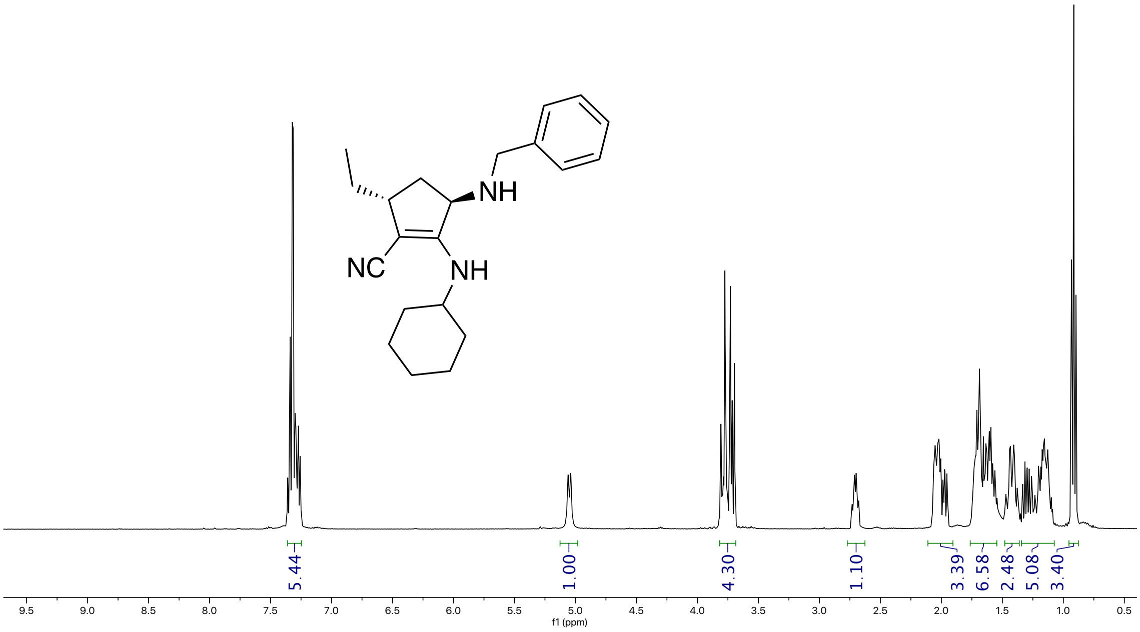

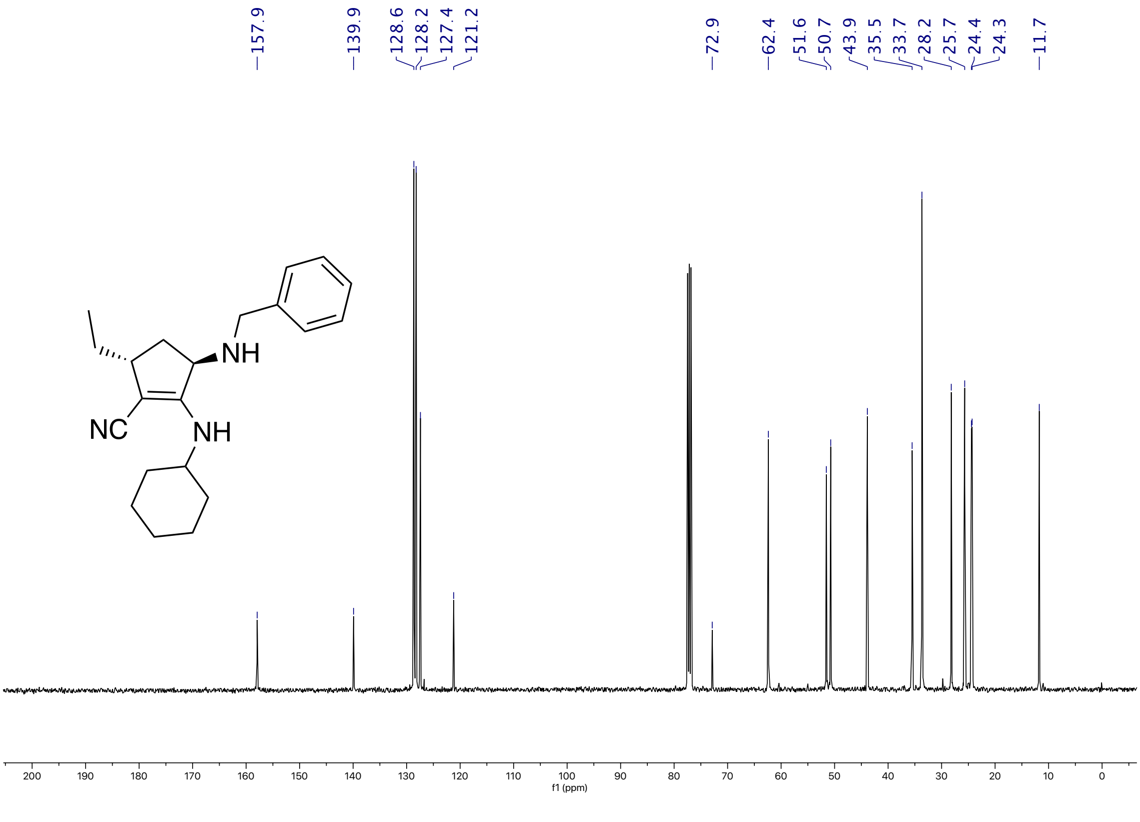

Figure S23. ^1H and ^{13}C NMR spectra in $CDCl_3$ of compound **2h**.

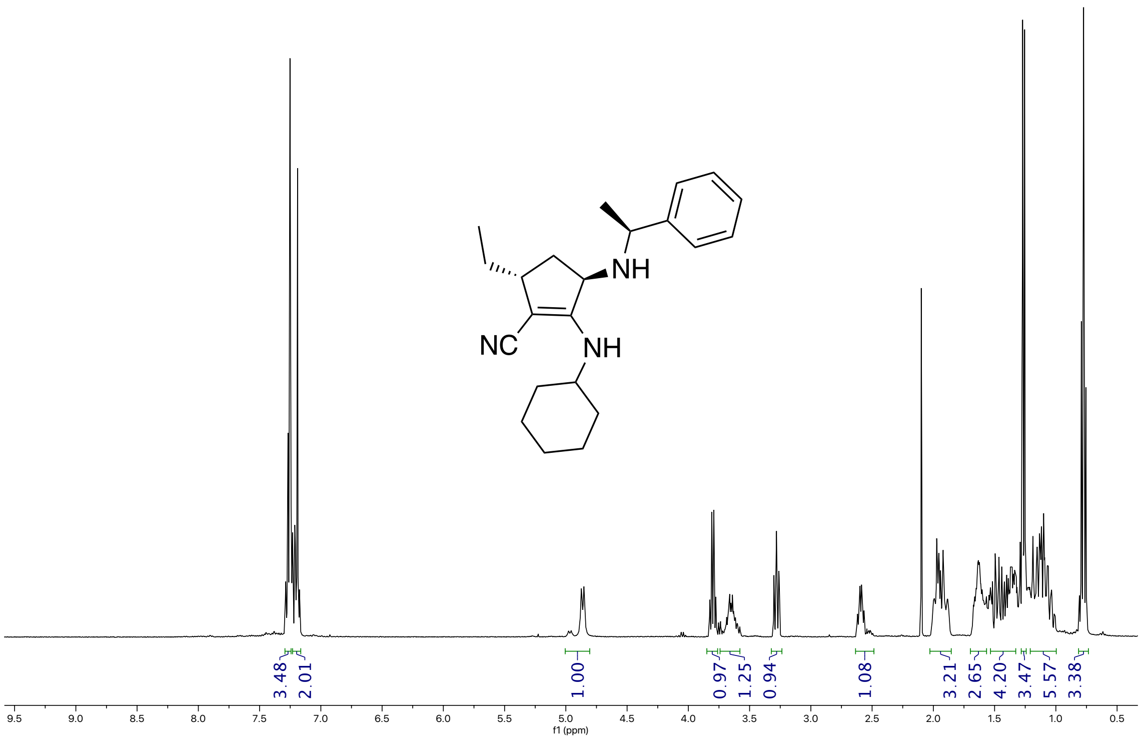

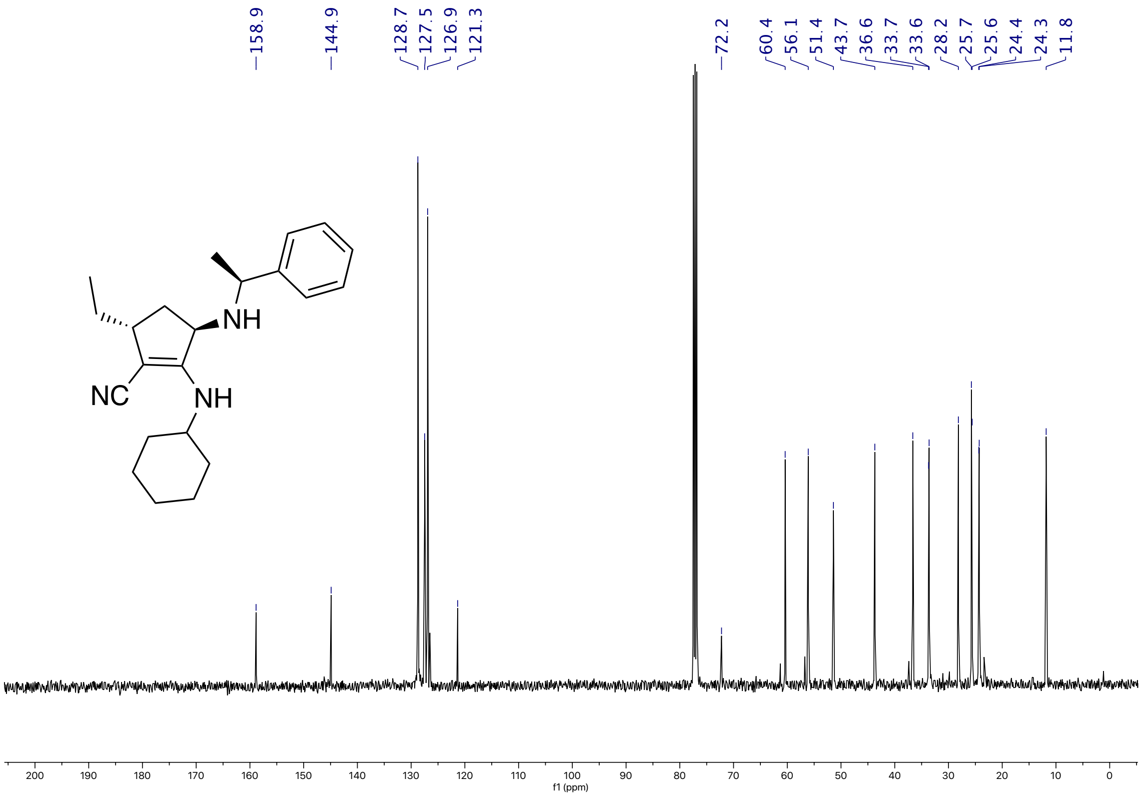

Figure S24. ^{1}H and ^{13}C NMR spectra in $CDCl_3$ of compound **2i**.

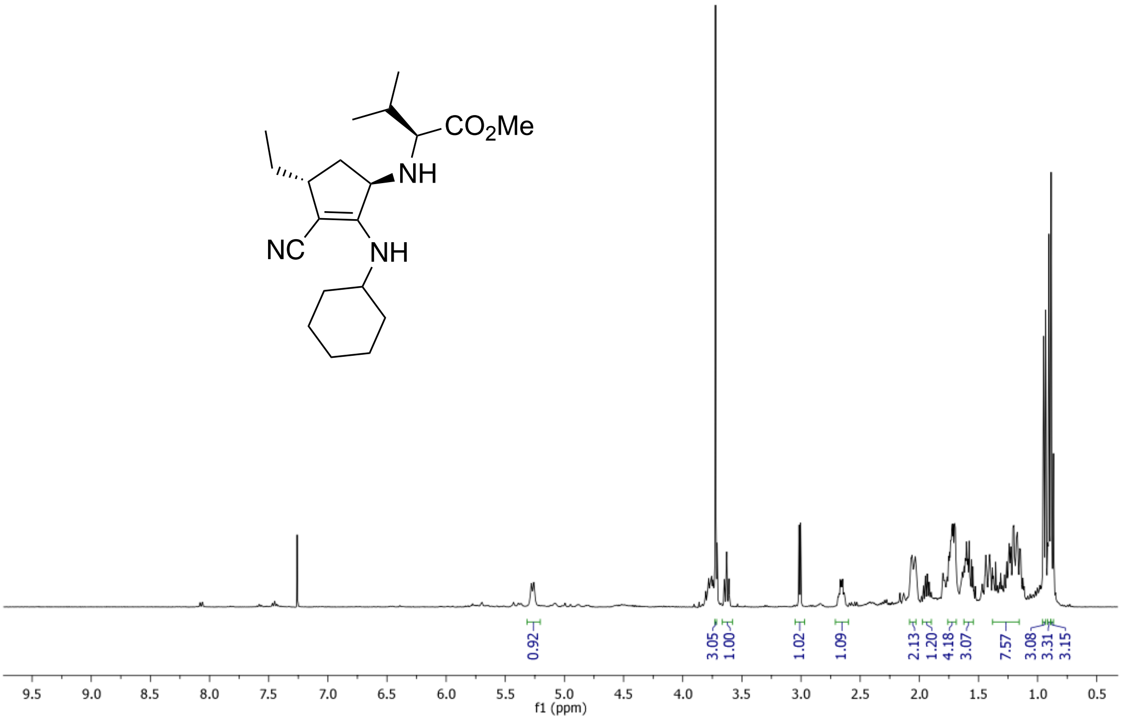

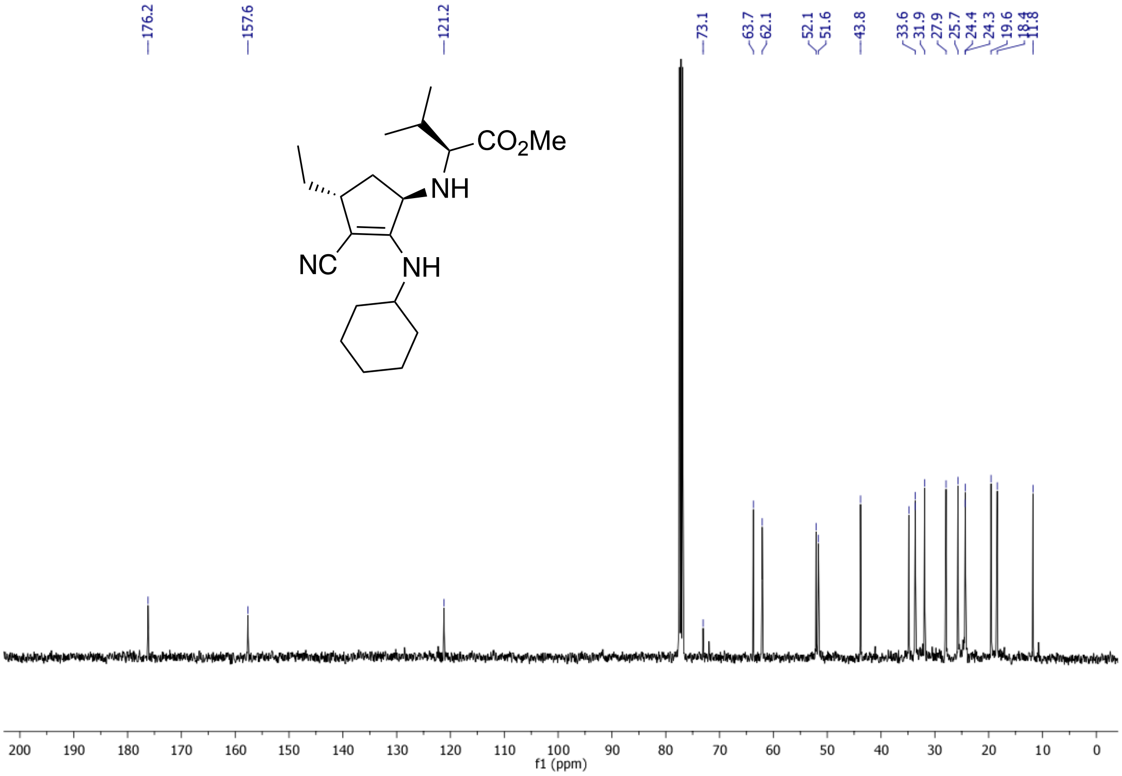

Figure S25. ^{1}H and ^{13}C NMR spectra in $CDCl_3$ of compound **2j**.

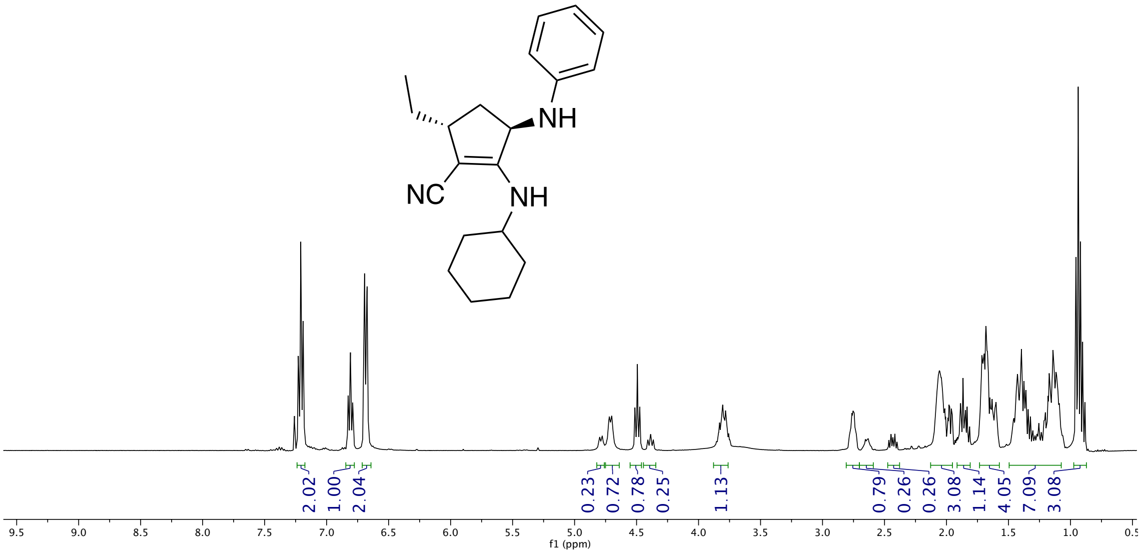

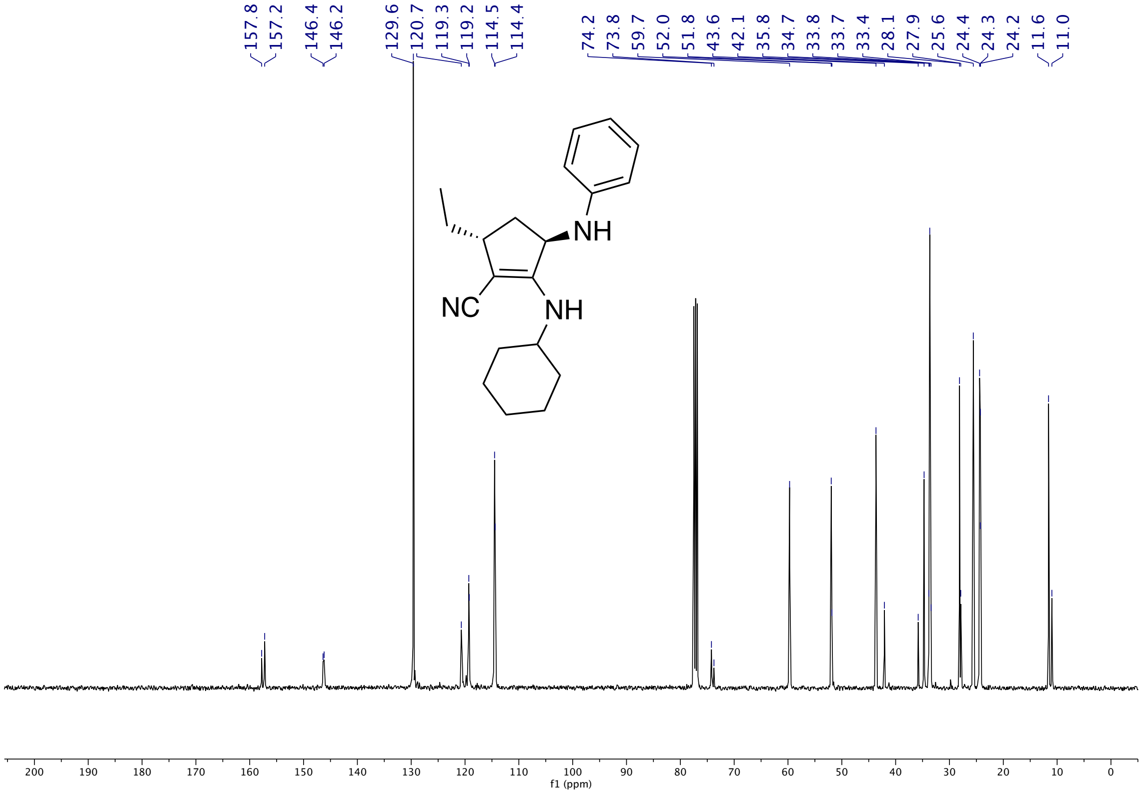

Figure S26. ^{1}H and ^{13}C NMR spectra in $CDCl_3$ of compound **2k**.

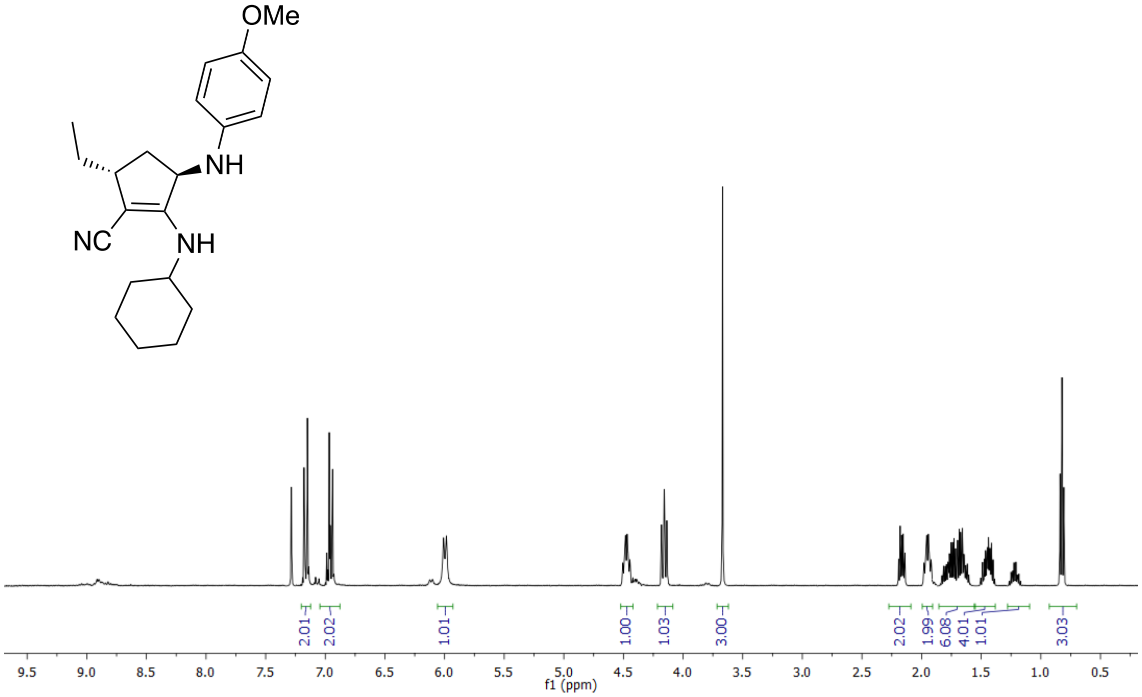

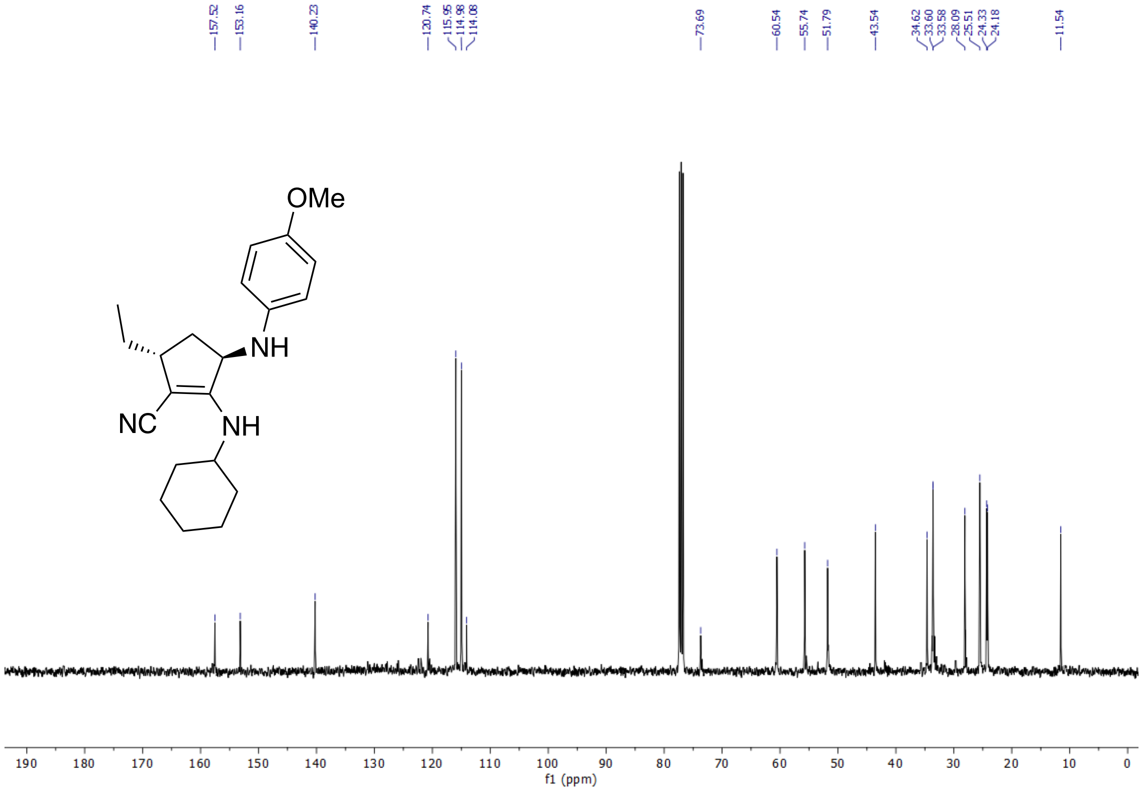

Figure S27. ^{1}H and ^{13}C NMR spectra in $CDCl_3$ of compound **2l**.

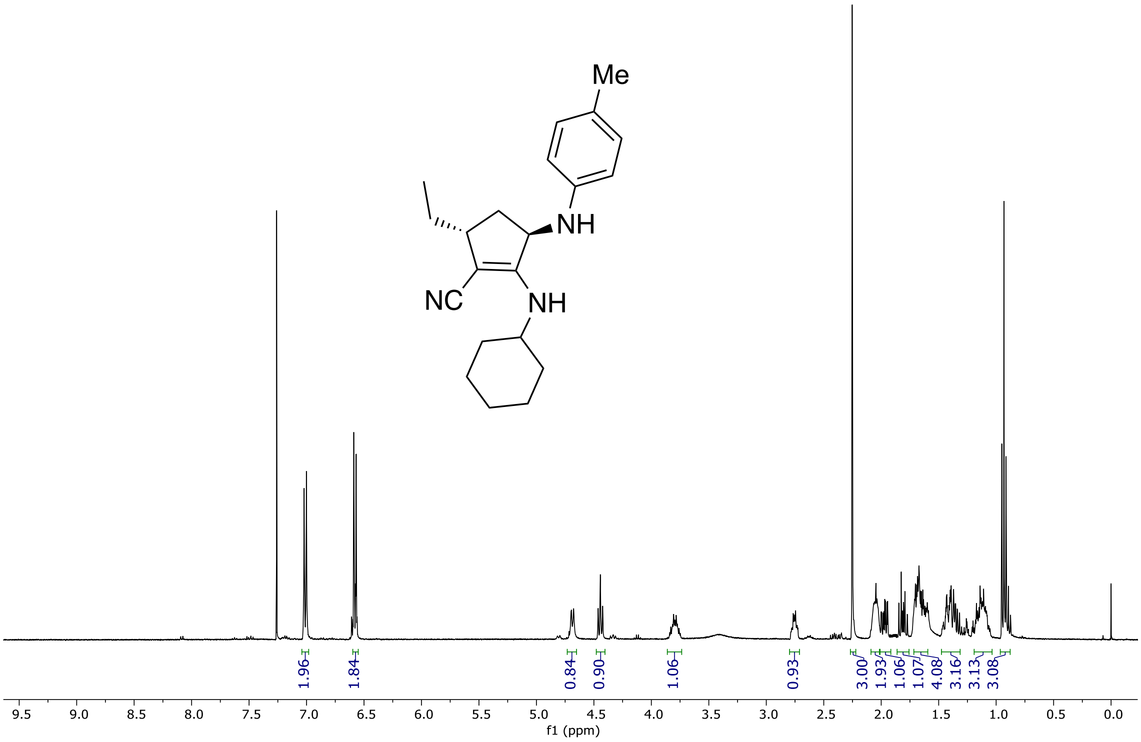

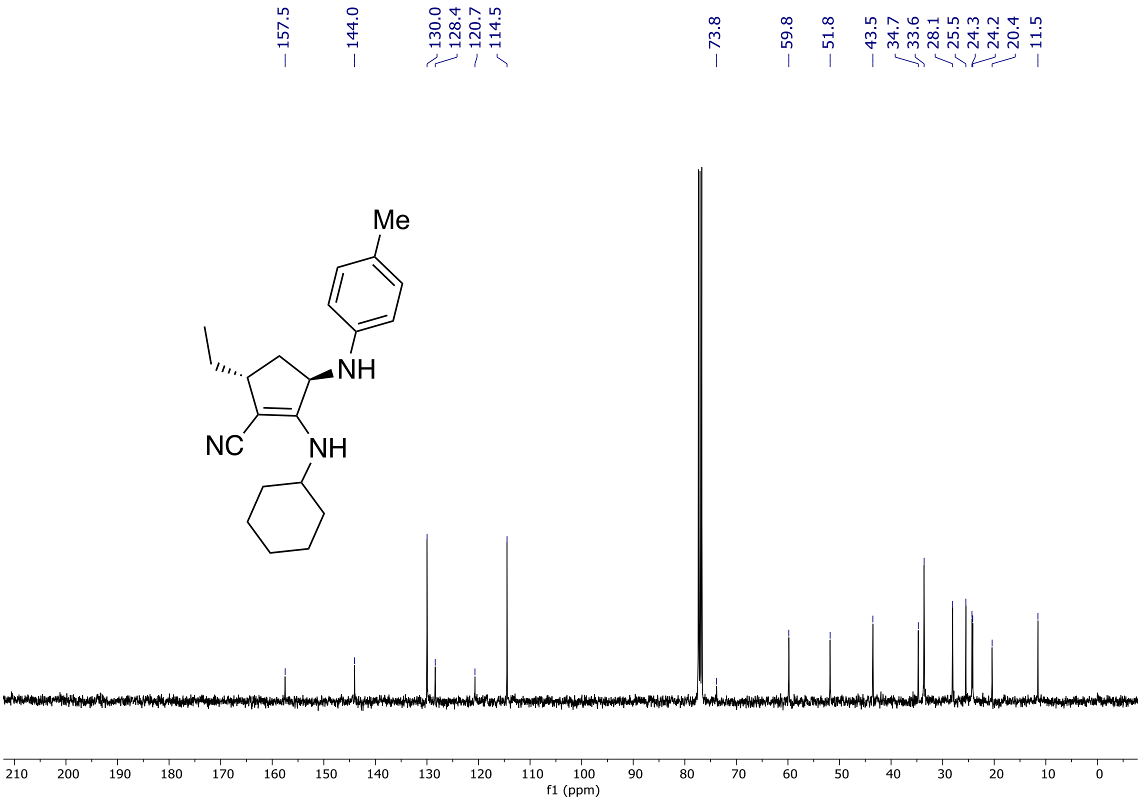

Figure S28. ^{1}H and ^{13}C NMR spectra in $CDCl_3$ of compound **2m**.

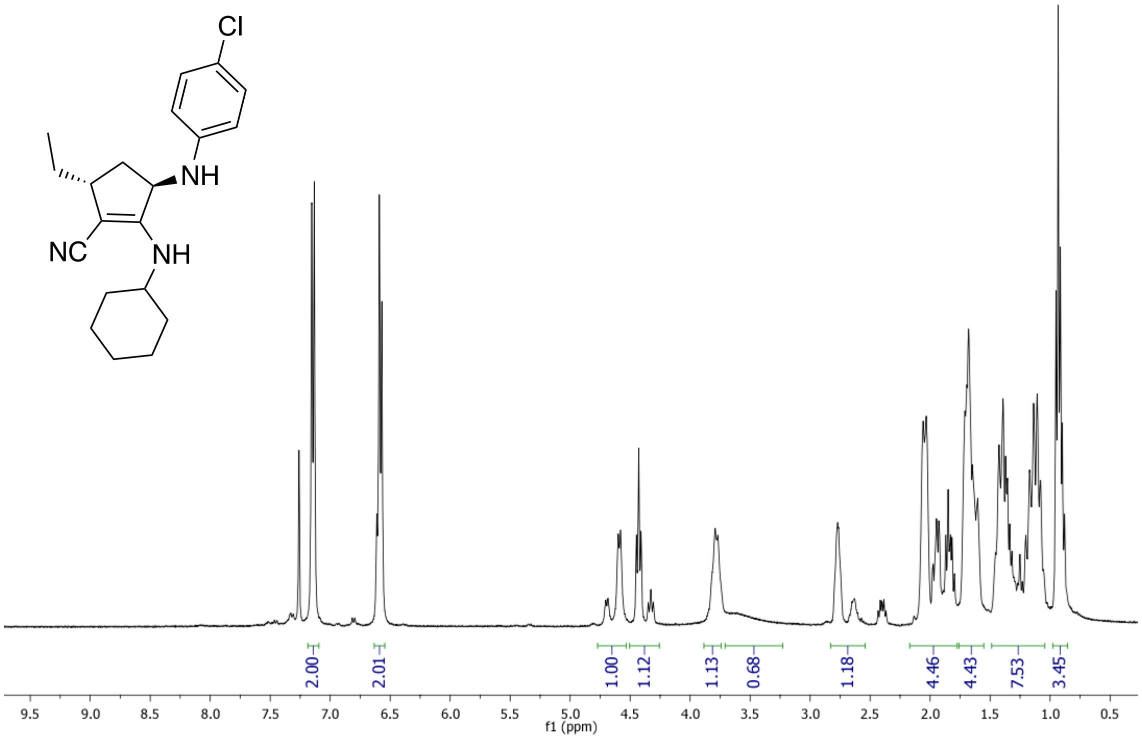

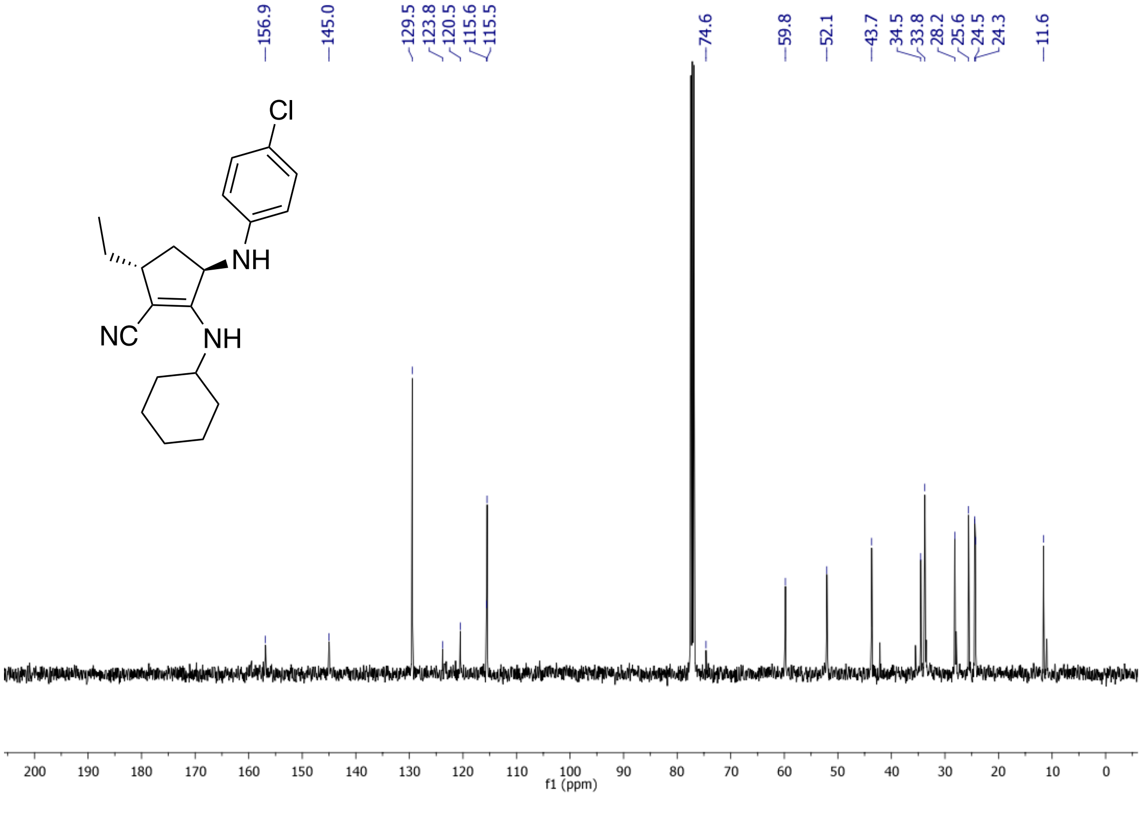

Figure S29. ^{1}H and ^{13}C NMR spectra in $CDCl_3$ of compound **2n**.

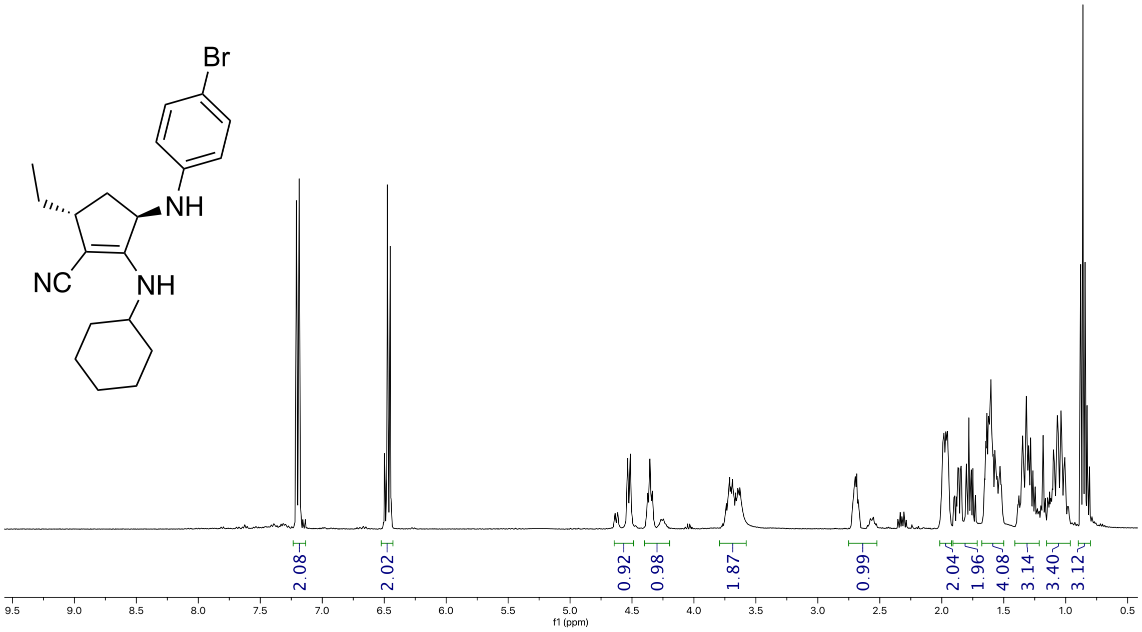

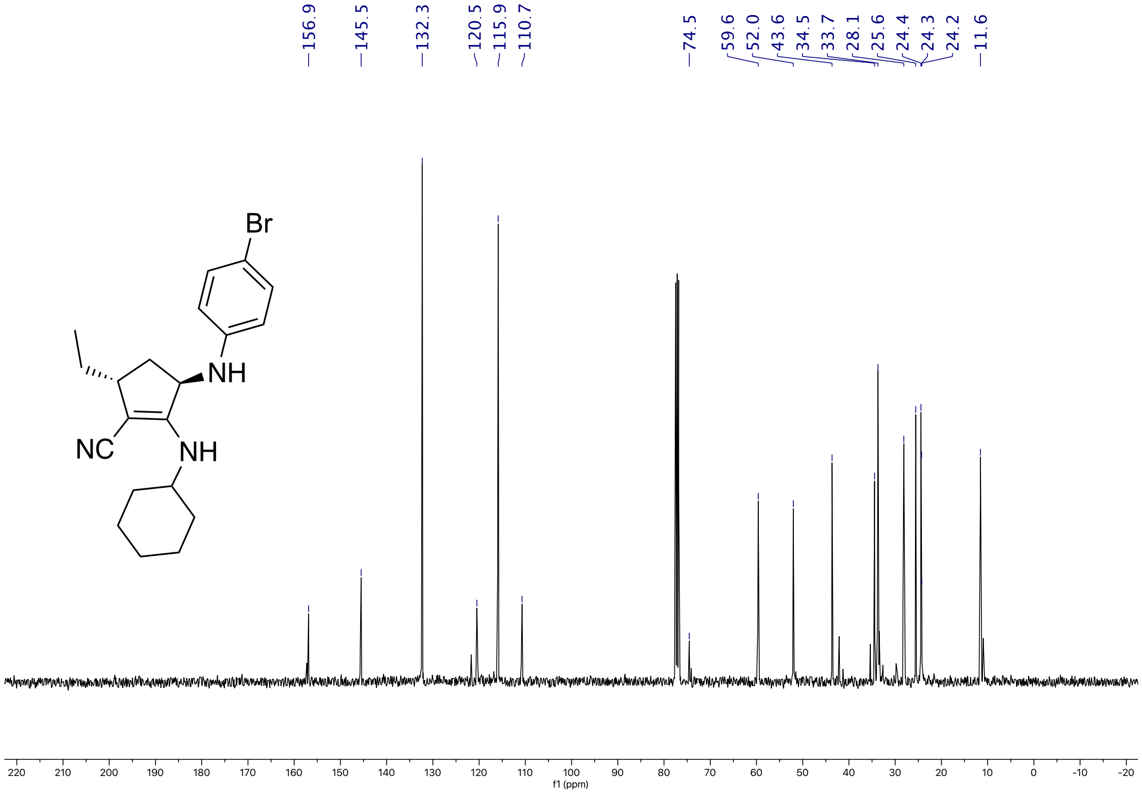

Figure S30. ^{1}H and ^{13}C NMR spectra in $CDCl_3$ of compound **2o.**

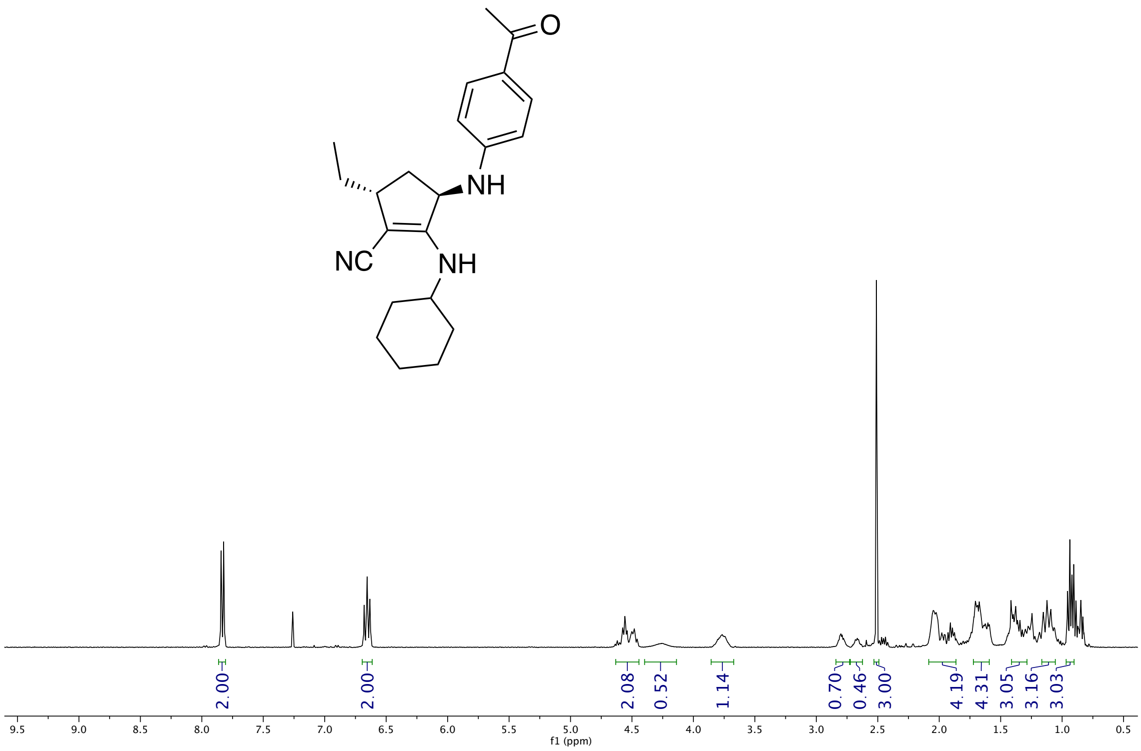

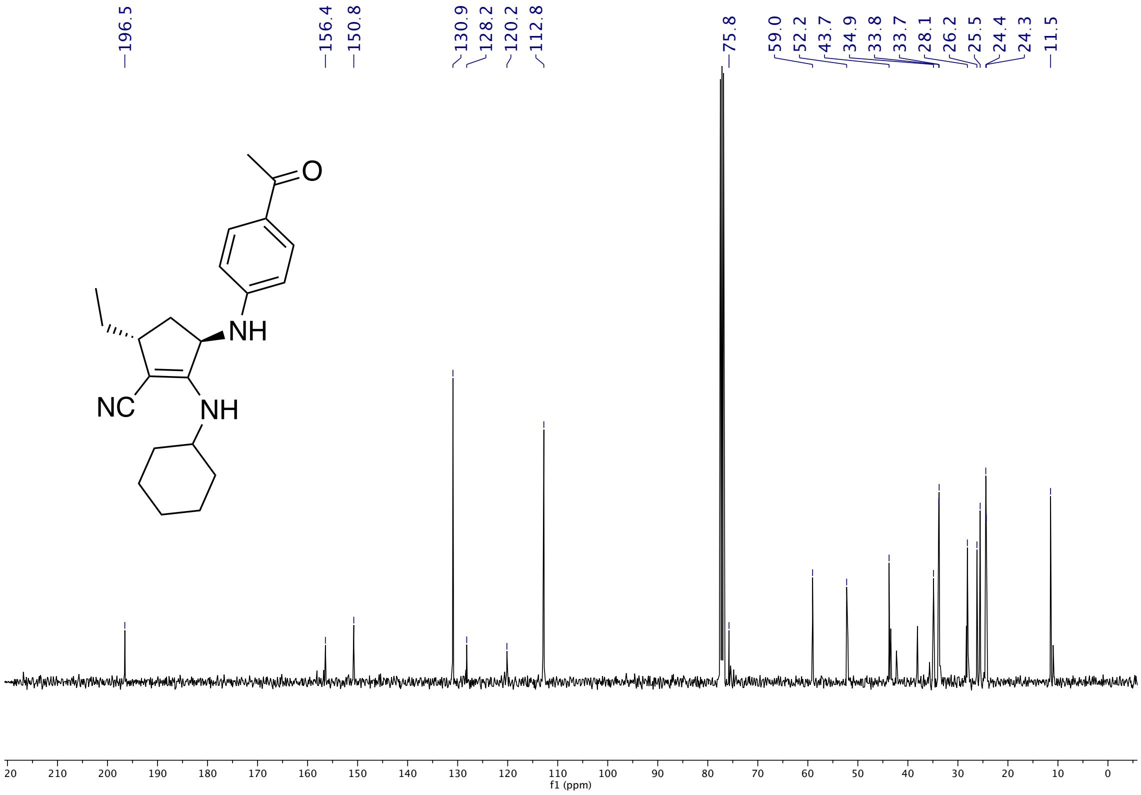

Figure S31. ^{1}H and ^{13}C NMR spectra in $CDCl_3$ of compound **2p.**

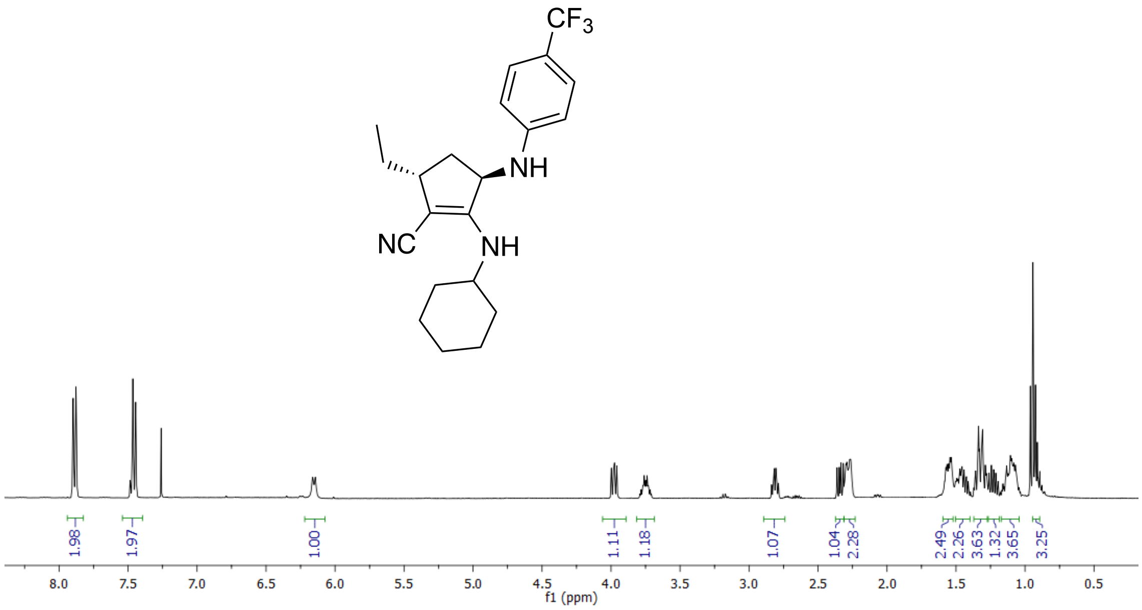

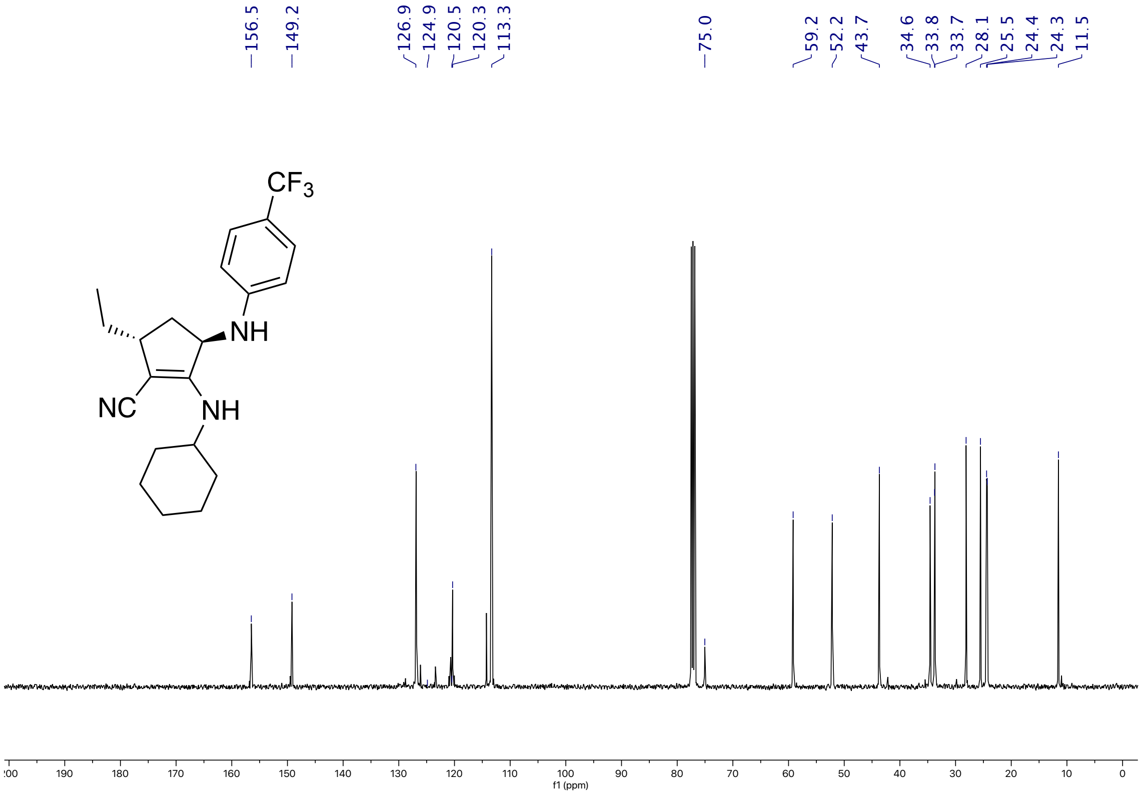

Figure S32. ^{1}H and ^{13}C NMR spectra in $CDCl_3$ of compound **2q**.

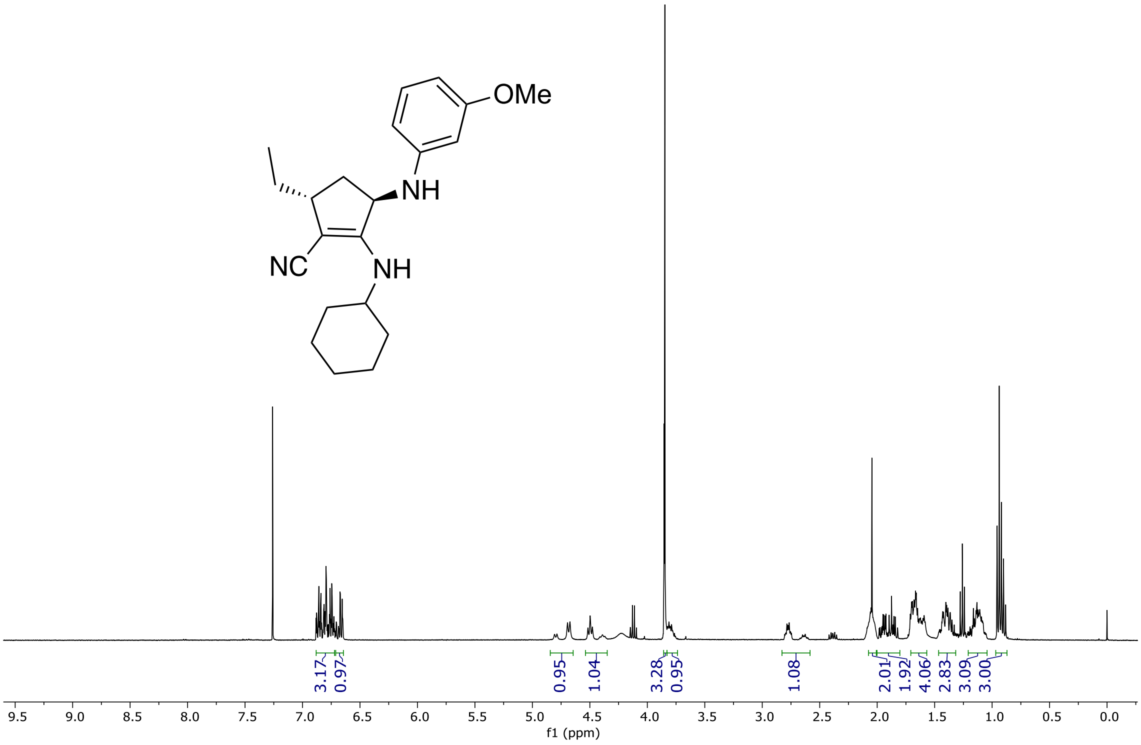
OMe
NH
NC
NH
3.17
0.97
0.95
1.04
3.28
0.95
1.08
2.01
1.92
4.06
2.83
3.09
3.00
9.5 9.0 8.5 8.0 7.5 7.0 6.5 6.0 5.5 5.0 4.5 4.0 3.5 3.0 2.5 2.0 1.5 1.0 0.5 0.0
f1 (ppm)

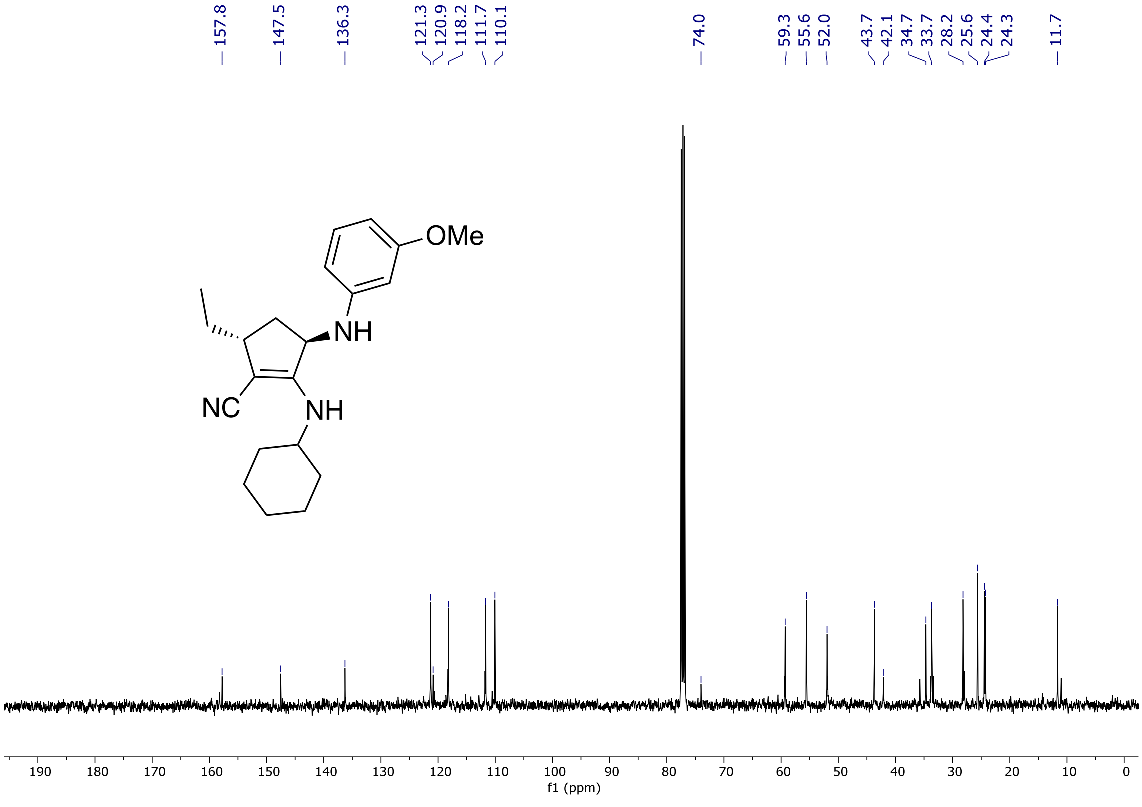
157.8
147.5
136.3
121.3
120.9
118.2
111.7
110.1
74.0
59.3
55.6
52.0
43.7
42.1
34.7
33.7
28.2
25.6
24.4
24.3
11.7
OMe
NH
NC
NH
190 180 170 160 150 140 130 120 110 100 90 80 70 60 50 40 30 20 10 0
f1 (ppm)

Figure S33. ^{1}H and ^{13}C NMR spectra in $CDCl_3$ of compound **2s**.

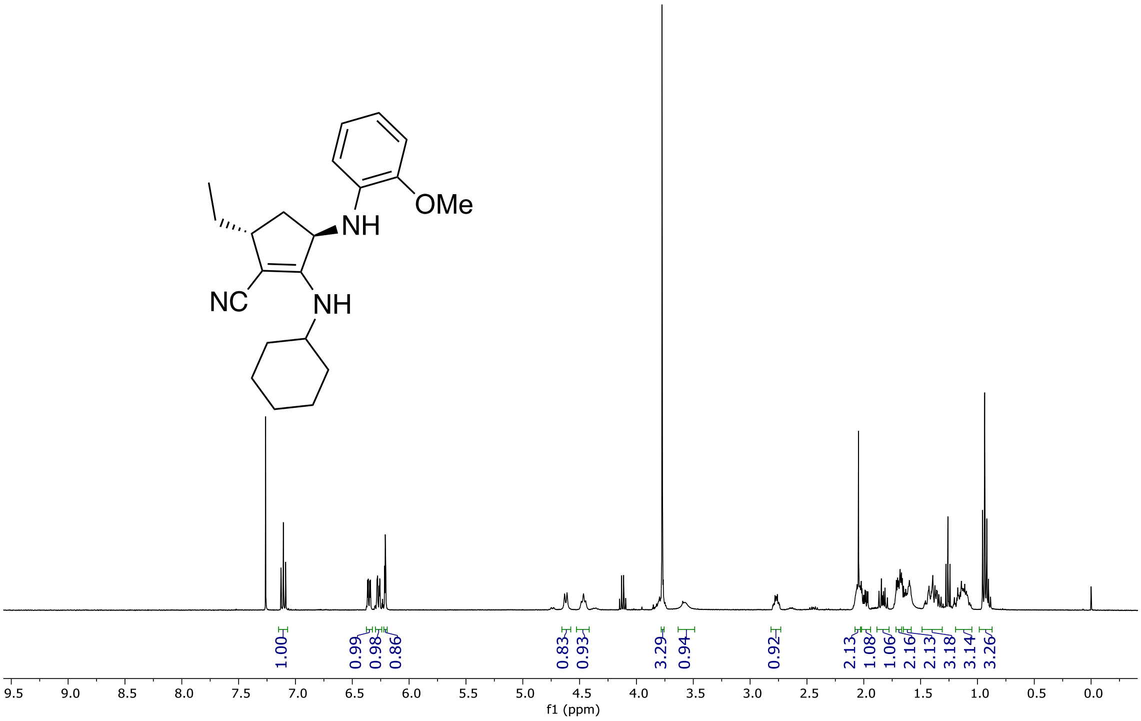

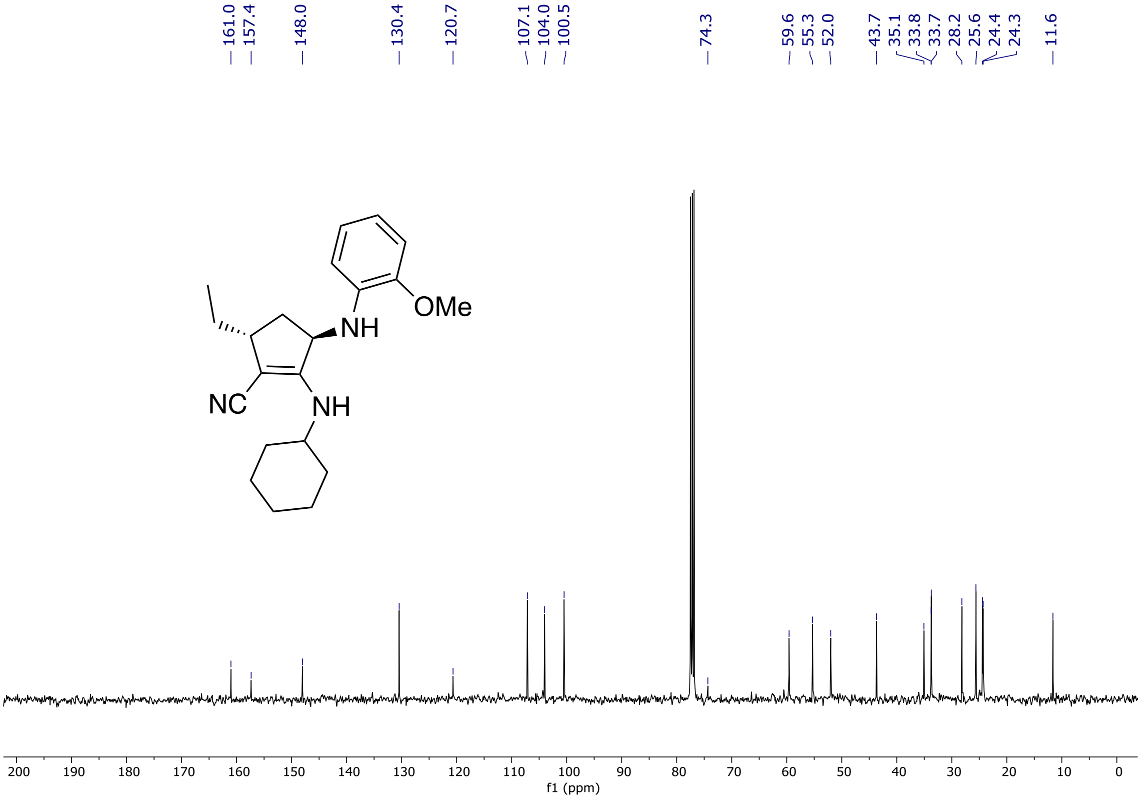

Figure S34. ^{1}H and ^{13}C NMR spectra in $CDCl_3$ of compound **2t**.

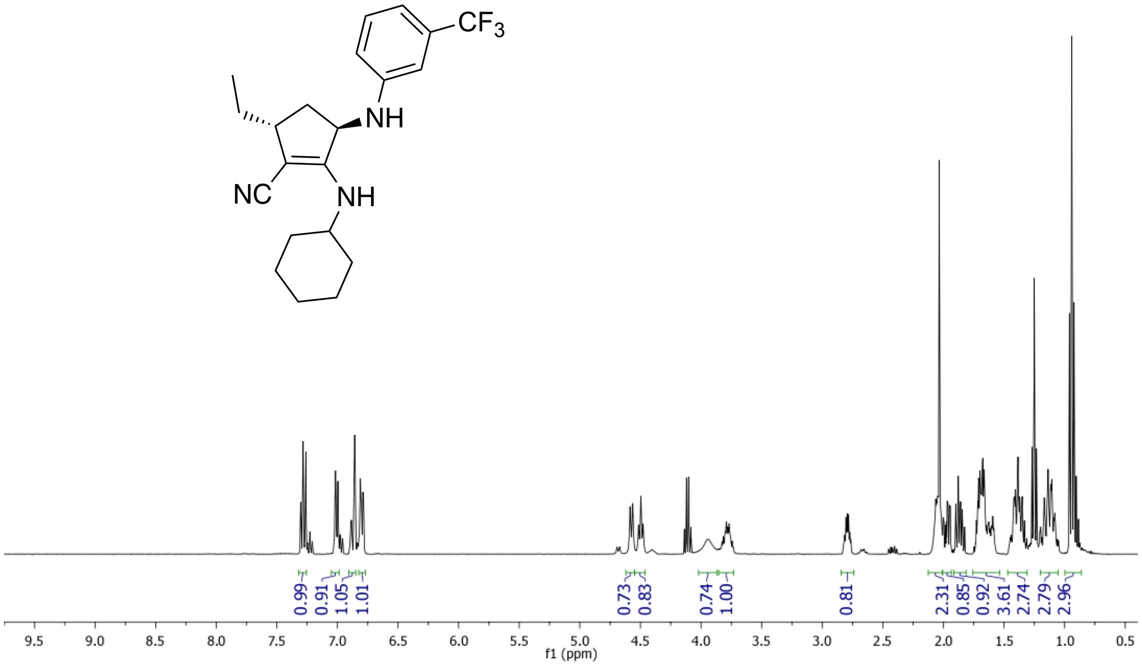

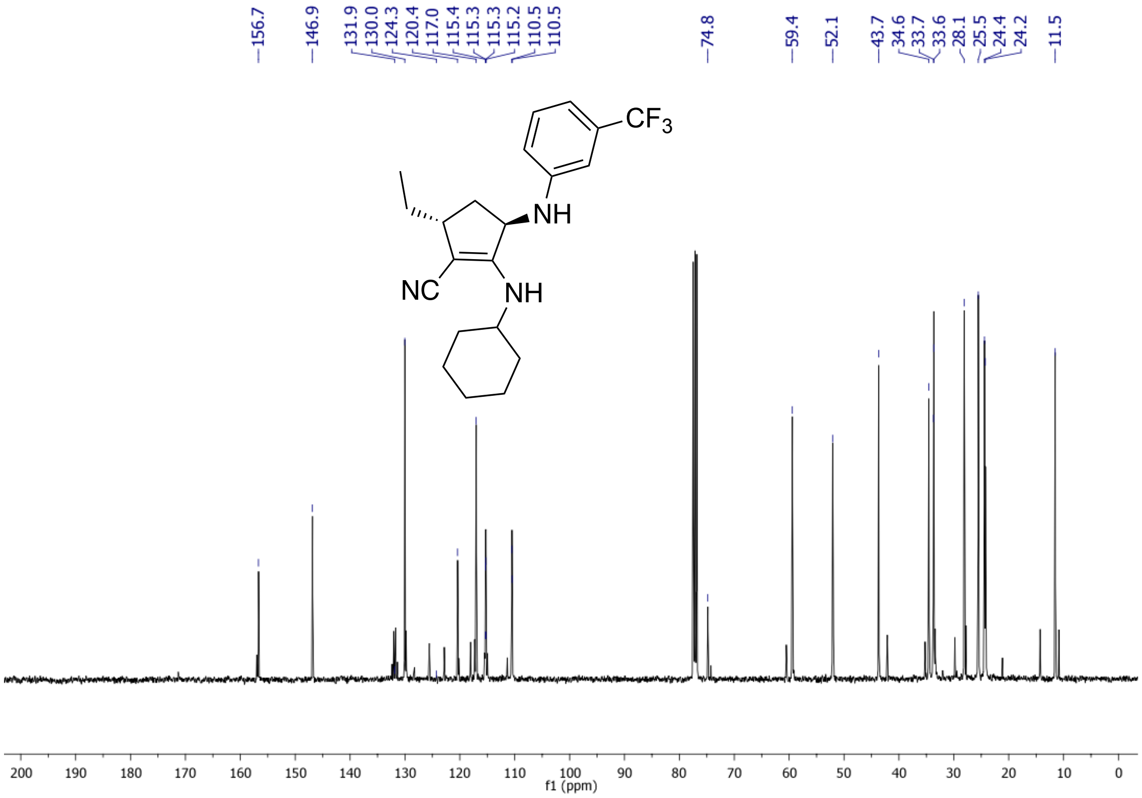

Figure S35. ^{1}H and ^{13}C NMR spectra in $CDCl_3$ of compound **2u.**

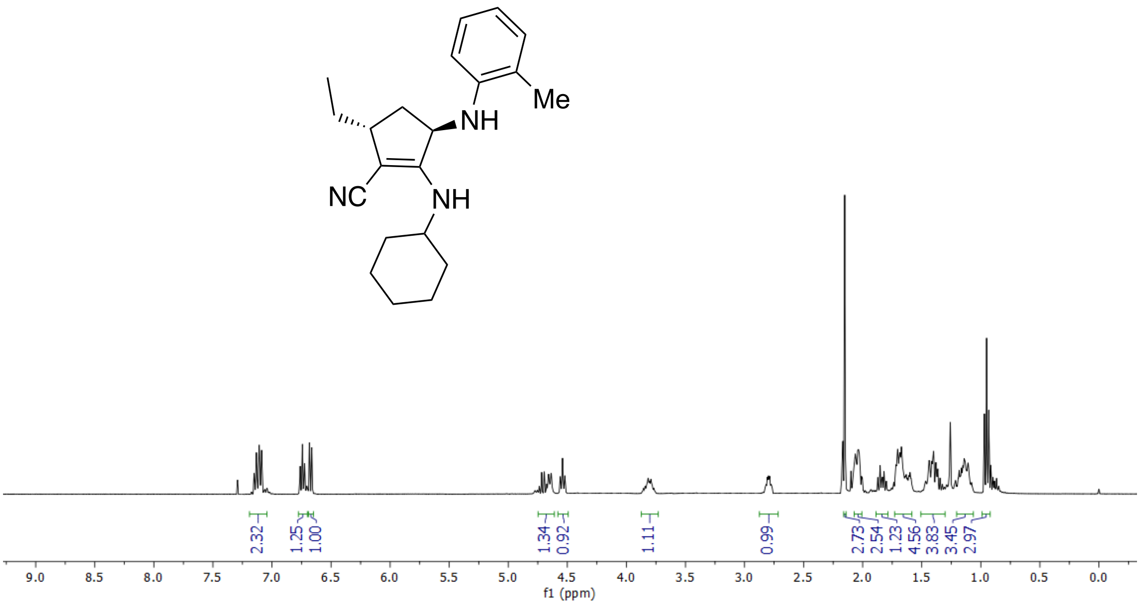

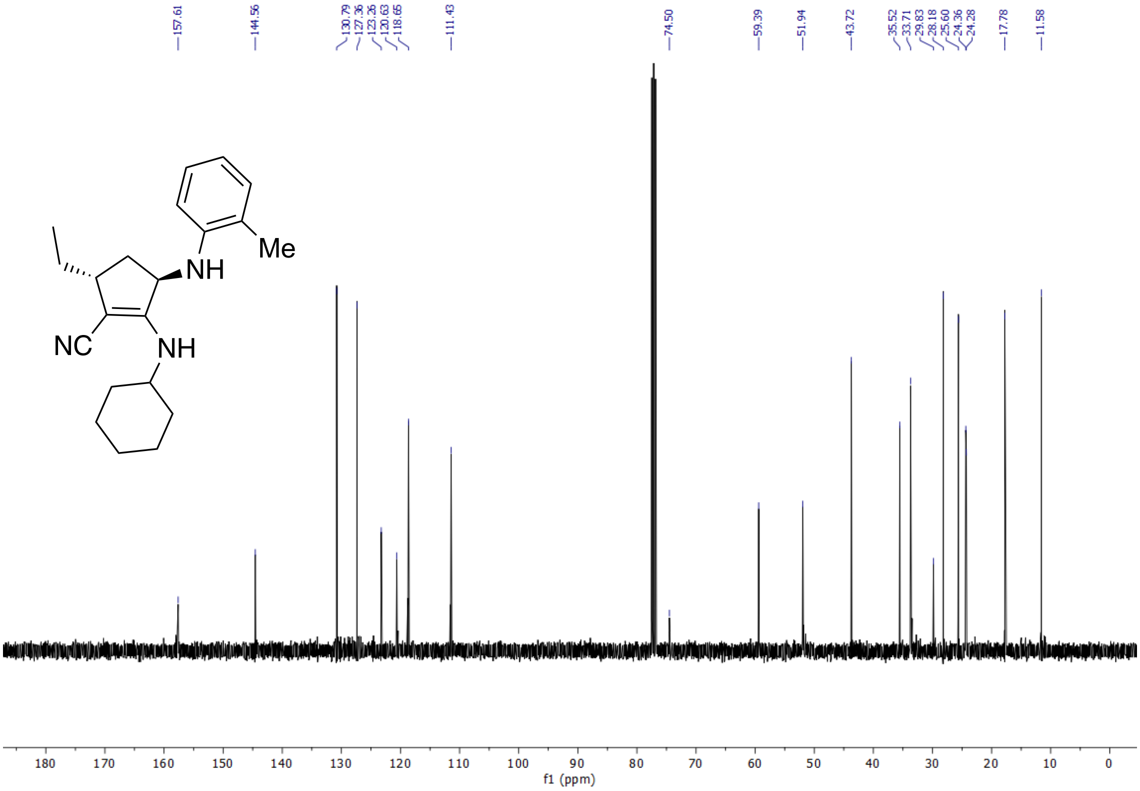

Figure S36. ^{1}H and ^{13}C NMR spectra in $CDCl_3$ of compound **2w.**

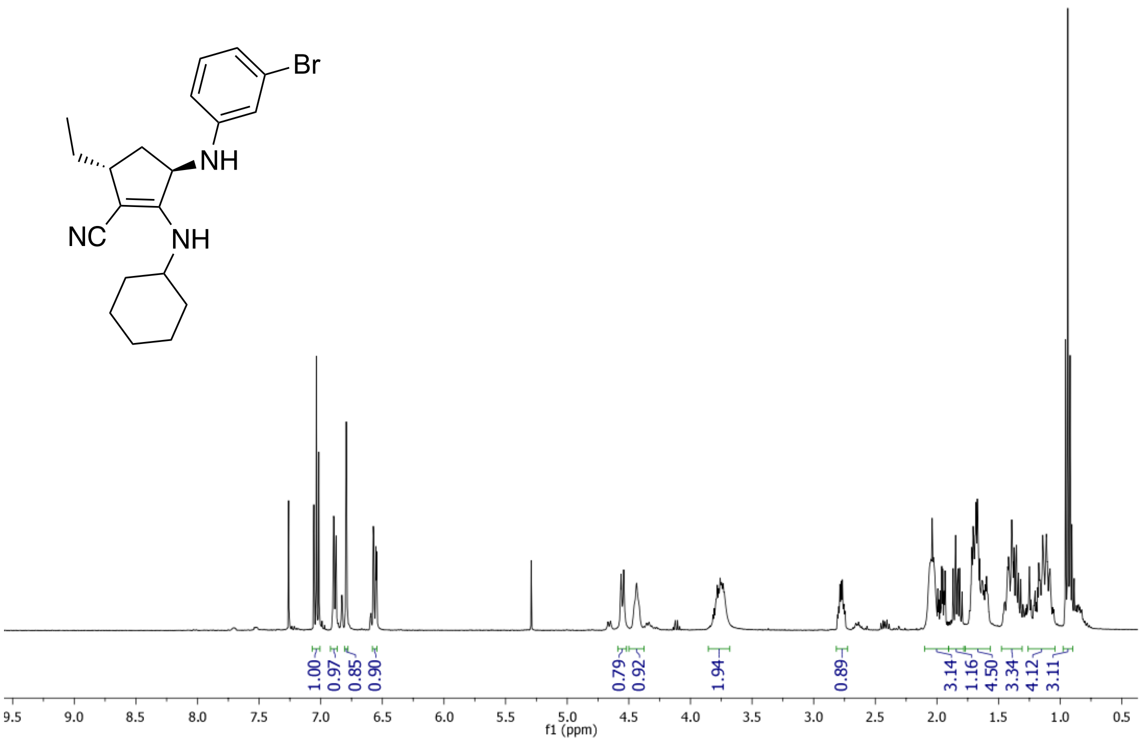

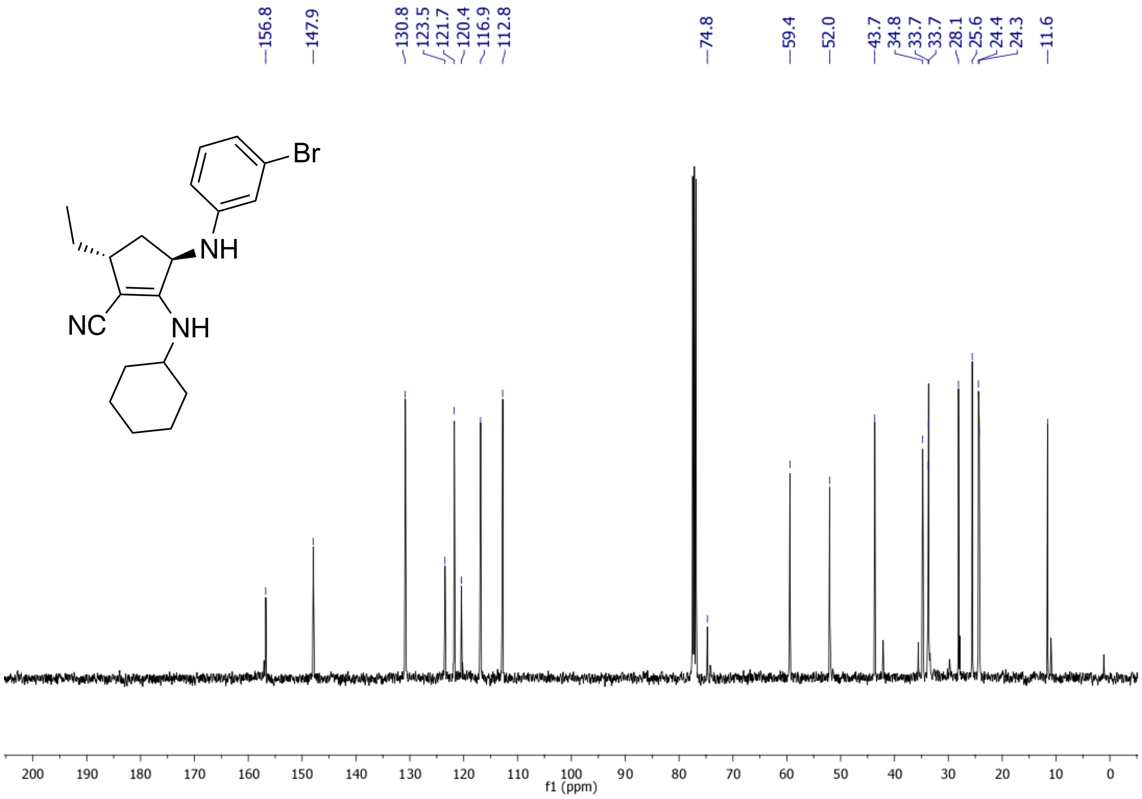

Figure S37. ^{1}H and ^{13}C NMR spectra in $CDCl_3$ of compound **2x.**

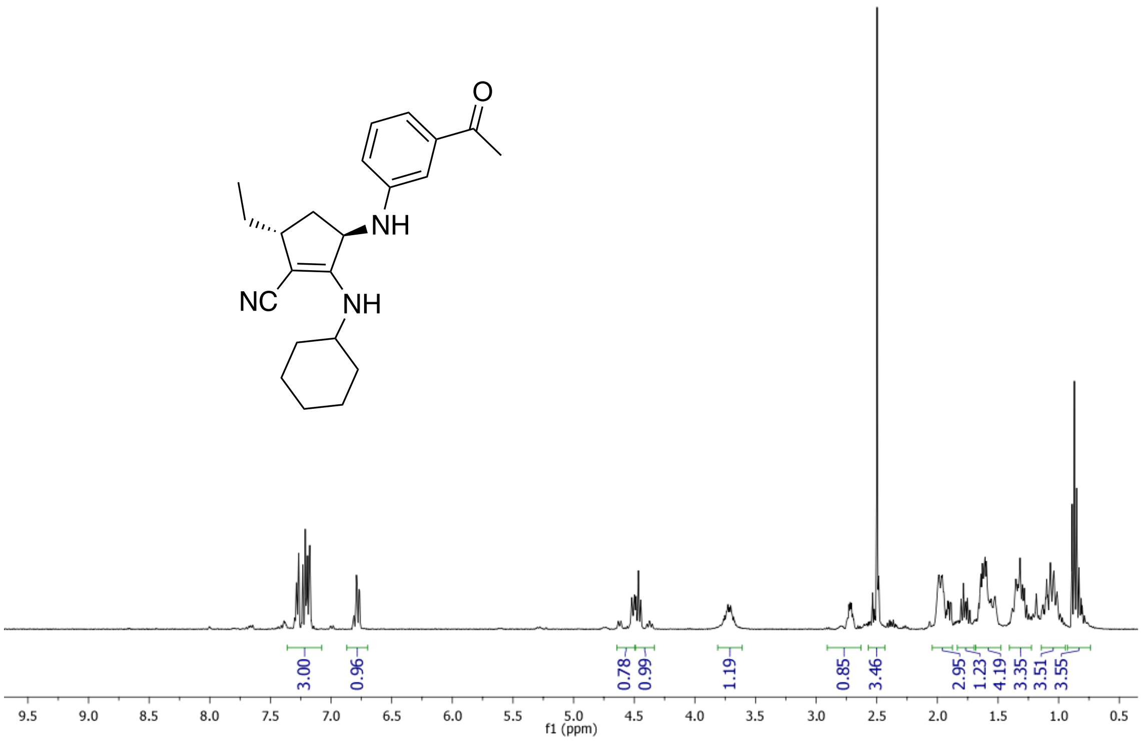

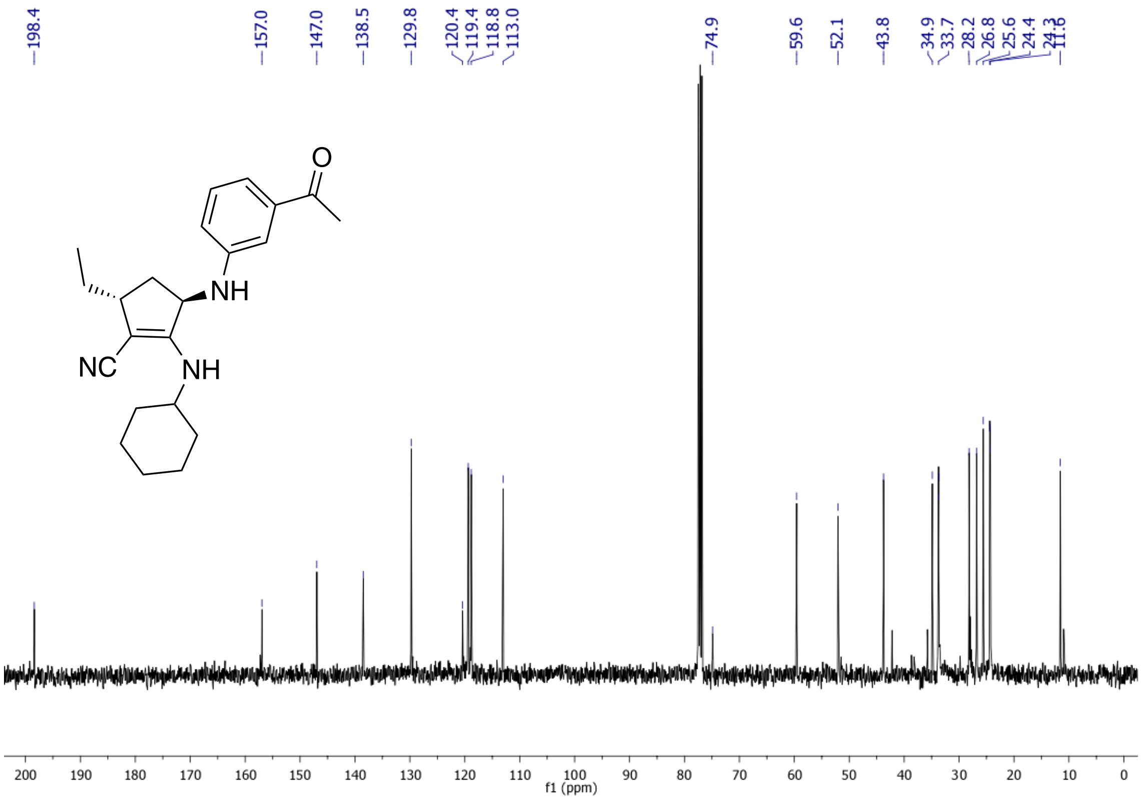

Figure S38. ^{1}H and ^{13}C NMR spectra in $CDCl_3$ of compound **2y.**

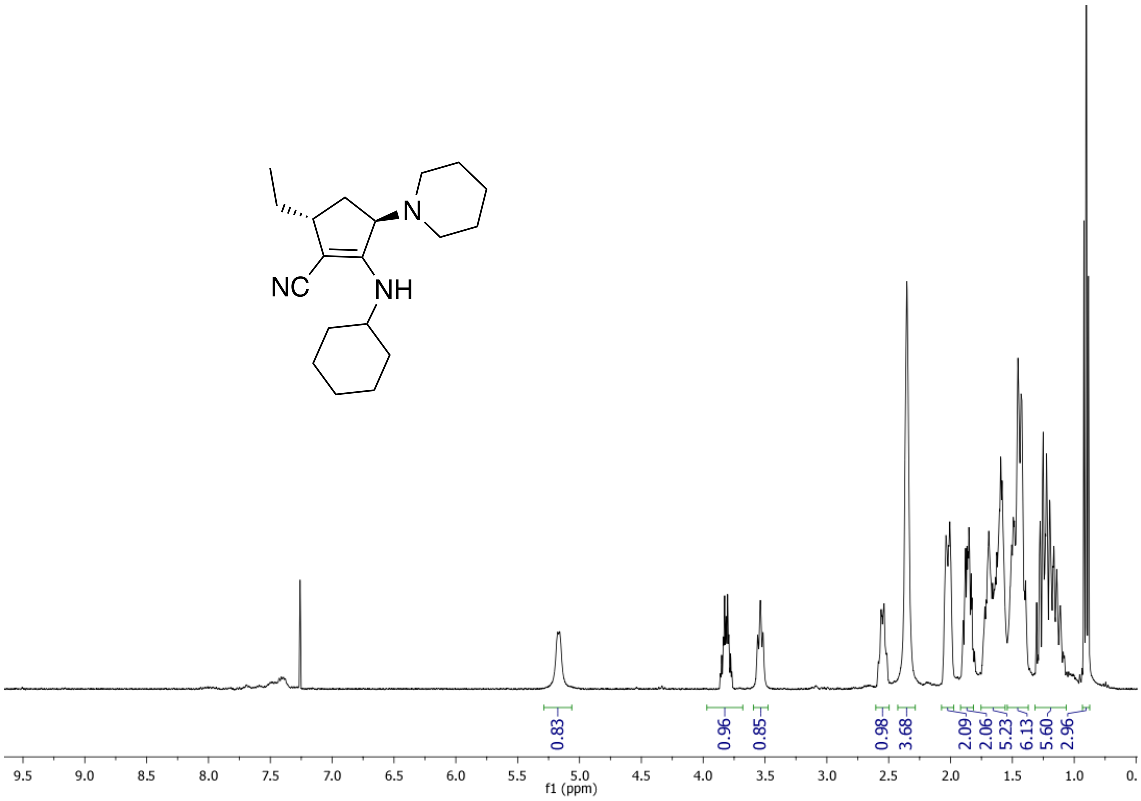

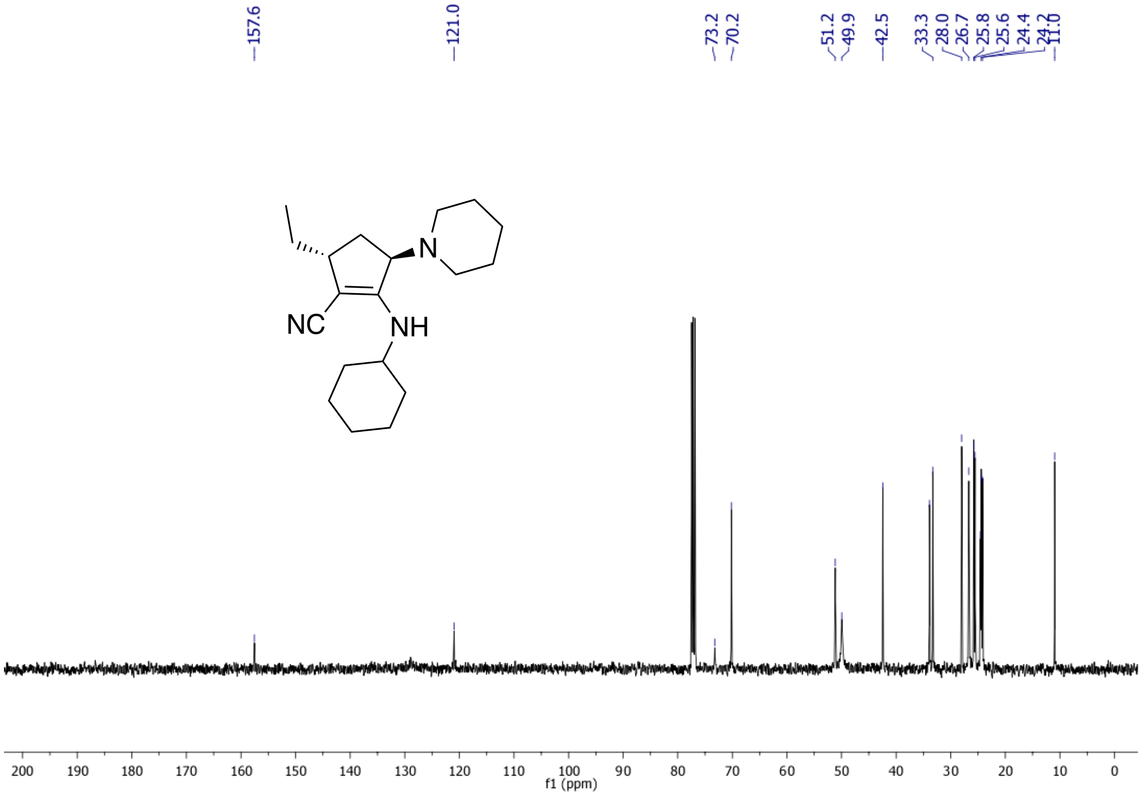

Figure S39. ^{1}H and ^{13}C NMR spectra in $CDCl_3$ of compound **2z.**

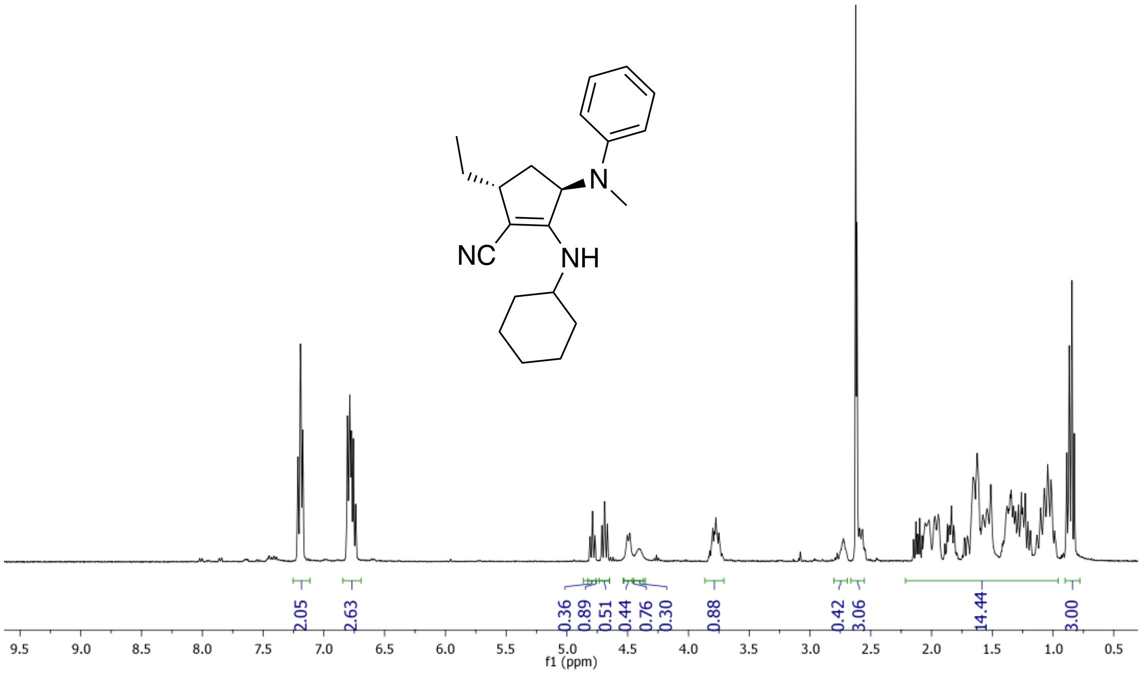

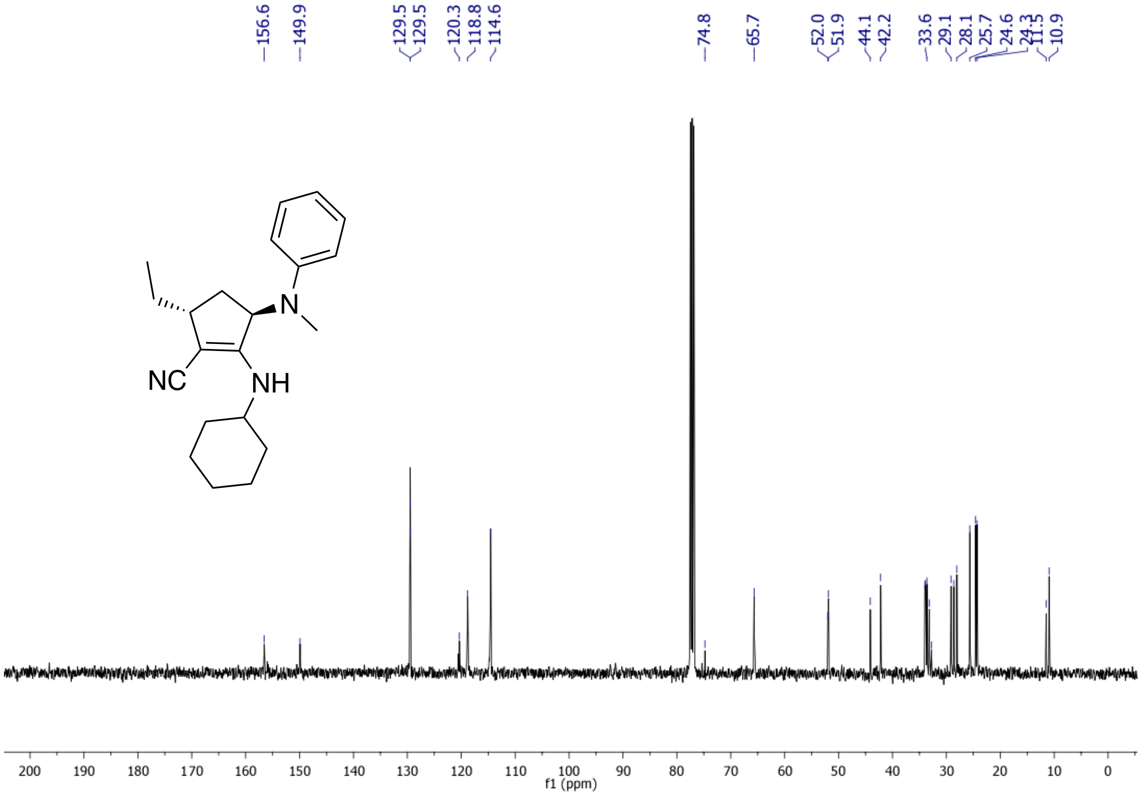

Figure S40. ^{1}H and ^{13}C NMR spectra in $CDCl_3$ of compound **2aa.**

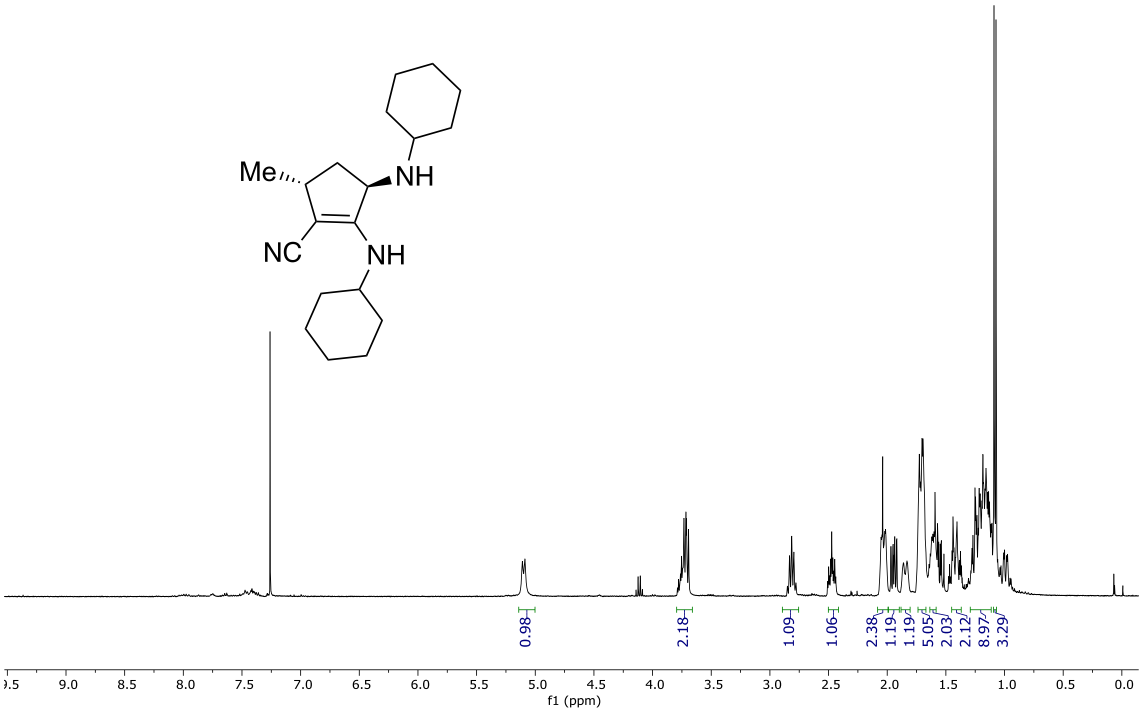

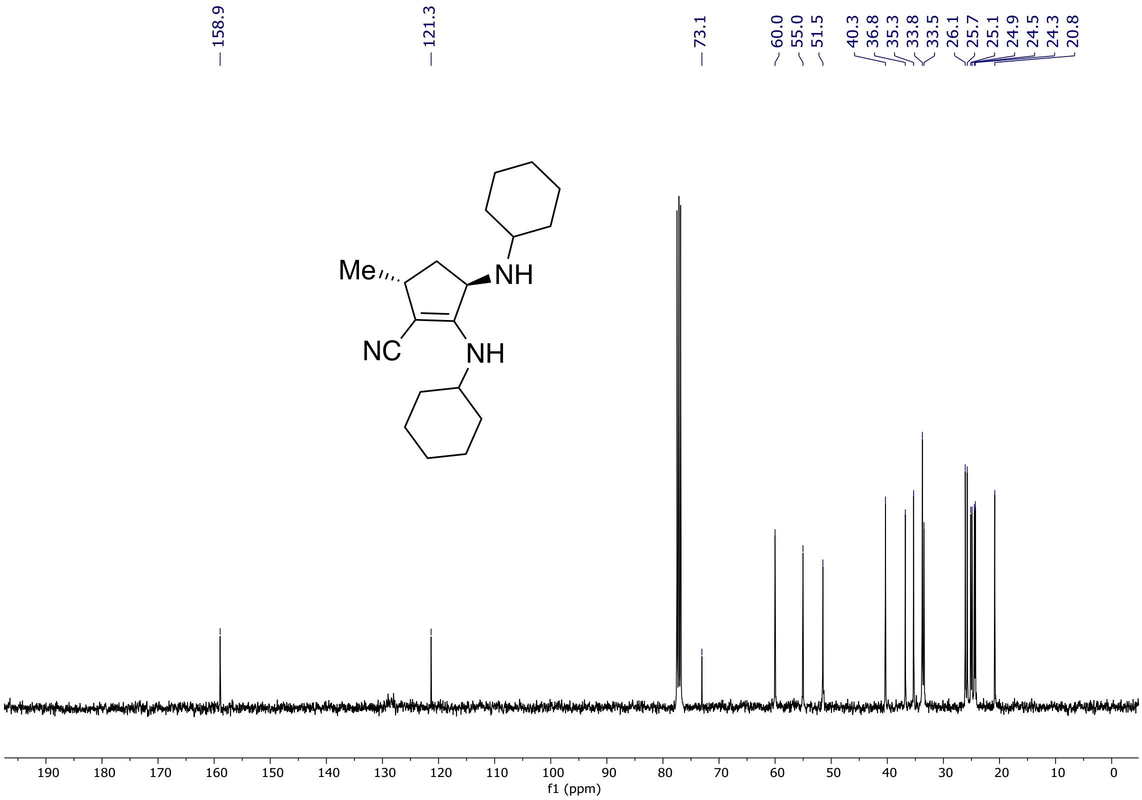

Figure S41. ^{1}H and ^{13}C NMR spectra in $CDCl_3$ of compound **2ab**.

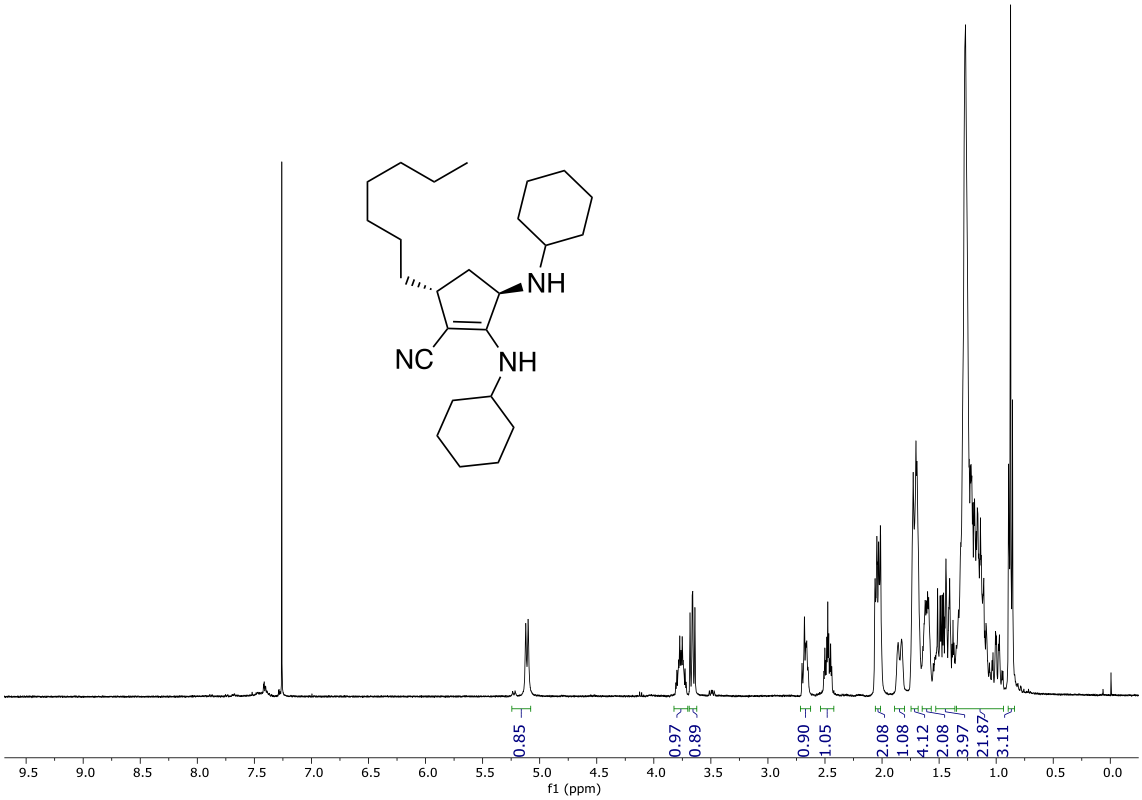

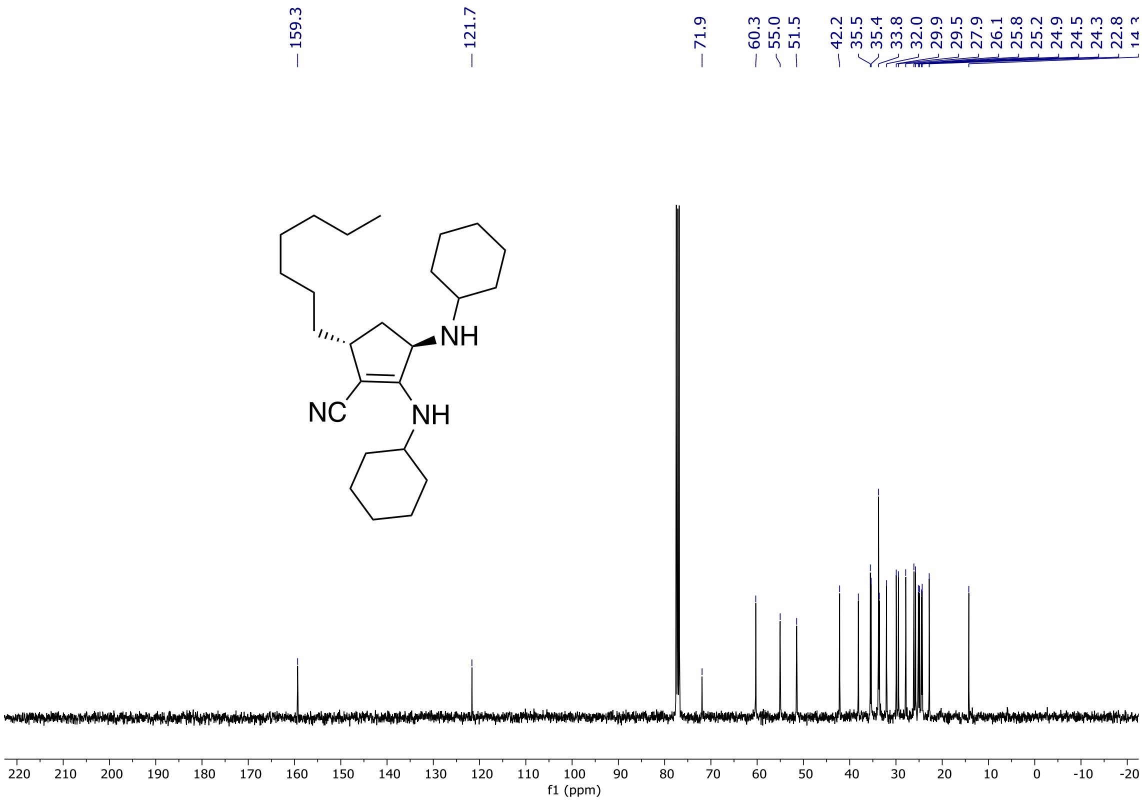

Figure S42. ^{1}H and ^{13}C NMR spectra in $CDCl_3$ of compound **2ac**.

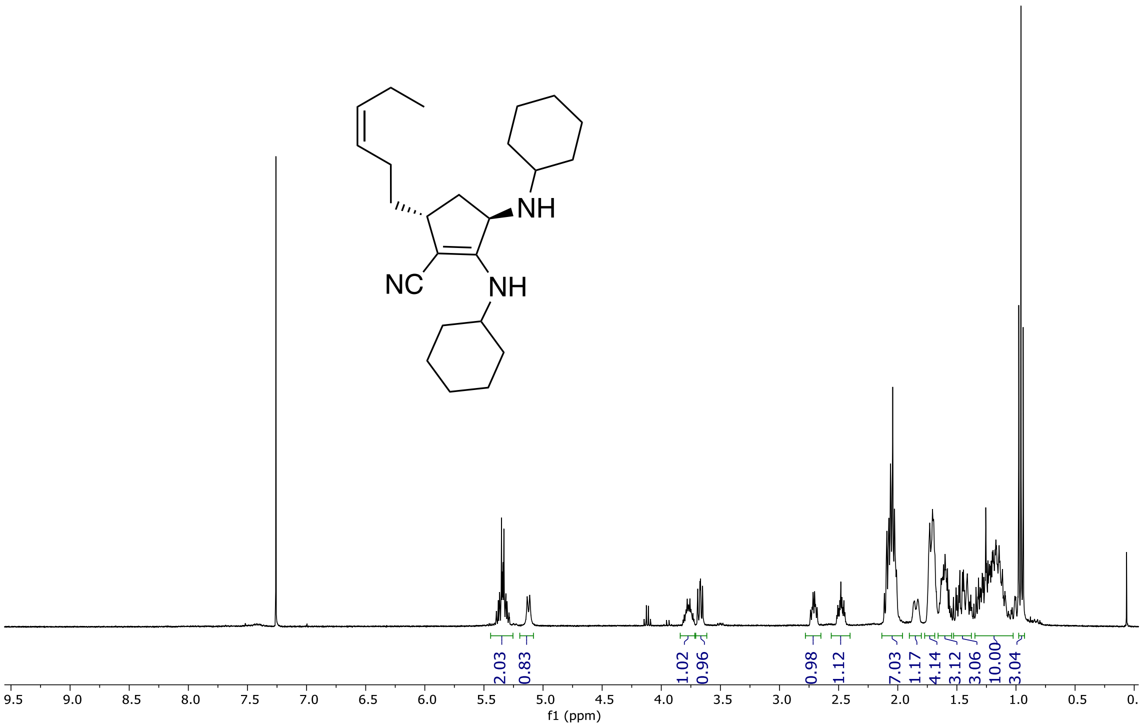

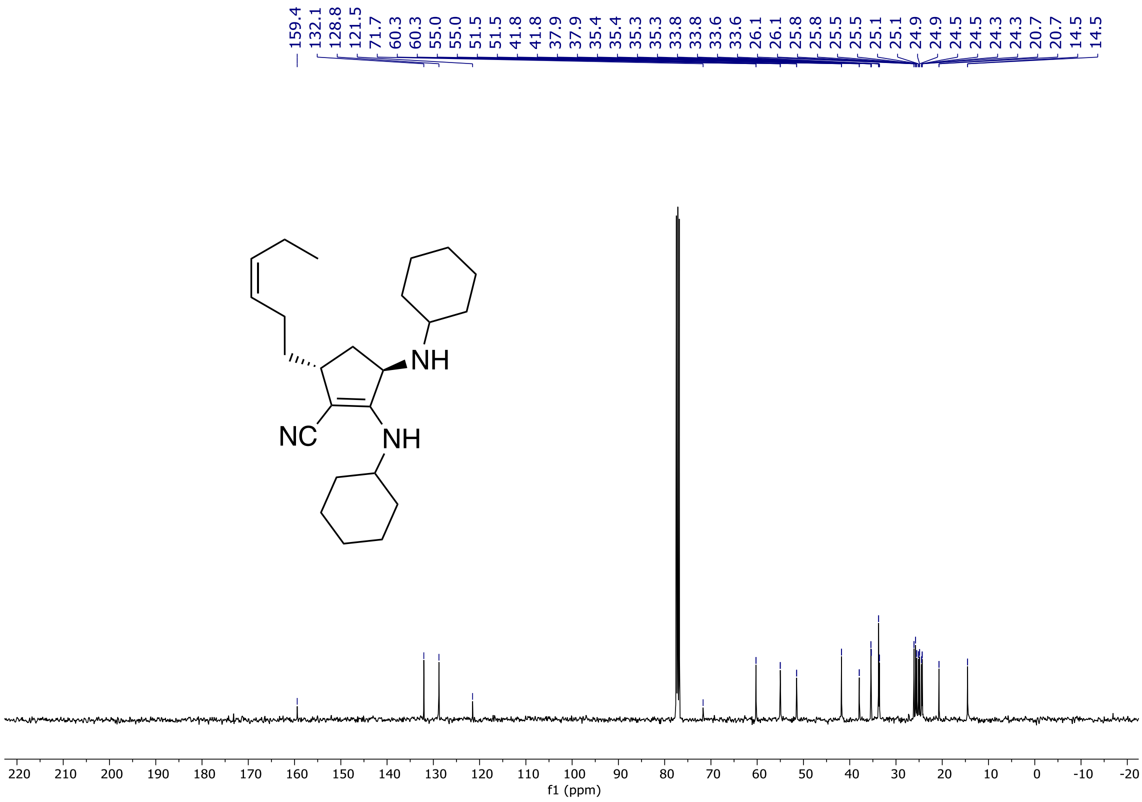

Figure S43. ^{1}H and ^{13}C NMR spectra in $CDCl_3$ of compound **2ad**.

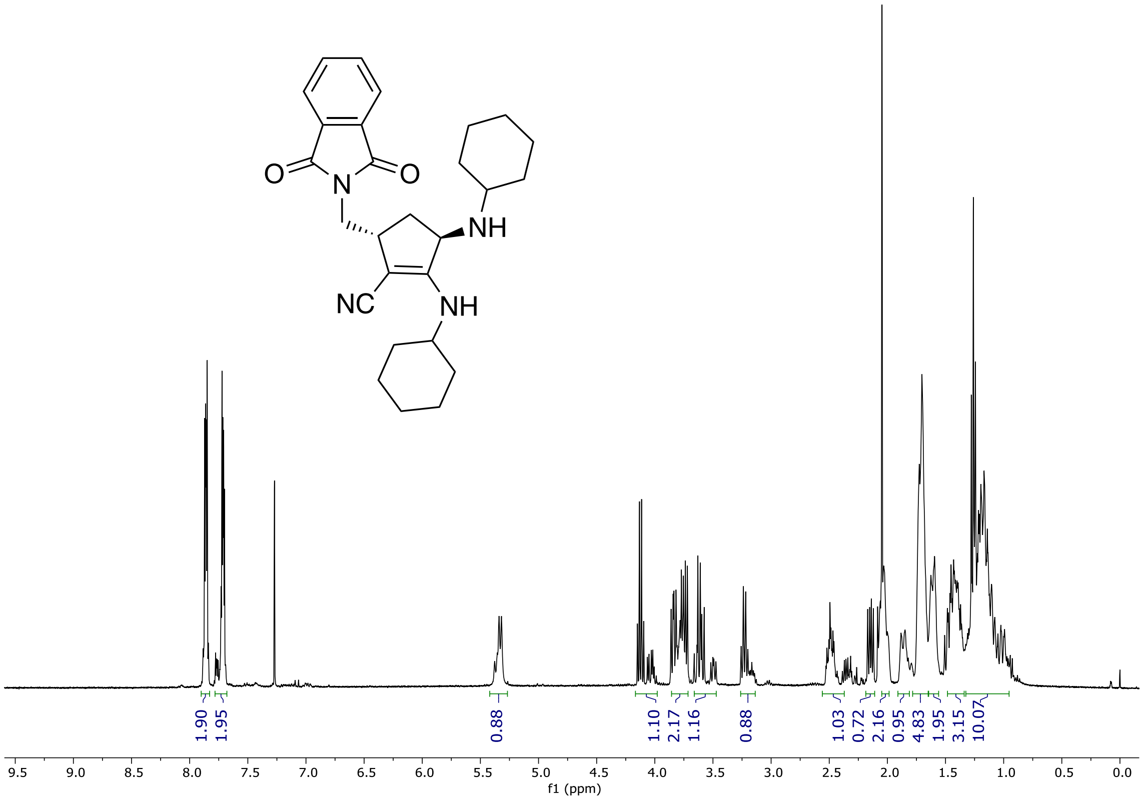

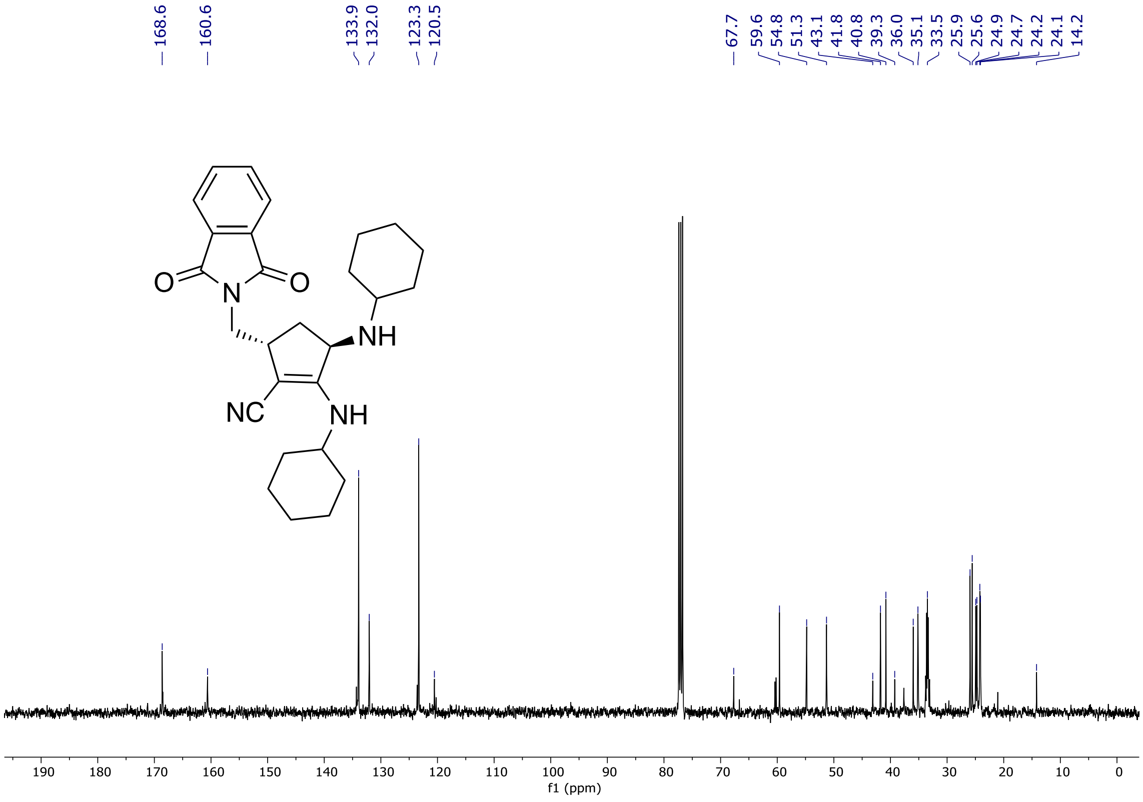

Figure S44. ^{1}H and ^{13}C NMR spectra in $CDCl_3$ of compound **2ae**.

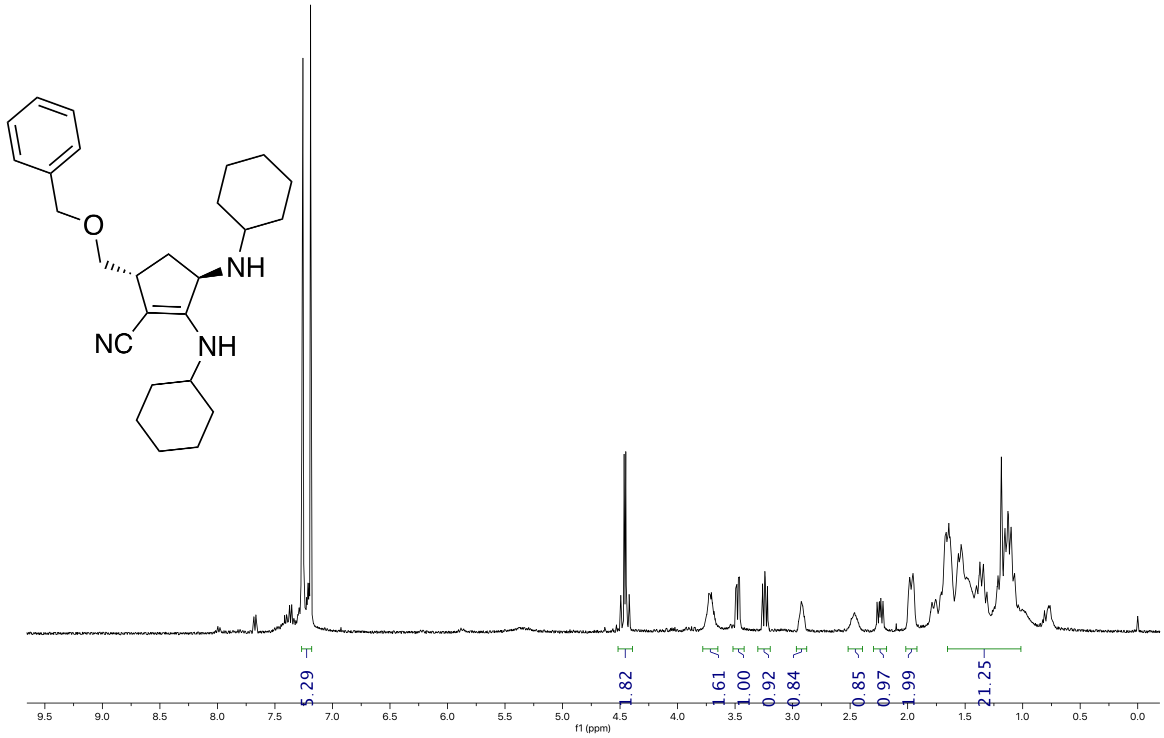

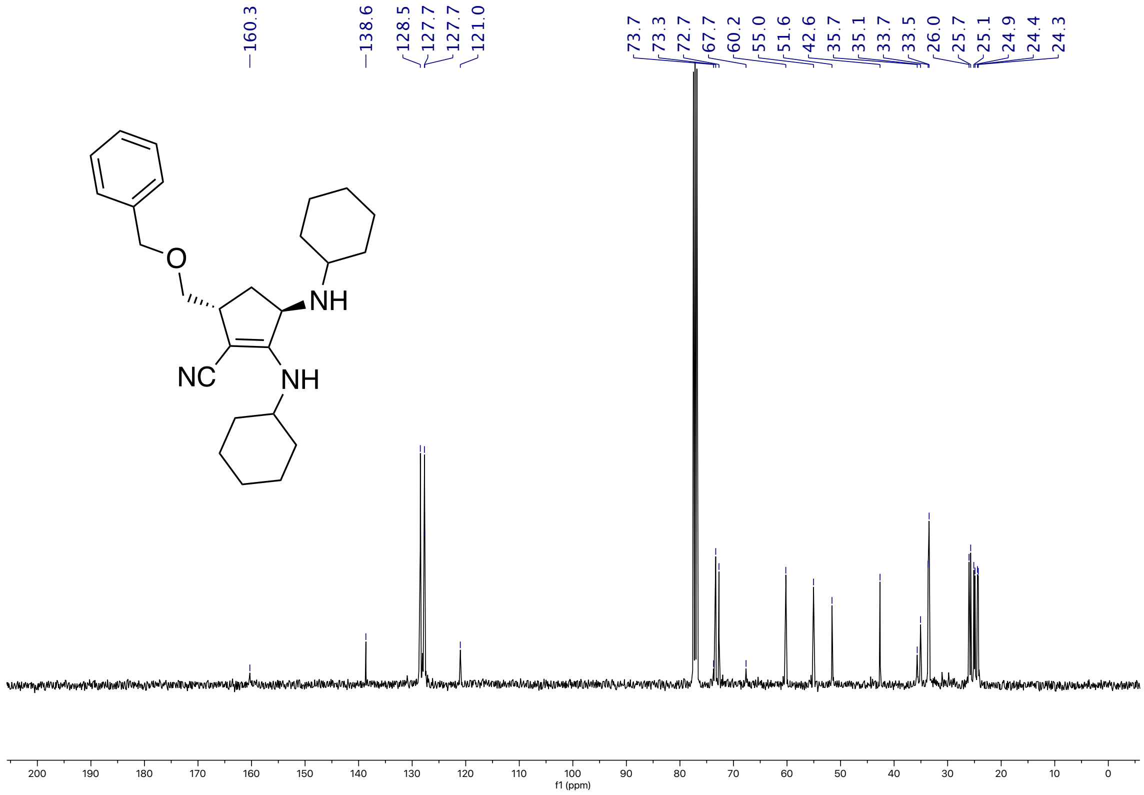

Figure S45. ${}^{1}H$ and ${}^{13}C$ NMR spectra in $CDCl_3$ of compound **2af**.

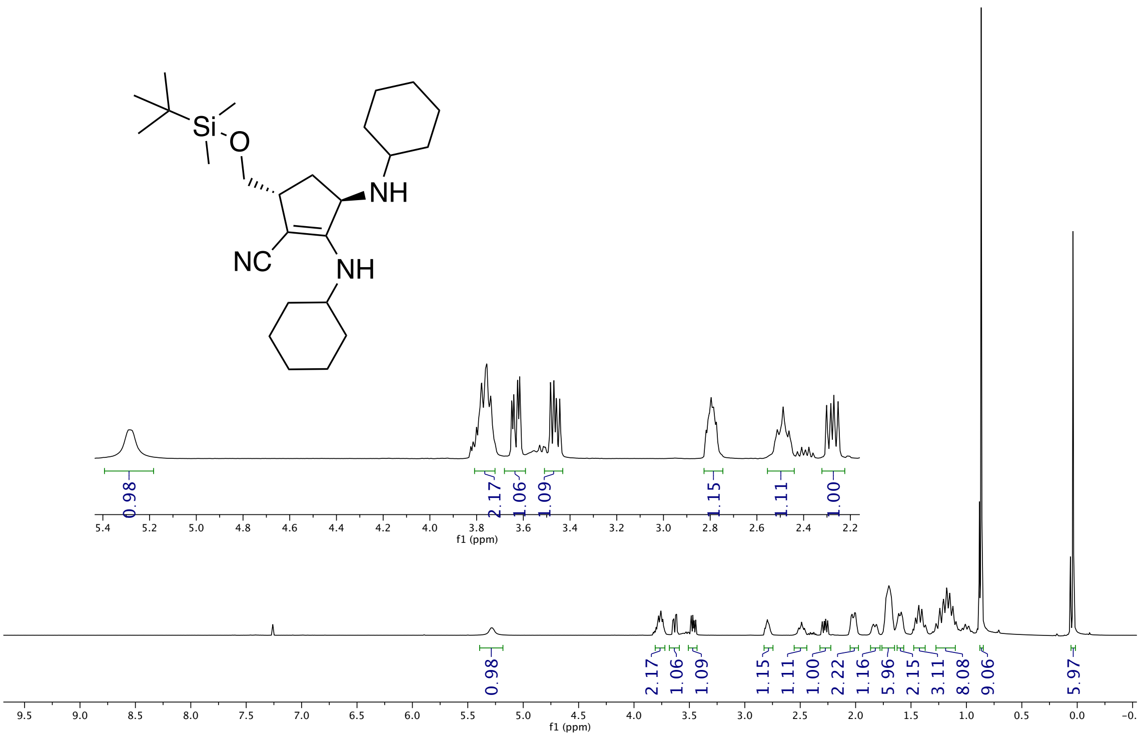

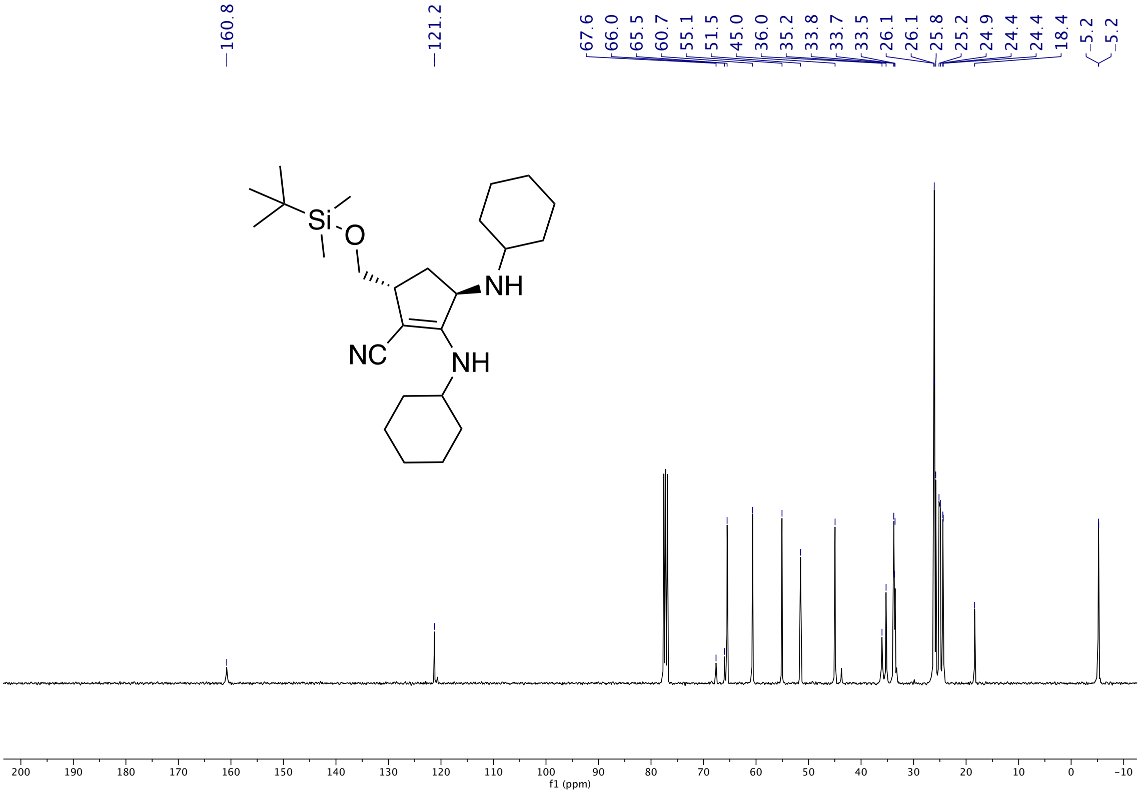

Figure S46. ^{1}H and ^{13}C NMR spectra in $CDCl_3$ of compound **2ag**.

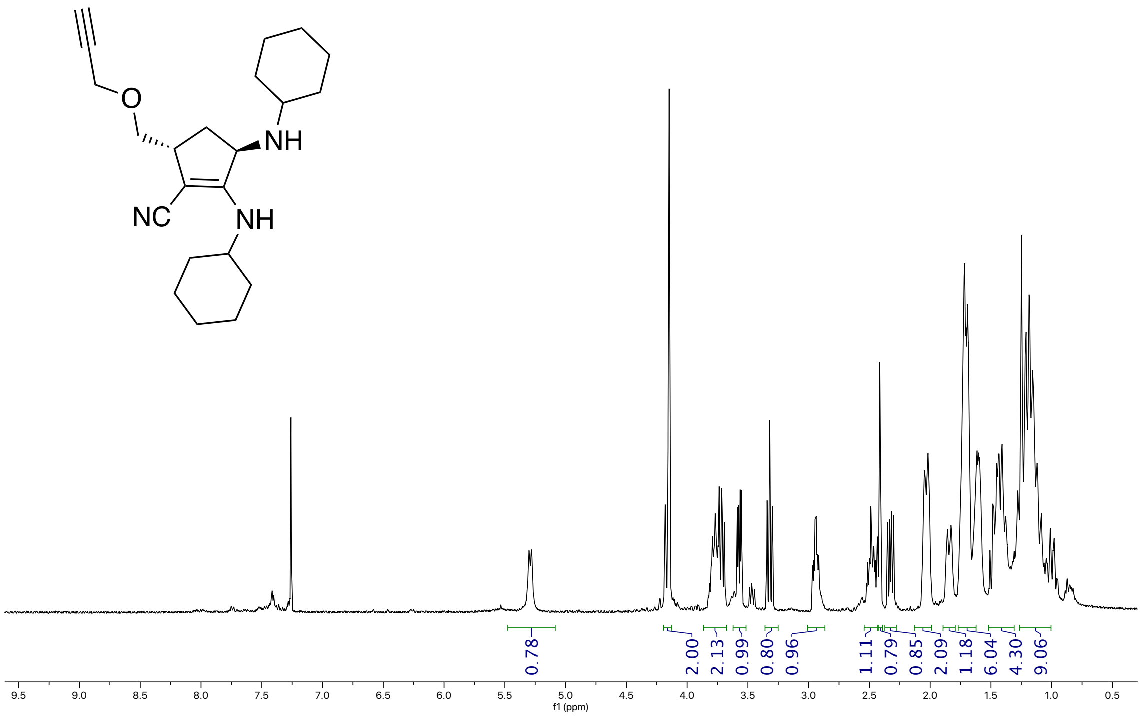

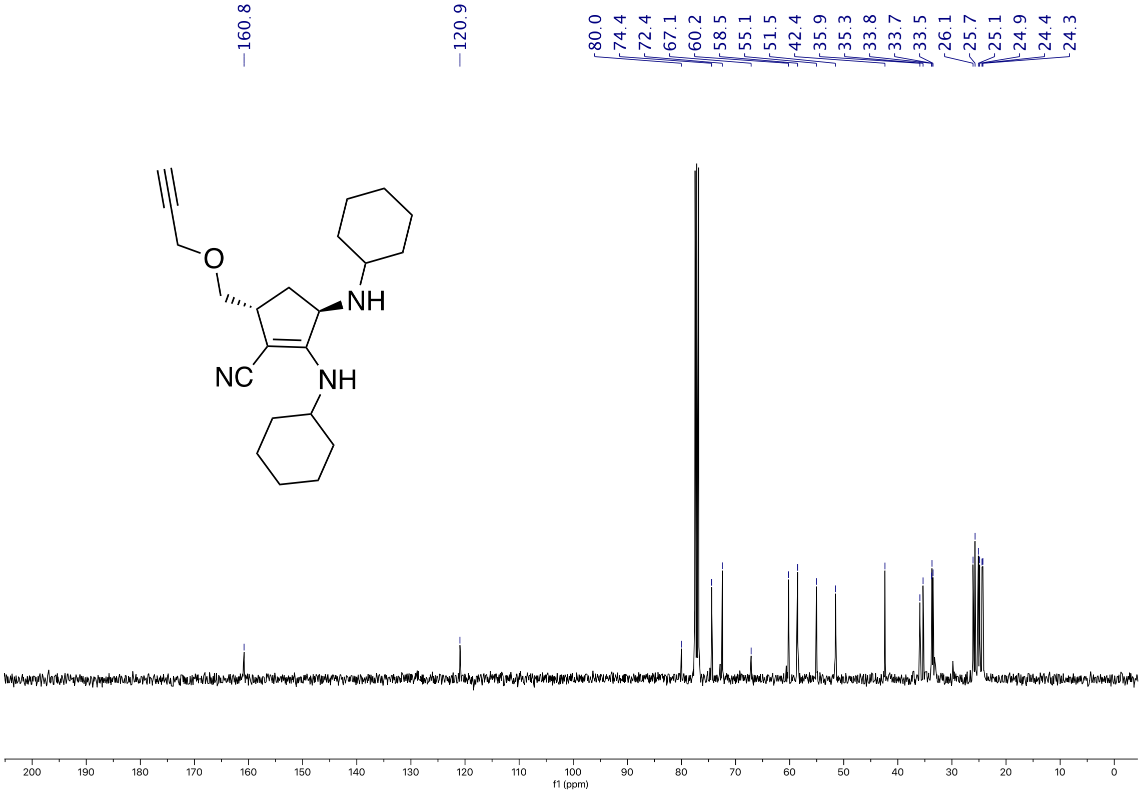

Figure S47. ^{1}H and ^{13}C NMR spectra in $CDCl_3$ of compound **2ah**.

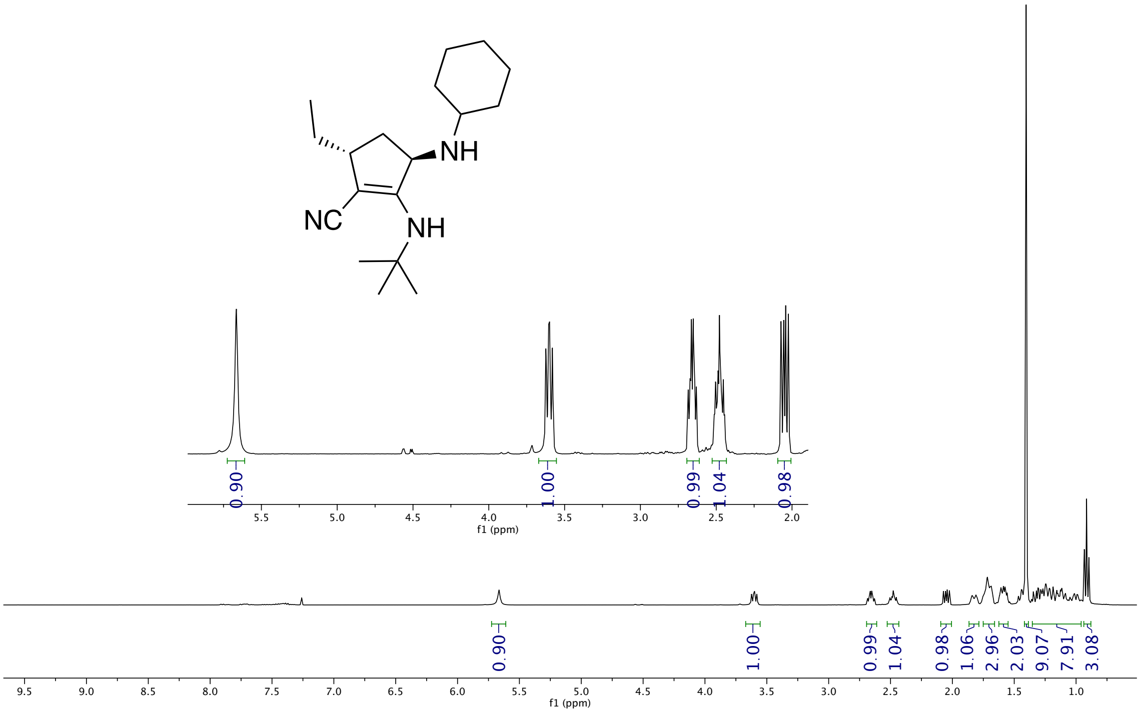

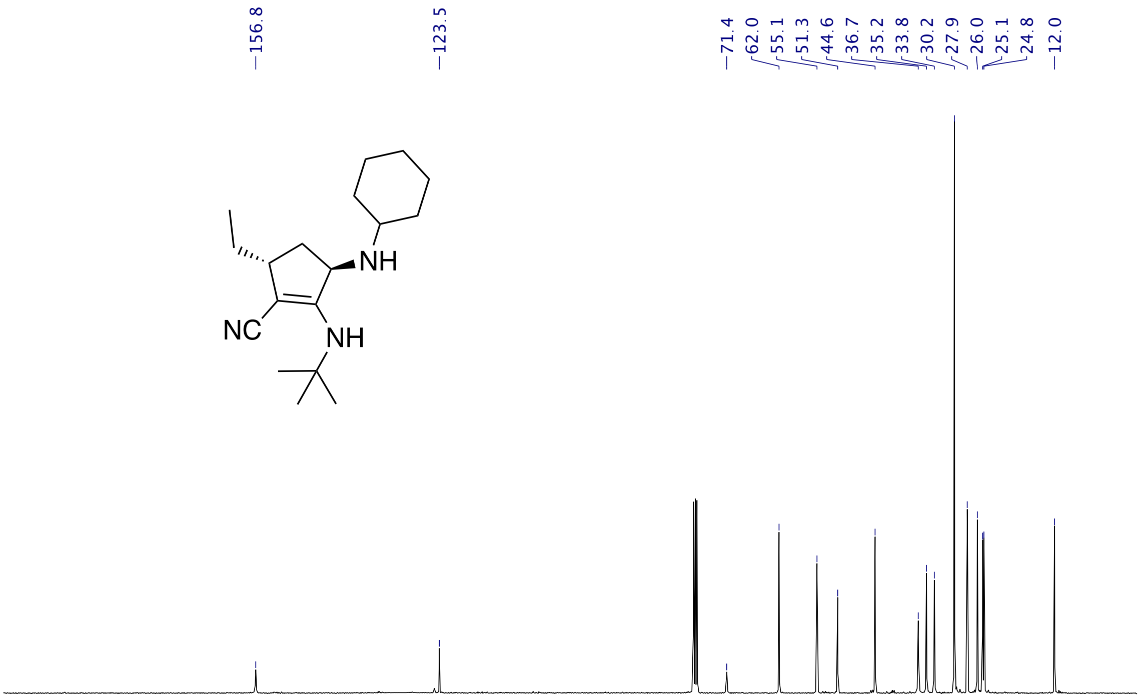

Figure S48. ^{1}H and ^{13}C NMR spectra in $CDCl_3$ of compound **2ai**.

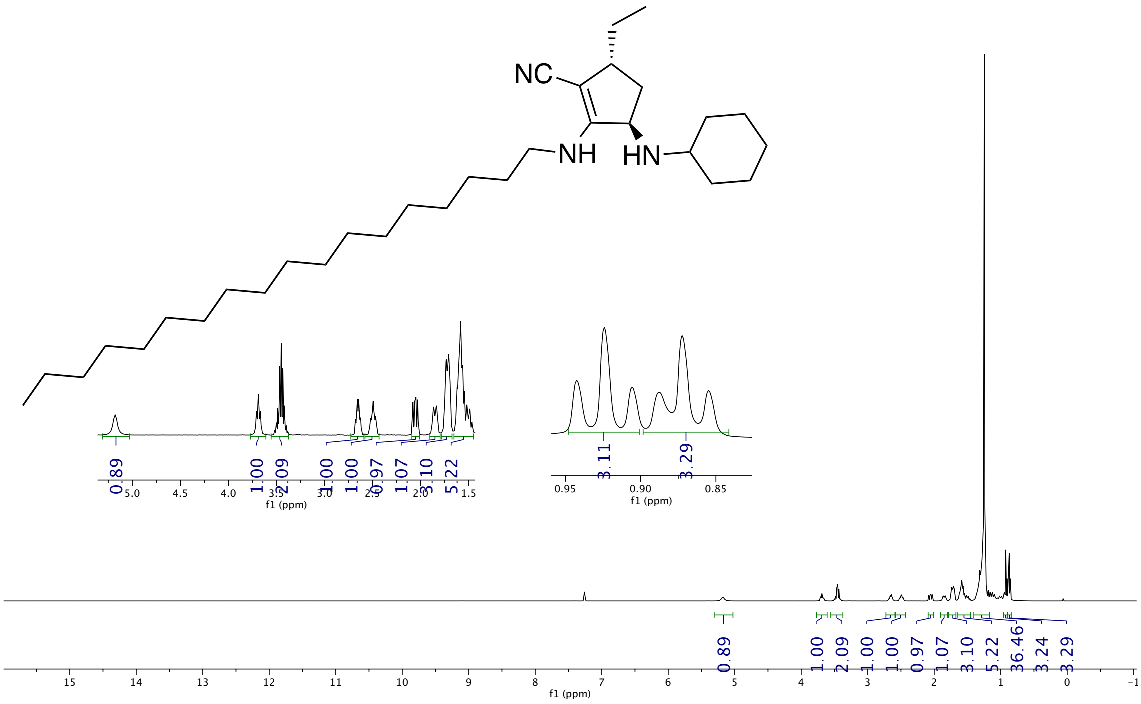

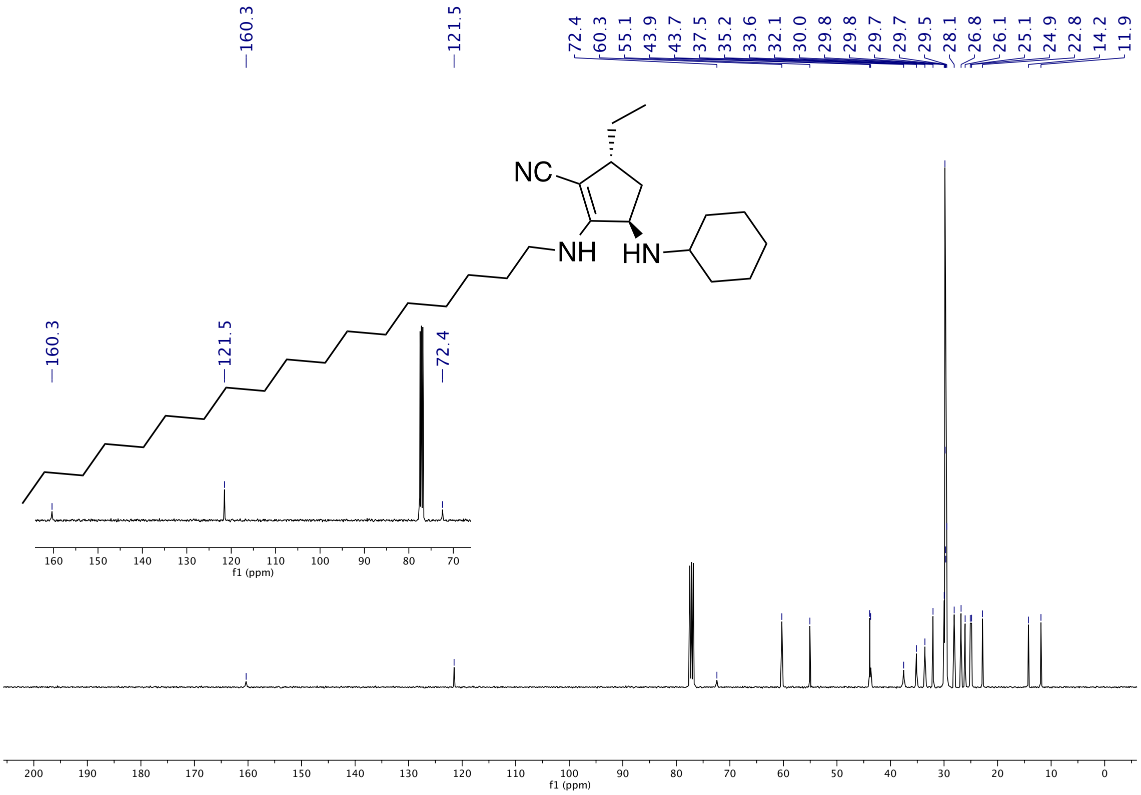

Figure S49. ^{1}H and ^{13}C NMR spectra in $CDCl_3$ of compound **2aj.**

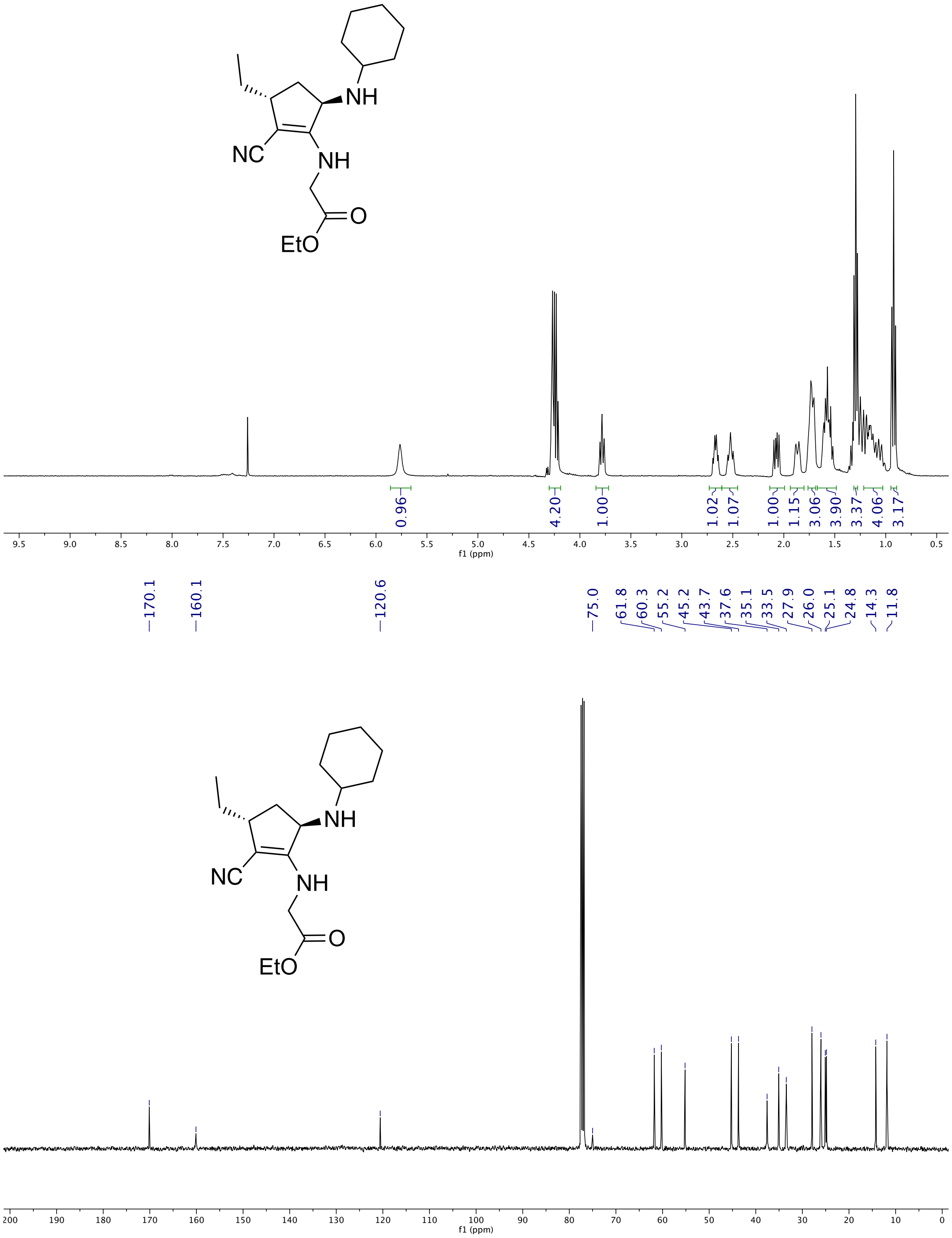

Figure S50. ^{1}H and ^{13}C NMR spectra in $CDCl_3$ of compound **2ak**.

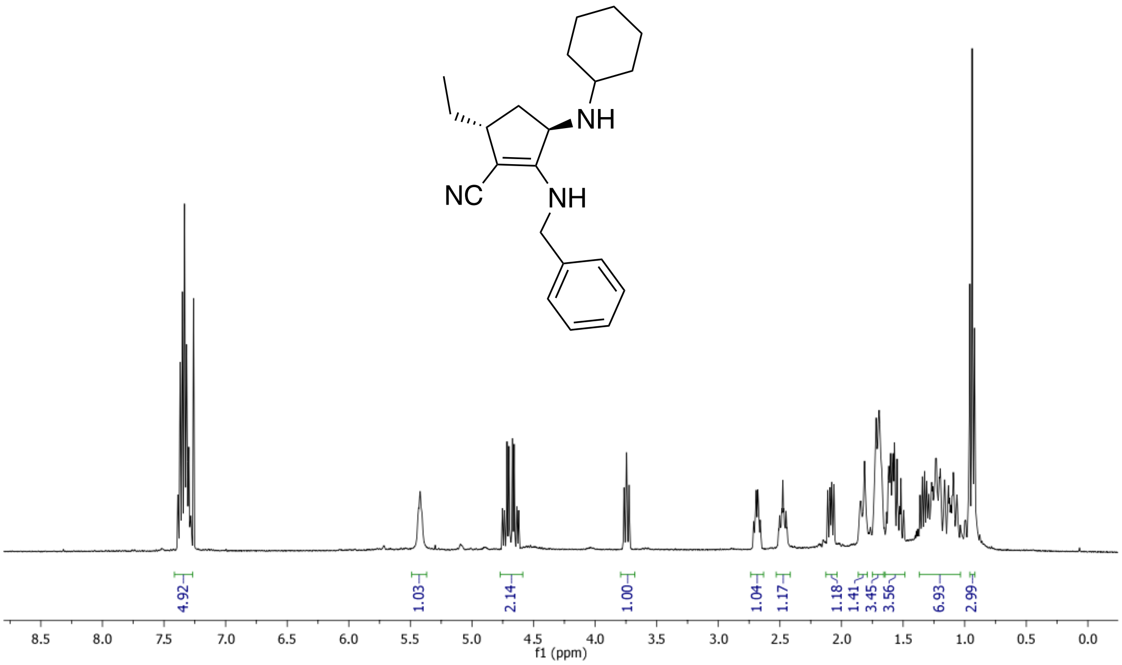

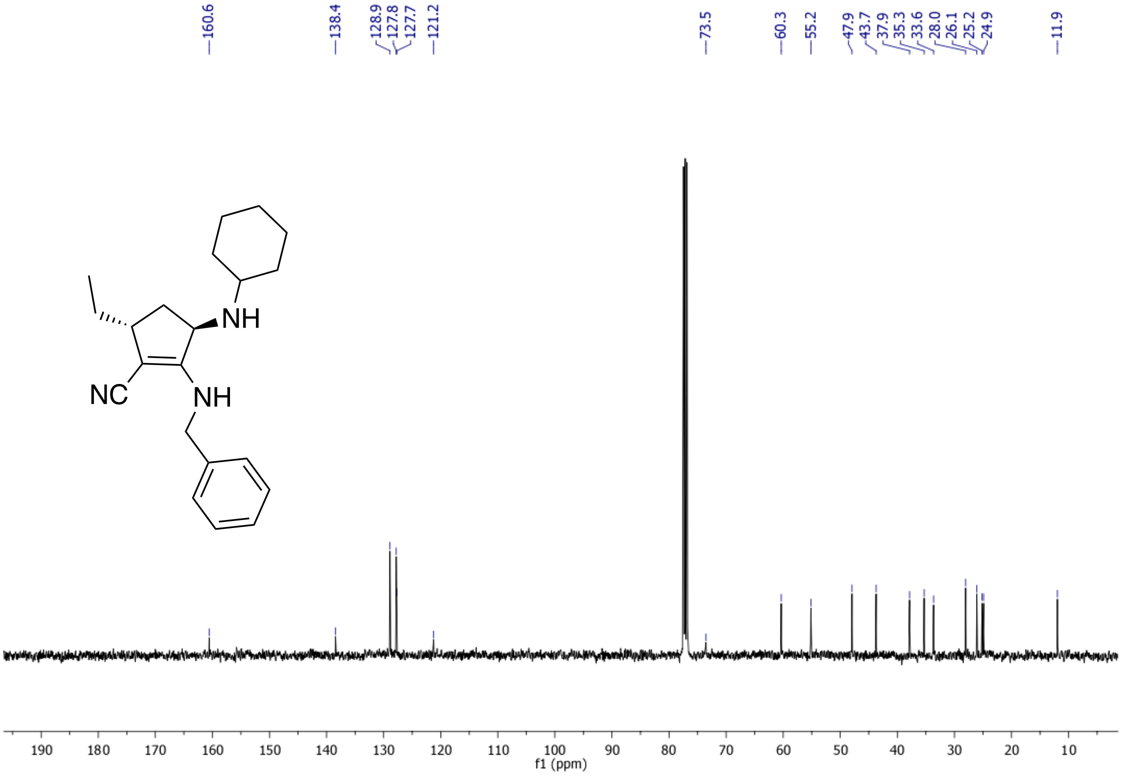

Figure S51. ^{1}H and ^{13}C NMR spectra in $CDCl_3$ of compound **2al.**

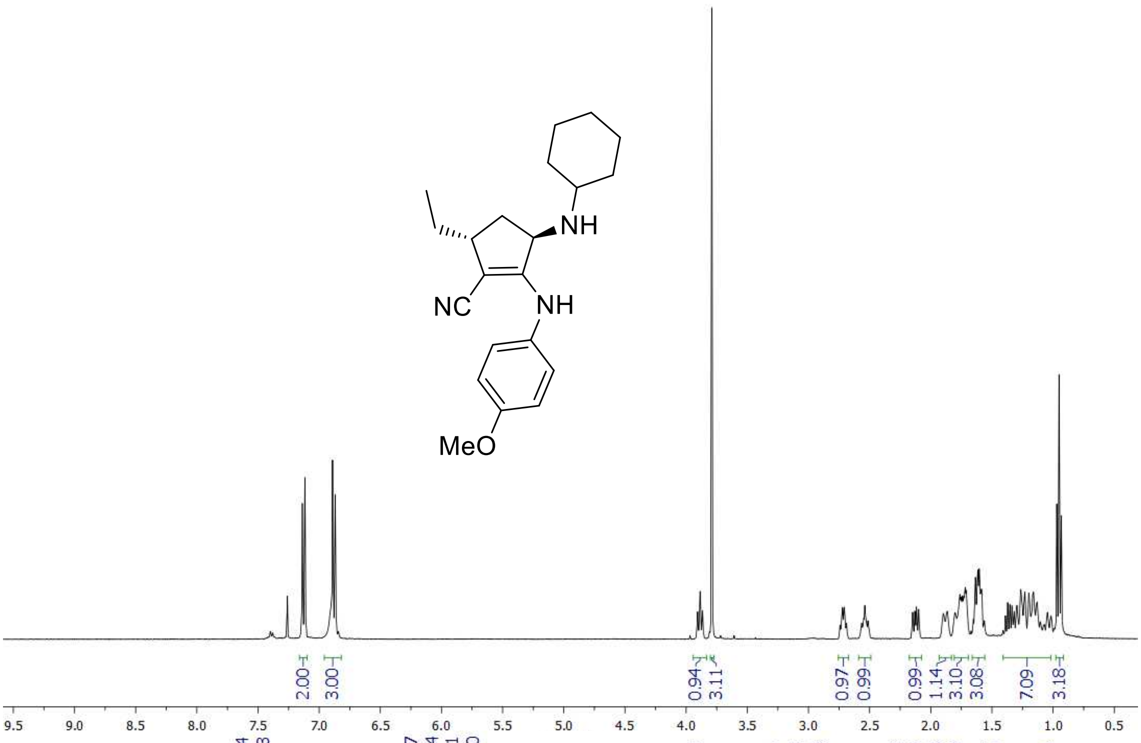

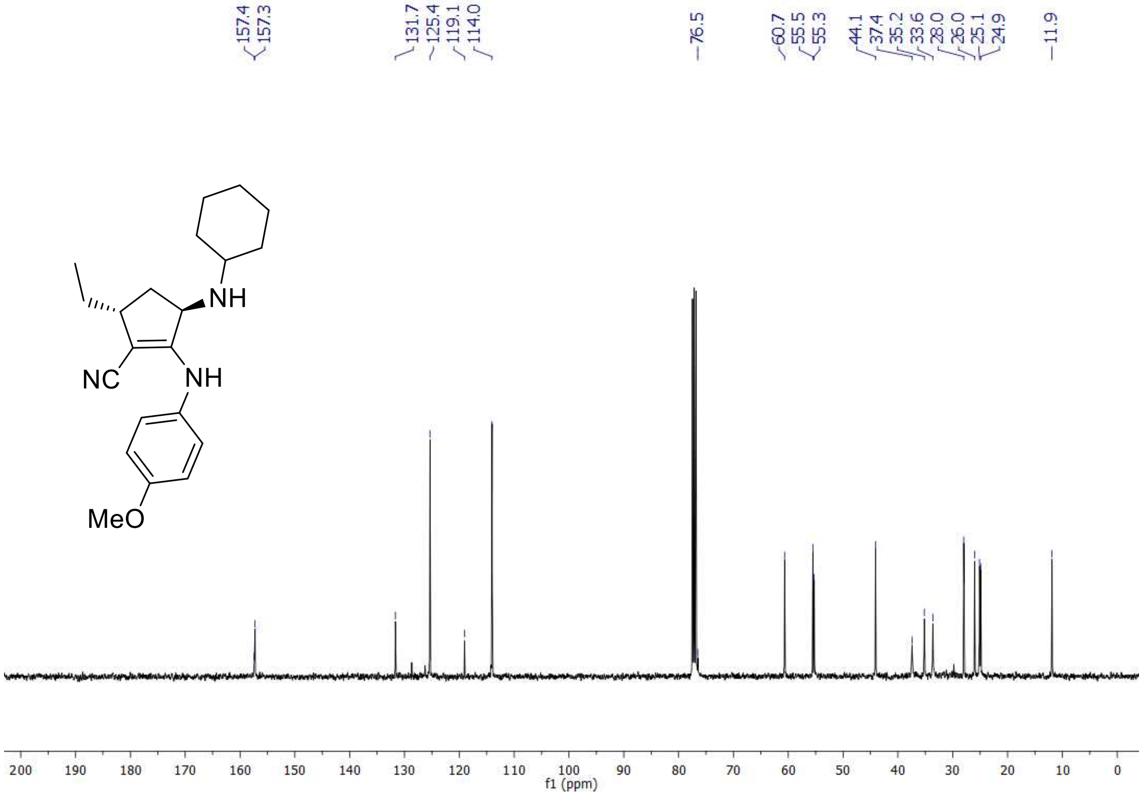

Figure S52. ^{1}H and ^{13}C NMR spectra in $CDCl_3$ of compound **2am.**

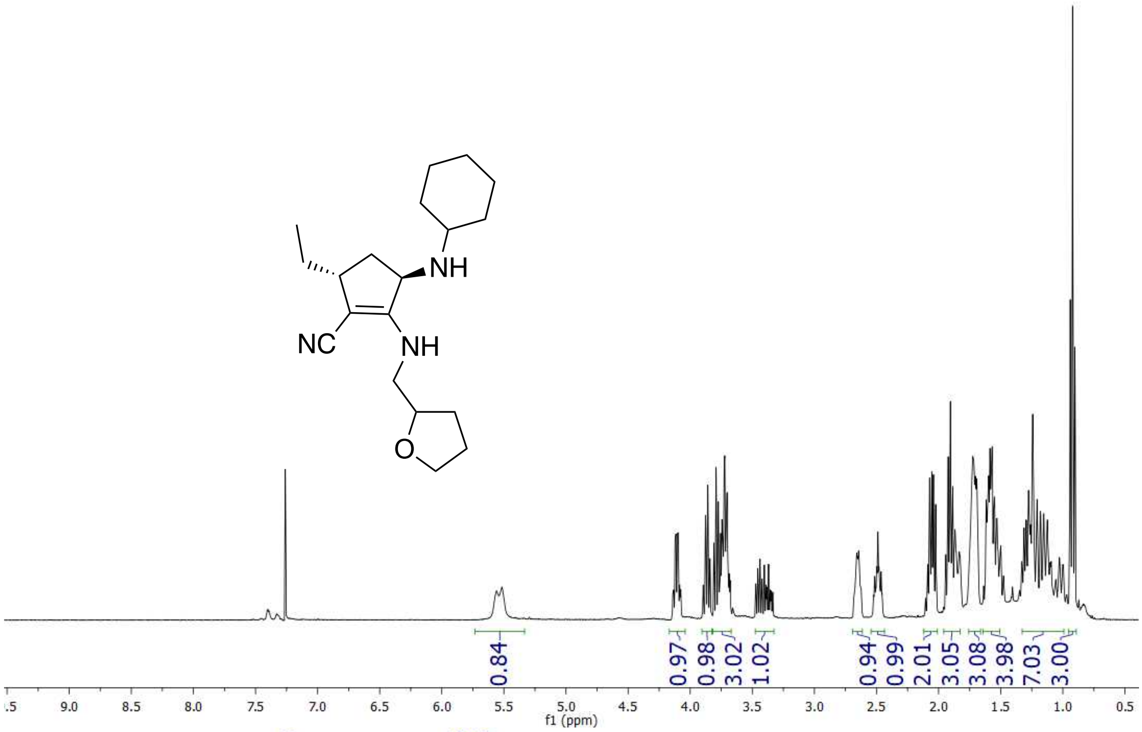

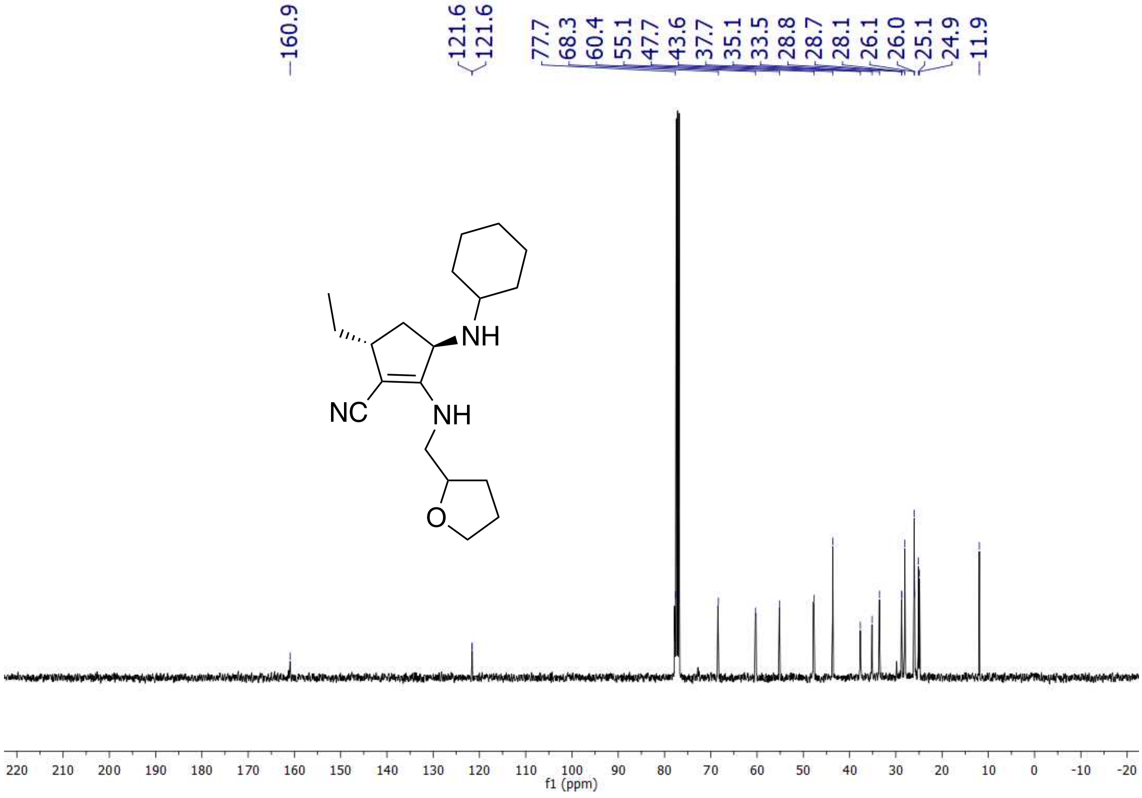

Figure S53. ^{1}H and ^{13}C NMR spectra in $CDCl_3$ of compound **2an.**

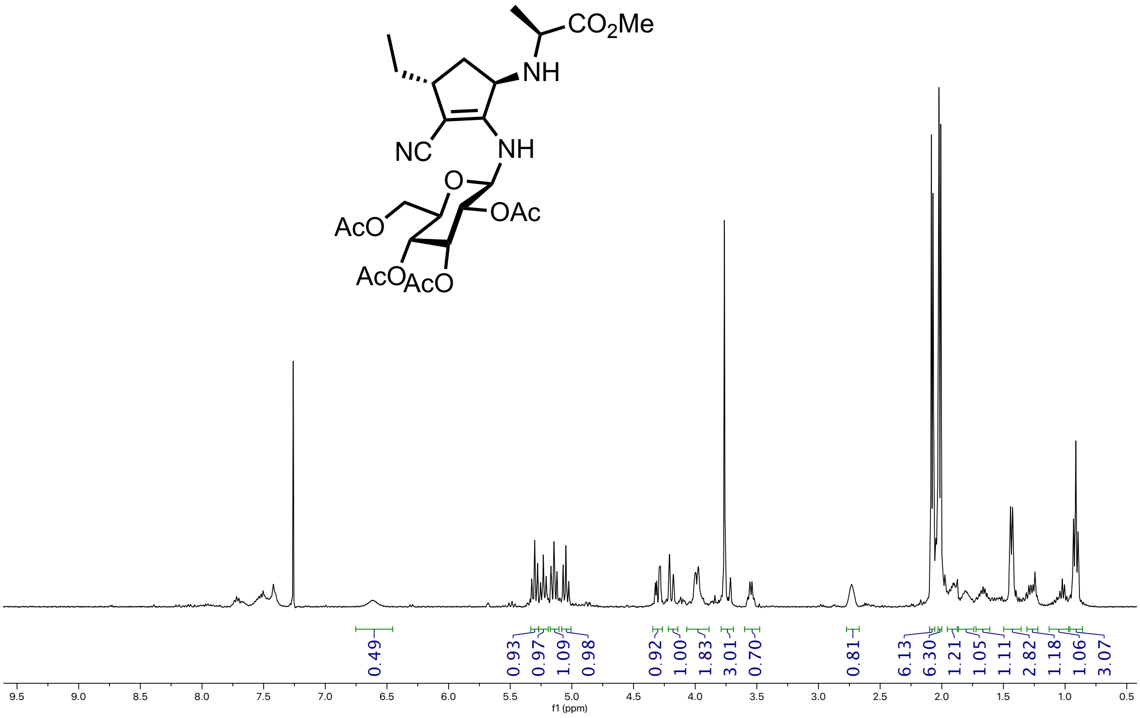

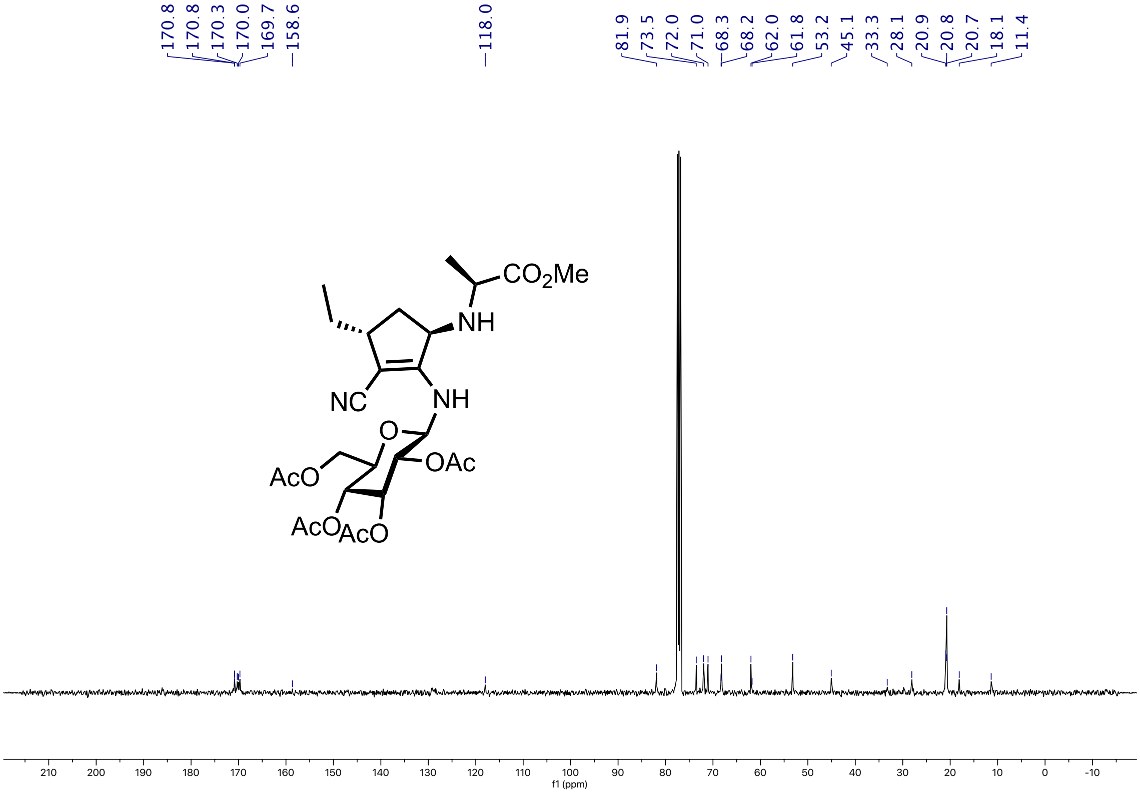

Figure S54. ^{1}H and ^{13}C NMR spectra in $CDCl_3$ of compound **2ao**.

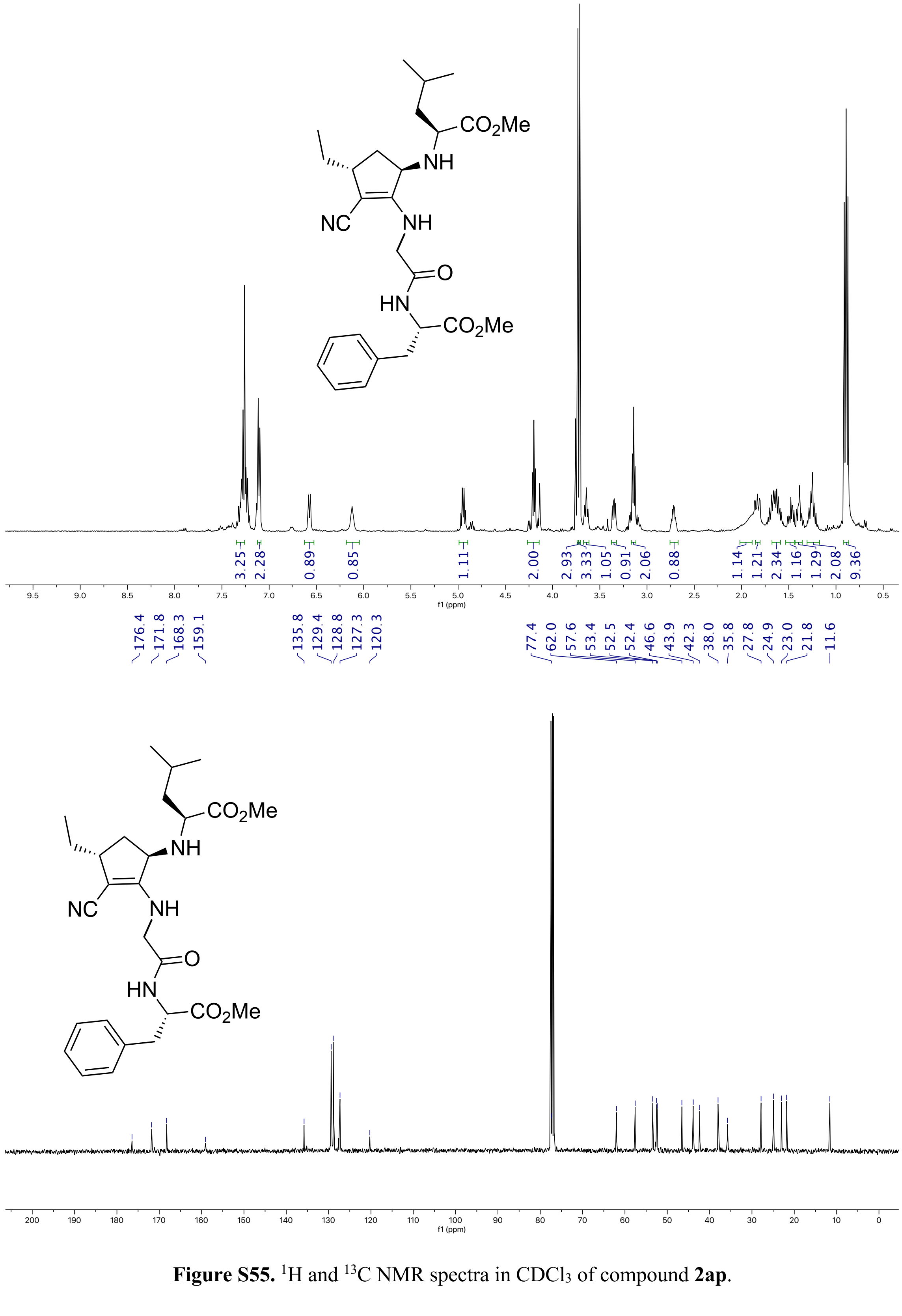

Figure S55. ^{1}H and ^{13}C NMR spectra in $CDCl_3$ of compound **2ap**.

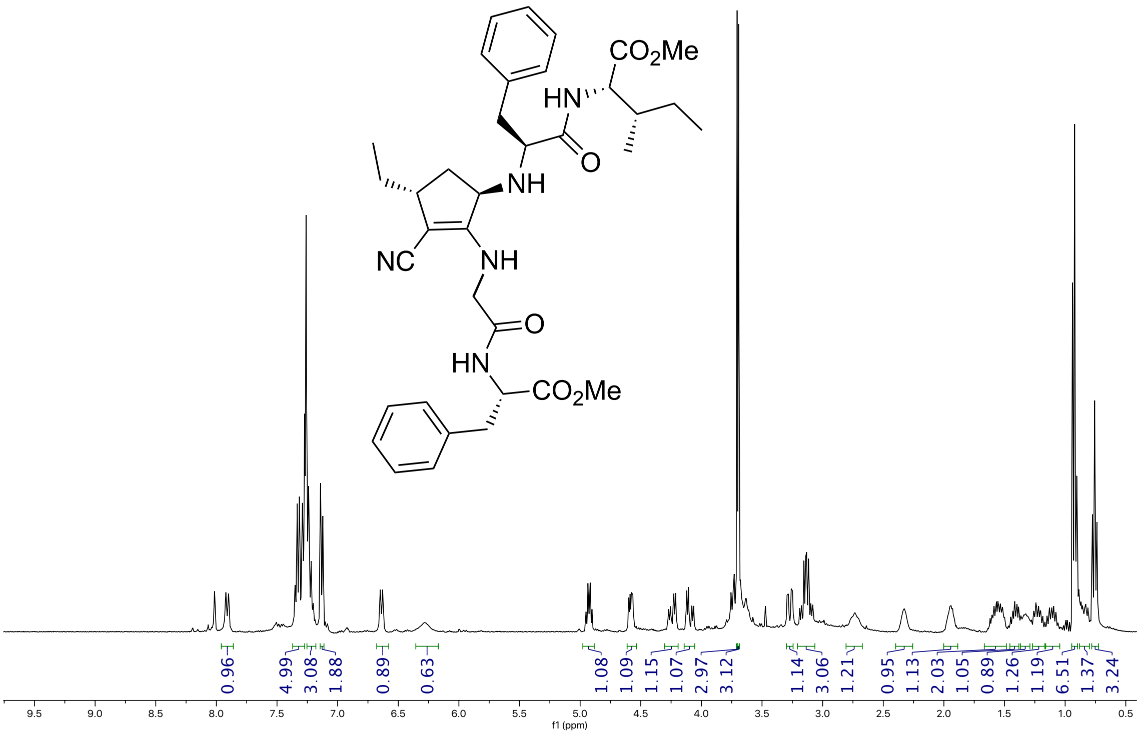

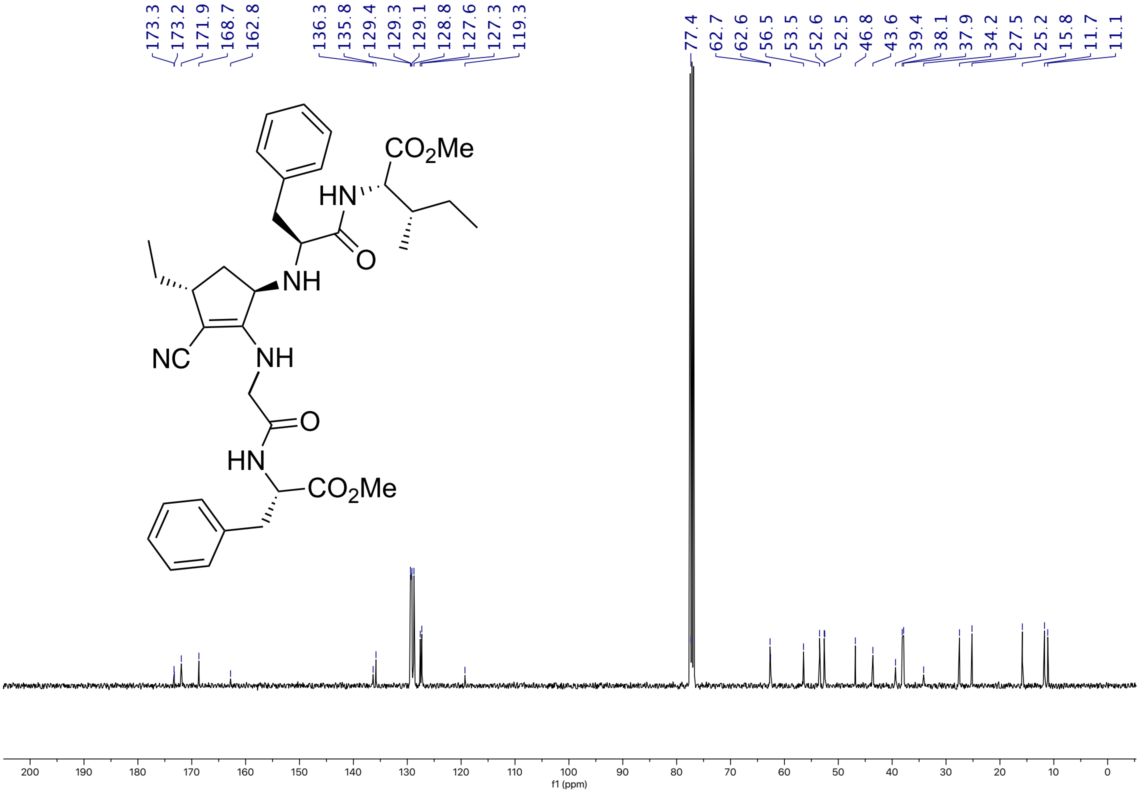

Figure S56. ^{1}H and ^{13}C NMR spectra in $CDCl_3$ of compound **2aq**.

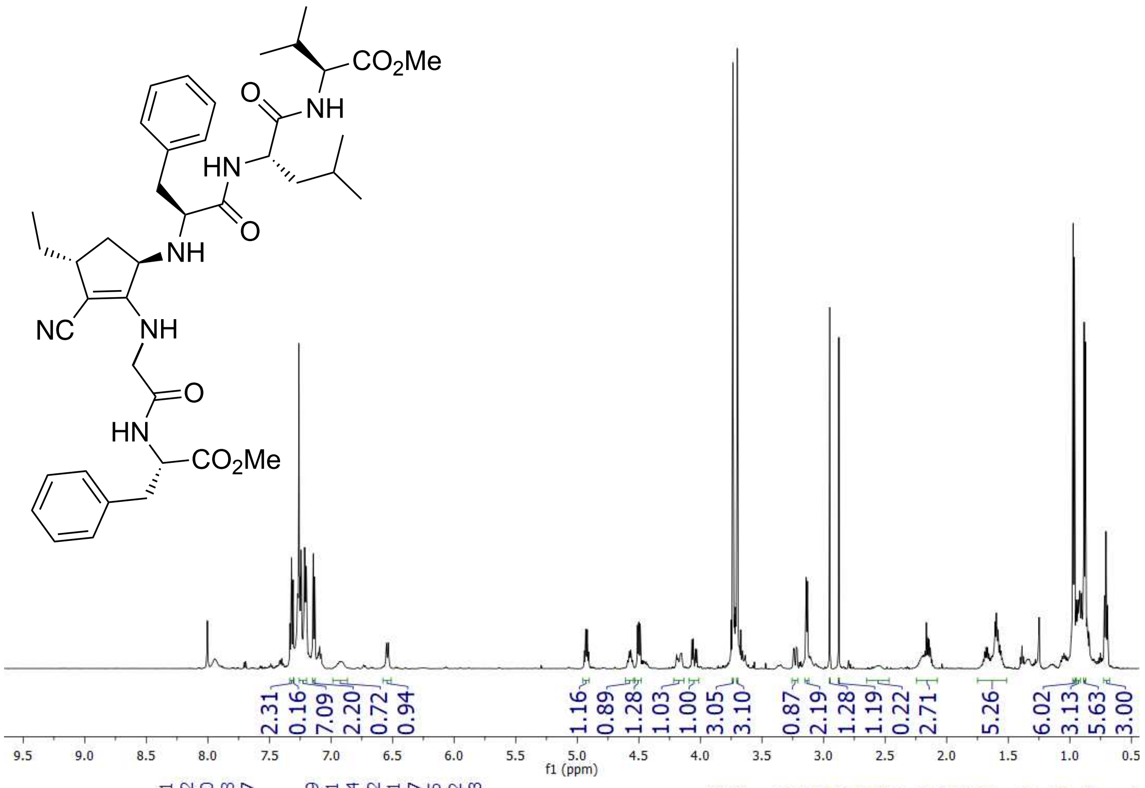

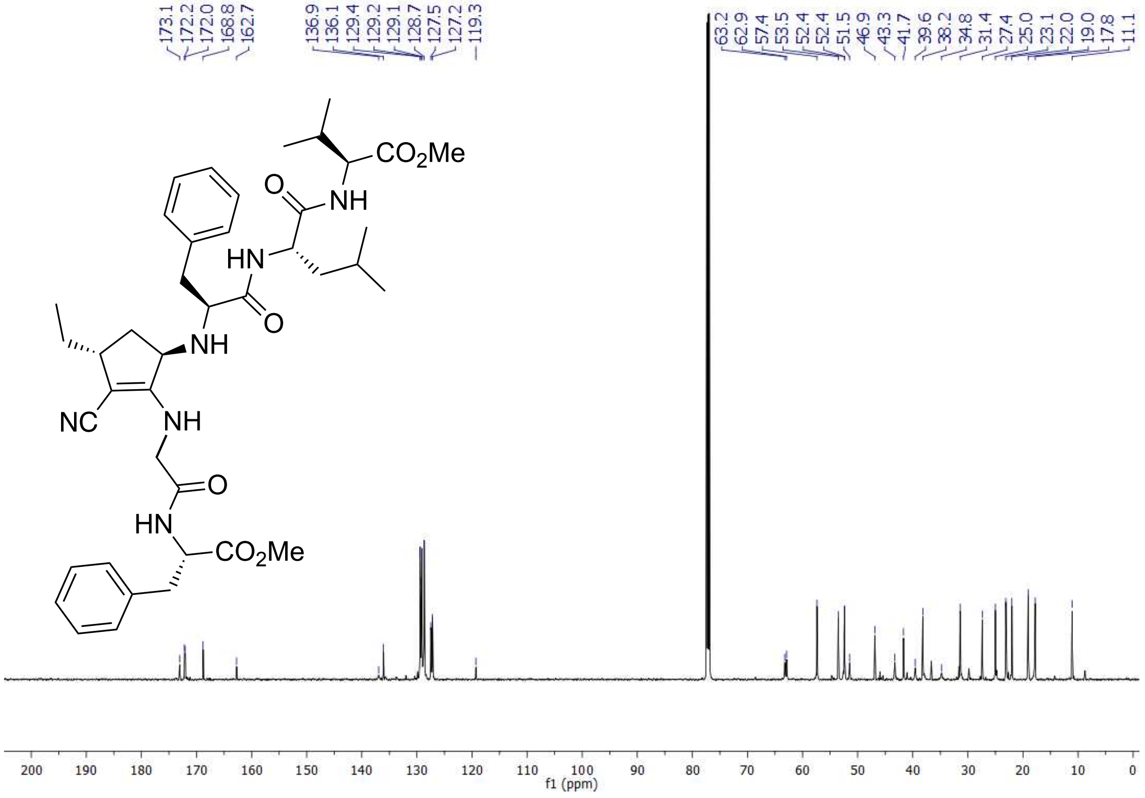

Figure S57. ¹H and ¹³C NMR spectra in $CDCl_3$ of compound **2ar**.

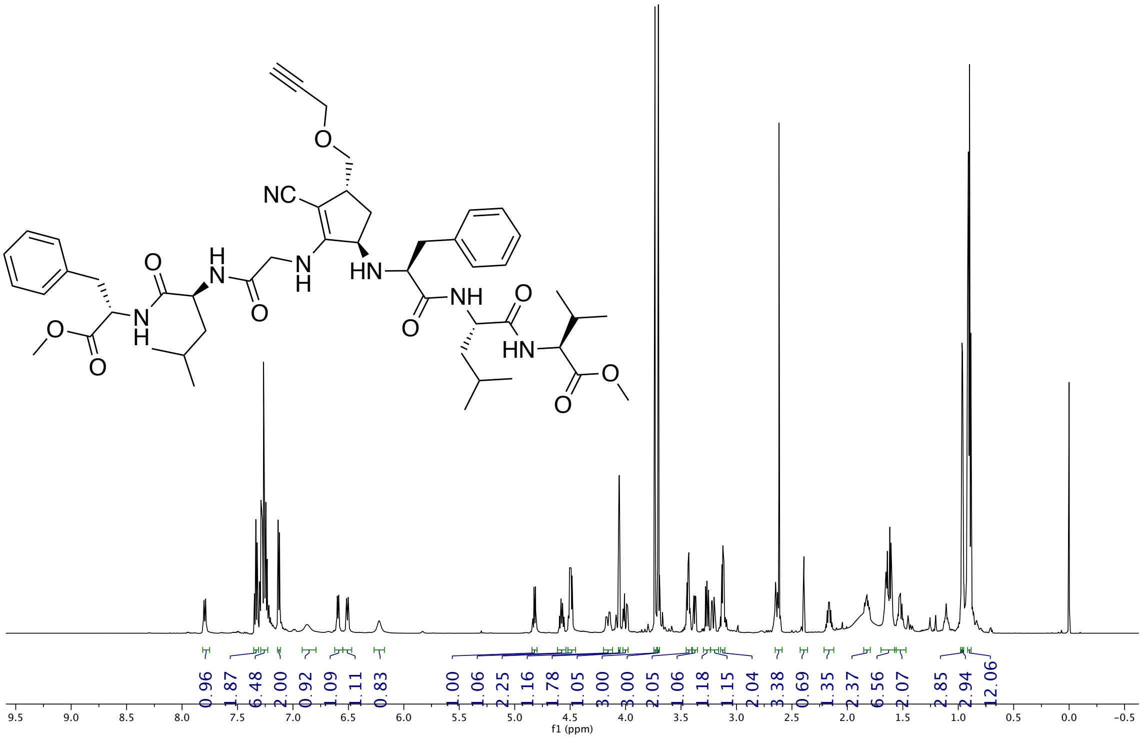

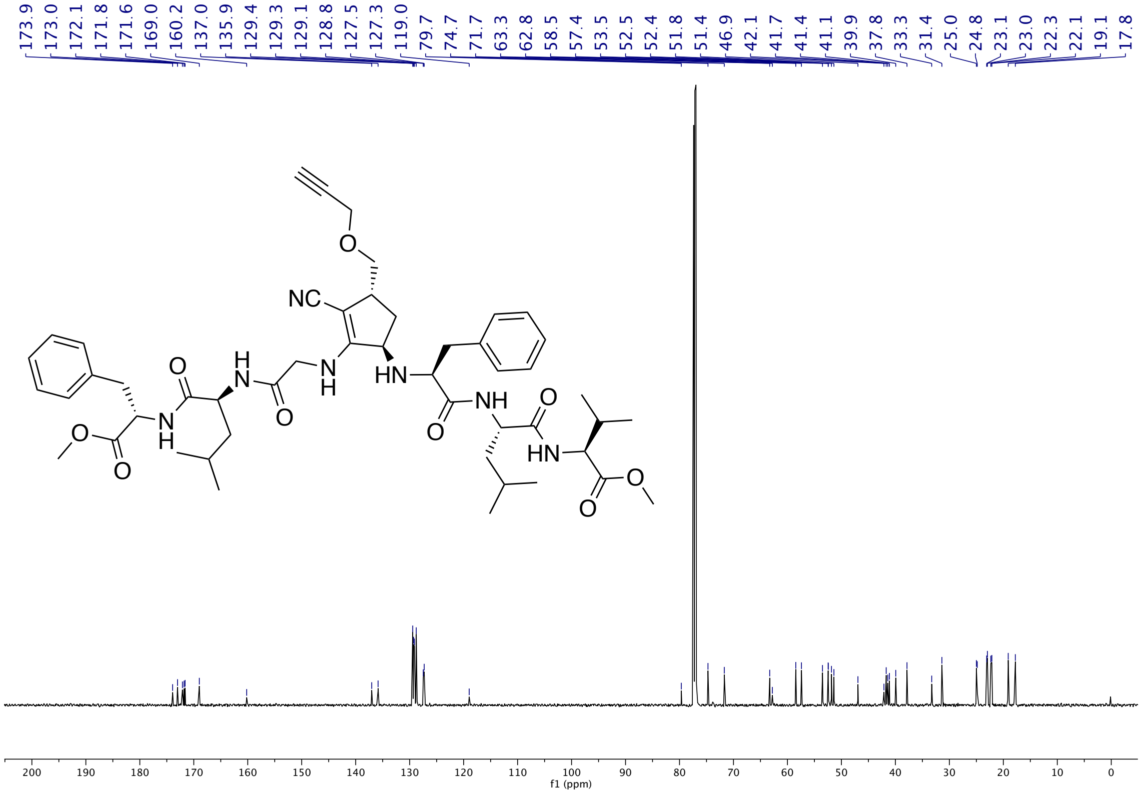

Figure S58. ^{1}H and ^{13}C NMR spectra in $CDCl_3$ of compound **2as**.

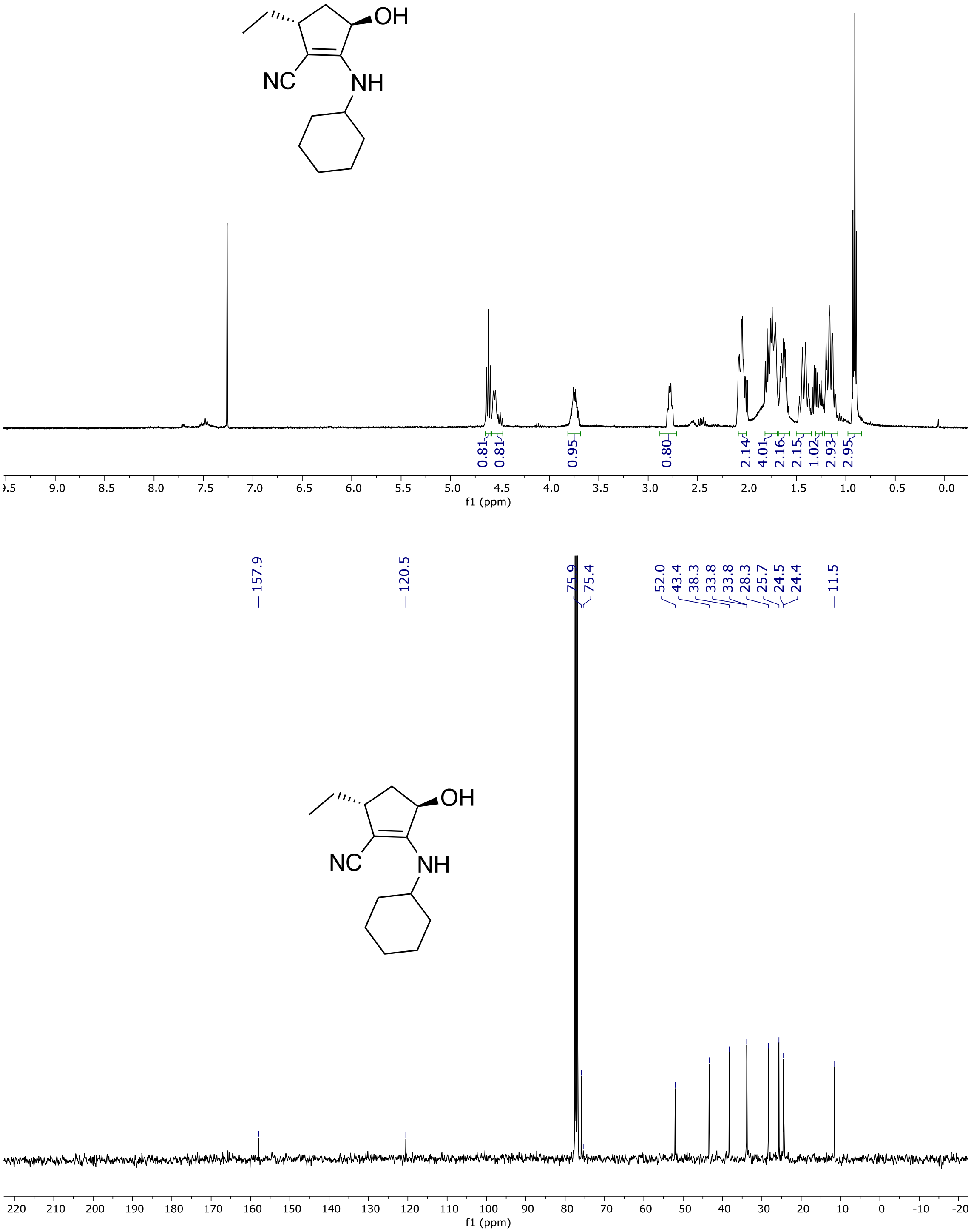

Figure S59. ^{1}H and ^{13}C NMR spectra in $CDCl_3$ of compound **3a**.

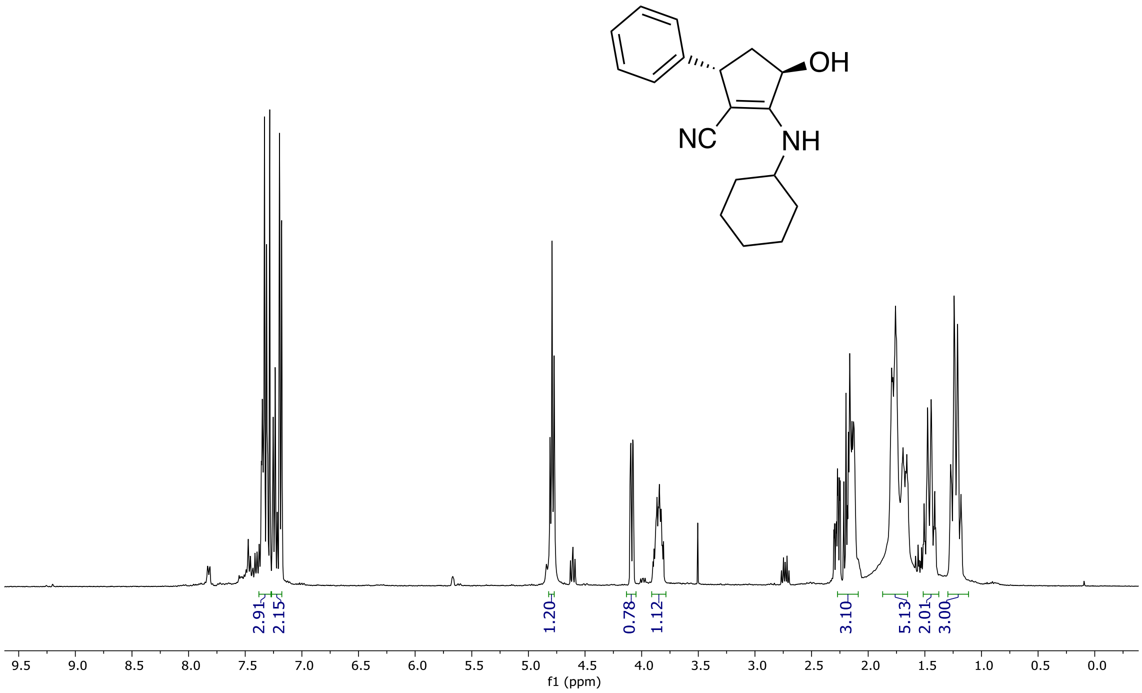

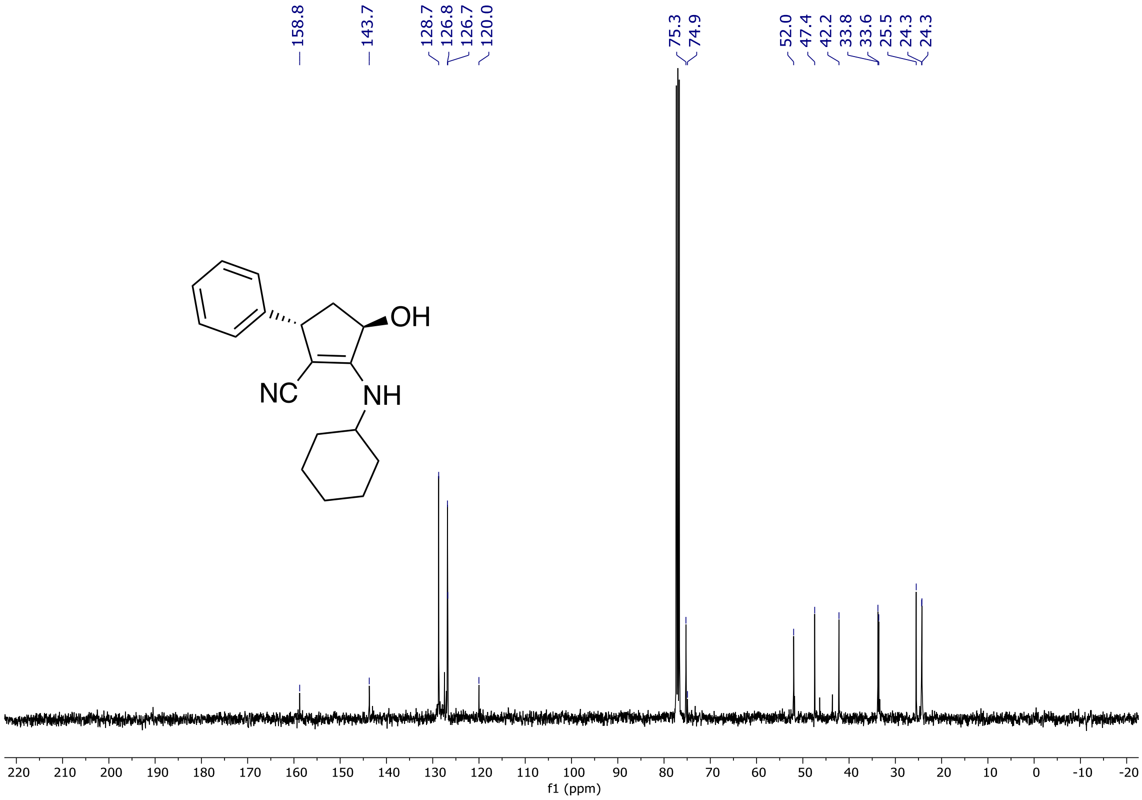

Figure S60. ^{1}H and ^{13}C NMR spectra in $CDCl_3$ of compound **3b**.

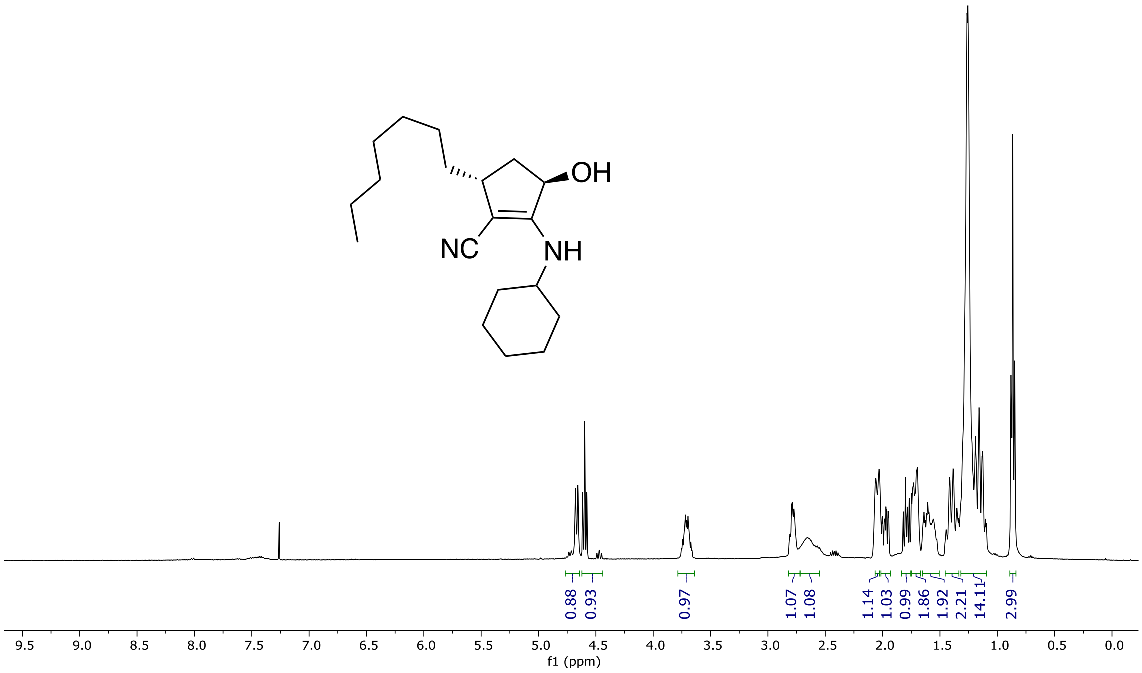

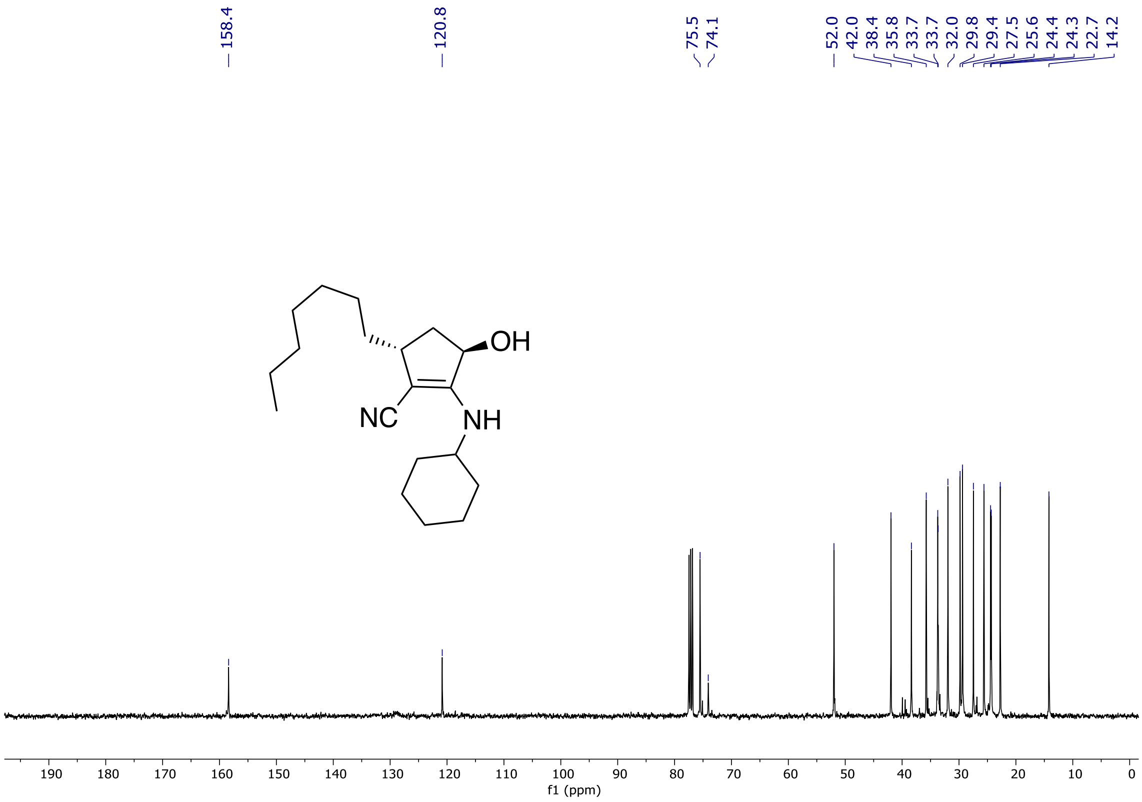

Figure S61. ^{1}H and ^{13}C NMR spectra in $CDCl_3$ of compound **3c**.

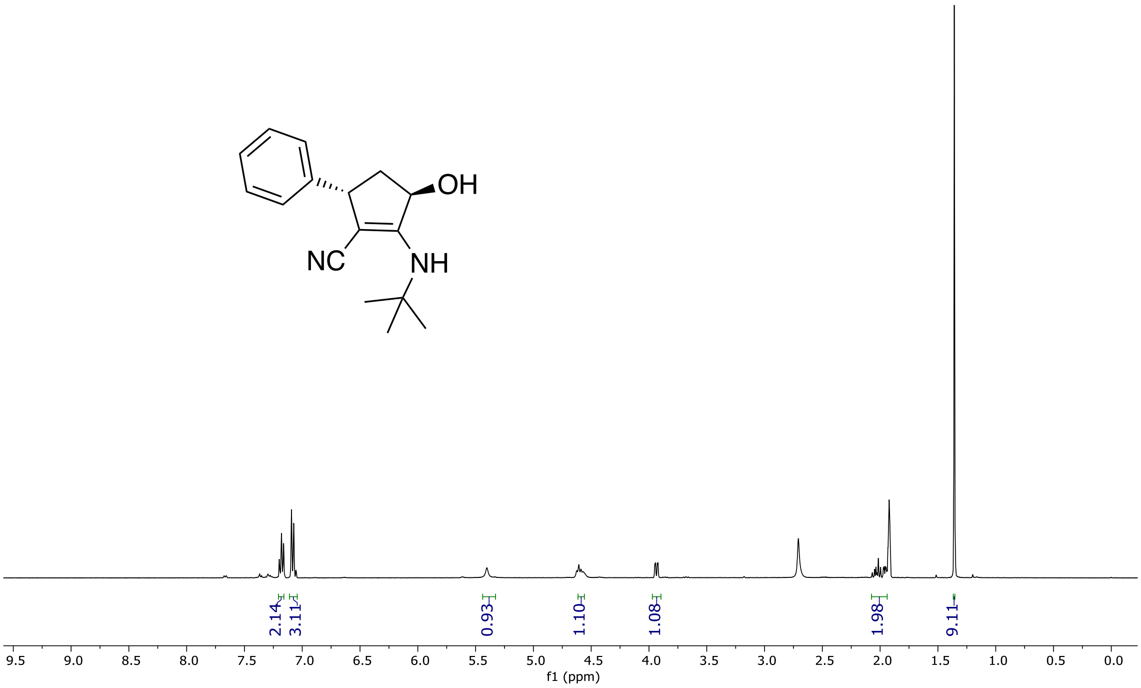

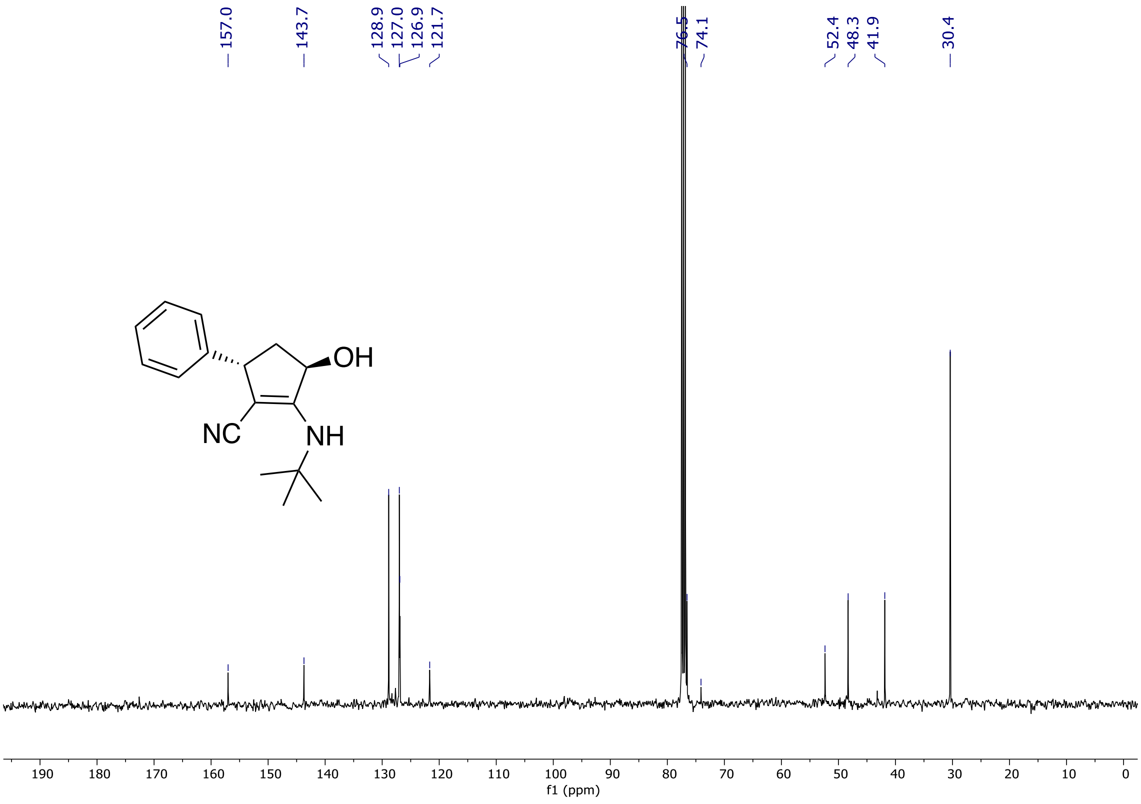

Figure S62. ^{1}H and ^{13}C NMR spectra in acetone-d^6 and $CDCl_3$, respectively, of compound of **3d**.

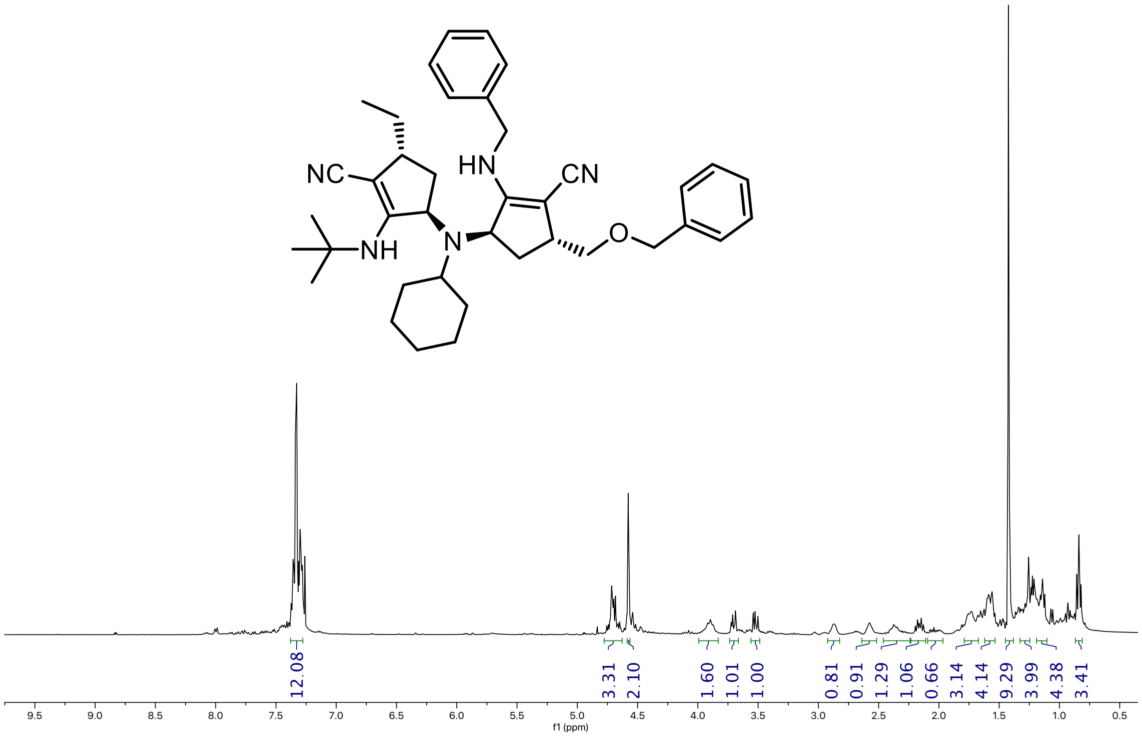

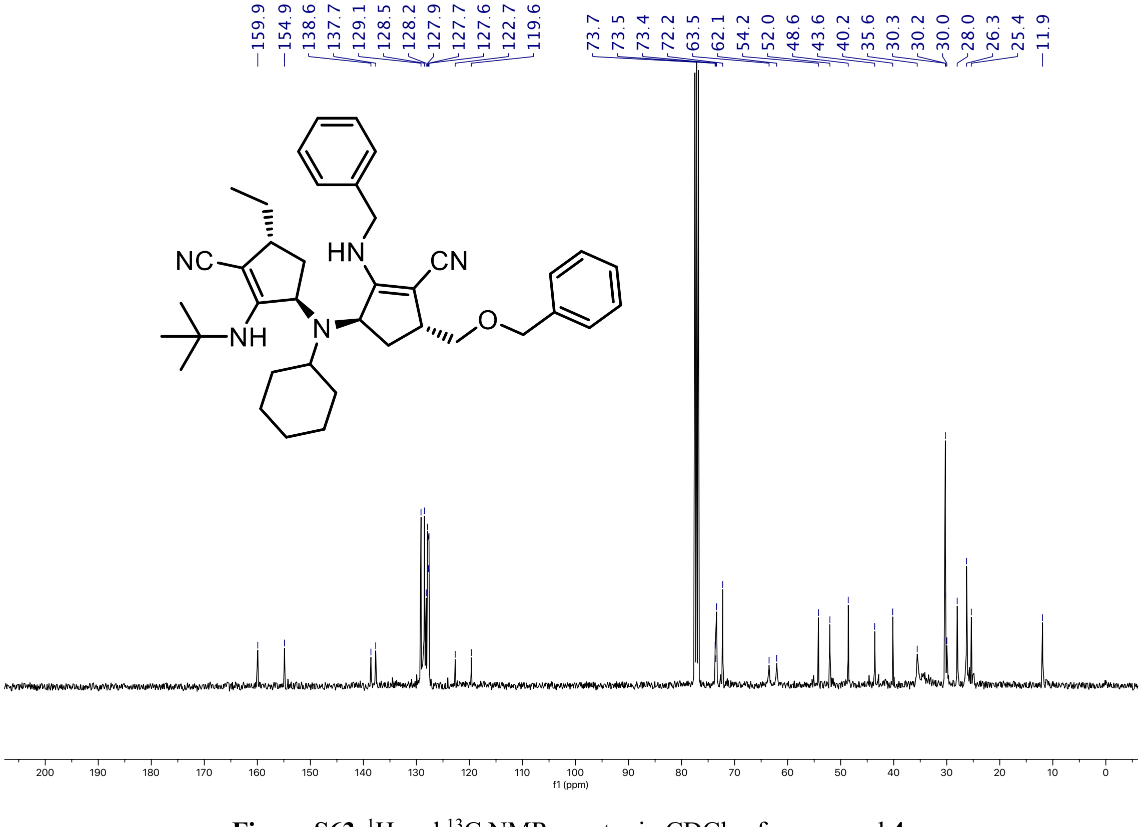

Figure S63. ^{1}H and ^{13}C NMR spectra in $CDCl_3$ of compound **4**.

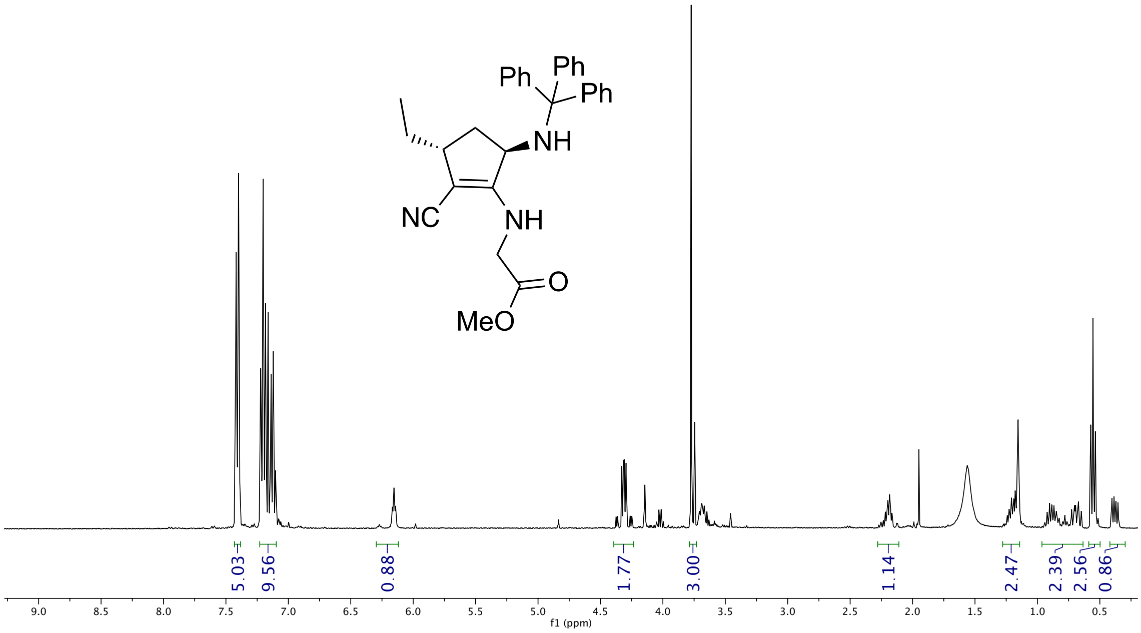

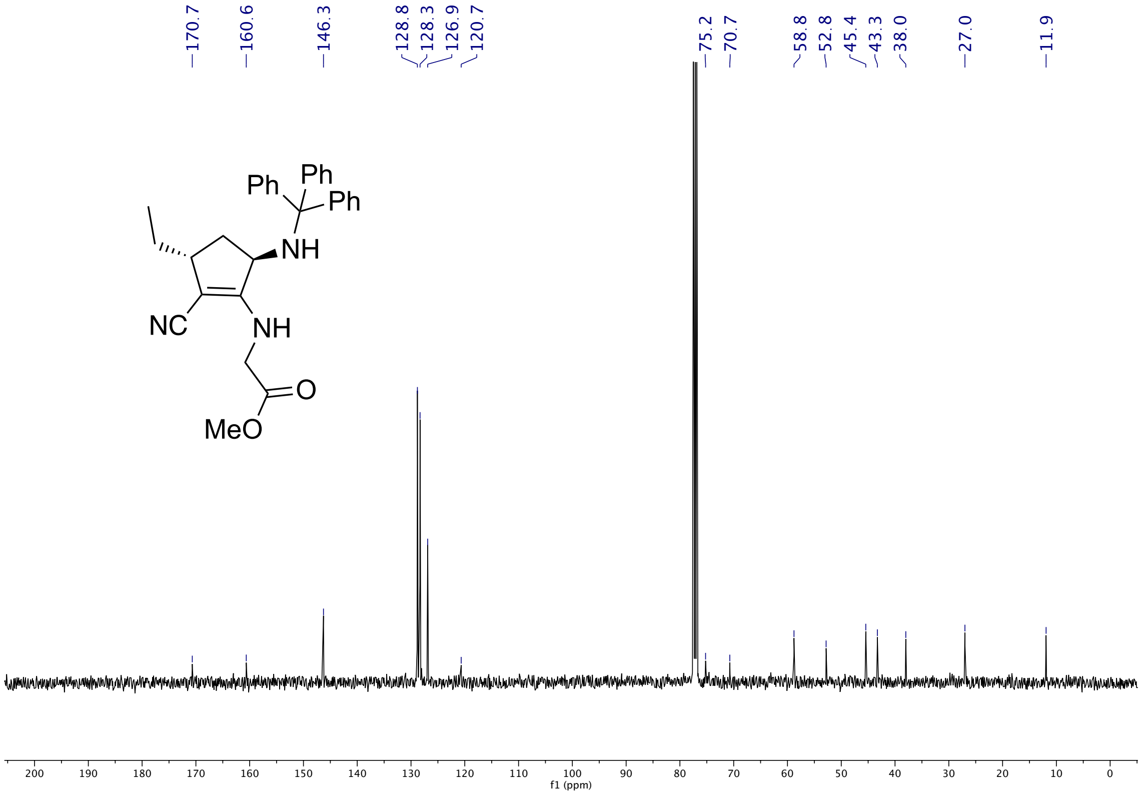

Figure S64. ^{1}H and ^{13}C NMR spectra in $CDCl_3$ of compound **5**.

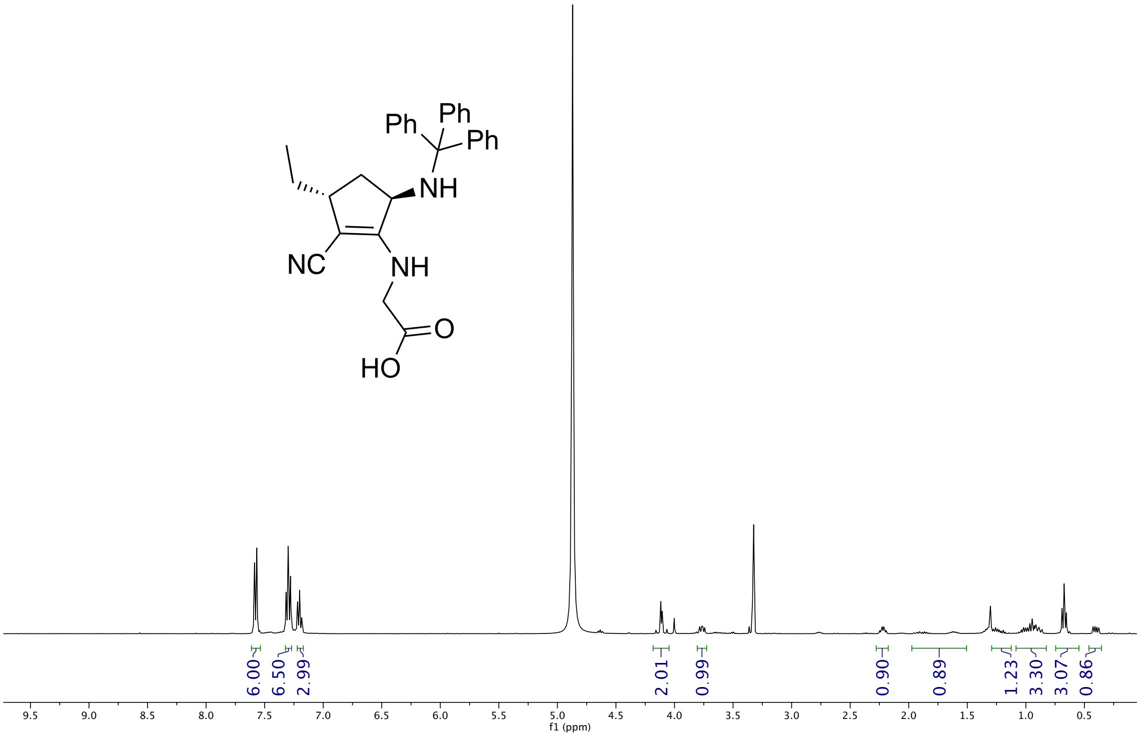

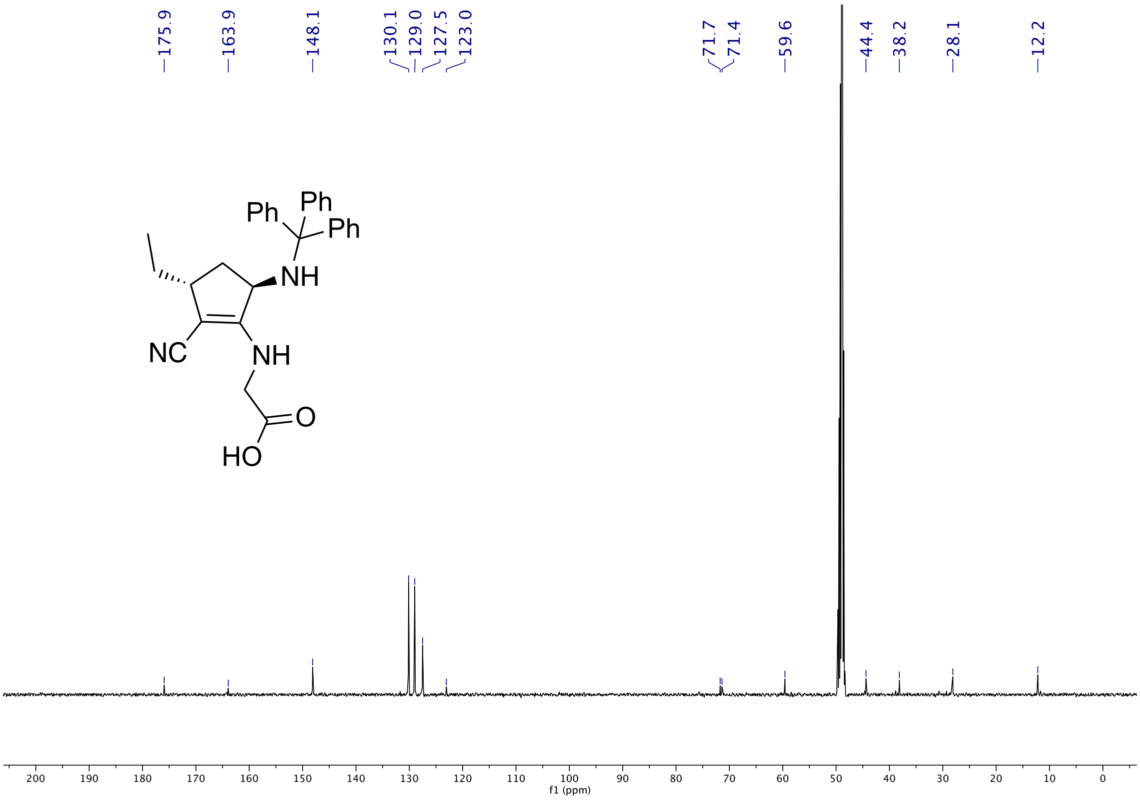

Figure S65. ^{1}H and ^{13}C NMR spectra in CD_3OD of compound **6**.

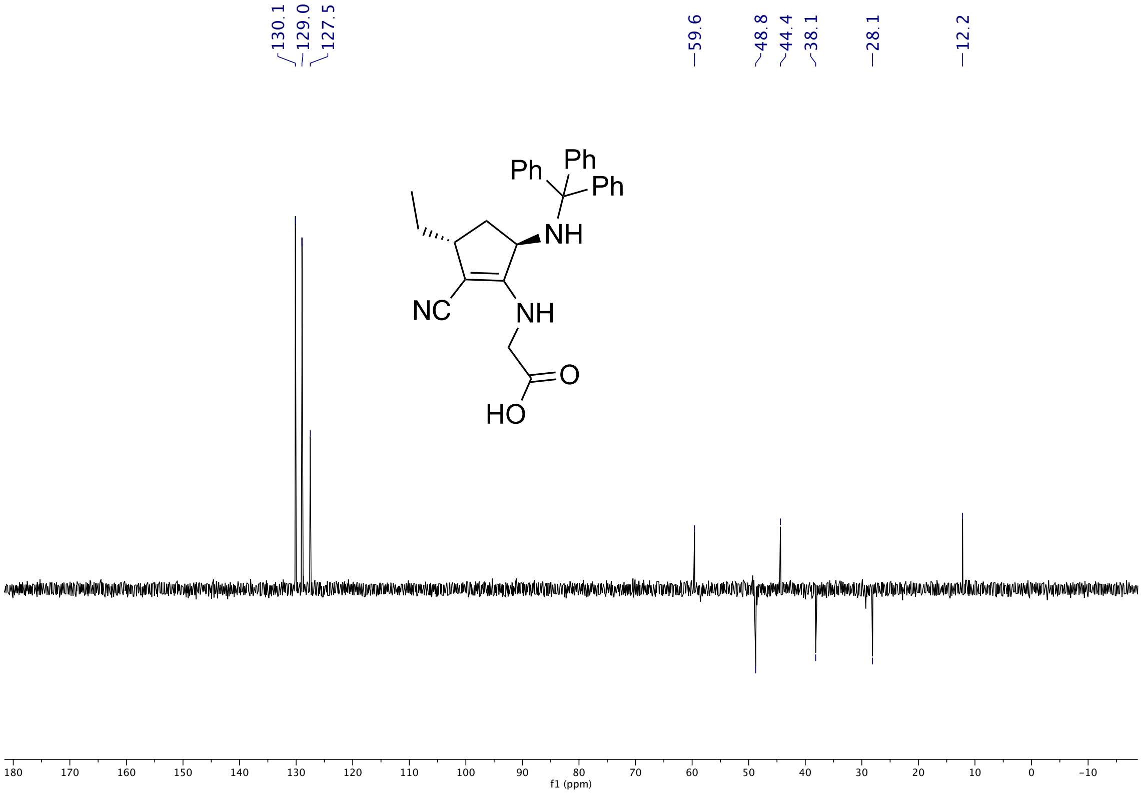

Figure S66. ^{13}C DEPT 135 spectrum in CD_3OD of compound **6**.

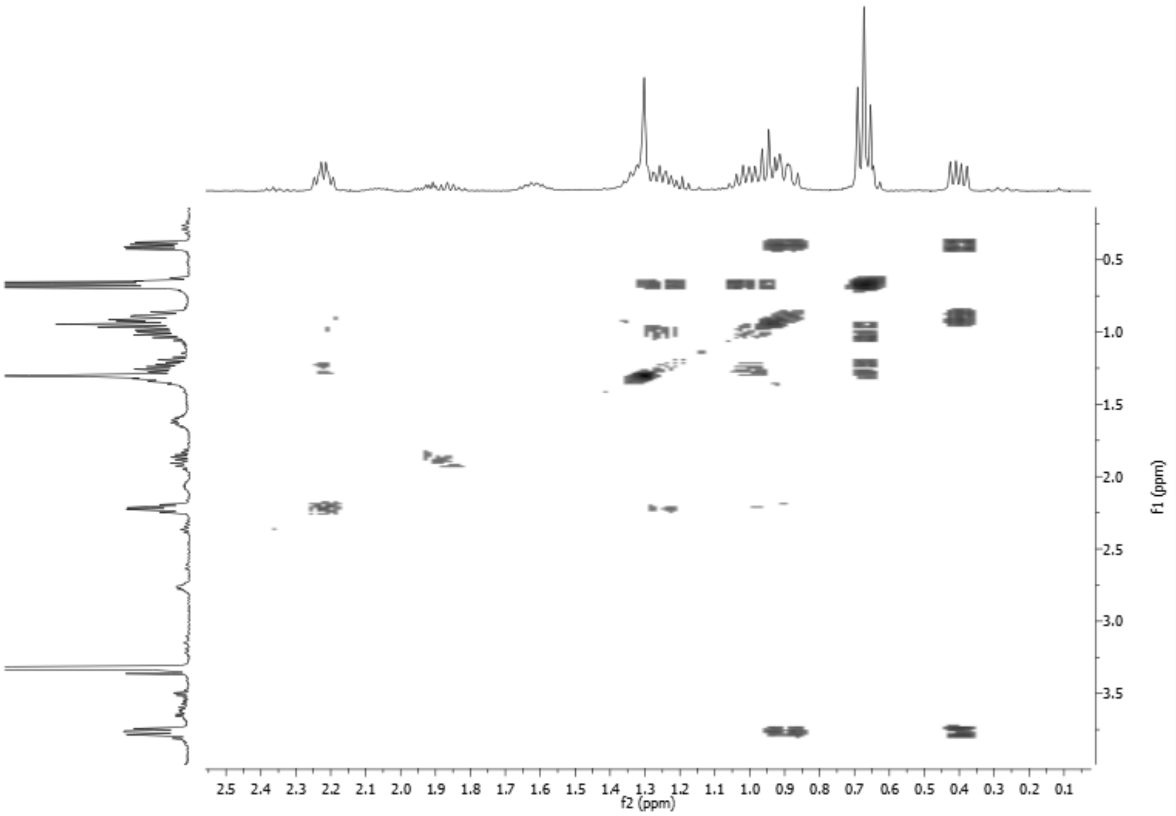

Figure S67. ^{1}H COSY spectrum in CD_3OD of compound **6**.

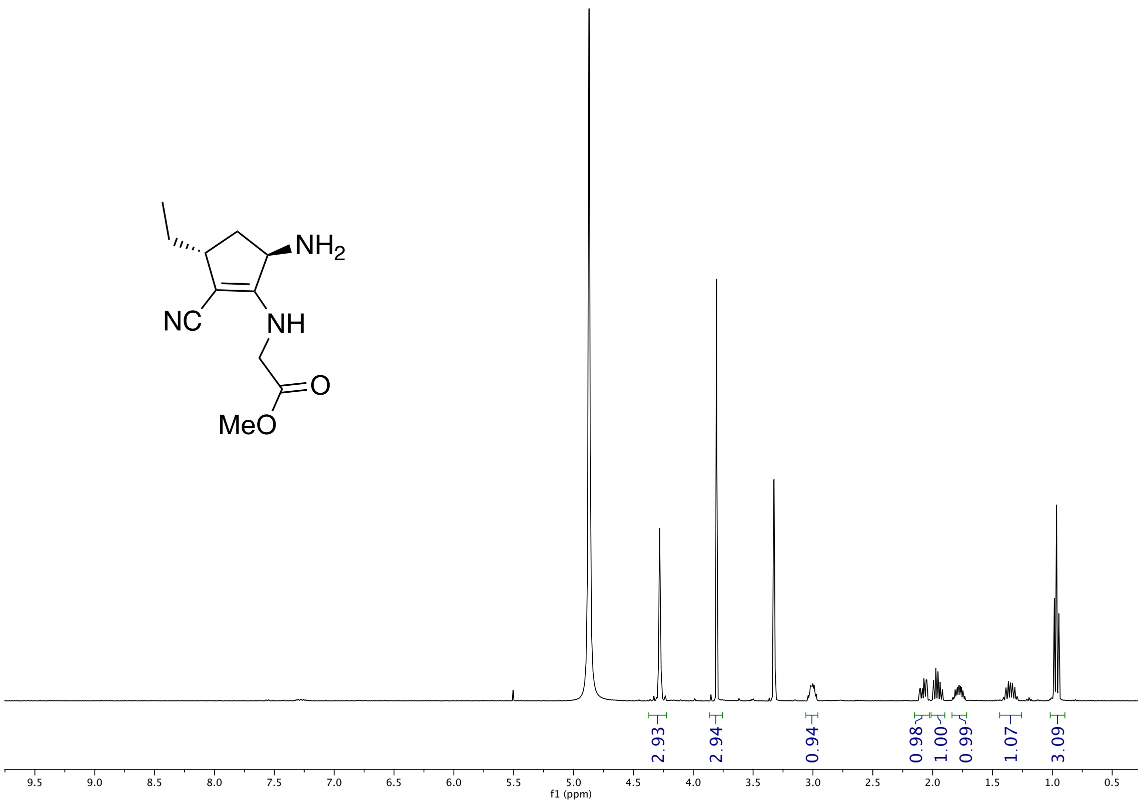

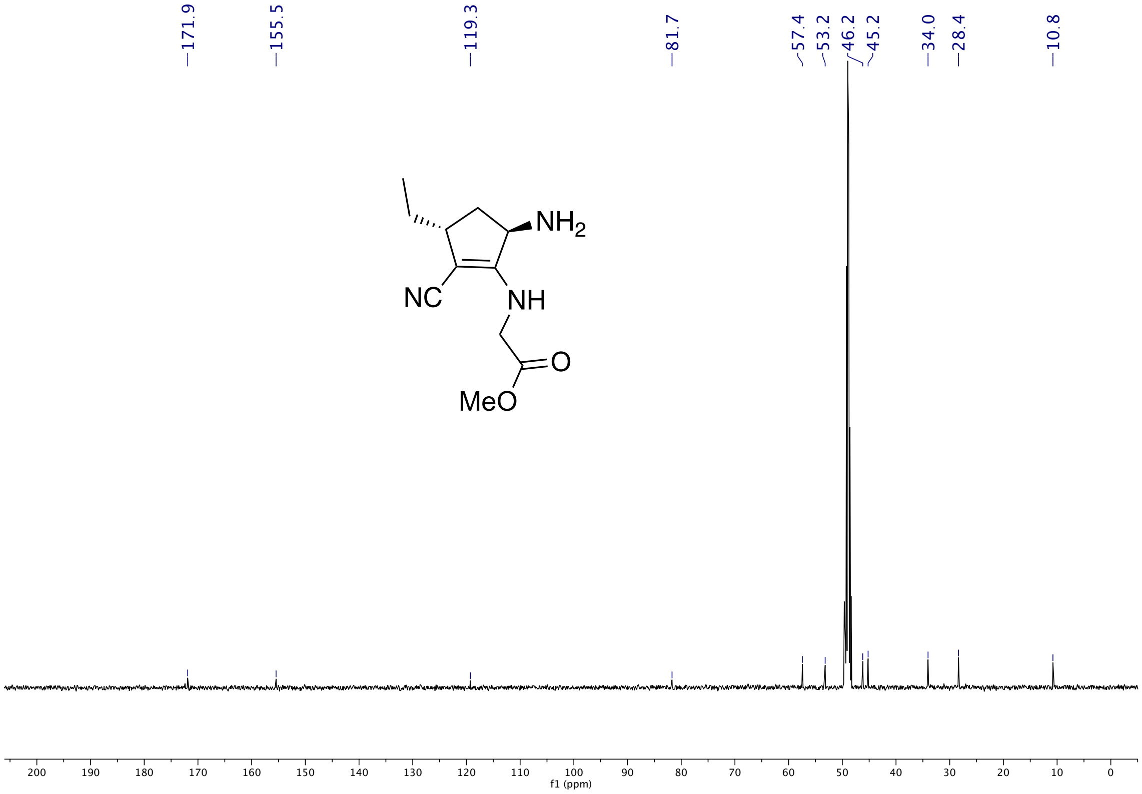

Figure S68. ^{1}H and ^{13}C NMR spectra in CD_3OD of compound **7**.

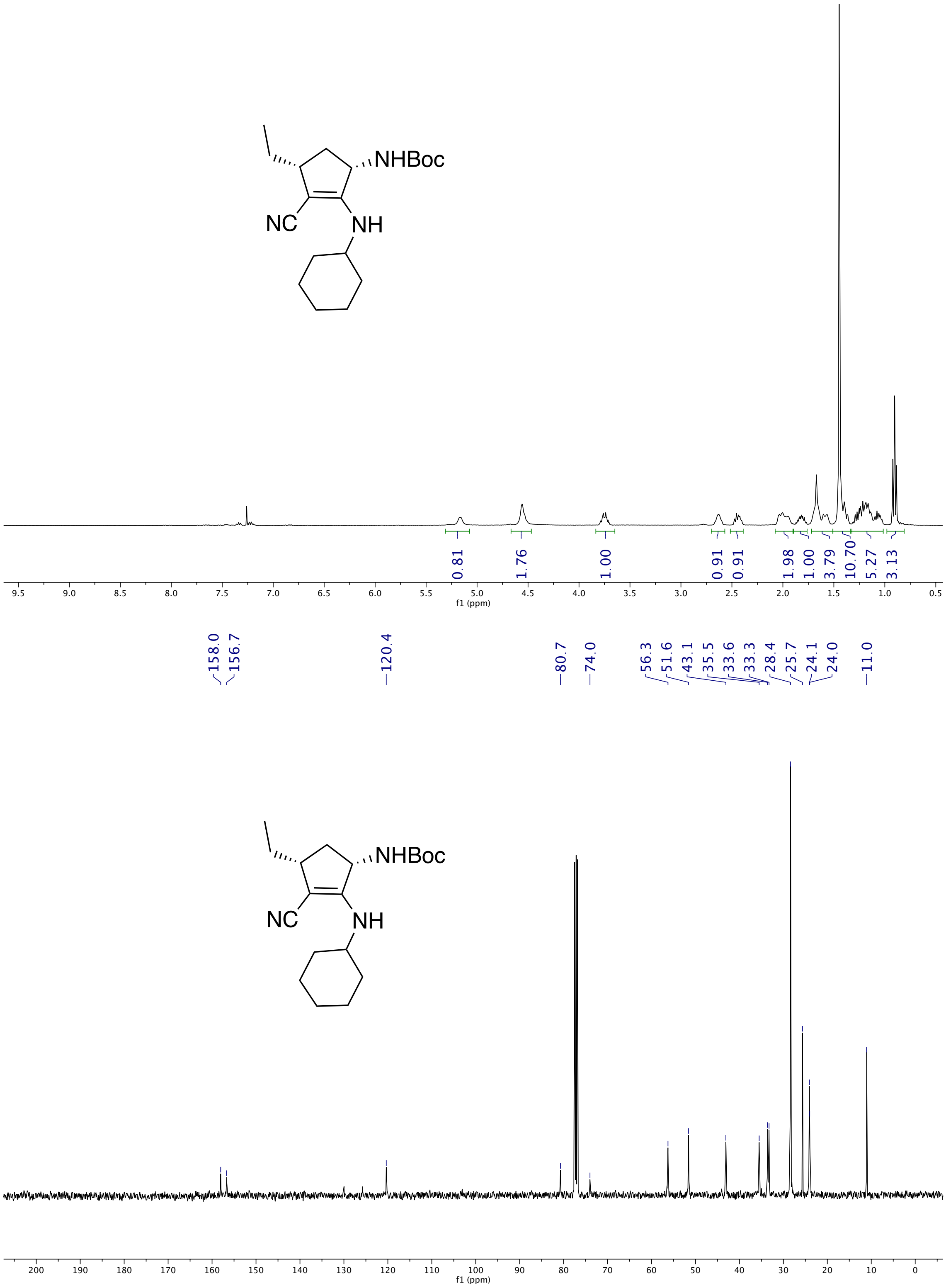

Figure S69. ^{1}H and ^{13}C NMR spectra in $CDCl_3$ of compound **8**.

UPC^2 and HPLC chromatograms

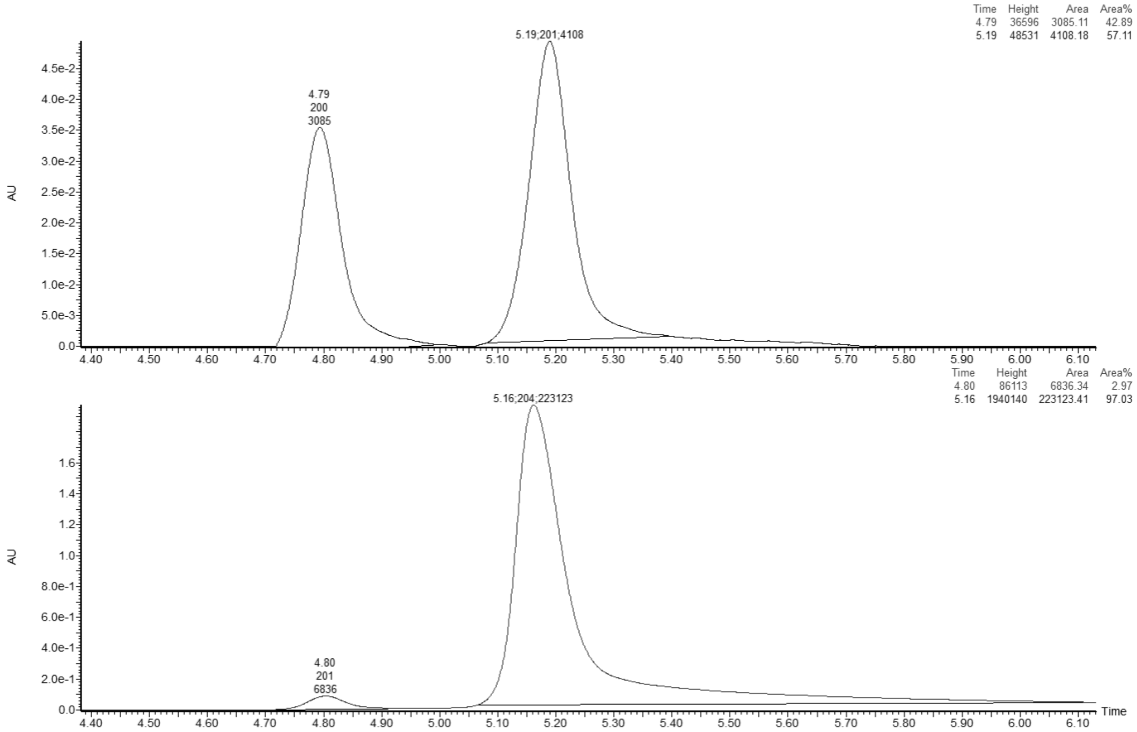

Figure S70. Chromatograms of compound **1a**.

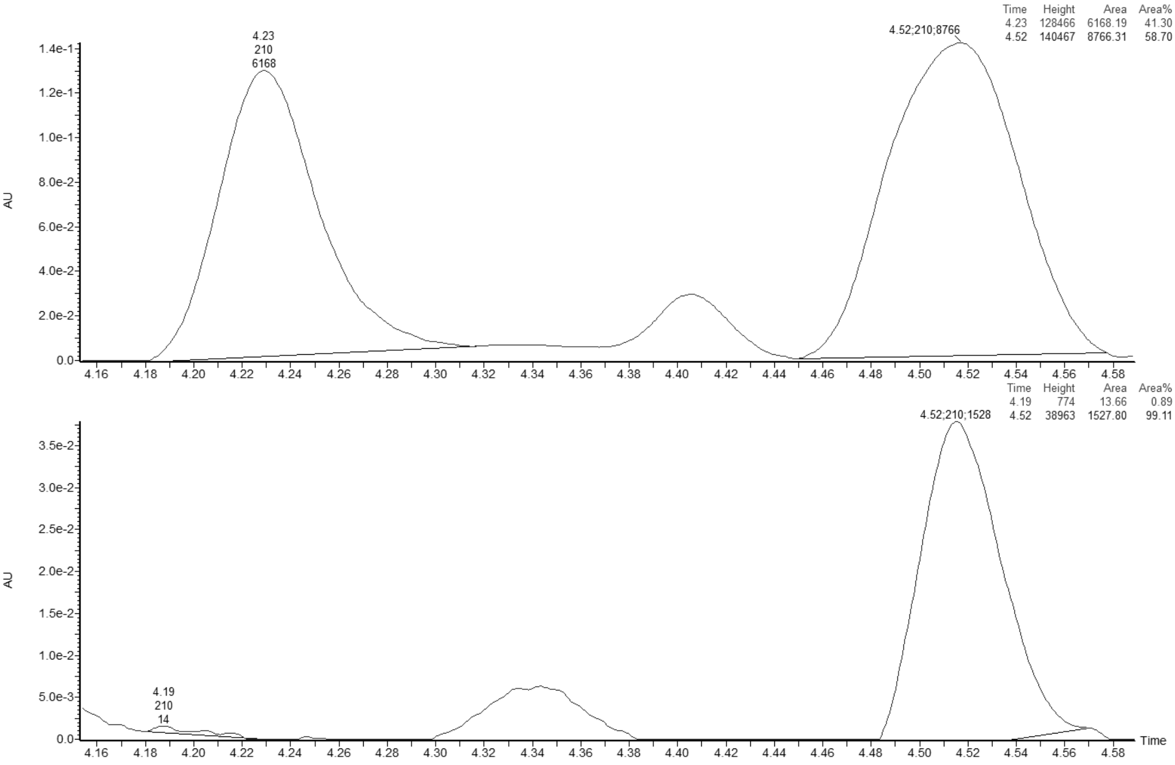

Figure S71. Chromatograms of compound **1b**.

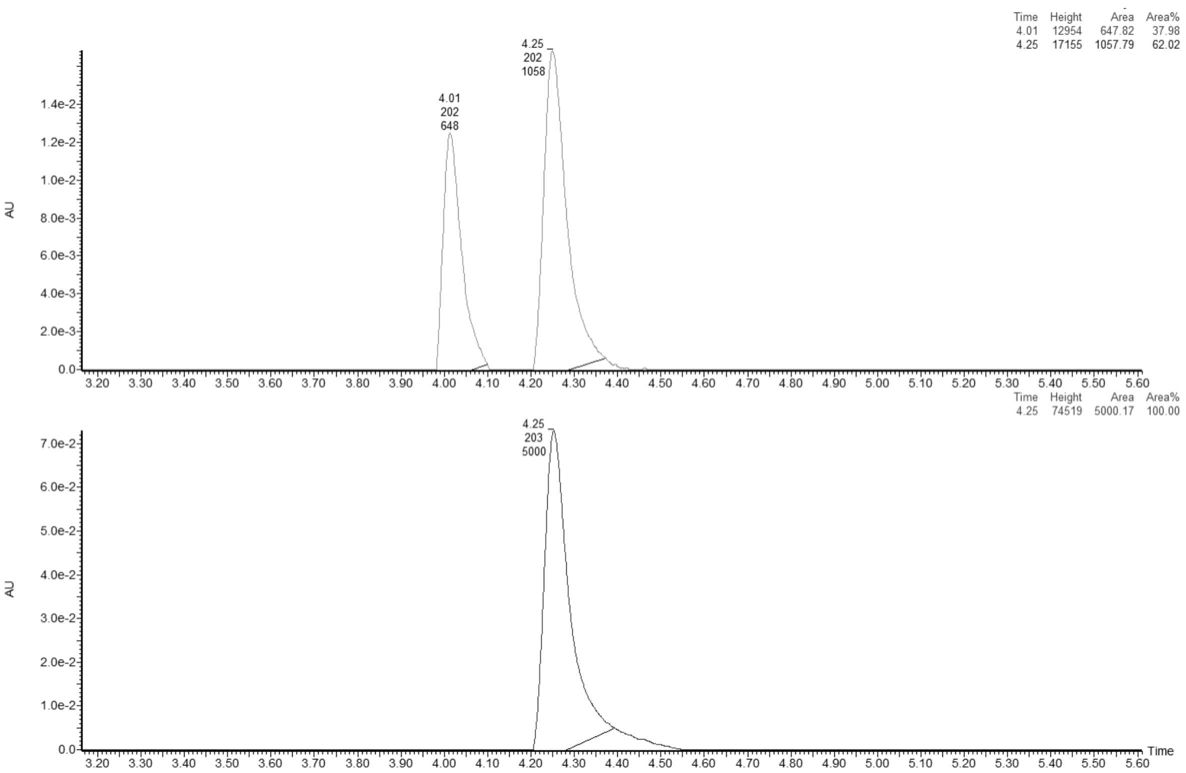

Figure S72. Chromatograms of compound **1c**.

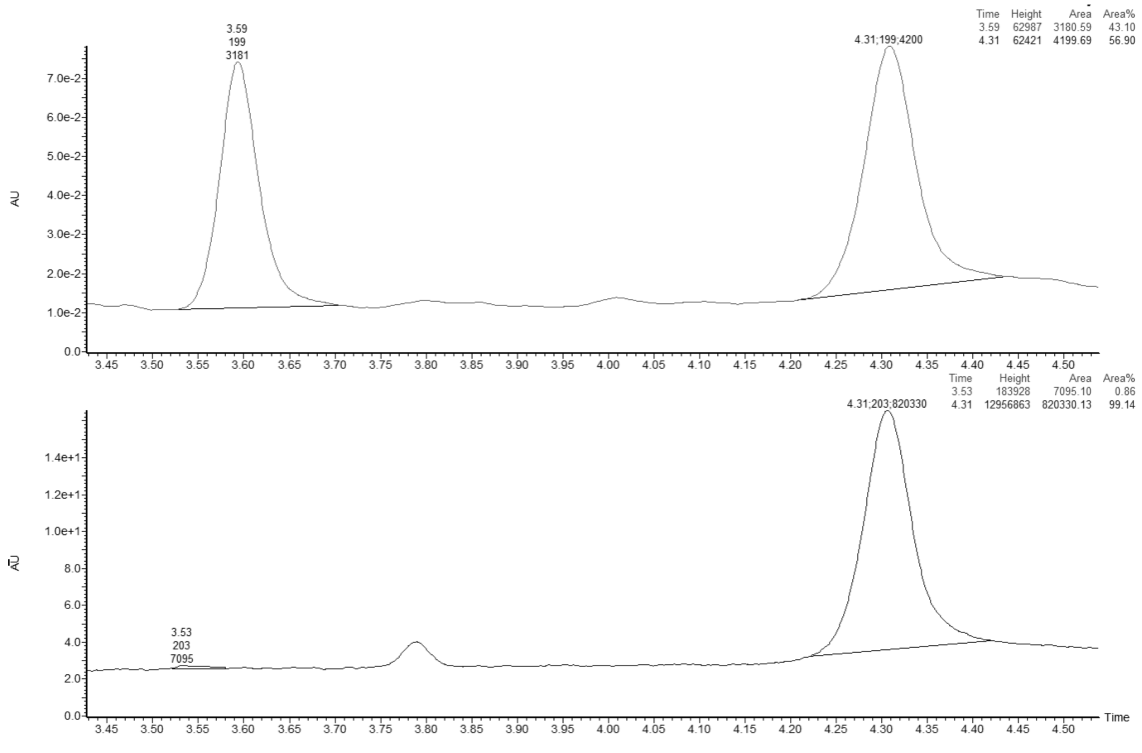

Figure S73. Chromatograms of compound **1d**.

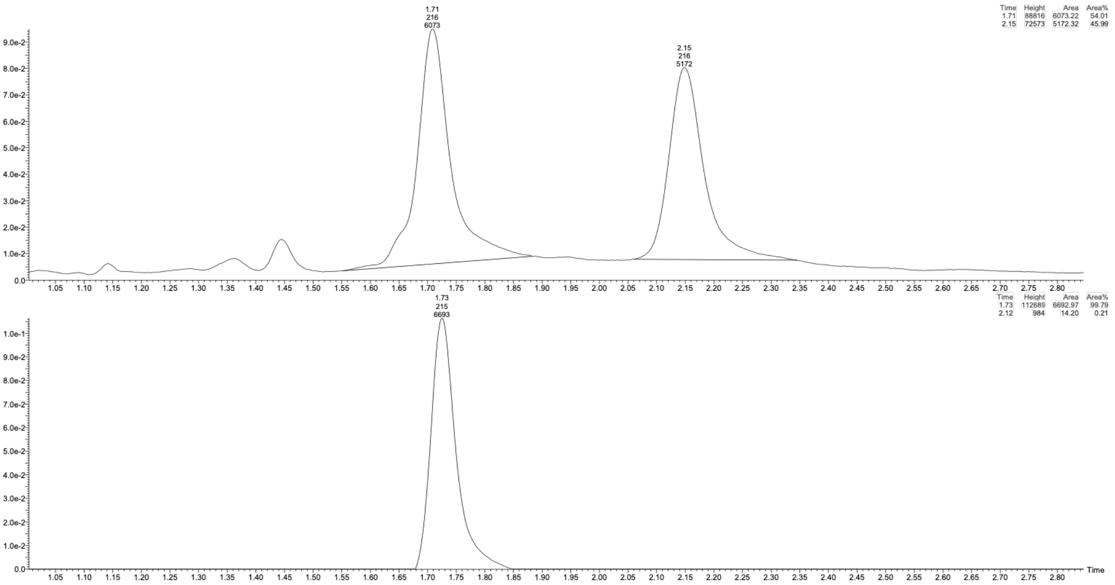

Figure S74. Chromatograms of compound **1e**.

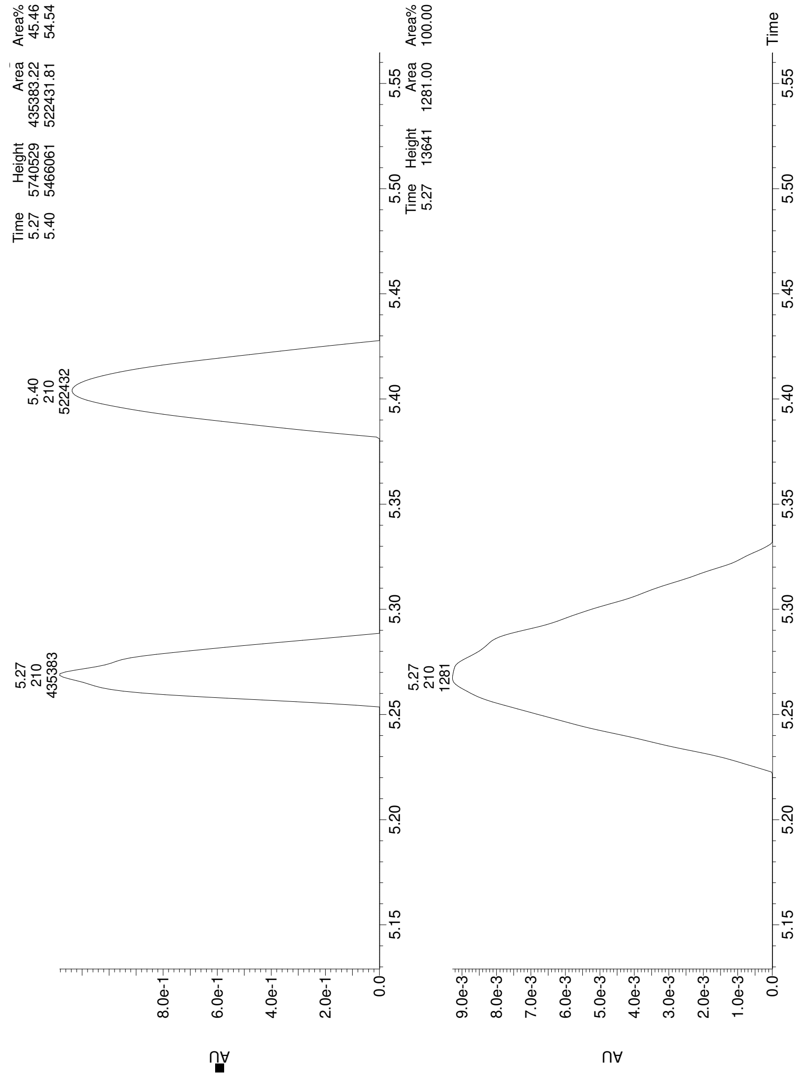

Figure S75. Chromatograms of compound **1f**.

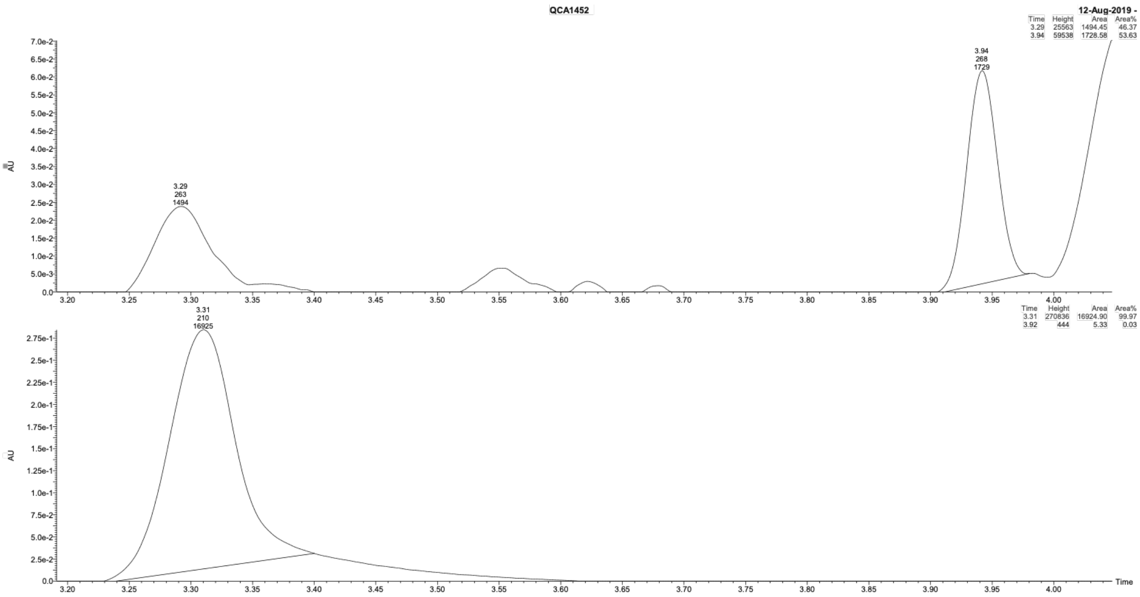

Figure S76. Chromatograms of compound 1g.

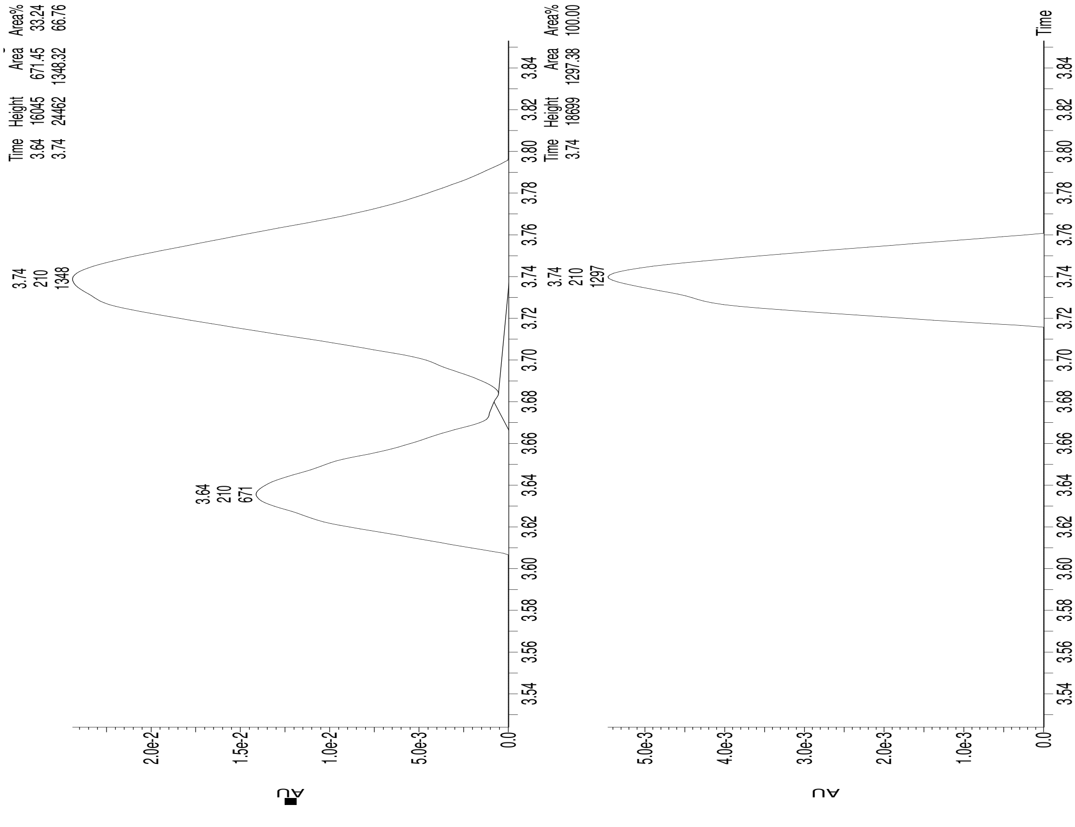

Figure S77. Chromatograms of compound **1h**.

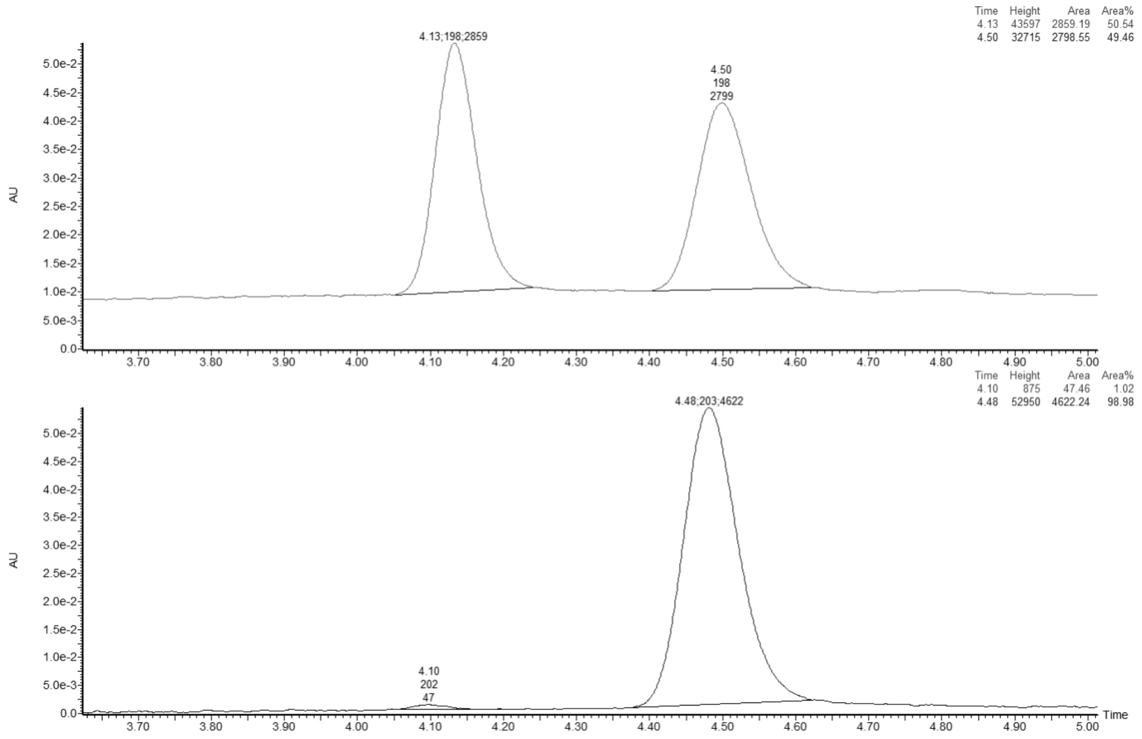

Figure S78. Chromatograms of compound **1i**.

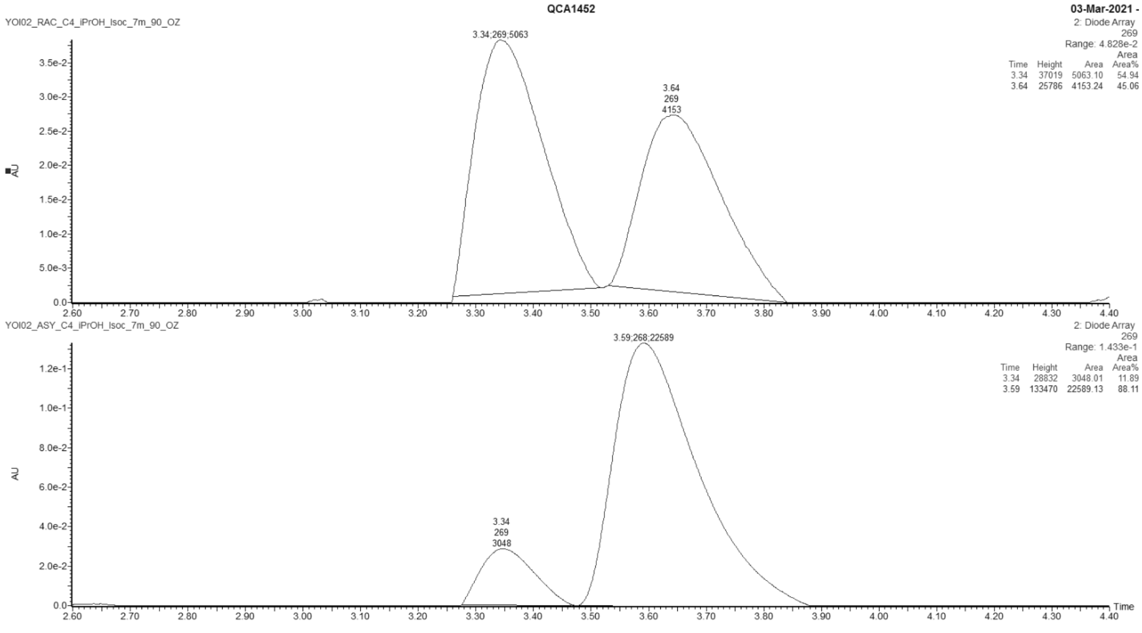

Figure S79. Chromatograms of compound **2a**.

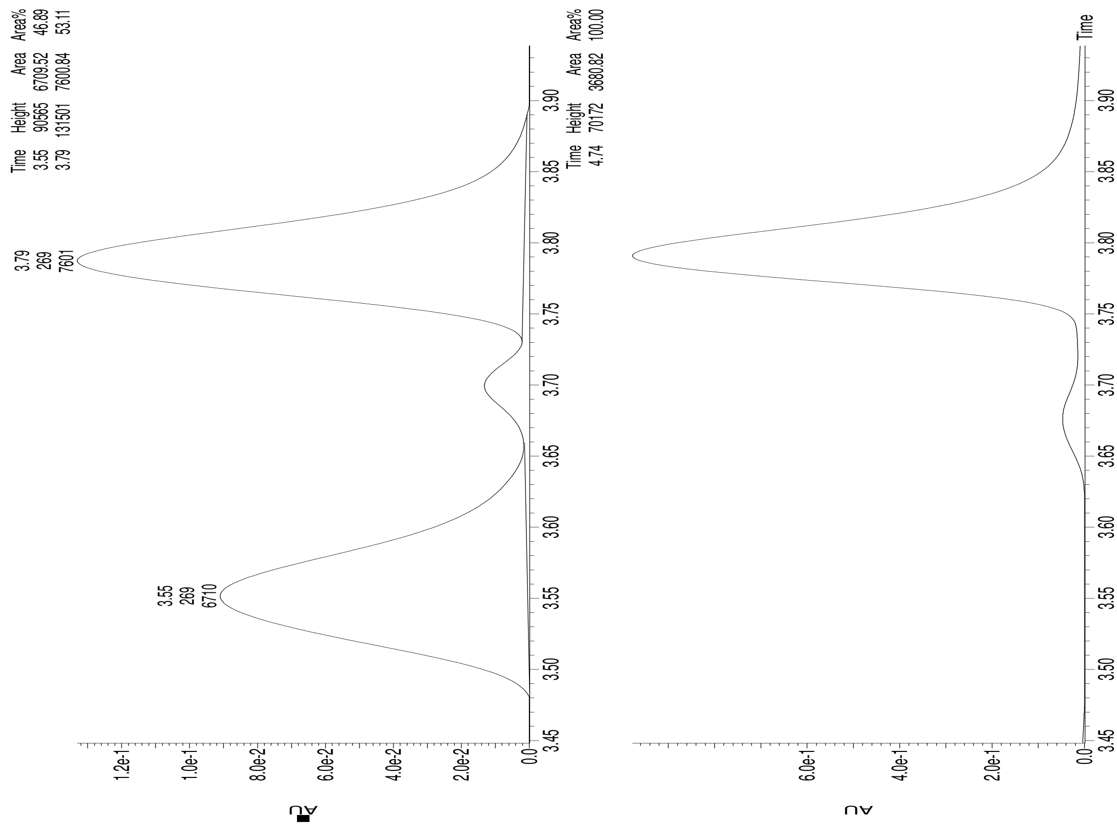

Figure S80. Chromatograms of compound **2b**.

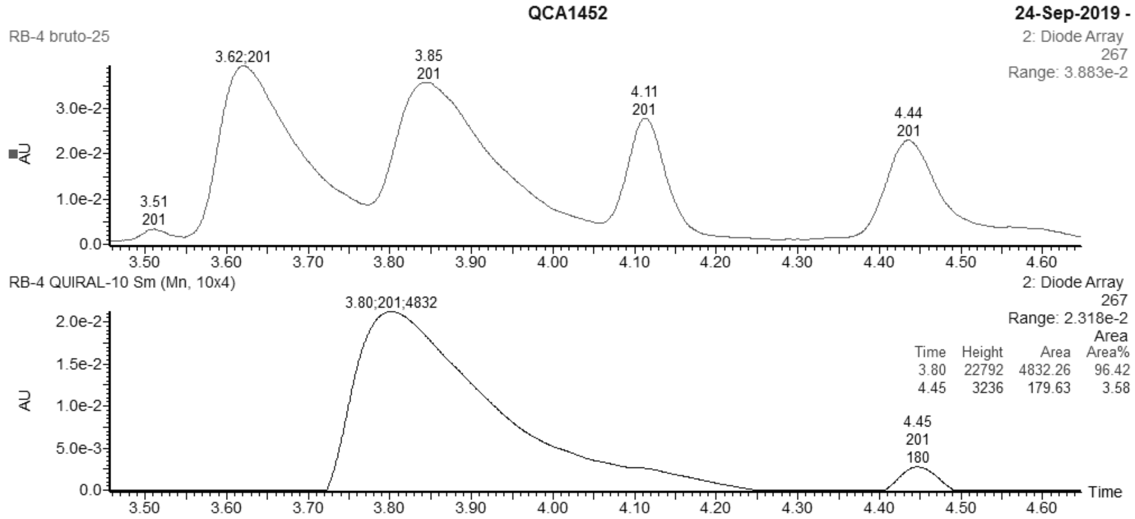

Figure S81. Chromatograms of compound **2c**.

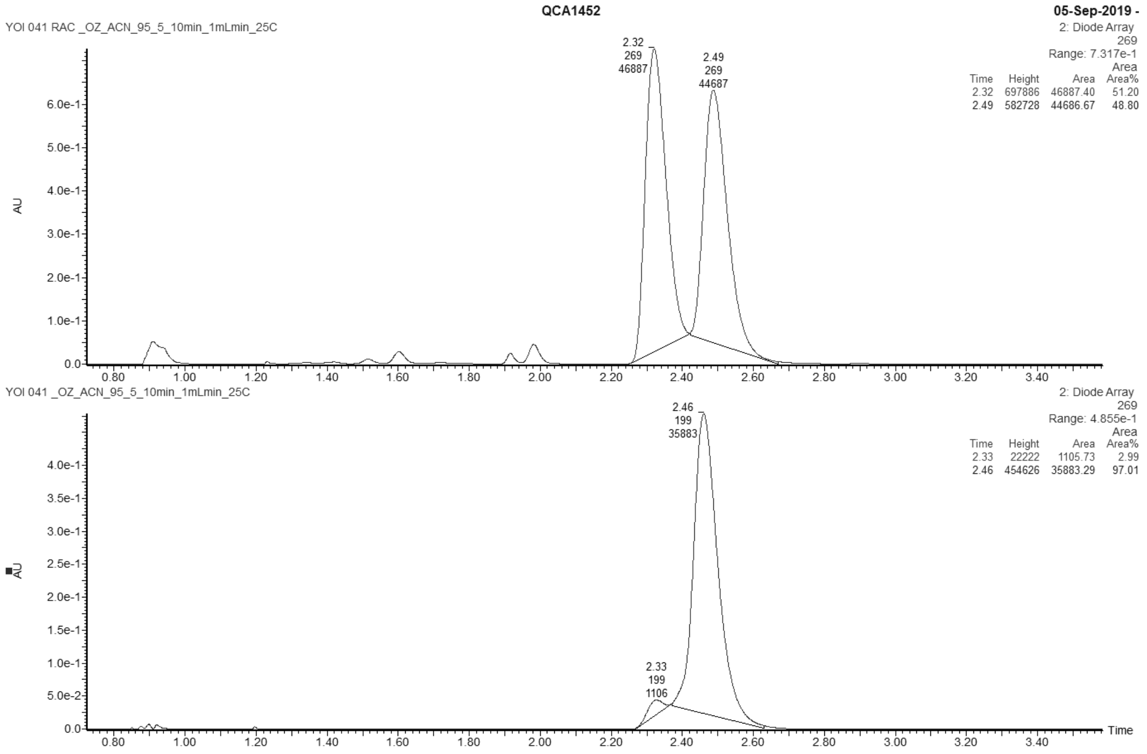

Figure S82. Chromatograms of compound **2d**.

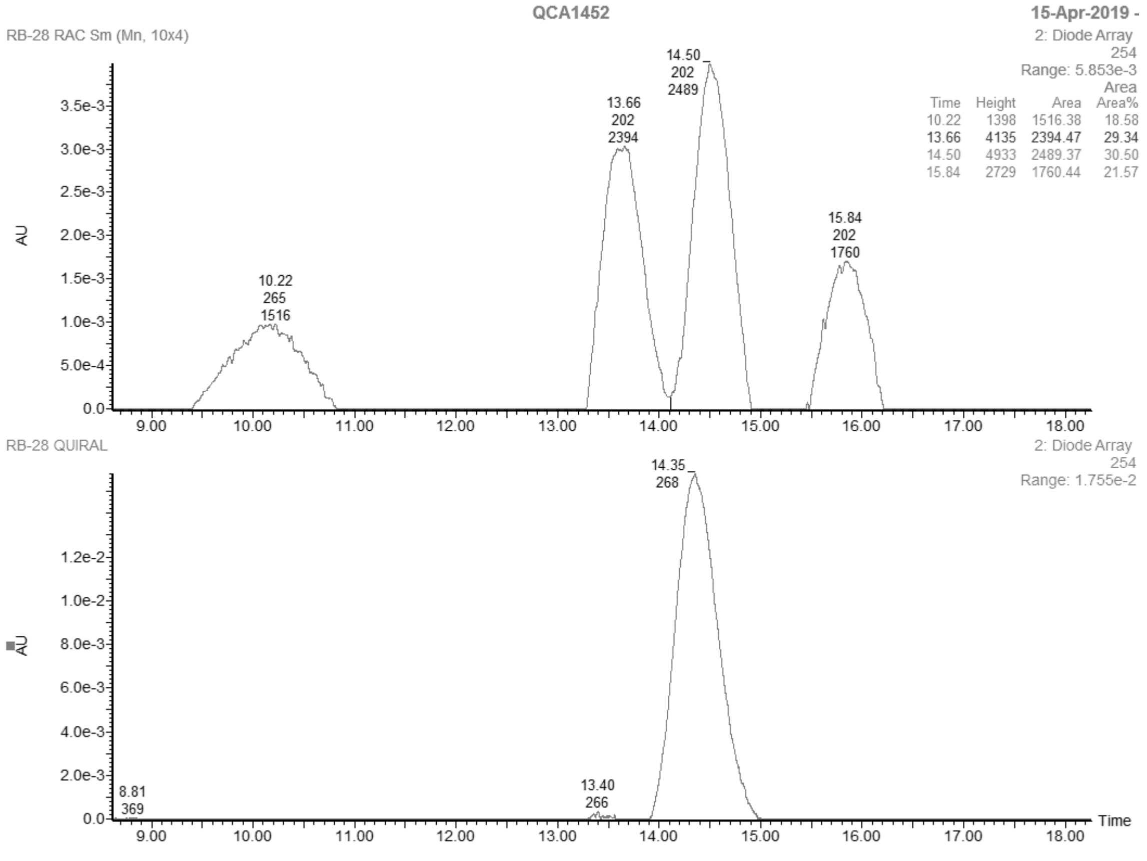

Figure S83. Chromatograms of compound **2e**.

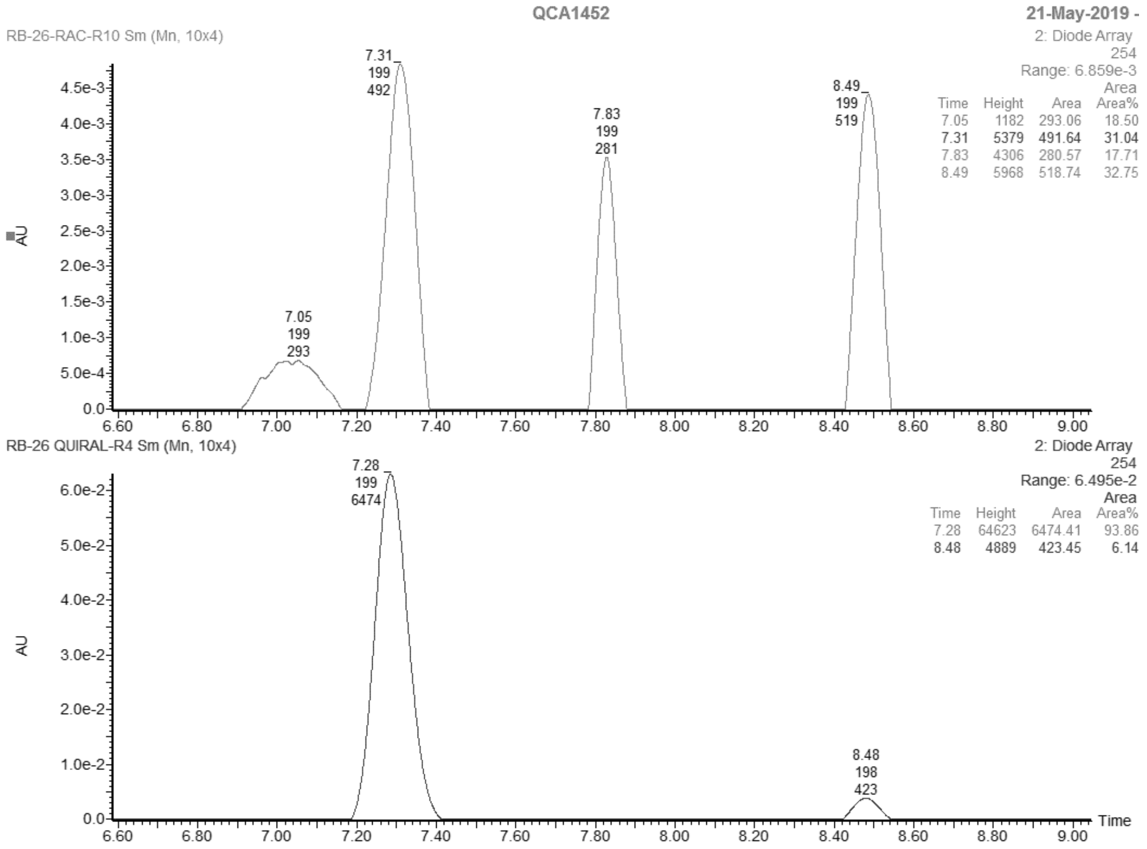

Figure S84. Chromatograms of compound **2f**.

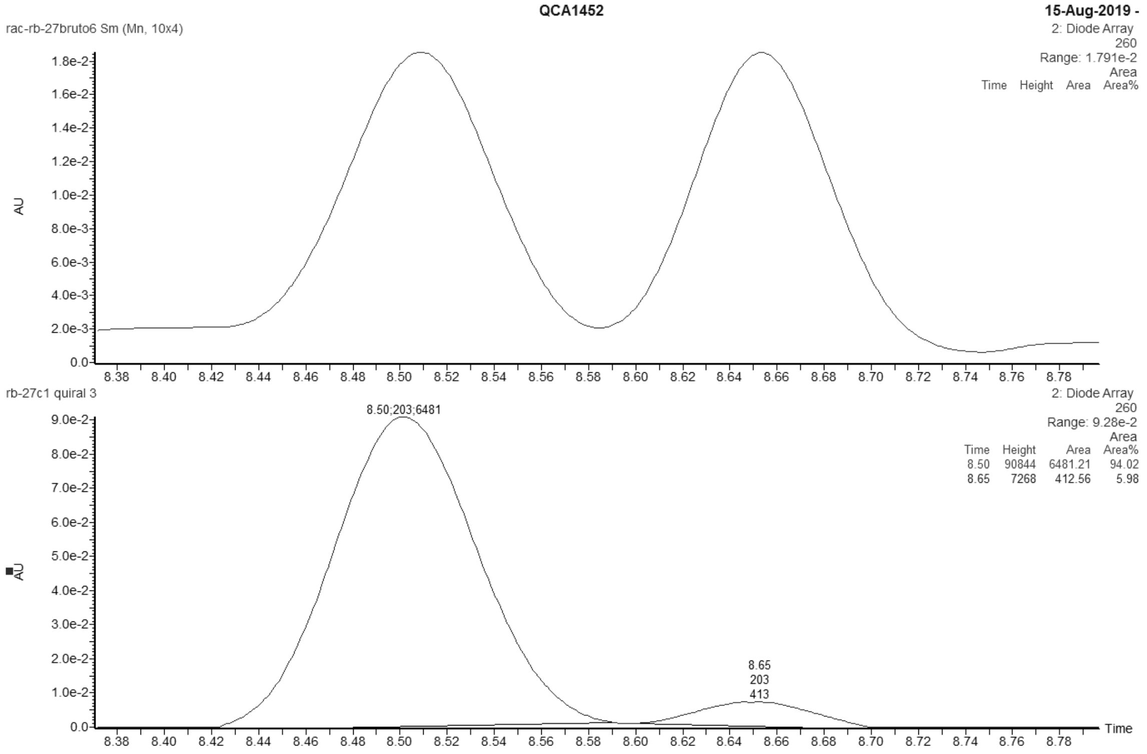

Figure S85. Chromatograms of compound **2g**.

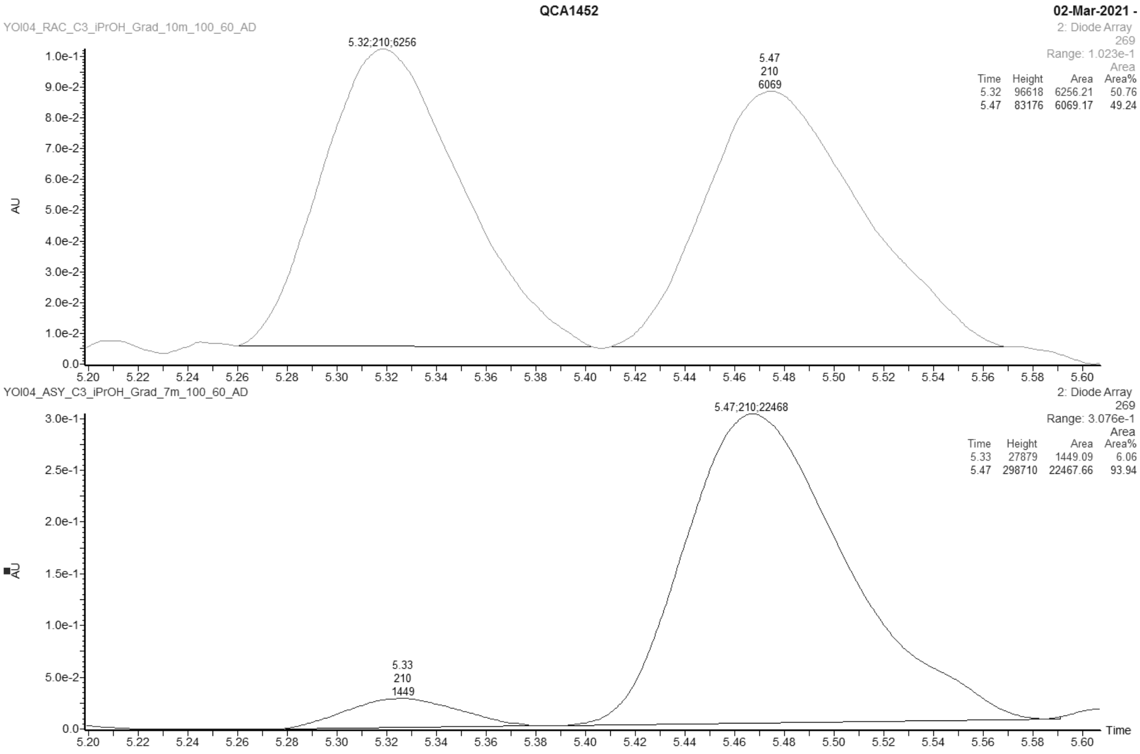

Figure S86. Chromatograms of compound **2h**.

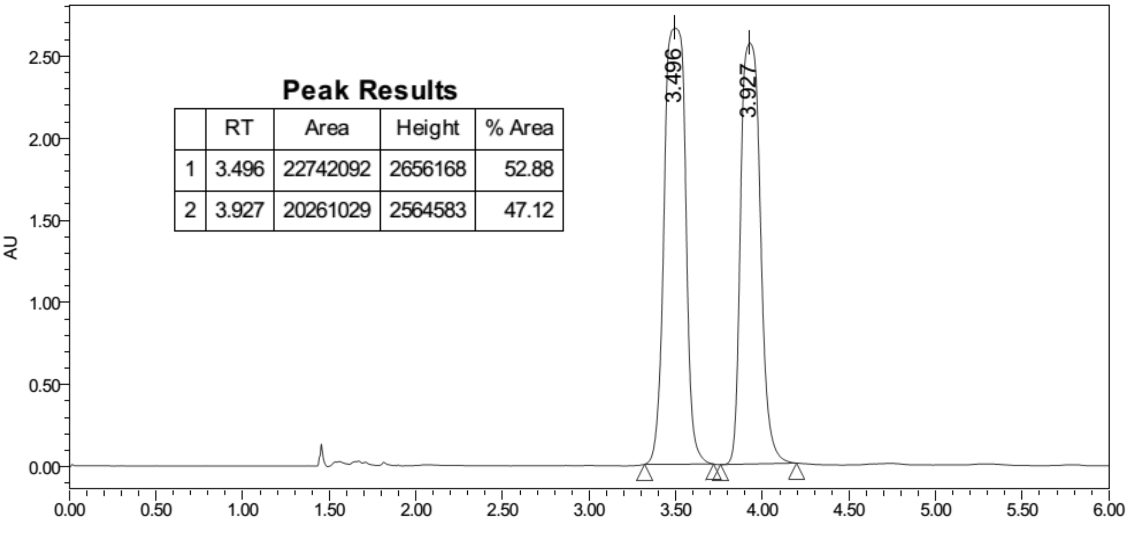

Figure S87. Chromatograms of compound **2i**.

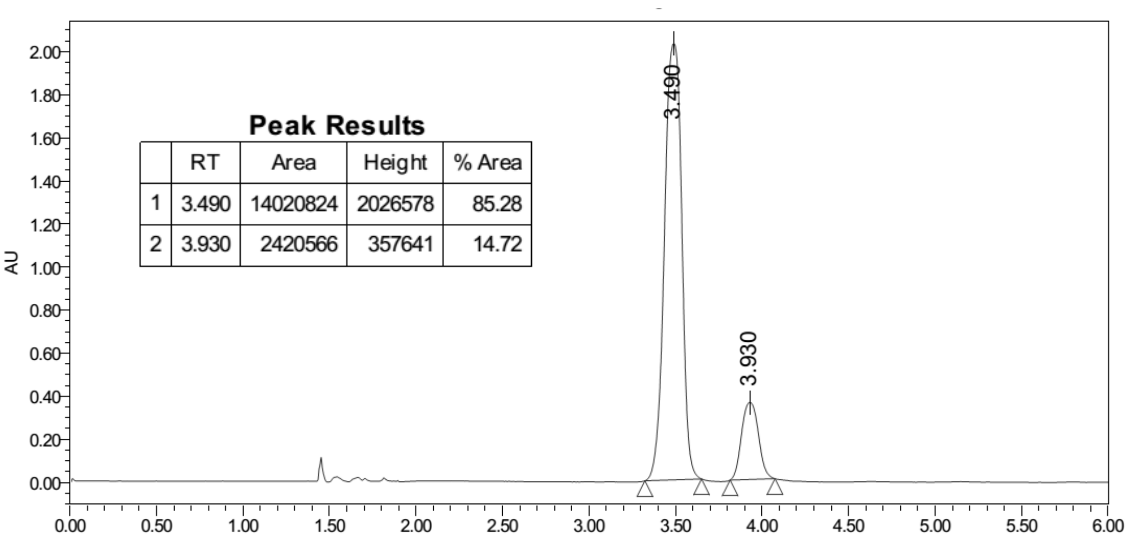

Figure S88. Chromatograms of compound **2j**.

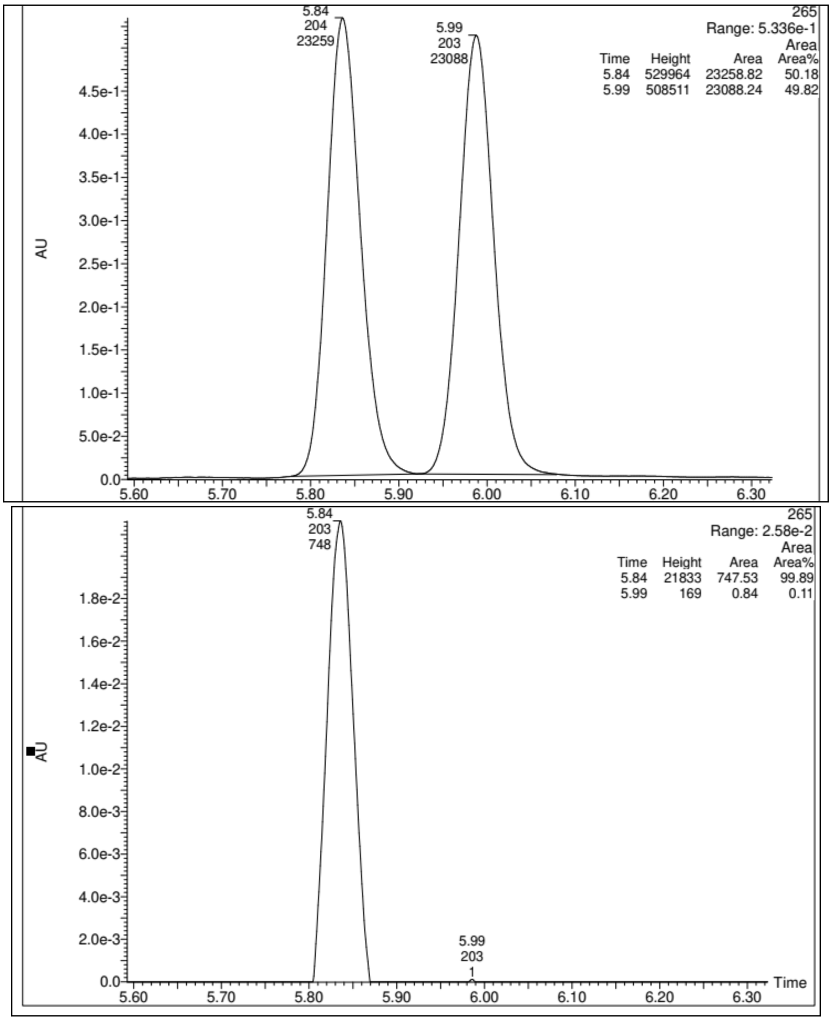

Figure S89. Chromatograms of compound **2k**.

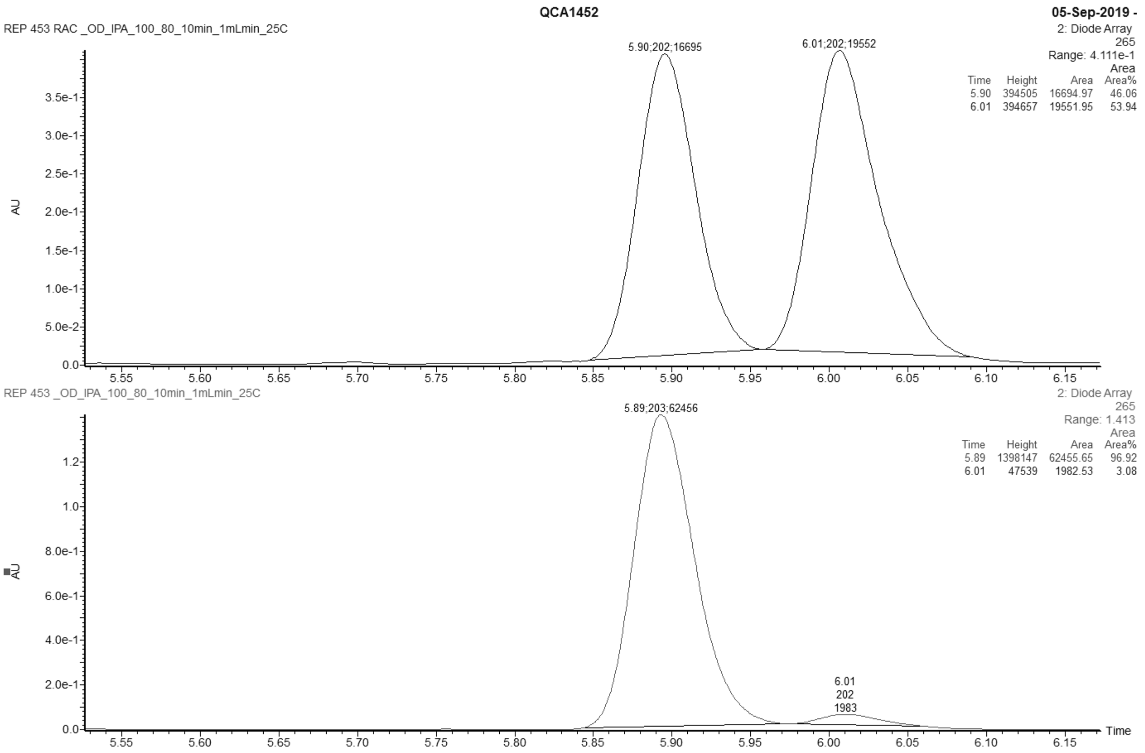

Figure S90. Chromatograms of compound **2l**.

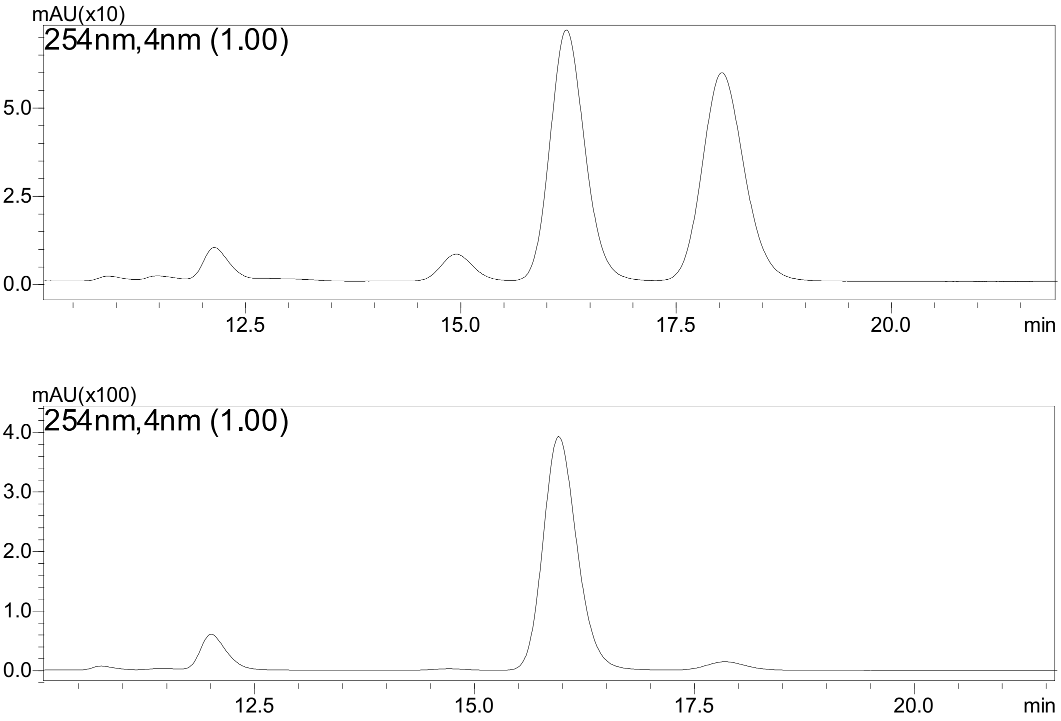

Figure S91. Chromatograms of compound **2m**.

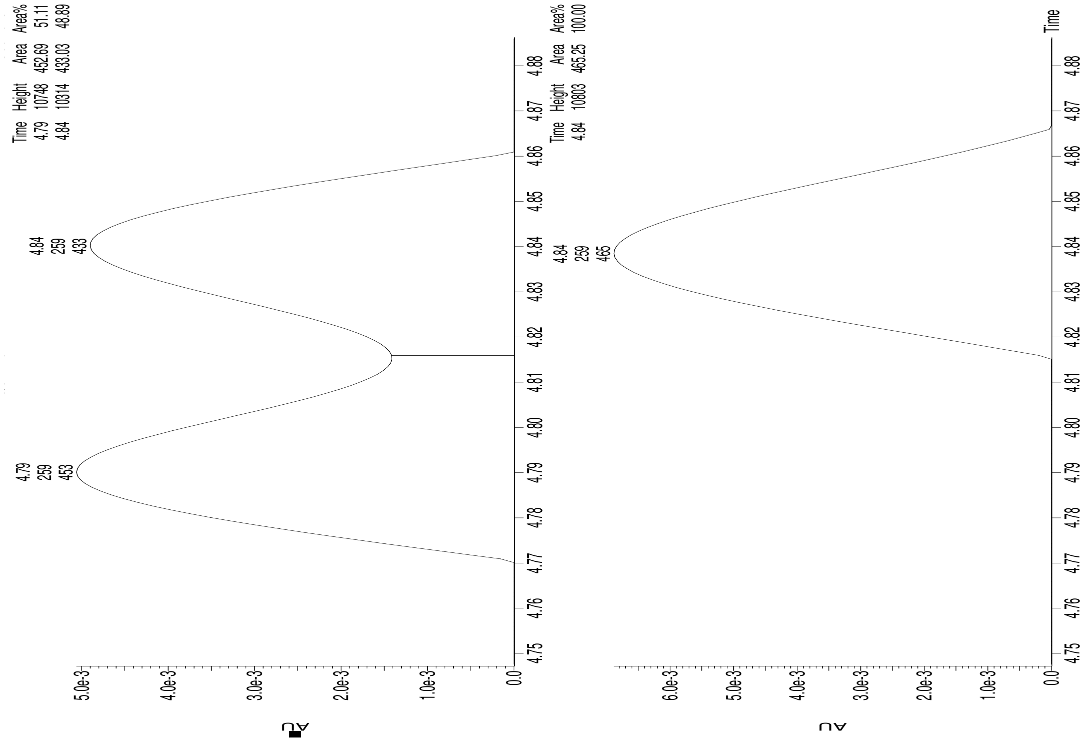

Figure S92. Chromatograms of compound **2n**.

Compound 2o

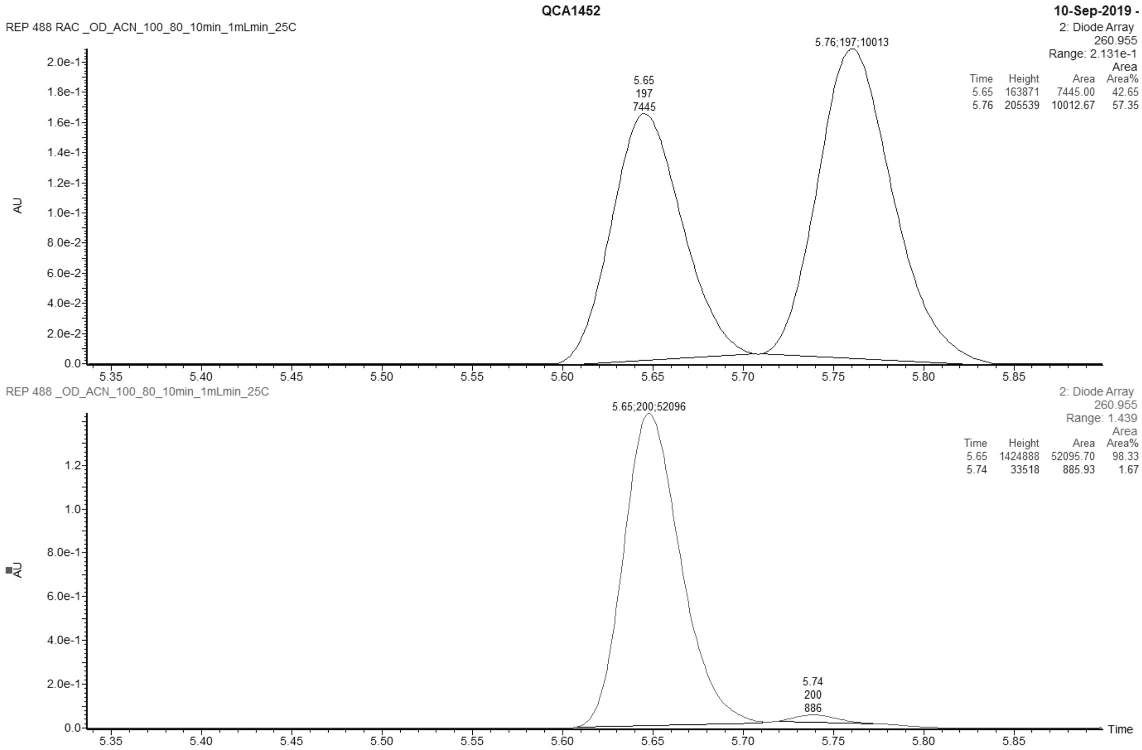

Figure S93. Chromatograms of compound **2o**.

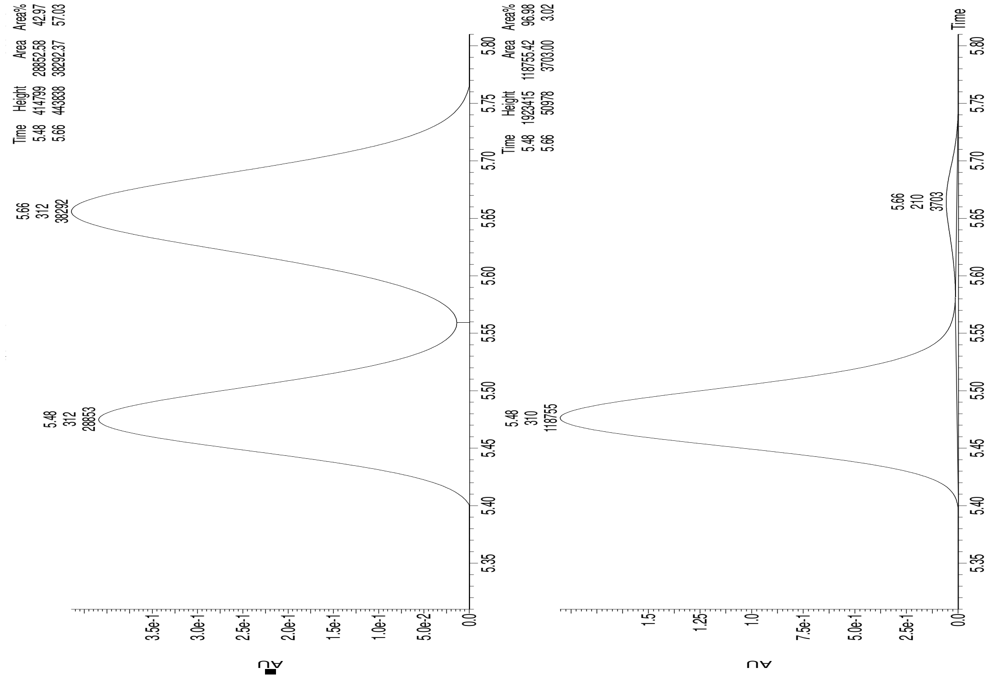

Figure S94. Chromatograms of compound **2p**.

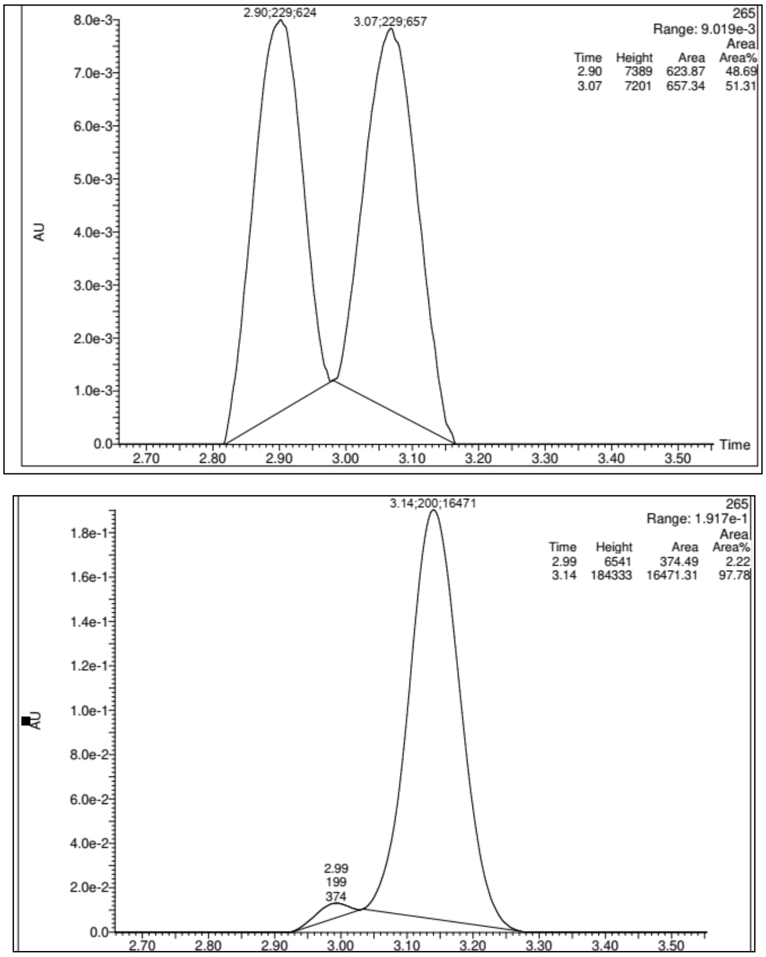

Figure S95. Chromatograms of compound **2q**.

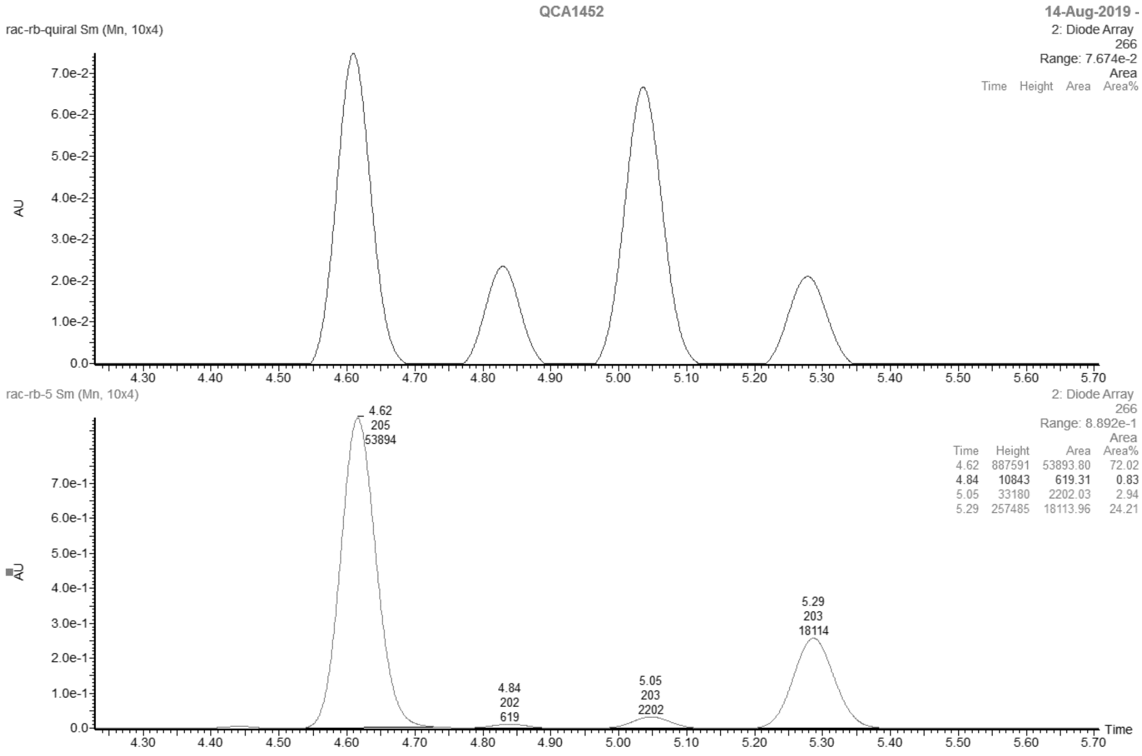

Figure S96. Chromatograms of compound **2s**.

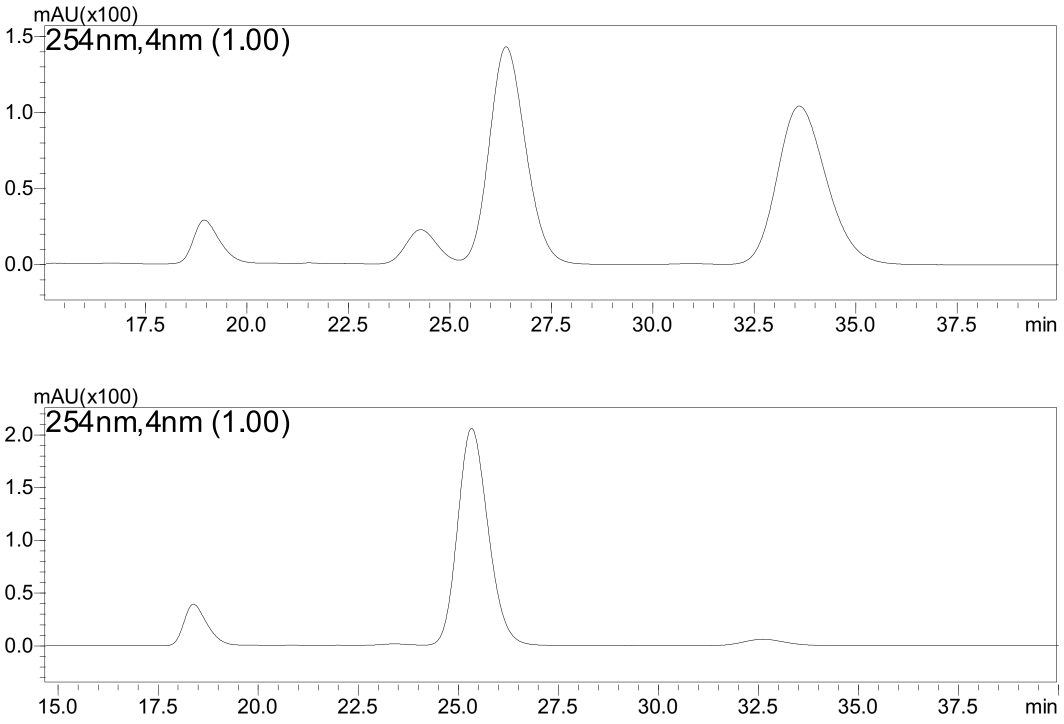

Figure S97. Chromatograms of compound **2t**.

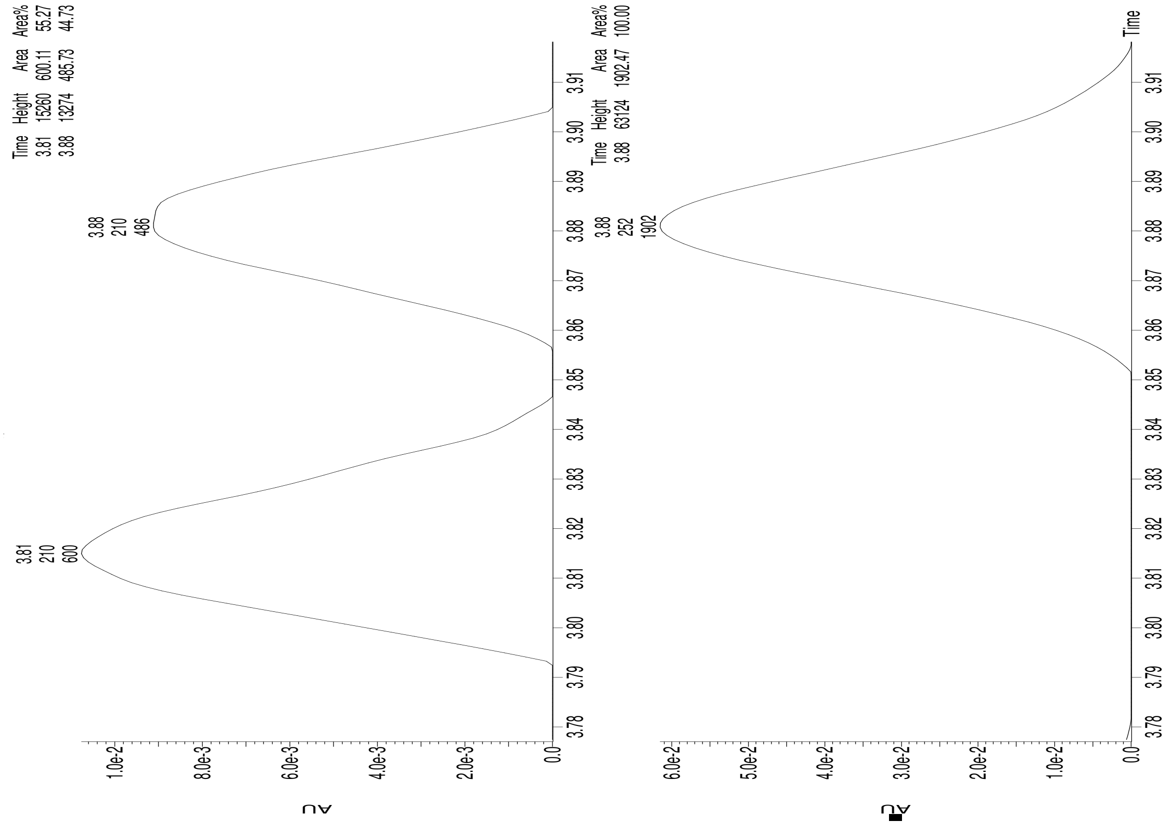

Figure S98. Chromatograms of compound **2u**.

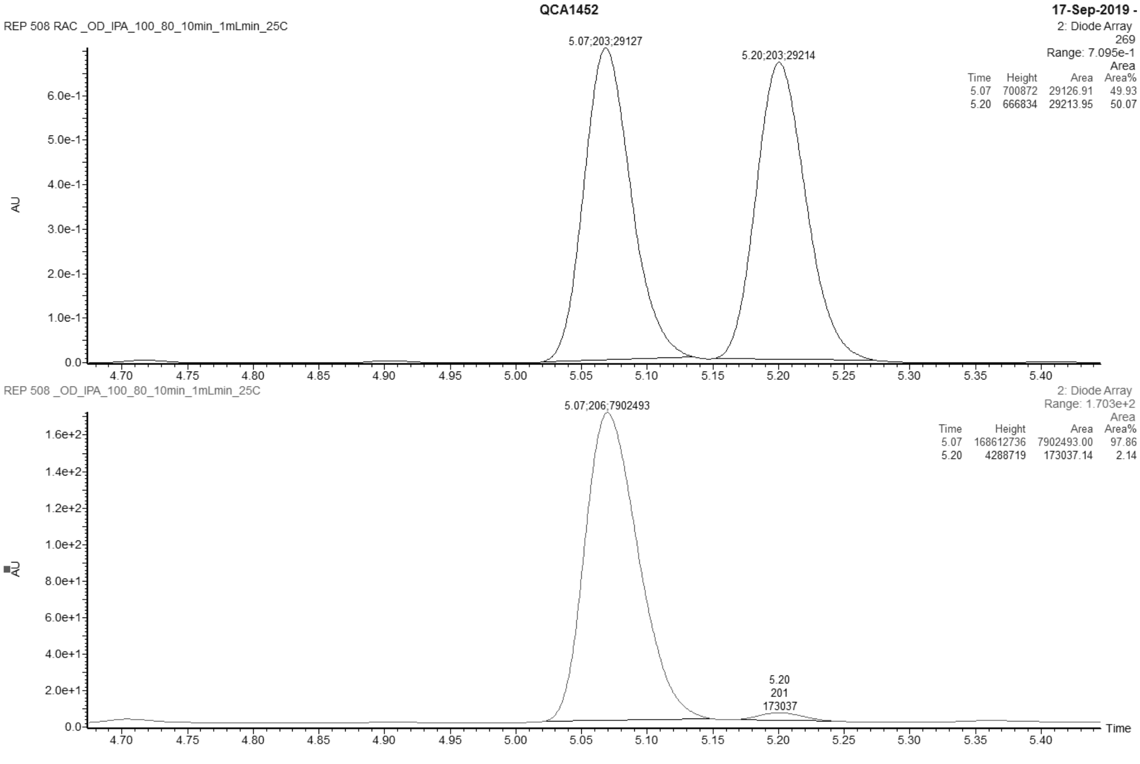

Figure S99. Chromatograms of compound **2w**.

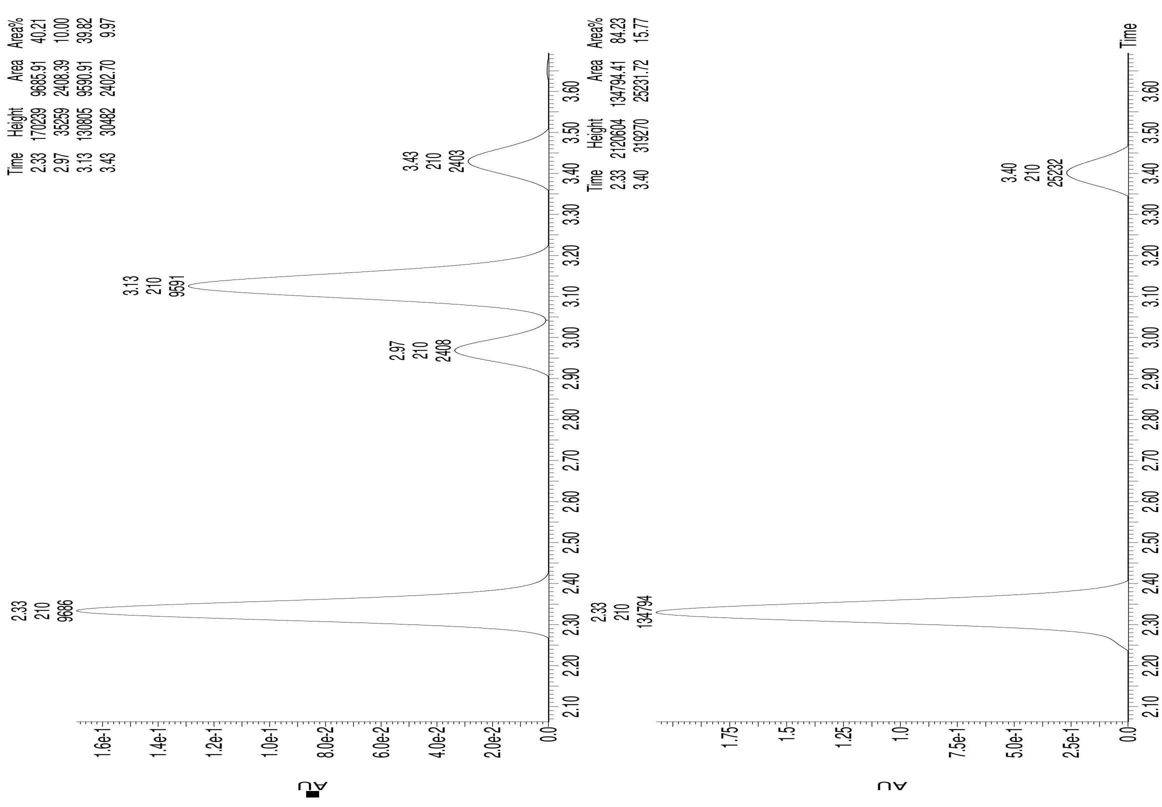

Figure S100. Chromatograms of compound **2x**.

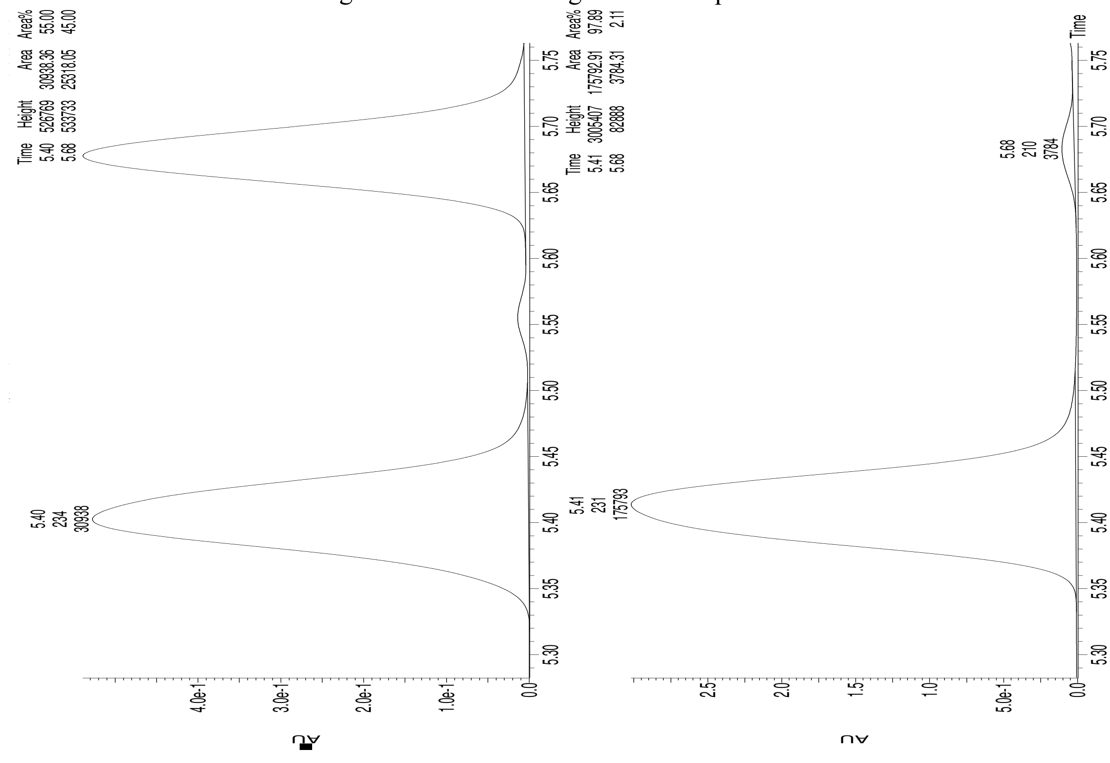

Figure S101. Chromatograms of compound **2y**.

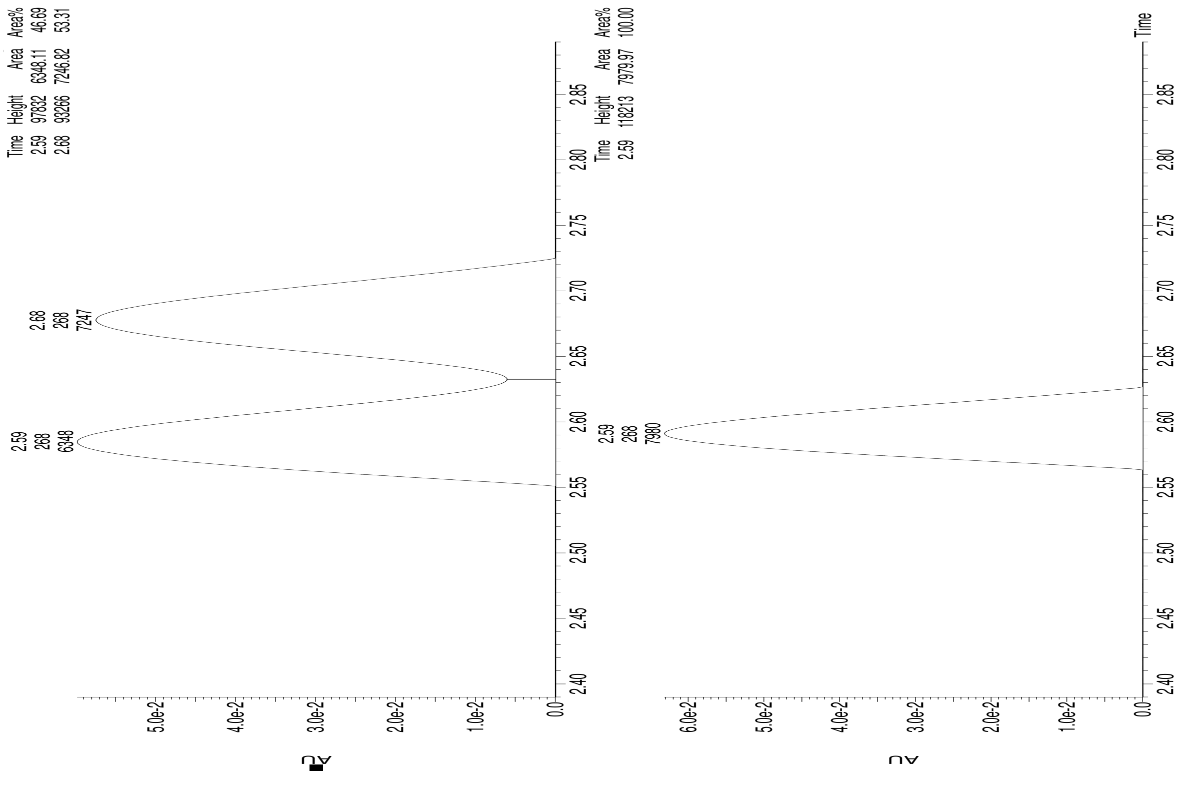

Figure S102. Chromatograms of compound **2z**.

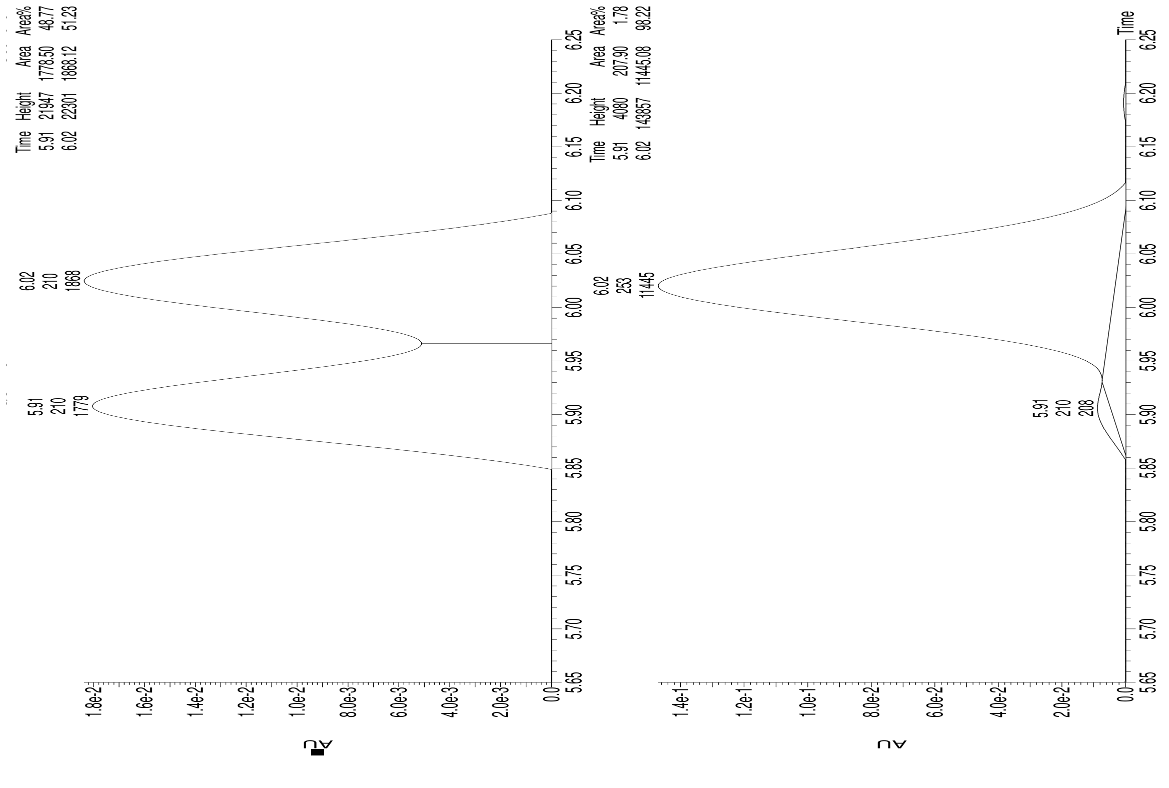

Figure S103. Chromatograms of compound **2aa**.

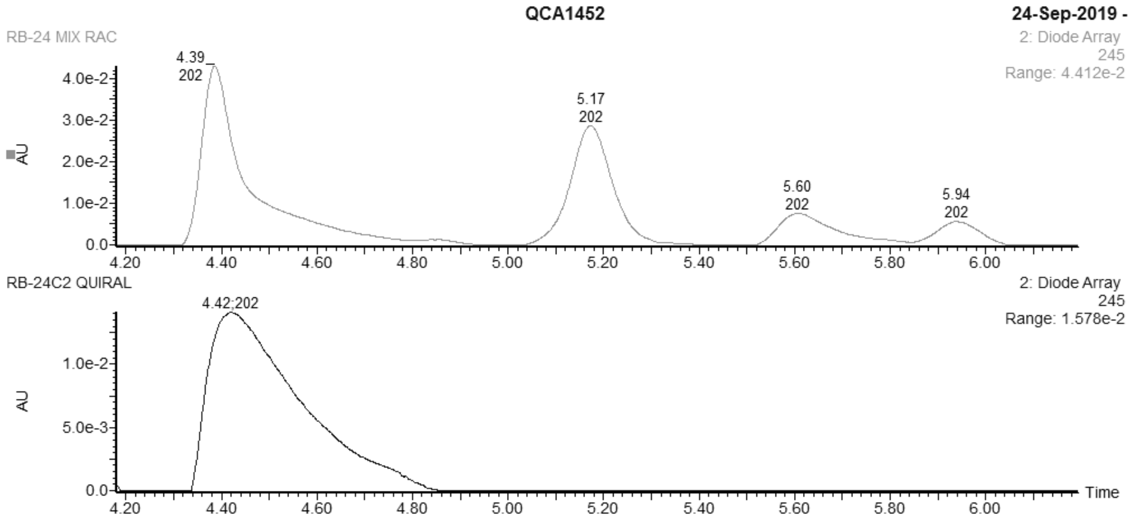

Figure S104. Chromatograms of compound **2ab**.

Compound 2ac

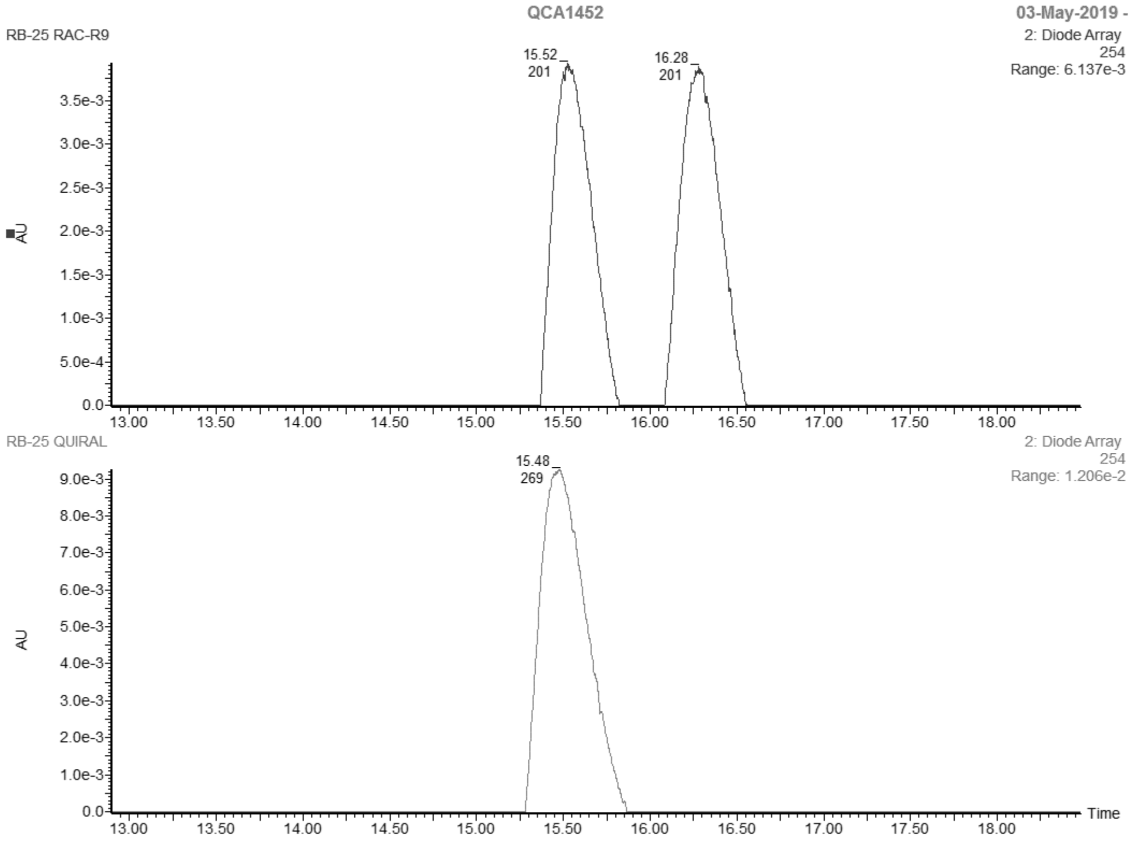

Figure S105. Chromatograms of compound **2ac**.

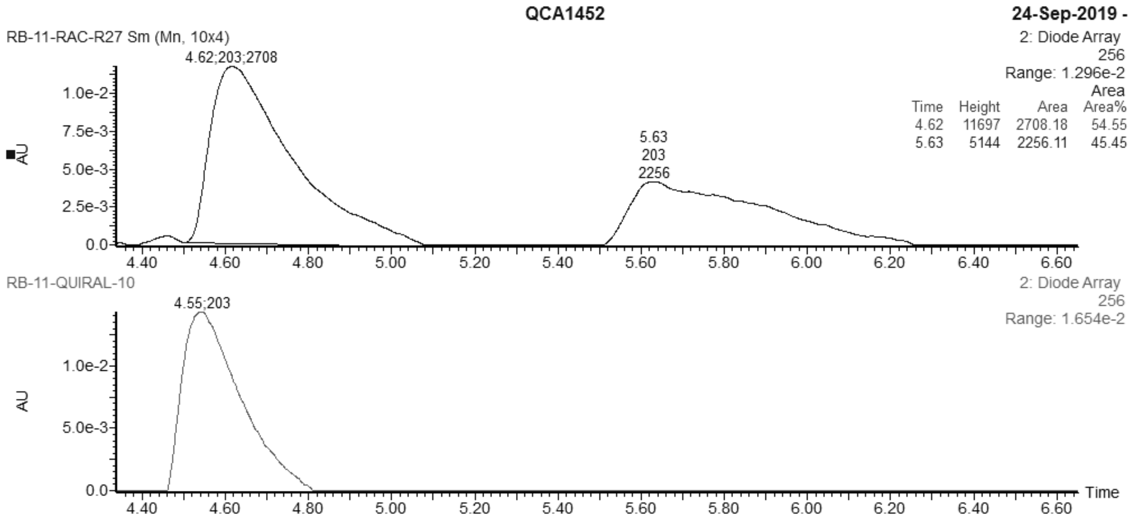

Figure S106. Chromatograms of compound **2ad**.

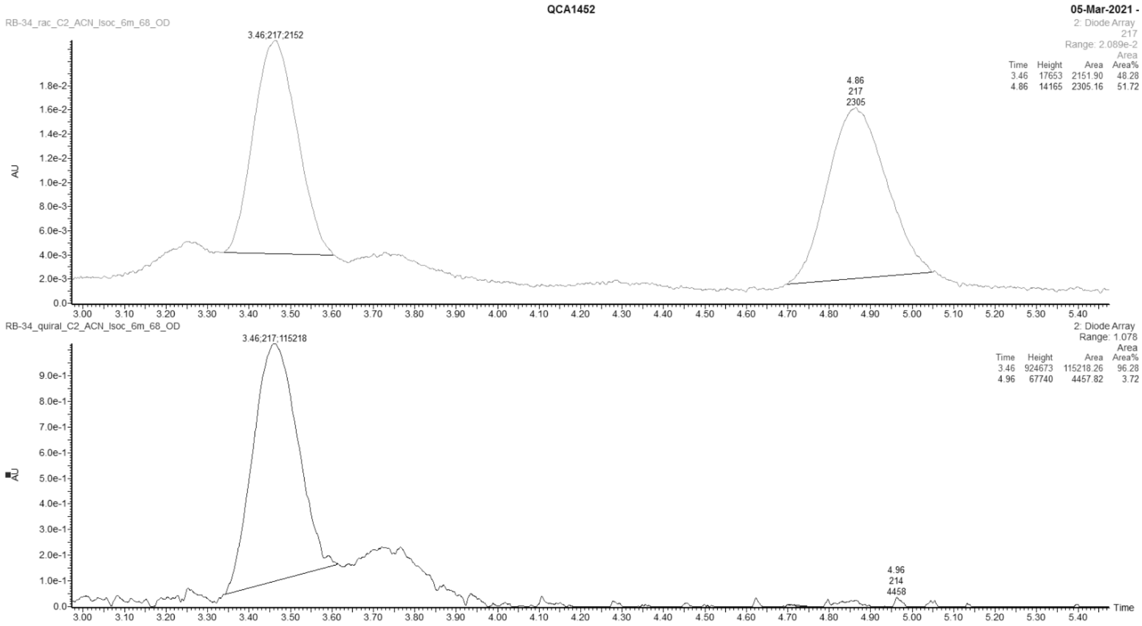

Figure S107. Chromatograms of compound **2ae**.

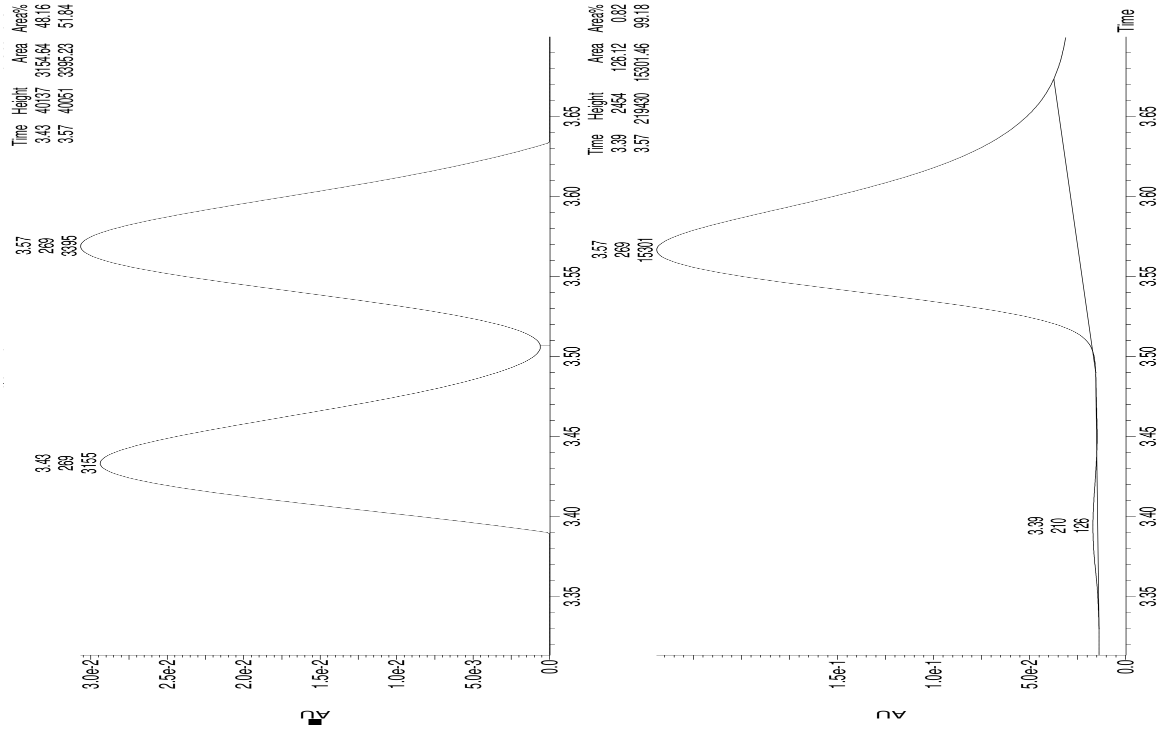

Figure S108. Chromatograms of compound **2af**.

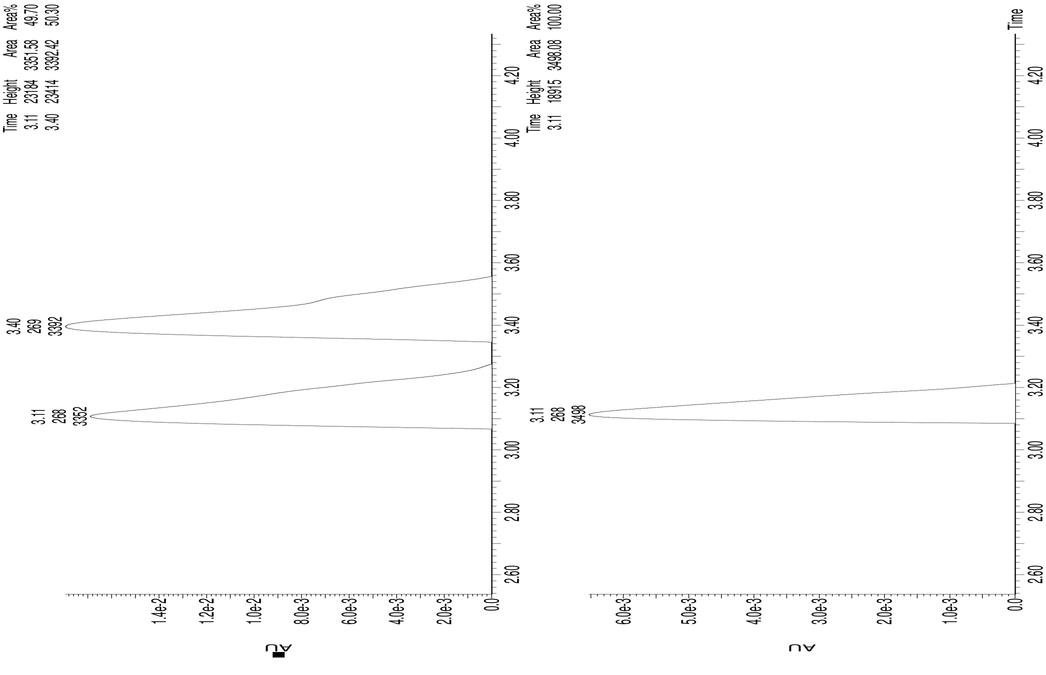

Figure S109. Chromatograms of compound **2ag**.

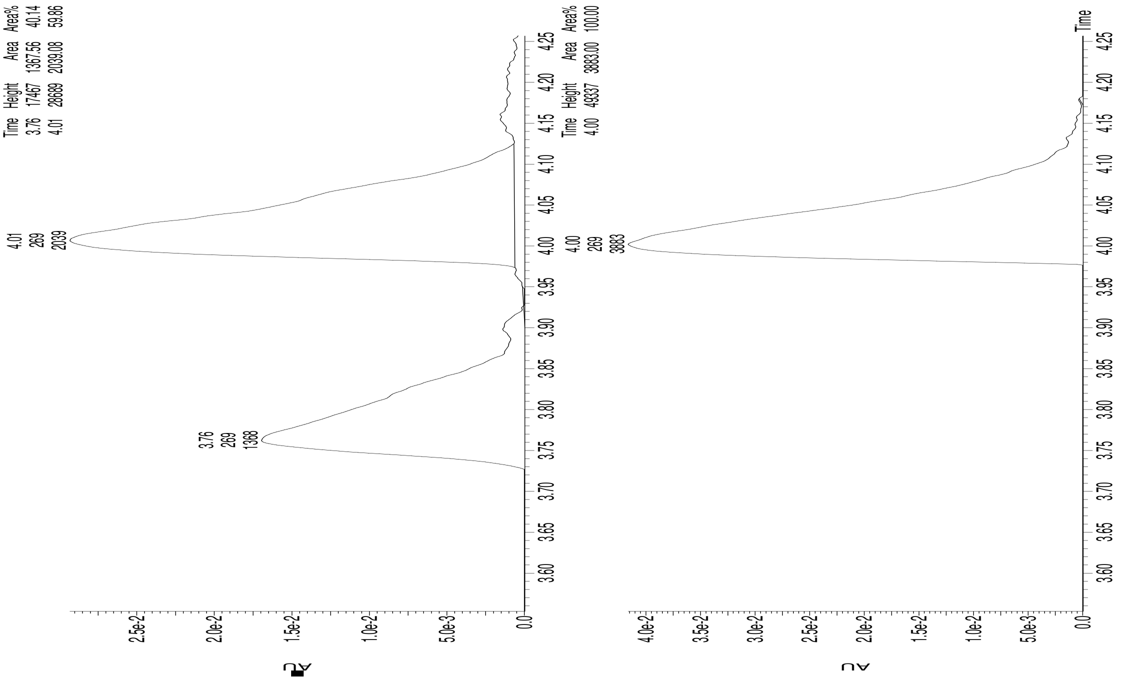

Figure S110. Chromatograms of compound **2ah**.

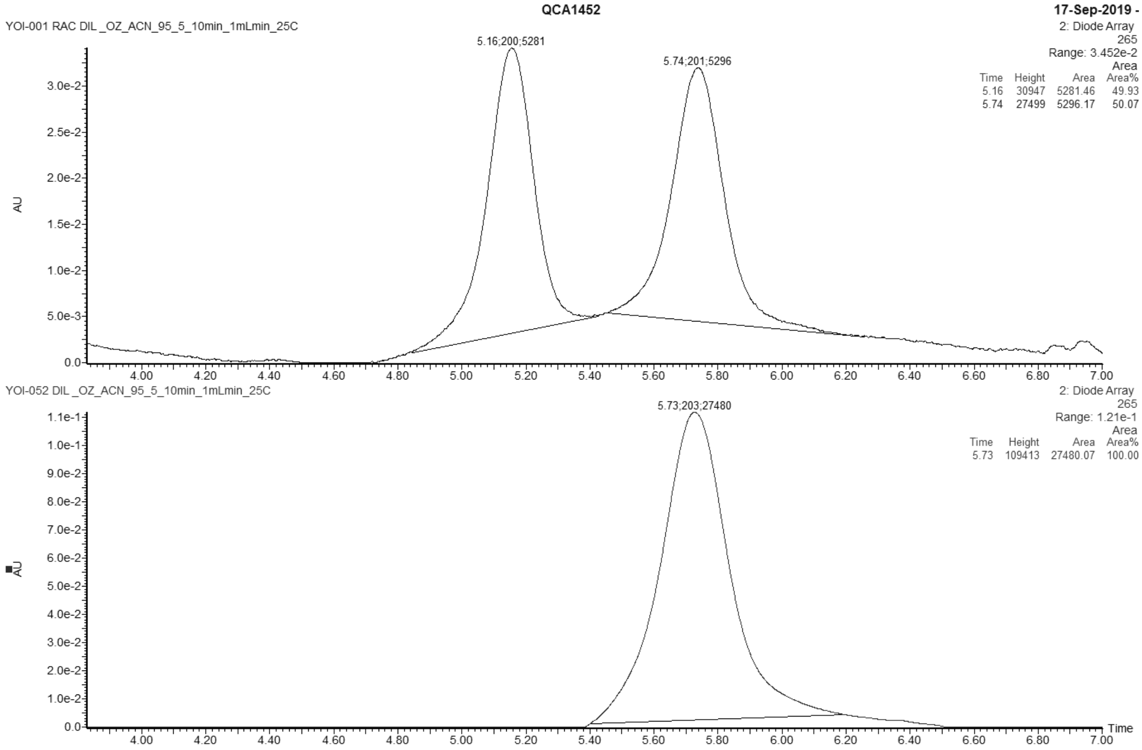

Figure S111. Chromatograms of compound **2ai**.

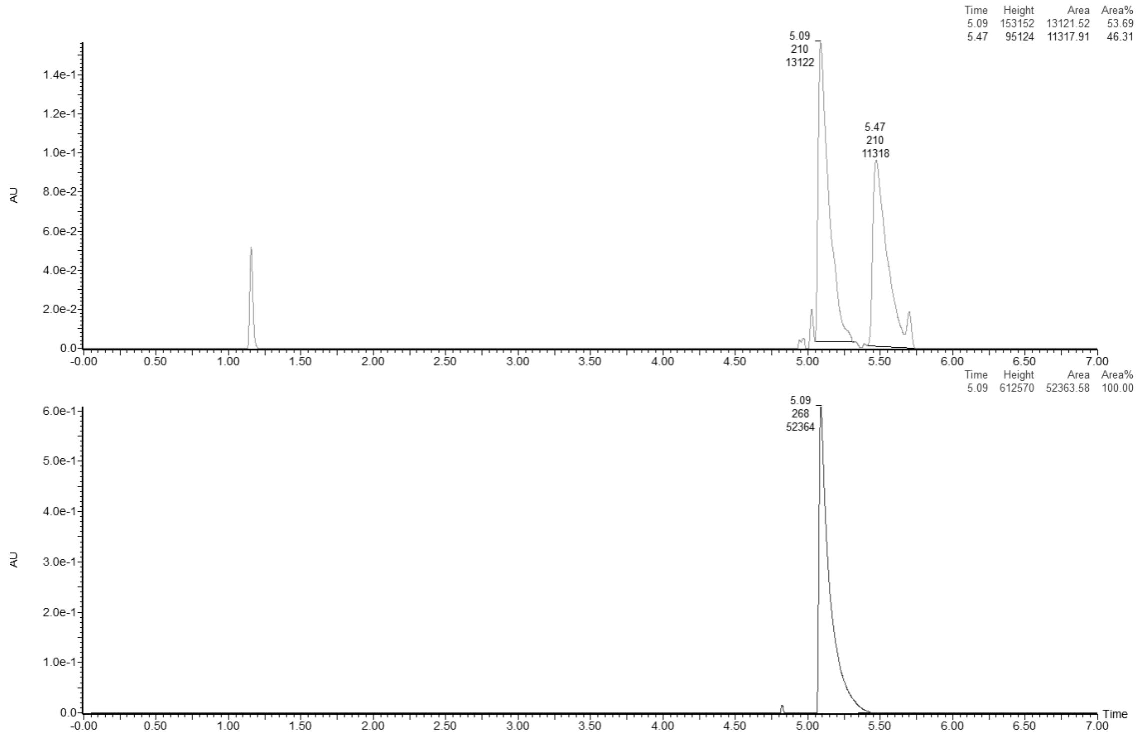

Figure S112. Chromatograms of compound **2aj**.

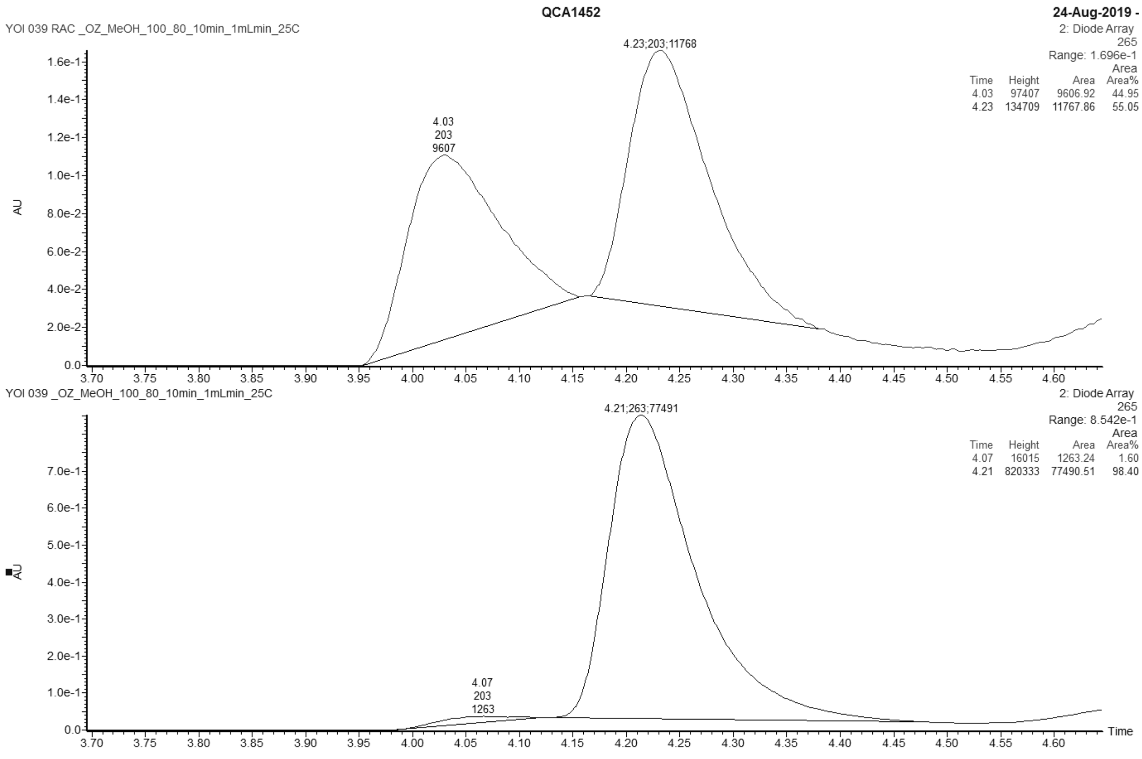

Figure S113. Chromatograms of compound **2ak**.

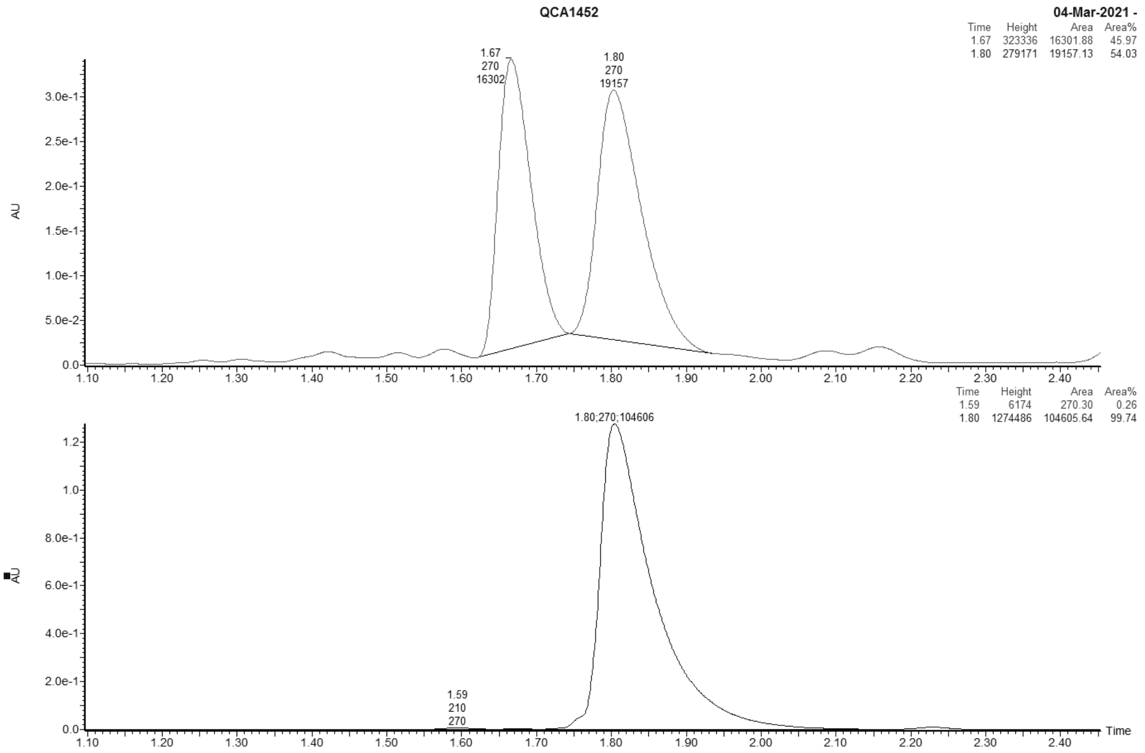

Figure S114. Chromatograms of compound **2al**.

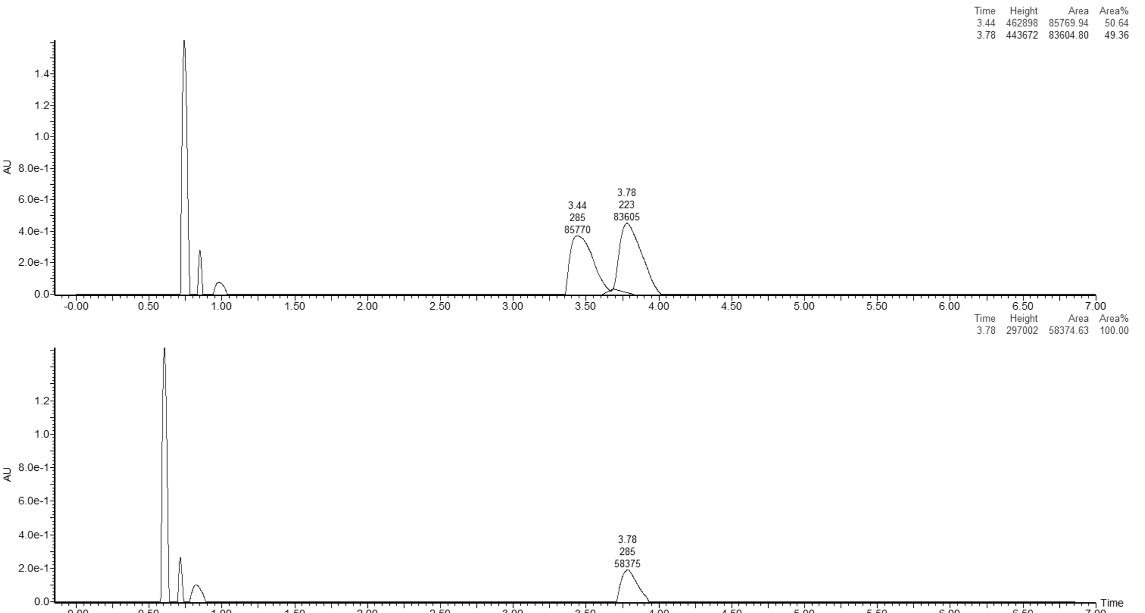

Figure S115. Chromatograms of compound **2am**.

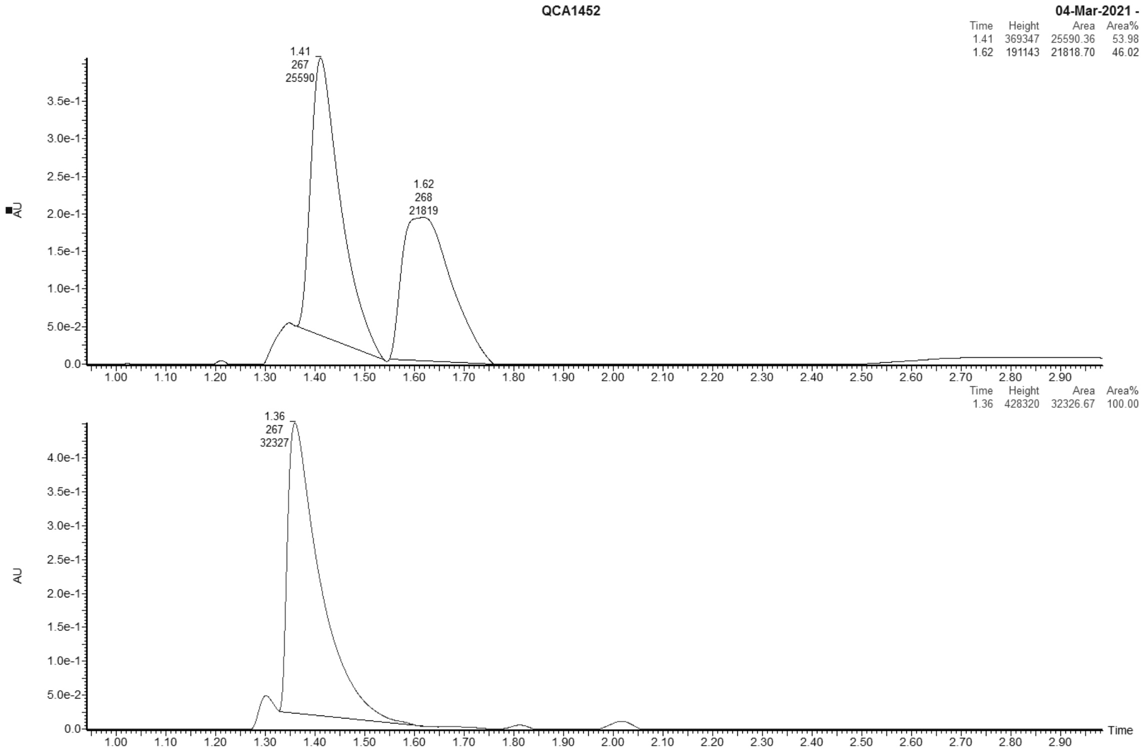

Figure S116. Chromatograms of compound **2an**.

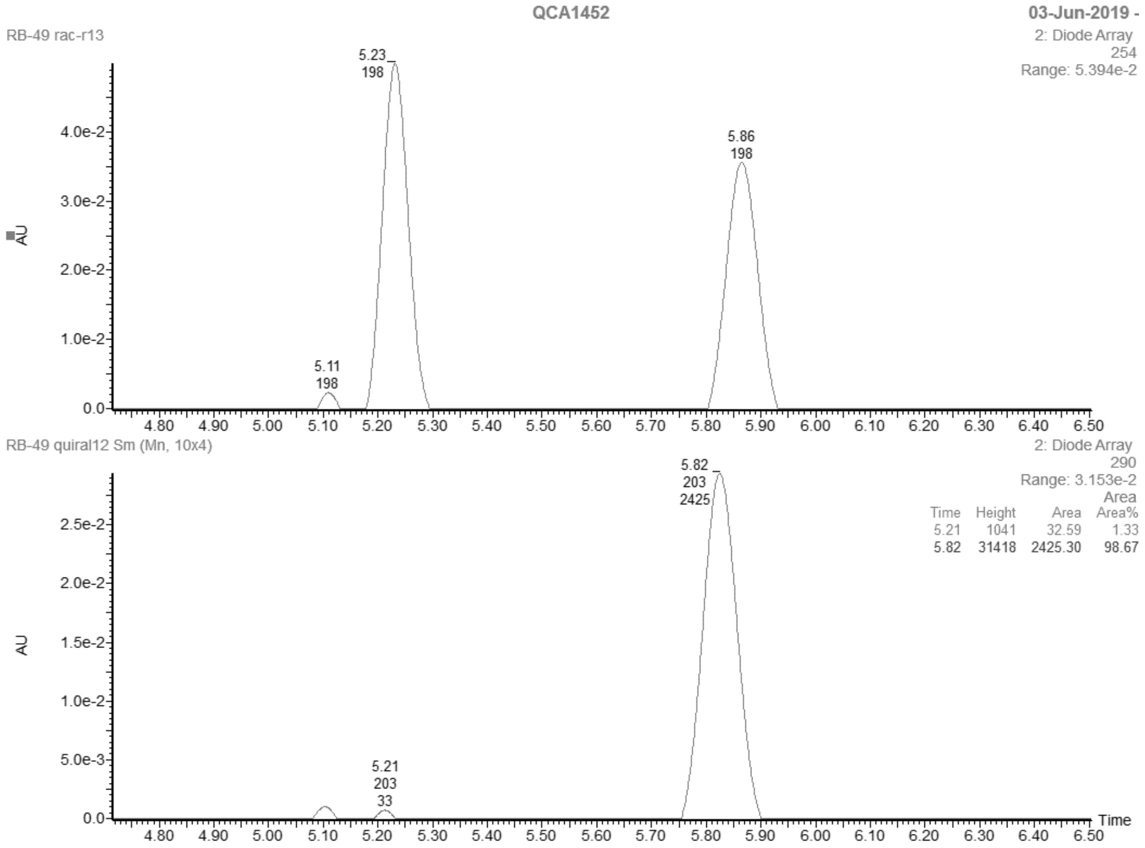

Figure S117. Chromatograms of compound **3a**.

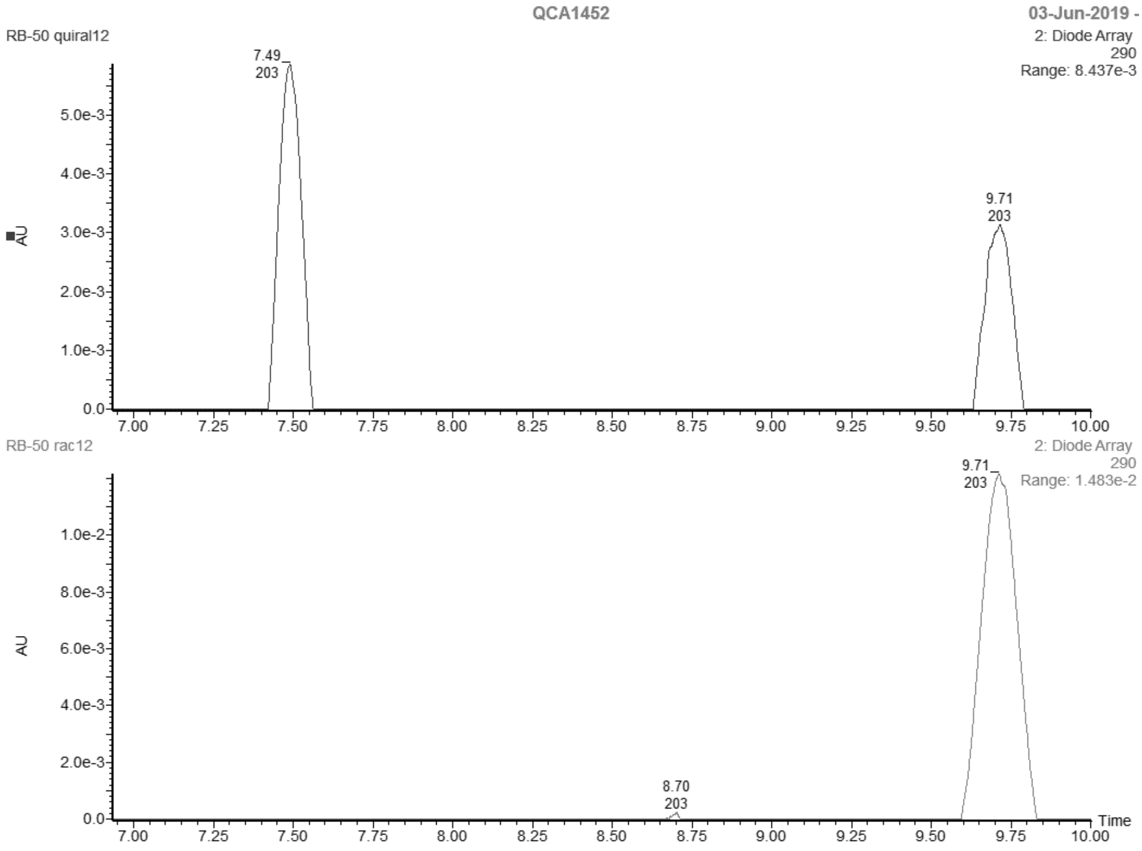

Figure S118. Chromatograms of compound **3b**.

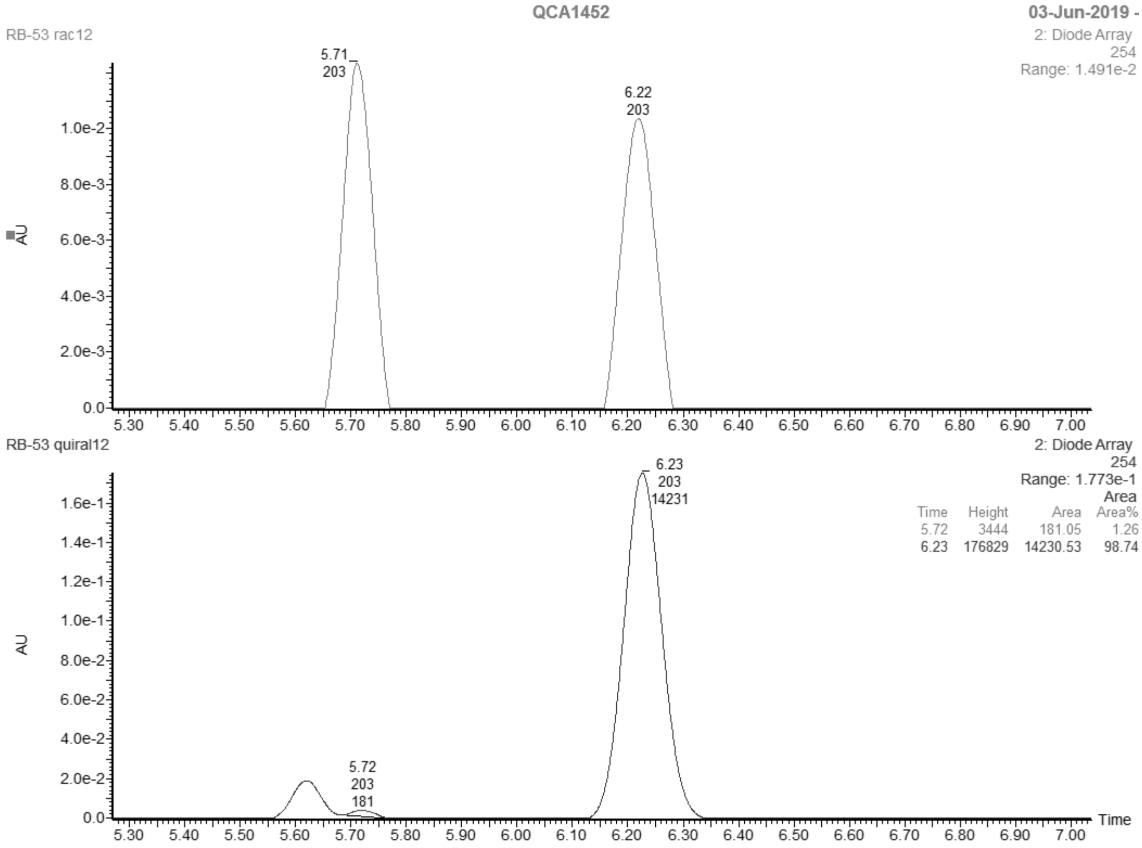

Figure S119. Chromatograms of compound **3c**.

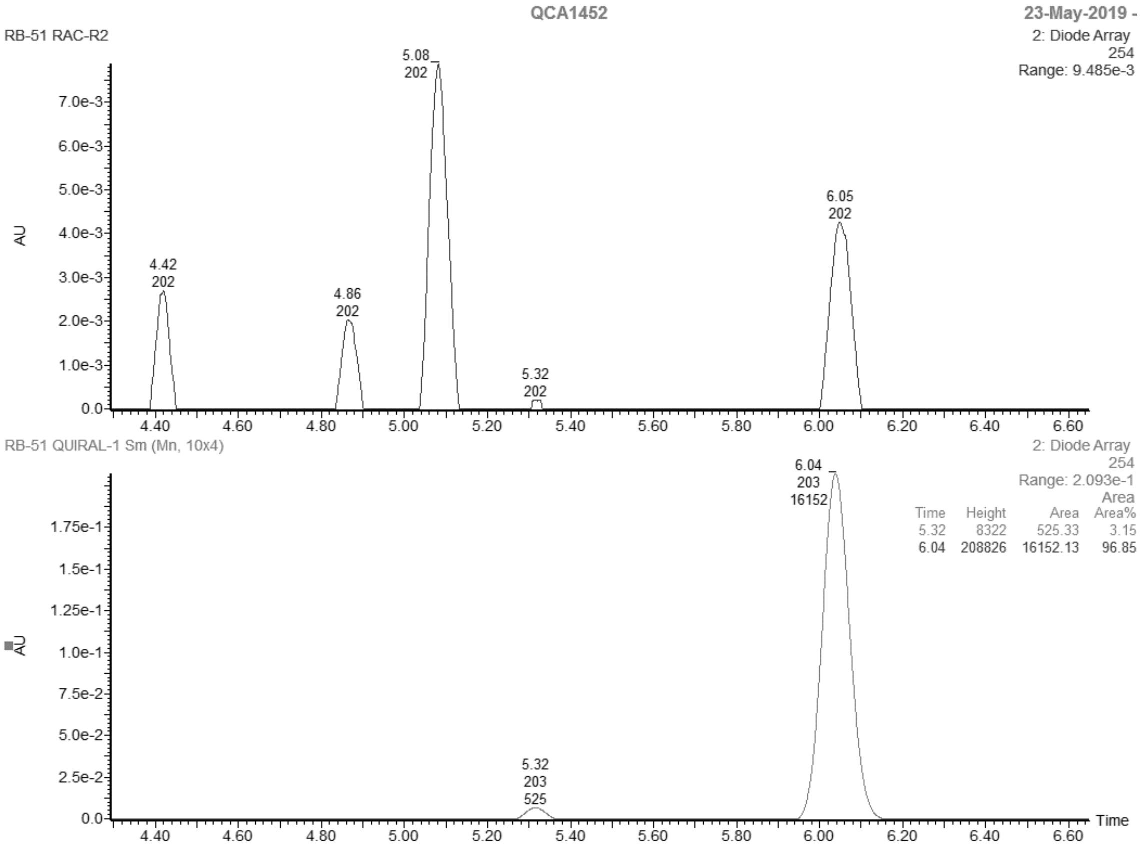

Figure S120. Chromatograms of compound **3d**.

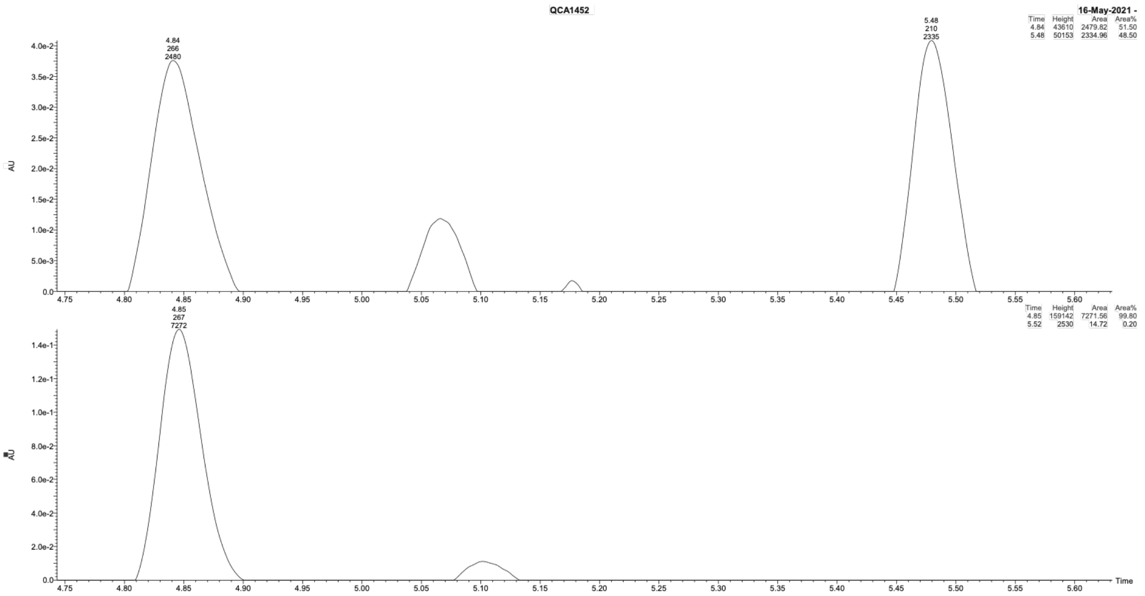

Figure S121. Chromatograms of compound **4**.
