# Supplementary material for: Direct access to tetrasubstituted cyclopentenyl scaffolds through a diastereoselective isocyanide-based multicomponent reaction
Source: Chem Sci. 2021 Sep 16;12(48):15862–9. doi: 10.1039/d1sc04158d (PMC8672720; doi:10.1039/d1sc04158d)
Supplement: SC-012-D1SC04158D-s003 [file SC-012-D1SC04158D-s003.pdf]

Electronic Supplementary Material (ESI) for Chemical Science.
This journal is © The Royal Society of Chemistry 2021

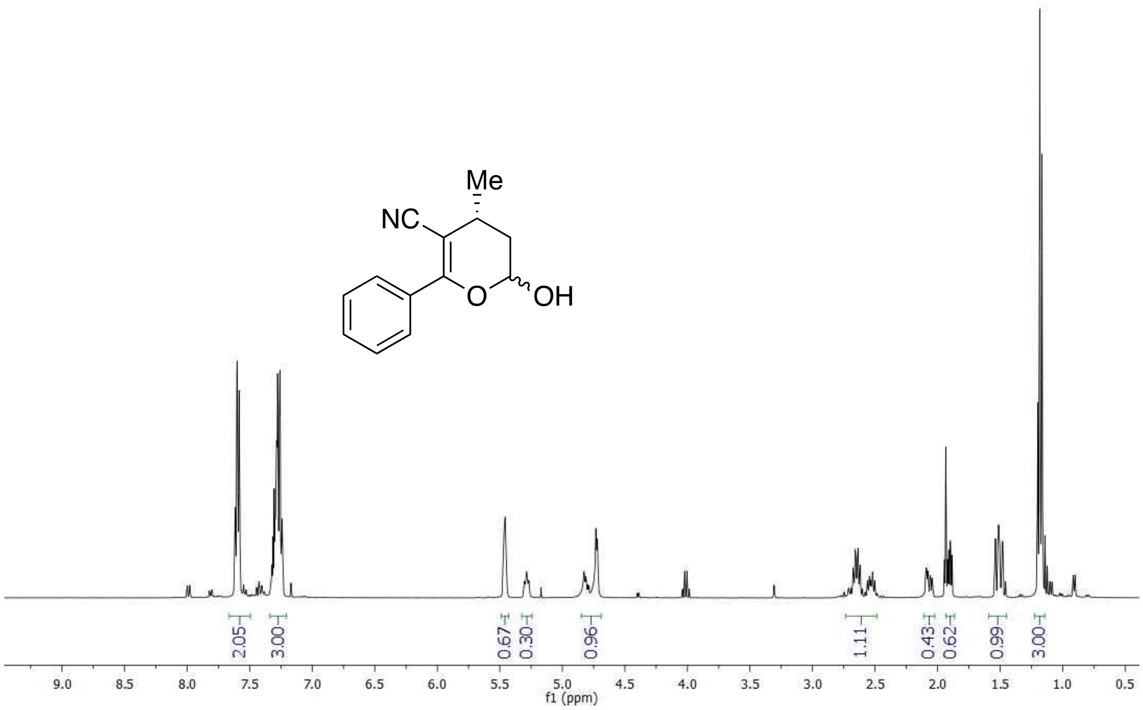

Figure 1. First submission ^{1}H NMR spectrum in $CDCl_3$ of compound **1b**.

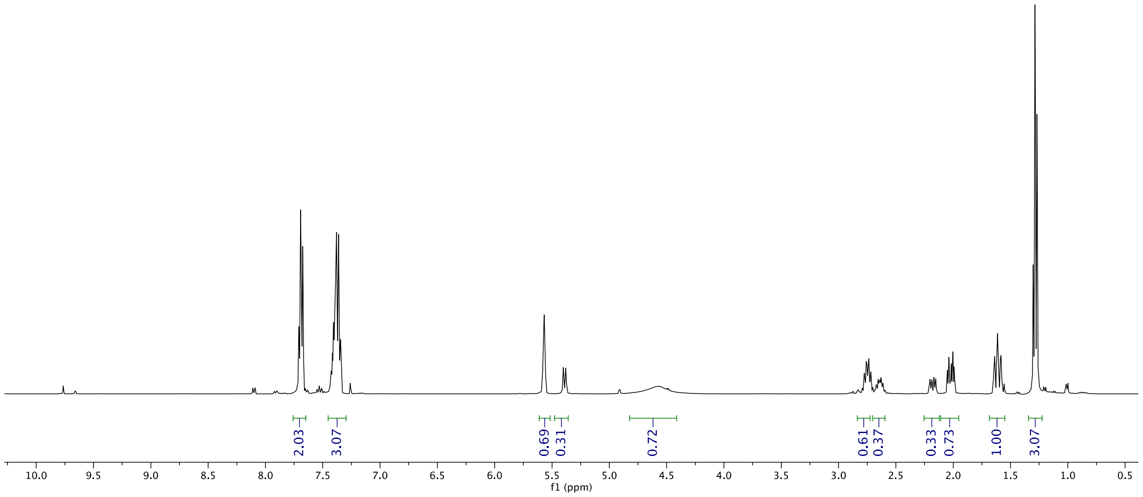

Figure 2. New submission ^{1}H NMR spectrum in $CDCl_3$ of compound **1b**.

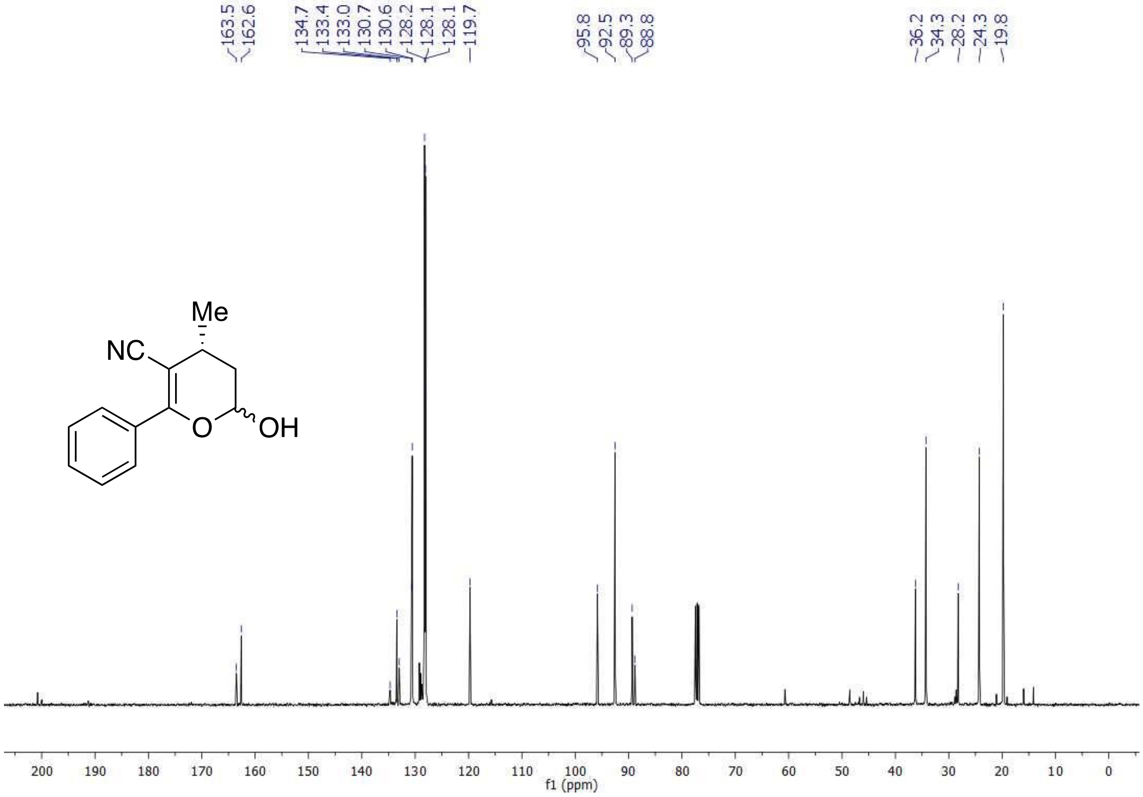

Figure 3. First submission ^{13}C NMR spectrum in $CDCl_3$ of compound **1b**.

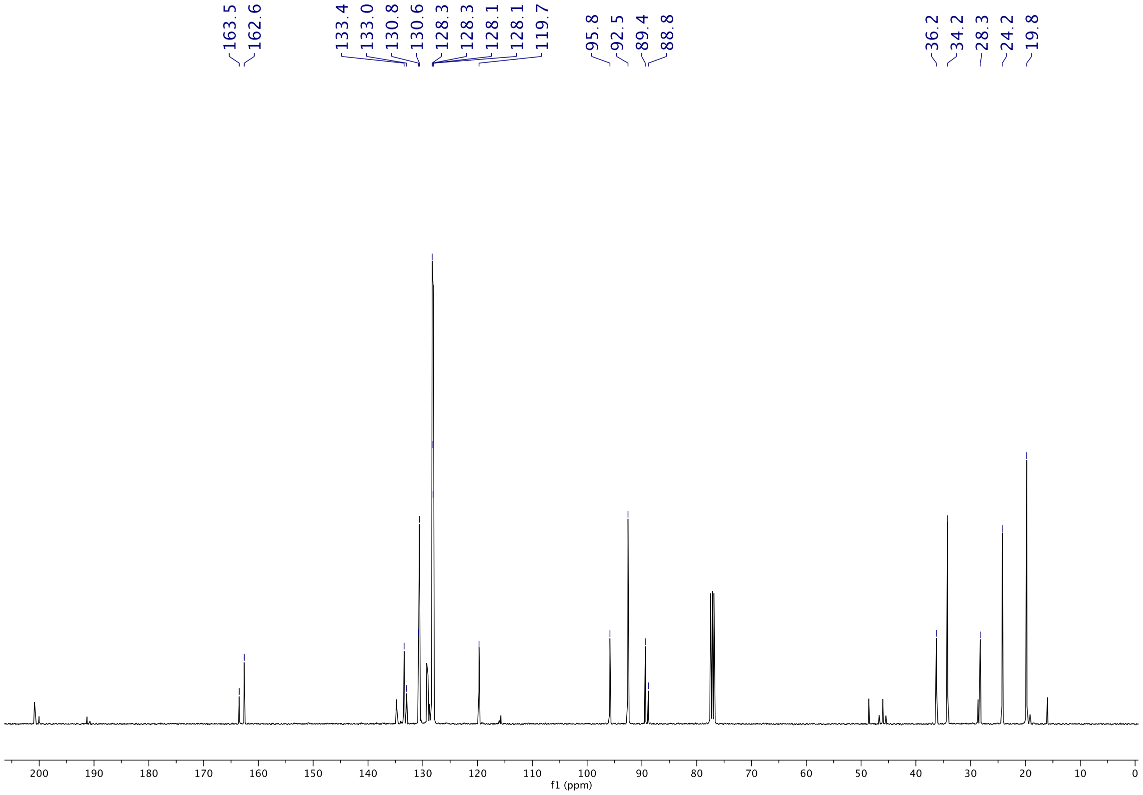

Figure 4. New submission ^{13}C NMR spectrum in $CDCl_3$ of compound **1b**.

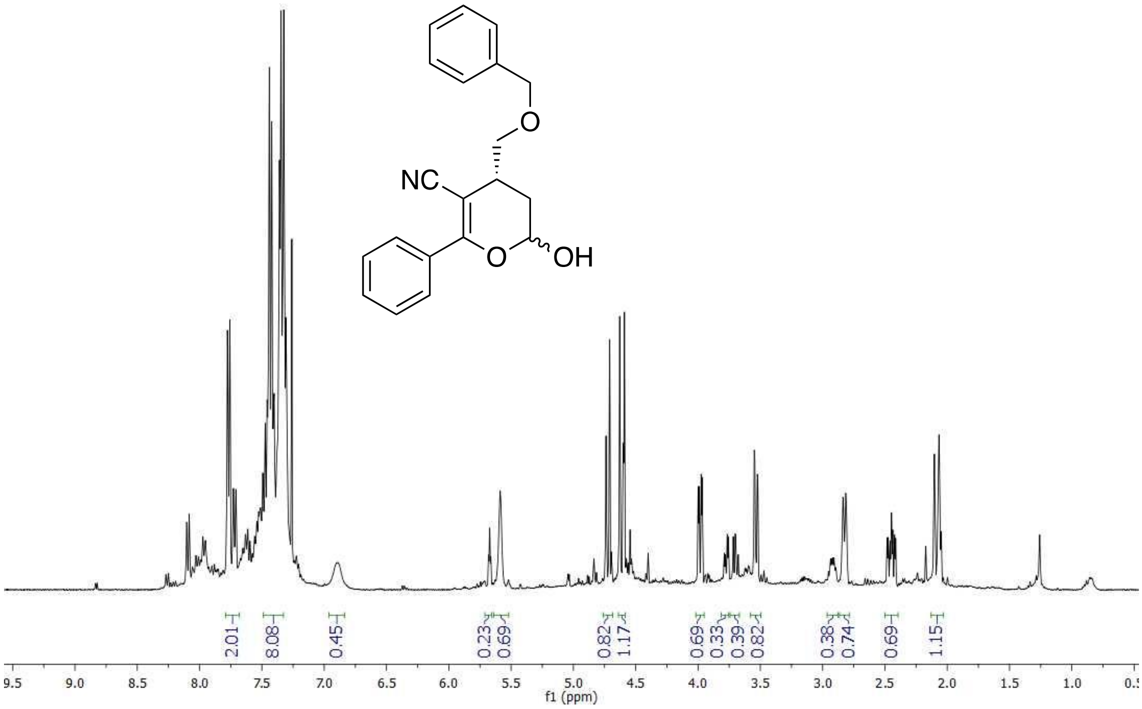

Figure 5. First submission ^{1}H NMR spectrum in $CDCl_3$ of compound **1f**.

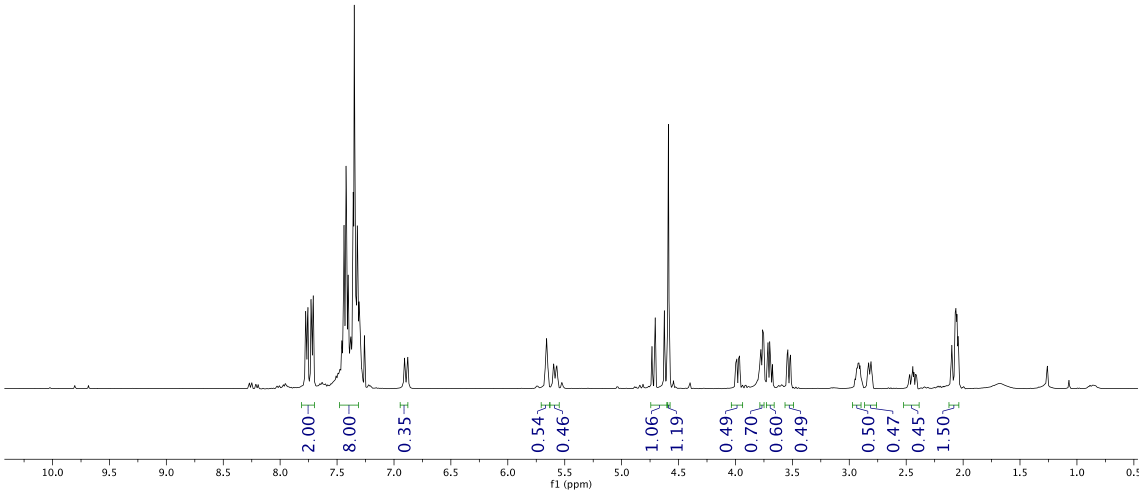

Figure 6. New submission ^{1}H NMR spectrum in $CDCl_3$ of compound **1f**.

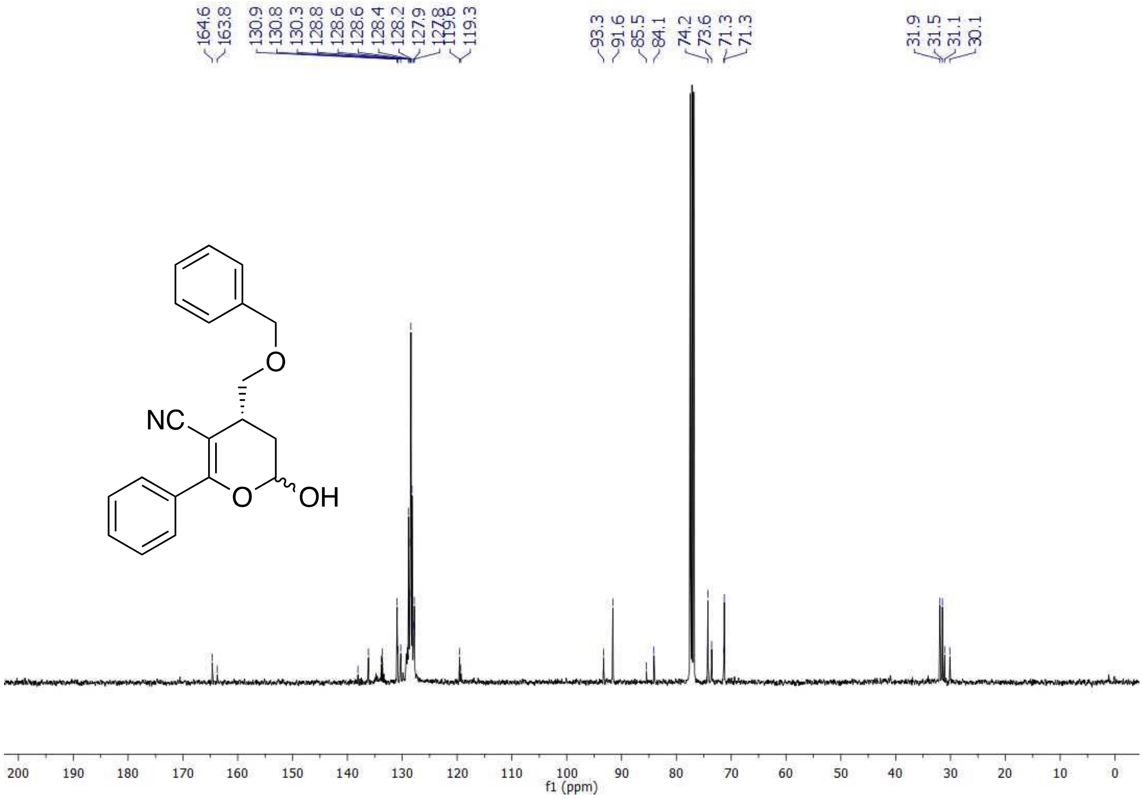

Figure 7. First submission ^{13}C NMR spectrum in $CDCl_3$ of compound **1f**.

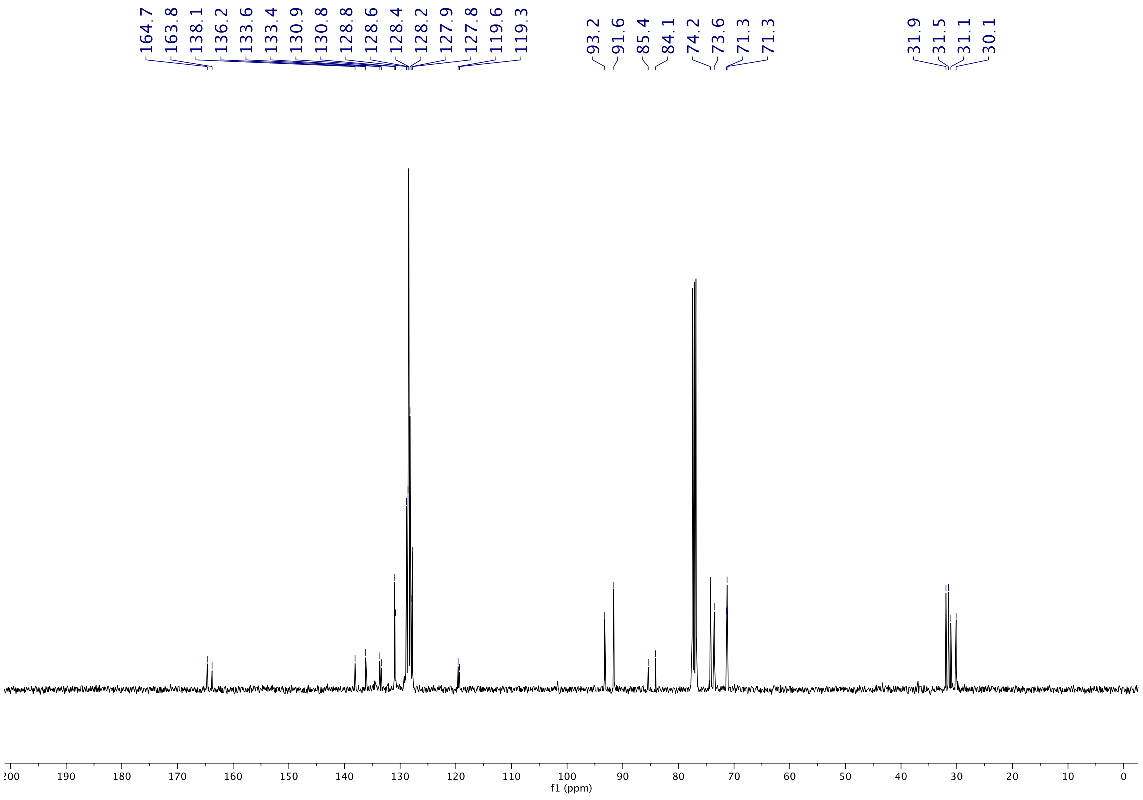

Figure 8. New submission ^{13}C NMR spectrum in $CDCl_3$ of compound **1f**.

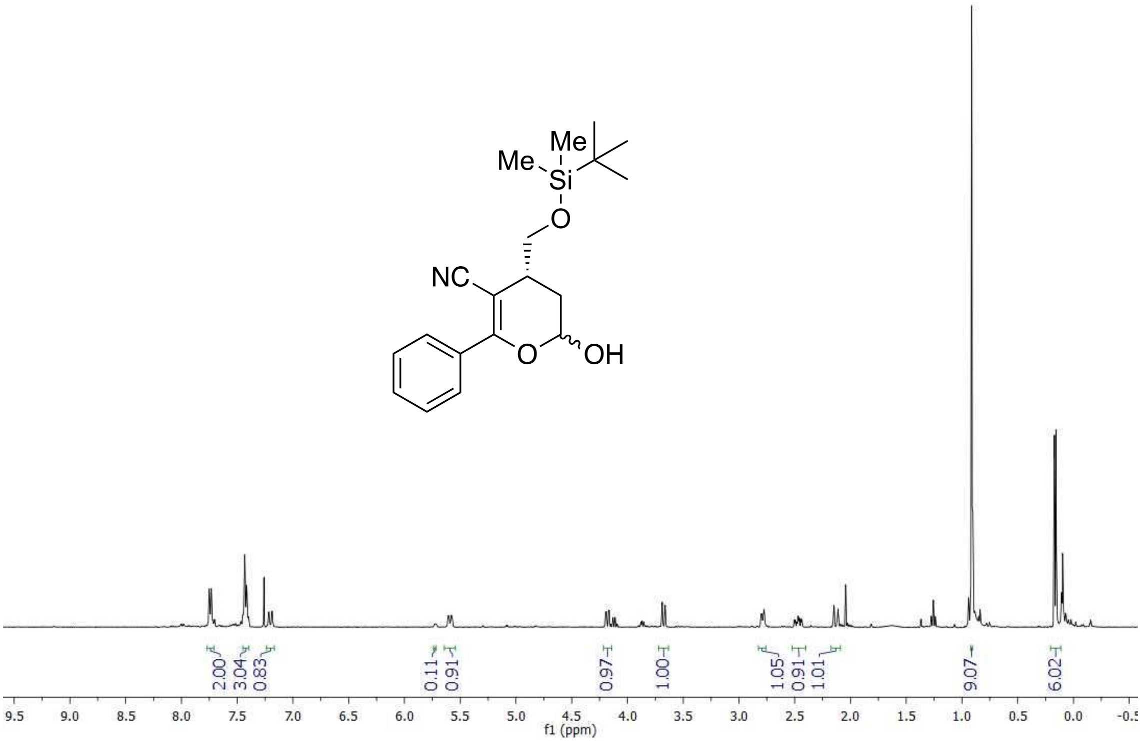

Figure 9. First submission ^{1}H NMR spectrum in $CDCl_3$ of compound **1g**.

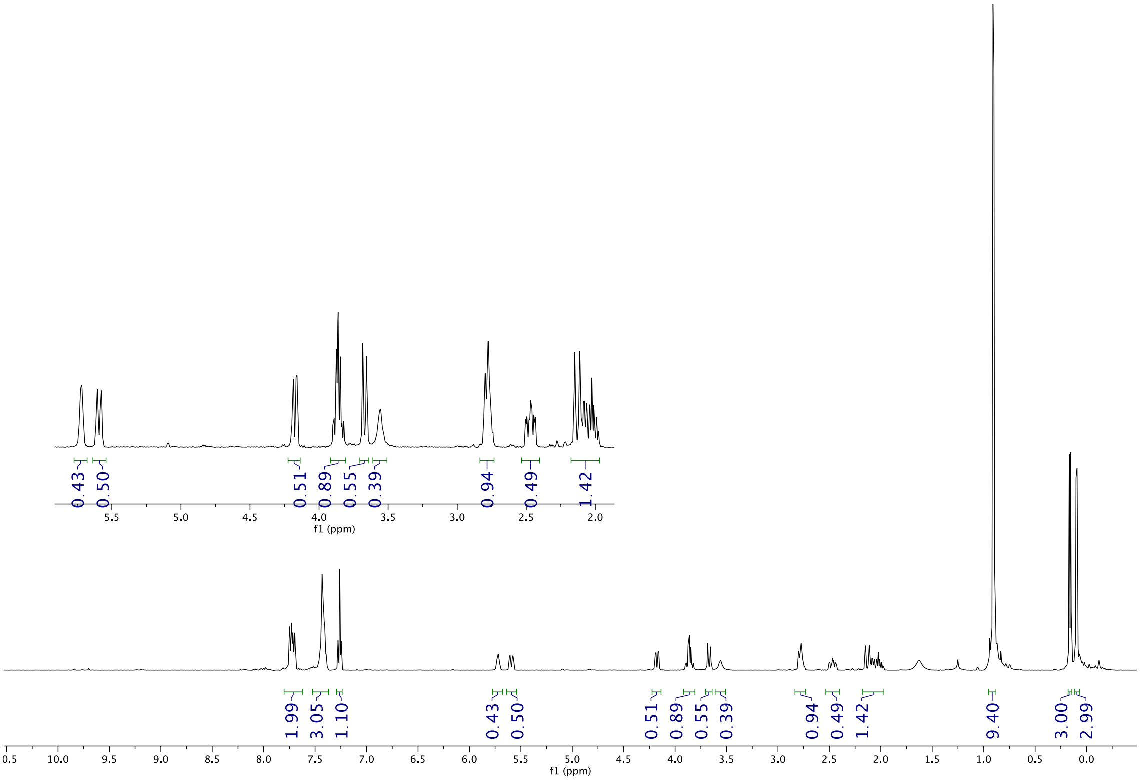

Figure 10. New submission ^{1}H NMR spectrum in $CDCl_3$ of compound **1g**.

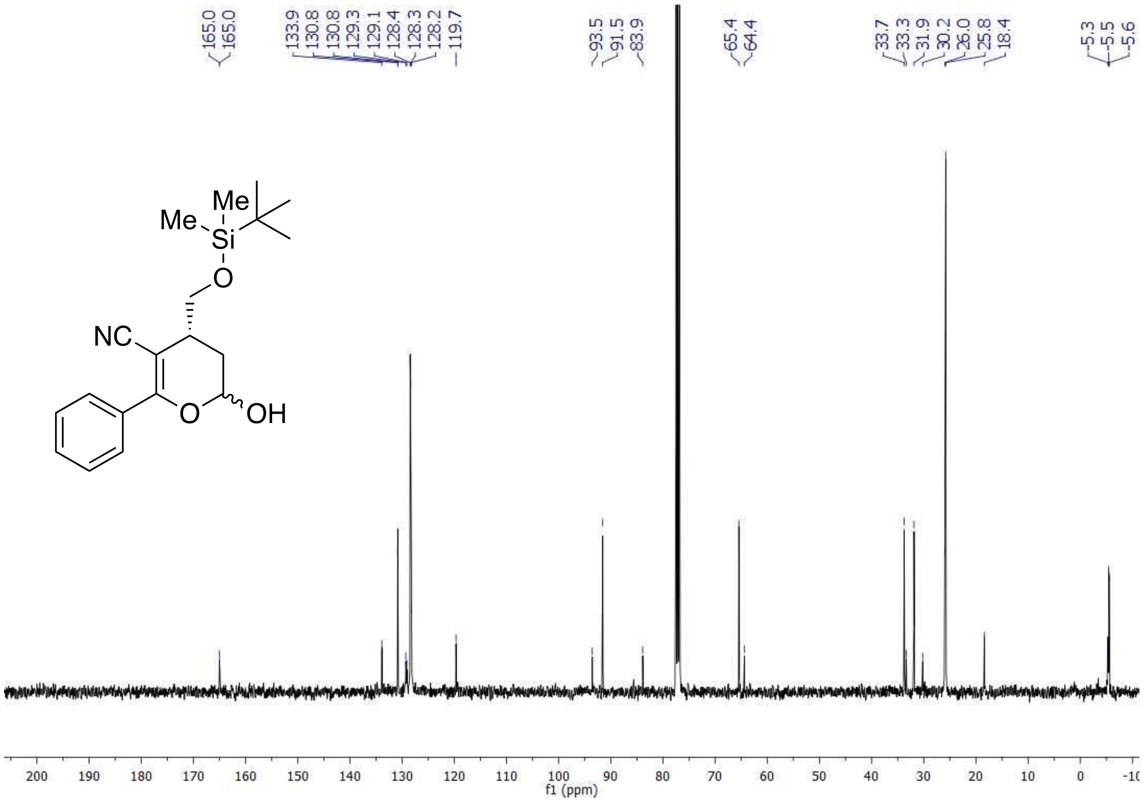

Figure 11. First submission ^{13}C NMR spectrum in $CDCl_3$ of compound **1g**.

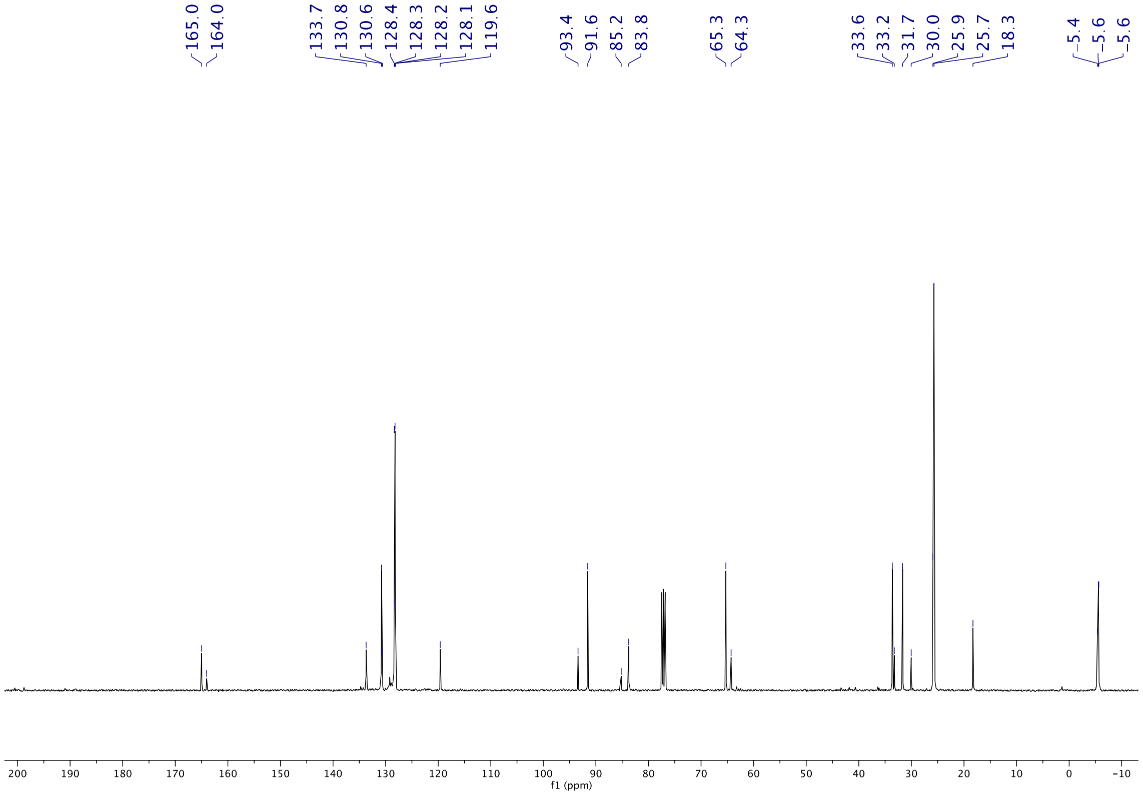

Figure 12. New submission ^{13}C NMR spectrum in $CDCl_3$ of compound **1g**.

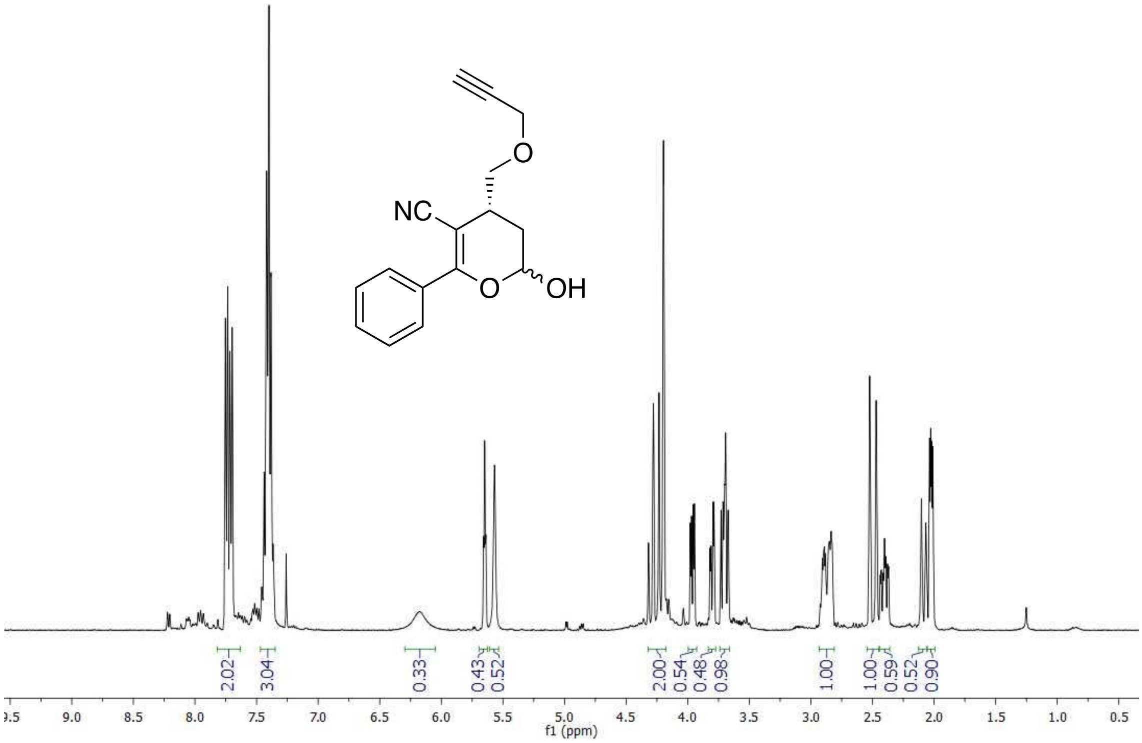

Figure 13. First submission ^{1}H NMR spectrum in $CDCl_3$ of compound **1h**.

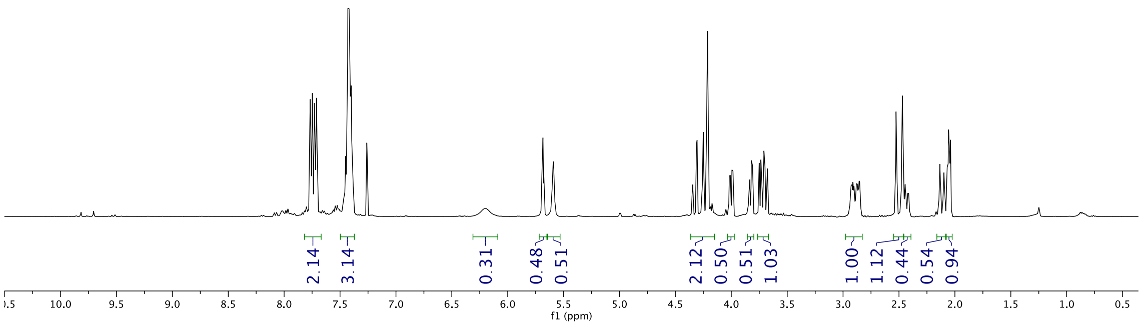

Figure 14. New submission ^{1}H NMR spectrum in $CDCl_3$ of compound **1h**.

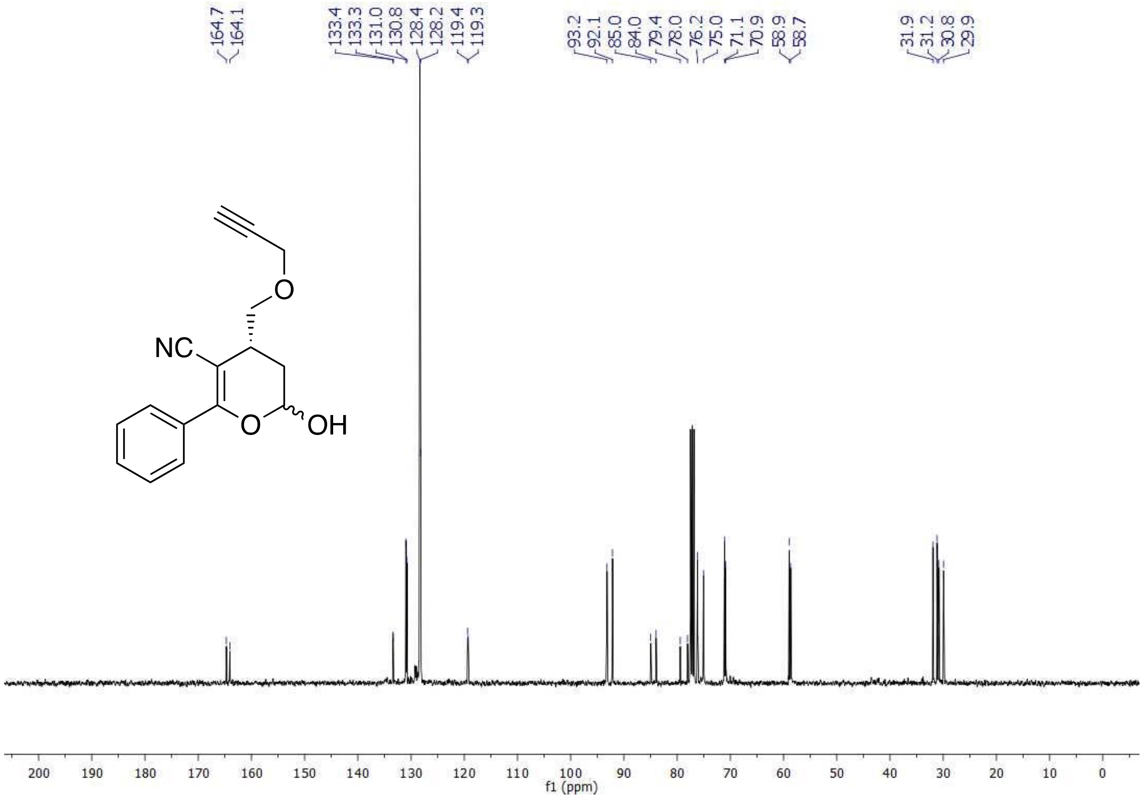

Figure 15. First submission ^{13}C NMR spectrum in $CDCl_3$ of compound **1h**.

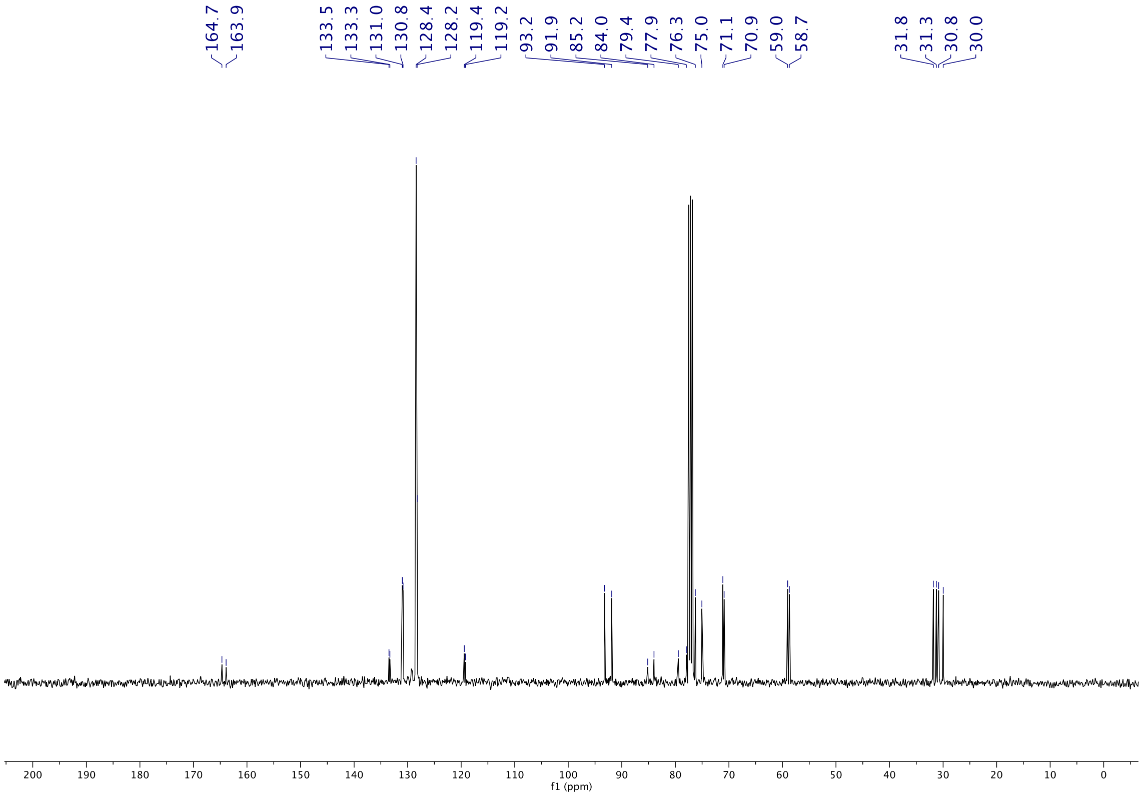

Figure 16. New submission ^{13}C NMR spectrum in $CDCl_3$ of compound **1h**.

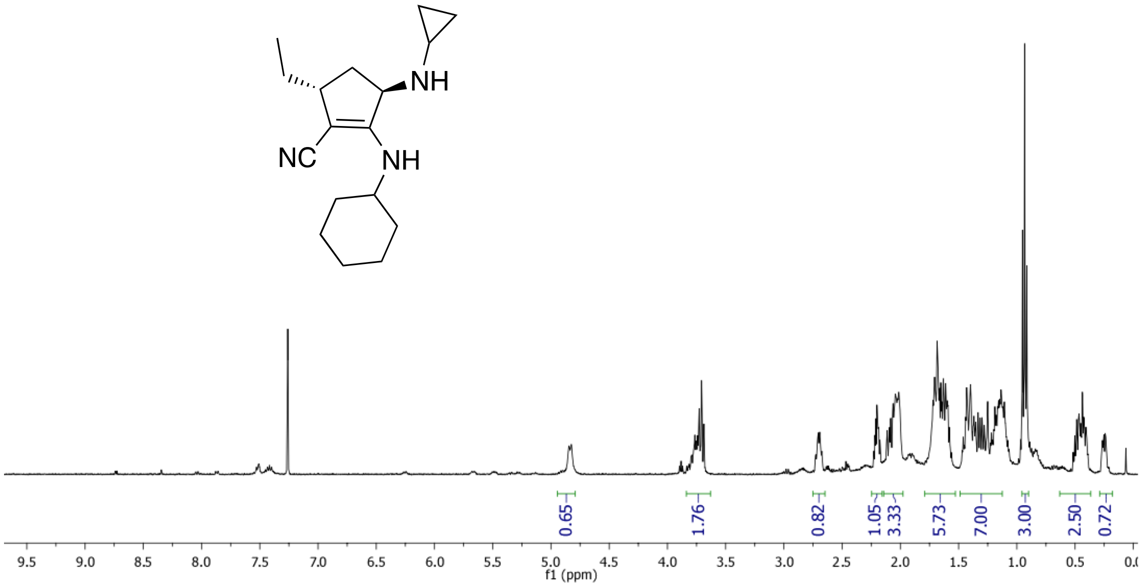

Figure 17. First submission ^{1}H NMR spectrum in $CDCl_3$ of compound **2b**.

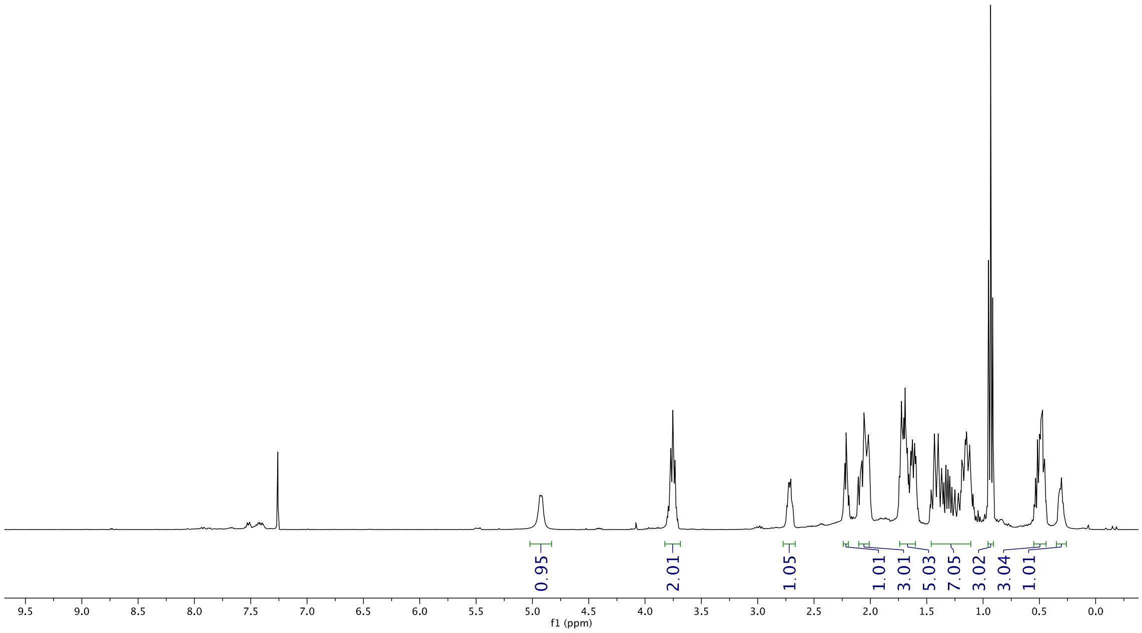

Figure 18. New submission ^{1}H NMR spectrum in $CDCl_3$ of compound **2b**.

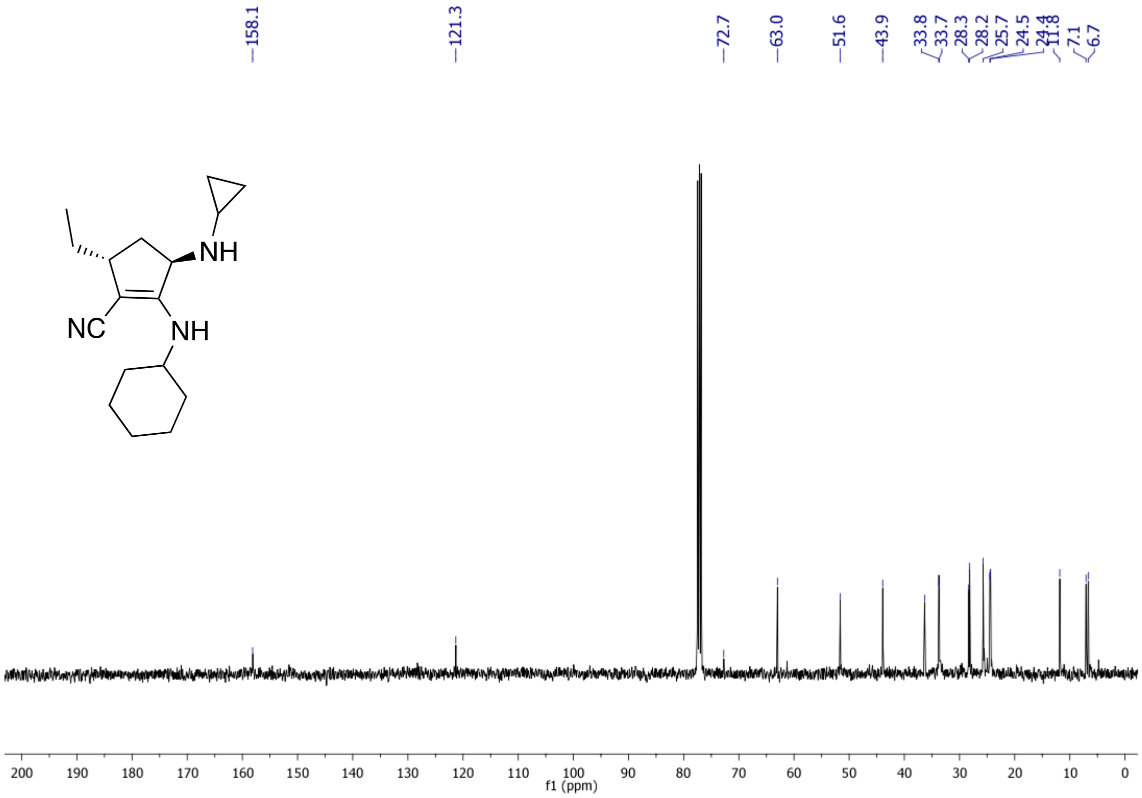

Figure 19. First submission ^{13}C NMR spectrum in $CDCl_3$ of compound **2b**.

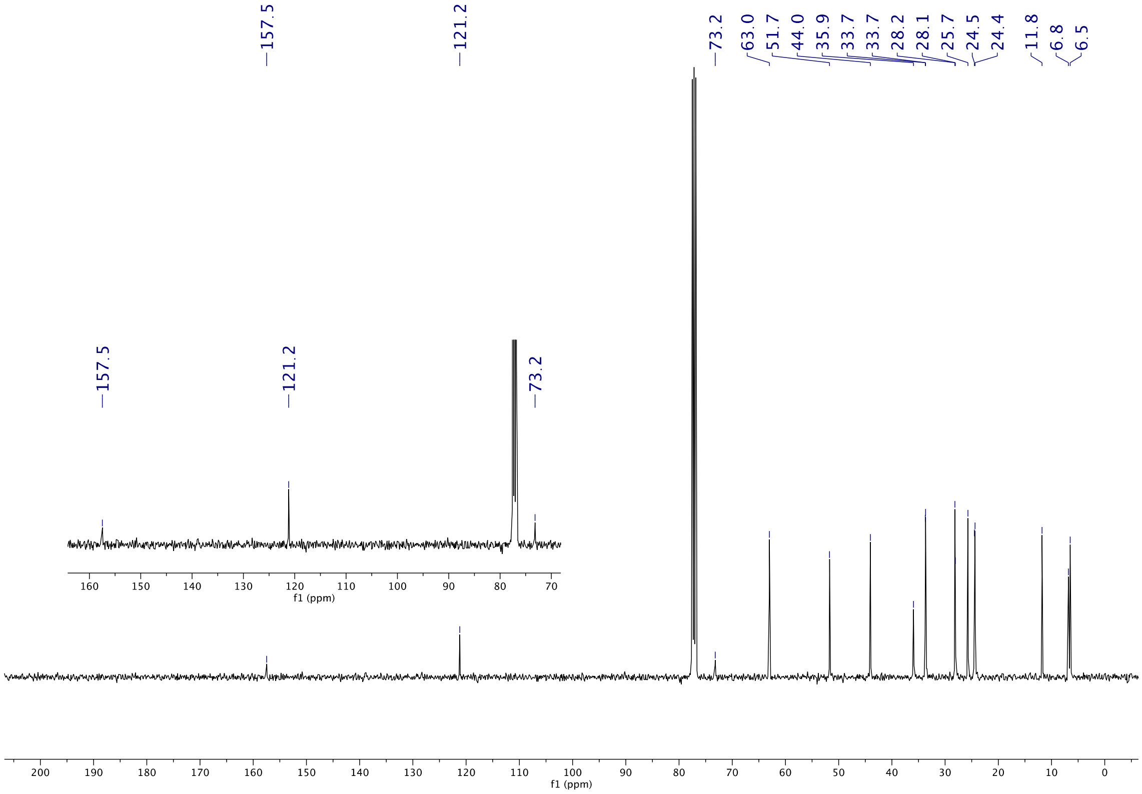

Figure 20. New submission ^{13}C NMR spectrum in $CDCl_3$ of compound **2b**.

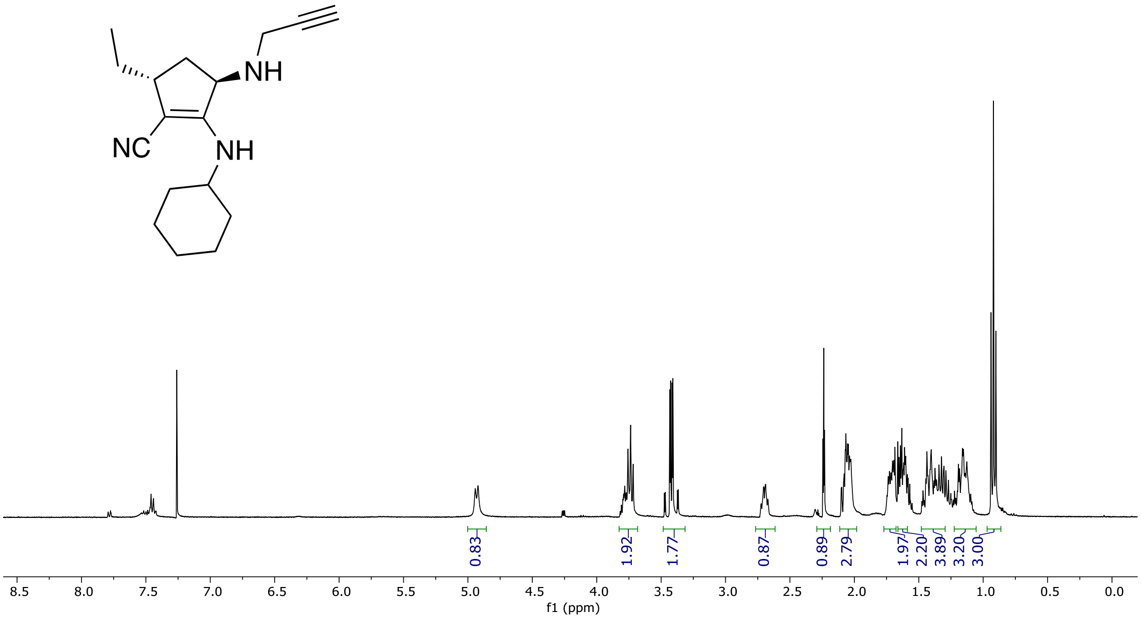

Figure 21. First submission ^{1}H NMR spectrum in $CDCl_3$ of compound **2e**.

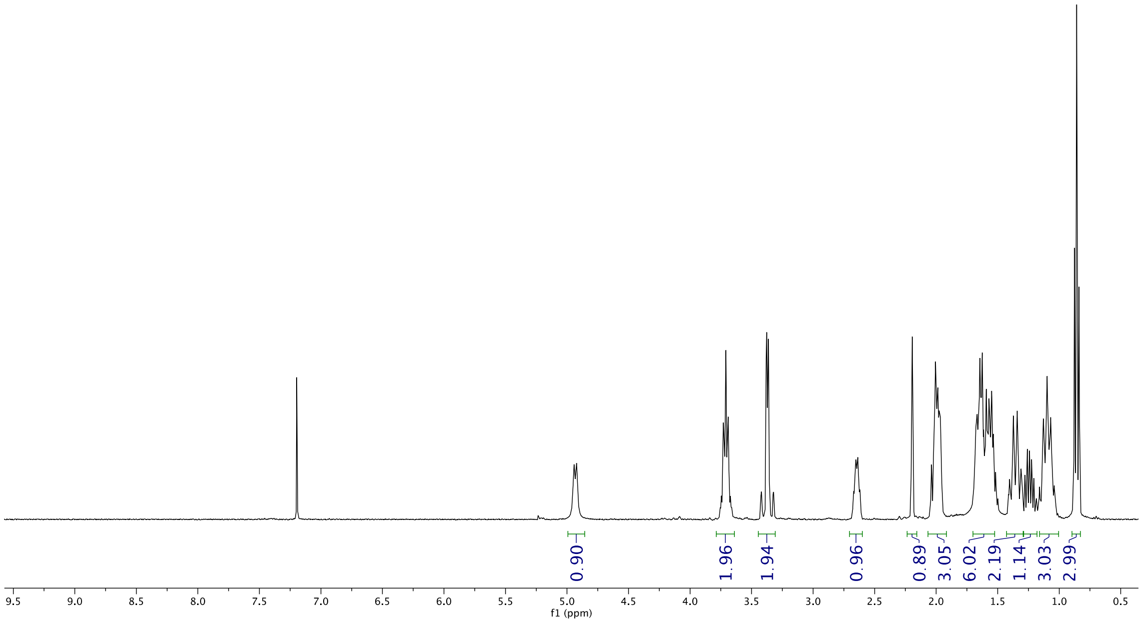

Figure 22. New submission ^{1}H NMR spectrum in $CDCl_3$ of compound **2e**.

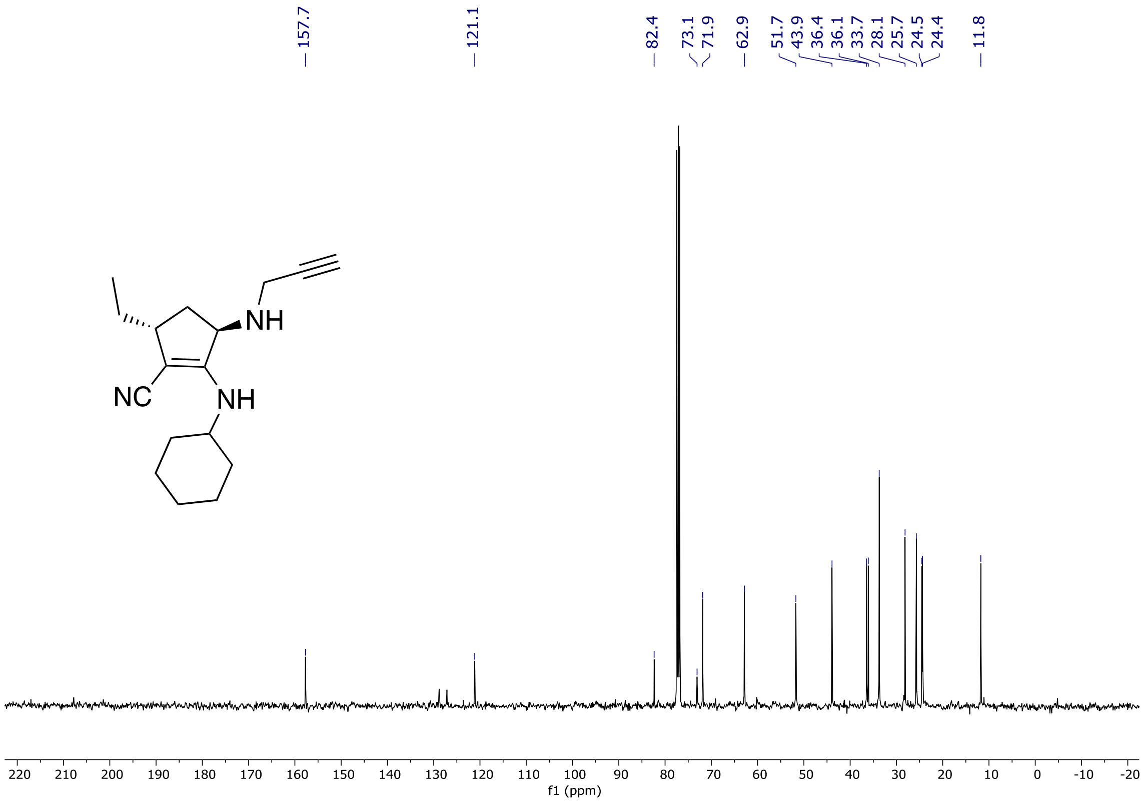

Figure 23. First submission ^{13}C NMR spectrum in $CDCl_3$ of compound **2e**.

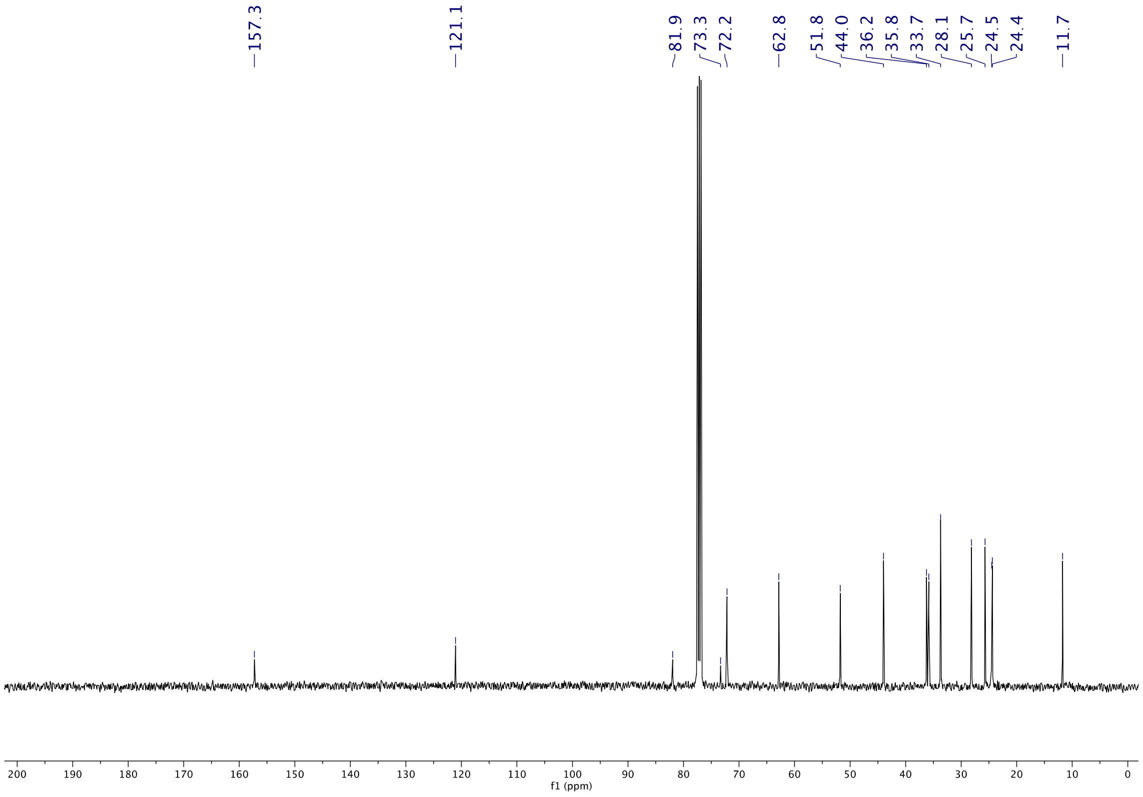

Figure 24. New submission ^{13}C NMR spectrum in $CDCl_3$ of compound **2e**.

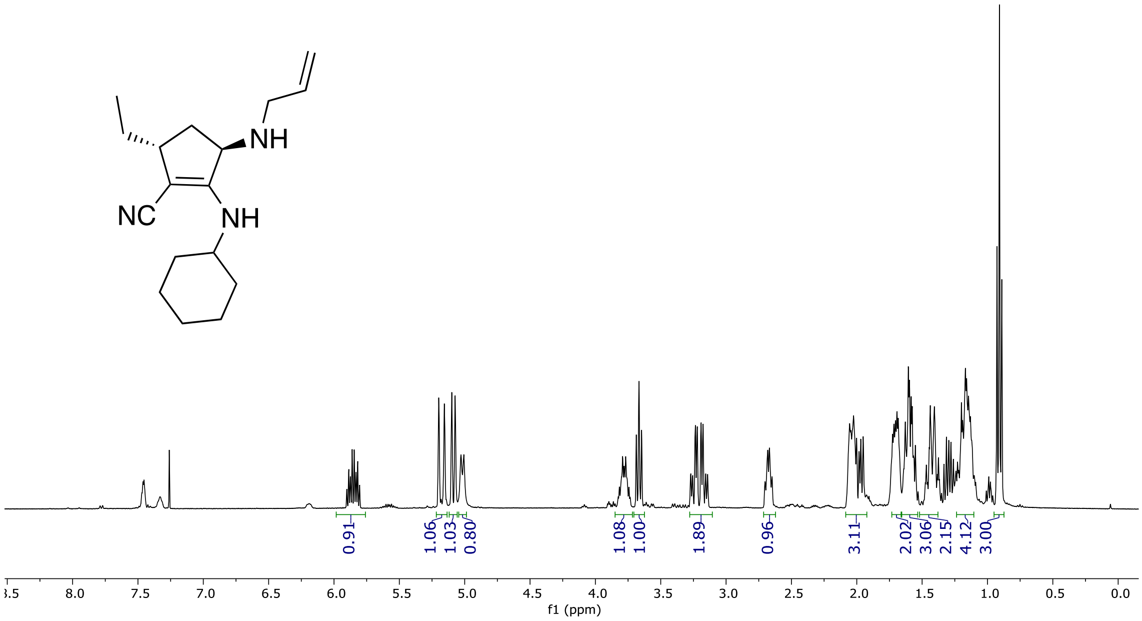

Figure 25. First submission ^{1}H NMR spectrum in $CDCl_3$ of compound **2f**.

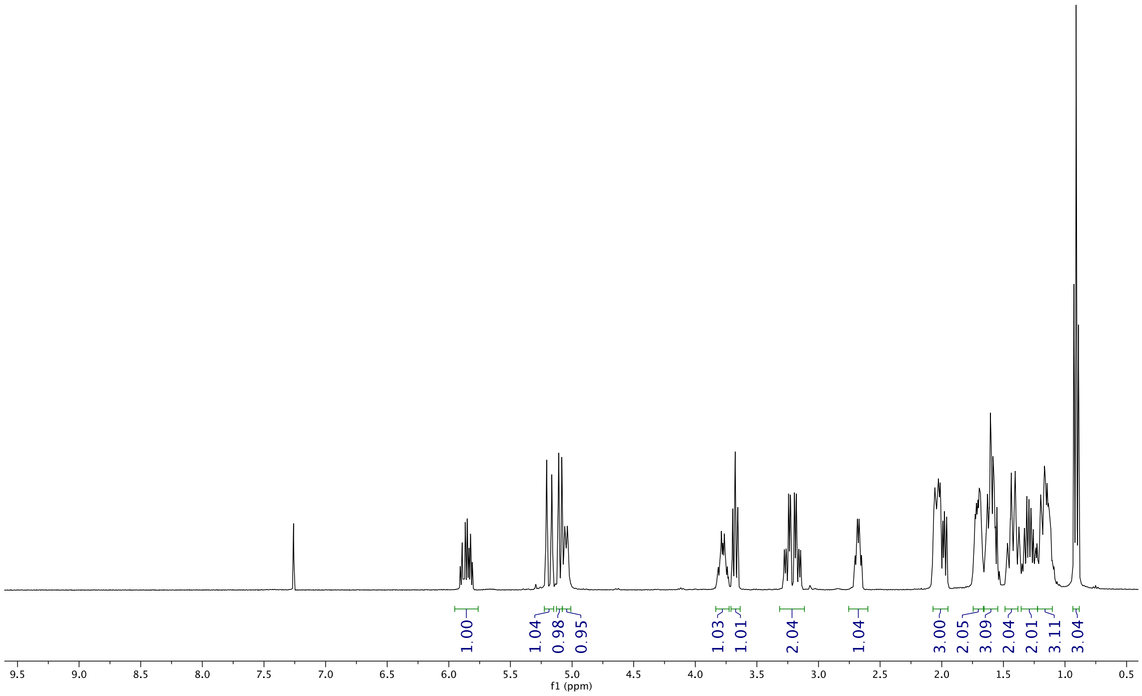

Figure 26. New submission ^{1}H NMR spectrum in $CDCl_3$ of compound **2f**.

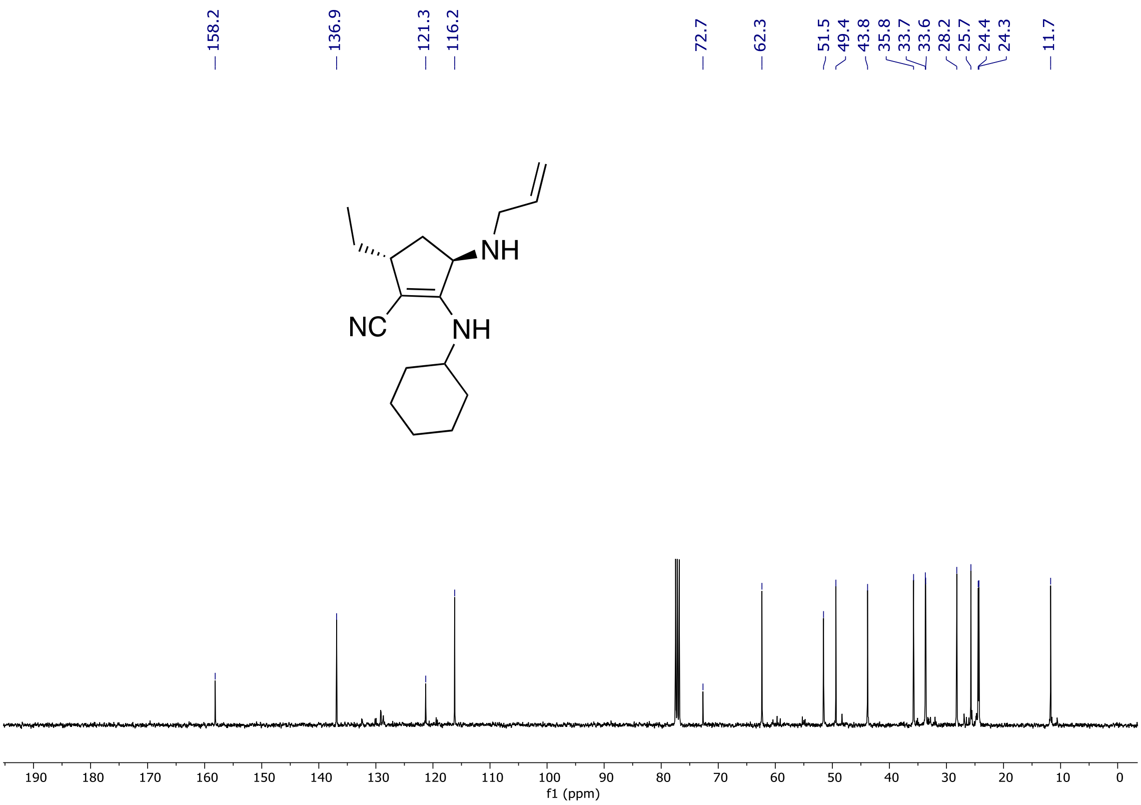

Figure 27. First submission ^{13}C NMR spectrum in $CDCl_3$ of compound **2f**.

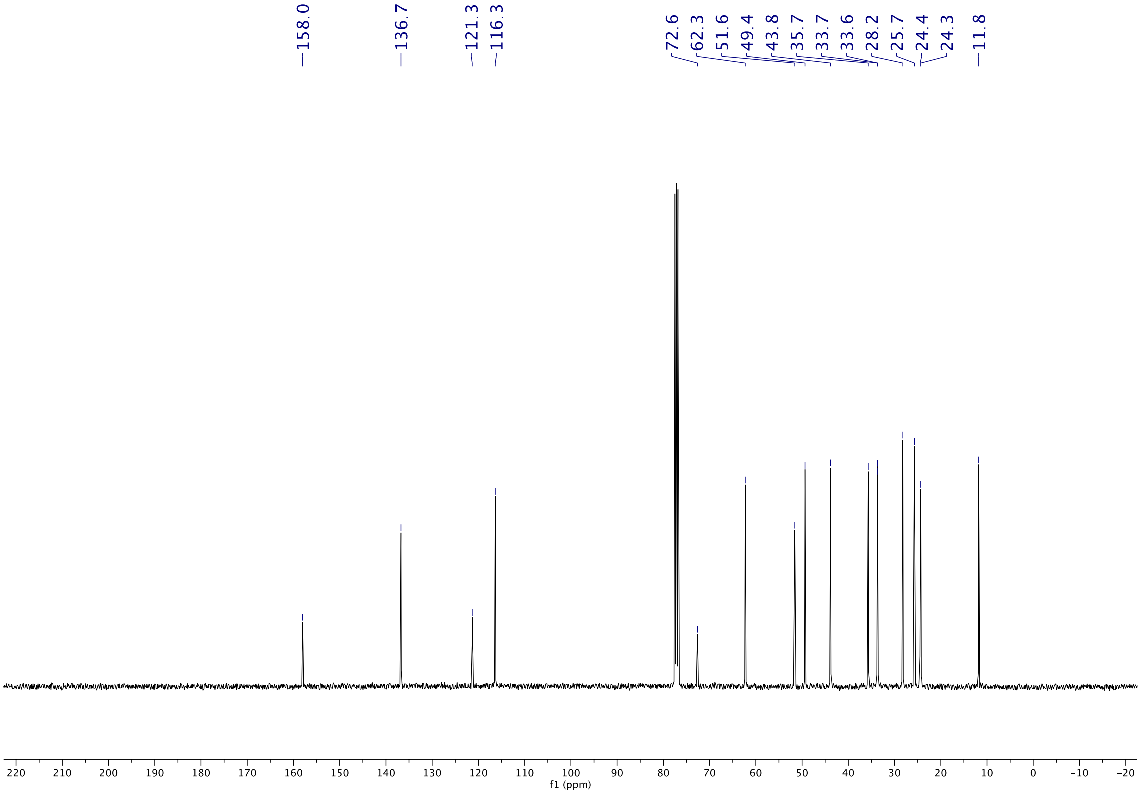

Figure 28. New submission ^{13}C NMR spectrum in $CDCl_3$ of compound **2f**.

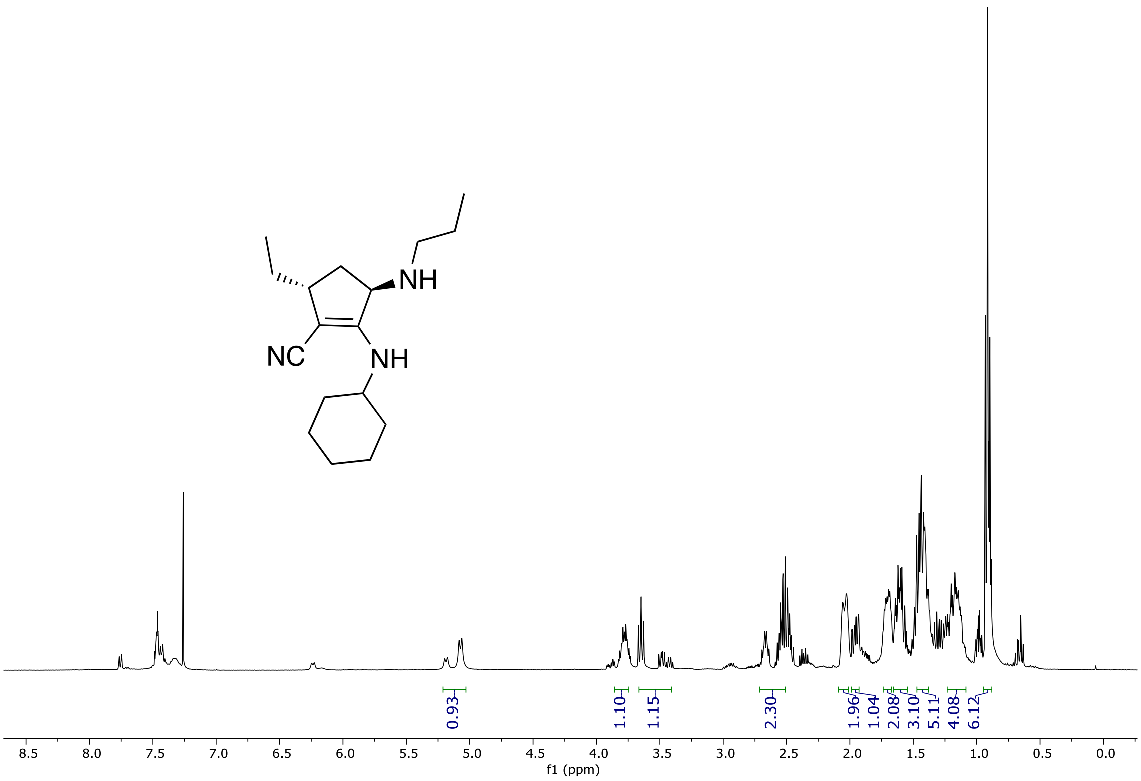

Figure 29. First submission ^{1}H NMR spectrum in $CDCl_3$ of compound **2g**.

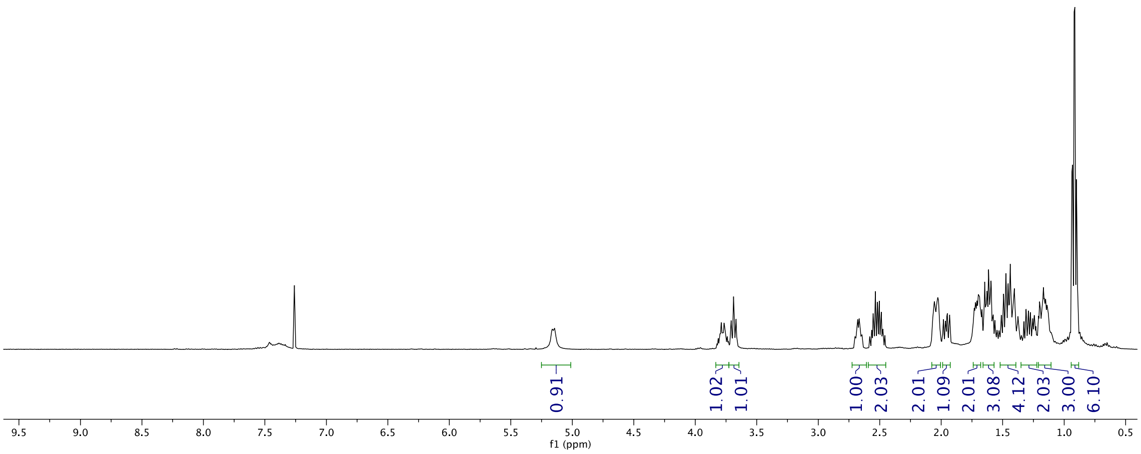

Figure 30. New submission ^{1}H NMR spectrum in $CDCl_3$ of compound **2g**.

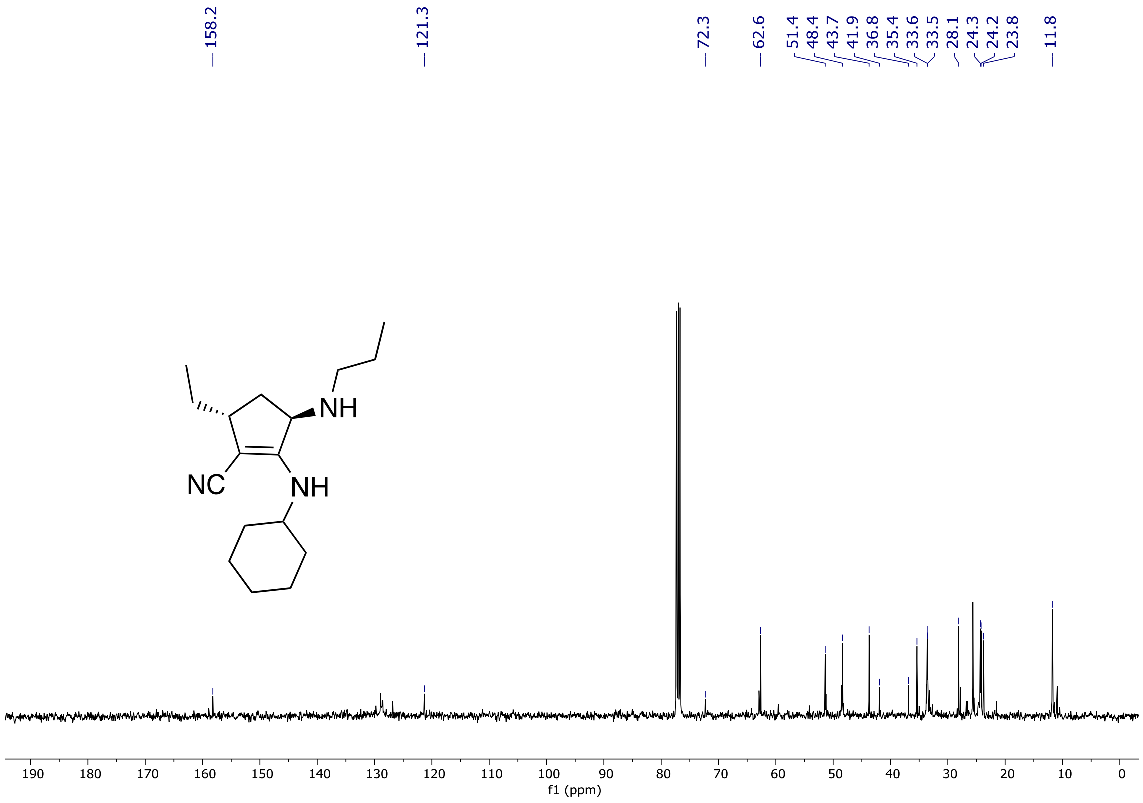

Figure 31. First submission ^{13}C NMR spectrum in $CDCl_3$ of compound **2g**.

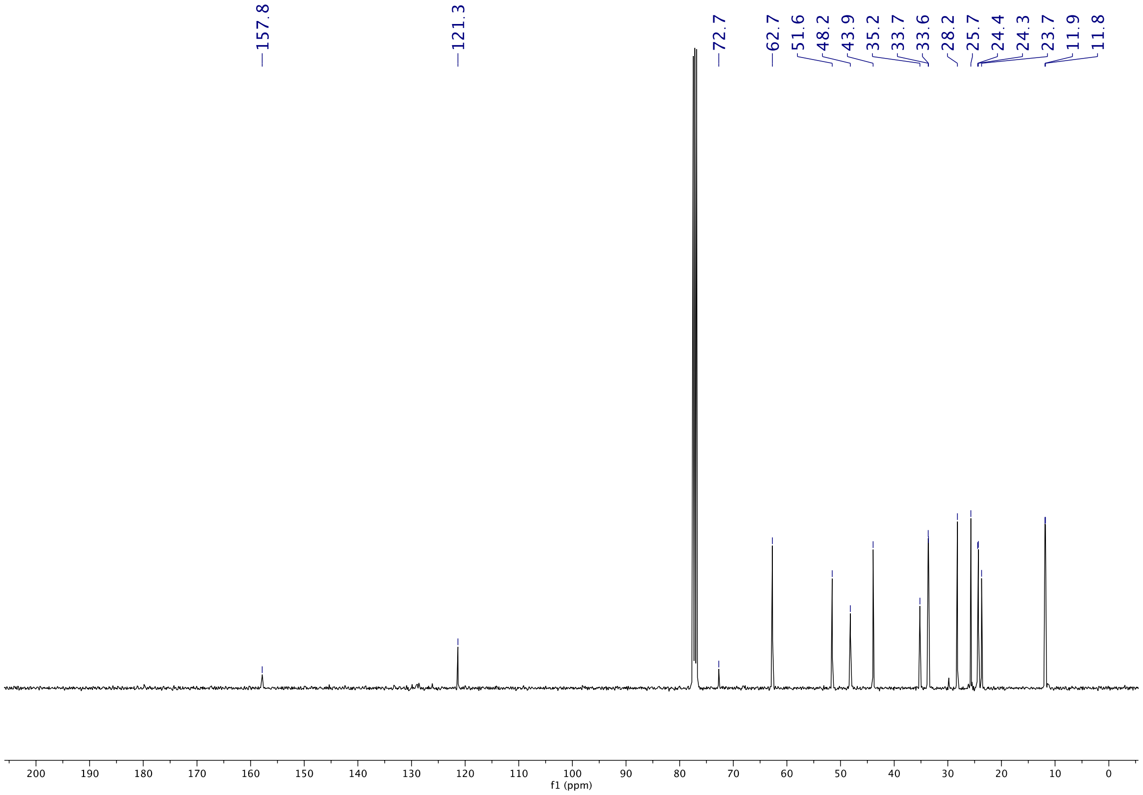

Figure 32. New submission ^{13}C NMR spectrum in $CDCl_3$ of compound **2g**.

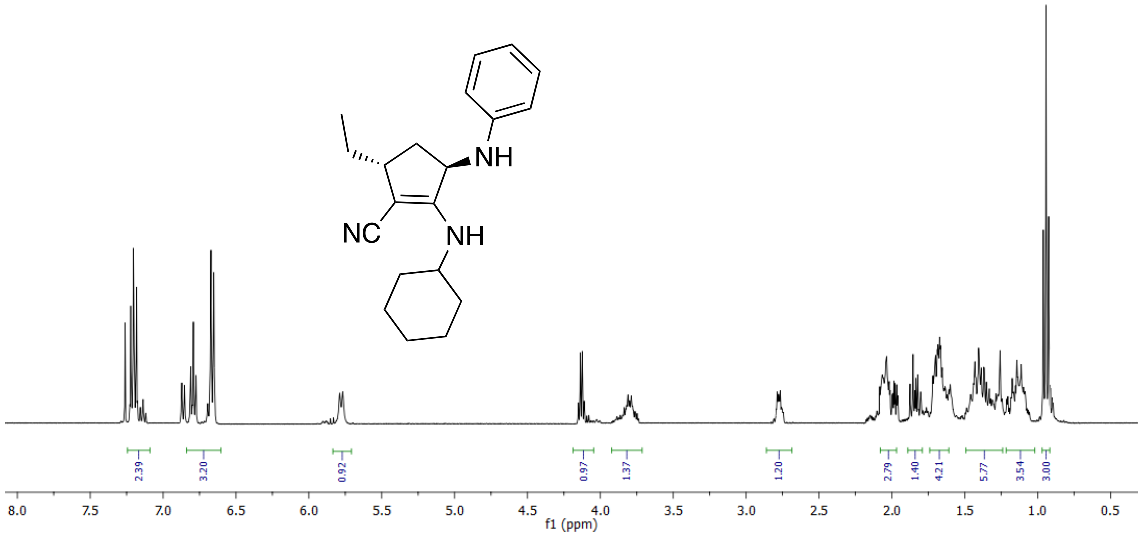

Figure 33. First submission ^{1}H NMR spectrum in $CDCl_3$ of compound **2k**.

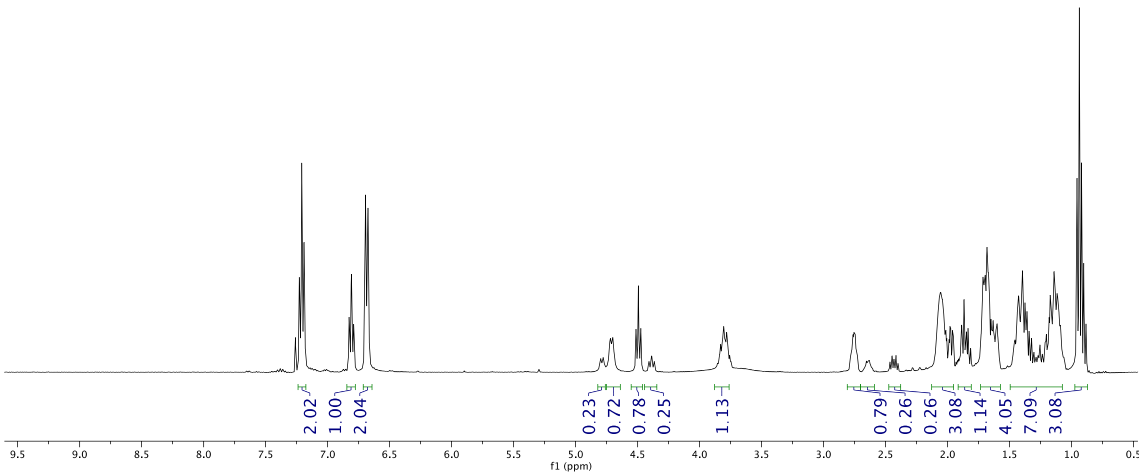

Figure 34. New submission ^{1}H NMR spectrum in $CDCl_3$ of compound **2k**.

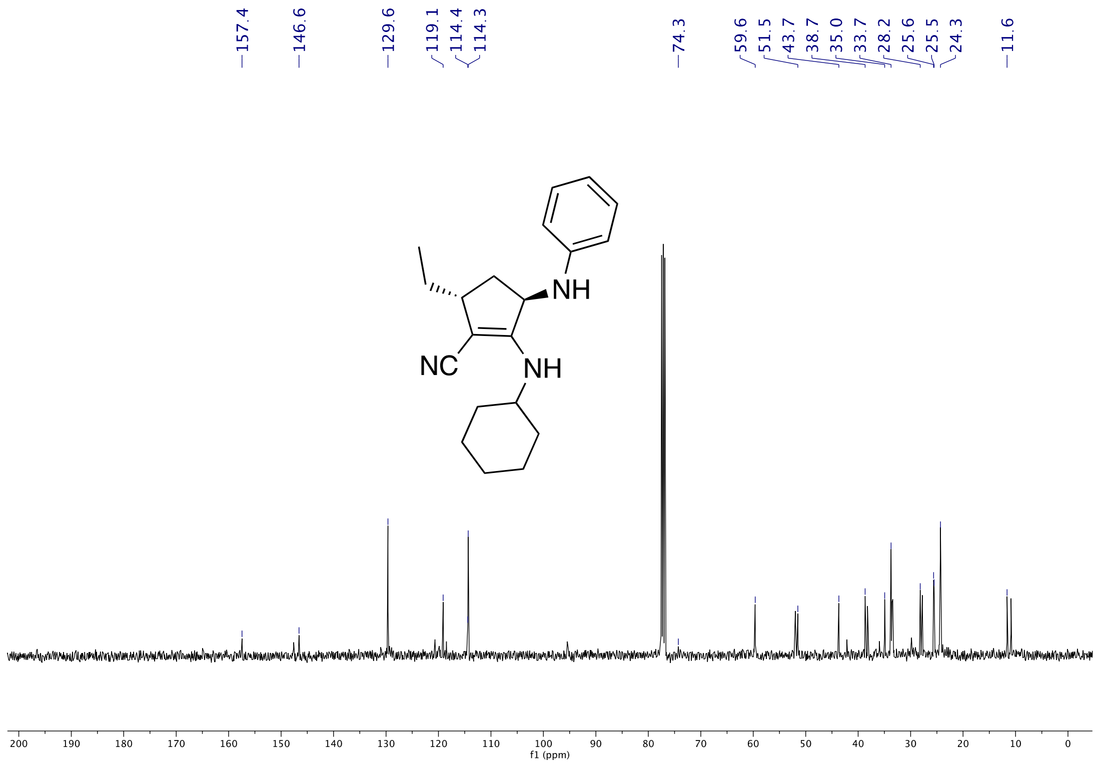

Figure 35. First submission ^{13}C NMR spectrum in $CDCl_3$ of compound **2k**.

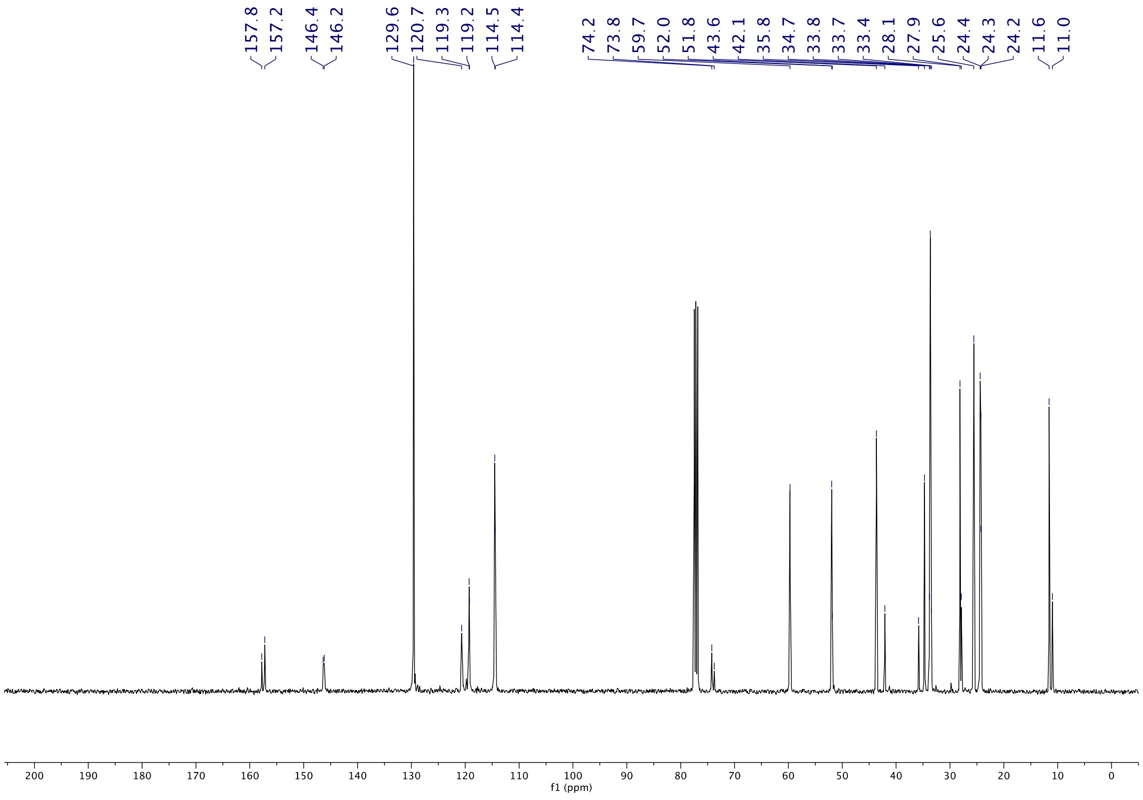

Figure 36. New submission ^{13}C NMR spectrum in $CDCl_3$ of compound **2k**.

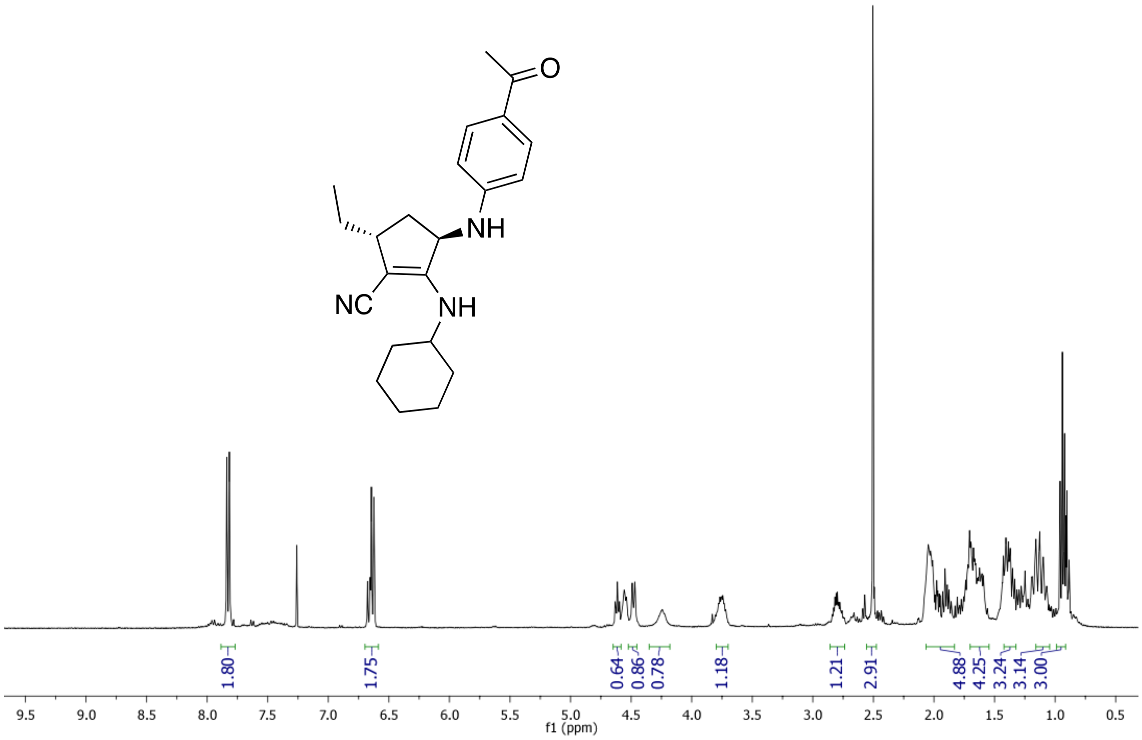

Figure 37. First submission ¹H NMR spectrum in $CDCl_3$ of compound **2p**.

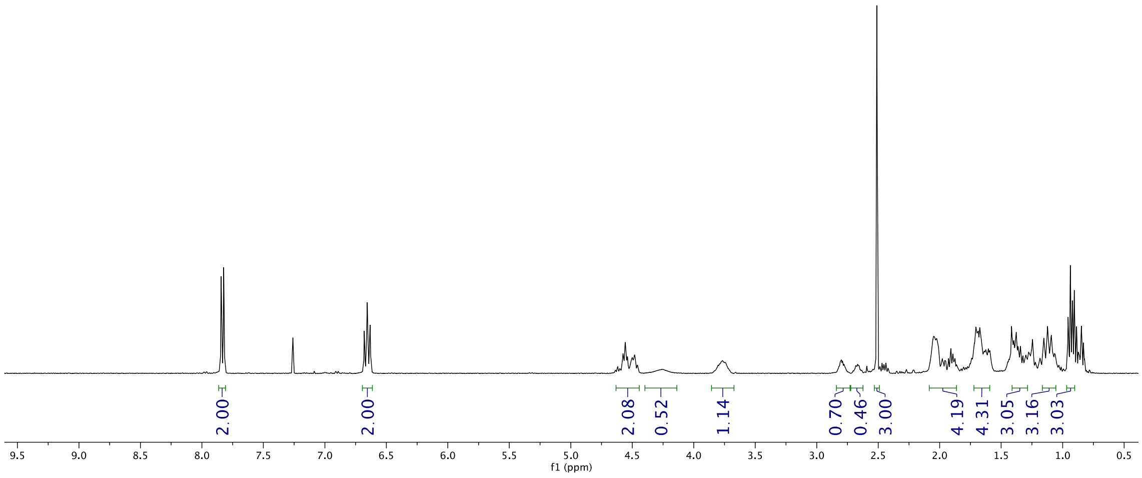

Figure 38. New submission ¹H NMR spectrum in $CDCl_3$ of compound **2p**.

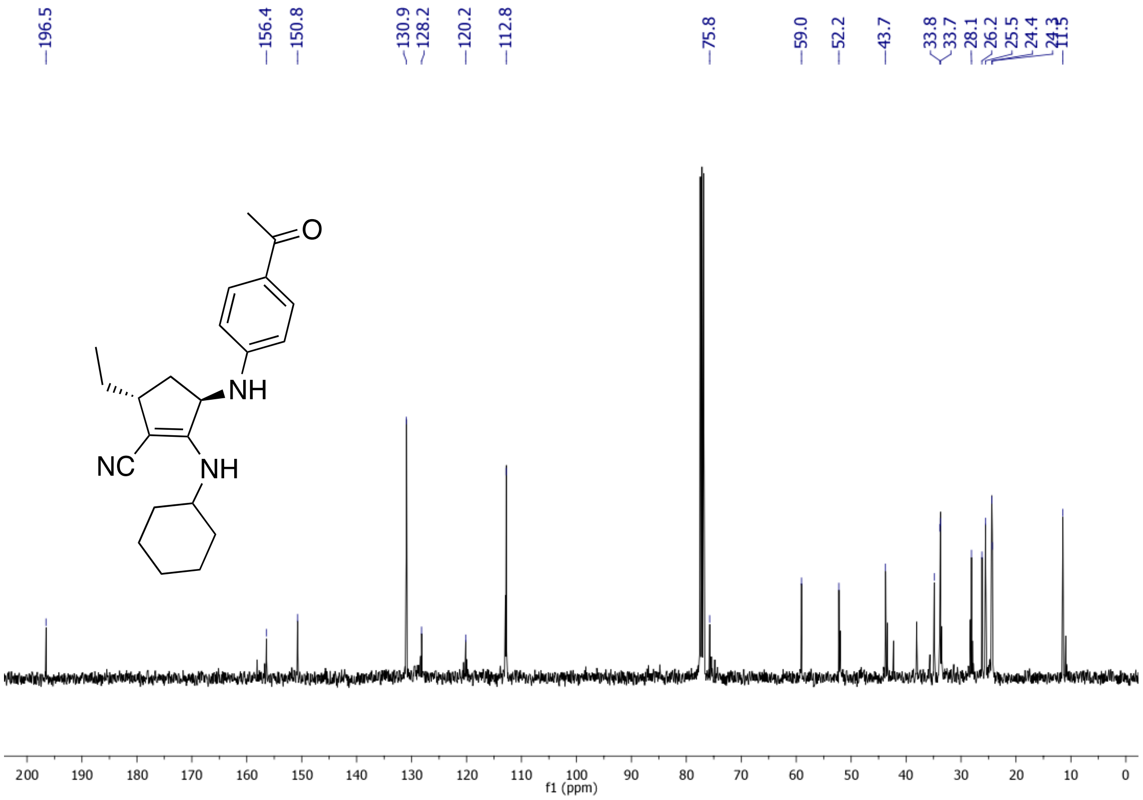

Figure 39. First submission ^{13}C NMR spectrum in $CDCl_3$ of compound **2p**.

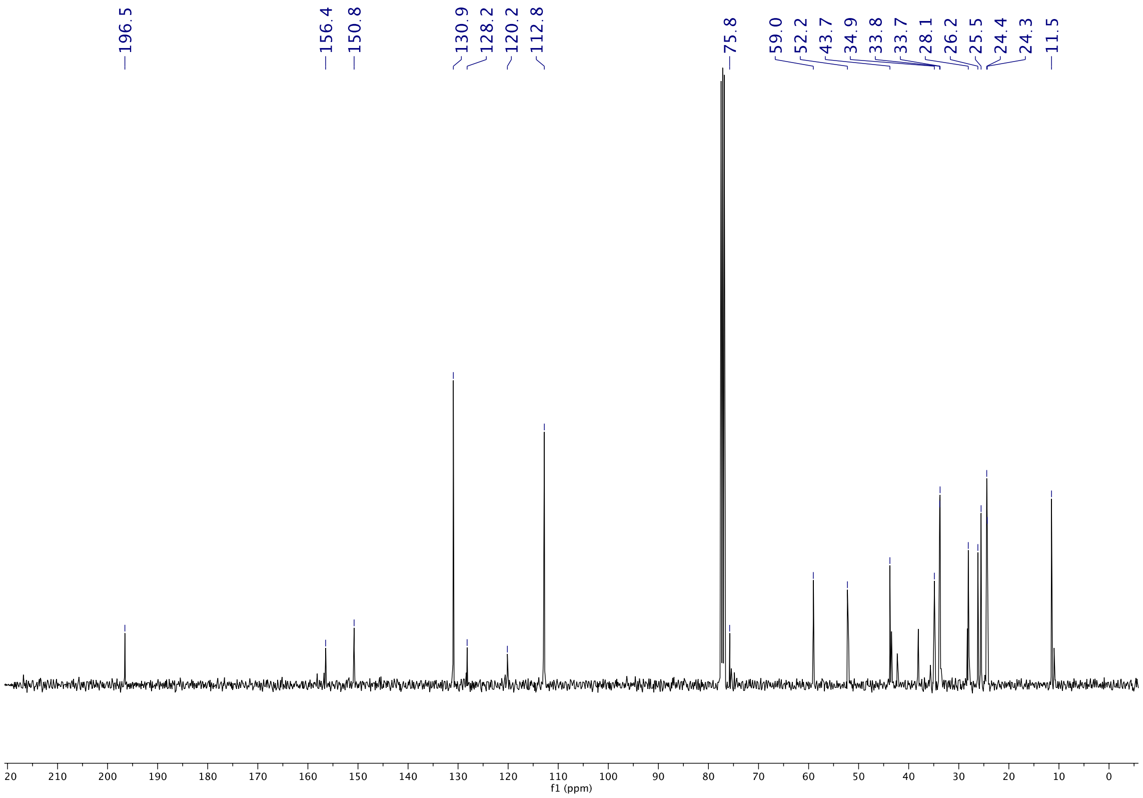

Figure 40. New submission ^{13}C NMR spectrum in $CDCl_3$ of compound **2p**.

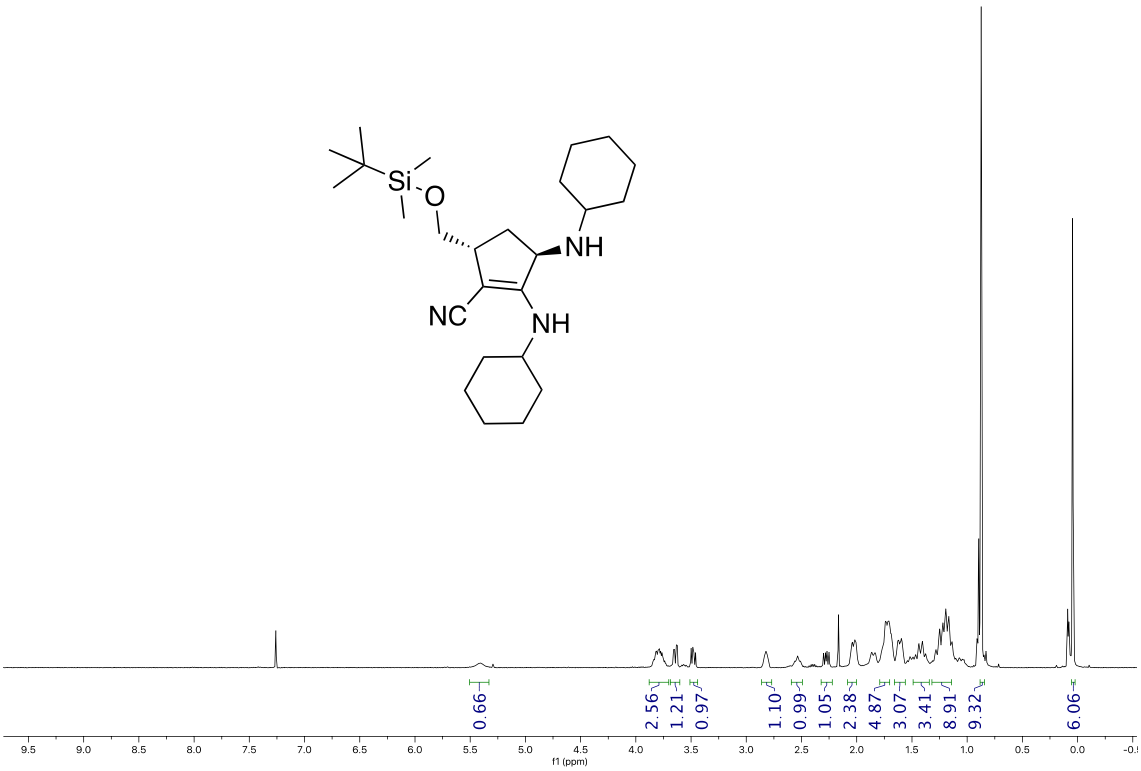

Figure 41. First submission ^{1}H NMR spectrum in $CDCl_3$ of compound **2ag**.

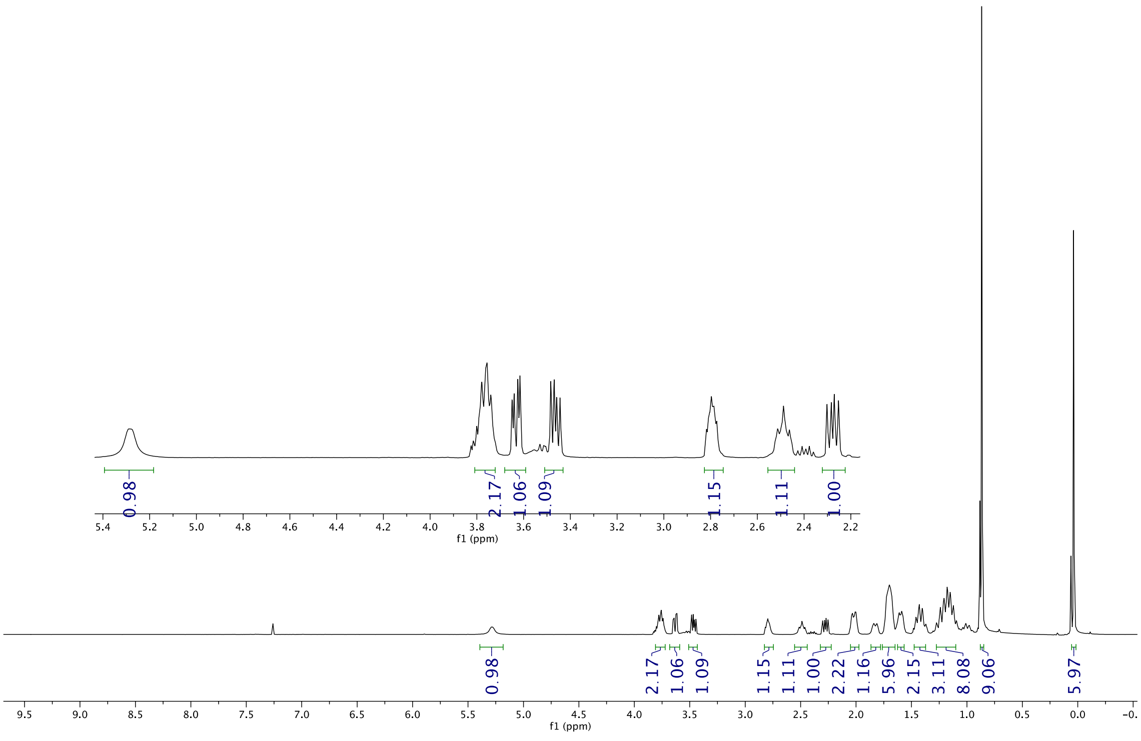

Figure 42. New submission ^{1}H NMR spectrum in $CDCl_3$ of compound **2ag**.

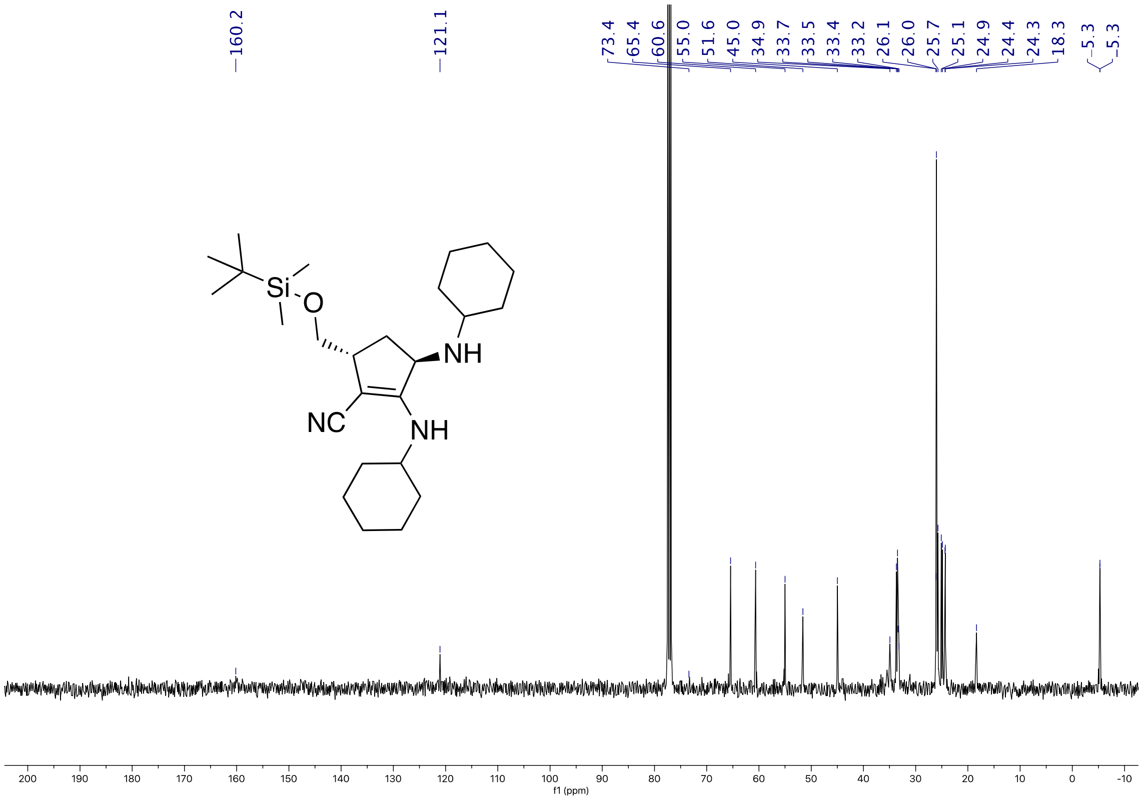

Figure 43. First submission ^{13}C NMR spectrum in $CDCl_3$ of compound **2ag**.

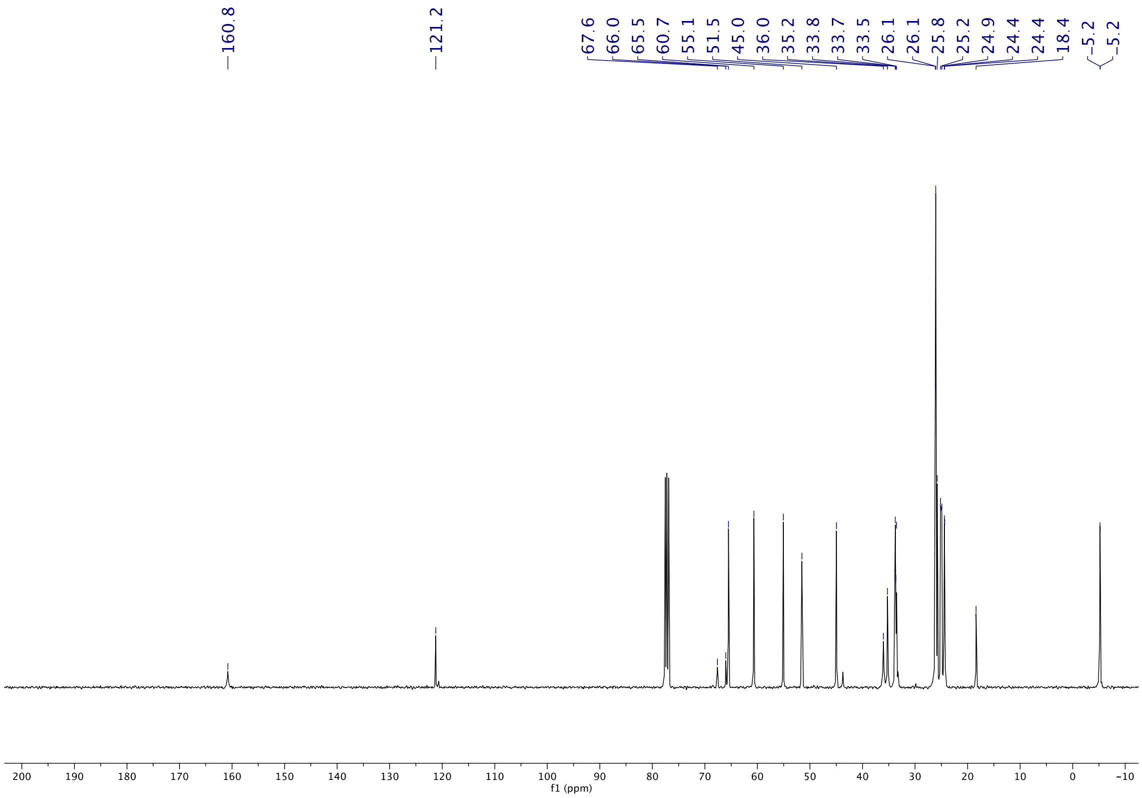

Figure 44. New submission ^{13}C NMR spectrum in $CDCl_3$ of compound **2ag**.

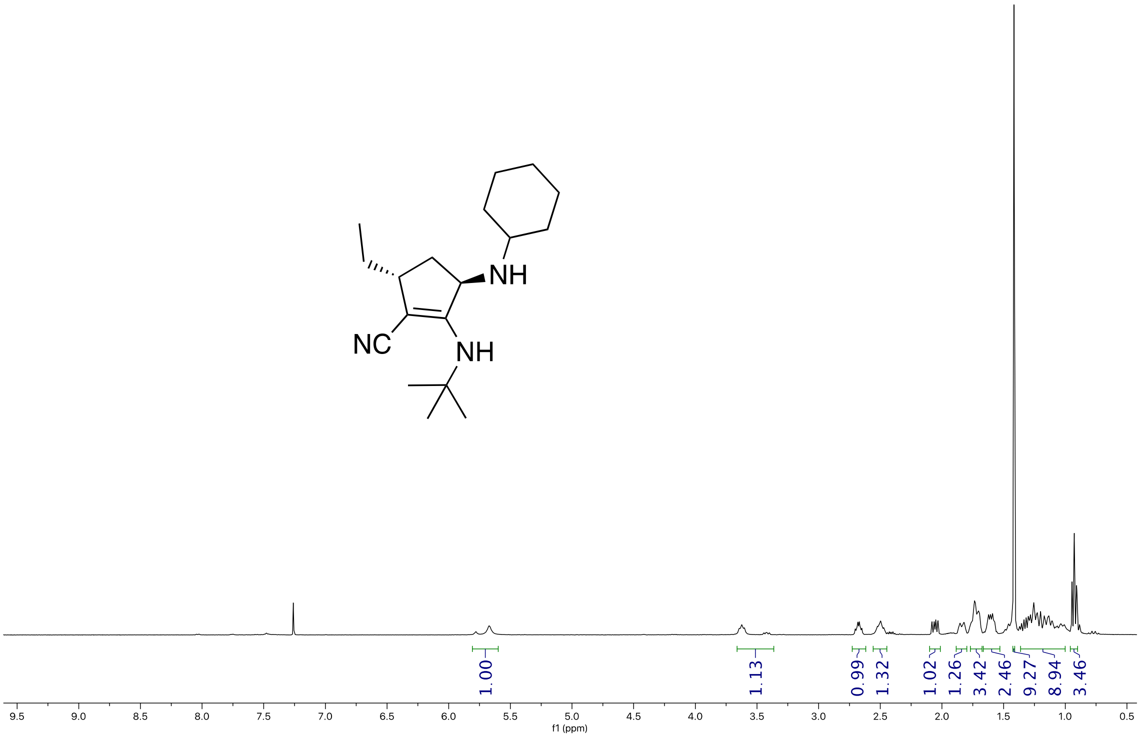

Figure 45. First submission ^{1}H NMR spectrum in $CDCl_3$ of compound **2ai**.

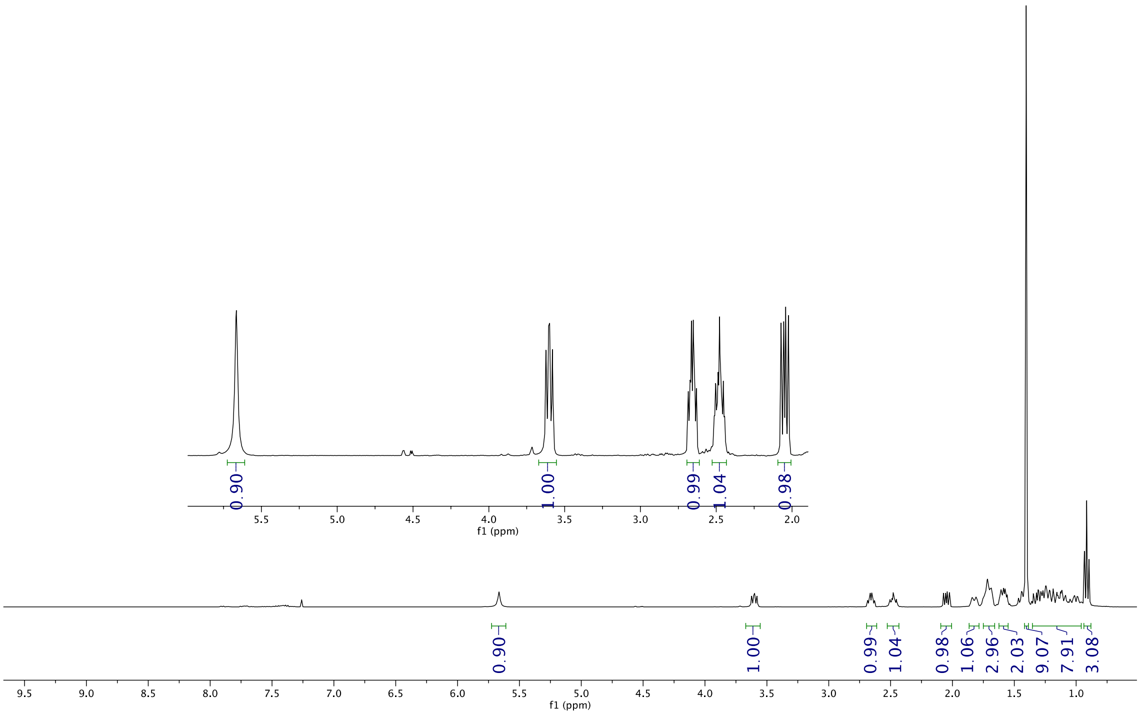

Figure 46. New submission ^{1}H NMR spectrum in $CDCl_3$ of compound **2ai**.

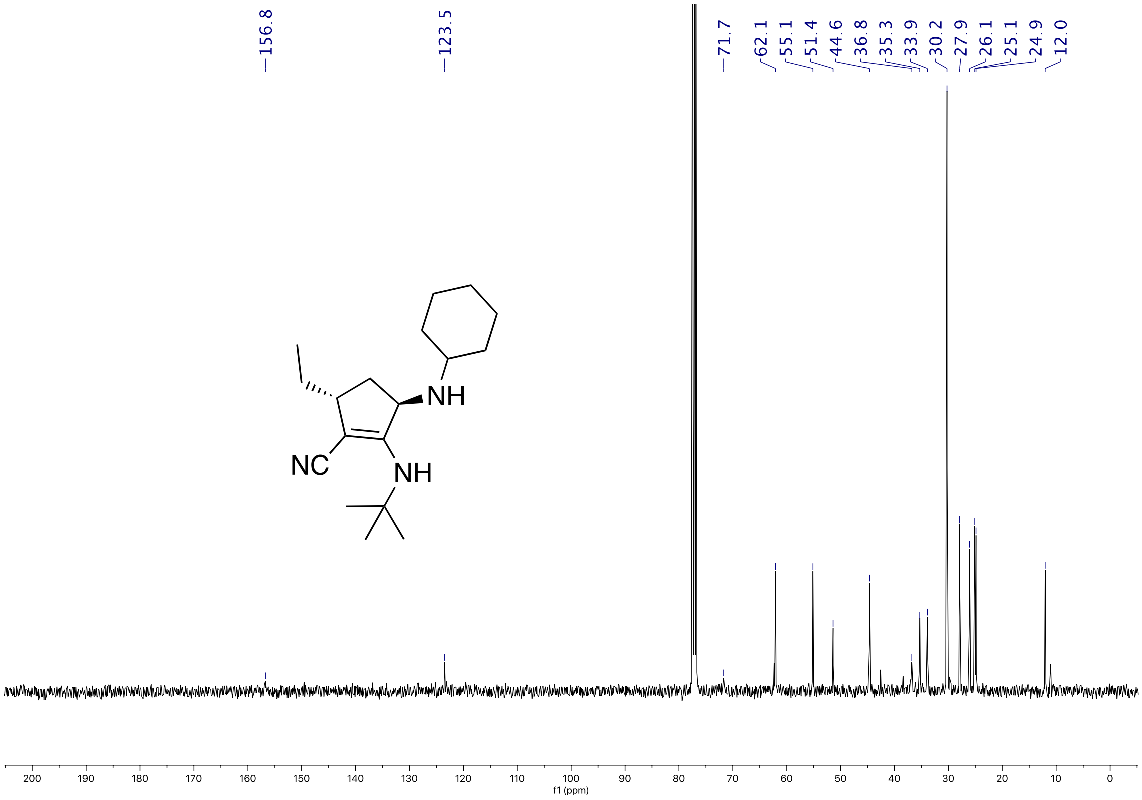

Figure 47. First submission ^{13}C NMR spectrum in $CDCl_3$ of compound **2ai**.

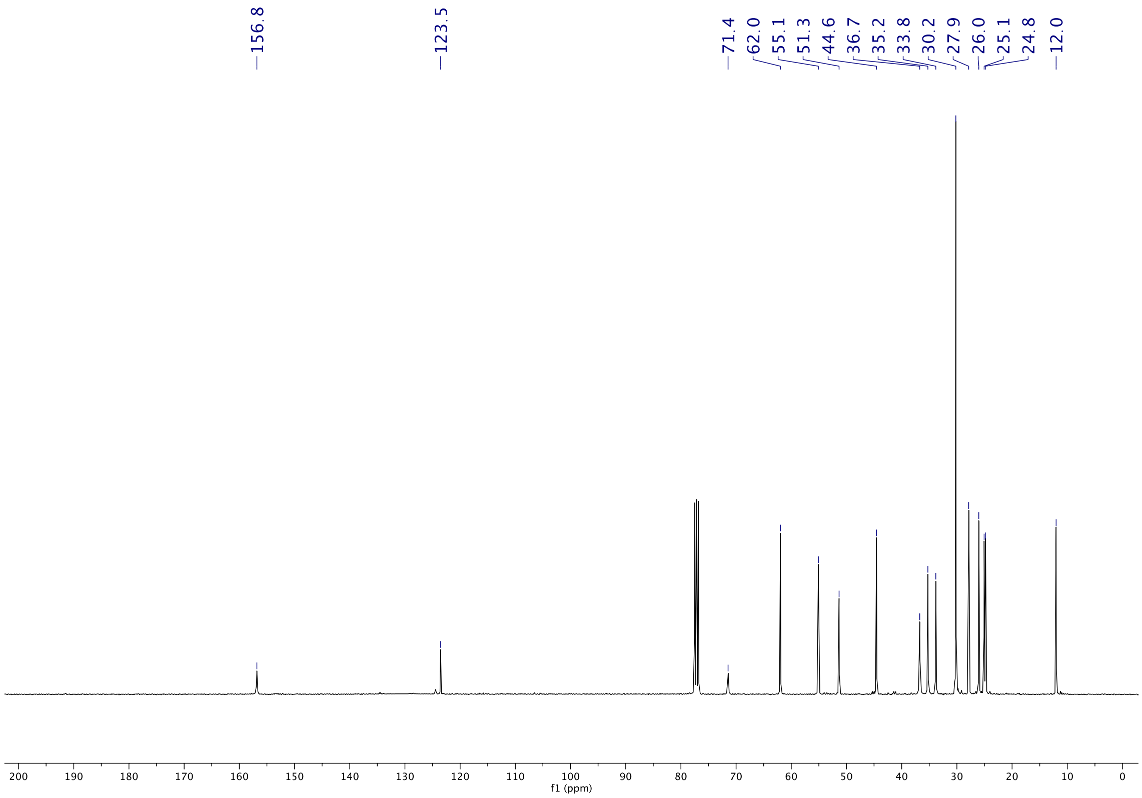

Figure 48. New submission ^{13}C NMR spectrum in $CDCl_3$ of compound **2ai**.

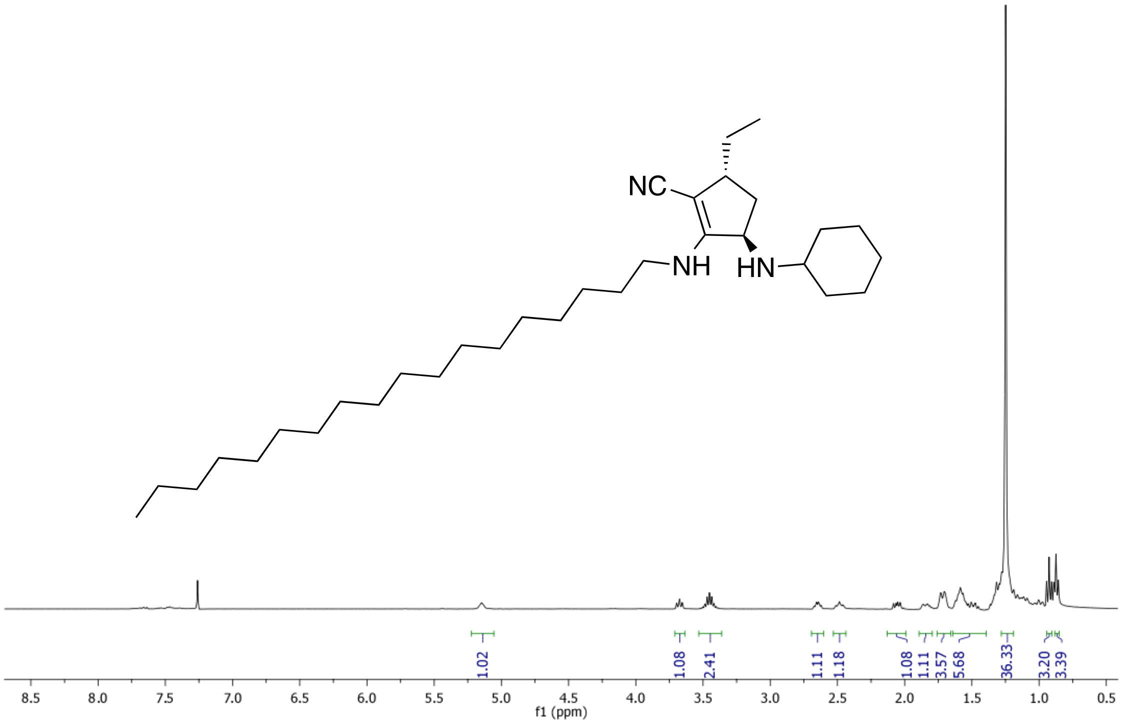

Figure 49. First submission ^{1}H NMR spectrum in $CDCl_3$ of compound **2aj**.

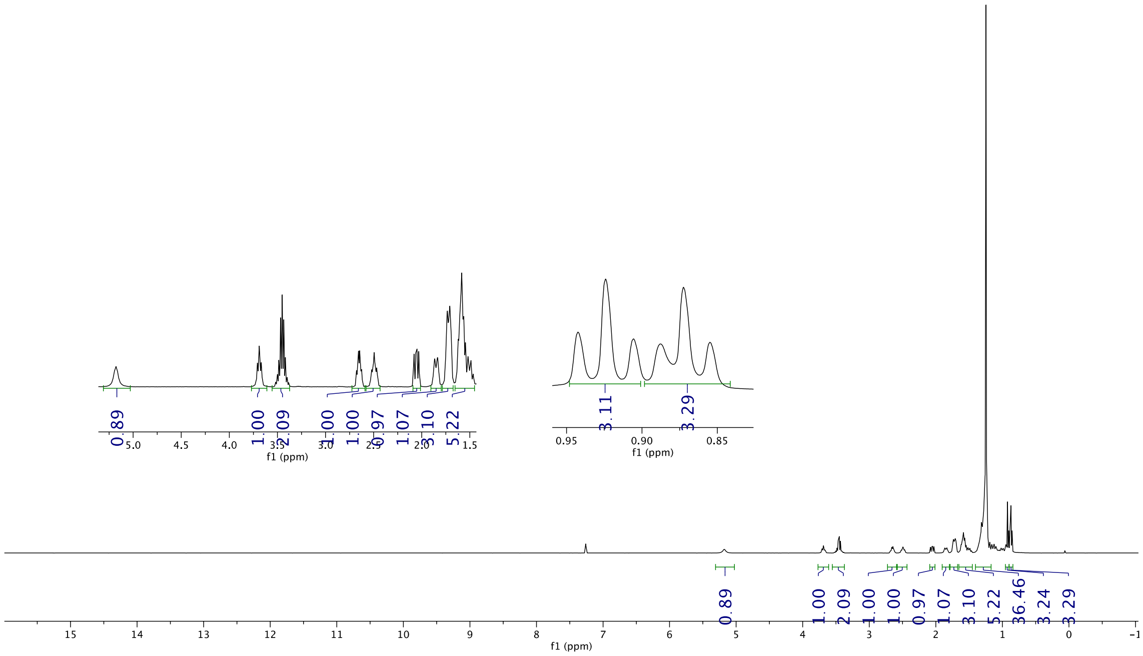

Figure 50. New submission ^{1}H NMR spectrum in $CDCl_3$ of compound **2aj**.

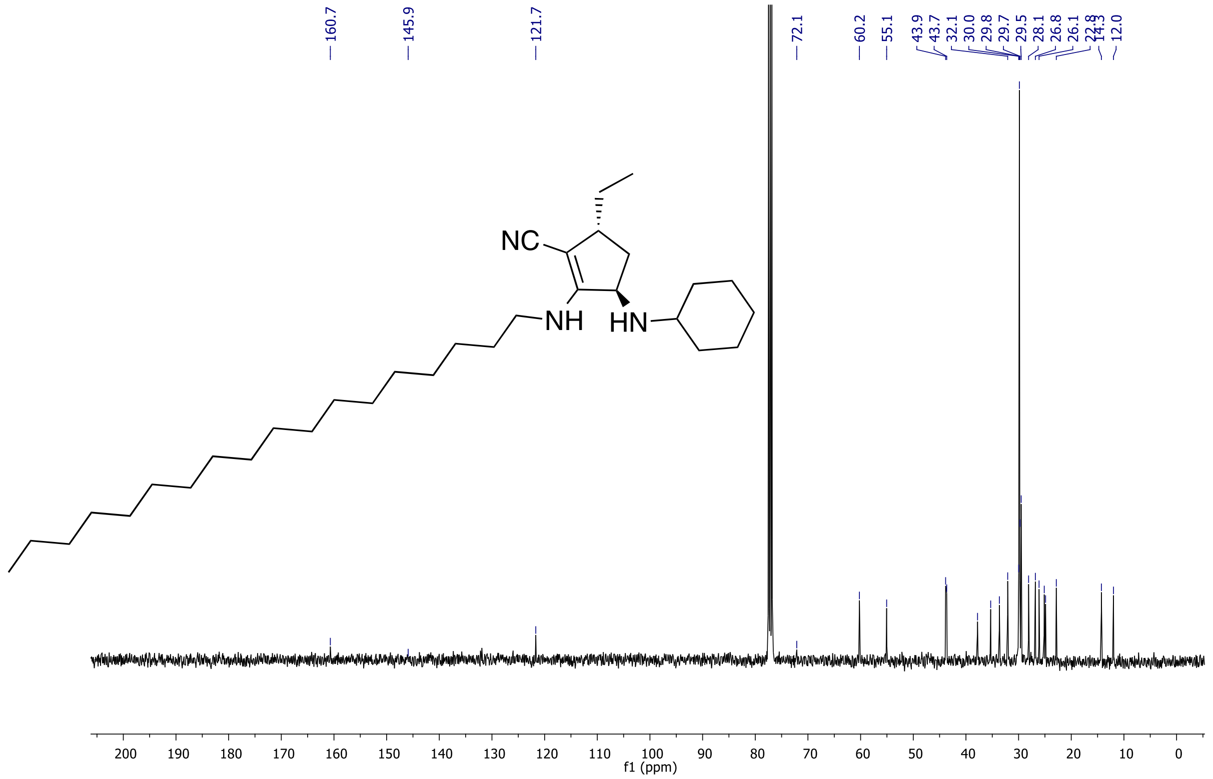

Figure 51. First submission ^{13}C NMR spectrum in $CDCl_3$ of compound **2aj**.

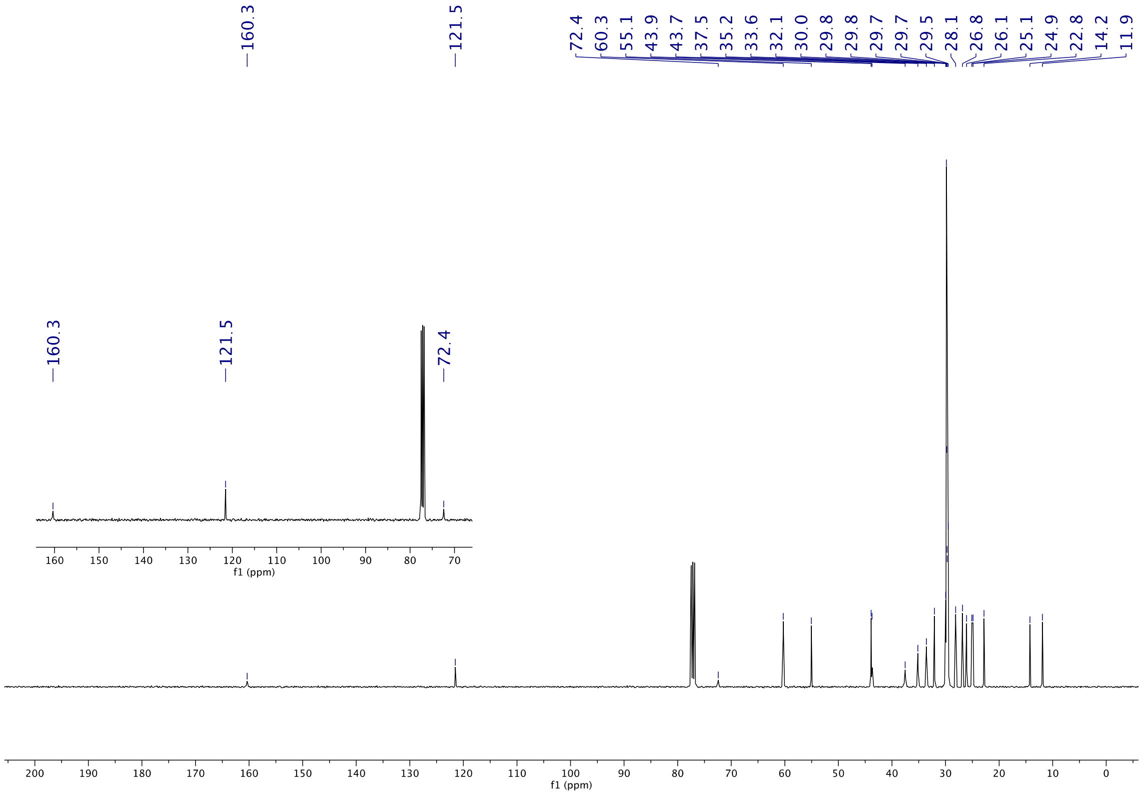

Figure 52. New submission ^{13}C NMR spectrum in $CDCl_3$ of compound **2aj**.

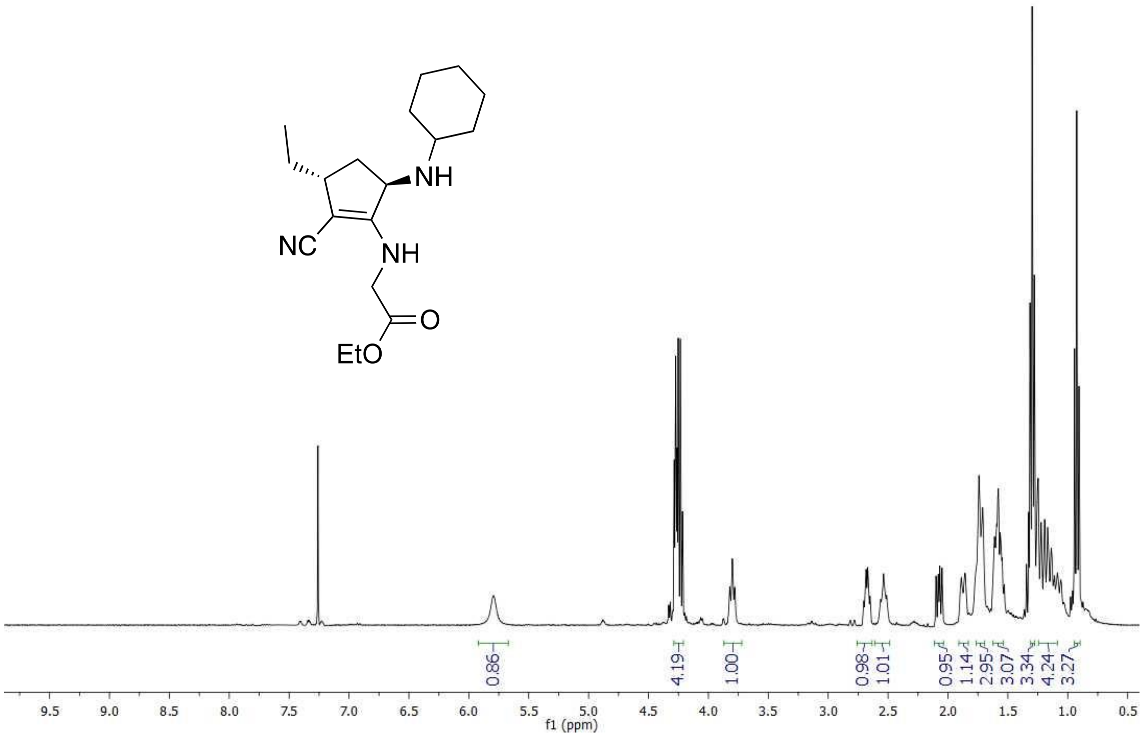

Figure 53. First submission ^{1}H NMR spectrum in $CDCl_3$ of compound **2ak**.

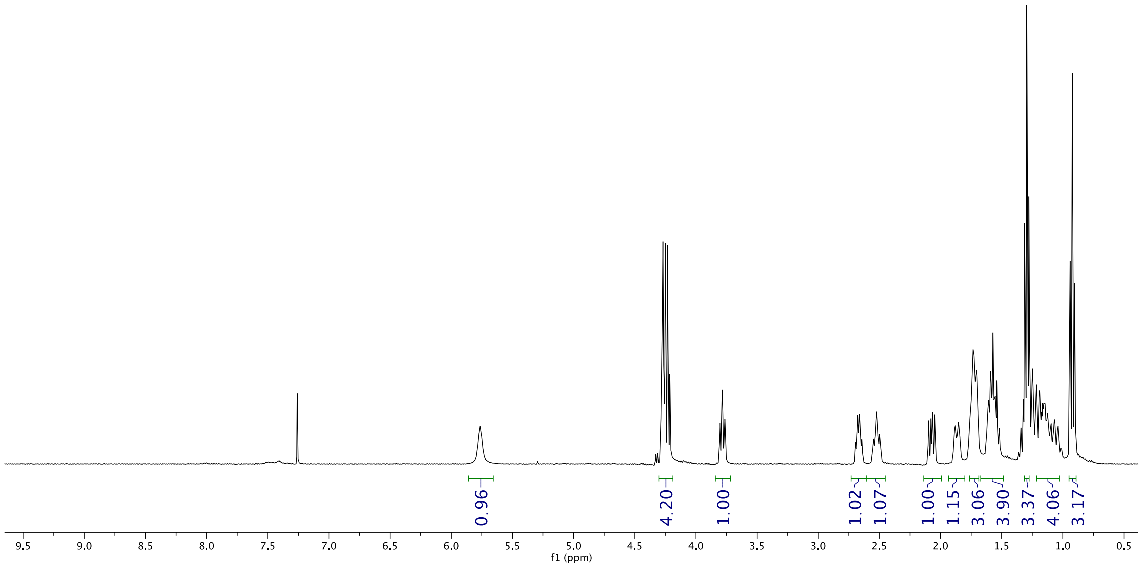

Figure 54. New submission ^{1}H NMR spectrum in $CDCl_3$ of compound **2ak**.

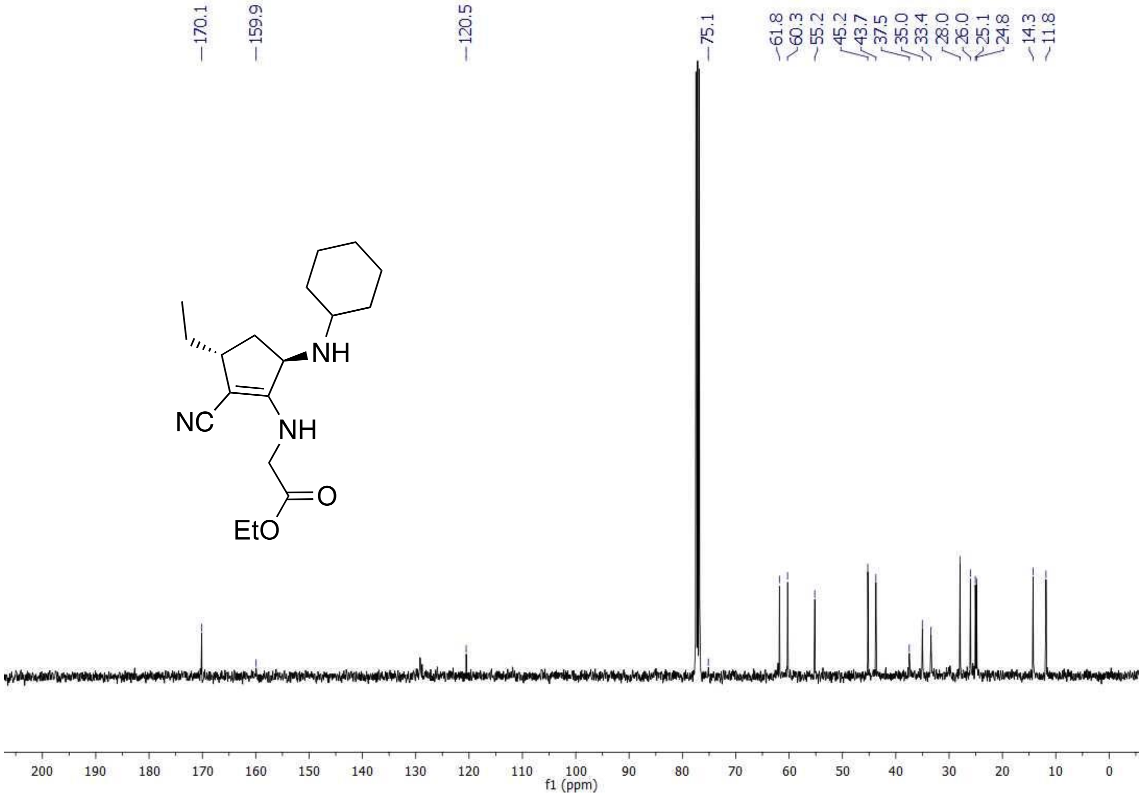

Figure 55. First submission ^{13}C NMR spectrum in $CDCl_3$ of compound **2ak**.

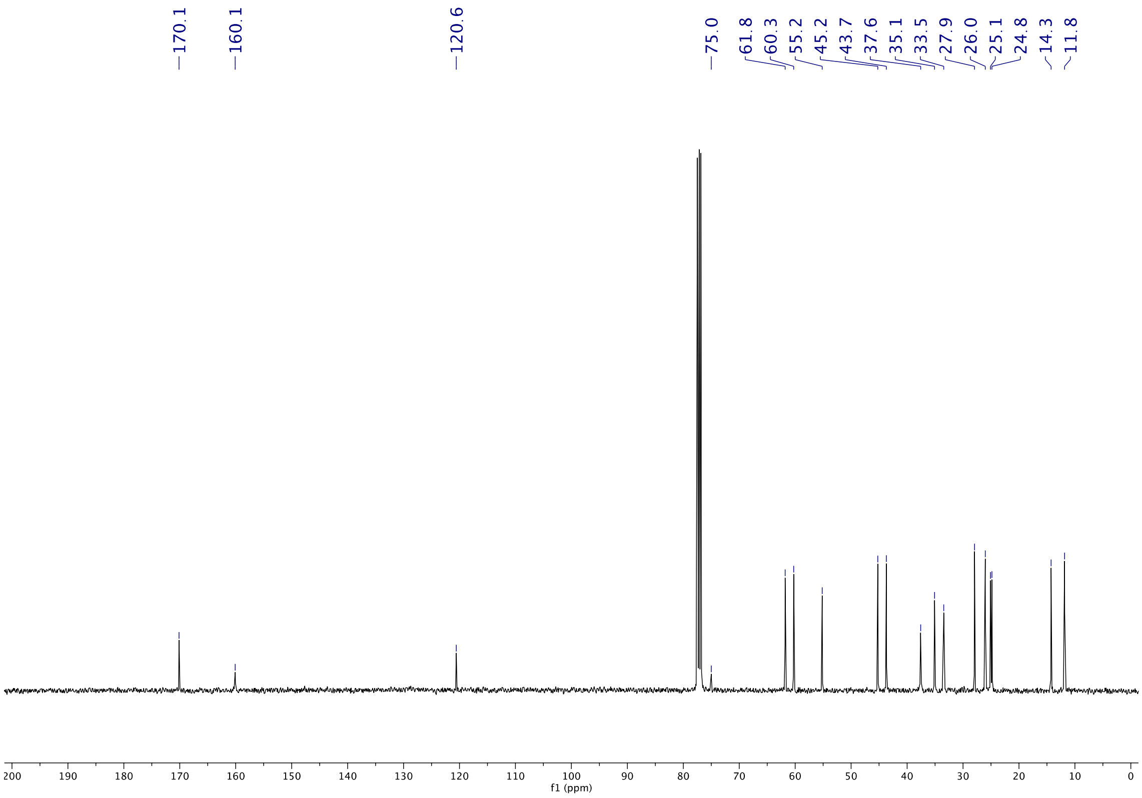

Figure 56. New submission ^{13}C NMR spectrum in $CDCl_3$ of compound **2ak**.
